# DNA barcoding of North American freshwater copepods (Diaptomidae and Cyclopoida): an overview after 20 years with emphasis in the Mexican fauna, the transition between the Nearctic and Neotropics

**DOI:** 10.7717/peerj.20989

**Published:** 2026-04-09

**Authors:** Manuel Elías-Gutiérrez, Eduardo Suárez-Morales, Alma E. Garcia-Morales, José Angel Cohuo-Colli

**Affiliations:** Departamento de Sistemática y Ecología Acuática, Zooplancton y Oceanografía, El Colegio de la Frontera Sur, Unidad Chetumal, Chetumal, Quintana Roo, Mexico

**Keywords:** Diaptomidae, Cyclopidae, Biogeography, COI, Canada, United States, Mexico

## Abstract

**Background:**

In 2003, Paul Hebert proposed DNA barcoding, based on the first half of a standardized gene, the Cytochrome c Oxidase subunit I (COI), to identify animals. Subsequently, two large-scale projects enabled the sequencing of more than 1.3 million putative species worldwide. Two decades ago, we decided to adopt this approach as a tool to investigate the freshwater zooplankton diversity of Mexican aquatic systems. Several copepod species have been described by us with the aid of this marker, mainly of the family Diaptomidae, particularly of the species-rich genera *Leptodiaptomus* and *Mastigodiaptomus*. We also re-described topotypes of the widespread *M. albuquerquensis* and documented the invasion of exotic cyclopid species.

**Methods:**

In Mexico, we have sequenced the COI of 1,725 free-living freshwater copepods, including 925 diaptomid calanoids and 811 cyclopid cyclopoids, representing up to 43.7% of the total specimens sequenced for North America. To delineate the putative species diversity, we used the Barcode Index Number (BIN) and the Assemble Species by Automatic Partitioning method and compared both. For *Leptodiaptomus* we prepared a Maximum Likelihood (ML) tree, for a detailed analysis.

**Results:**

Our results suggest that central-southeastern Mexico may represent a potential radiation center for speciose diaptomid genera like *Mastigodiaptomus* (15 species), *Leptodiaptomus* (eight species), and *Arctodiaptomus*, which likely constitutes a regional species complex yet to be described. A comparison of Mexican data with that from North America (NA) showed that the only truly widespread copepod species, distributed from Arctic latitudes to the central Mexican plateau, is *Leptodiaptomus sicilis*, while all others have more restricted distributions. From the total specimens sequenced in NA, the BIN count revealed 89 Molecular Operational Taxonomic Units (MOTUs), but only 47 of them have been identified to species level. In some cases, diaptomid haplotype variants have received different BINs for a single specimen. The taxonomic impediment appears to be more pronounced in Cyclopoida, with only 32% of the total 235 BINs identified to species level. Despite these limitations, the use of MOTUs from these baselines is valuable for biomonitoring changes in freshwater ecosystems. We found that in some cases, mostly where singletons represented a BIN, the Assemble Species by Automatic Partitioning (ASAP) method provided a better representation of MOTUs. Conversely, when haplotypes of different species, such as those found in the *Leptodiaptomus novamexicanus* complex, are closely similar, ASAP fails, but ML can distinguish them. Therefore, it is urgent to apply an integrative taxonomy approach to propose the most convincing hypotheses regarding these issues. This publicly available online copepod baseline represents a useful tool for exploring and understanding species distributions, detecting possible new species and translocations, and revealing centers of speciation in NA.

## Introduction

In 2003, Prof. Paul Hebert proposed DNA barcoding (Hebert et al., 2003) based on the first half of a standardized gene, the Cytochrome c Oxidase I (COI) aimed to reliably identify animal species. Subsequently, a large project, the International Barcode of Life (iBOL), was launched; it concluded in 2015, after sequencing more than half a million putative species. In 2020, a second project (Bioscan) continued this initiative, with nearly 1,283,000 putative species sequenced worldwide, most of them being insects (see boldsystems.org). We used the term “putative species” because the taxonomic impediment (Raposo et al., 2021) pertains to most specimens sequenced. To temporarily overcome this problem, the Barcode Index Number (BIN) was created to group similar sequences that theoretically would refer to the same species (Ratnasingham & Hebert, 2013). Concurrently, several other methods were devised to reveal the “gap” among putative species (Puillandre, Brouillet & Achaz, 2021; Puillandre et al., 2012), including Bayesian coalescence (Liu et al., 2009; Matz & Nielsen, 2005). The latter requires intense use of computing time but has been successfully used to establish the Molecular Taxonomic Units (MOTUs) in some freshwater copepods (Gutiérrez-Aguirre et al., 2020); however, it is difficult to use it for large data sets.

Despite the enormous advance with DNA barcoding, the aquatic life requires more attention (Elías-Gutiérrez et al., 2021). The first wide study including DNA barcoding of freshwater calanoid copepods (Elías-Gutiérrez et al., 2008) included 21 Neotropical species from Mexico and Guatemala. The authors proposed several cryptic diaptomid species, one of which was described as *Mastigodiaptomus albuquerquensis* (Herrick, 1895) *s.l*. complex, partly disentangled recently (Gutiérrez-Aguirre, Cervantes-Martínez & Elías-Gutiérrez, 2014). Several other species of this complex are yet to be described. The genus *Mastigodiaptomus* Light, 1939 is particularly speciose in Mexican highlands and lowlands (Gutiérrez-Aguirre et al., 2020).

Overall, DNA barcoding methods have been highly successful in the last 20 years; up to 19,658 published article (Web of Science, Topic: DNA and barcod*, consulted on 07/Nov/2021), including more than 1,400 each year since 2020 (Elías-Gutiérrez et al., 2021) have been published. However, a search for copepods (Web of Science, topic: “DNA barcod*” and copepod*, consulted on 07/Nov/2025), showed that the first one was by Elías-Gutiérrez et al. (2008), and only 145 article were published thereafter. Of those, only 35 refer to freshwater copepods (by adding freshwat* to previous search terms, same date), most of them developed by our research team. These figures are disproportionately low in reference to the most abundant aquatic metazoans on Earth.

Since 2008, we have described or re-described up to nine freshwater copepod species, and we had no difficulties in amplifying the COI gene following the suggestions for fixation, preservation and PCR amplification proposed by Elías-Gutiérrez et al. (2008). So far, we have sequenced 1,725 copepod specimens (including some marine species), comprising 925 diaptomid calanoids and 811 cyclopoids from a wide variety of freshwater environments of Mexico. From our results, we infer that central-southeast Mexico has been a possible radiation center for different diaptomid genera, including *Mastigodiaptomus*, with 15 species, *Leptodiaptomus*, with eight species, and *Arctodiaptomus* likely representing a regional complex yet to be described, although it involves haplotypes recognized as different BINs, being the same nominal species (Elías-Gutiérrez et al., 2008; Elías-Gutiérrez, Suárez-Morales & Romano-Márquez, 1999; Gutiérrez-Aguirre et al., 2020; Montiel-Martínez et al., 2008; Jaime et al., in prep).

Some examples of new species described or re-described by us with the aid of the COI gene include *Leptodiaptomus garciai, Mastigodiaptomus patzcuarensis, M. cuneatus, M. siankanensis, M. nesus, M. alexei, M. ha, M. cihuatlan* (see Montiel-Martínez et al., 2008; Gutiérrez-Aguirre, Cervantes-Martínez & Elías-Gutiérrez, 2014; Gutiérrez-Aguirre & Cervantes-Martínez, 2016; Gutiérrez-Aguirre et al., 2020). We obtained, described and sequenced topotypes of key species like *M. albuquerquensis* (Gutiérrez-Aguirre, Cervantes-Martínez & Elías-Gutiérrez, 2014), and we also documented the occurrence of exotic species in Mexico and Spain, like the Asian cyclopoid *Mesocyclops pehpeiensis* Hu, 1943 (Montoliu, Miracle & Elías-Gutiérrez, 2015).

With all the data gathered in this process, we are assembling a regional baseline of copepod sequences (*sensu* Valdez-Moreno et al., 2021), an urgent work to be done in tropical and subtropical freshwater ecosystems, because of the ongoing environmental changes affecting aquatic habitats. For example, *Mastigodiaptomus amatitlanensis* (Wilson, 1941), endemic from Guatemala, could now be extinct by human agency, as well as the recently described *M. galapagoensis* Elías-Gutiérrez, Steinitz-Kannan and Suárez-Morales, 2023 from the Galápagos Archipelago (Elías-Gutiérrez et al., 2023).

Currently, with the aid of the assembled baselines, we can identify and describe species using integrative taxonomy, but we are also developing eDNA methods to biomonitoring the freshwater ecosystems based on the whole zooplankton community. However, for several years the conditions for developing innovative research in Mexico have been far from being favorable (Lazcano, 2019; Reverchon et al., 2025; Reyes-Galindo, 2023; Winkler & Moreno-Ulloa, 2026), thereby negatively affecting our studies as well as other similar research initiatives.

The main goal of this study is to provide an overview and analyze all freshwater Copepoda data from North America (Mexico, USA and Canada) available in the Barcode of Life database (boldsystems.org) to aid in understanding the distribution and taxonomy of the free-living freshwater Calanoida and Cyclopoida processed with DNA barcoding. To emphasize the above, we provide a representative example of a widespread North American complex: *Leptodiaptomus* Light, 1938.

## Materials & Methods

We compiled all sequences (June 10, 2025) of North American Free-living freshwater Copepoda (Calanoida: Diaptomidae and Cyclopoida: Cyclopidae) available in BOLD (boldsystems.org), ours and in public record. We used all public sequences, and we added in the Record Search screen after logging as user in the following fields: Taxonomy: Diaptomidae; Geography: Mexico Canada “United States”. The case *Include public records* at the bottom of the screen was marked. In case of cyclopoids, we replaced the word Diaptomidae with Cyclopoida.

We calculated an Id Tree with the BOLD tools, using a Neighbor joining method and the Two Parameter Kimura Distance Model (Kimura, 1980). For this calculation, we applied a filter to use only sequences >500 bp, excluding records deemed as misidentifications, sequences with stop codons, and with no contaminants. All filters used are explained in the first page of Figs. S1 and S2. From the tree, we recovered the Barcode Index Numbers (BINs) (Ratnasingham & Hebert, 2013) and considered them as putative species. The trees are presented in Figs. S1 and S2. We compared these results with other recent method to discriminate species: Assemble Species by Automatic Partitioning (ASAP) (Puillandre et al., 2021). For ASAP, we aligned sequences with MEGA v7.0 (Kumar, Stecher & Tamura, 2016) using default parameters to obtain a final dataset of 1,701 sequences with 574 bp for Diaptomidae, using a calanid as external group. As for cyclopoids, we gathered 2,264 sequences after alignment. All results were compared with the identifications of the material and the BINs proposed. These results are presented in Figs. S2 and S3, and the number of specimens in each analysis are resumed in Table S1.

**Figure 1:**
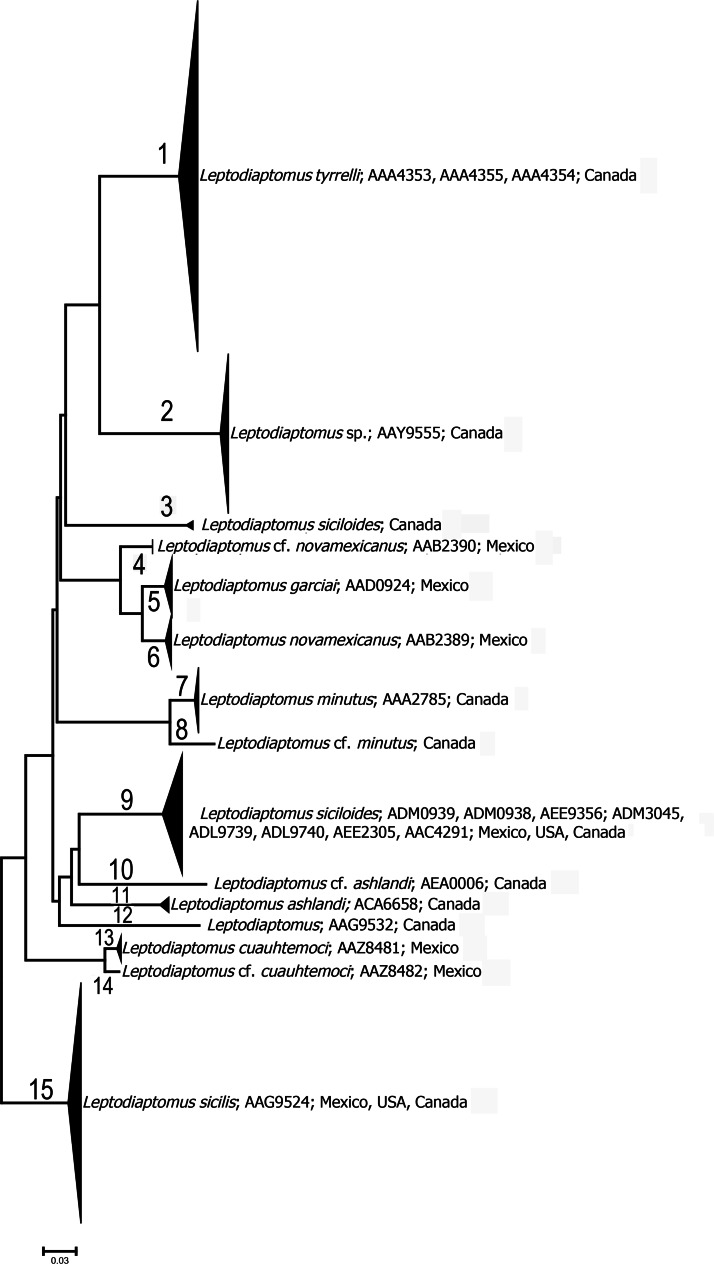
Condensed Id tree of *Leptodiaptomus* based on 674 COI sequences. The numbers after the name correspond to BINs included in each numbered branch. The latter also include the country where the BINs were recorded.

In a second step, we exemplify one complex in need of deeper analyses as an attempt to disentangle the species within it. We worked on a maximum likelihood tree in Mega v7.0 (Kumar, Stecher & Tamura, 2016), with 1,000 replications on *Leptodiaptomus*. In this case the best fit model obtained in the same software was T92+G. Visually, we included in the proposed clusters the singletons for several BINs (see Fig. 1). Finally, for each BIN proposed in a cluster with at least three specimens, we obtained the distance and the standard error (SE) and compared these results with the ASAP results.

Finally, all studied specimens were included in two publicly available datasets (including a Digital Object Identifier, DOI) in BOLD: for Diaptomidae it is dx.doi.org/10.5883/DS-167DIAPOMID, and for Cyclopoida, dx.doi.org/10.5883/DS-CYCLOPNA.

## Results

We analyzed 3,965 sequences of copepods, including 1,701 diaptomids and 2,264 cyclopoids. We recovered 89 diaptomid putative species (MOTUs) (Fig. S1), and 235 for cyclopoids (Fig. S2), supported by the BINs, (Ratnasingham & Hebert, 2013). In reference to the ASAP analyses, the selected split groups were selected after the lowest ASAP score (5.5, with p<.001) and we found 38 MOTUs for diaptomids (Fig. S3), the closest value to the number of species reported for this dataset in the BOLD report (47) (Fig. S1). By contrast, there are 77 species described after morphology from the region (Boxshall & Defaye, 2008). For cyclopoids, we obtained 170 MOTUs after ASAP (Fig. S4), but only 75 species were identified to species level (Fig. S2). In the NA region, there are 114 cyclopids described (Boxshall & Defaye, 2008). In the case of Mexico, the number of species described (117) includes 35 diaptomids and 82 cyclopids (Cervantes-Martínez et al., 2023).

For diaptomids our results are explained by a more detailed taxonomic knowledge of the group, as most taxa were identified to species level, and 67% of the larger clades with at least five individuals accurately correspond with the BIN and the ASAP subset (Fig. S3). The major discordance was found within the nominal species *Arctodiaptomus dorsalis* s.l., for which we found 19 BINs, and only one subset proposed by ASAP in the general analysis (Fig. S1). The main explanation for this effect is that the BIN system starts with 2% divergence (Ratnasingham & Hebert, 2013). Other clusters not distinguished by ASAP were the *Leptodiaptomus novamexicanus* species group, in which *L. garciai*, which inhabits in a saline lake, is a well-recognized species, recently re-described with integrative taxonomy (Montiel-Martínez et al., 2008) and some *Mastigodiaptomus*, as *M. patzcuarensis*, another well-established species after integrative taxonomy (Gutiérrez-Aguirre, Cervantes-Martínez & Elías-Gutiérrez, 2014). Explanation can be that split starts at 2%, and these species evolved rapidly after local adaptation to different environments, then mitochondrial genes do not change at the same speed.

Consequently, it is recommended to adopt an integrative taxonomy approach as in *L. garciai*, and it is also necessary to test the MOTUs, based on different algorithms, and emphasize that these are hypotheses yet to be carefully tested.

In the case of *Arctodiaptomus*, we detected strong differences between ASAP and the BIN system. Most of the different BINs found (15) could be haplotype variations, not separate species, mostly when they are from the same locality. For example, in Lake Bacalar, we found the largest analyzed group with 111 specimens, 79 specimens included in BIN ACE4750, with a mean distance of 0.36 ± 0.0. Additionally, the BIN system included five more, four of them singletons, and another one with 27 (Table 1; Fig. S1). All of them were included in the same cluster by ASAP.

**Table 1:** Comparison of the BINs of *Arctodiaptomus* cf. *dorsalis* in a single locality. Condensed information related the haplotypes found in Lake Bacalar for *Arctodiaptomus dorsalis* (Mexico; 18.70 N, −88.37 W).

Linnean name	Number of cluster	Bins included in cluster	Number of specimens	Distribution	% Distance Mean ± SE
*Arctodiaptomus* cf. *dorsalis*	1	ACE4750	79	Lake Bacalar and associated wetlands, Mexico	0.36 ± 0.0
		ADO0079	1	Lake Bacalar, Mexico	–
		ADD6759	1	Lake Bacalar, Mexico	–
		ADD7671	1	Lake Bacalar, Mexico	–
		ADD8857	1	Lake Bacalar, Mexico	–
		AEM6544	27	Lake Bacalar and associated wetlands, Mexico	1.35 ± 0.01
		Total	110		

The case of *Leptodiaptomus* was represented by 23 BINs, plus two more clades with no BIN, because they lack essential metadata as the trace files, image, and coordinates. Here, we recognized 15 major clades (with more than a singleton), a figure close to that proposed by the ASAP (14) (Table 2; Fig. 1; Figs. S1, S3). *Leptodiaptomus tyrelli* (Poppe, 1888) was represented by 213 specimens and three BINs (Fig. 1), two of them singletons, and one of them coincided with ASAP (AAA4353) (Fig. S1), with 213 individuals. All specimens of this clade, except two, are from a small area near Churchill, north of Manitoba. The two exceptions in the same big clade are from Rock Isle Lake, south of Alberta.

**Table 2:** Condensed information related to Fig. 1. Includes the BINs, a comparison with ASAP method, number of specimens involved in each cluster, distribution and the mean distance among the haplotypes of the same BIN.

Linnean name	BINs included in cluster	Cluster proposed by ASAP	Number of specimens	Distribution	% Distance Mean ± SE
*Leptodiaptomus tyrelli*	AAA4353	7	212	Churchill region, and Near Calgary, Canada	0.3 ± 0.00
	AAA4355	8	1	Churchill region, Canada	–
	AAA4354	7	1	Churchill region, Canada	–
*Leptodiaptomus* sp.	AAY9555	9–10 (one specimen)	98	North of Canada, subarctic	0.001 ± 0.0
*Leptodiaptomus* cf. *siciloides*	NA	6	5	Lake Erie, Canadian side	0.06 ± 0.01
*Leptodiaptomus* cf. *novamexicanus*	AAB2390	5	9	Central Mexico	0.06 ± 0.0
*Leptodiaptomus garciai*	AAD0924	5	37	Central Mexico (endemic, only in four close sites	0.74 ± 0.0
*Leptodiaptomus novamexicanus*	AAB2389	5	29	Central Mexico	0.23 ± 0.0
*Leptodiaptomus minutus*	AAA2785	13	93	USA (north), Canada (south to subarctic)	0.32 ± 0.0
*Leptodiaptomus* cf. *minutus*	NA	12	1	Canada	–
*Leptodiaptomus siciloides*	ADM0939	4	41	Central Mexico, USA, Canada	0.51 ± 0.0
	ADM0938	4	21	North of Mexico, South of USA	0.15 ± 0.0
	AEE9356	4	1	Central Mexico	–
	ADM3045	4	1	Central Mexico	–
	ADL9740	4	1	Central Mexico	–
	AAC4291	4	8	Central, North of Mexico, North USA	0.45 ± 0.02
	AEE2305	4	1	Central Plateau of Mexico	–
*Leptodiaptomus* cf. *ashlandi*	AEA0006	11	1	Huron Lake, Canada side	–
*Leptodiaptomus ashlandi*	ACA6658	3	7	Lake Erie, Canadian side	0.0 ± 0.0
*Leptodiaptomus*	AAG9532	14	7	Canada	0.0 ± 0.0
*Leptodiaptomus cuauhtemoci*	AAZ8481	2	15	Central Mexico, Zempoala lake	0.26 ± 0.0
*Leptodiaptomus cuauhtemoci*	AAZ8482	–	1	Central Mexico, Zempoala lake	–
*Leptodiaptomus sicilis*	AAG9524	1	146	From Central Mexico, USA to Nunavut territory, Canada	1.8 ± 0.0

The other major clade is the AAG9524 belonging to *Leptodiaptomus sicilis* (Forbes, 1882), with 146 specimens (Table 2). This species shows the widest distribution among all other freshwater zooplankters studied by us in North America, with little haplotype variations. The mean distance among all sequences was 1.8 ± 0.0, yet another surprising result, meaning that inter-populations differences are minimal. Its distribution stretches from the Mexican central plateau (2,000 m above the sea level) to the Nunavut territory, in the north of Canada. For this latter case the BIN proposal coincides with the ASAP results (Fig. S13).

Other species with a widespread distribution include *Leptodiaptomus siciloides* (Lilljeborg, 1889), closely related to *L. sicilis*, represented by 77 specimens in the ASAP and eight BINs, forming a large single clade (Fig. 1, Table 2) and also distributed from the central plateau of Mexico to Canada. In this case, four BINs were singletons, ADM3045, ADL9740, AEE9356 and AEE2305). The BIN ADL9739 with seven individuals was found from Albuquerque in the United States to Lake Erie, the two remaining being different widespread haplotypes. The *L. siciloides* AAC4291, with seven specimens is distributed from the central plateau to the north of Mexico. The BIN ADM0938, represented by 21 specimens in the same clade (Fig. 1) from northern Mexico (Sonora state) to California, New Mexico, and Texas (Table 2). The most widespread BIN from this clade was ADM0939, ranging from the Mexican central plateau to southern Canada, and from the center to the eastern USA, with four northernmost specimens found near Hudson Bay in Canada (Table 2, Fig. S2).

In reference to cyclopid copepods, we found a major discordance between the proposed BINs (235) and the ASAP subsets (170, Split Score 5.0, *p* < .001) (Fig. S4). The number of singletons were 42 (out of 235), and 23 duotones totaling 65 putative species under-represented. As in calanoids, the distribution of each major clade was restricted with few exceptions: *Eucyclops* sp., with BIN ABW5480 (with 28 specimens), was found from the Mexican central plateau (17 specimens) to Lake Erie and Guelph Lake in Canada (11 specimens). This BIN fully agrees with ASAP result. Other widespread BIN is AAG9230, with 41 specimens, distributed from central Mexico to southern Canada and from east to west in the United States. In this case ASAP formed a single group with *Mesocyclops edax* from Mexico (BINs AEI4896 and AEI4895) and BIN AEI4894 from Canada. Furthermore, the identification as *Cyclops* for the material from Canada and the USA could be erroneous for 43 specimens (and two from Mexico) whose mean genetic distance was 2.12 ± 0.0. These cases need further investigation to clarify this point.

*Diacyclops thomasi* (Forbes, 1882), represented by seven BINs (ACY4297, ADV7940, ADV7941, AAV0657, AEF5007, AEF3849, and AVV0661) is another large clade of related specimens forming one subset in ASAP, and spreading from the USA to Canada, absent in Mexico. Then, all these singletons could be deemed as haplotypes of the same species.

*Mesocyclops pehpeiensis*, with the BIN ABA8110, is an exotic species in America, and it was found in Mexico and the USA (Suárez-Morales et al., 2005), but there are also confirmed records from Spain (Montoliu, Miracle & Elías-Gutiérrez, 2015). It has been probably dispersed by human agency.

*Acanthocyclops americanus* (Marsh, 1893) (BIN AAG9784, 121 specimens) was found from the Mexican central plateau to southern Canada. This taxon could be the most widespread cyclopoid in the continent, but it has been introduced in several places, including Europe and Russia (Alekseev et al., 2021).

*Macrocyclops albidus* (Jurine, 1820) appears to be yet another widespread species, which in terms of ASAP it was found in the same subset of BINs AAZ8506, ACX1052, and the singleton AAV0648, with a total of 77 specimens. Undoubtedly, *Macrocyclops* Claus, 1893 represents a complex of related species, but translocation among them likely exists (Karanovic & Krajicek, 2012).

Finally, another possible complex is represented by *Tropocyclops prasinus* (Fischer, 1860) related species, with 12 BINs (Fig. S4) and nine subsets by ASAP, widely distributed in North America.

## Discussion

Based on our observations we consider that DNA barcoding is an excellent tool to explore the freshwater copepod diversity, not only to identify copepod species. However, it requires at least three specimens sequenced, and as many as possible to obtain accurate species identifications.

All detected differences between the two methods used (BINs and ASAP) result mainly from the presence of singletons or duotones both in calanoids and cyclopoids, with one exception, *Mesocyclops edax*, represented by two large BIN clades (AAG9230 and AEI4896). Part of the material from Canada (15 specimens), the United States (27 specimens), and Mexico (two specimens), in the latter case misidentified as *Cyclops* (Fig. S4), but in Canada and United States likely corresponding to one of the few species of the genus reported from the Great Lakes Basin (Connolly et al., 2022).

Having access to this kind of data pertaining to the same species from different habitats and geographic regions delivers an instant sketch of its distribution and haplotype variability, including possible translocations as well. As we can see from the Id Trees (Figs. S1 and S2), most species have restricted distributions, a pattern previously noticed by Elías-Gutiérrez et al. (2018), who examined material from Mexico and Canada. The dispersal of northern species onto Mexico can take place only through the central plateau, one of the world’s largest and highest in the tropics, reaching up to 3,000 m, with volcano peaks above 4,000 m, where climate conditions during most of the year resemble those of temperate latitudes, corresponding to a moderately dry climate (Alcocer & Bernal-Brooks, 2010). The two coastal plains are limited by two long mountain chains, and the southern limit of the Mexican Plateau is the Mexican Volcanic Belt. All these geographical features, including the highest peak of North America, the Pico de Orizaba volcano or Citlaltepetl (5,636 m) promote an enormous habitat diversity ranging from semi-arid deserts to humid tropical lowlands. An example of these unique ecosystems is the fauna found in two lakes inside the crater of another volcano, Nevado de Toluca (4,680 m) (Cervantes-Martínez, Gutiérrez-Aguirre & Elías-Gutiérrez, 2000; Sinev & Zawisza, 2013), with several possibly endemic species, including some copepods (Suárez-Morales, Barrera & Ciros-Pérez, 2013).

### Differences between North and South North America

Although Mexico is a usual destination and/or part of the main migration routes of many species of waterfowl (Severo, Lopez-Lopez & Stanley, 2002), we believe that invertebrates propagules carried in their feathers or gut, do not find adequate conditions to establish in most of the aquatic ecosystems in this region, although many of these birds breed in Mexico (Navarro-Sigüenza et al., 2014). Among the main limiting factors we found for the successful establishment of non-tropical planktonic species, we can mention that conditions of all karstic environments of the Yucatan Peninsula and the western Caribbean, a hotspot of fish and crustacean diversity (Gutiérrez-Aguirre, Cervantes-Martínez & Suárez-Morales, 2025): (1) high salinity and high temperatures (Carrillo et al., 2024; Perry, Velazquez-Oliman & Marín, 2002), (2) oligotrophy (Yáñez-Montalvo et al., 2020), (3) predation by fish year-round, mainly by the young stages (Poot-Lopez et al., 2009 and (4) competence (Cervantes-Martínez & Gutiérrez-Aguirre, 2015), are the main limiting factors. For example, Sousa et al. (2017) concluded that the southeast of Mexico was suitable for colonization by the African cladoceran *Daphnia lumholtzi* Sars, 1885, a species that had already colonized eastern USA, from the Great Lakes to Texas (Tudorancea, Bowen & Gerlofsma, 2009); contrastingly, this species has been recorded from a few sites of northern Mexico (Elías-Gutiérrez et al., 2008; Silva-Briano et al., 2010), but not from tropical areas.

The only exception is *L. sicilis*, previously known with a wider distribution in arctic freshwater ecosystems (Samchyshyna, Hansson & Christoffersen, 2008) and found from the Arctic to the central plateau of Mexico, but not found in the true tropical lowlands. We have not found other diaptomid with such a wide distribution.

We clearly found a better consistency between the ASAP subsets and the known taxonomy in reference to the diaptomid copepods. This results from a better understanding of their diversity and abundance in the samples, thus making it easier to process a larger number of specimens from each population. The difference with the BINs is mainly related to genera as *Arctodiaptomus*, possibly showing local adaptation processes as it has been observed in other freshwater copepods (Ortega-Mayagoitia et al., 2022), and having different haplotypes, sometimes in the same ecosystem, as Bacalar Lake (Quintana Roo, Mexico), probably derived from passive dispersion. Also, singletons and duotones have a strong influence on the BIN system. We suggest in these cases sequencing more specimens, the use of a secondary marker, and the use of integrative taxonomy. There are some proposals, but they are not recent, and they focus on other limited types of copepods (Karanovic & Krajicek, 2012).

The case of *Arctodiaptomus* cf. *dorsalis* is in urgent need of clarification. Here, we highlight the relevance of vouchers deposited in a public collection and the conservation of original samples. This group of species closely related to *A. dorsalis* is distributed from Guatemala, where *Arctodiaptomus dampfi* (Brehm, 1932) was described twice (Brehm, 1932; Brehm, 1939) in Lake Petén. Subsequently Suárez-Morales & Elías-Gutiérrez (2001) synonymized it with *A. dorsalis*. However, the COI haplotypes indicate at least nine possible species and the ASAP and BIN probably failed, showing 19 and 20 to 40 possible species. *Arctodiaptomus* is particularly speciose around the world with more than 80 species described, and maybe it does not involve a single genus. Apart from North America, in BOLD we found only 22 specimens of four identified species, and one not identified from Europe and Russia. Moreover, with exception of three specimens of *Arctodiaptomus laticeps* Sars, 1863 from Germany, and two *Arctodiaptomus stewartianus* possibly collected from China (mined to BOLD from GenBank; periodically data are mined from this latter database), all others have no sequences. This limited availability of materials makes any other comparison difficult. Among the main problems of GenBank sequences are errors in identifications, incomplete data, and lower quality sequences than BOLD (Zhou et al., 2025).

In case of cyclopoids, the great discordance between the species identified and the MOTUs proposed by both the BIN (235 putative species) and the ASAP (170 subsets) is given by the number of singletons and duotones, being 38% of the putative species like that. More sequences of this limited number of specimens are needed to detect possible haplotypes or sequence edition errors.

For *Leptodiaptomus*, we identified 11 species, excluding singletons: some are restricted in distribution as *Leptodiaptomus garciai* (Osorio-Tafall, 1942), formerly reported as *L. novamexicanus* (Herrick, 1895), and recognized as a unique endemic to saline lakes of the central plateau of Mexico (Montiel-Martínez et al., 2008); ASAP could not discern the three related species in this complex, but they had a different BIN. However, they appeared after the work of Montiel-Martínez et al. (2008), and the ML and morphology allowed distinguishing them. Another widespread species in Canada is *Leptodiaptomus* sp., possibly representing an undescribed species forming a consistent clade with little variation. *L. sicilis* appears to be the most widespread North American freshwater zooplankter, at least when compared with all the other species sequenced. The 144 specimens reported here had little variation, and the Mexican specimens could be a slightly different haplotype, but not a different species. We can consider the specimens collected and sequenced from the Laurentian Great Lakes, particularly the specimens with sequences ZOOPS013-18 to ZOOPS015-18 as topotypes, collected from the type locality (Lake Michigan). These sequenced specimens were available before Ortega-Mayagoitia et al. (2022) study reporting some local adaptation and pointing out the absence of type material. The wide distribution of *L. sicilis* could be explained by various factors: (1) high survival in polluted waters (Forbes, 1882) and hyper-eutrophic lakes (Arts, Evans & Robarts, 1992), (2) Its ability to survive strong predation pressure both by invertebrates (Bourdeau, Pangle & Peacor, 2015; Yan & Pawson, 1997), and vertebrates (Bunnell et al., 2015; Galat et al., 1981), (3) it can live both, below and above the thermocline (Rudstam et al., 2015 and (4) it is tolerant to SO_4_/CO_3_ and variable salinities (Derry, Prepas & Hebert, 2003; Galat et al., 1981). We consider its wide distribution as “natural” due to zoochory, an outcome of its survival abilities, but not by human agency.

Most other known species of *Leptodiaptomus* seem to be much less ubiquitous, like *L. dodsoni*, only known from its type locality and few specimens found in the largest lake of Mexico, Chapala (Elías-Gutiérrez, Suárez-Morales & Romano-Márquez, 1999). Near this site, another likely endemic species was recently discovered (Velázquez-Ornelas, Ayón-Parente & Suárez-Morales, 2026).

The differences observed among the cyclopoid species identified, the BINs and the ASAP results, are explained mainly by the presence of singletons and duotons (38% of all BINs proposed), and the taxonomic impediment. Some clades need a detailed revision, like all *Macrocyclops*, Ergasilidae, and all specimens labeled as Cyclopidae and *Cyclops*. The BOLD database is constantly being revised and modified on its data. We expect that this process will continue to overcome the taxonomic impediment linked to cyclopoids, where an extra effort is urgently needed. The lesser number of taxonomists working with this group, their smaller size than diaptomid copepods, and a classification based only on females difficult the progress on their study, however the DNA barcodes represent a good opportunity to start solving this problem, as part of the integrative taxonomy.

DNA barcoding can also be a powerful tool to detect introduced species. The Asian *Mesocyclops pehpeiensis* has been reported from several locations in Mexico, and other invasive crustaceans like *Daphnia lumholtzi* have been detected with this method. We believe that metabarcoding can be a promising method to spot exotic species, once the taxonomic baselines are properly established. This method has been applied for zooplankton, but lacking previous baselines, results will not have a way to detect false positives or false negatives or misidentifications (Elías-Gutiérrez & Valdez-Moreno, 2023). With fish, metabarcoding provided good results in tropical environments (Valdez-Moreno et al., 2019; Valdez-Moreno et al., 2021), but the fish baseline used in these two studies (with more than 3,000 specimens) has been in development since 2006 (Valdez-Moreno et al., 2009).

Overall, we can state that DNA barcoding provides new insights about the diversity and distribution of continental free-living copepods; however, the number of publications dealing with Copepoda and DNA barcodes are scarce. We believe that, for many researchers, a major problem is linked with successfully amplifying the COI gene. In BOLD, it is notorious that the processing of 2,316 diaptomid specimens for North America resulted in only 1,829 sequences, being 1,740 > 500 bp (consulted on November 7, 2024), representing 75.13% success. As a comparison, the North American Lepidoptera in the same database, in the same date, includes 100,000 specimens with a 99.9% success. This problem is shared with different laboratories visited by us, where the use of different markers like 28S or others is still a standard procedure (Hirai, Shimode & Tsuda, 2013; Karanovic & Krajicek, 2012; Zagoskin et al., 2014). We were able to solve the amplification problem in most freshwater copepods, and currently we have an 80–100% success. We consider that the main problems are the primers used, and the fixation and storing processes, but the related publications had little attention, with only 94 citations for the Zplank primers that are clearly more effective than Folmer (Prosser, Martínez-Arce & Elías-Gutiérrez, 2013) and 44 for the improved protocols study, where we demonstrated that these primers are effective for most animal groups and the care that should be taken for fixation and preservation of copepod samples (Elías-Gutiérrez et al., 2018).

## Conclusions

Our planet is facing a rapid, likely irreversible environmental change with severe potential effects on aquatic habitats. It is urgent to document the freshwater fauna that is highly sensitive to these changes (Elías-Gutiérrez et al., 2025), and the DNA barcoding is one of the best available tools to identify most of the species, their distribution and variability.

We consider it a success that, after 20 years of DNA barcoding, freshwater copepods, North America can now be considered the most advanced region in the study of freshwater calanoids and cyclopoids. Nearly all species, including the MOTUs with only two exceptions, have a restricted range of distribution in this region. We recognize that the main challenge is the need to expand these studies in order to know if this path is real, because most specimens sequenced are from Mexico (925 diaptomids; 811 cyclopoids) and Canada (890 diaptomids; 1,151 cyclopoids). There are only 98 sequences for freshwater diaptomids and 311 for cyclopoids from the USA (consulted on November 7, 2025).

BOLD plays a key role in the knowledge of global biodiversity, allowing rapid access to big data, and with tools promoting a better understanding of the geographic distribution of any species with uploaded DNA sequences. Another task will be the need to keep the process of updating the Linnean species names, adding the possible new species after integrative taxonomy work, which should be based on expert taxonomists’ input for each group.

The construction of a baseline for marine and freshwater copepods is a fundamental step before attempting further analyses such as metabarcoding, where several problems still require attention, such as the presence of NUMTS (Schultz & Hebert, 2022) and the presence of false positives/negatives, among others.

We need to work extensively on all diaptomid Copepoda and taxonomically clarify each case because most of the BINs obtained have a restricted distribution or involve only one or two specimens. With DNA barcodes, we can match males and females at any stage of the life cycle, which may be difficult in some of these taxa, thus allowing new insights on the ecology and biology of these organisms.

We propose expanding this kind of study to all copepods on which barcoding is feasible.

## Supplemental Information

10.7717/peerj.20989/supp-1 Supplemental Information 1 COI tree of Diaptomidae in North America after Kimura 2 Parameter Model Includes1,701 sequences. Each branch shows the Process Id, the country where it was collected, and the BIN assigned to each sequenced specimen.

10.7717/peerj.20989/supp-2 Supplemental Information 2 COI tree of Cyclopidae and Ergasilidae in North America after Kimura 2 Parameter Model Includes 2,269 sequences. Each branch shows the Process Id, the country where it was collected, and the BIN assigned for each sequenced specimen.

10.7717/peerj.20989/supp-3 Supplemental Information 3 Subsets (= putative species) for 1,701 sequences of Diaptomidae after ASAP analysis The colored bars indicate each subset. The lowest score is represented in Rank 1 (third group from right to left). The proposed ID tree is on the right side of the bars. Each terminal branch includes same data as Supplementary Figure 1

10.7717/peerj.20989/supp-4 Supplemental Information 4 Subsets (= putative species) for 2,269 sequences of North American Cyclopidae and Ergasilidae after ASAP analysis The colored bars indicate each subset. The lowest score is represented in the Rank 1 at the top of the bars, being the sixth from right to left. The proposed ID tree is on the right side of the bars. Each terminal branch includes the same data as Supplementary Figure 2

10.7717/peerj.20989/supp-5 Supplemental Information 5 Raw metadata of Diaptomidae downloaded from BOLD The search used was: Tax: Diaptomidae; Geo: Mexico Canada ”United States”; Length¿500 bp. Public Records. Identifiers for each specimen are the same as in all supplementary figures: Process Id.

10.7717/peerj.20989/supp-6 Supplemental Information 6 Raw metadata of Cyclopoida downloaded from BOLD The search used was: Tax: Cyclopoida Ergasilidae Cyclopoida-family_incertae_sedis; Geo: Mexico Canada “United States”; Length > 500 bp. Public Records. Identifiers for each specimen are the same as all supplementary figures: Process Id.

10.7717/peerj.20989/supp-7 Supplemental Information 7 Raw aligned fasta sequences of Diaptomidae Identifiers for each sequence are the same as supplementary figures of Diaptomidae: Process Id. Only sequences with ¿500 bp were selected.

10.7717/peerj.20989/supp-8 Supplemental Information 8 Raw aligned fasta sequences of Cyclopoida Identifiers for each sequence are the same as supplementary figures of Cyclopoida: Process Id. Only sequences with ¿500 bp were selected.

10.7717/peerj.20989/supp-9 Supplemental Information 9 Filters used, and final number of specimens used in the calculations

## Additional Information and Declarations

### DNA Deposition

The following information was supplied regarding the deposition of DNA sequences: Barcode of Life Database.

### Data Availability

The following information was supplied regarding data availability: The DS-DIAPOMID and DS-CYCLOPNA datasets are available at BOLD (Barcode of Life Database): - dx.doi.org/10.5883/DS-DIAPOMID. - dx.doi.org/10.5883/DS-CYCLOPNA.
