# Supplementary material for: DNA barcoding of North American freshwater copepods (Diaptomidae and Cyclopoida): an overview after 20 years with emphasis in the Mexican fauna, the transition between the Nearctic and Neotropics
Source: PeerJ. 2026 Apr 9;14:e20989. doi: 10.7717/peerj.20989 (PMC13070316; doi:10.7717/peerj.20989)
Supplement: Supplemental Information 1 — Includes1,701 sequences. Each branch shows the Process Id, the country where it was collected, and the BIN assigned to each sequenced specimen. [file peerj-14-20989-s001.pdf]

BOLD TaxonID Tree

Title : Tree Result - Search: Tax(Diaptomidae); Geo(Mexico; Canada; United States); Include public records (2288 records returned) (2288 records selected)

Date : 09-Jun-2025

Data Type : Nucleotide

Distance Model : Kimura 2 Parameter

Marker : COI-5P

Colourization : [blue]=Stop Codons [red]=Contamination or misidentification

Label : Process ID

Label : Taxon

Label : Country

Label : Barcode Cluster (BIN)

Filter : length > 500bp only

Filter : exclude records flagged as misidentifications

Filter : exclude records with stop codons

Filter : exclude contaminants

Sequence Count : 1701

Species count : 47

Genus count : 9

Family count : 1

Unidentified : 310

BIN Count : 89

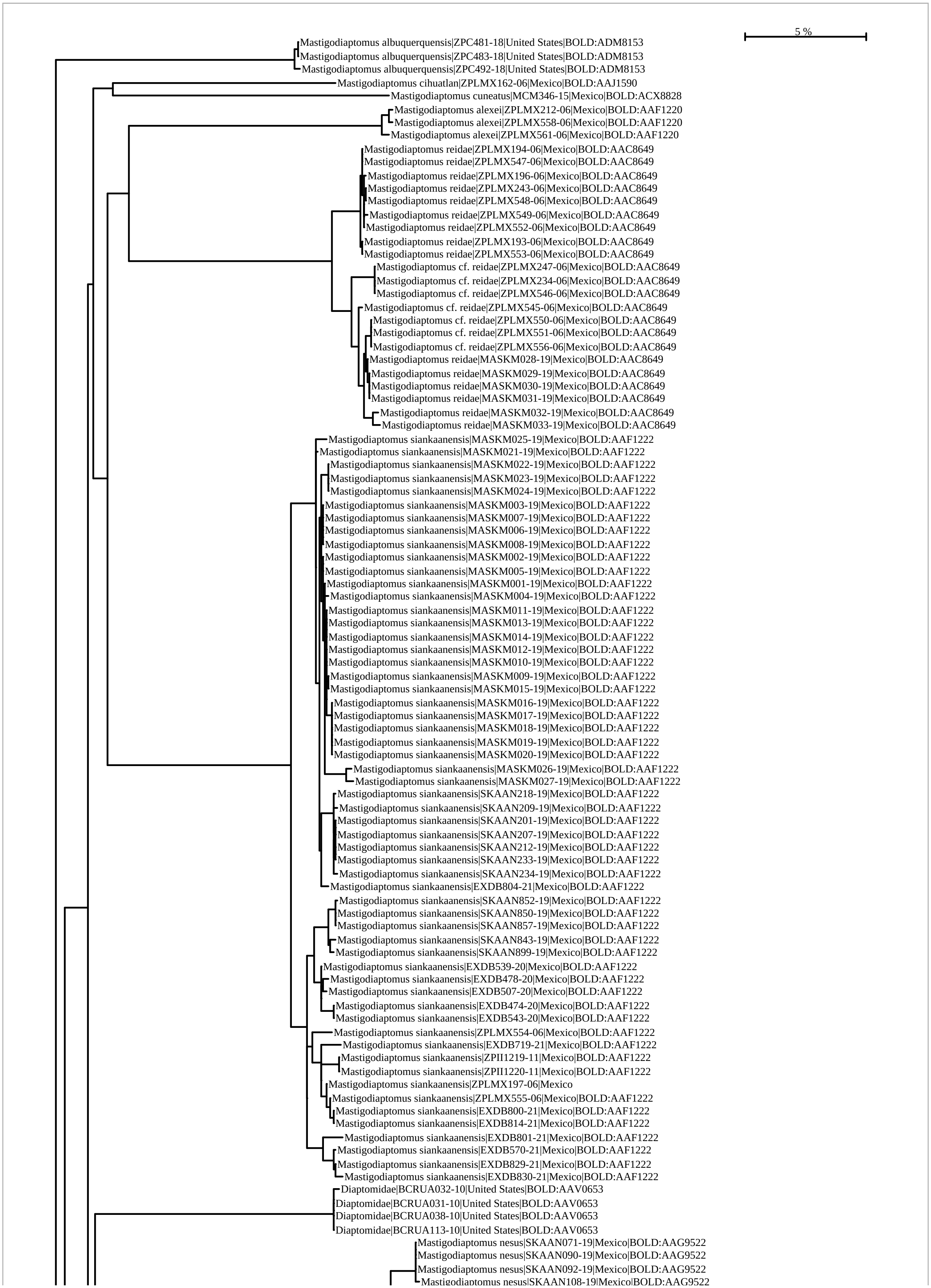
5 %
Mastigodiaptomus albuquerquensis|ZPC481-18|United States|BOLD:ADM8153
Mastigodiaptomus albuquerquensis|ZPC483-18|United States|BOLD:ADM8153
Mastigodiaptomus albuquerquensis|ZPC492-18|United States|BOLD:ADM8153
Mastigodiaptomus cihuatlan|ZPLMX162-06|Mexico|BOLD:AAJ1590
Mastigodiaptomus cuneatus|MCM346-15|Mexico|BOLD:ACX8828
Mastigodiaptomus alexei|ZPLMX212-06|Mexico|BOLD:AAF1220
Mastigodiaptomus alexei|ZPLMX558-06|Mexico|BOLD:AAF1220
Mastigodiaptomus alexei|ZPLMX561-06|Mexico|BOLD:AAF1220
Mastigodiaptomus reidae|ZPLMX194-06|Mexico|BOLD:AAC8649
Mastigodiaptomus reidae|ZPLMX547-06|Mexico|BOLD:AAC8649
Mastigodiaptomus reidae|ZPLMX196-06|Mexico|BOLD:AAC8649
Mastigodiaptomus reidae|ZPLMX243-06|Mexico|BOLD:AAC8649
Mastigodiaptomus reidae|ZPLMX548-06|Mexico|BOLD:AAC8649
Mastigodiaptomus reidae|ZPLMX549-06|Mexico|BOLD:AAC8649
Mastigodiaptomus reidae|ZPLMX552-06|Mexico|BOLD:AAC8649
Mastigodiaptomus reidae|ZPLMX193-06|Mexico|BOLD:AAC8649
Mastigodiaptomus reidae|ZPLMX553-06|Mexico|BOLD:AAC8649
Mastigodiaptomus cf. reidae|ZPLMX247-06|Mexico|BOLD:AAC8649
Mastigodiaptomus cf. reidae|ZPLMX234-06|Mexico|BOLD:AAC8649
Mastigodiaptomus cf. reidae|ZPLMX546-06|Mexico|BOLD:AAC8649
Mastigodiaptomus cf. reidae|ZPLMX545-06|Mexico|BOLD:AAC8649
Mastigodiaptomus cf. reidae|ZPLMX550-06|Mexico|BOLD:AAC8649
Mastigodiaptomus cf. reidae|ZPLMX551-06|Mexico|BOLD:AAC8649
Mastigodiaptomus cf. reidae|ZPLMX556-06|Mexico|BOLD:AAC8649
Mastigodiaptomus reidae|MASKM028-19|Mexico|BOLD:AAC8649
Mastigodiaptomus reidae|MASKM029-19|Mexico|BOLD:AAC8649
Mastigodiaptomus reidae|MASKM030-19|Mexico|BOLD:AAC8649
Mastigodiaptomus reidae|MASKM031-19|Mexico|BOLD:AAC8649
Mastigodiaptomus reidae|MASKM032-19|Mexico|BOLD:AAC8649
Mastigodiaptomus reidae|MASKM033-19|Mexico|BOLD:AAC8649
Mastigodiaptomus siankaanensis|MASKM025-19|Mexico|BOLD:AAF1222
Mastigodiaptomus siankaanensis|MASKM021-19|Mexico|BOLD:AAF1222
Mastigodiaptomus siankaanensis|MASKM022-19|Mexico|BOLD:AAF1222
Mastigodiaptomus siankaanensis|MASKM023-19|Mexico|BOLD:AAF1222
Mastigodiaptomus siankaanensis|MASKM024-19|Mexico|BOLD:AAF1222
Mastigodiaptomus siankaanensis|MASKM003-19|Mexico|BOLD:AAF1222
Mastigodiaptomus siankaanensis|MASKM007-19|Mexico|BOLD:AAF1222
Mastigodiaptomus siankaanensis|MASKM006-19|Mexico|BOLD:AAF1222
Mastigodiaptomus siankaanensis|MASKM008-19|Mexico|BOLD:AAF1222
Mastigodiaptomus siankaanensis|MASKM002-19|Mexico|BOLD:AAF1222
Mastigodiaptomus siankaanensis|MASKM005-19|Mexico|BOLD:AAF1222
Mastigodiaptomus siankaanensis|MASKM001-19|Mexico|BOLD:AAF1222
Mastigodiaptomus siankaanensis|MASKM004-19|Mexico|BOLD:AAF1222
Mastigodiaptomus siankaanensis|MASKM011-19|Mexico|BOLD:AAF1222
Mastigodiaptomus siankaanensis|MASKM013-19|Mexico|BOLD:AAF1222
Mastigodiaptomus siankaanensis|MASKM014-19|Mexico|BOLD:AAF1222
Mastigodiaptomus siankaanensis|MASKM012-19|Mexico|BOLD:AAF1222
Mastigodiaptomus siankaanensis|MASKM010-19|Mexico|BOLD:AAF1222
Mastigodiaptomus siankaanensis|MASKM009-19|Mexico|BOLD:AAF1222
Mastigodiaptomus siankaanensis|MASKM015-19|Mexico|BOLD:AAF1222
Mastigodiaptomus siankaanensis|MASKM016-19|Mexico|BOLD:AAF1222
Mastigodiaptomus siankaanensis|MASKM017-19|Mexico|BOLD:AAF1222
Mastigodiaptomus siankaanensis|MASKM018-19|Mexico|BOLD:AAF1222
Mastigodiaptomus siankaanensis|MASKM019-19|Mexico|BOLD:AAF1222
Mastigodiaptomus siankaanensis|MASKM020-19|Mexico|BOLD:AAF1222
Mastigodiaptomus siankaanensis|MASKM026-19|Mexico|BOLD:AAF1222
Mastigodiaptomus siankaanensis|MASKM027-19|Mexico|BOLD:AAF1222
Mastigodiaptomus siankaanensis|SKAAN218-19|Mexico|BOLD:AAF1222
Mastigodiaptomus siankaanensis|SKAAN209-19|Mexico|BOLD:AAF1222
Mastigodiaptomus siankaanensis|SKAAN201-19|Mexico|BOLD:AAF1222
Mastigodiaptomus siankaanensis|SKAAN207-19|Mexico|BOLD:AAF1222
Mastigodiaptomus siankaanensis|SKAAN212-19|Mexico|BOLD:AAF1222
Mastigodiaptomus siankaanensis|SKAAN233-19|Mexico|BOLD:AAF1222
Mastigodiaptomus siankaanensis|SKAAN234-19|Mexico|BOLD:AAF1222
Mastigodiaptomus siankaanensis|EXDB804-21|Mexico|BOLD:AAF1222
Mastigodiaptomus siankaanensis|SKAAN852-19|Mexico|BOLD:AAF1222
Mastigodiaptomus siankaanensis|SKAAN850-19|Mexico|BOLD:AAF1222
Mastigodiaptomus siankaanensis|SKAAN857-19|Mexico|BOLD:AAF1222
Mastigodiaptomus siankaanensis|SKAAN843-19|Mexico|BOLD:AAF1222
Mastigodiaptomus siankaanensis|SKAAN899-19|Mexico|BOLD:AAF1222
Mastigodiaptomus siankaanensis|EXDB539-20|Mexico|BOLD:AAF1222
Mastigodiaptomus siankaanensis|EXDB478-20|Mexico|BOLD:AAF1222
Mastigodiaptomus siankaanensis|EXDB507-20|Mexico|BOLD:AAF1222
Mastigodiaptomus siankaanensis|EXDB474-20|Mexico|BOLD:AAF1222
Mastigodiaptomus siankaanensis|EXDB543-20|Mexico|BOLD:AAF1222
Mastigodiaptomus siankaanensis|ZPLMX554-06|Mexico|BOLD:AAF1222
Mastigodiaptomus siankaanensis|EXDB719-21|Mexico|BOLD:AAF1222
Mastigodiaptomus siankaanensis|ZPII1219-11|Mexico|BOLD:AAF1222
Mastigodiaptomus siankaanensis|ZPII1220-11|Mexico|BOLD:AAF1222
Mastigodiaptomus siankaanensis|ZPLMX197-06|Mexico
Mastigodiaptomus siankaanensis|ZPLMX555-06|Mexico|BOLD:AAF1222
Mastigodiaptomus siankaanensis|EXDB800-21|Mexico|BOLD:AAF1222
Mastigodiaptomus siankaanensis|EXDB814-21|Mexico|BOLD:AAF1222
Mastigodiaptomus siankaanensis|EXDB801-21|Mexico|BOLD:AAF1222
Mastigodiaptomus siankaanensis|EXDB570-21|Mexico|BOLD:AAF1222
Mastigodiaptomus siankaanensis|EXDB829-21|Mexico|BOLD:AAF1222
Mastigodiaptomus siankaanensis|EXDB830-21|Mexico|BOLD:AAF1222
Diaptomidae|BCRUA032-10|United States|BOLD:AAV0653
Diaptomidae|BCRUA031-10|United States|BOLD:AAV0653
Diaptomidae|BCRUA038-10|United States|BOLD:AAV0653
Diaptomidae|BCRUA113-10|United States|BOLD:AAV0653
Mastigodiaptomus nesus|SKAAN071-19|Mexico|BOLD:AAG9522
Mastigodiaptomus nesus|SKAAN090-19|Mexico|BOLD:AAG9522
Mastigodiaptomus nesus|SKAAN092-19|Mexico|BOLD:AAG9522
Mastigodiaptomus nesus|SKAAN108-19|Mexico|BOLD:AAG9522

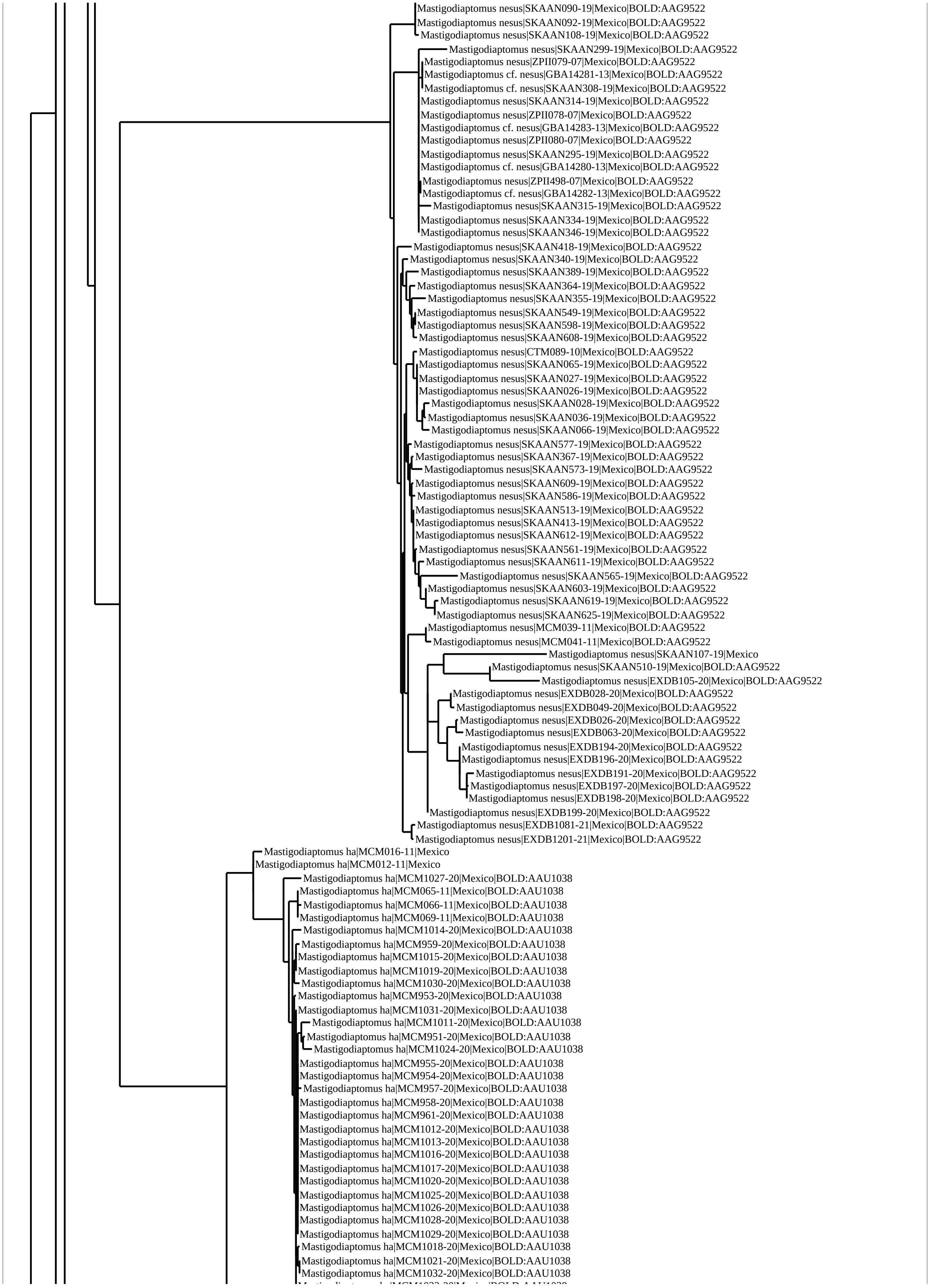
Mastigodiaptomus nesus|SKAAN090-19|Mexico|BOLD:AAG9522
Mastigodiaptomus nesus|SKAAN092-19|Mexico|BOLD:AAG9522
Mastigodiaptomus nesus|SKAAN108-19|Mexico|BOLD:AAG9522
Mastigodiaptomus nesus|SKAAN299-19|Mexico|BOLD:AAG9522
Mastigodiaptomus nesus|ZPII079-07|Mexico|BOLD:AAG9522
Mastigodiaptomus cf. nesus|GBA14281-13|Mexico|BOLD:AAG9522
Mastigodiaptomus cf. nesus|SKAAN308-19|Mexico|BOLD:AAG9522
Mastigodiaptomus nesus|SKAAN314-19|Mexico|BOLD:AAG9522
Mastigodiaptomus nesus|ZPII078-07|Mexico|BOLD:AAG9522
Mastigodiaptomus cf. nesus|GBA14283-13|Mexico|BOLD:AAG9522
Mastigodiaptomus nesus|ZPII080-07|Mexico|BOLD:AAG9522
Mastigodiaptomus nesus|SKAAN295-19|Mexico|BOLD:AAG9522
Mastigodiaptomus cf. nesus|GBA14280-13|Mexico|BOLD:AAG9522
Mastigodiaptomus nesus|ZPII498-07|Mexico|BOLD:AAG9522
Mastigodiaptomus cf. nesus|GBA14282-13|Mexico|BOLD:AAG9522
Mastigodiaptomus nesus|SKAAN315-19|Mexico|BOLD:AAG9522
Mastigodiaptomus nesus|SKAAN334-19|Mexico|BOLD:AAG9522
Mastigodiaptomus nesus|SKAAN346-19|Mexico|BOLD:AAG9522
Mastigodiaptomus nesus|SKAAN418-19|Mexico|BOLD:AAG9522
Mastigodiaptomus nesus|SKAAN340-19|Mexico|BOLD:AAG9522
Mastigodiaptomus nesus|SKAAN389-19|Mexico|BOLD:AAG9522
Mastigodiaptomus nesus|SKAAN364-19|Mexico|BOLD:AAG9522
Mastigodiaptomus nesus|SKAAN355-19|Mexico|BOLD:AAG9522
Mastigodiaptomus nesus|SKAAN549-19|Mexico|BOLD:AAG9522
Mastigodiaptomus nesus|SKAAN598-19|Mexico|BOLD:AAG9522
Mastigodiaptomus nesus|SKAAN608-19|Mexico|BOLD:AAG9522
Mastigodiaptomus nesus|CTM089-10|Mexico|BOLD:AAG9522
Mastigodiaptomus nesus|SKAAN065-19|Mexico|BOLD:AAG9522
Mastigodiaptomus nesus|SKAAN027-19|Mexico|BOLD:AAG9522
Mastigodiaptomus nesus|SKAAN026-19|Mexico|BOLD:AAG9522
Mastigodiaptomus nesus|SKAAN028-19|Mexico|BOLD:AAG9522
Mastigodiaptomus nesus|SKAAN036-19|Mexico|BOLD:AAG9522
Mastigodiaptomus nesus|SKAAN066-19|Mexico|BOLD:AAG9522
Mastigodiaptomus nesus|SKAAN577-19|Mexico|BOLD:AAG9522
Mastigodiaptomus nesus|SKAAN367-19|Mexico|BOLD:AAG9522
Mastigodiaptomus nesus|SKAAN573-19|Mexico|BOLD:AAG9522
Mastigodiaptomus nesus|SKAAN609-19|Mexico|BOLD:AAG9522
Mastigodiaptomus nesus|SKAAN586-19|Mexico|BOLD:AAG9522
Mastigodiaptomus nesus|SKAAN513-19|Mexico|BOLD:AAG9522
Mastigodiaptomus nesus|SKAAN413-19|Mexico|BOLD:AAG9522
Mastigodiaptomus nesus|SKAAN612-19|Mexico|BOLD:AAG9522
Mastigodiaptomus nesus|SKAAN561-19|Mexico|BOLD:AAG9522
Mastigodiaptomus nesus|SKAAN611-19|Mexico|BOLD:AAG9522
Mastigodiaptomus nesus|SKAAN565-19|Mexico|BOLD:AAG9522
Mastigodiaptomus nesus|SKAAN603-19|Mexico|BOLD:AAG9522
Mastigodiaptomus nesus|SKAAN619-19|Mexico|BOLD:AAG9522
Mastigodiaptomus nesus|SKAAN625-19|Mexico|BOLD:AAG9522
Mastigodiaptomus nesus|MCM039-11|Mexico|BOLD:AAG9522
Mastigodiaptomus nesus|MCM041-11|Mexico|BOLD:AAG9522
Mastigodiaptomus nesus|SKAAN107-19|Mexico
Mastigodiaptomus nesus|SKAAN510-19|Mexico|BOLD:AAG9522
Mastigodiaptomus nesus|EXDB105-20|Mexico|BOLD:AAG9522
Mastigodiaptomus nesus|EXDB028-20|Mexico|BOLD:AAG9522
Mastigodiaptomus nesus|EXDB049-20|Mexico|BOLD:AAG9522
Mastigodiaptomus nesus|EXDB026-20|Mexico|BOLD:AAG9522
Mastigodiaptomus nesus|EXDB063-20|Mexico|BOLD:AAG9522
Mastigodiaptomus nesus|EXDB194-20|Mexico|BOLD:AAG9522
Mastigodiaptomus nesus|EXDB196-20|Mexico|BOLD:AAG9522
Mastigodiaptomus nesus|EXDB191-20|Mexico|BOLD:AAG9522
Mastigodiaptomus nesus|EXDB197-20|Mexico|BOLD:AAG9522
Mastigodiaptomus nesus|EXDB198-20|Mexico|BOLD:AAG9522
Mastigodiaptomus nesus|EXDB199-20|Mexico|BOLD:AAG9522
Mastigodiaptomus nesus|EXDB1081-21|Mexico|BOLD:AAG9522
Mastigodiaptomus nesus|EXDB1201-21|Mexico|BOLD:AAG9522
Mastigodiaptomus ha|MCM016-11|Mexico
Mastigodiaptomus ha|MCM012-11|Mexico
Mastigodiaptomus ha|MCM1027-20|Mexico|BOLD:AAU1038
Mastigodiaptomus ha|MCM065-11|Mexico|BOLD:AAU1038
Mastigodiaptomus ha|MCM066-11|Mexico|BOLD:AAU1038
Mastigodiaptomus ha|MCM069-11|Mexico|BOLD:AAU1038
Mastigodiaptomus ha|MCM1014-20|Mexico|BOLD:AAU1038
Mastigodiaptomus ha|MCM959-20|Mexico|BOLD:AAU1038
Mastigodiaptomus ha|MCM1015-20|Mexico|BOLD:AAU1038
Mastigodiaptomus ha|MCM1019-20|Mexico|BOLD:AAU1038
Mastigodiaptomus ha|MCM1030-20|Mexico|BOLD:AAU1038
Mastigodiaptomus ha|MCM953-20|Mexico|BOLD:AAU1038
Mastigodiaptomus ha|MCM1031-20|Mexico|BOLD:AAU1038
Mastigodiaptomus ha|MCM1011-20|Mexico|BOLD:AAU1038
Mastigodiaptomus ha|MCM951-20|Mexico|BOLD:AAU1038
Mastigodiaptomus ha|MCM1024-20|Mexico|BOLD:AAU1038
Mastigodiaptomus ha|MCM955-20|Mexico|BOLD:AAU1038
Mastigodiaptomus ha|MCM954-20|Mexico|BOLD:AAU1038
Mastigodiaptomus ha|MCM957-20|Mexico|BOLD:AAU1038
Mastigodiaptomus ha|MCM958-20|Mexico|BOLD:AAU1038
Mastigodiaptomus ha|MCM961-20|Mexico|BOLD:AAU1038
Mastigodiaptomus ha|MCM1012-20|Mexico|BOLD:AAU1038
Mastigodiaptomus ha|MCM1013-20|Mexico|BOLD:AAU1038
Mastigodiaptomus ha|MCM1016-20|Mexico|BOLD:AAU1038
Mastigodiaptomus ha|MCM1017-20|Mexico|BOLD:AAU1038
Mastigodiaptomus ha|MCM1020-20|Mexico|BOLD:AAU1038
Mastigodiaptomus ha|MCM1025-20|Mexico|BOLD:AAU1038
Mastigodiaptomus ha|MCM1026-20|Mexico|BOLD:AAU1038
Mastigodiaptomus ha|MCM1028-20|Mexico|BOLD:AAU1038
Mastigodiaptomus ha|MCM1029-20|Mexico|BOLD:AAU1038
Mastigodiaptomus ha|MCM1018-20|Mexico|BOLD:AAU1038
Mastigodiaptomus ha|MCM1021-20|Mexico|BOLD:AAU1038
Mastigodiaptomus ha|MCM1032-20|Mexico|BOLD:AAU1038

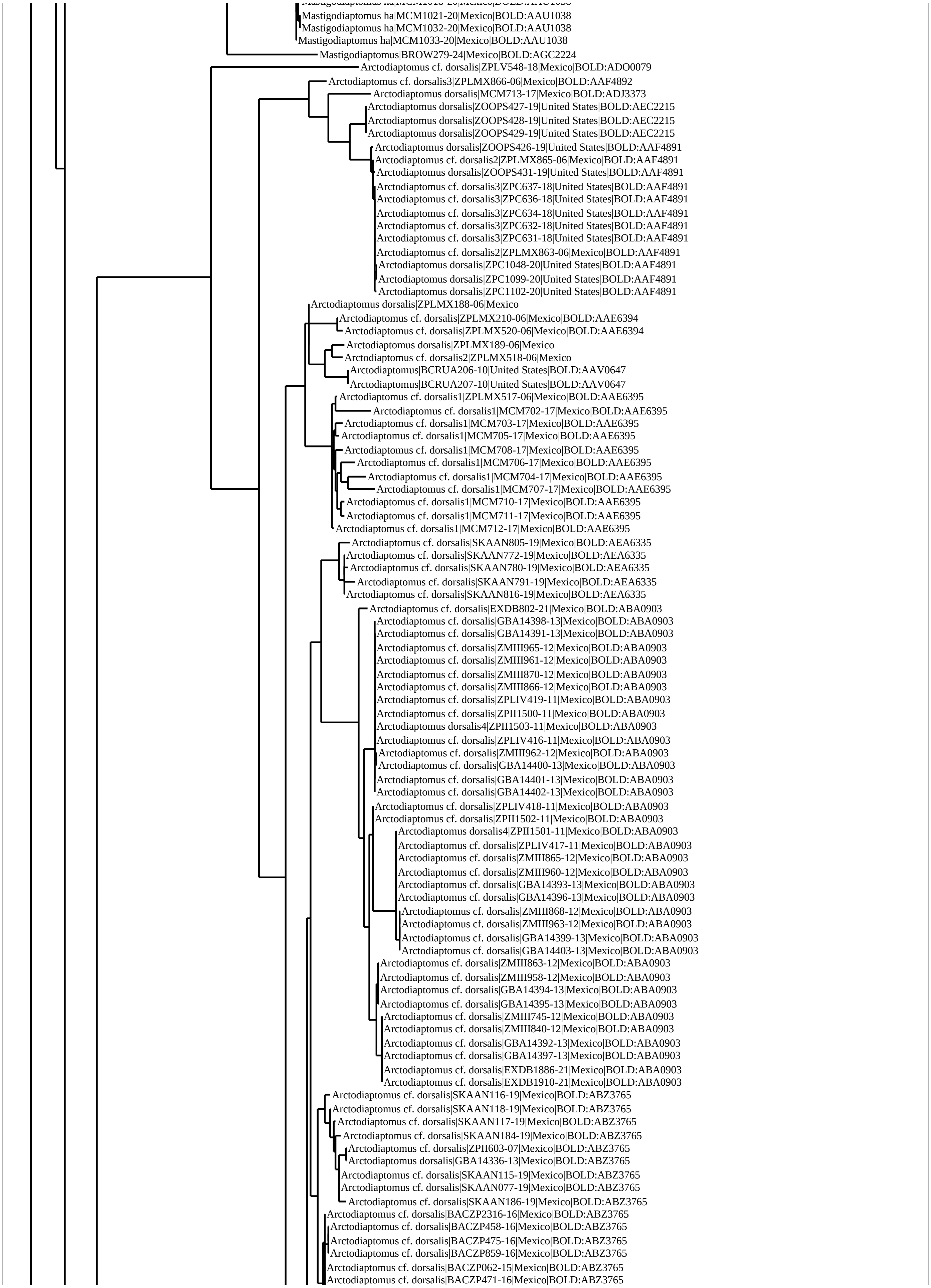
Mastigodiaptomus ha|MCM1021-20|Mexico|BOLD:AAU1038
Mastigodiaptomus ha|MCM1032-20|Mexico|BOLD:AAU1038
Mastigodiaptomus ha|MCM1033-20|Mexico|BOLD:AAU1038
Mastigodiaptomus|BROW279-24|Mexico|BOLD:AGC2224
Arctodiaptomus cf. dorsalis|ZPLV548-18|Mexico|BOLD:ADO0079
Arctodiaptomus cf. dorsalis3|ZPLMX866-06|Mexico|BOLD:AAF4892
Arctodiaptomus dorsalis|MCM713-17|Mexico|BOLD:ADJ3373
Arctodiaptomus dorsalis|ZOOPS427-19|United States|BOLD:AEC2215
Arctodiaptomus dorsalis|ZOOPS428-19|United States|BOLD:AEC2215
Arctodiaptomus dorsalis|ZOOPS429-19|United States|BOLD:AEC2215
Arctodiaptomus dorsalis|ZOOPS426-19|United States|BOLD:AAF4891
Arctodiaptomus cf. dorsalis2|ZPLMX865-06|Mexico|BOLD:AAF4891
Arctodiaptomus dorsalis|ZOOPS431-19|United States|BOLD:AAF4891
Arctodiaptomus cf. dorsalis3|ZPC637-18|United States|BOLD:AAF4891
Arctodiaptomus cf. dorsalis3|ZPC636-18|United States|BOLD:AAF4891
Arctodiaptomus cf. dorsalis3|ZPC634-18|United States|BOLD:AAF4891
Arctodiaptomus cf. dorsalis3|ZPC632-18|United States|BOLD:AAF4891
Arctodiaptomus cf. dorsalis3|ZPC631-18|United States|BOLD:AAF4891
Arctodiaptomus cf. dorsalis2|ZPLMX863-06|Mexico|BOLD:AAF4891
Arctodiaptomus dorsalis|ZPC1048-20|United States|BOLD:AAF4891
Arctodiaptomus dorsalis|ZPC1099-20|United States|BOLD:AAF4891
Arctodiaptomus dorsalis|ZPC1102-20|United States|BOLD:AAF4891
Arctodiaptomus dorsalis|ZPLMX188-06|Mexico
Arctodiaptomus cf. dorsalis|ZPLMX210-06|Mexico|BOLD:AAE6394
Arctodiaptomus cf. dorsalis|ZPLMX520-06|Mexico|BOLD:AAE6394
Arctodiaptomus dorsalis|ZPLMX189-06|Mexico
Arctodiaptomus cf. dorsalis2|ZPLMX518-06|Mexico
Arctodiaptomus|BCRUA206-10|United States|BOLD:AAV0647
Arctodiaptomus|BCRUA207-10|United States|BOLD:AAV0647
Arctodiaptomus cf. dorsalis1|ZPLMX517-06|Mexico|BOLD:AAE6395
Arctodiaptomus cf. dorsalis1|MCM702-17|Mexico|BOLD:AAE6395
Arctodiaptomus cf. dorsalis1|MCM703-17|Mexico|BOLD:AAE6395
Arctodiaptomus cf. dorsalis1|MCM705-17|Mexico|BOLD:AAE6395
Arctodiaptomus cf. dorsalis1|MCM708-17|Mexico|BOLD:AAE6395
Arctodiaptomus cf. dorsalis1|MCM706-17|Mexico|BOLD:AAE6395
Arctodiaptomus cf. dorsalis1|MCM704-17|Mexico|BOLD:AAE6395
Arctodiaptomus cf. dorsalis1|MCM707-17|Mexico|BOLD:AAE6395
Arctodiaptomus cf. dorsalis1|MCM710-17|Mexico|BOLD:AAE6395
Arctodiaptomus cf. dorsalis1|MCM711-17|Mexico|BOLD:AAE6395
Arctodiaptomus cf. dorsalis1|MCM712-17|Mexico|BOLD:AAE6395
Arctodiaptomus cf. dorsalis|SKAAN805-19|Mexico|BOLD:AEA6335
Arctodiaptomus cf. dorsalis|SKAAN772-19|Mexico|BOLD:AEA6335
Arctodiaptomus cf. dorsalis|SKAAN780-19|Mexico|BOLD:AEA6335
Arctodiaptomus cf. dorsalis|SKAAN791-19|Mexico|BOLD:AEA6335
Arctodiaptomus cf. dorsalis|SKAAN816-19|Mexico|BOLD:AEA6335
Arctodiaptomus cf. dorsalis|EXDB802-21|Mexico|BOLD:ABA0903
Arctodiaptomus cf. dorsalis|GBA14398-13|Mexico|BOLD:ABA0903
Arctodiaptomus cf. dorsalis|GBA14391-13|Mexico|BOLD:ABA0903
Arctodiaptomus cf. dorsalis|ZMIII965-12|Mexico|BOLD:ABA0903
Arctodiaptomus cf. dorsalis|ZMIII961-12|Mexico|BOLD:ABA0903
Arctodiaptomus cf. dorsalis|ZMIII870-12|Mexico|BOLD:ABA0903
Arctodiaptomus cf. dorsalis|ZMIII866-12|Mexico|BOLD:ABA0903
Arctodiaptomus cf. dorsalis|ZPLIV419-11|Mexico|BOLD:ABA0903
Arctodiaptomus cf. dorsalis|ZPII1500-11|Mexico|BOLD:ABA0903
Arctodiaptomus dorsalis4|ZPII1503-11|Mexico|BOLD:ABA0903
Arctodiaptomus cf. dorsalis|ZPLIV416-11|Mexico|BOLD:ABA0903
Arctodiaptomus cf. dorsalis|ZMIII962-12|Mexico|BOLD:ABA0903
Arctodiaptomus cf. dorsalis|GBA14400-13|Mexico|BOLD:ABA0903
Arctodiaptomus cf. dorsalis|GBA14401-13|Mexico|BOLD:ABA0903
Arctodiaptomus cf. dorsalis|GBA14402-13|Mexico|BOLD:ABA0903
Arctodiaptomus cf. dorsalis|ZPLIV418-11|Mexico|BOLD:ABA0903
Arctodiaptomus cf. dorsalis|ZPII1502-11|Mexico|BOLD:ABA0903
Arctodiaptomus dorsalis4|ZPII1501-11|Mexico|BOLD:ABA0903
Arctodiaptomus cf. dorsalis|ZPLIV417-11|Mexico|BOLD:ABA0903
Arctodiaptomus cf. dorsalis|ZMIII865-12|Mexico|BOLD:ABA0903
Arctodiaptomus cf. dorsalis|ZMIII960-12|Mexico|BOLD:ABA0903
Arctodiaptomus cf. dorsalis|GBA14393-13|Mexico|BOLD:ABA0903
Arctodiaptomus cf. dorsalis|GBA14396-13|Mexico|BOLD:ABA0903
Arctodiaptomus cf. dorsalis|ZMIII868-12|Mexico|BOLD:ABA0903
Arctodiaptomus cf. dorsalis|ZMIII963-12|Mexico|BOLD:ABA0903
Arctodiaptomus cf. dorsalis|GBA14399-13|Mexico|BOLD:ABA0903
Arctodiaptomus cf. dorsalis|GBA14403-13|Mexico|BOLD:ABA0903
Arctodiaptomus cf. dorsalis|ZMIII863-12|Mexico|BOLD:ABA0903
Arctodiaptomus cf. dorsalis|ZMIII958-12|Mexico|BOLD:ABA0903
Arctodiaptomus cf. dorsalis|GBA14394-13|Mexico|BOLD:ABA0903
Arctodiaptomus cf. dorsalis|GBA14395-13|Mexico|BOLD:ABA0903
Arctodiaptomus cf. dorsalis|ZMIII745-12|Mexico|BOLD:ABA0903
Arctodiaptomus cf. dorsalis|ZMIII840-12|Mexico|BOLD:ABA0903
Arctodiaptomus cf. dorsalis|GBA14392-13|Mexico|BOLD:ABA0903
Arctodiaptomus cf. dorsalis|GBA14397-13|Mexico|BOLD:ABA0903
Arctodiaptomus cf. dorsalis|EXDB1886-21|Mexico|BOLD:ABA0903
Arctodiaptomus cf. dorsalis|EXDB1910-21|Mexico|BOLD:ABA0903
Arctodiaptomus cf. dorsalis|SKAAN116-19|Mexico|BOLD:ABZ3765
Arctodiaptomus cf. dorsalis|SKAAN118-19|Mexico|BOLD:ABZ3765
Arctodiaptomus cf. dorsalis|SKAAN117-19|Mexico|BOLD:ABZ3765
Arctodiaptomus cf. dorsalis|SKAAN184-19|Mexico|BOLD:ABZ3765
Arctodiaptomus cf. dorsalis|ZPII603-07|Mexico|BOLD:ABZ3765
Arctodiaptomus dorsalis|GBA14336-13|Mexico|BOLD:ABZ3765
Arctodiaptomus cf. dorsalis|SKAAN115-19|Mexico|BOLD:ABZ3765
Arctodiaptomus cf. dorsalis|SKAAN077-19|Mexico|BOLD:ABZ3765
Arctodiaptomus cf. dorsalis|SKAAN186-19|Mexico|BOLD:ABZ3765
Arctodiaptomus cf. dorsalis|BACZP2316-16|Mexico|BOLD:ABZ3765
Arctodiaptomus cf. dorsalis|BACZP458-16|Mexico|BOLD:ABZ3765
Arctodiaptomus cf. dorsalis|BACZP475-16|Mexico|BOLD:ABZ3765
Arctodiaptomus cf. dorsalis|BACZP859-16|Mexico|BOLD:ABZ3765
Arctodiaptomus cf. dorsalis|BACZP062-15|Mexico|BOLD:ABZ3765
Arctodiaptomus cf. dorsalis|BACZP471-16|Mexico|BOLD:ABZ3765

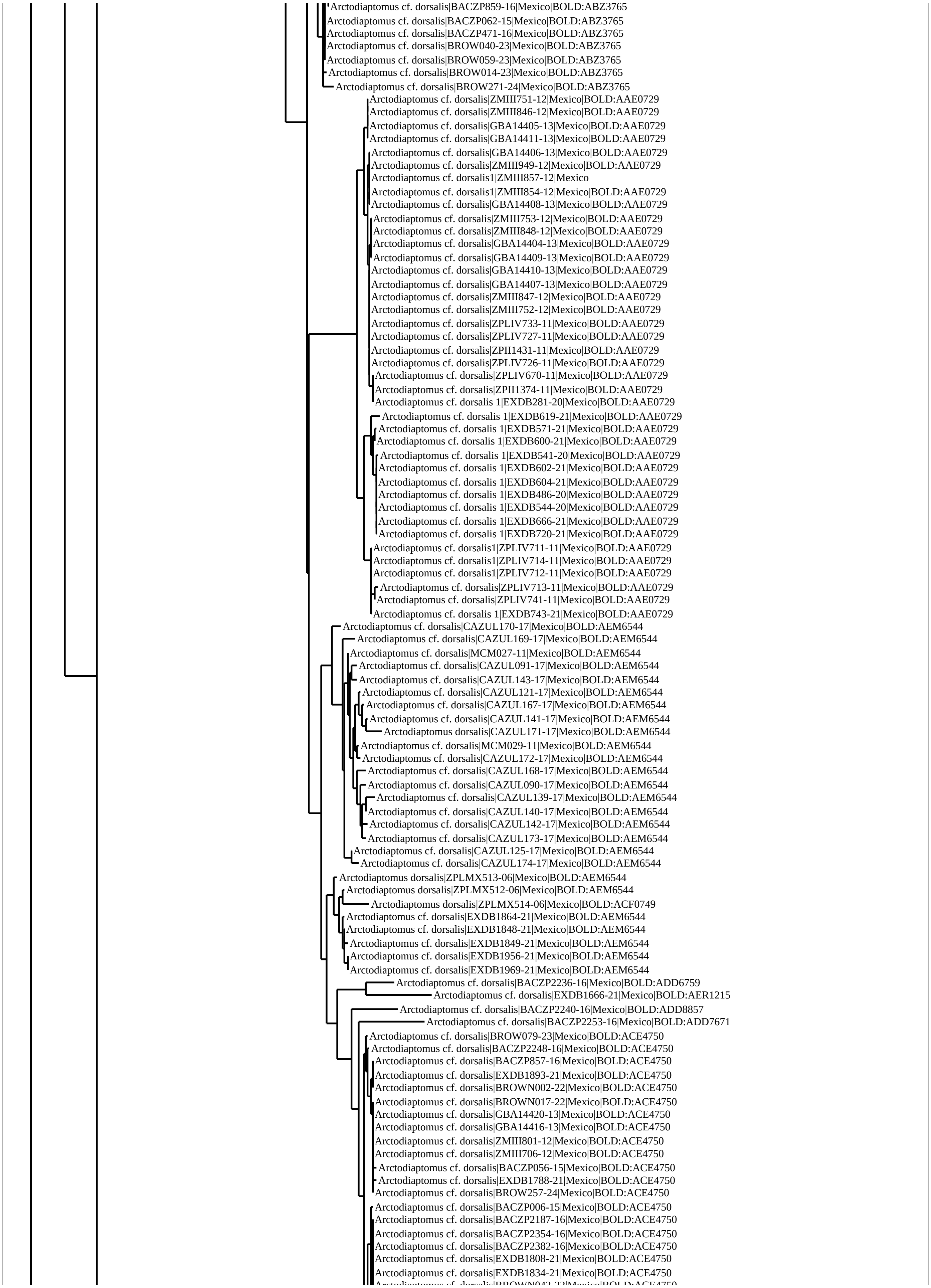

Arctodiaptomus cf. dorsalis|BACZP859-16|Mexico|BOLD:ABZ3765
Arctodiaptomus cf. dorsalis|BACZP062-15|Mexico|BOLD:ABZ3765
Arctodiaptomus cf. dorsalis|BACZP471-16|Mexico|BOLD:ABZ3765
Arctodiaptomus cf. dorsalis|BROW040-23|Mexico|BOLD:ABZ3765
Arctodiaptomus cf. dorsalis|BROW059-23|Mexico|BOLD:ABZ3765
Arctodiaptomus cf. dorsalis|BROW014-23|Mexico|BOLD:ABZ3765
Arctodiaptomus cf. dorsalis|BROW271-24|Mexico|BOLD:ABZ3765
Arctodiaptomus cf. dorsalis|ZMIII751-12|Mexico|BOLD:AAE0729
Arctodiaptomus cf. dorsalis|ZMIII846-12|Mexico|BOLD:AAE0729
Arctodiaptomus cf. dorsalis|GBA14405-13|Mexico|BOLD:AAE0729
Arctodiaptomus cf. dorsalis|GBA14411-13|Mexico|BOLD:AAE0729
Arctodiaptomus cf. dorsalis|GBA14406-13|Mexico|BOLD:AAE0729
Arctodiaptomus cf. dorsalis|ZMIII949-12|Mexico|BOLD:AAE0729
Arctodiaptomus cf. dorsalis1|ZMIII857-12|Mexico
Arctodiaptomus cf. dorsalis1|ZMIII854-12|Mexico|BOLD:AAE0729
Arctodiaptomus cf. dorsalis|GBA14408-13|Mexico|BOLD:AAE0729
Arctodiaptomus cf. dorsalis|ZMIII753-12|Mexico|BOLD:AAE0729
Arctodiaptomus cf. dorsalis|ZMIII848-12|Mexico|BOLD:AAE0729
Arctodiaptomus cf. dorsalis|GBA14404-13|Mexico|BOLD:AAE0729
Arctodiaptomus cf. dorsalis|GBA14409-13|Mexico|BOLD:AAE0729
Arctodiaptomus cf. dorsalis|GBA14410-13|Mexico|BOLD:AAE0729
Arctodiaptomus cf. dorsalis|GBA14407-13|Mexico|BOLD:AAE0729
Arctodiaptomus cf. dorsalis|ZMIII847-12|Mexico|BOLD:AAE0729
Arctodiaptomus cf. dorsalis|ZMIII752-12|Mexico|BOLD:AAE0729
Arctodiaptomus cf. dorsalis|ZPLIV733-11|Mexico|BOLD:AAE0729
Arctodiaptomus cf. dorsalis|ZPLIV727-11|Mexico|BOLD:AAE0729
Arctodiaptomus cf. dorsalis|ZPII1431-11|Mexico|BOLD:AAE0729
Arctodiaptomus cf. dorsalis|ZPLIV726-11|Mexico|BOLD:AAE0729
Arctodiaptomus cf. dorsalis|ZPLIV670-11|Mexico|BOLD:AAE0729
Arctodiaptomus cf. dorsalis|ZPII1374-11|Mexico|BOLD:AAE0729
Arctodiaptomus cf. dorsalis 1|EXDB281-20|Mexico|BOLD:AAE0729
Arctodiaptomus cf. dorsalis 1|EXDB619-21|Mexico|BOLD:AAE0729
Arctodiaptomus cf. dorsalis 1|EXDB571-21|Mexico|BOLD:AAE0729
Arctodiaptomus cf. dorsalis 1|EXDB600-21|Mexico|BOLD:AAE0729
Arctodiaptomus cf. dorsalis 1|EXDB541-20|Mexico|BOLD:AAE0729
Arctodiaptomus cf. dorsalis 1|EXDB602-21|Mexico|BOLD:AAE0729
Arctodiaptomus cf. dorsalis 1|EXDB604-21|Mexico|BOLD:AAE0729
Arctodiaptomus cf. dorsalis 1|EXDB486-20|Mexico|BOLD:AAE0729
Arctodiaptomus cf. dorsalis 1|EXDB544-20|Mexico|BOLD:AAE0729
Arctodiaptomus cf. dorsalis 1|EXDB666-21|Mexico|BOLD:AAE0729
Arctodiaptomus cf. dorsalis 1|EXDB720-21|Mexico|BOLD:AAE0729
Arctodiaptomus cf. dorsalis1|ZPLIV711-11|Mexico|BOLD:AAE0729
Arctodiaptomus cf. dorsalis1|ZPLIV714-11|Mexico|BOLD:AAE0729
Arctodiaptomus cf. dorsalis1|ZPLIV712-11|Mexico|BOLD:AAE0729
Arctodiaptomus cf. dorsalis|ZPLIV713-11|Mexico|BOLD:AAE0729
Arctodiaptomus cf. dorsalis|ZPLIV741-11|Mexico|BOLD:AAE0729
Arctodiaptomus cf. dorsalis 1|EXDB743-21|Mexico|BOLD:AAE0729
Arctodiaptomus cf. dorsalis|CAZUL170-17|Mexico|BOLD:AEM6544
Arctodiaptomus cf. dorsalis|CAZUL169-17|Mexico|BOLD:AEM6544
Arctodiaptomus cf. dorsalis|MCM027-11|Mexico|BOLD:AEM6544
Arctodiaptomus cf. dorsalis|CAZUL091-17|Mexico|BOLD:AEM6544
Arctodiaptomus cf. dorsalis|CAZUL143-17|Mexico|BOLD:AEM6544
Arctodiaptomus cf. dorsalis|CAZUL121-17|Mexico|BOLD:AEM6544
Arctodiaptomus cf. dorsalis|CAZUL167-17|Mexico|BOLD:AEM6544
Arctodiaptomus cf. dorsalis|CAZUL141-17|Mexico|BOLD:AEM6544
Arctodiaptomus dorsalis|CAZUL171-17|Mexico|BOLD:AEM6544
Arctodiaptomus cf. dorsalis|MCM029-11|Mexico|BOLD:AEM6544
Arctodiaptomus cf. dorsalis|CAZUL172-17|Mexico|BOLD:AEM6544
Arctodiaptomus cf. dorsalis|CAZUL168-17|Mexico|BOLD:AEM6544
Arctodiaptomus cf. dorsalis|CAZUL090-17|Mexico|BOLD:AEM6544
Arctodiaptomus cf. dorsalis|CAZUL139-17|Mexico|BOLD:AEM6544
Arctodiaptomus cf. dorsalis|CAZUL140-17|Mexico|BOLD:AEM6544
Arctodiaptomus cf. dorsalis|CAZUL142-17|Mexico|BOLD:AEM6544
Arctodiaptomus cf. dorsalis|CAZUL173-17|Mexico|BOLD:AEM6544
Arctodiaptomus cf. dorsalis|CAZUL125-17|Mexico|BOLD:AEM6544
Arctodiaptomus cf. dorsalis|CAZUL174-17|Mexico|BOLD:AEM6544
Arctodiaptomus dorsalis|ZPLMX513-06|Mexico|BOLD:AEM6544
Arctodiaptomus dorsalis|ZPLMX512-06|Mexico|BOLD:AEM6544
Arctodiaptomus dorsalis|ZPLMX514-06|Mexico|BOLD:ACF0749
Arctodiaptomus cf. dorsalis|EXDB1864-21|Mexico|BOLD:AEM6544
Arctodiaptomus cf. dorsalis|EXDB1848-21|Mexico|BOLD:AEM6544
Arctodiaptomus cf. dorsalis|EXDB1849-21|Mexico|BOLD:AEM6544
Arctodiaptomus cf. dorsalis|EXDB1956-21|Mexico|BOLD:AEM6544
Arctodiaptomus cf. dorsalis|EXDB1969-21|Mexico|BOLD:AEM6544
Arctodiaptomus cf. dorsalis|BACZP2236-16|Mexico|BOLD:ADD6759
Arctodiaptomus cf. dorsalis|EXDB1666-21|Mexico|BOLD:AER1215
Arctodiaptomus cf. dorsalis|BACZP2240-16|Mexico|BOLD:ADD8857
Arctodiaptomus cf. dorsalis|BACZP2253-16|Mexico|BOLD:ADD7671
Arctodiaptomus cf. dorsalis|BROW079-23|Mexico|BOLD:ACE4750
Arctodiaptomus cf. dorsalis|BACZP2248-16|Mexico|BOLD:ACE4750
Arctodiaptomus cf. dorsalis|BACZP857-16|Mexico|BOLD:ACE4750
Arctodiaptomus cf. dorsalis|EXDB1893-21|Mexico|BOLD:ACE4750
Arctodiaptomus cf. dorsalis|BROWN002-22|Mexico|BOLD:ACE4750
Arctodiaptomus cf. dorsalis|BROWN017-22|Mexico|BOLD:ACE4750
Arctodiaptomus cf. dorsalis|GBA14420-13|Mexico|BOLD:ACE4750
Arctodiaptomus cf. dorsalis|GBA14416-13|Mexico|BOLD:ACE4750
Arctodiaptomus cf. dorsalis|ZMIII801-12|Mexico|BOLD:ACE4750
Arctodiaptomus cf. dorsalis|ZMIII706-12|Mexico|BOLD:ACE4750
Arctodiaptomus cf. dorsalis|BACZP056-15|Mexico|BOLD:ACE4750
Arctodiaptomus cf. dorsalis|EXDB1788-21|Mexico|BOLD:ACE4750
Arctodiaptomus cf. dorsalis|BROW257-24|Mexico|BOLD:ACE4750
Arctodiaptomus cf. dorsalis|BACZP006-15|Mexico|BOLD:ACE4750
Arctodiaptomus cf. dorsalis|BACZP2187-16|Mexico|BOLD:ACE4750
Arctodiaptomus cf. dorsalis|BACZP2354-16|Mexico|BOLD:ACE4750
Arctodiaptomus cf. dorsalis|BACZP2382-16|Mexico|BOLD:ACE4750
Arctodiaptomus cf. dorsalis|EXDB1808-21|Mexico|BOLD:ACE4750
Arctodiaptomus cf. dorsalis|EXDB1834-21|Mexico|BOLD:ACE4750

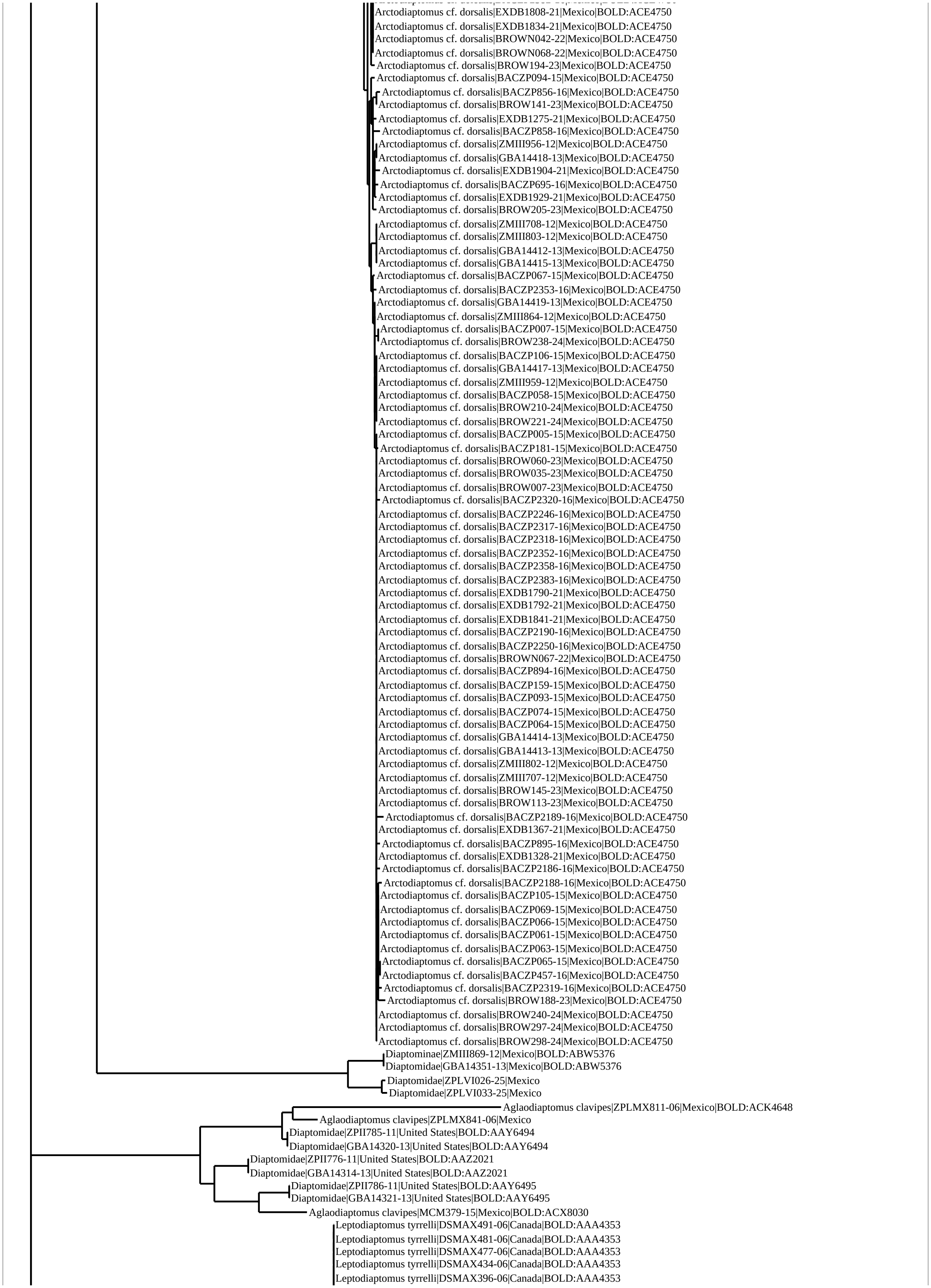
Arctodiaptomus cf. dorsalis|EXDB1808-21|Mexico|BOLD:ACE4750
Arctodiaptomus cf. dorsalis|EXDB1834-21|Mexico|BOLD:ACE4750
Arctodiaptomus cf. dorsalis|BROWN042-22|Mexico|BOLD:ACE4750
Arctodiaptomus cf. dorsalis|BROWN068-22|Mexico|BOLD:ACE4750
Arctodiaptomus cf. dorsalis|BROW194-23|Mexico|BOLD:ACE4750
Arctodiaptomus cf. dorsalis|BACZP094-15|Mexico|BOLD:ACE4750
Arctodiaptomus cf. dorsalis|BACZP856-16|Mexico|BOLD:ACE4750
Arctodiaptomus cf. dorsalis|BROW141-23|Mexico|BOLD:ACE4750
Arctodiaptomus cf. dorsalis|EXDB1275-21|Mexico|BOLD:ACE4750
Arctodiaptomus cf. dorsalis|BACZP858-16|Mexico|BOLD:ACE4750
Arctodiaptomus cf. dorsalis|ZMIII956-12|Mexico|BOLD:ACE4750
Arctodiaptomus cf. dorsalis|GBA14418-13|Mexico|BOLD:ACE4750
Arctodiaptomus cf. dorsalis|EXDB1904-21|Mexico|BOLD:ACE4750
Arctodiaptomus cf. dorsalis|BACZP695-16|Mexico|BOLD:ACE4750
Arctodiaptomus cf. dorsalis|EXDB1929-21|Mexico|BOLD:ACE4750
Arctodiaptomus cf. dorsalis|BROW205-23|Mexico|BOLD:ACE4750
Arctodiaptomus cf. dorsalis|ZMIII708-12|Mexico|BOLD:ACE4750
Arctodiaptomus cf. dorsalis|ZMIII803-12|Mexico|BOLD:ACE4750
Arctodiaptomus cf. dorsalis|GBA14412-13|Mexico|BOLD:ACE4750
Arctodiaptomus cf. dorsalis|GBA14415-13|Mexico|BOLD:ACE4750
Arctodiaptomus cf. dorsalis|BACZP067-15|Mexico|BOLD:ACE4750
Arctodiaptomus cf. dorsalis|BACZP2353-16|Mexico|BOLD:ACE4750
Arctodiaptomus cf. dorsalis|GBA14419-13|Mexico|BOLD:ACE4750
Arctodiaptomus cf. dorsalis|ZMIII864-12|Mexico|BOLD:ACE4750
Arctodiaptomus cf. dorsalis|BACZP007-15|Mexico|BOLD:ACE4750
Arctodiaptomus cf. dorsalis|BROW238-24|Mexico|BOLD:ACE4750
Arctodiaptomus cf. dorsalis|BACZP106-15|Mexico|BOLD:ACE4750
Arctodiaptomus cf. dorsalis|GBA14417-13|Mexico|BOLD:ACE4750
Arctodiaptomus cf. dorsalis|ZMIII959-12|Mexico|BOLD:ACE4750
Arctodiaptomus cf. dorsalis|BACZP058-15|Mexico|BOLD:ACE4750
Arctodiaptomus cf. dorsalis|BROW210-24|Mexico|BOLD:ACE4750
Arctodiaptomus cf. dorsalis|BROW221-24|Mexico|BOLD:ACE4750
Arctodiaptomus cf. dorsalis|BACZP005-15|Mexico|BOLD:ACE4750
Arctodiaptomus cf. dorsalis|BACZP181-15|Mexico|BOLD:ACE4750
Arctodiaptomus cf. dorsalis|BROW060-23|Mexico|BOLD:ACE4750
Arctodiaptomus cf. dorsalis|BROW035-23|Mexico|BOLD:ACE4750
Arctodiaptomus cf. dorsalis|BROW007-23|Mexico|BOLD:ACE4750
Arctodiaptomus cf. dorsalis|BACZP2320-16|Mexico|BOLD:ACE4750
Arctodiaptomus cf. dorsalis|BACZP2246-16|Mexico|BOLD:ACE4750
Arctodiaptomus cf. dorsalis|BACZP2317-16|Mexico|BOLD:ACE4750
Arctodiaptomus cf. dorsalis|BACZP2318-16|Mexico|BOLD:ACE4750
Arctodiaptomus cf. dorsalis|BACZP2352-16|Mexico|BOLD:ACE4750
Arctodiaptomus cf. dorsalis|BACZP2358-16|Mexico|BOLD:ACE4750
Arctodiaptomus cf. dorsalis|BACZP2383-16|Mexico|BOLD:ACE4750
Arctodiaptomus cf. dorsalis|EXDB1790-21|Mexico|BOLD:ACE4750
Arctodiaptomus cf. dorsalis|EXDB1792-21|Mexico|BOLD:ACE4750
Arctodiaptomus cf. dorsalis|EXDB1841-21|Mexico|BOLD:ACE4750
Arctodiaptomus cf. dorsalis|BACZP2190-16|Mexico|BOLD:ACE4750
Arctodiaptomus cf. dorsalis|BACZP2250-16|Mexico|BOLD:ACE4750
Arctodiaptomus cf. dorsalis|BROWN067-22|Mexico|BOLD:ACE4750
Arctodiaptomus cf. dorsalis|BACZP894-16|Mexico|BOLD:ACE4750
Arctodiaptomus cf. dorsalis|BACZP159-15|Mexico|BOLD:ACE4750
Arctodiaptomus cf. dorsalis|BACZP093-15|Mexico|BOLD:ACE4750
Arctodiaptomus cf. dorsalis|BACZP074-15|Mexico|BOLD:ACE4750
Arctodiaptomus cf. dorsalis|BACZP064-15|Mexico|BOLD:ACE4750
Arctodiaptomus cf. dorsalis|GBA14414-13|Mexico|BOLD:ACE4750
Arctodiaptomus cf. dorsalis|GBA14413-13|Mexico|BOLD:ACE4750
Arctodiaptomus cf. dorsalis|ZMIII802-12|Mexico|BOLD:ACE4750
Arctodiaptomus cf. dorsalis|ZMIII707-12|Mexico|BOLD:ACE4750
Arctodiaptomus cf. dorsalis|BROW145-23|Mexico|BOLD:ACE4750
Arctodiaptomus cf. dorsalis|BROW113-23|Mexico|BOLD:ACE4750
Arctodiaptomus cf. dorsalis|BACZP2189-16|Mexico|BOLD:ACE4750
Arctodiaptomus cf. dorsalis|EXDB1367-21|Mexico|BOLD:ACE4750
Arctodiaptomus cf. dorsalis|BACZP895-16|Mexico|BOLD:ACE4750
Arctodiaptomus cf. dorsalis|EXDB1328-21|Mexico|BOLD:ACE4750
Arctodiaptomus cf. dorsalis|BACZP2186-16|Mexico|BOLD:ACE4750
Arctodiaptomus cf. dorsalis|BACZP2188-16|Mexico|BOLD:ACE4750
Arctodiaptomus cf. dorsalis|BACZP105-15|Mexico|BOLD:ACE4750
Arctodiaptomus cf. dorsalis|BACZP069-15|Mexico|BOLD:ACE4750
Arctodiaptomus cf. dorsalis|BACZP066-15|Mexico|BOLD:ACE4750
Arctodiaptomus cf. dorsalis|BACZP061-15|Mexico|BOLD:ACE4750
Arctodiaptomus cf. dorsalis|BACZP063-15|Mexico|BOLD:ACE4750
Arctodiaptomus cf. dorsalis|BACZP065-15|Mexico|BOLD:ACE4750
Arctodiaptomus cf. dorsalis|BACZP457-16|Mexico|BOLD:ACE4750
Arctodiaptomus cf. dorsalis|BACZP2319-16|Mexico|BOLD:ACE4750
Arctodiaptomus cf. dorsalis|BROW188-23|Mexico|BOLD:ACE4750
Arctodiaptomus cf. dorsalis|BROW240-24|Mexico|BOLD:ACE4750
Arctodiaptomus cf. dorsalis|BROW297-24|Mexico|BOLD:ACE4750
Arctodiaptomus cf. dorsalis|BROW298-24|Mexico|BOLD:ACE4750
Diaptominae|ZMIII869-12|Mexico|BOLD:ABW5376
Diaptomidae|GBA14351-13|Mexico|BOLD:ABW5376
Diaptomidae|ZPLVI026-25|Mexico
Diaptomidae|ZPLVI033-25|Mexico
Aglaodiaptomus clavipes|ZPLMX811-06|Mexico|BOLD:ACK4648
Aglaodiaptomus clavipes|ZPLMX841-06|Mexico
Diaptomidae|ZPII785-11|United States|BOLD:AAY6494
Diaptomidae|GBA14320-13|United States|BOLD:AAY6494
Diaptomidae|ZPII776-11|United States|BOLD:AAZ2021
Diaptomidae|GBA14314-13|United States|BOLD:AAZ2021
Diaptomidae|ZPII786-11|United States|BOLD:AAY6495
Diaptomidae|GBA14321-13|United States|BOLD:AAY6495
Aglaodiaptomus clavipes|MCM379-15|Mexico|BOLD:ACX8030
Leptodiaptomus tyrrelli|DSMAX491-06|Canada|BOLD:AAA4353
Leptodiaptomus tyrrelli|DSMAX481-06|Canada|BOLD:AAA4353
Leptodiaptomus tyrrelli|DSMAX477-06|Canada|BOLD:AAA4353
Leptodiaptomus tyrrelli|DSMAX434-06|Canada|BOLD:AAA4353
Leptodiaptomus tyrrelli|DSMAX396-06|Canada|BOLD:AAA4353

Leptodiaptomus tyrrelli|DSMAX477-06|Canada|BOLD:AAA4353
Leptodiaptomus tyrrelli|DSMAX434-06|Canada|BOLD:AAA4353
Leptodiaptomus tyrrelli|DSMAX396-06|Canada|BOLD:AAA4353
Leptodiaptomus tyrrelli|DSMAX385-06|Canada|BOLD:AAA4353
Leptodiaptomus tyrrelli|DSMAX384-06|Canada|BOLD:AAA4353
Leptodiaptomus tyrrelli|DSMAX374-06|Canada|BOLD:AAA4353
Leptodiaptomus tyrrelli|DSMAX369-06|Canada|BOLD:AAA4353
Leptodiaptomus tyrrelli|AEDNA690-12|Canada|BOLD:AAA4353
Leptodiaptomus tyrrelli sp. 3 CHU|OZFWC961-12|Canada|BOLD:AAA4353
Leptodiaptomus tyrrelli sp. 3 CHU|OZFWC824-12|Canada|BOLD:AAA4353
Leptodiaptomus tyrrelli sp. 3 CHU|OZFWC821-12|Canada|BOLD:AAA4353
Leptodiaptomus tyrrelli sp. 3 CHU|OZFWC816-12|Canada|BOLD:AAA4353
Leptodiaptomus tyrrelli sp. 3 CHU|OZFWC811-12|Canada|BOLD:AAA4353
Leptodiaptomus tyrrelli sp. 3 CHU|OZFWC810-12|Canada|BOLD:AAA4353
Leptodiaptomus tyrrelli sp. 3 CHU|OZFWC804-12|Canada|BOLD:AAA4353
Leptodiaptomus tyrrelli sp. 3 CHU|OZFWC776-12|Canada|BOLD:AAA4353
Leptodiaptomus tyrrelli sp. 3 CHU|OZFWC765-12|Canada|BOLD:AAA4353
Leptodiaptomus tyrrelli sp. 3 CHU|OZFWC706-12|Canada|BOLD:AAA4353
Leptodiaptomus tyrrelli sp. 3 CHU|OZFWC704-12|Canada|BOLD:AAA4353
Leptodiaptomus tyrrelli sp. 3 CHU|OZFWC680-12|Canada|BOLD:AAA4353
Leptodiaptomus tyrrelli sp. 3 CHU|OZFWC634-12|Canada|BOLD:AAA4353
Leptodiaptomus tyrrelli sp. 3 CHU|OZFWC575-12|Canada|BOLD:AAA4353
Leptodiaptomus tyrrelli sp. 3 CHU|OZFWC530-12|Canada|BOLD:AAA4353
Leptodiaptomus tyrrelli sp. 3 CHU|OZFWC411-11|Canada|BOLD:AAA4353
Leptodiaptomus tyrrelli sp. 3 CHU|OZFWC394-11|Canada|BOLD:AAA4353
Leptodiaptomus tyrrelli sp. 3 CHU|OZFWC383-11|Canada|BOLD:AAA4353
Leptodiaptomus tyrrelli sp. 3 CHU|OZFWC362-11|Canada|BOLD:AAA4353
Leptodiaptomus tyrrelli sp. 3 CHU|OZFWC361-11|Canada|BOLD:AAA4353
Leptodiaptomus tyrrelli sp. 3 CHU|OZFWC325-11|Canada|BOLD:AAA4353
Leptodiaptomus tyrrelli sp. 3 CHU|OZFWC311-11|Canada|BOLD:AAA4353
Leptodiaptomus tyrrelli sp. 3 CHU|OZFWC231-11|Canada|BOLD:AAA4353
Leptodiaptomus tyrrelli sp. 3 CHU|OZFWC206-11|Canada|BOLD:AAA4353
Leptodiaptomus tyrrelli sp. 3 CHU|OZFWZ464-11|Canada|BOLD:AAA4353
Leptodiaptomus tyrrelli sp. 3 CHU|OZFWZ463-11|Canada|BOLD:AAA4353
Leptodiaptomus tyrrelli sp. 3 CHU|OZFWZ338-11|Canada|BOLD:AAA4353
Leptodiaptomus tyrrelli sp. 3 CHU|OZFWZ337-11|Canada|BOLD:AAA4353
Leptodiaptomus tyrrelli sp. 3 CHU|OZFWZ182-11|Canada|BOLD:AAA4353
Leptodiaptomus tyrrelli|SGRZC043-09|Canada|BOLD:AAA4353
Leptodiaptomus tyrrelli|SGRZC042-09|Canada|BOLD:AAA4353
Leptodiaptomus tyrrelli|CRCH031-09|Canada|BOLD:AAA4353
Leptodiaptomus tyrrelli|CRCH030-09|Canada|BOLD:AAA4353
Leptodiaptomus tyrrelli|CRCH028-09|Canada|BOLD:AAA4353
Leptodiaptomus tyrrelli|SACOP041-08|Canada|BOLD:AAA4353
Leptodiaptomus tyrrelli|SACOP017-08|Canada|BOLD:AAA4353
Leptodiaptomus tyrrelli|DSMAX535-06|Canada|BOLD:AAA4353
Leptodiaptomus tyrrelli|DSMAX523-06|Canada|BOLD:AAA4353
Leptodiaptomus tyrrelli|DSMAX513-06|Canada|BOLD:AAA4353
Leptodiaptomus tyrrelli sp. 3 CHU|OZFWC964-12|Canada|BOLD:AAA4353
Leptodiaptomus tyrrelli sp. 3 CHU|OZFWC962-12|Canada|BOLD:AAA4353
Leptodiaptomus tyrrelli|DSMAX348-06|Canada|BOLD:AAA4353
Leptodiaptomus tyrrelli sp. 3 CHU|OZFWC191-11|Canada|BOLD:AAA4353
Leptodiaptomus tyrrelli sp. 3 CHU|OZFWC276-11|Canada|BOLD:AAA4353
Leptodiaptomus tyrrelli sp. 3 CHU|OZFWC840-12|Canada|BOLD:AAA4353
Leptodiaptomus tyrrelli sp. 3 CHU|OZFWC718-12|Canada|BOLD:AAA4353
Leptodiaptomus tyrrelli sp. 3 CHU|OZFWC661-12|Canada|BOLD:AAA4353
Leptodiaptomus tyrrelli sp. 3 CHU|OZFWC303-11|Canada|BOLD:AAA4353
Leptodiaptomus tyrrelli|DSMAX442-06|Canada|BOLD:AAA4353
Leptodiaptomus tyrrelli|DSMAX420-06|Canada|BOLD:AAA4353
Leptodiaptomus tyrrelli|SGRZC033-09|Canada|BOLD:AAA4353
Leptodiaptomus tyrrelli|SGRZC032-09|Canada|BOLD:AAA4353
Leptodiaptomus tyrrelli|DSMAX416-06|Canada|BOLD:AAA4353
Leptodiaptomus tyrrelli|SGRZC023-09|Canada|BOLD:AAA4353
Leptodiaptomus tyrrelli|DSMAX489-06|Canada|BOLD:AAA4353
Leptodiaptomus tyrrelli|DSMAX407-06|Canada|BOLD:AAA4353
Leptodiaptomus tyrrelli|SGRZC022-09|Canada|BOLD:AAA4353
Leptodiaptomus tyrrelli|SGRZC034-09|Canada|BOLD:AAA4353
Leptodiaptomus tyrrelli sp. 3 CHU|OZFWZ364-11|Canada|BOLD:AAA4353
Leptodiaptomus tyrrelli sp. 3 CHU|OZFWC290-11|Canada|BOLD:AAA4353
Leptodiaptomus tyrrelli sp. 3 CHU|OZFWC302-11|Canada|BOLD:AAA4353
Leptodiaptomus tyrrelli sp. 3 CHU|OZFWC301-11|Canada|BOLD:AAA4353
Leptodiaptomus tyrrelli sp. 3 CHU|OZFWC275-11|Canada|BOLD:AAA4353
Leptodiaptomus tyrrelli sp. 3 CHU|OZFWZ353-11|Canada|BOLD:AAA4353
Leptodiaptomus tyrrelli|DSMAX529-06|Canada|BOLD:AAA4353
Leptodiaptomus tyrrelli|ELPPC035-09|Canada|BOLD:AAA4353
Leptodiaptomus tyrrelli sp. 3 CHU|OZFWZ330-11|Canada|BOLD:AAA4353
Leptodiaptomus tyrrelli sp. 3 CHU|OZFWC312-11|Canada|BOLD:AAA4353
Leptodiaptomus tyrrelli|DSMAX543-06|Canada|BOLD:AAA4353
Leptodiaptomus tyrrelli sp. 3 CHU|OZFWC221-11|Canada|BOLD:AAA4353
Leptodiaptomus tyrrelli|SGRZC021-09|Canada|BOLD:AAA4353
Leptodiaptomus tyrrelli sp. 3 CHU|OZFWC715-12|Canada|BOLD:AAA4353
Leptodiaptomus tyrrelli sp. 3 CHU|OZFWZ367-11|Canada|BOLD:AAA4353
Leptodiaptomus tyrrelli|DSMAX512-06|Canada|BOLD:AAA4353
Leptodiaptomus tyrrelli|KLMPC003-09|Canada|BOLD:AAA4353
Leptodiaptomus tyrrelli sp. 3 CHU|OZFWZ358-11|Canada|BOLD:AAA4353
Leptodiaptomus tyrrelli sp. 3 CHU|OZFWZ336-11|Canada|BOLD:AAA4353
Leptodiaptomus tyrrelli sp. 3 CHU|OZFWZ340-11|Canada|BOLD:AAA4353
Leptodiaptomus tyrrelli sp. 3 CHU|OZFWZ355-11|Canada|BOLD:AAA4353
Leptodiaptomus tyrrelli|CRCN046-09|Canada|BOLD:AAA4353
Leptodiaptomus tyrrelli|CRCN047-09|Canada|BOLD:AAA4353
Leptodiaptomus tyrrelli sp. 3 CHU|OZFWZ341-11|Canada|BOLD:AAA4353
Leptodiaptomus tyrrelli sp. 3 CHU|OZFWZ346-11|Canada|BOLD:AAA4353
Leptodiaptomus tyrrelli sp. 3 CHU|OZFWZ365-11|Canada|BOLD:AAA4353
Leptodiaptomus tyrrelli sp. 3 CHU|OZFWZ356-11|Canada|BOLD:AAA4353
Leptodiaptomus tyrrelli|DSMAX500-06|Canada|BOLD:AAA4353
Leptodiaptomus tyrrelli sp. 3 CHU|OZFWC297-11|Canada|BOLD:AAA4353
Leptodiaptomus tyrrelli sp. 3 CHU|OZFWC299-11|Canada|BOLD:AAA4353
Leptodiaptomus tyrrelli|DSMAX507-06|Canada|BOLD:AAA4353
Leptodiaptomus tyrrelli|DSMAX501-06|Canada|BOLD:AAA4353

Leptodiaptomus tyrrelli sp. 3 CHU|OZFWC299-11|Canada|BOLD:AAA4353
Leptodiaptomus tyrrelli|DSMAX507-06|Canada|BOLD:AAA4353
Leptodiaptomus tyrrelli|DSMAX501-06|Canada|BOLD:AAA4353
Leptodiaptomus tyrrelli|DSMAX497-06|Canada|BOLD:AAA4353
Leptodiaptomus tyrrelli sp. 3 CHU|OZFWZ335-11|Canada|BOLD:AAA4353
Leptodiaptomus tyrrelli|SGRZC012-09|Canada|BOLD:AAA4353
Leptodiaptomus tyrrelli sp. 3 CHU|OZFWZ343-11|Canada|BOLD:AAA4353
Leptodiaptomus tyrrelli sp. 3 CHU|OZFWZ451-11|Canada|BOLD:AAA4353
Leptodiaptomus tyrrelli sp. 3 CHU|OZFWC140-11|Canada|BOLD:AAA4353
Leptodiaptomus tyrrelli sp. 3 CHU|OZFWC797-12|Canada|BOLD:AAA4353
Leptodiaptomus tyrrelli sp. 3 CHU|OZFWC796-12|Canada|BOLD:AAA4353
Leptodiaptomus tyrrelli sp. 3 CHU|OZFWC789-12|Canada|BOLD:AAA4353
Leptodiaptomus tyrrelli sp. 3 CHU|OZFWC787-12|Canada|BOLD:AAA4353
Leptodiaptomus tyrrelli sp. 3 CHU|OZFWC784-12|Canada|BOLD:AAA4353
Leptodiaptomus tyrrelli sp. 3 CHU|OZFWC782-12|Canada|BOLD:AAA4353
Leptodiaptomus tyrrelli sp. 3 CHU|OZFWC749-12|Canada|BOLD:AAA4353
Leptodiaptomus tyrrelli sp. 3 CHU|OZFWC660-12|Canada|BOLD:AAA4353
Leptodiaptomus tyrrelli sp. 3 CHU|OZFWC650-12|Canada|BOLD:AAA4353
Leptodiaptomus tyrrelli sp. 3 CHU|OZFWC649-12|Canada|BOLD:AAA4353
Leptodiaptomus tyrrelli sp. 3 CHU|OZFWC565-12|Canada|BOLD:AAA4353
Leptodiaptomus tyrrelli sp. 3 CHU|OZFWC374-11|Canada|BOLD:AAA4353
Leptodiaptomus tyrrelli sp. 3 CHU|OZFWC355-11|Canada|BOLD:AAA4353
Leptodiaptomus tyrrelli sp. 3 CHU|OZFWZ475-11|Canada|BOLD:AAA4353
Leptodiaptomus tyrrelli sp. 3 CHU|OZFWZ474-11|Canada|BOLD:AAA4353
Leptodiaptomus tyrrelli sp. 3 CHU|OZFWZ348-11|Canada|BOLD:AAA4353
Leptodiaptomus tyrrelli sp. 3 CHU|OZFWC752-12|Canada|BOLD:AAA4353
Leptodiaptomus tyrrelli sp. 3 CHU|OZFWC754-12|Canada|BOLD:AAA4353
Leptodiaptomus tyrrelli sp. 3 CHU|OZFWC748-12|Canada|BOLD:AAA4353
Leptodiaptomus tyrrelli sp. 3 CHU|OZFWZ362-11|Canada|BOLD:AAA4353
Leptodiaptomus tyrrelli sp. 3 CHU|OZFWZ361-11|Canada|BOLD:AAA4353
Leptodiaptomus tyrrelli sp. 3 CHU|OZFWZ360-11|Canada|BOLD:AAA4353
Leptodiaptomus tyrrelli sp. 3 CHU|OZFWZ344-11|Canada|BOLD:AAA4353
Leptodiaptomus tyrrelli sp. 3 CHU|OZFWZ329-11|Canada|BOLD:AAA4353
Leptodiaptomus tyrrelli|DSMAX326-06|Canada|BOLD:AAA4353
Leptodiaptomus tyrrelli|DSMAX398-06|Canada|BOLD:AAA4353
Leptodiaptomus tyrrelli|DSMAX386-06|Canada|BOLD:AAA4353
Leptodiaptomus tyrrelli|DSMAX383-06|Canada|BOLD:AAA4353
Leptodiaptomus tyrrelli|DSMAX373-06|Canada|BOLD:AAA4353
Leptodiaptomus tyrrelli|DSMAX350-06|Canada|BOLD:AAA4353
Leptodiaptomus tyrrelli|DSMAX314-06|Canada|BOLD:AAA4353
Leptodiaptomus tyrrelli sp. 3 CHU|OZFWZ342-11|Canada|BOLD:AAA4353
Leptodiaptomus tyrrelli sp. 3 CHU|OZFWC493-11|Canada|BOLD:AAA4353
Leptodiaptomus tyrrelli|DSMAX446-06|Canada|BOLD:AAA4353
Leptodiaptomus tyrrelli|ZPII374-07|Canada|BOLD:AAA4353
Leptodiaptomus tyrrelli sp. 3 CHU|OZFWZ471-11|Canada|BOLD:AAA4353
Leptodiaptomus tyrrelli|DSMAX453-06|Canada|BOLD:AAA4353
Leptodiaptomus tyrrelli sp. 3 CHU|OZFWC220-11|Canada|BOLD:AAA4353
Leptodiaptomus tyrrelli|DSMAX441-06|Canada|BOLD:AAA4353
Leptodiaptomus tyrrelli sp. 3 CHU|OZFWC260-11|Canada|BOLD:AAA4353
Leptodiaptomus tyrrelli sp. 3 CHU|OZFWC261-11|Canada|BOLD:AAA4353
Leptodiaptomus tyrrelli sp. 3 CHU|OZFWC356-11|Canada|BOLD:AAA4353
Leptodiaptomus tyrrelli sp. 3 CHU|OZFWC352-11|Canada|BOLD:AAA4353
Leptodiaptomus tyrrelli sp. 3 CHU|OZFWC370-11|Canada|BOLD:AAA4353
Leptodiaptomus tyrelli|NJCGS081-09|Canada|BOLD:AAA4353
Leptodiaptomus tyrrelli|DSMAX360-06|Canada|BOLD:AAA4353
Leptodiaptomus tyrrelli|DSMAX375-06|Canada|BOLD:AAA4353
Leptodiaptomus tyrelli|NJCGS080-09|Canada|BOLD:AAA4353
Leptodiaptomus tyrelli|NJCGS084-09|Canada|BOLD:AAA4353
Leptodiaptomus tyrrelli sp. 3 CHU|OZFWC310-11|Canada|BOLD:AAA4353
Leptodiaptomus tyrrelli|DSMAX311-06|Canada|BOLD:AAA4353
Leptodiaptomus tyrrelli|DSMAX359-06|Canada|BOLD:AAA4353
Leptodiaptomus tyrrelli sp. 3 CHU|OZFWC212-11|Canada|BOLD:AAA4353
Leptodiaptomus tyrrelli sp. 3 CHU|OZFWC396-11|Canada|BOLD:AAA4353
Leptodiaptomus tyrrelli|AEDNA660-12|Canada|BOLD:AAA4353
Leptodiaptomus tyrrelli|DSMAX362-06|Canada|BOLD:AAA4353
Leptodiaptomus tyrrelli|DSMAX349-06|Canada|BOLD:AAA4353
Leptodiaptomus tyrrelli|DSMAX338-06|Canada|BOLD:AAA4353
Leptodiaptomus tyrrelli|AEDNA698-12|Canada|BOLD:AAA4353
Leptodiaptomus tyrrelli sp. 3 CHU|OZFWC793-12|Canada|BOLD:AAA4353
Leptodiaptomus tyrrelli sp. 3 CHU|OZFWC433-11|Canada|BOLD:AAA4353
Leptodiaptomus tyrrelli|DSMAX327-06|Canada|BOLD:AAA4353
Leptodiaptomus tyrrelli|DSMAX323-06|Canada|BOLD:AAA4353
Leptodiaptomus tyrrelli|DSMAX313-06|Canada|BOLD:AAA4353
Leptodiaptomus tyrrelli|DSMAX333-06|Canada|BOLD:AAA4353
Leptodiaptomus tyrrelli|DSMAX351-06|Canada|BOLD:AAA4353
Leptodiaptomus tyrrelli sp. 3 CHU|OZFWZ354-11|Canada|BOLD:AAA4353
Leptodiaptomus tyrrelli sp. 3 CHU|OZFWC388-11|Canada|BOLD:AAA4353
Leptodiaptomus tyrrelli sp. 3 CHU|OZFWZ357-11|Canada|BOLD:AAA4353
Leptodiaptomus tyrrelli sp. 3 CHU|OZFWC296-11|Canada|BOLD:AAA4353
Leptodiaptomus tyrrelli|DSMAX412-06|Canada|BOLD:AAA4353
Leptodiaptomus tyrrelli|SACOP020-08|Canada|BOLD:AAA4353
Leptodiaptomus tyrrelli sp. 3 CHU|OZFWC502-12|Canada|BOLD:AAA4353
Leptodiaptomus tyrrelli|SACOP059-08|Canada|BOLD:AAA4353
Leptodiaptomus tyrrelli sp. 3 CHU|OZFWZ368-11|Canada|BOLD:AAA4353
Leptodiaptomus tyrrelli|DSMAX368-06|Canada|BOLD:AAA4353
Leptodiaptomus tyrrelli sp. 3 CHU|OZFWZ288-11|Canada|BOLD:AAA4353
Leptodiaptomus tyrrelli|DSMAX321-06|Canada|BOLD:AAA4353
Leptodiaptomus tyrrelli sp. 3 CHU|OZFWZ345-11|Canada|BOLD:AAA4353
Leptodiaptomus tyrrelli sp. 3 CHU|OZFWC363-11|Canada|BOLD:AAA4353
Leptodiaptomus tyrrelli|SACOP018-08|Canada|BOLD:AAA4353
Leptodiaptomus tyrrelli|DSMAX345-06|Canada|BOLD:AAA4353
Leptodiaptomus tyrrelli|DSMAX357-06|Canada|BOLD:AAA4353
Leptodiaptomus tyrrelli|SACOP042-08|Canada|BOLD:AAA4353
Leptodiaptomus tyrrelli sp. 3 CHU|OZFWZ349-11|Canada|BOLD:AAA4353
Leptodiaptomus tyrrelli|DSMAX361-06|Canada|BOLD:AAA4353
Leptodiaptomus tyrrelli sp. 3 CHU|OZFWC689-12|Canada|BOLD:AAA4353
Leptodiaptomus tyrrelli sp. 3 CHU|OZFWZ339-11|Canada|BOLD:AAA4353

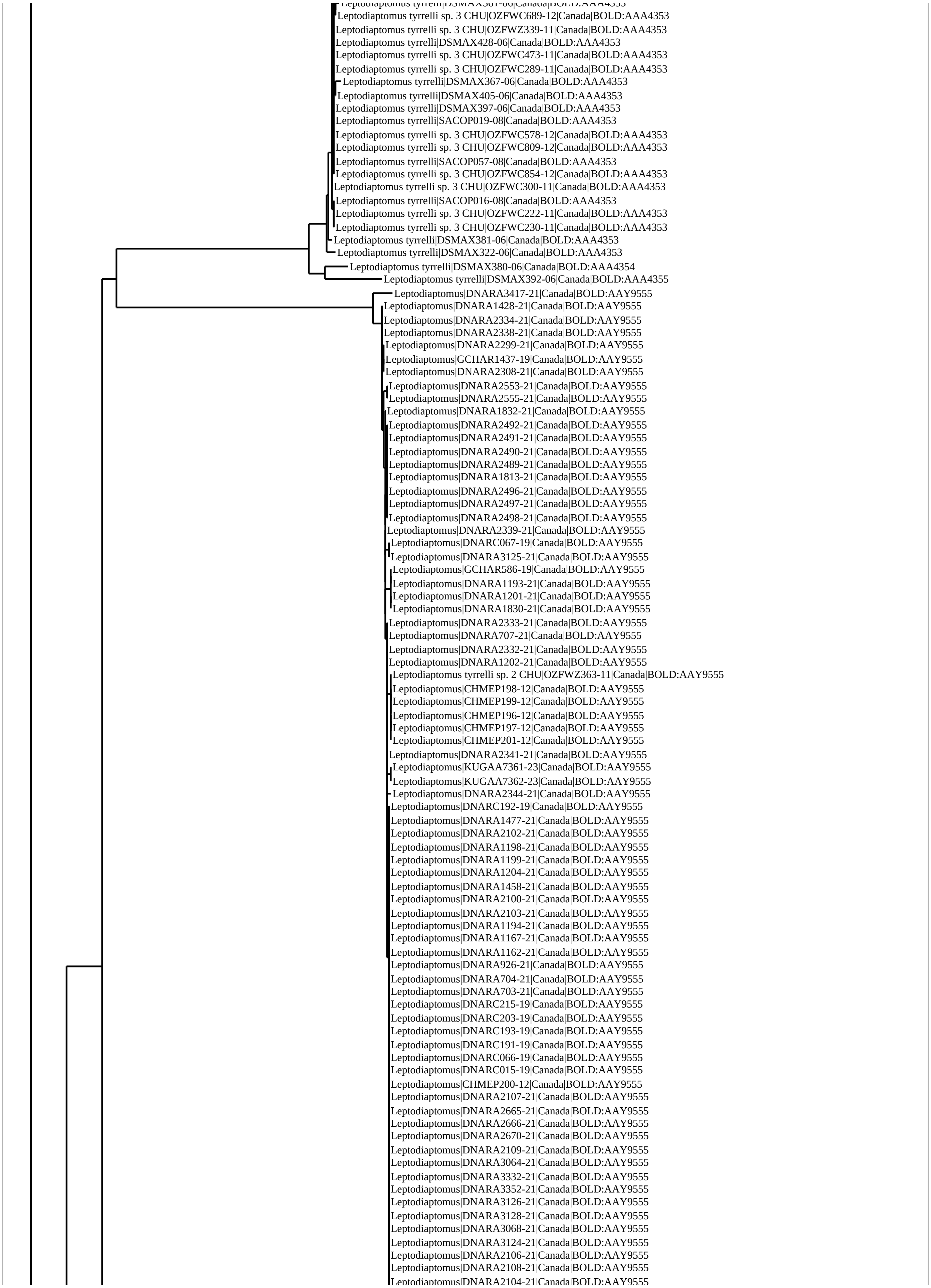
Leptodiaptomus tyrrelli|DSMAX361-06|Canada|BOLD:AAA4353
Leptodiaptomus tyrrelli sp. 3 CHU|OZFWC689-12|Canada|BOLD:AAA4353
Leptodiaptomus tyrrelli sp. 3 CHU|OZFWZ339-11|Canada|BOLD:AAA4353
Leptodiaptomus tyrrelli|DSMAX428-06|Canada|BOLD:AAA4353
Leptodiaptomus tyrrelli sp. 3 CHU|OZFWC473-11|Canada|BOLD:AAA4353
Leptodiaptomus tyrrelli sp. 3 CHU|OZFWC289-11|Canada|BOLD:AAA4353
Leptodiaptomus tyrrelli|DSMAX367-06|Canada|BOLD:AAA4353
Leptodiaptomus tyrrelli|DSMAX405-06|Canada|BOLD:AAA4353
Leptodiaptomus tyrrelli|DSMAX397-06|Canada|BOLD:AAA4353
Leptodiaptomus tyrrelli|SACOP019-08|Canada|BOLD:AAA4353
Leptodiaptomus tyrrelli sp. 3 CHU|OZFWC578-12|Canada|BOLD:AAA4353
Leptodiaptomus tyrrelli sp. 3 CHU|OZFWC809-12|Canada|BOLD:AAA4353
Leptodiaptomus tyrrelli|SACOP057-08|Canada|BOLD:AAA4353
Leptodiaptomus tyrrelli sp. 3 CHU|OZFWC854-12|Canada|BOLD:AAA4353
Leptodiaptomus tyrrelli sp. 3 CHU|OZFWC300-11|Canada|BOLD:AAA4353
Leptodiaptomus tyrrelli|SACOP016-08|Canada|BOLD:AAA4353
Leptodiaptomus tyrrelli sp. 3 CHU|OZFWC222-11|Canada|BOLD:AAA4353
Leptodiaptomus tyrrelli sp. 3 CHU|OZFWC230-11|Canada|BOLD:AAA4353
Leptodiaptomus tyrrelli|DSMAX381-06|Canada|BOLD:AAA4353
Leptodiaptomus tyrrelli|DSMAX322-06|Canada|BOLD:AAA4353
Leptodiaptomus tyrrelli|DSMAX380-06|Canada|BOLD:AAA4354
Leptodiaptomus tyrrelli|DSMAX392-06|Canada|BOLD:AAA4355
Leptodiaptomus|DNARA3417-21|Canada|BOLD:AAY9555
Leptodiaptomus|DNARA1428-21|Canada|BOLD:AAY9555
Leptodiaptomus|DNARA2334-21|Canada|BOLD:AAY9555
Leptodiaptomus|DNARA2338-21|Canada|BOLD:AAY9555
Leptodiaptomus|DNARA2299-21|Canada|BOLD:AAY9555
Leptodiaptomus|GCHAR1437-19|Canada|BOLD:AAY9555
Leptodiaptomus|DNARA2308-21|Canada|BOLD:AAY9555
Leptodiaptomus|DNARA2553-21|Canada|BOLD:AAY9555
Leptodiaptomus|DNARA2555-21|Canada|BOLD:AAY9555
Leptodiaptomus|DNARA1832-21|Canada|BOLD:AAY9555
Leptodiaptomus|DNARA2492-21|Canada|BOLD:AAY9555
Leptodiaptomus|DNARA2491-21|Canada|BOLD:AAY9555
Leptodiaptomus|DNARA2490-21|Canada|BOLD:AAY9555
Leptodiaptomus|DNARA2489-21|Canada|BOLD:AAY9555
Leptodiaptomus|DNARA1813-21|Canada|BOLD:AAY9555
Leptodiaptomus|DNARA2496-21|Canada|BOLD:AAY9555
Leptodiaptomus|DNARA2497-21|Canada|BOLD:AAY9555
Leptodiaptomus|DNARA2498-21|Canada|BOLD:AAY9555
Leptodiaptomus|DNARA2339-21|Canada|BOLD:AAY9555
Leptodiaptomus|DNARC067-19|Canada|BOLD:AAY9555
Leptodiaptomus|DNARA3125-21|Canada|BOLD:AAY9555
Leptodiaptomus|GCHAR586-19|Canada|BOLD:AAY9555
Leptodiaptomus|DNARA1193-21|Canada|BOLD:AAY9555
Leptodiaptomus|DNARA1201-21|Canada|BOLD:AAY9555
Leptodiaptomus|DNARA1830-21|Canada|BOLD:AAY9555
Leptodiaptomus|DNARA2333-21|Canada|BOLD:AAY9555
Leptodiaptomus|DNARA707-21|Canada|BOLD:AAY9555
Leptodiaptomus|DNARA2332-21|Canada|BOLD:AAY9555
Leptodiaptomus|DNARA1202-21|Canada|BOLD:AAY9555
Leptodiaptomus tyrrelli sp. 2 CHU|OZFWZ363-11|Canada|BOLD:AAY9555
Leptodiaptomus|CHMEP198-12|Canada|BOLD:AAY9555
Leptodiaptomus|CHMEP199-12|Canada|BOLD:AAY9555
Leptodiaptomus|CHMEP196-12|Canada|BOLD:AAY9555
Leptodiaptomus|CHMEP197-12|Canada|BOLD:AAY9555
Leptodiaptomus|CHMEP201-12|Canada|BOLD:AAY9555
Leptodiaptomus|DNARA2341-21|Canada|BOLD:AAY9555
Leptodiaptomus|KUGAA7361-23|Canada|BOLD:AAY9555
Leptodiaptomus|KUGAA7362-23|Canada|BOLD:AAY9555
Leptodiaptomus|DNARA2344-21|Canada|BOLD:AAY9555
Leptodiaptomus|DNARC192-19|Canada|BOLD:AAY9555
Leptodiaptomus|DNARA1477-21|Canada|BOLD:AAY9555
Leptodiaptomus|DNARA2102-21|Canada|BOLD:AAY9555
Leptodiaptomus|DNARA1198-21|Canada|BOLD:AAY9555
Leptodiaptomus|DNARA1199-21|Canada|BOLD:AAY9555
Leptodiaptomus|DNARA1204-21|Canada|BOLD:AAY9555
Leptodiaptomus|DNARA1458-21|Canada|BOLD:AAY9555
Leptodiaptomus|DNARA2100-21|Canada|BOLD:AAY9555
Leptodiaptomus|DNARA2103-21|Canada|BOLD:AAY9555
Leptodiaptomus|DNARA1194-21|Canada|BOLD:AAY9555
Leptodiaptomus|DNARA1167-21|Canada|BOLD:AAY9555
Leptodiaptomus|DNARA1162-21|Canada|BOLD:AAY9555
Leptodiaptomus|DNARA926-21|Canada|BOLD:AAY9555
Leptodiaptomus|DNARA704-21|Canada|BOLD:AAY9555
Leptodiaptomus|DNARA703-21|Canada|BOLD:AAY9555
Leptodiaptomus|DNARC215-19|Canada|BOLD:AAY9555
Leptodiaptomus|DNARC203-19|Canada|BOLD:AAY9555
Leptodiaptomus|DNARC193-19|Canada|BOLD:AAY9555
Leptodiaptomus|DNARC191-19|Canada|BOLD:AAY9555
Leptodiaptomus|DNARC066-19|Canada|BOLD:AAY9555
Leptodiaptomus|DNARC015-19|Canada|BOLD:AAY9555
Leptodiaptomus|CHMEP200-12|Canada|BOLD:AAY9555
Leptodiaptomus|DNARA2107-21|Canada|BOLD:AAY9555
Leptodiaptomus|DNARA2665-21|Canada|BOLD:AAY9555
Leptodiaptomus|DNARA2666-21|Canada|BOLD:AAY9555
Leptodiaptomus|DNARA2670-21|Canada|BOLD:AAY9555
Leptodiaptomus|DNARA2109-21|Canada|BOLD:AAY9555
Leptodiaptomus|DNARA3064-21|Canada|BOLD:AAY9555
Leptodiaptomus|DNARA3332-21|Canada|BOLD:AAY9555
Leptodiaptomus|DNARA3352-21|Canada|BOLD:AAY9555
Leptodiaptomus|DNARA3126-21|Canada|BOLD:AAY9555
Leptodiaptomus|DNARA3128-21|Canada|BOLD:AAY9555
Leptodiaptomus|DNARA3068-21|Canada|BOLD:AAY9555
Leptodiaptomus|DNARA3124-21|Canada|BOLD:AAY9555
Leptodiaptomus|DNARA2106-21|Canada|BOLD:AAY9555
Leptodiaptomus|DNARA2108-21|Canada|BOLD:AAY9555
Leptodiaptomus|DNARA2104-21|Canada|BOLD:AAY9555

Leptodiaptomus|DNARA2106-21|Canada|BOLD:AAY9555
Leptodiaptomus|DNARA2108-21|Canada|BOLD:AAY9555
Leptodiaptomus|DNARA2104-21|Canada|BOLD:AAY9555
Leptodiaptomus|DNARA2105-21|Canada|BOLD:AAY9555
Leptodiaptomus|DNARA1891-21|Canada|BOLD:AAY9555
Leptodiaptomus|DNARA2099-21|Canada|BOLD:AAY9555
Leptodiaptomus|DNARA1829-21|Canada|BOLD:AAY9555
Leptodiaptomus|DNARA1831-21|Canada|BOLD:AAY9555
Leptodiaptomus|DNARA1679-21|Canada|BOLD:AAY9555
Leptodiaptomus|DNARA1826-21|Canada|BOLD:AAY9555
Leptodiaptomus|DNARA1827-21|Canada|BOLD:AAY9555
Leptodiaptomus|DNARA1828-21|Canada|BOLD:AAY9555
Leptodiaptomus|DNARA1677-21|Canada|BOLD:AAY9555
Leptodiaptomus|DNARA1678-21|Canada|BOLD:AAY9555
Leptodiaptomus|DNARA1674-21|Canada|BOLD:AAY9555
Leptodiaptomus|DNARA1676-21|Canada|BOLD:AAY9555
Leptodiaptomus|DNARA1471-21|Canada|BOLD:AAY9555
Leptodiaptomus|DNARA1479-21|Canada|BOLD:AAY9555
Leptodiaptomus|DNARA3411-21|Canada|BOLD:AAY9555
Leptodiaptomus|KUGAA7440-23|Canada|BOLD:AAY9555
Leptodiaptomus|KUGAA7441-23|Canada|BOLD:AAY9555
Leptodiaptomus|KUGAA7442-23|Canada|BOLD:AAY9555
Leptodiaptomus|KUGAA7443-23|Canada|BOLD:AAY9555
Leptodiaptomus|KUGAA7444-23|Canada|BOLD:AAY9555
Skistodiaptomus oregonensis|SACOP047-08|Canada|BOLD:AAF4664
Skistodiaptomus oregonensis|BBCRU019-10|Canada|BOLD:AAF4664
Skistodiaptomus oregonensis|SACOP044-08|Canada|BOLD:AAF4664
Skistodiaptomus oregonensis|BBCRU020-10|Canada|BOLD:AAF4664
Skistodiaptomus oregonensis|BBCRU022-10|Canada|BOLD:AAF4664
Skistodiaptomus oregonensis|BBCRU024-10|Canada|BOLD:AAF4664
Skistodiaptomus oregonensis|CAISN849-13|Canada|BOLD:AAF4664
Skistodiaptomus oregonensis|ZOOPS681-20|Canada|BOLD:AAG9231
Skistodiaptomus oregonensis|GLC223-06|Canada
Skistodiaptomus oregonensis|ZPLMX912-06|Canada|BOLD:AAG9231
Skistodiaptomus oregonensis|COAPP498-13|Canada|BOLD:AAG9231
Skistodiaptomus oregonensis|GLC015-06|Canada
Skistodiaptomus oregonensis|CAISN033-12|Canada|BOLD:AAG9231
Skistodiaptomus oregonensis|CAISN923-13|Canada|BOLD:AAG9231
Skistodiaptomus oregonensis|ZPLMX994-06|Canada|BOLD:AAG9231
Skistodiaptomus oregonensis|COAPP484-13|Canada|BOLD:AAG9231
Skistodiaptomus oregonensis|BACZP1558-16|Canada|BOLD:AAG9231
Skistodiaptomus oregonensis|BACZP1562-16|Canada|BOLD:AAG9231
Skistodiaptomus oregonensis|ZPLMX911-06|Canada|BOLD:AAG9231
Skistodiaptomus oregonensis|ZPLCA005-06|Canada
Skistodiaptomus oregonensis|BACZP1066-16|Canada|BOLD:AAG9231
Skistodiaptomus oregonensis|ZOOPS682-20|Canada|BOLD:AAG9231
Skistodiaptomus oregonensis|CTM057-10|United States|BOLD:AAG9231
Skistodiaptomus oregonensis|SWCHL2231-16|Canada|BOLD:AAG9231
Skistodiaptomus oregonensis|SWCHL2237-16|Canada|BOLD:AAG9231
Skistodiaptomus oregonensis|BACZP1646-16|Canada|BOLD:AAG9231
Skistodiaptomus oregonensis|ZOOPS684-20|Canada|BOLD:AAG9231
Skistodiaptomus oregonensis|CAISN922-13|Canada|BOLD:AAG9231
Skistodiaptomus oregonensis|SWCHL2101-16|Canada|BOLD:AAG9231
Skistodiaptomus oregonensis|SWCHL2110-16|Canada|BOLD:AAG9231
Skistodiaptomus oregonensis|COAPP431-13|Canada|BOLD:AAG9231
Skistodiaptomus oregonensis|ZOOPS683-20|Canada|BOLD:AAG9231
Skistodiaptomus oregonensis|COAPP337-13|Canada|BOLD:AAG9231
Skistodiaptomus oregonensis|COAPP514-13|Canada|BOLD:AAG9231
Skistodiaptomus oregonensis|BACZP1727-16|Canada|BOLD:AAG9231
Skistodiaptomus oregonensis|BACZP1643-16|Canada|BOLD:AAG9231
Skistodiaptomus oregonensis|BACZP1647-16|Canada|BOLD:AAG9231
Skistodiaptomus oregonensis|BACZP1723-16|Canada|BOLD:AAG9231
Skistodiaptomus oregonensis|COAPP435-13|Canada|BOLD:AAG9231
Skistodiaptomus oregonensis|COAPP430-13|Canada|BOLD:AAG9231
Skistodiaptomus oregonensis|ZPLMX908-06|Canada|BOLD:AAG9231
Skistodiaptomus oregonensis|ZPLMX909-06|Canada|BOLD:AAG9231
Skistodiaptomus oregonensis|ZPLMX910-06|Canada
Skistodiaptomus oregonensis|COAPP314-13|Canada|BOLD:AAG9231
Skistodiaptomus oregonensis|SWCHL2098-16|Canada|BOLD:AAG9231
Skistodiaptomus oregonensis|ZOOPS685-20|Canada|BOLD:AAG9231
Diaptominae|ZPLIV578-11|Mexico|BOLD:ABA0904
Diaptominae|ZPLIV483-11|Mexico|BOLD:ABA0904
Diaptominae|ZPLIV579-11|Mexico|BOLD:ABA0904
Leptodiaptomus novamexicanus|ZPLIV485-11|Mexico|BOLD:AAB2389
Leptodiaptomus novamexicanus|ZPLIV580-11|Mexico|BOLD:AAB2389
Leptodiaptomus novamexicanus|ZPLMX180-06|Mexico
Leptodiaptomus novamexicanus|ZPLMX927-06|Mexico|BOLD:AAB2389
Leptodiaptomus novamexicanus|ZPLMX182-06|Mexico|BOLD:AAB2389
Leptodiaptomus novamexicanus|ZPLMX936-06|Mexico|BOLD:AAB2389
Leptodiaptomus novamexicanus|ZPLMX183-06|Mexico|BOLD:AAB2389
Leptodiaptomus novamexicanus|ZPLMX921-06|Mexico|BOLD:AAB2389
Leptodiaptomus novamexicanus|ZPLMX181-06|Mexico|BOLD:AAB2389
Leptodiaptomus novamexicanus|ZPLMX939-06|Mexico|BOLD:AAB2389
Leptodiaptomus novamexicanus|ZPLMX933-06|Mexico|BOLD:AAB2389
Leptodiaptomus novamexicanus|ZPII190-07|Mexico|BOLD:AAB2389
Leptodiaptomus novamexicanus|ZPII191-07|Mexico|BOLD:AAB2389
Leptodiaptomus novamexicanus|ZPLMX940-06|Mexico|BOLD:AAB2389
Leptodiaptomus novamexicanus|ZPLMX937-06|Mexico|BOLD:AAB2389
Leptodiaptomus novamexicanus|ZPLMX934-06|Mexico|BOLD:AAB2389
Leptodiaptomus novamexicanus|ZPLMX929-06|Mexico|BOLD:AAB2389
Leptodiaptomus novamexicanus|ZPII189-07|Mexico|BOLD:AAB2389
Leptodiaptomus novamexicanus|ZPII192-07|Mexico|BOLD:AAB2389
Leptodiaptomus novamexicanus|ZPII193-07|Mexico|BOLD:AAB2389
Leptodiaptomus novamexicanus|ZPLMX938-06|Mexico|BOLD:AAB2389
Leptodiaptomus novamexicanus|ZPII195-07|Mexico|BOLD:AAB2389
Leptodiaptomus novamexicanus|ZPII194-07|Mexico|BOLD:AAB2389
Leptodiaptomus novamexicanus|ZPII197-07|Mexico|BOLD:AAB2389
[illegible]

Leptodiaptomus novamexicanus|ZPII19[illegible]-07|Mexico|BOLD:AAB2389
Leptodiaptomus novamexicanus|ZPII194-07|Mexico|BOLD:AAB2389
Leptodiaptomus novamexicanus|ZPII197-07|Mexico|BOLD:AAB2389
Leptodiaptomus novamexicanus|ZPLMX922-06|Mexico|BOLD:AAB2389
Leptodiaptomus novamexicanus|ZPII198-07|Mexico|BOLD:AAB2389
Leptodiaptomus novamexicanus|ZPII201-07|Mexico|BOLD:AAB2389
Leptodiaptomus novamexicanus|ZPII203-07|Mexico|BOLD:AAB2389
Leptodiaptomus novamexicanus|ZPII1410-11|Mexico|BOLD:AAB2389
Leptodiaptomus novamexicanus|ZPLIV706-11|Mexico|BOLD:AAB2389
Leptodiaptomus garciai|ZPII074-07|Mexico|BOLD:AAD0924
Leptodiaptomus garciai|GBA14293-13|Mexico|BOLD:AAD0924
Leptodiaptomus garciai|ZPII206-07|Mexico|BOLD:AAD0924
Leptodiaptomus garciai|ZPII630-07|Mexico|BOLD:AAD0924
Leptodiaptomus garciai|GBA14290-13|Mexico|BOLD:AAD0924
Leptodiaptomus garciai|ZPII204-07|Mexico|BOLD:AAD0924
Leptodiaptomus garciai|ZPLMX942-06|Mexico|BOLD:AAD0924
Leptodiaptomus garciai|GBA14289-13|Mexico|BOLD:AAD0924
Leptodiaptomus garciai|ZPII633-07|Mexico|BOLD:AAD0924
Leptodiaptomus garciai|ZPII068-07|Mexico|BOLD:AAD0924
Leptodiaptomus garciai|GBA14292-13|Mexico|BOLD:AAD0924
Leptodiaptomus garciai|ZPII076-07|Mexico|BOLD:AAD0924
Leptodiaptomus garciai|GBA14296-13|Mexico|BOLD:AAD0924
Leptodiaptomus garciai|ZPII629-07|Mexico|BOLD:AAD0924
Leptodiaptomus garciai|GBA14291-13|Mexico|BOLD:AAD0924
Leptodiaptomus garciai|ZPLMX928-06|Mexico
Leptodiaptomus garciai|ZPLMX924-06|Mexico|BOLD:AAD0924
Leptodiaptomus garciai|ZPII637-07|Mexico|BOLD:AAD0924
Leptodiaptomus garciai|GBA14300-13|Mexico|BOLD:AAD0924
Leptodiaptomus garciai|ZPII205-07|Mexico|BOLD:AAD0924
Leptodiaptomus garciai|GBA14295-13|Mexico|BOLD:AAD0924
Leptodiaptomus garciai|ZPII077-07|Mexico|BOLD:AAD0924
Leptodiaptomus garciai|ZPII635-07|Mexico|BOLD:AAD0924
Leptodiaptomus garciai|GBA14288-13|Mexico|BOLD:AAD0924
Leptodiaptomus garciai|ZPII208-07|Mexico|BOLD:AAD0924
Leptodiaptomus garciai|ZPII207-07|Mexico|BOLD:AAD0924
Leptodiaptomus garciai|ZPII071-07|Mexico|BOLD:AAD0924
Leptodiaptomus garciai|GBA14294-13|Mexico|BOLD:AAD0924
Leptodiaptomus garciai|ZPII636-07|Mexico|BOLD:AAD0924
Leptodiaptomus garciai|GBA14299-13|Mexico|BOLD:AAD0924
Leptodiaptomus garciai|ZPII645-07|Mexico|BOLD:AAD0924
Leptodiaptomus garciai|GBA14301-13|Mexico|BOLD:AAD0924
Leptodiaptomus garciai|ZPII639-07|Mexico|BOLD:AAD0924
Leptodiaptomus garciai|GBA14302-13|Mexico|BOLD:AAD0924
Leptodiaptomus cf. novamexicanus|ZPII200-07|Mexico|BOLD:AAB2390
Leptodiaptomus cf. novamexicanus|ZPII1324-11|Mexico|BOLD:AAB2390
Leptodiaptomus cf. novamexicanus|ZPII1325-11|Mexico|BOLD:AAB2390
Leptodiaptomus cf. novamexicanus|ZPII196-07|Mexico|BOLD:AAB2390
Leptodiaptomus cf. novamexicanus|ZPLMX935-06|Mexico|BOLD:AAB2390
Leptodiaptomus cf. novamexicanus|ZPLMX925-06|Mexico|BOLD:AAB2390
Leptodiaptomus cf. novamexicanus|ZPII199-07|Mexico|BOLD:AAB2390
Leptodiaptomus cf. novamexicanus|ZPII202-07|Mexico|BOLD:AAB2390
Leptodiaptomus cf. novamexicanus|GBA14307-13|Mexico|BOLD:AAB2390
Leptodiaptomus minutus|GLC208-06|Canada
Leptodiaptomus minutus|JMCRU243-09|Canada|BOLD:AAA2785
Leptodiaptomus minutus|COAPP376-13|Canada|BOLD:AAA2785
Leptodiaptomus minutus|BACZP1422-16|Canada|BOLD:AAA2785
Leptodiaptomus minutus|GLC209-06|Canada
Leptodiaptomus minutus|GLC210-06|Canada
Leptodiaptomus minutus|COAPP415-13|Canada|BOLD:AAA2785
Leptodiaptomus minutus|COAPP416-13|Canada|BOLD:AAA2785
Leptodiaptomus minutus|COAPP545-13|Canada|BOLD:AAA2785
Leptodiaptomus minutus|ZPII286-07|Canada|BOLD:AAA2785
Leptodiaptomus minutus|SACOP091-08|Canada|BOLD:AAA2785
Leptodiaptomus minutus|OZFWZ352-11|Canada|BOLD:AAA2785
Leptodiaptomus minutus|JMCRU241-09|Canada|BOLD:AAA2785
Leptodiaptomus minutus|BACZP1421-16|Canada|BOLD:AAA2785
Leptodiaptomus minutus|BACZP1379-16|Canada|BOLD:AAA2785
Leptodiaptomus minutus|BACZP1395-16|Canada|BOLD:AAA2785
Leptodiaptomus minutus|ZPII289-07|Canada|BOLD:AAA2785
Leptodiaptomus minutus|COAPP410-13|Canada|BOLD:AAA2785
Leptodiaptomus minutus|JMCRU249-09|Canada|BOLD:AAA2785
Leptodiaptomus minutus|ELPPC006-09|Canada|BOLD:AAA2785
Leptodiaptomus minutus|SWCHL2107-16|Canada|BOLD:AAA2785
Leptodiaptomus minutus|SWCHL2104-16|Canada|BOLD:AAA2785
Leptodiaptomus minutus|SWCHL2102-16|Canada|BOLD:AAA2785
Leptodiaptomus minutus|COAPP560-13|Canada|BOLD:AAA2785
Leptodiaptomus minutus|COAPP315-13|Canada|BOLD:AAA2785
Leptodiaptomus minutus|COAPP393-13|Canada|BOLD:AAA2785
Leptodiaptomus minutus|ELPPC007-09|Canada|BOLD:AAA2785
Leptodiaptomus minutus|GLC459-07|United States
Leptodiaptomus minutus|GLC408-06|United States
Diaptominae|ZPLMX982-06|Canada
Leptodiaptomus minutus|ZPII285-07|Canada|BOLD:AAA2785
Leptodiaptomus minutus|ZPII287-07|Canada|BOLD:AAA2785
Leptodiaptomus minutus|SWCHL2109-16|Canada|BOLD:AAA2785
Leptodiaptomus minutus|BACZP1381-16|Canada|BOLD:AAA2785
Leptodiaptomus minutus|BACZP1427-16|Canada|BOLD:AAA2785
Leptodiaptomus minutus|BACZP1501-16|Canada|BOLD:AAA2785
Leptodiaptomus minutus|BACZP1380-16|Canada|BOLD:AAA2785
Leptodiaptomus minutus|COAPP327-13|Canada|BOLD:AAA2785
Leptodiaptomus minutus|COAPP339-13|Canada|BOLD:AAA2785
Leptodiaptomus minutus|ZOOPS673-20|United States|BOLD:AAA2785
Leptodiaptomus minutus|ZOOPS674-20|United States|BOLD:AAA2785
Leptodiaptomus minutus|ZOOPS675-20|United States|BOLD:AAA2785
Hesperodiaptomus arcticus|NNMC379-08|Canada|BOLD:AAA4429
Hesperodiaptomus arcticus|NNMC410-08|Canada|BOLD:AAA4429
Hesperodiaptomus arcticus|NNMC411-08|Canada|BOLD:AAA4429
Hesperodiaptomus arcticus|NNMC413-08|Canada|BOLD:AAA4429

Hesperodiaptomus arcticus|NNMC410-08|Canada|BOLD:AAA4429
Hesperodiaptomus arcticus|NNMC411-08|Canada|BOLD:AAA4429
Hesperodiaptomus arcticus|NNMC413-08|Canada|BOLD:AAA4429
Hesperodiaptomus arcticus|NNMC414-08|Canada|BOLD:AAA4429
Hesperodiaptomus arcticus|DSMAX499-06|Canada|BOLD:AAA4427
Hesperodiaptomus arcticus|CRCH102-09|Canada|BOLD:AAA4427
Hesperodiaptomus arcticus|ZPII542-07|Canada|BOLD:AAA4427
Hesperodiaptomus arcticus sp. 3 CHU|OZFWZ419-11|Canada|BOLD:AAA4427
Hesperodiaptomus|ZMIII547-08|Canada|BOLD:AAA4427
Hesperodiaptomus arcticus sp. 3 CHU|OZFWZ426-11|Canada|BOLD:AAA4427
Hesperodiaptomus arcticus|ZPII321-07|Canada|BOLD:AAA4427
Hesperodiaptomus arcticus sp. 3 CHU|OZFWZ428-11|Canada|BOLD:AAA4427
Hesperodiaptomus arcticus|SACOP009-08|Canada|BOLD:AAA4427
Hesperodiaptomus arcticus sp. 3 CHU|OZFWZ453-11|Canada|BOLD:AAA4427
Hesperodiaptomus arcticus|ZPII318-07|Canada|BOLD:AAA4427
Hesperodiaptomus arcticus|ZPII495-07|Canada|BOLD:AAA4427
Hesperodiaptomus arcticus|ZPII496-07|Canada|BOLD:AAA4427
Hesperodiaptomus arcticus sp. 3 CHU|OZFWC959-12|Canada|BOLD:AAA4427
Hesperodiaptomus arcticus|SGRZC047-09|Canada|BOLD:AAA4427
Hesperodiaptomus arcticus|SACOP061-08|Canada|BOLD:AAA4427
Hesperodiaptomus arcticus sp. 3 CHU|OZFWZ412-11|Canada|BOLD:AAA4427
Hesperodiaptomus arcticus|DSMAX435-06|Canada|BOLD:AAA4427
Hesperodiaptomus arcticus|DSMAX356-06|Canada|BOLD:AAA4427
Hesperodiaptomus arcticus|DSMAX355-06|Canada|BOLD:AAA4427
Hesperodiaptomus arcticus|DSMAX332-06|Canada|BOLD:AAA4427
Hesperodiaptomus arcticus|SACOP063-08|Canada|BOLD:AAA4427
Hesperodiaptomus arcticus|CRCH100-09|Canada|BOLD:AAA4427
Hesperodiaptomus arcticus sp. 3 CHU|OZFWC701-12|Canada|BOLD:AAA4427
Hesperodiaptomus arcticus|SACOP051-08|Canada|BOLD:AAA4427
Hesperodiaptomus arcticus|SACOP032-08|Canada|BOLD:AAA4427
Hesperodiaptomus arcticus|ZPII514-07|Canada|BOLD:AAA4427
Hesperodiaptomus arcticus|DSMAX344-06|Canada|BOLD:AAA4427
Hesperodiaptomus arcticus sp. 3 CHU|OZFWZ433-11|Canada|BOLD:AAA4427
Hesperodiaptomus arcticus|DSMAX495-06|Canada|BOLD:AAA4427
Hesperodiaptomus arcticus|DSMAX494-06|Canada|BOLD:AAA4427
Hesperodiaptomus arcticus|DSMAX483-06|Canada|BOLD:AAA4427
Hesperodiaptomus arcticus|DSMAX471-06|Canada|BOLD:AAA4427
Hesperodiaptomus arcticus|DSMAX463-06|Canada|BOLD:AAA4427
Hesperodiaptomus arcticus|DSMAX459-06|Canada|BOLD:AAA4427
Hesperodiaptomus arcticus|DSMAX439-06|Canada|BOLD:AAA4427
Hesperodiaptomus arcticus|DSMAX427-06|Canada|BOLD:AAA4427
Hesperodiaptomus arcticus|DSMAX308-06|Canada|BOLD:AAA4427
Hesperodiaptomus arcticus|DSMAX379-06|Canada|BOLD:AAA4427
Hesperodiaptomus arcticus|DSMAX391-06|Canada|BOLD:AAA4427
Hesperodiaptomus arcticus|DSMAX423-06|Canada|BOLD:AAA4427
Hesperodiaptomus arcticus|DSMAX403-06|Canada|BOLD:AAA4427
Hesperodiaptomus arcticus|DSMAX516-06|Canada|BOLD:AAA4427
Hesperodiaptomus arcticus|DSMAX528-06|Canada|BOLD:AAA4427
Hesperodiaptomus arcticus|DSMAX346-06|Canada|BOLD:AAA4427
Hesperodiaptomus arcticus|ZPII322-07|Canada|BOLD:AAA4427
Hesperodiaptomus arcticus sp. 3 CHU|OZFWZ446-11|Canada|BOLD:AAA4427
Hesperodiaptomus arcticus sp. 3 CHU|OZFWZ434-11|Canada|BOLD:AAA4427
Hesperodiaptomus arcticus sp. 3 CHU|OZFWZ424-11|Canada|BOLD:AAA4427
Hesperodiaptomus arcticus sp. 3 CHU|OZFWC963-12|Canada|BOLD:AAA4427
Hesperodiaptomus arcticus sp. 3 CHU|OZFWZ432-11|Canada|BOLD:AAA4427
Hesperodiaptomus arcticus sp. 3 CHU|OZFWZ421-11|Canada|BOLD:AAA4427
Hesperodiaptomus arcticus sp. 3 CHU|OZFWZ467-11|Canada|BOLD:AAA4427
Hesperodiaptomus arcticus sp. 3 CHU|OZFWC259-11|Canada|BOLD:AAA4427
Hesperodiaptomus arcticus sp. 3 CHU|OZFWC226-11|Canada|BOLD:AAA4427
Hesperodiaptomus arcticus|DSMAX320-06|Canada|BOLD:AAA4427
Hesperodiaptomus arcticus|ZPII319-07|Canada|BOLD:AAA4427
Hesperodiaptomus arcticus sp. 3 CHU|OZFWC125-11|Canada|BOLD:AAA4427
Hesperodiaptomus arcticus sp. 3 CHU|OZFWZ459-11|Canada|BOLD:AAA4427
Hesperodiaptomus arcticus sp. 3 CHU|OZFWZ373-11|Canada|BOLD:AAA4427
Hesperodiaptomus arcticus sp. 3 CHU|OZFWC688-12|Canada|BOLD:AAA4427
Hesperodiaptomus arcticus sp. 3 CHU|OZFWC492-11|Canada|BOLD:AAA4427
Hesperodiaptomus arcticus sp. 3 CHU|OZFWC965-12|Canada|BOLD:AAA4427
Hesperodiaptomus arcticus sp. 3 CHU|OZFWZ429-11|Canada|BOLD:AAA4427
Hesperodiaptomus arcticus sp. 3 CHU|OZFWC817-12|Canada|BOLD:AAA4427
Hesperodiaptomus arcticus sp. 3 CHU|OZFWZ323-11|Canada|BOLD:AAA4427
Hesperodiaptomus arcticus sp. 3 CHU|OZFWZ320-11|Canada|BOLD:AAA4427
Hesperodiaptomus arcticus|NJCGS089-09|Canada|BOLD:AAA4427
Hesperodiaptomus arcticus|DSMAX534-06|Canada|BOLD:AAA4427
Hesperodiaptomus arcticus sp. 3 CHU|OZFWZ418-11|Canada|BOLD:AAA4427
Hesperodiaptomus arcticus sp. 3 CHU|OZFWZ440-11|Canada|BOLD:AAA4427
Hesperodiaptomus arcticus sp. 3 CHU|OZFWZ443-11|Canada|BOLD:AAA4427
Hesperodiaptomus arcticus sp. 3 CHU|OZFWZ444-11|Canada|BOLD:AAA4427
Hesperodiaptomus arcticus sp. 3 CHU|OZFWZ456-11|Canada|BOLD:AAA4427
Hesperodiaptomus arcticus sp. 3 CHU|OZFWZ461-11|Canada|BOLD:AAA4427
Hesperodiaptomus arcticus sp. 3 CHU|OZFWC128-11|Canada|BOLD:AAA4427
Hesperodiaptomus arcticus sp. 3 CHU|OZFWC181-11|Canada|BOLD:AAA4427
Hesperodiaptomus arcticus sp. 3 CHU|OZFWC211-11|Canada|BOLD:AAA4427
Hesperodiaptomus arcticus sp. 3 CHU|OZFWC214-11|Canada|BOLD:AAA4427
Hesperodiaptomus arcticus sp. 3 CHU|OZFWC218-11|Canada|BOLD:AAA4427
Hesperodiaptomus arcticus sp. 3 CHU|OZFWC273-11|Canada|BOLD:AAA4427
Hesperodiaptomus arcticus sp. 3 CHU|OZFWC386-11|Canada|BOLD:AAA4427
Hesperodiaptomus arcticus sp. 3 CHU|OZFWC393-11|Canada|BOLD:AAA4427
Hesperodiaptomus arcticus sp. 3 CHU|OZFWZ431-11|Canada|BOLD:AAA4427
Hesperodiaptomus arcticus sp. 3 CHU|OZFWZ435-11|Canada|BOLD:AAA4427
Hesperodiaptomus arcticus sp. 3 CHU|OZFWZ436-11|Canada|BOLD:AAA4427
Hesperodiaptomus arcticus sp. 3 CHU|OZFWZ438-11|Canada|BOLD:AAA4427
Hesperodiaptomus arcticus sp. 3 CHU|OZFWZ439-11|Canada|BOLD:AAA4427
Hesperodiaptomus arcticus sp. 3 CHU|OZFWZ441-11|Canada|BOLD:AAA4427
Hesperodiaptomus arcticus sp. 3 CHU|OZFWZ414-11|Canada|BOLD:AAA4427
Hesperodiaptomus arcticus sp. 3 CHU|OZFWZ415-11|Canada|BOLD:AAA4427
Hesperodiaptomus arcticus sp. 3 CHU|OZFWZ425-11|Canada|BOLD:AAA4427
Hesperodiaptomus arcticus sp. 3 CHU|OZFWZ430-11|Canada|BOLD:AAA4427
Hesperodiaptomus arcticus sp. 3 CHU|OZFWZ420-11|Canada|BOLD:AAA4427

[illegible]
Hesperodiaptomus arcticus sp. 3 CHU|OZFWZ425-11|Canada|BOLD:AAA4427
Hesperodiaptomus arcticus sp. 3 CHU|OZFWZ430-11|Canada|BOLD:AAA4427
Hesperodiaptomus arcticus sp. 3 CHU|OZFWZ420-11|Canada|BOLD:AAA4427
Hesperodiaptomus arcticus sp. 3 CHU|OZFWZ422-11|Canada|BOLD:AAA4427
Hesperodiaptomus arcticus|NJCGS073-09|Canada|BOLD:AAA4427
Hesperodiaptomus arcticus|NJCGS074-09|Canada|BOLD:AAA4427
Hesperodiaptomus arcticus|NJCGS075-09|Canada|BOLD:AAA4427
Hesperodiaptomus arcticus|NJCGS088-09|Canada|BOLD:AAA4427
Hesperodiaptomus arcticus|NJCGS090-09|Canada|BOLD:AAA4427
Hesperodiaptomus arcticus sp. 3 CHU|OZFWZ410-11|Canada|BOLD:AAA4427
Hesperodiaptomus arcticus|SGRZC014-09|Canada|BOLD:AAA4427
Hesperodiaptomus arcticus|SGRZC024-09|Canada|BOLD:AAA4427
Hesperodiaptomus arcticus|SGRZC004-09|Canada|BOLD:AAA4427
Hesperodiaptomus arcticus|SGRZC007-09|Canada|BOLD:AAA4427
Hesperodiaptomus arcticus|CRCH099-09|Canada|BOLD:AAA4427
Hesperodiaptomus arcticus|CRCH101-09|Canada|BOLD:AAA4427
Hesperodiaptomus arcticus|ELPPC041-09|Canada|BOLD:AAA4427
Hesperodiaptomus arcticus|ELPPC042-09|Canada|BOLD:AAA4427
Hesperodiaptomus arcticus|SACOP053-08|Canada|BOLD:AAA4427
Hesperodiaptomus arcticus|SACOP054-08|Canada|BOLD:AAA4427
Hesperodiaptomus arcticus|SACOP055-08|Canada|BOLD:AAA4427
Hesperodiaptomus arcticus|SACOP076-08|Canada|BOLD:AAA4427
Hesperodiaptomus arcticus sp. 3 CHU|OZFWC844-12|Canada|BOLD:AAA4427
Hesperodiaptomus arcticus sp. 3 CHU|OZFWC866-12|Canada|BOLD:AAA4427
Hesperodiaptomus arcticus sp. 3 CHU|OZFWC532-12|Canada|BOLD:AAA4427
Hesperodiaptomus arcticus sp. 3 CHU|OZFWC545-12|Canada|BOLD:AAA4427
Hesperodiaptomus arcticus sp. 3 CHU|OZFWC573-12|Canada|BOLD:AAA4427
Hesperodiaptomus arcticus sp. 3 CHU|OZFWC681-12|Canada|BOLD:AAA4427
Hesperodiaptomus arcticus sp. 3 CHU|OZFWC714-12|Canada|BOLD:AAA4427
Hesperodiaptomus arcticus sp. 3 CHU|OZFWC762-12|Canada|BOLD:AAA4427
Hesperodiaptomus arcticus sp. 3 CHU|OZFWC764-12|Canada|BOLD:AAA4427
Hesperodiaptomus arcticus sp. 3 CHU|OZFWC772-12|Canada|BOLD:AAA4427
Hesperodiaptomus arcticus sp. 3 CHU|OZFWC819-12|Canada|BOLD:AAA4427
Hesperodiaptomus arcticus sp. 3 CHU|OZFWC826-12|Canada|BOLD:AAA4427
Hesperodiaptomus arcticus sp. 3 CHU|OZFWZ454-11|Canada|BOLD:AAA4427
Hesperodiaptomus arcticus sp. 3 CHU|OZFWZ455-11|Canada|BOLD:AAA4427
Hesperodiaptomus arcticus sp. 3 CHU|OZFWZ445-11|Canada|BOLD:AAA4427
Hesperodiaptomus arcticus sp. 3 CHU|OZFWZ452-11|Canada|BOLD:AAA4427
Hesperodiaptomus arcticus|SACOP050-08|Canada|BOLD:AAA4427
Hesperodiaptomus arcticus|SACOP040-08|Canada|BOLD:AAA4427
Hesperodiaptomus arcticus|SACOP039-08|Canada|BOLD:AAA4427
Hesperodiaptomus arcticus|SACOP010-08|Canada|BOLD:AAA4427
Hesperodiaptomus arcticus|SACOP008-08|Canada|BOLD:AAA4427
Hesperodiaptomus arcticus|SACOP007-08|Canada|BOLD:AAA4427
Hesperodiaptomus arcticus|SACOP006-08|Canada|BOLD:AAA4427
Hesperodiaptomus arcticus|ZPII543-07|Canada|BOLD:AAA4427
Hesperodiaptomus arcticus|ZPII320-07|Canada|BOLD:AAA4427
Hesperodiaptomus arcticus|DSMAX541-06|Canada|BOLD:AAA4427
Hesperodiaptomus arcticus|DSMAX511-06|Canada|BOLD:AAA4427
Hesperodiaptomus arcticus|DSMAX522-06|Canada|BOLD:AAA4427
Hesperodiaptomus arcticus|DSMAX504-06|Canada|BOLD:AAA4427
Hesperodiaptomus arcticus sp. 3 CHU|OZFWZ458-11|Canada|BOLD:AAA4427
Hesperodiaptomus arcticus sp. 3 CHU|OZFWZ462-11|Canada|BOLD:AAA4427
Hesperodiaptomus arcticus sp. 3 CHU|OZFWZ466-11|Canada|BOLD:AAA4427
Hesperodiaptomus arcticus|SACOP079-08|Canada|BOLD:AAA4427
Hesperodiaptomus arcticus sp. 3 CHU|OZFWZ449-11|Canada|BOLD:AAA4427
Hesperodiaptomus arcticus sp. 3 CHU|OZFWC119-11|Canada|BOLD:AAA4427
Hesperodiaptomus arcticus sp. 3 CHU|OZFWC434-11|Canada|BOLD:AAA4427
Hesperodiaptomus arcticus sp. 3 CHU|OZFWC966-12|Canada|BOLD:AAA4427
Hesperodiaptomus arcticus|DSMAX510-06|Canada|BOLD:AAA4427
Hesperodiaptomus arcticus|DSMAX451-06|Canada|BOLD:AAA4427
Hesperodiaptomus arcticus sp. 3 CHU|OZFWZ324-11|Canada|BOLD:AAA4427
Hesperodiaptomus arcticus sp. 3 CHU|OZFWC967-12|Canada|BOLD:AAA4427
Hesperodiaptomus|GBA14389-13|Canada|BOLD:AAA4427
Hesperodiaptomus|ZMIII556-08|Canada|BOLD:AAA4427
Hesperodiaptomus|GBA14390-13|Canada|BOLD:AAA4427
Hesperodiaptomus|DNARA2985-21|Canada|BOLD:AAA4428
Hesperodiaptomus arcticus|DSMAX536-06|Canada
Hesperodiaptomus arcticus|SGRZC019-09|Canada|BOLD:AAA4428
Hesperodiaptomus arcticus|DSMAX467-06|Canada|BOLD:AAA4428
Hesperodiaptomus arcticus|SGRZC026-09|Canada|BOLD:AAA4428
Hesperodiaptomus arcticus|SGRZC018-09|Canada|BOLD:AAA4428
Hesperodiaptomus arcticus|SGRZC025-09|Canada|BOLD:AAA4428
Hesperodiaptomus arcticus|SGRZC027-09|Canada|BOLD:AAA4428
Hesperodiaptomus arcticus|SGRZC030-09|Canada|BOLD:AAA4428
Hesperodiaptomus arcticus|ELPPC045-09|Canada|BOLD:AAA4428
Hesperodiaptomus arcticus|SGRZC011-09|Canada|BOLD:AAA4428
Hesperodiaptomus arcticus|SGRZC045-09|Canada|BOLD:AAA4428
Hesperodiaptomus arcticus sp. 2 CHU|OZFWZ321-11|Canada|BOLD:AAA4428
Hesperodiaptomus arcticus sp. 2 CHU|OZFWZ411-11|Canada|BOLD:AAA4428
Hesperodiaptomus arcticus|ACHAR3270-19|Canada|BOLD:AAA4428
Hesperodiaptomus arcticus|DNARC236-19|Canada|BOLD:AAA4428
Hesperodiaptomus|DNARA2550-21|Canada|BOLD:AAA4428
Hesperodiaptomus arcticus|DNARC609-19|Canada|BOLD:AAA4428
Hesperodiaptomus|DNARA730-21|Canada|BOLD:AAA4428
Hesperodiaptomus arcticus sp. 2 CHU|OZFWZ293-11|Canada|BOLD:AAA4428
Hesperodiaptomus|DNARA757-21|Canada|BOLD:AAA4428
Hesperodiaptomus arcticus|ACHAR1355-18|Canada|BOLD:AAA4428
Hesperodiaptomus|DNARA759-21|Canada|BOLD:AAA4428
Hesperodiaptomus arcticus|ACHAR3250-19|Canada|BOLD:AAA4428
Hesperodiaptomus arcticus|ACHAR1364-18|Canada|BOLD:AAA4428
Hesperodiaptomus|GCHAR239-19|Canada|BOLD:AAA4428
Hesperodiaptomus|DNARA2114-21|Canada|BOLD:AAA4428
Hesperodiaptomus arcticus|ACHAR3255-19|Canada|BOLD:AAA4428
Hesperodiaptomus arcticus|ACHAR337-18|Canada|BOLD:AAA4428
Hesperodiaptomus arcticus sp. 2 CHU|OZFWZ448-11|Canada|BOLD:AAA4428
Hesperodiaptomus arcticus|DNARC606-19|Canada|BOLD:AAA4428

Hesperodiaptomus arcticus|ACHAR337-18|Canada|BOLD:AAA4428
Hesperodiaptomus arcticus sp. 2 CHU|OZFWZ448-11|Canada|BOLD:AAA4428
Hesperodiaptomus arcticus|DNARC606-19|Canada|BOLD:AAA4428
Hesperodiaptomus arcticus|ACHAR3252-19|Canada|BOLD:AAA4428
Hesperodiaptomus arcticus|DNARC608-19|Canada|BOLD:AAA4428
Hesperodiaptomus arcticus|ACHAR369-18|Canada|BOLD:AAA4428
Hesperodiaptomus arcticus|ACHAR1359-18|Canada|BOLD:AAA4428
Hesperodiaptomus|DNARA731-21|Canada|BOLD:AAA4428
Hesperodiaptomus arcticus|SACOP038-08|Canada|BOLD:AAA4428
Hesperodiaptomus arcticus|DNARC213-19|Canada|BOLD:AAA4428
Hesperodiaptomus arcticus sp. 2 CHU|OZFWZ460-11|Canada|BOLD:AAA4428
Hesperodiaptomus arcticus sp. 2 CHU|OZFWZ450-11|Canada|BOLD:AAA4428
Hesperodiaptomus arcticus|DNARC607-19|Canada|BOLD:AAA4428
Hesperodiaptomus arcticus|ACHAR1410-18|Canada|BOLD:AAA4428
Hesperodiaptomus arcticus|ACHAR1406-18|Canada|BOLD:AAA4428
Hesperodiaptomus|GCHAR241-19|Canada|BOLD:AAA4428
Hesperodiaptomus|GCHAR1136-19|Canada|BOLD:AAA4428
Hesperodiaptomus arcticus|DNARC625-19|Canada|BOLD:AAA4428
Hesperodiaptomus arcticus|ACHAR3247-19|Canada|BOLD:AAA4428
Hesperodiaptomus arcticus sp. 2 CHU|OZFWC291-11|Canada|BOLD:AAA4428
Hesperodiaptomus arcticus|ACHAR363-18|Canada|BOLD:AAA4428
Hesperodiaptomus arcticus|ACHAR362-18|Canada|BOLD:AAA4428
Hesperodiaptomus arcticus|SACOP066-08|Canada|BOLD:AAA4428
Hesperodiaptomus arcticus|ACHAR335-18|Canada|BOLD:AAA4428
Hesperodiaptomus|DNARA2176-21|Canada|BOLD:AAA4428
Hesperodiaptomus arcticus|ACHAR3269-19|Canada|BOLD:AAA4428
Hesperodiaptomus arcticus|ACHAR1404-18|Canada|BOLD:AAA4428
Hesperodiaptomus|DNARA760-21|Canada|BOLD:AAA4428
Hesperodiaptomus|DNARA2172-21|Canada|BOLD:AAA4428
Hesperodiaptomus arcticus|ACHAR3256-19|Canada|BOLD:AAA4428
Hesperodiaptomus|DNARA2146-21|Canada|BOLD:AAA4428
Hesperodiaptomus|DNARA2119-21|Canada|BOLD:AAA4428
Hesperodiaptomus|DNARA2120-21|Canada|BOLD:AAA4428
Hesperodiaptomus|DNARA2143-21|Canada|BOLD:AAA4428
Hesperodiaptomus|DNARA2174-21|Canada|BOLD:AAA4428
Hesperodiaptomus arcticus|ACHAR1395-18|Canada|BOLD:AAA4428
Hesperodiaptomus arcticus|ACHAR365-18|Canada|BOLD:AAA4428
Hesperodiaptomus arcticus|ACHAR3257-19|Canada|BOLD:AAA4428
Hesperodiaptomus arcticus|DNARC088-19|Canada|BOLD:AAA4428
Hesperodiaptomus arcticus|DNARC026-19|Canada|BOLD:AAA4428
Hesperodiaptomus arcticus|ACHAR3251-19|Canada|BOLD:AAA4428
Hesperodiaptomus arcticus sp. 2 CHU|OZFWC409-11|Canada|BOLD:AAA4428
Hesperodiaptomus arcticus|ELPPC046-09|Canada|BOLD:AAA4428
Hesperodiaptomus arcticus sp. 2 CHU|OZFWZ437-11|Canada|BOLD:AAA4428
Hesperodiaptomus arcticus|SGRZC048-09|Canada|BOLD:AAA4428
Hesperodiaptomus arcticus sp. 2 CHU|OZFWZ447-11|Canada|BOLD:AAA4428
Hesperodiaptomus arcticus sp. 2 CHU|OZFWC788-12|Canada|BOLD:AAA4428
Hesperodiaptomus|GCHAR278-19|Canada|BOLD:AAA4428
Hesperodiaptomus arcticus|ACHAR364-18|Canada|BOLD:AAA4428
Hesperodiaptomus arcticus|ACHAR336-18|Canada|BOLD:AAA4428
Hesperodiaptomus arcticus|ACHAR344-18|Canada|BOLD:AAA4428
Hesperodiaptomus arcticus|ACHAR1403-18|Canada|BOLD:AAA4428
Hesperodiaptomus arcticus|ACHAR361-18|Canada|BOLD:AAA4428
Hesperodiaptomus arcticus|ACHAR358-18|Canada|BOLD:AAA4428
Hesperodiaptomus arcticus|ACHAR327-18|Canada|BOLD:AAA4428
Hesperodiaptomus arcticus|ACHAR357-18|Canada|BOLD:AAA4428
Hesperodiaptomus arcticus|ACHAR1371-18|Canada|BOLD:AAA4428
Hesperodiaptomus arcticus|ACHAR359-18|Canada|BOLD:AAA4428
Hesperodiaptomus arcticus|ACHAR405-18|Canada|BOLD:AAA4428
Hesperodiaptomus|DNARA2549-21|Canada|BOLD:AAA4428
Hesperodiaptomus arcticus|ACHAR3248-19|Canada|BOLD:AAA4428
Hesperodiaptomus arcticus|ACHAR1394-18|Canada|BOLD:AAA4428
Hesperodiaptomus arcticus sp. 2 CHU|OZFWC292-11|Canada|BOLD:AAA4428
Hesperodiaptomus|DNARA2173-21|Canada|BOLD:AAA4428
Hesperodiaptomus arcticus|SACOP065-08|Canada|BOLD:AAA4428
Hesperodiaptomus arcticus sp. 2 CHU|OZFWZ401-11|Canada|BOLD:AAA4428
Hesperodiaptomus|DNARA2551-21|Canada|BOLD:AAA4428
Hesperodiaptomus|DNARA3098-21|Canada|BOLD:AAA4428
Hesperodiaptomus|DNARA3121-21|Canada|BOLD:AAA4428
Hesperodiaptomus arcticus sp. 2 CHU|OZFWZ427-11|Canada|BOLD:AAA4428
Hesperodiaptomus|DNARA2145-21|Canada|BOLD:AAA4428
Hesperodiaptomus|DNARA2144-21|Canada|BOLD:AAA4428
Hesperodiaptomus|DNARA2175-21|Canada|BOLD:AAA4428
Hesperodiaptomus|DNARA3405-21|Canada|BOLD:AAA4428
Hesperodiaptomus|DNARA2115-21|Canada|BOLD:AAA4428
Hesperodiaptomus|GJOA3581-21|Canada|BOLD:AAA4428
Hesperodiaptomus|DNARA2113-21|Canada|BOLD:AAA4428
Hesperodiaptomus|GJOA3584-21|Canada|BOLD:AAA4428
Hesperodiaptomus|GJOA3609-21|Canada|BOLD:AAA4428
Leptodiaptomus siciloides|GLC215-06|Canada
Leptodiaptomus siciloides|GLC221-06|Canada
Skistodiaptomus reighardi|GBMNE18508-21|United States|BOLD:AEM5029
Leptodiaptomus siciloides|GLC213-06|Canada
Leptodiaptomus siciloides|GLC214-06|Canada
Leptodiaptomus siciloides|GLC216-06|Canada
Skistodiaptomus reighardi|GBMNE18509-21|United States|BOLD:AEM5029
Skistodiaptomus sp. 2LMUSK061914|GBMNE18511-21|United States|BOLD:AEM5029
Nordodiaptomus alaskaensis|GCHAR587-19|Canada|BOLD:ADK1628
Skistodiaptomus|BCRUA041-10|United States|BOLD:AAV0654
Skistodiaptomus|BCRUA040-10|United States|BOLD:AAV0654
Skistodiaptomus|BCRUA044-10|United States|BOLD:AAV0654
Skistodiaptomus|BCRUA218-10|United States|BOLD:ACG1323
Skistodiaptomus|BBCRT010-12|United States|BOLD:ACG1323
Skistodiaptomus|BBCRT026-12|United States|BOLD:ACG1323
Skistodiaptomus|BCRUA220-10|United States|BOLD:ABX5000
Skistodiaptomus|BBCRT011-12|United States|BOLD:ABX5000
Skistodiaptomus|BBCRT025-12|United States|BOLD:ABX5000
Skistodiaptomus|BBCRT035-12|United States|BOLD:ABX5000

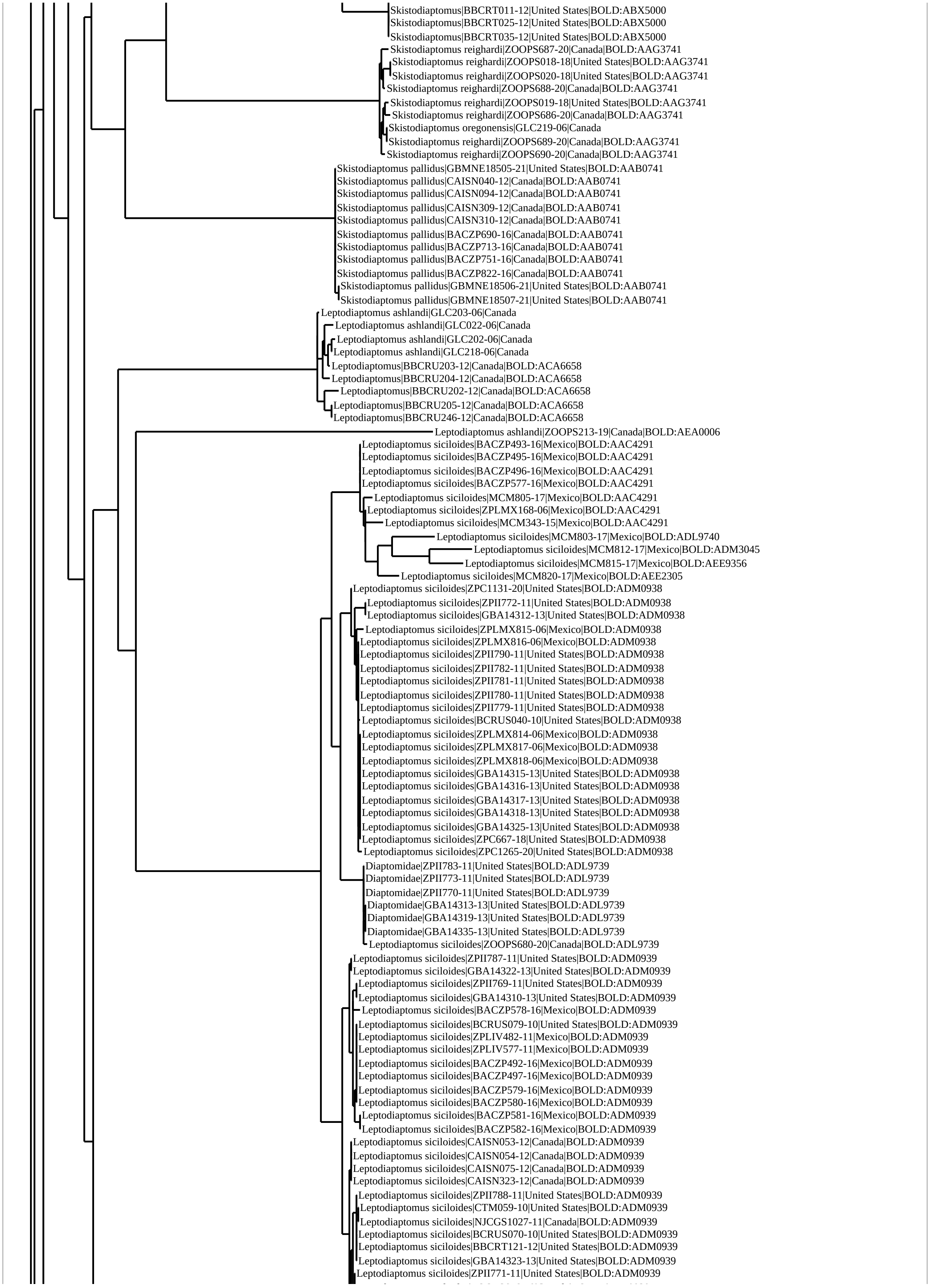
Skistodiaptomus|BBCRT011-12|United States|BOLD:ABX5000
Skistodiaptomus|BBCRT025-12|United States|BOLD:ABX5000
Skistodiaptomus|BBCRT035-12|United States|BOLD:ABX5000
Skistodiaptomus reighardi|ZOOPS687-20|Canada|BOLD:AAG3741
Skistodiaptomus reighardi|ZOOPS018-18|United States|BOLD:AAG3741
Skistodiaptomus reighardi|ZOOPS020-18|United States|BOLD:AAG3741
Skistodiaptomus reighardi|ZOOPS688-20|Canada|BOLD:AAG3741
Skistodiaptomus reighardi|ZOOPS019-18|United States|BOLD:AAG3741
Skistodiaptomus reighardi|ZOOPS686-20|Canada|BOLD:AAG3741
Skistodiaptomus oregonensis|GLC219-06|Canada
Skistodiaptomus reighardi|ZOOPS689-20|Canada|BOLD:AAG3741
Skistodiaptomus reighardi|ZOOPS690-20|Canada|BOLD:AAG3741
Skistodiaptomus pallidus|GBMNE18505-21|United States|BOLD:AAB0741
Skistodiaptomus pallidus|CAISN040-12|Canada|BOLD:AAB0741
Skistodiaptomus pallidus|CAISN094-12|Canada|BOLD:AAB0741
Skistodiaptomus pallidus|CAISN309-12|Canada|BOLD:AAB0741
Skistodiaptomus pallidus|CAISN310-12|Canada|BOLD:AAB0741
Skistodiaptomus pallidus|BACZP690-16|Canada|BOLD:AAB0741
Skistodiaptomus pallidus|BACZP713-16|Canada|BOLD:AAB0741
Skistodiaptomus pallidus|BACZP751-16|Canada|BOLD:AAB0741
Skistodiaptomus pallidus|BACZP822-16|Canada|BOLD:AAB0741
Skistodiaptomus pallidus|GBMNE18506-21|United States|BOLD:AAB0741
Skistodiaptomus pallidus|GBMNE18507-21|United States|BOLD:AAB0741
Leptodiaptomus ashlandi|GLC203-06|Canada
Leptodiaptomus ashlandi|GLC022-06|Canada
Leptodiaptomus ashlandi|GLC202-06|Canada
Leptodiaptomus ashlandi|GLC218-06|Canada
Leptodiaptomus|BBCRU203-12|Canada|BOLD:ACA6658
Leptodiaptomus|BBCRU204-12|Canada|BOLD:ACA6658
Leptodiaptomus|BBCRU202-12|Canada|BOLD:ACA6658
Leptodiaptomus|BBCRU205-12|Canada|BOLD:ACA6658
Leptodiaptomus|BBCRU246-12|Canada|BOLD:ACA6658
Leptodiaptomus ashlandi|ZOOPS213-19|Canada|BOLD:AEA0006
Leptodiaptomus siciloides|BACZP493-16|Mexico|BOLD:AAC4291
Leptodiaptomus siciloides|BACZP495-16|Mexico|BOLD:AAC4291
Leptodiaptomus siciloides|BACZP496-16|Mexico|BOLD:AAC4291
Leptodiaptomus siciloides|BACZP577-16|Mexico|BOLD:AAC4291
Leptodiaptomus siciloides|MCM805-17|Mexico|BOLD:AAC4291
Leptodiaptomus siciloides|ZPLMX168-06|Mexico|BOLD:AAC4291
Leptodiaptomus siciloides|MCM343-15|Mexico|BOLD:AAC4291
Leptodiaptomus siciloides|MCM803-17|Mexico|BOLD:ADL9740
Leptodiaptomus siciloides|MCM812-17|Mexico|BOLD:ADM3045
Leptodiaptomus siciloides|MCM815-17|Mexico|BOLD:AEE9356
Leptodiaptomus siciloides|MCM820-17|Mexico|BOLD:AEE2305
Leptodiaptomus siciloides|ZPC1131-20|United States|BOLD:ADM0938
Leptodiaptomus siciloides|ZPII772-11|United States|BOLD:ADM0938
Leptodiaptomus siciloides|GBA14312-13|United States|BOLD:ADM0938
Leptodiaptomus siciloides|ZPLMX815-06|Mexico|BOLD:ADM0938
Leptodiaptomus siciloides|ZPLMX816-06|Mexico|BOLD:ADM0938
Leptodiaptomus siciloides|ZPII790-11|United States|BOLD:ADM0938
Leptodiaptomus siciloides|ZPII782-11|United States|BOLD:ADM0938
Leptodiaptomus siciloides|ZPII781-11|United States|BOLD:ADM0938
Leptodiaptomus siciloides|ZPII780-11|United States|BOLD:ADM0938
Leptodiaptomus siciloides|ZPII779-11|United States|BOLD:ADM0938
Leptodiaptomus siciloides|BCRUS040-10|United States|BOLD:ADM0938
Leptodiaptomus siciloides|ZPLMX814-06|Mexico|BOLD:ADM0938
Leptodiaptomus siciloides|ZPLMX817-06|Mexico|BOLD:ADM0938
Leptodiaptomus siciloides|ZPLMX818-06|Mexico|BOLD:ADM0938
Leptodiaptomus siciloides|GBA14315-13|United States|BOLD:ADM0938
Leptodiaptomus siciloides|GBA14316-13|United States|BOLD:ADM0938
Leptodiaptomus siciloides|GBA14317-13|United States|BOLD:ADM0938
Leptodiaptomus siciloides|GBA14318-13|United States|BOLD:ADM0938
Leptodiaptomus siciloides|GBA14325-13|United States|BOLD:ADM0938
Leptodiaptomus siciloides|ZPC667-18|United States|BOLD:ADM0938
Leptodiaptomus siciloides|ZPC1265-20|United States|BOLD:ADM0938
Diaptomidae|ZPII783-11|United States|BOLD:ADL9739
Diaptomidae|ZPII773-11|United States|BOLD:ADL9739
Diaptomidae|ZPII770-11|United States|BOLD:ADL9739
Diaptomidae|GBA14313-13|United States|BOLD:ADL9739
Diaptomidae|GBA14319-13|United States|BOLD:ADL9739
Diaptomidae|GBA14335-13|United States|BOLD:ADL9739
Leptodiaptomus siciloides|ZOOPS680-20|Canada|BOLD:ADL9739
Leptodiaptomus siciloides|ZPII787-11|United States|BOLD:ADM0939
Leptodiaptomus siciloides|GBA14322-13|United States|BOLD:ADM0939
Leptodiaptomus siciloides|ZPII769-11|United States|BOLD:ADM0939
Leptodiaptomus siciloides|GBA14310-13|United States|BOLD:ADM0939
Leptodiaptomus siciloides|BACZP578-16|Mexico|BOLD:ADM0939
Leptodiaptomus siciloides|BCRUS079-10|United States|BOLD:ADM0939
Leptodiaptomus siciloides|ZPLIV482-11|Mexico|BOLD:ADM0939
Leptodiaptomus siciloides|ZPLIV577-11|Mexico|BOLD:ADM0939
Leptodiaptomus siciloides|BACZP492-16|Mexico|BOLD:ADM0939
Leptodiaptomus siciloides|BACZP497-16|Mexico|BOLD:ADM0939
Leptodiaptomus siciloides|BACZP579-16|Mexico|BOLD:ADM0939
Leptodiaptomus siciloides|BACZP580-16|Mexico|BOLD:ADM0939
Leptodiaptomus siciloides|BACZP581-16|Mexico|BOLD:ADM0939
Leptodiaptomus siciloides|BACZP582-16|Mexico|BOLD:ADM0939
Leptodiaptomus siciloides|CAISN053-12|Canada|BOLD:ADM0939
Leptodiaptomus siciloides|CAISN054-12|Canada|BOLD:ADM0939
Leptodiaptomus siciloides|CAISN075-12|Canada|BOLD:ADM0939
Leptodiaptomus siciloides|CAISN323-12|Canada|BOLD:ADM0939
Leptodiaptomus siciloides|ZPII788-11|United States|BOLD:ADM0939
Leptodiaptomus siciloides|CTM059-10|United States|BOLD:ADM0939
Leptodiaptomus siciloides|NJCGS1027-11|Canada|BOLD:ADM0939
Leptodiaptomus siciloides|BCRUS070-10|United States|BOLD:ADM0939
Leptodiaptomus siciloides|BBCRT121-12|United States|BOLD:ADM0939
Leptodiaptomus siciloides|GBA14323-13|United States|BOLD:ADM0939
Leptodiaptomus siciloides|ZPII771-11|United States|BOLD:ADM0939

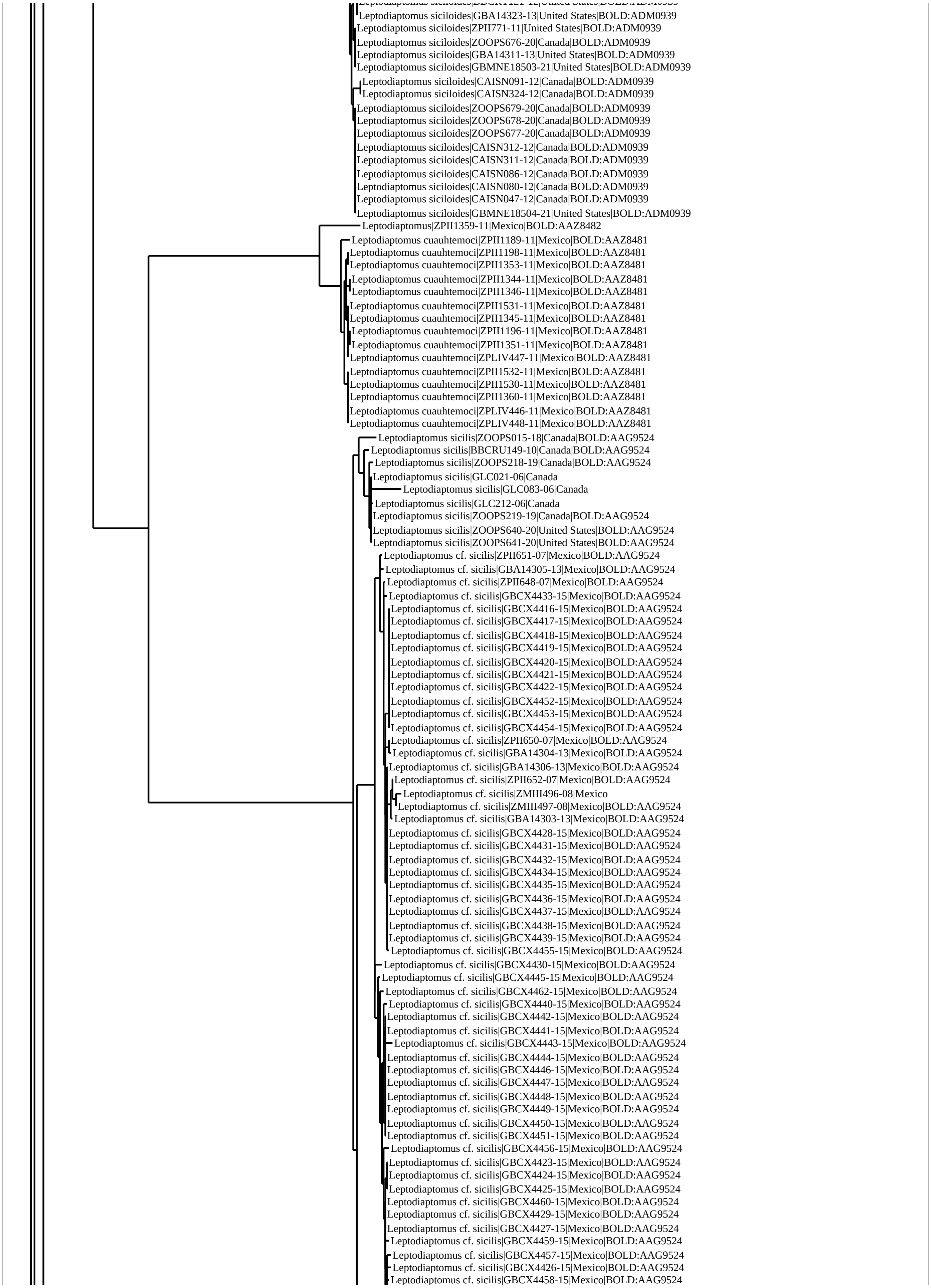
Leptodiaptomus siciloides|GBA14323-13|United States|BOLD:ADM0939
Leptodiaptomus siciloides|ZPII771-11|United States|BOLD:ADM0939
Leptodiaptomus siciloides|ZOOPS676-20|Canada|BOLD:ADM0939
Leptodiaptomus siciloides|GBA14311-13|United States|BOLD:ADM0939
Leptodiaptomus siciloides|GBMNE18503-21|United States|BOLD:ADM0939
Leptodiaptomus siciloides|CAISN091-12|Canada|BOLD:ADM0939
Leptodiaptomus siciloides|CAISN324-12|Canada|BOLD:ADM0939
Leptodiaptomus siciloides|ZOOPS679-20|Canada|BOLD:ADM0939
Leptodiaptomus siciloides|ZOOPS678-20|Canada|BOLD:ADM0939
Leptodiaptomus siciloides|ZOOPS677-20|Canada|BOLD:ADM0939
Leptodiaptomus siciloides|CAISN312-12|Canada|BOLD:ADM0939
Leptodiaptomus siciloides|CAISN311-12|Canada|BOLD:ADM0939
Leptodiaptomus siciloides|CAISN086-12|Canada|BOLD:ADM0939
Leptodiaptomus siciloides|CAISN080-12|Canada|BOLD:ADM0939
Leptodiaptomus siciloides|CAISN047-12|Canada|BOLD:ADM0939
Leptodiaptomus siciloides|GBMNE18504-21|United States|BOLD:ADM0939
Leptodiaptomus|ZPII1359-11|Mexico|BOLD:AAZ8482
Leptodiaptomus cuauhtemoci|ZPII1189-11|Mexico|BOLD:AAZ8481
Leptodiaptomus cuauhtemoci|ZPII1198-11|Mexico|BOLD:AAZ8481
Leptodiaptomus cuauhtemoci|ZPII1353-11|Mexico|BOLD:AAZ8481
Leptodiaptomus cuauhtemoci|ZPII1344-11|Mexico|BOLD:AAZ8481
Leptodiaptomus cuauhtemoci|ZPII1346-11|Mexico|BOLD:AAZ8481
Leptodiaptomus cuauhtemoci|ZPII1531-11|Mexico|BOLD:AAZ8481
Leptodiaptomus cuauhtemoci|ZPII1345-11|Mexico|BOLD:AAZ8481
Leptodiaptomus cuauhtemoci|ZPII1196-11|Mexico|BOLD:AAZ8481
Leptodiaptomus cuauhtemoci|ZPII1351-11|Mexico|BOLD:AAZ8481
Leptodiaptomus cuauhtemoci|ZPLIV447-11|Mexico|BOLD:AAZ8481
Leptodiaptomus cuauhtemoci|ZPII1532-11|Mexico|BOLD:AAZ8481
Leptodiaptomus cuauhtemoci|ZPII1530-11|Mexico|BOLD:AAZ8481
Leptodiaptomus cuauhtemoci|ZPII1360-11|Mexico|BOLD:AAZ8481
Leptodiaptomus cuauhtemoci|ZPLIV446-11|Mexico|BOLD:AAZ8481
Leptodiaptomus cuauhtemoci|ZPLIV448-11|Mexico|BOLD:AAZ8481
Leptodiaptomus sicilis|ZOOPS015-18|Canada|BOLD:AAG9524
Leptodiaptomus sicilis|BBCRU149-10|Canada|BOLD:AAG9524
Leptodiaptomus sicilis|ZOOPS218-19|Canada|BOLD:AAG9524
Leptodiaptomus sicilis|GLC021-06|Canada
Leptodiaptomus sicilis|GLC083-06|Canada
Leptodiaptomus sicilis|GLC212-06|Canada
Leptodiaptomus sicilis|ZOOPS219-19|Canada|BOLD:AAG9524
Leptodiaptomus sicilis|ZOOPS640-20|United States|BOLD:AAG9524
Leptodiaptomus sicilis|ZOOPS641-20|United States|BOLD:AAG9524
Leptodiaptomus cf. sicilis|ZPII651-07|Mexico|BOLD:AAG9524
Leptodiaptomus cf. sicilis|GBA14305-13|Mexico|BOLD:AAG9524
Leptodiaptomus cf. sicilis|ZPII648-07|Mexico|BOLD:AAG9524
Leptodiaptomus cf. sicilis|GBCX4433-15|Mexico|BOLD:AAG9524
Leptodiaptomus cf. sicilis|GBCX4416-15|Mexico|BOLD:AAG9524
Leptodiaptomus cf. sicilis|GBCX4417-15|Mexico|BOLD:AAG9524
Leptodiaptomus cf. sicilis|GBCX4418-15|Mexico|BOLD:AAG9524
Leptodiaptomus cf. sicilis|GBCX4419-15|Mexico|BOLD:AAG9524
Leptodiaptomus cf. sicilis|GBCX4420-15|Mexico|BOLD:AAG9524
Leptodiaptomus cf. sicilis|GBCX4421-15|Mexico|BOLD:AAG9524
Leptodiaptomus cf. sicilis|GBCX4422-15|Mexico|BOLD:AAG9524
Leptodiaptomus cf. sicilis|GBCX4452-15|Mexico|BOLD:AAG9524
Leptodiaptomus cf. sicilis|GBCX4453-15|Mexico|BOLD:AAG9524
Leptodiaptomus cf. sicilis|GBCX4454-15|Mexico|BOLD:AAG9524
Leptodiaptomus cf. sicilis|ZPII650-07|Mexico|BOLD:AAG9524
Leptodiaptomus cf. sicilis|GBA14304-13|Mexico|BOLD:AAG9524
Leptodiaptomus cf. sicilis|GBA14306-13|Mexico|BOLD:AAG9524
Leptodiaptomus cf. sicilis|ZPII652-07|Mexico|BOLD:AAG9524
Leptodiaptomus cf. sicilis|ZMIII496-08|Mexico
Leptodiaptomus cf. sicilis|ZMIII497-08|Mexico|BOLD:AAG9524
Leptodiaptomus cf. sicilis|GBA14303-13|Mexico|BOLD:AAG9524
Leptodiaptomus cf. sicilis|GBCX4428-15|Mexico|BOLD:AAG9524
Leptodiaptomus cf. sicilis|GBCX4431-15|Mexico|BOLD:AAG9524
Leptodiaptomus cf. sicilis|GBCX4432-15|Mexico|BOLD:AAG9524
Leptodiaptomus cf. sicilis|GBCX4434-15|Mexico|BOLD:AAG9524
Leptodiaptomus cf. sicilis|GBCX4435-15|Mexico|BOLD:AAG9524
Leptodiaptomus cf. sicilis|GBCX4436-15|Mexico|BOLD:AAG9524
Leptodiaptomus cf. sicilis|GBCX4437-15|Mexico|BOLD:AAG9524
Leptodiaptomus cf. sicilis|GBCX4438-15|Mexico|BOLD:AAG9524
Leptodiaptomus cf. sicilis|GBCX4439-15|Mexico|BOLD:AAG9524
Leptodiaptomus cf. sicilis|GBCX4455-15|Mexico|BOLD:AAG9524
Leptodiaptomus cf. sicilis|GBCX4430-15|Mexico|BOLD:AAG9524
Leptodiaptomus cf. sicilis|GBCX4445-15|Mexico|BOLD:AAG9524
Leptodiaptomus cf. sicilis|GBCX4462-15|Mexico|BOLD:AAG9524
Leptodiaptomus cf. sicilis|GBCX4440-15|Mexico|BOLD:AAG9524
Leptodiaptomus cf. sicilis|GBCX4442-15|Mexico|BOLD:AAG9524
Leptodiaptomus cf. sicilis|GBCX4441-15|Mexico|BOLD:AAG9524
Leptodiaptomus cf. sicilis|GBCX4443-15|Mexico|BOLD:AAG9524
Leptodiaptomus cf. sicilis|GBCX4444-15|Mexico|BOLD:AAG9524
Leptodiaptomus cf. sicilis|GBCX4446-15|Mexico|BOLD:AAG9524
Leptodiaptomus cf. sicilis|GBCX4447-15|Mexico|BOLD:AAG9524
Leptodiaptomus cf. sicilis|GBCX4448-15|Mexico|BOLD:AAG9524
Leptodiaptomus cf. sicilis|GBCX4449-15|Mexico|BOLD:AAG9524
Leptodiaptomus cf. sicilis|GBCX4450-15|Mexico|BOLD:AAG9524
Leptodiaptomus cf. sicilis|GBCX4451-15|Mexico|BOLD:AAG9524
Leptodiaptomus cf. sicilis|GBCX4456-15|Mexico|BOLD:AAG9524
Leptodiaptomus cf. sicilis|GBCX4423-15|Mexico|BOLD:AAG9524
Leptodiaptomus cf. sicilis|GBCX4424-15|Mexico|BOLD:AAG9524
Leptodiaptomus cf. sicilis|GBCX4425-15|Mexico|BOLD:AAG9524
Leptodiaptomus cf. sicilis|GBCX4460-15|Mexico|BOLD:AAG9524
Leptodiaptomus cf. sicilis|GBCX4429-15|Mexico|BOLD:AAG9524
Leptodiaptomus cf. sicilis|GBCX4427-15|Mexico|BOLD:AAG9524
Leptodiaptomus cf. sicilis|GBCX4459-15|Mexico|BOLD:AAG9524
Leptodiaptomus cf. sicilis|GBCX4457-15|Mexico|BOLD:AAG9524
Leptodiaptomus cf. sicilis|GBCX4426-15|Mexico|BOLD:AAG9524
Leptodiaptomus cf. sicilis|GBCX4458-15|Mexico|BOLD:AAG9524

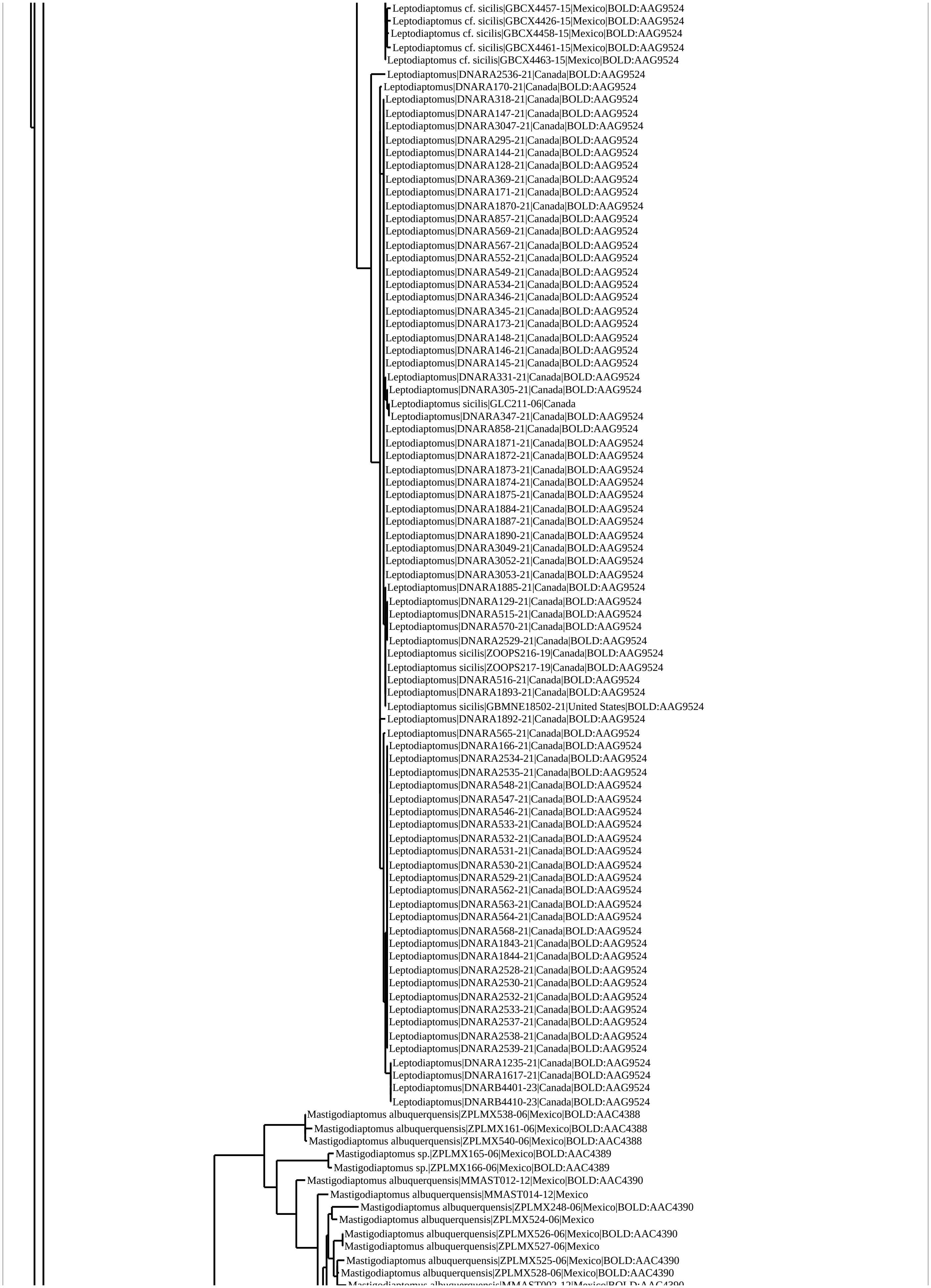
Leptodiaptomus cf. sicilis|GBCX4457-15|Mexico|BOLD:AAG9524
Leptodiaptomus cf. sicilis|GBCX4426-15|Mexico|BOLD:AAG9524
Leptodiaptomus cf. sicilis|GBCX4458-15|Mexico|BOLD:AAG9524
Leptodiaptomus cf. sicilis|GBCX4461-15|Mexico|BOLD:AAG9524
Leptodiaptomus cf. sicilis|GBCX4463-15|Mexico|BOLD:AAG9524
Leptodiaptomus|DNARA2536-21|Canada|BOLD:AAG9524
Leptodiaptomus|DNARA170-21|Canada|BOLD:AAG9524
Leptodiaptomus|DNARA318-21|Canada|BOLD:AAG9524
Leptodiaptomus|DNARA147-21|Canada|BOLD:AAG9524
Leptodiaptomus|DNARA3047-21|Canada|BOLD:AAG9524
Leptodiaptomus|DNARA295-21|Canada|BOLD:AAG9524
Leptodiaptomus|DNARA144-21|Canada|BOLD:AAG9524
Leptodiaptomus|DNARA128-21|Canada|BOLD:AAG9524
Leptodiaptomus|DNARA369-21|Canada|BOLD:AAG9524
Leptodiaptomus|DNARA171-21|Canada|BOLD:AAG9524
Leptodiaptomus|DNARA1870-21|Canada|BOLD:AAG9524
Leptodiaptomus|DNARA857-21|Canada|BOLD:AAG9524
Leptodiaptomus|DNARA569-21|Canada|BOLD:AAG9524
Leptodiaptomus|DNARA567-21|Canada|BOLD:AAG9524
Leptodiaptomus|DNARA552-21|Canada|BOLD:AAG9524
Leptodiaptomus|DNARA549-21|Canada|BOLD:AAG9524
Leptodiaptomus|DNARA534-21|Canada|BOLD:AAG9524
Leptodiaptomus|DNARA346-21|Canada|BOLD:AAG9524
Leptodiaptomus|DNARA345-21|Canada|BOLD:AAG9524
Leptodiaptomus|DNARA173-21|Canada|BOLD:AAG9524
Leptodiaptomus|DNARA148-21|Canada|BOLD:AAG9524
Leptodiaptomus|DNARA146-21|Canada|BOLD:AAG9524
Leptodiaptomus|DNARA145-21|Canada|BOLD:AAG9524
Leptodiaptomus|DNARA331-21|Canada|BOLD:AAG9524
Leptodiaptomus|DNARA305-21|Canada|BOLD:AAG9524
Leptodiaptomus sicilis|GLC211-06|Canada
Leptodiaptomus|DNARA347-21|Canada|BOLD:AAG9524
Leptodiaptomus|DNARA858-21|Canada|BOLD:AAG9524
Leptodiaptomus|DNARA1871-21|Canada|BOLD:AAG9524
Leptodiaptomus|DNARA1872-21|Canada|BOLD:AAG9524
Leptodiaptomus|DNARA1873-21|Canada|BOLD:AAG9524
Leptodiaptomus|DNARA1874-21|Canada|BOLD:AAG9524
Leptodiaptomus|DNARA1875-21|Canada|BOLD:AAG9524
Leptodiaptomus|DNARA1884-21|Canada|BOLD:AAG9524
Leptodiaptomus|DNARA1887-21|Canada|BOLD:AAG9524
Leptodiaptomus|DNARA1890-21|Canada|BOLD:AAG9524
Leptodiaptomus|DNARA3049-21|Canada|BOLD:AAG9524
Leptodiaptomus|DNARA3052-21|Canada|BOLD:AAG9524
Leptodiaptomus|DNARA3053-21|Canada|BOLD:AAG9524
Leptodiaptomus|DNARA1885-21|Canada|BOLD:AAG9524
Leptodiaptomus|DNARA129-21|Canada|BOLD:AAG9524
Leptodiaptomus|DNARA515-21|Canada|BOLD:AAG9524
Leptodiaptomus|DNARA570-21|Canada|BOLD:AAG9524
Leptodiaptomus|DNARA2529-21|Canada|BOLD:AAG9524
Leptodiaptomus sicilis|ZOOPS216-19|Canada|BOLD:AAG9524
Leptodiaptomus sicilis|ZOOPS217-19|Canada|BOLD:AAG9524
Leptodiaptomus|DNARA516-21|Canada|BOLD:AAG9524
Leptodiaptomus|DNARA1893-21|Canada|BOLD:AAG9524
Leptodiaptomus sicilis|GBMNE18502-21|United States|BOLD:AAG9524
Leptodiaptomus|DNARA1892-21|Canada|BOLD:AAG9524
Leptodiaptomus|DNARA565-21|Canada|BOLD:AAG9524
Leptodiaptomus|DNARA166-21|Canada|BOLD:AAG9524
Leptodiaptomus|DNARA2534-21|Canada|BOLD:AAG9524
Leptodiaptomus|DNARA2535-21|Canada|BOLD:AAG9524
Leptodiaptomus|DNARA548-21|Canada|BOLD:AAG9524
Leptodiaptomus|DNARA547-21|Canada|BOLD:AAG9524
Leptodiaptomus|DNARA546-21|Canada|BOLD:AAG9524
Leptodiaptomus|DNARA533-21|Canada|BOLD:AAG9524
Leptodiaptomus|DNARA532-21|Canada|BOLD:AAG9524
Leptodiaptomus|DNARA531-21|Canada|BOLD:AAG9524
Leptodiaptomus|DNARA530-21|Canada|BOLD:AAG9524
Leptodiaptomus|DNARA529-21|Canada|BOLD:AAG9524
Leptodiaptomus|DNARA562-21|Canada|BOLD:AAG9524
Leptodiaptomus|DNARA563-21|Canada|BOLD:AAG9524
Leptodiaptomus|DNARA564-21|Canada|BOLD:AAG9524
Leptodiaptomus|DNARA568-21|Canada|BOLD:AAG9524
Leptodiaptomus|DNARA1843-21|Canada|BOLD:AAG9524
Leptodiaptomus|DNARA1844-21|Canada|BOLD:AAG9524
Leptodiaptomus|DNARA2528-21|Canada|BOLD:AAG9524
Leptodiaptomus|DNARA2530-21|Canada|BOLD:AAG9524
Leptodiaptomus|DNARA2532-21|Canada|BOLD:AAG9524
Leptodiaptomus|DNARA2533-21|Canada|BOLD:AAG9524
Leptodiaptomus|DNARA2537-21|Canada|BOLD:AAG9524
Leptodiaptomus|DNARA2538-21|Canada|BOLD:AAG9524
Leptodiaptomus|DNARA2539-21|Canada|BOLD:AAG9524
Leptodiaptomus|DNARA1235-21|Canada|BOLD:AAG9524
Leptodiaptomus|DNARA1617-21|Canada|BOLD:AAG9524
Leptodiaptomus|DNARB4401-23|Canada|BOLD:AAG9524
Leptodiaptomus|DNARB4410-23|Canada|BOLD:AAG9524
Mastigodiaptomus albuquerquensis|ZPLMX538-06|Mexico|BOLD:AAC4388
Mastigodiaptomus albuquerquensis|ZPLMX161-06|Mexico|BOLD:AAC4388
Mastigodiaptomus albuquerquensis|ZPLMX540-06|Mexico|BOLD:AAC4388
Mastigodiaptomus sp.|ZPLMX165-06|Mexico|BOLD:AAC4389
Mastigodiaptomus sp.|ZPLMX166-06|Mexico|BOLD:AAC4389
Mastigodiaptomus albuquerquensis|MMAST012-12|Mexico|BOLD:AAC4390
Mastigodiaptomus albuquerquensis|MMAST014-12|Mexico
Mastigodiaptomus albuquerquensis|ZPLMX248-06|Mexico|BOLD:AAC4390
Mastigodiaptomus albuquerquensis|ZPLMX524-06|Mexico
Mastigodiaptomus albuquerquensis|ZPLMX526-06|Mexico|BOLD:AAC4390
Mastigodiaptomus albuquerquensis|ZPLMX527-06|Mexico
Mastigodiaptomus albuquerquensis|ZPLMX525-06|Mexico|BOLD:AAC4390
Mastigodiaptomus albuquerquensis|ZPLMX528-06|Mexico|BOLD:AAC4390

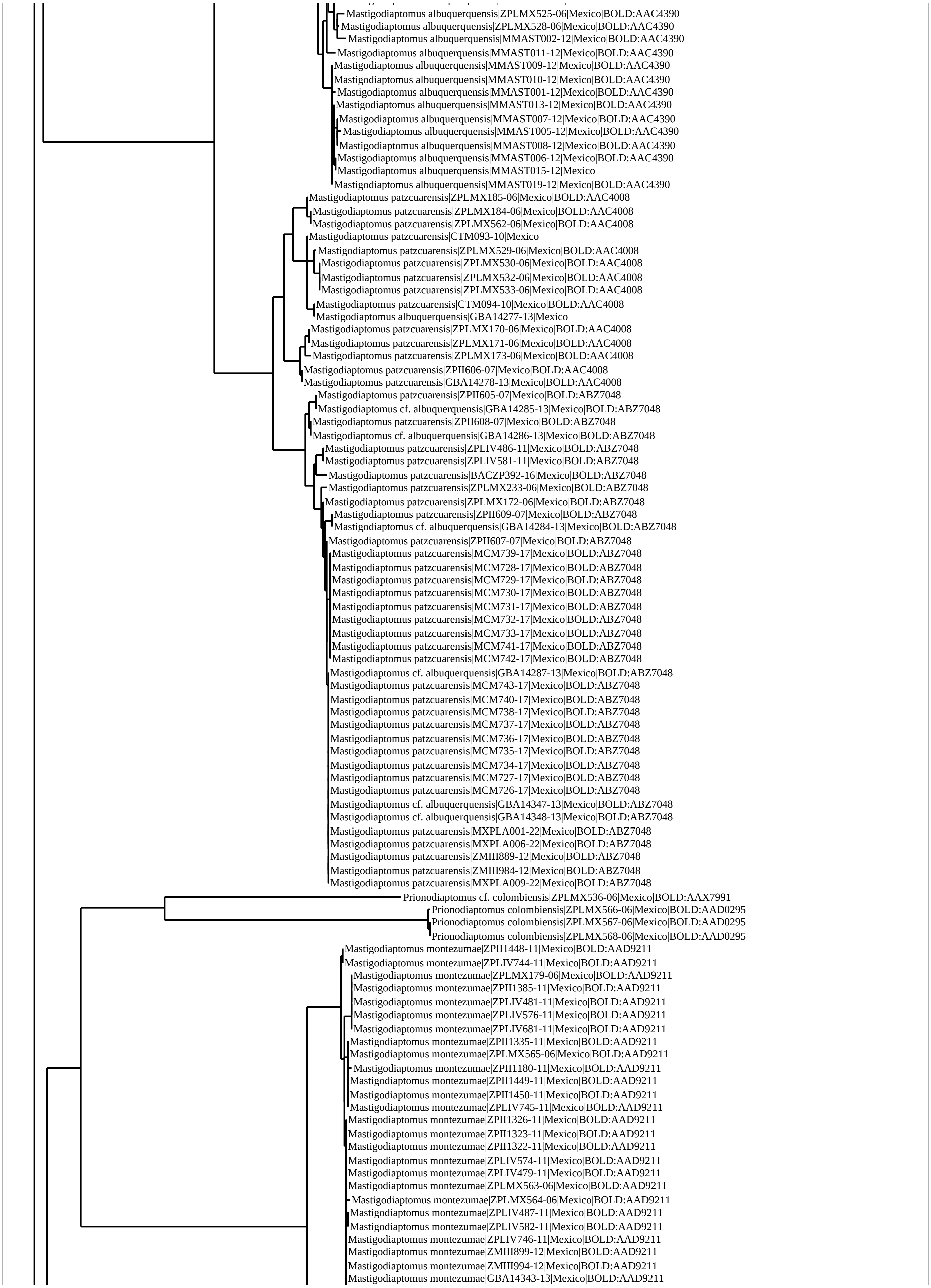
Mastigodiaptomus albuquerquensis|ZPLMX525-06|Mexico|BOLD:AAC4390
Mastigodiaptomus albuquerquensis|ZPLMX528-06|Mexico|BOLD:AAC4390
Mastigodiaptomus albuquerquensis|MMAST002-12|Mexico|BOLD:AAC4390
Mastigodiaptomus albuquerquensis|MMAST011-12|Mexico|BOLD:AAC4390
Mastigodiaptomus albuquerquensis|MMAST009-12|Mexico|BOLD:AAC4390
Mastigodiaptomus albuquerquensis|MMAST010-12|Mexico|BOLD:AAC4390
Mastigodiaptomus albuquerquensis|MMAST001-12|Mexico|BOLD:AAC4390
Mastigodiaptomus albuquerquensis|MMAST013-12|Mexico|BOLD:AAC4390
Mastigodiaptomus albuquerquensis|MMAST007-12|Mexico|BOLD:AAC4390
Mastigodiaptomus albuquerquensis|MMAST005-12|Mexico|BOLD:AAC4390
Mastigodiaptomus albuquerquensis|MMAST008-12|Mexico|BOLD:AAC4390
Mastigodiaptomus albuquerquensis|MMAST006-12|Mexico|BOLD:AAC4390
Mastigodiaptomus albuquerquensis|MMAST015-12|Mexico
Mastigodiaptomus albuquerquensis|MMAST019-12|Mexico|BOLD:AAC4390
Mastigodiaptomus patzcuarensis|ZPLMX185-06|Mexico|BOLD:AAC4008
Mastigodiaptomus patzcuarensis|ZPLMX184-06|Mexico|BOLD:AAC4008
Mastigodiaptomus patzcuarensis|ZPLMX562-06|Mexico|BOLD:AAC4008
Mastigodiaptomus patzcuarensis|CTM093-10|Mexico
Mastigodiaptomus patzcuarensis|ZPLMX529-06|Mexico|BOLD:AAC4008
Mastigodiaptomus patzcuarensis|ZPLMX530-06|Mexico|BOLD:AAC4008
Mastigodiaptomus patzcuarensis|ZPLMX532-06|Mexico|BOLD:AAC4008
Mastigodiaptomus patzcuarensis|ZPLMX533-06|Mexico|BOLD:AAC4008
Mastigodiaptomus patzcuarensis|CTM094-10|Mexico|BOLD:AAC4008
Mastigodiaptomus albuquerquensis|GBA14277-13|Mexico
Mastigodiaptomus patzcuarensis|ZPLMX170-06|Mexico|BOLD:AAC4008
Mastigodiaptomus patzcuarensis|ZPLMX171-06|Mexico|BOLD:AAC4008
Mastigodiaptomus patzcuarensis|ZPLMX173-06|Mexico|BOLD:AAC4008
Mastigodiaptomus patzcuarensis|ZPII606-07|Mexico|BOLD:AAC4008
Mastigodiaptomus patzcuarensis|GBA14278-13|Mexico|BOLD:AAC4008
Mastigodiaptomus patzcuarensis|ZPII605-07|Mexico|BOLD:ABZ7048
Mastigodiaptomus cf. albuquerquensis|GBA14285-13|Mexico|BOLD:ABZ7048
Mastigodiaptomus patzcuarensis|ZPII608-07|Mexico|BOLD:ABZ7048
Mastigodiaptomus cf. albuquerquensis|GBA14286-13|Mexico|BOLD:ABZ7048
Mastigodiaptomus patzcuarensis|ZPLIV486-11|Mexico|BOLD:ABZ7048
Mastigodiaptomus patzcuarensis|ZPLIV581-11|Mexico|BOLD:ABZ7048
Mastigodiaptomus patzcuarensis|BACZP392-16|Mexico|BOLD:ABZ7048
Mastigodiaptomus patzcuarensis|ZPLMX233-06|Mexico|BOLD:ABZ7048
Mastigodiaptomus patzcuarensis|ZPLMX172-06|Mexico|BOLD:ABZ7048
Mastigodiaptomus patzcuarensis|ZPII609-07|Mexico|BOLD:ABZ7048
Mastigodiaptomus cf. albuquerquensis|GBA14284-13|Mexico|BOLD:ABZ7048
Mastigodiaptomus patzcuarensis|ZPII607-07|Mexico|BOLD:ABZ7048
Mastigodiaptomus patzcuarensis|MCM739-17|Mexico|BOLD:ABZ7048
Mastigodiaptomus patzcuarensis|MCM728-17|Mexico|BOLD:ABZ7048
Mastigodiaptomus patzcuarensis|MCM729-17|Mexico|BOLD:ABZ7048
Mastigodiaptomus patzcuarensis|MCM730-17|Mexico|BOLD:ABZ7048
Mastigodiaptomus patzcuarensis|MCM731-17|Mexico|BOLD:ABZ7048
Mastigodiaptomus patzcuarensis|MCM732-17|Mexico|BOLD:ABZ7048
Mastigodiaptomus patzcuarensis|MCM733-17|Mexico|BOLD:ABZ7048
Mastigodiaptomus patzcuarensis|MCM741-17|Mexico|BOLD:ABZ7048
Mastigodiaptomus patzcuarensis|MCM742-17|Mexico|BOLD:ABZ7048
Mastigodiaptomus cf. albuquerquensis|GBA14287-13|Mexico|BOLD:ABZ7048
Mastigodiaptomus patzcuarensis|MCM743-17|Mexico|BOLD:ABZ7048
Mastigodiaptomus patzcuarensis|MCM740-17|Mexico|BOLD:ABZ7048
Mastigodiaptomus patzcuarensis|MCM738-17|Mexico|BOLD:ABZ7048
Mastigodiaptomus patzcuarensis|MCM737-17|Mexico|BOLD:ABZ7048
Mastigodiaptomus patzcuarensis|MCM736-17|Mexico|BOLD:ABZ7048
Mastigodiaptomus patzcuarensis|MCM735-17|Mexico|BOLD:ABZ7048
Mastigodiaptomus patzcuarensis|MCM734-17|Mexico|BOLD:ABZ7048
Mastigodiaptomus patzcuarensis|MCM727-17|Mexico|BOLD:ABZ7048
Mastigodiaptomus patzcuarensis|MCM726-17|Mexico|BOLD:ABZ7048
Mastigodiaptomus cf. albuquerquensis|GBA14347-13|Mexico|BOLD:ABZ7048
Mastigodiaptomus cf. albuquerquensis|GBA14348-13|Mexico|BOLD:ABZ7048
Mastigodiaptomus patzcuarensis|MXPLA001-22|Mexico|BOLD:ABZ7048
Mastigodiaptomus patzcuarensis|MXPLA006-22|Mexico|BOLD:ABZ7048
Mastigodiaptomus patzcuarensis|ZMIII889-12|Mexico|BOLD:ABZ7048
Mastigodiaptomus patzcuarensis|ZMIII984-12|Mexico|BOLD:ABZ7048
Mastigodiaptomus patzcuarensis|MXPLA009-22|Mexico|BOLD:ABZ7048
Prionodiaptomus cf. colombiensis|ZPLMX536-06|Mexico|BOLD:AAX7991
Prionodiaptomus colombiensis|ZPLMX566-06|Mexico|BOLD:AAD0295
Prionodiaptomus colombiensis|ZPLMX567-06|Mexico|BOLD:AAD0295
Prionodiaptomus colombiensis|ZPLMX568-06|Mexico|BOLD:AAD0295
Mastigodiaptomus montezumae|ZPII1448-11|Mexico|BOLD:AAD9211
Mastigodiaptomus montezumae|ZPLIV744-11|Mexico|BOLD:AAD9211
Mastigodiaptomus montezumae|ZPLMX179-06|Mexico|BOLD:AAD9211
Mastigodiaptomus montezumae|ZPII1385-11|Mexico|BOLD:AAD9211
Mastigodiaptomus montezumae|ZPLIV481-11|Mexico|BOLD:AAD9211
Mastigodiaptomus montezumae|ZPLIV576-11|Mexico|BOLD:AAD9211
Mastigodiaptomus montezumae|ZPLIV681-11|Mexico|BOLD:AAD9211
Mastigodiaptomus montezumae|ZPII1335-11|Mexico|BOLD:AAD9211
Mastigodiaptomus montezumae|ZPLMX565-06|Mexico|BOLD:AAD9211
Mastigodiaptomus montezumae|ZPII1180-11|Mexico|BOLD:AAD9211
Mastigodiaptomus montezumae|ZPII1449-11|Mexico|BOLD:AAD9211
Mastigodiaptomus montezumae|ZPII1450-11|Mexico|BOLD:AAD9211
Mastigodiaptomus montezumae|ZPLIV745-11|Mexico|BOLD:AAD9211
Mastigodiaptomus montezumae|ZPII1326-11|Mexico|BOLD:AAD9211
Mastigodiaptomus montezumae|ZPII1323-11|Mexico|BOLD:AAD9211
Mastigodiaptomus montezumae|ZPII1322-11|Mexico|BOLD:AAD9211
Mastigodiaptomus montezumae|ZPLIV574-11|Mexico|BOLD:AAD9211
Mastigodiaptomus montezumae|ZPLIV479-11|Mexico|BOLD:AAD9211
Mastigodiaptomus montezumae|ZPLMX563-06|Mexico|BOLD:AAD9211
Mastigodiaptomus montezumae|ZPLMX564-06|Mexico|BOLD:AAD9211
Mastigodiaptomus montezumae|ZPLIV487-11|Mexico|BOLD:AAD9211
Mastigodiaptomus montezumae|ZPLIV582-11|Mexico|BOLD:AAD9211
Mastigodiaptomus montezumae|ZPLIV746-11|Mexico|BOLD:AAD9211
Mastigodiaptomus montezumae|ZMIII899-12|Mexico|BOLD:AAD9211
Mastigodiaptomus montezumae|ZMIII994-12|Mexico|BOLD:AAD9211
Mastigodiaptomus montezumae|GBA14343-13|Mexico|BOLD:AAD9211

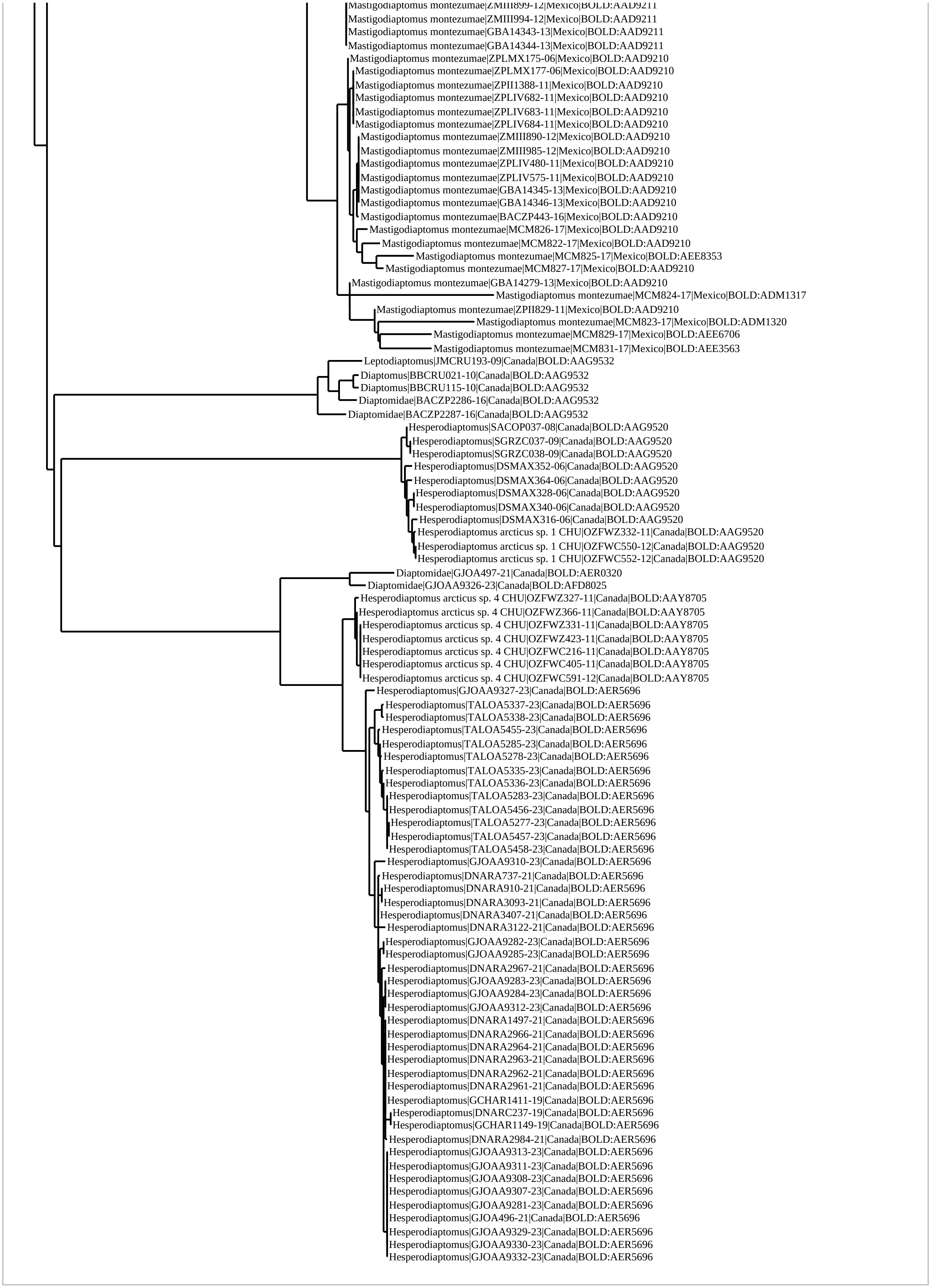
Mastigodiaptomus montezumae|ZMIII899-12|Mexico|BOLD:AAD9211
Mastigodiaptomus montezumae|ZMIII994-12|Mexico|BOLD:AAD9211
Mastigodiaptomus montezumae|GBA14343-13|Mexico|BOLD:AAD9211
Mastigodiaptomus montezumae|GBA14344-13|Mexico|BOLD:AAD9211
Mastigodiaptomus montezumae|ZPLMX175-06|Mexico|BOLD:AAD9210
Mastigodiaptomus montezumae|ZPLMX177-06|Mexico|BOLD:AAD9210
Mastigodiaptomus montezumae|ZPII1388-11|Mexico|BOLD:AAD9210
Mastigodiaptomus montezumae|ZPLIV682-11|Mexico|BOLD:AAD9210
Mastigodiaptomus montezumae|ZPLIV683-11|Mexico|BOLD:AAD9210
Mastigodiaptomus montezumae|ZPLIV684-11|Mexico|BOLD:AAD9210
Mastigodiaptomus montezumae|ZMIII890-12|Mexico|BOLD:AAD9210
Mastigodiaptomus montezumae|ZMIII985-12|Mexico|BOLD:AAD9210
Mastigodiaptomus montezumae|ZPLIV480-11|Mexico|BOLD:AAD9210
Mastigodiaptomus montezumae|ZPLIV575-11|Mexico|BOLD:AAD9210
Mastigodiaptomus montezumae|GBA14345-13|Mexico|BOLD:AAD9210
Mastigodiaptomus montezumae|GBA14346-13|Mexico|BOLD:AAD9210
Mastigodiaptomus montezumae|BACZP443-16|Mexico|BOLD:AAD9210
Mastigodiaptomus montezumae|MCM826-17|Mexico|BOLD:AAD9210
Mastigodiaptomus montezumae|MCM822-17|Mexico|BOLD:AAD9210
Mastigodiaptomus montezumae|MCM825-17|Mexico|BOLD:AEE8353
Mastigodiaptomus montezumae|MCM827-17|Mexico|BOLD:AAD9210
Mastigodiaptomus montezumae|GBA14279-13|Mexico|BOLD:AAD9210
Mastigodiaptomus montezumae|MCM824-17|Mexico|BOLD:ADM1317
Mastigodiaptomus montezumae|ZPII829-11|Mexico|BOLD:AAD9210
Mastigodiaptomus montezumae|MCM823-17|Mexico|BOLD:ADM1320
Mastigodiaptomus montezumae|MCM829-17|Mexico|BOLD:AEE6706
Mastigodiaptomus montezumae|MCM831-17|Mexico|BOLD:AEE3563
Leptodiaptomus|JMCRU193-09|Canada|BOLD:AAG9532
Diaptomus|BBCRU021-10|Canada|BOLD:AAG9532
Diaptomus|BBCRU115-10|Canada|BOLD:AAG9532
Diaptomidae|BACZP2286-16|Canada|BOLD:AAG9532
Diaptomidae|BACZP2287-16|Canada|BOLD:AAG9532
Hesperodiaptomus|SACOP037-08|Canada|BOLD:AAG9520
Hesperodiaptomus|SGRZC037-09|Canada|BOLD:AAG9520
Hesperodiaptomus|SGRZC038-09|Canada|BOLD:AAG9520
Hesperodiaptomus|DSMAX352-06|Canada|BOLD:AAG9520
Hesperodiaptomus|DSMAX364-06|Canada|BOLD:AAG9520
Hesperodiaptomus|DSMAX328-06|Canada|BOLD:AAG9520
Hesperodiaptomus|DSMAX340-06|Canada|BOLD:AAG9520
Hesperodiaptomus|DSMAX316-06|Canada|BOLD:AAG9520
Hesperodiaptomus arcticus sp. 1 CHU|OZFWZ332-11|Canada|BOLD:AAG9520
Hesperodiaptomus arcticus sp. 1 CHU|OZFWC550-12|Canada|BOLD:AAG9520
Hesperodiaptomus arcticus sp. 1 CHU|OZFWC552-12|Canada|BOLD:AAG9520
Diaptomidae|GJOA497-21|Canada|BOLD:AER0320
Diaptomidae|GJOAA9326-23|Canada|BOLD:AFD8025
Hesperodiaptomus arcticus sp. 4 CHU|OZFWZ327-11|Canada|BOLD:AAY8705
Hesperodiaptomus arcticus sp. 4 CHU|OZFWZ366-11|Canada|BOLD:AAY8705
Hesperodiaptomus arcticus sp. 4 CHU|OZFWZ331-11|Canada|BOLD:AAY8705
Hesperodiaptomus arcticus sp. 4 CHU|OZFWZ423-11|Canada|BOLD:AAY8705
Hesperodiaptomus arcticus sp. 4 CHU|OZFWC216-11|Canada|BOLD:AAY8705
Hesperodiaptomus arcticus sp. 4 CHU|OZFWC405-11|Canada|BOLD:AAY8705
Hesperodiaptomus arcticus sp. 4 CHU|OZFWC591-12|Canada|BOLD:AAY8705
Hesperodiaptomus|GJOAA9327-23|Canada|BOLD:AER5696
Hesperodiaptomus|TALOA5337-23|Canada|BOLD:AER5696
Hesperodiaptomus|TALOA5338-23|Canada|BOLD:AER5696
Hesperodiaptomus|TALOA5455-23|Canada|BOLD:AER5696
Hesperodiaptomus|TALOA5285-23|Canada|BOLD:AER5696
Hesperodiaptomus|TALOA5278-23|Canada|BOLD:AER5696
Hesperodiaptomus|TALOA5335-23|Canada|BOLD:AER5696
Hesperodiaptomus|TALOA5336-23|Canada|BOLD:AER5696
Hesperodiaptomus|TALOA5283-23|Canada|BOLD:AER5696
Hesperodiaptomus|TALOA5456-23|Canada|BOLD:AER5696
Hesperodiaptomus|TALOA5277-23|Canada|BOLD:AER5696
Hesperodiaptomus|TALOA5457-23|Canada|BOLD:AER5696
Hesperodiaptomus|TALOA5458-23|Canada|BOLD:AER5696
Hesperodiaptomus|GJOAA9310-23|Canada|BOLD:AER5696
Hesperodiaptomus|DNARA737-21|Canada|BOLD:AER5696
Hesperodiaptomus|DNARA910-21|Canada|BOLD:AER5696
Hesperodiaptomus|DNARA3093-21|Canada|BOLD:AER5696
Hesperodiaptomus|DNARA3407-21|Canada|BOLD:AER5696
Hesperodiaptomus|DNARA3122-21|Canada|BOLD:AER5696
Hesperodiaptomus|GJOAA9282-23|Canada|BOLD:AER5696
Hesperodiaptomus|GJOAA9285-23|Canada|BOLD:AER5696
Hesperodiaptomus|DNARA2967-21|Canada|BOLD:AER5696
Hesperodiaptomus|GJOAA9283-23|Canada|BOLD:AER5696
Hesperodiaptomus|GJOAA9284-23|Canada|BOLD:AER5696
Hesperodiaptomus|GJOAA9312-23|Canada|BOLD:AER5696
Hesperodiaptomus|DNARA1497-21|Canada|BOLD:AER5696
Hesperodiaptomus|DNARA2966-21|Canada|BOLD:AER5696
Hesperodiaptomus|DNARA2964-21|Canada|BOLD:AER5696
Hesperodiaptomus|DNARA2963-21|Canada|BOLD:AER5696
Hesperodiaptomus|DNARA2962-21|Canada|BOLD:AER5696
Hesperodiaptomus|DNARA2961-21|Canada|BOLD:AER5696
Hesperodiaptomus|GCHAR1411-19|Canada|BOLD:AER5696
Hesperodiaptomus|DNARC237-19|Canada|BOLD:AER5696
Hesperodiaptomus|GCHAR1149-19|Canada|BOLD:AER5696
Hesperodiaptomus|DNARA2984-21|Canada|BOLD:AER5696
Hesperodiaptomus|GJOAA9313-23|Canada|BOLD:AER5696
Hesperodiaptomus|GJOAA9311-23|Canada|BOLD:AER5696
Hesperodiaptomus|GJOAA9308-23|Canada|BOLD:AER5696
Hesperodiaptomus|GJOAA9307-23|Canada|BOLD:AER5696
Hesperodiaptomus|GJOAA9281-23|Canada|BOLD:AER5696
Hesperodiaptomus|GJOA496-21|Canada|BOLD:AER5696
Hesperodiaptomus|GJOAA9329-23|Canada|BOLD:AER5696
Hesperodiaptomus|GJOAA9330-23|Canada|BOLD:AER5696
Hesperodiaptomus|GJOAA9332-23|Canada|BOLD:AER5696
