# Supplementary material for: DNA barcoding of North American freshwater copepods (Diaptomidae and Cyclopoida): an overview after 20 years with emphasis in the Mexican fauna, the transition between the Nearctic and Neotropics
Source: PeerJ. 2026 Apr 9;14:e20989. doi: 10.7717/peerj.20989 (PMC13070316; doi:10.7717/peerj.20989)
Supplement: Supplemental Information 2 — Includes 2,269 sequences. Each branch shows the Process Id, the country where it was collected, and the BIN assigned for each sequenced specimen. [file peerj-14-20989-s002.pdf]

BOLD TaxonID Tree

Title : Tree Result - Search: Tax(Cyclopoida; Ergasilidae; Cyclopoida_family_incertae_sedis); Geo(Mexico; Canada; United States); Include public records (4136 records returned) (4136 records selected)

Date : 09-Jun-2025

Data Type : Nucleotide

Distance Model : Kimura 2 Parameter

Marker : COI-5P

Colourization : [blue]=Stop Codons [red]=Contamination or misidentification

Label : Process ID

Label : Taxon

Label : Country

Label : Barcode Cluster (BIN)

Filter : length > 500bp only

Filter : exclude records flagged as misidentifications

Filter : exclude records with stop codons

Filter : exclude contaminants

Sequence Count : 2269

Species count : 75

Genus count : 24

Family count : 9

Unidentified : 1120

BIN Count : 235

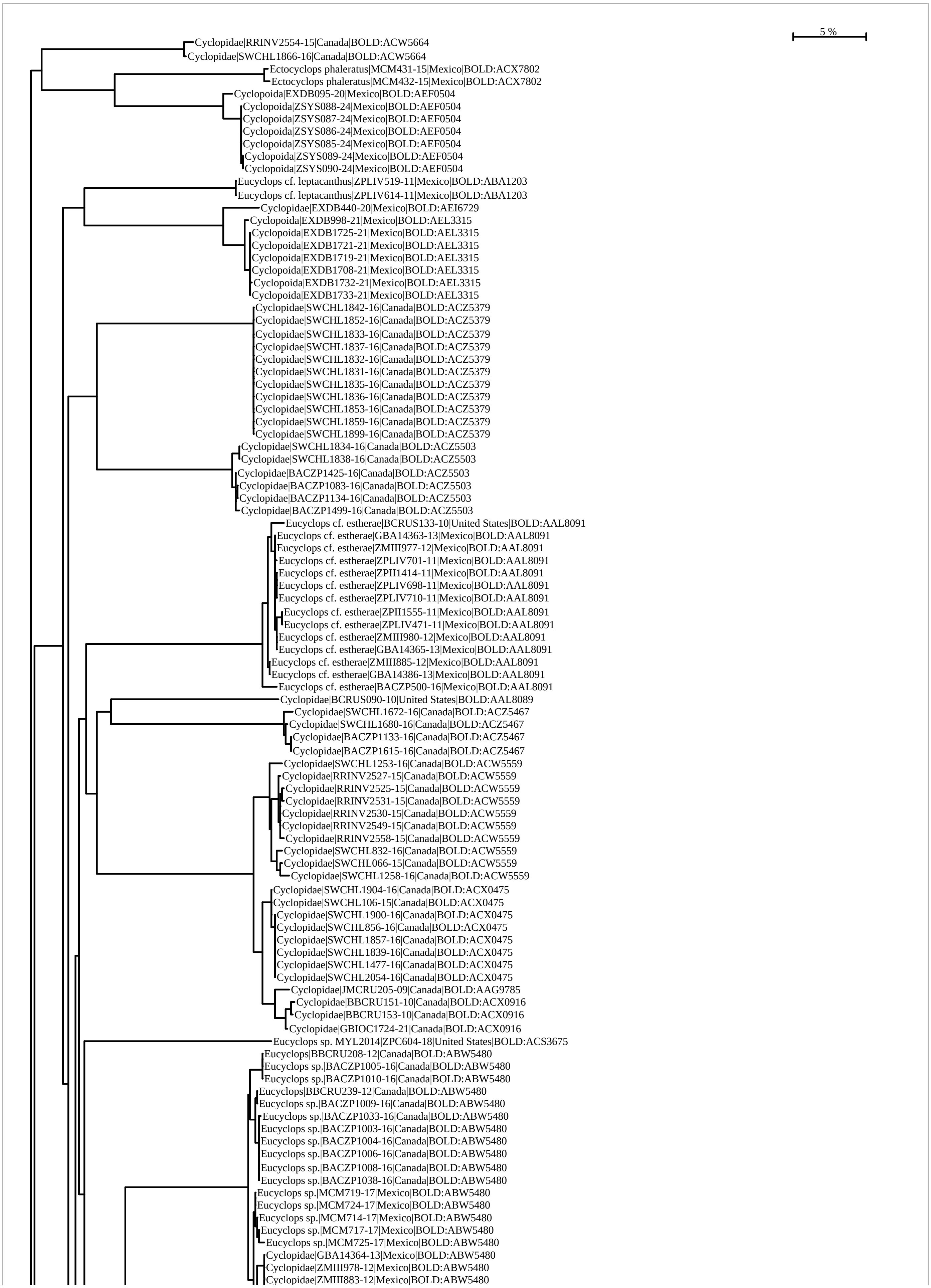
5 %
Cyclopidae|RRINV2554-15|Canada|BOLD:ACW5664
Cyclopidae|SWCHL1866-16|Canada|BOLD:ACW5664
Ectocyclops phaleratus|MCM431-15|Mexico|BOLD:ACX7802
Ectocyclops phaleratus|MCM432-15|Mexico|BOLD:ACX7802
Cyclopoida|EXDB095-20|Mexico|BOLD:AEF0504
Cyclopoida|ZSYS088-24|Mexico|BOLD:AEF0504
Cyclopoida|ZSYS087-24|Mexico|BOLD:AEF0504
Cyclopoida|ZSYS086-24|Mexico|BOLD:AEF0504
Cyclopoida|ZSYS085-24|Mexico|BOLD:AEF0504
Cyclopoida|ZSYS089-24|Mexico|BOLD:AEF0504
Cyclopoida|ZSYS090-24|Mexico|BOLD:AEF0504
Eucyclops cf. leptacanthus|ZPLIV519-11|Mexico|BOLD:ABA1203
Eucyclops cf. leptacanthus|ZPLIV614-11|Mexico|BOLD:ABA1203
Cyclopidae|EXDB440-20|Mexico|BOLD:AEI6729
Cyclopoida|EXDB998-21|Mexico|BOLD:AEL3315
Cyclopoida|EXDB1725-21|Mexico|BOLD:AEL3315
Cyclopoida|EXDB1721-21|Mexico|BOLD:AEL3315
Cyclopoida|EXDB1719-21|Mexico|BOLD:AEL3315
Cyclopoida|EXDB1708-21|Mexico|BOLD:AEL3315
Cyclopoida|EXDB1732-21|Mexico|BOLD:AEL3315
Cyclopoida|EXDB1733-21|Mexico|BOLD:AEL3315
Cyclopidae|SWCHL1842-16|Canada|BOLD:ACZ5379
Cyclopidae|SWCHL1852-16|Canada|BOLD:ACZ5379
Cyclopidae|SWCHL1833-16|Canada|BOLD:ACZ5379
Cyclopidae|SWCHL1837-16|Canada|BOLD:ACZ5379
Cyclopidae|SWCHL1832-16|Canada|BOLD:ACZ5379
Cyclopidae|SWCHL1831-16|Canada|BOLD:ACZ5379
Cyclopidae|SWCHL1835-16|Canada|BOLD:ACZ5379
Cyclopidae|SWCHL1836-16|Canada|BOLD:ACZ5379
Cyclopidae|SWCHL1853-16|Canada|BOLD:ACZ5379
Cyclopidae|SWCHL1859-16|Canada|BOLD:ACZ5379
Cyclopidae|SWCHL1899-16|Canada|BOLD:ACZ5379
Cyclopidae|SWCHL1834-16|Canada|BOLD:ACZ5503
Cyclopidae|SWCHL1838-16|Canada|BOLD:ACZ5503
Cyclopidae|BACZP1425-16|Canada|BOLD:ACZ5503
Cyclopidae|BACZP1083-16|Canada|BOLD:ACZ5503
Cyclopidae|BACZP1134-16|Canada|BOLD:ACZ5503
Cyclopidae|BACZP1499-16|Canada|BOLD:ACZ5503
Eucyclops cf. estherae|BCRUS133-10|United States|BOLD:AAL8091
Eucyclops cf. estherae|GBA14363-13|Mexico|BOLD:AAL8091
Eucyclops cf. estherae|ZMIII977-12|Mexico|BOLD:AAL8091
Eucyclops cf. estherae|ZPLIV701-11|Mexico|BOLD:AAL8091
Eucyclops cf. estherae|ZPII1414-11|Mexico|BOLD:AAL8091
Eucyclops cf. estherae|ZPLIV698-11|Mexico|BOLD:AAL8091
Eucyclops cf. estherae|ZPLIV710-11|Mexico|BOLD:AAL8091
Eucyclops cf. estherae|ZPII1555-11|Mexico|BOLD:AAL8091
Eucyclops cf. estherae|ZPLIV471-11|Mexico|BOLD:AAL8091
Eucyclops cf. estherae|ZMIII980-12|Mexico|BOLD:AAL8091
Eucyclops cf. estherae|GBA14365-13|Mexico|BOLD:AAL8091
Eucyclops cf. estherae|ZMIII885-12|Mexico|BOLD:AAL8091
Eucyclops cf. estherae|GBA14386-13|Mexico|BOLD:AAL8091
Eucyclops cf. estherae|BACZP500-16|Mexico|BOLD:AAL8091
Cyclopidae|BCRUS090-10|United States|BOLD:AAL8089
Cyclopidae|SWCHL1672-16|Canada|BOLD:ACZ5467
Cyclopidae|SWCHL1680-16|Canada|BOLD:ACZ5467
Cyclopidae|BACZP1133-16|Canada|BOLD:ACZ5467
Cyclopidae|BACZP1615-16|Canada|BOLD:ACZ5467
Cyclopidae|SWCHL1253-16|Canada|BOLD:ACW5559
Cyclopidae|RRINV2527-15|Canada|BOLD:ACW5559
Cyclopidae|RRINV2525-15|Canada|BOLD:ACW5559
Cyclopidae|RRINV2531-15|Canada|BOLD:ACW5559
Cyclopidae|RRINV2530-15|Canada|BOLD:ACW5559
Cyclopidae|RRINV2549-15|Canada|BOLD:ACW5559
Cyclopidae|RRINV2558-15|Canada|BOLD:ACW5559
Cyclopidae|SWCHL832-16|Canada|BOLD:ACW5559
Cyclopidae|SWCHL066-15|Canada|BOLD:ACW5559
Cyclopidae|SWCHL1258-16|Canada|BOLD:ACW5559
Cyclopidae|SWCHL1904-16|Canada|BOLD:ACX0475
Cyclopidae|SWCHL106-15|Canada|BOLD:ACX0475
Cyclopidae|SWCHL1900-16|Canada|BOLD:ACX0475
Cyclopidae|SWCHL856-16|Canada|BOLD:ACX0475
Cyclopidae|SWCHL1857-16|Canada|BOLD:ACX0475
Cyclopidae|SWCHL1839-16|Canada|BOLD:ACX0475
Cyclopidae|SWCHL1477-16|Canada|BOLD:ACX0475
Cyclopidae|SWCHL2054-16|Canada|BOLD:ACX0475
Cyclopidae|JMCRU205-09|Canada|BOLD:AAG9785
Cyclopidae|BBCRU151-10|Canada|BOLD:ACX0916
Cyclopidae|BBCRU153-10|Canada|BOLD:ACX0916
Cyclopidae|GBIOC1724-21|Canada|BOLD:ACX0916
Eucyclops sp. MYL2014|ZPC604-18|United States|BOLD:ACS3675
Eucyclops|BBCRU208-12|Canada|BOLD:ABW5480
Eucyclops sp.|BACZP1005-16|Canada|BOLD:ABW5480
Eucyclops sp.|BACZP1010-16|Canada|BOLD:ABW5480
Eucyclops|BBCRU239-12|Canada|BOLD:ABW5480
Eucyclops sp.|BACZP1009-16|Canada|BOLD:ABW5480
Eucyclops sp.|BACZP1033-16|Canada|BOLD:ABW5480
Eucyclops sp.|BACZP1003-16|Canada|BOLD:ABW5480
Eucyclops sp.|BACZP1004-16|Canada|BOLD:ABW5480
Eucyclops sp.|BACZP1006-16|Canada|BOLD:ABW5480
Eucyclops sp.|BACZP1008-16|Canada|BOLD:ABW5480
Eucyclops sp.|BACZP1038-16|Canada|BOLD:ABW5480
Eucyclops sp.|MCM719-17|Mexico|BOLD:ABW5480
Eucyclops sp.|MCM724-17|Mexico|BOLD:ABW5480
Eucyclops sp.|MCM714-17|Mexico|BOLD:ABW5480
Eucyclops sp.|MCM717-17|Mexico|BOLD:ABW5480
Eucyclops sp.|MCM725-17|Mexico|BOLD:ABW5480
Cyclopidae|GBA14364-13|Mexico|BOLD:ABW5480
Cyclopidae|ZMIII978-12|Mexico|BOLD:ABW5480
Cyclopidae|ZMIII883-12|Mexico|BOLD:ABW5480

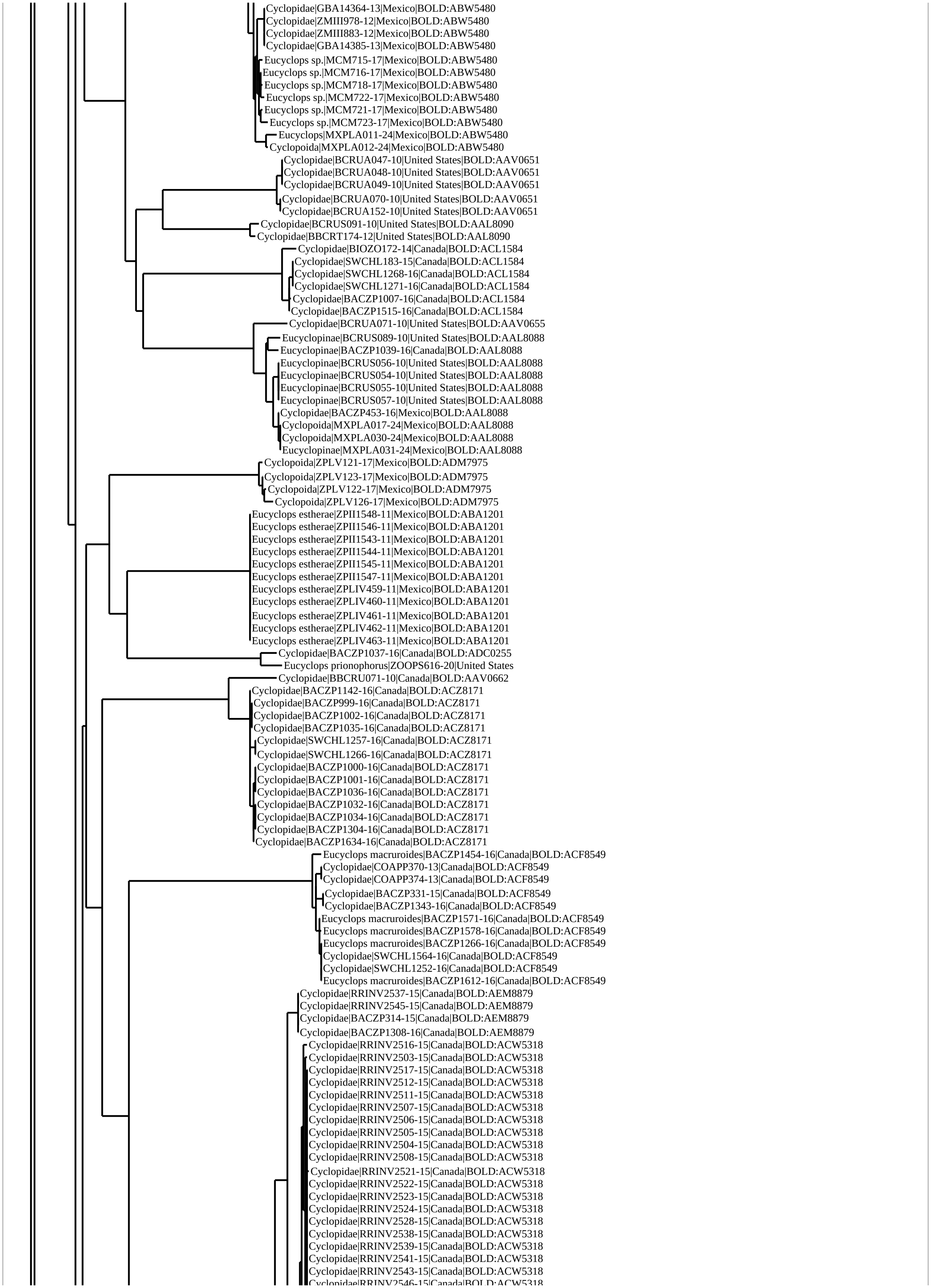
Cyclopidae|GBA14364-13|Mexico|BOLD:ABW5480
Cyclopidae|ZMIII978-12|Mexico|BOLD:ABW5480
Cyclopidae|ZMIII883-12|Mexico|BOLD:ABW5480
Cyclopidae|GBA14385-13|Mexico|BOLD:ABW5480
Eucyclops sp.|MCM715-17|Mexico|BOLD:ABW5480
Eucyclops sp.|MCM716-17|Mexico|BOLD:ABW5480
Eucyclops sp.|MCM718-17|Mexico|BOLD:ABW5480
Eucyclops sp.|MCM722-17|Mexico|BOLD:ABW5480
Eucyclops sp.|MCM721-17|Mexico|BOLD:ABW5480
Eucyclops sp.|MCM723-17|Mexico|BOLD:ABW5480
Eucyclops|MXPLA011-24|Mexico|BOLD:ABW5480
Cyclopoida|MXPLA012-24|Mexico|BOLD:ABW5480
Cyclopidae|BCRUA047-10|United States|BOLD:AAV0651
Cyclopidae|BCRUA048-10|United States|BOLD:AAV0651
Cyclopidae|BCRUA049-10|United States|BOLD:AAV0651
Cyclopidae|BCRUA070-10|United States|BOLD:AAV0651
Cyclopidae|BCRUA152-10|United States|BOLD:AAV0651
Cyclopidae|BCRUS091-10|United States|BOLD:AAL8090
Cyclopidae|BBCRT174-12|United States|BOLD:AAL8090
Cyclopidae|BIOZO172-14|Canada|BOLD:ACL1584
Cyclopidae|SWCHL183-15|Canada|BOLD:ACL1584
Cyclopidae|SWCHL1268-16|Canada|BOLD:ACL1584
Cyclopidae|SWCHL1271-16|Canada|BOLD:ACL1584
Cyclopidae|BACZP1007-16|Canada|BOLD:ACL1584
Cyclopidae|BACZP1515-16|Canada|BOLD:ACL1584
Cyclopidae|BCRUA071-10|United States|BOLD:AAV0655
Eucyclopinae|BCRUS089-10|United States|BOLD:AAL8088
Eucyclopinae|BACZP1039-16|Canada|BOLD:AAL8088
Eucyclopinae|BCRUS056-10|United States|BOLD:AAL8088
Eucyclopinae|BCRUS054-10|United States|BOLD:AAL8088
Eucyclopinae|BCRUS055-10|United States|BOLD:AAL8088
Eucyclopinae|BCRUS057-10|United States|BOLD:AAL8088
Cyclopidae|BACZP453-16|Mexico|BOLD:AAL8088
Cyclopoida|MXPLA017-24|Mexico|BOLD:AAL8088
Cyclopoida|MXPLA030-24|Mexico|BOLD:AAL8088
Eucyclopinae|MXPLA031-24|Mexico|BOLD:AAL8088
Cyclopoida|ZPLV121-17|Mexico|BOLD:ADM7975
Cyclopoida|ZPLV123-17|Mexico|BOLD:ADM7975
Cyclopoida|ZPLV122-17|Mexico|BOLD:ADM7975
Cyclopoida|ZPLV126-17|Mexico|BOLD:ADM7975
Eucyclops estherae|ZPII1548-11|Mexico|BOLD:ABA1201
Eucyclops estherae|ZPII1546-11|Mexico|BOLD:ABA1201
Eucyclops estherae|ZPII1543-11|Mexico|BOLD:ABA1201
Eucyclops estherae|ZPII1544-11|Mexico|BOLD:ABA1201
Eucyclops estherae|ZPII1545-11|Mexico|BOLD:ABA1201
Eucyclops estherae|ZPII1547-11|Mexico|BOLD:ABA1201
Eucyclops estherae|ZPLIV459-11|Mexico|BOLD:ABA1201
Eucyclops estherae|ZPLIV460-11|Mexico|BOLD:ABA1201
Eucyclops estherae|ZPLIV461-11|Mexico|BOLD:ABA1201
Eucyclops estherae|ZPLIV462-11|Mexico|BOLD:ABA1201
Eucyclops estherae|ZPLIV463-11|Mexico|BOLD:ABA1201
Cyclopidae|BACZP1037-16|Canada|BOLD:ADC0255
Eucyclops prionophorus|ZOOPS616-20|United States
Cyclopidae|BBCRU071-10|Canada|BOLD:AAV0662
Cyclopidae|BACZP1142-16|Canada|BOLD:ACZ8171
Cyclopidae|BACZP999-16|Canada|BOLD:ACZ8171
Cyclopidae|BACZP1002-16|Canada|BOLD:ACZ8171
Cyclopidae|BACZP1035-16|Canada|BOLD:ACZ8171
Cyclopidae|SWCHL1257-16|Canada|BOLD:ACZ8171
Cyclopidae|SWCHL1266-16|Canada|BOLD:ACZ8171
Cyclopidae|BACZP1000-16|Canada|BOLD:ACZ8171
Cyclopidae|BACZP1001-16|Canada|BOLD:ACZ8171
Cyclopidae|BACZP1036-16|Canada|BOLD:ACZ8171
Cyclopidae|BACZP1032-16|Canada|BOLD:ACZ8171
Cyclopidae|BACZP1034-16|Canada|BOLD:ACZ8171
Cyclopidae|BACZP1304-16|Canada|BOLD:ACZ8171
Cyclopidae|BACZP1634-16|Canada|BOLD:ACZ8171
Eucyclops macruroides|BACZP1454-16|Canada|BOLD:ACF8549
Cyclopidae|COAPP370-13|Canada|BOLD:ACF8549
Cyclopidae|COAPP374-13|Canada|BOLD:ACF8549
Cyclopidae|BACZP331-15|Canada|BOLD:ACF8549
Cyclopidae|BACZP1343-16|Canada|BOLD:ACF8549
Eucyclops macruroides|BACZP1571-16|Canada|BOLD:ACF8549
Eucyclops macruroides|BACZP1578-16|Canada|BOLD:ACF8549
Eucyclops macruroides|BACZP1266-16|Canada|BOLD:ACF8549
Cyclopidae|SWCHL1564-16|Canada|BOLD:ACF8549
Cyclopidae|SWCHL1252-16|Canada|BOLD:ACF8549
Eucyclops macruroides|BACZP1612-16|Canada|BOLD:ACF8549
Cyclopidae|RRINV2537-15|Canada|BOLD:AEM8879
Cyclopidae|RRINV2545-15|Canada|BOLD:AEM8879
Cyclopidae|BACZP314-15|Canada|BOLD:AEM8879
Cyclopidae|BACZP1308-16|Canada|BOLD:AEM8879
Cyclopidae|RRINV2516-15|Canada|BOLD:ACW5318
Cyclopidae|RRINV2503-15|Canada|BOLD:ACW5318
Cyclopidae|RRINV2517-15|Canada|BOLD:ACW5318
Cyclopidae|RRINV2512-15|Canada|BOLD:ACW5318
Cyclopidae|RRINV2511-15|Canada|BOLD:ACW5318
Cyclopidae|RRINV2507-15|Canada|BOLD:ACW5318
Cyclopidae|RRINV2506-15|Canada|BOLD:ACW5318
Cyclopidae|RRINV2505-15|Canada|BOLD:ACW5318
Cyclopidae|RRINV2504-15|Canada|BOLD:ACW5318
Cyclopidae|RRINV2508-15|Canada|BOLD:ACW5318
Cyclopidae|RRINV2521-15|Canada|BOLD:ACW5318
Cyclopidae|RRINV2522-15|Canada|BOLD:ACW5318
Cyclopidae|RRINV2523-15|Canada|BOLD:ACW5318
Cyclopidae|RRINV2524-15|Canada|BOLD:ACW5318
Cyclopidae|RRINV2528-15|Canada|BOLD:ACW5318
Cyclopidae|RRINV2538-15|Canada|BOLD:ACW5318
Cyclopidae|RRINV2539-15|Canada|BOLD:ACW5318
Cyclopidae|RRINV2541-15|Canada|BOLD:ACW5318
Cyclopidae|RRINV2543-15|Canada|BOLD:ACW5318
Cyclopidae|RRINV2546-15|Canada|BOLD:ACW5318

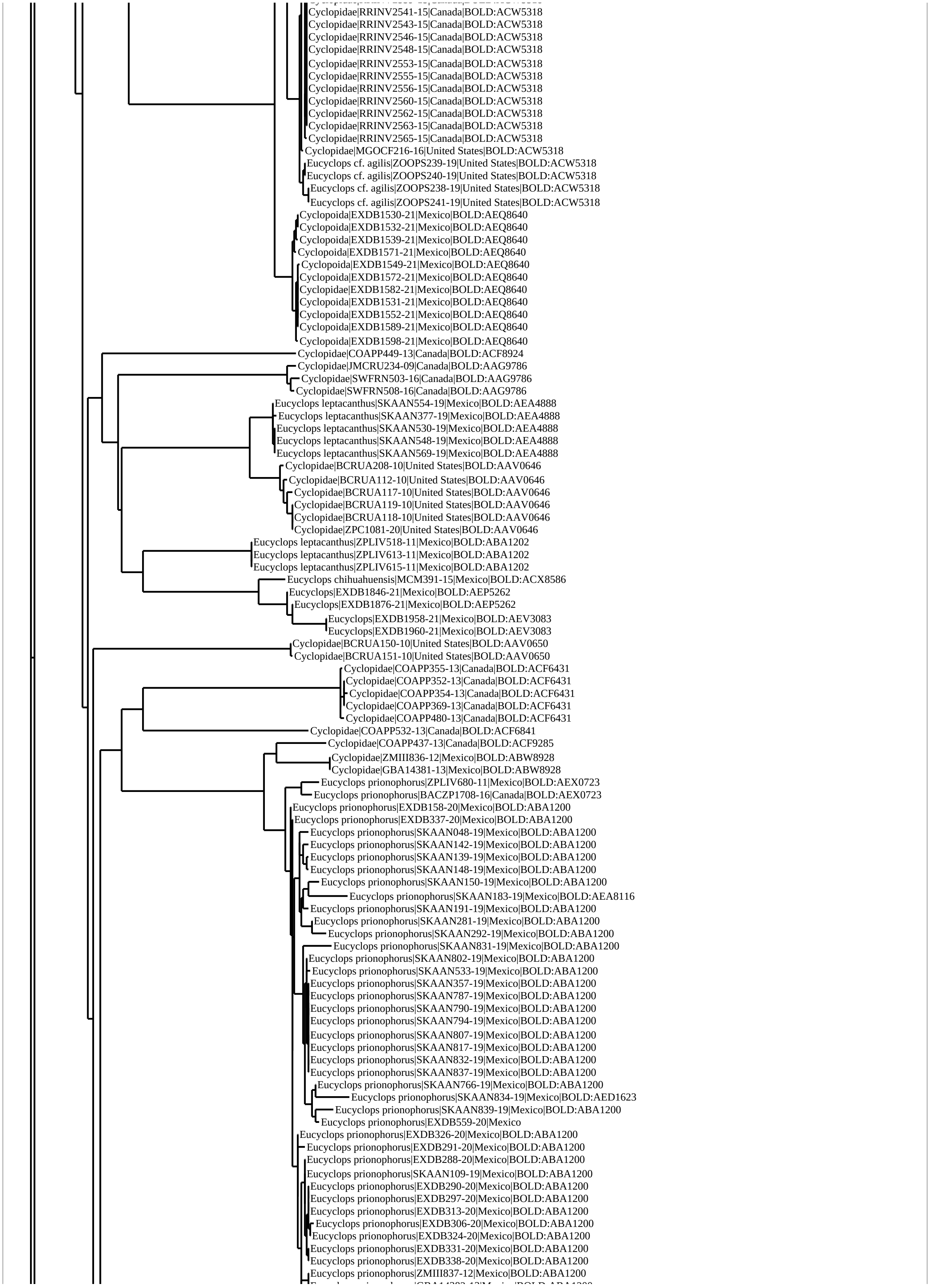
Cyclopidae|RRINV2541-15|Canada|BOLD:ACW5318
Cyclopidae|RRINV2543-15|Canada|BOLD:ACW5318
Cyclopidae|RRINV2546-15|Canada|BOLD:ACW5318
Cyclopidae|RRINV2548-15|Canada|BOLD:ACW5318
Cyclopidae|RRINV2553-15|Canada|BOLD:ACW5318
Cyclopidae|RRINV2555-15|Canada|BOLD:ACW5318
Cyclopidae|RRINV2556-15|Canada|BOLD:ACW5318
Cyclopidae|RRINV2560-15|Canada|BOLD:ACW5318
Cyclopidae|RRINV2562-15|Canada|BOLD:ACW5318
Cyclopidae|RRINV2563-15|Canada|BOLD:ACW5318
Cyclopidae|RRINV2565-15|Canada|BOLD:ACW5318
Cyclopidae|MGOCF216-16|United States|BOLD:ACW5318
Eucyclops cf. agilis|ZOOPS239-19|United States|BOLD:ACW5318
Eucyclops cf. agilis|ZOOPS240-19|United States|BOLD:ACW5318
Eucyclops cf. agilis|ZOOPS238-19|United States|BOLD:ACW5318
Eucyclops cf. agilis|ZOOPS241-19|United States|BOLD:ACW5318
Cyclopoida|EXDB1530-21|Mexico|BOLD:AEQ8640
Cyclopoida|EXDB1532-21|Mexico|BOLD:AEQ8640
Cyclopoida|EXDB1539-21|Mexico|BOLD:AEQ8640
Cyclopoida|EXDB1571-21|Mexico|BOLD:AEQ8640
Cyclopoida|EXDB1549-21|Mexico|BOLD:AEQ8640
Cyclopoida|EXDB1572-21|Mexico|BOLD:AEQ8640
Cyclopidae|EXDB1582-21|Mexico|BOLD:AEQ8640
Cyclopoida|EXDB1531-21|Mexico|BOLD:AEQ8640
Cyclopoida|EXDB1552-21|Mexico|BOLD:AEQ8640
Cyclopoida|EXDB1589-21|Mexico|BOLD:AEQ8640
Cyclopoida|EXDB1598-21|Mexico|BOLD:AEQ8640
Cyclopidae|COAPP449-13|Canada|BOLD:ACF8924
Cyclopidae|JMCRU234-09|Canada|BOLD:AAG9786
Cyclopidae|SWFRN503-16|Canada|BOLD:AAG9786
Cyclopidae|SWFRN508-16|Canada|BOLD:AAG9786
Eucyclops leptacanthus|SKAAN554-19|Mexico|BOLD:AEA4888
Eucyclops leptacanthus|SKAAN377-19|Mexico|BOLD:AEA4888
Eucyclops leptacanthus|SKAAN530-19|Mexico|BOLD:AEA4888
Eucyclops leptacanthus|SKAAN548-19|Mexico|BOLD:AEA4888
Eucyclops leptacanthus|SKAAN569-19|Mexico|BOLD:AEA4888
Cyclopidae|BCRUA208-10|United States|BOLD:AAV0646
Cyclopidae|BCRUA112-10|United States|BOLD:AAV0646
Cyclopidae|BCRUA117-10|United States|BOLD:AAV0646
Cyclopidae|BCRUA119-10|United States|BOLD:AAV0646
Cyclopidae|BCRUA118-10|United States|BOLD:AAV0646
Cyclopidae|ZPC1081-20|United States|BOLD:AAV0646
Eucyclops leptacanthus|ZPLIV518-11|Mexico|BOLD:ABA1202
Eucyclops leptacanthus|ZPLIV613-11|Mexico|BOLD:ABA1202
Eucyclops leptacanthus|ZPLIV615-11|Mexico|BOLD:ABA1202
Eucyclops chihuahuensis|MCM391-15|Mexico|BOLD:ACX8586
Eucyclops|EXDB1846-21|Mexico|BOLD:AEP5262
Eucyclops|EXDB1876-21|Mexico|BOLD:AEP5262
Eucyclops|EXDB1958-21|Mexico|BOLD:AEV3083
Eucyclops|EXDB1960-21|Mexico|BOLD:AEV3083
Cyclopidae|BCRUA150-10|United States|BOLD:AAV0650
Cyclopidae|BCRUA151-10|United States|BOLD:AAV0650
Cyclopidae|COAPP355-13|Canada|BOLD:ACF6431
Cyclopidae|COAPP352-13|Canada|BOLD:ACF6431
Cyclopidae|COAPP354-13|Canada|BOLD:ACF6431
Cyclopidae|COAPP369-13|Canada|BOLD:ACF6431
Cyclopidae|COAPP480-13|Canada|BOLD:ACF6431
Cyclopidae|COAPP532-13|Canada|BOLD:ACF6841
Cyclopidae|COAPP437-13|Canada|BOLD:ACF9285
Cyclopidae|ZMIII836-12|Mexico|BOLD:ABW8928
Cyclopidae|GBA14381-13|Mexico|BOLD:ABW8928
Eucyclops prionophorus|ZPLIV680-11|Mexico|BOLD:AEX0723
Eucyclops prionophorus|BACZP1708-16|Canada|BOLD:AEX0723
Eucyclops prionophorus|EXDB158-20|Mexico|BOLD:ABA1200
Eucyclops prionophorus|EXDB337-20|Mexico|BOLD:ABA1200
Eucyclops prionophorus|SKAAN048-19|Mexico|BOLD:ABA1200
Eucyclops prionophorus|SKAAN142-19|Mexico|BOLD:ABA1200
Eucyclops prionophorus|SKAAN139-19|Mexico|BOLD:ABA1200
Eucyclops prionophorus|SKAAN148-19|Mexico|BOLD:ABA1200
Eucyclops prionophorus|SKAAN150-19|Mexico|BOLD:ABA1200
Eucyclops prionophorus|SKAAN183-19|Mexico|BOLD:AEA8116
Eucyclops prionophorus|SKAAN191-19|Mexico|BOLD:ABA1200
Eucyclops prionophorus|SKAAN281-19|Mexico|BOLD:ABA1200
Eucyclops prionophorus|SKAAN292-19|Mexico|BOLD:ABA1200
Eucyclops prionophorus|SKAAN831-19|Mexico|BOLD:ABA1200
Eucyclops prionophorus|SKAAN802-19|Mexico|BOLD:ABA1200
Eucyclops prionophorus|SKAAN533-19|Mexico|BOLD:ABA1200
Eucyclops prionophorus|SKAAN357-19|Mexico|BOLD:ABA1200
Eucyclops prionophorus|SKAAN787-19|Mexico|BOLD:ABA1200
Eucyclops prionophorus|SKAAN790-19|Mexico|BOLD:ABA1200
Eucyclops prionophorus|SKAAN794-19|Mexico|BOLD:ABA1200
Eucyclops prionophorus|SKAAN807-19|Mexico|BOLD:ABA1200
Eucyclops prionophorus|SKAAN817-19|Mexico|BOLD:ABA1200
Eucyclops prionophorus|SKAAN832-19|Mexico|BOLD:ABA1200
Eucyclops prionophorus|SKAAN837-19|Mexico|BOLD:ABA1200
Eucyclops prionophorus|SKAAN766-19|Mexico|BOLD:ABA1200
Eucyclops prionophorus|SKAAN834-19|Mexico|BOLD:AED1623
Eucyclops prionophorus|SKAAN839-19|Mexico|BOLD:ABA1200
Eucyclops prionophorus|EXDB559-20|Mexico
Eucyclops prionophorus|EXDB326-20|Mexico|BOLD:ABA1200
Eucyclops prionophorus|EXDB291-20|Mexico|BOLD:ABA1200
Eucyclops prionophorus|EXDB288-20|Mexico|BOLD:ABA1200
Eucyclops prionophorus|SKAAN109-19|Mexico|BOLD:ABA1200
Eucyclops prionophorus|EXDB290-20|Mexico|BOLD:ABA1200
Eucyclops prionophorus|EXDB297-20|Mexico|BOLD:ABA1200
Eucyclops prionophorus|EXDB313-20|Mexico|BOLD:ABA1200
Eucyclops prionophorus|EXDB306-20|Mexico|BOLD:ABA1200
Eucyclops prionophorus|EXDB324-20|Mexico|BOLD:ABA1200
Eucyclops prionophorus|EXDB331-20|Mexico|BOLD:ABA1200
Eucyclops prionophorus|EXDB338-20|Mexico|BOLD:ABA1200
Eucyclops prionophorus|ZMIII837-12|Mexico|BOLD:ABA1200

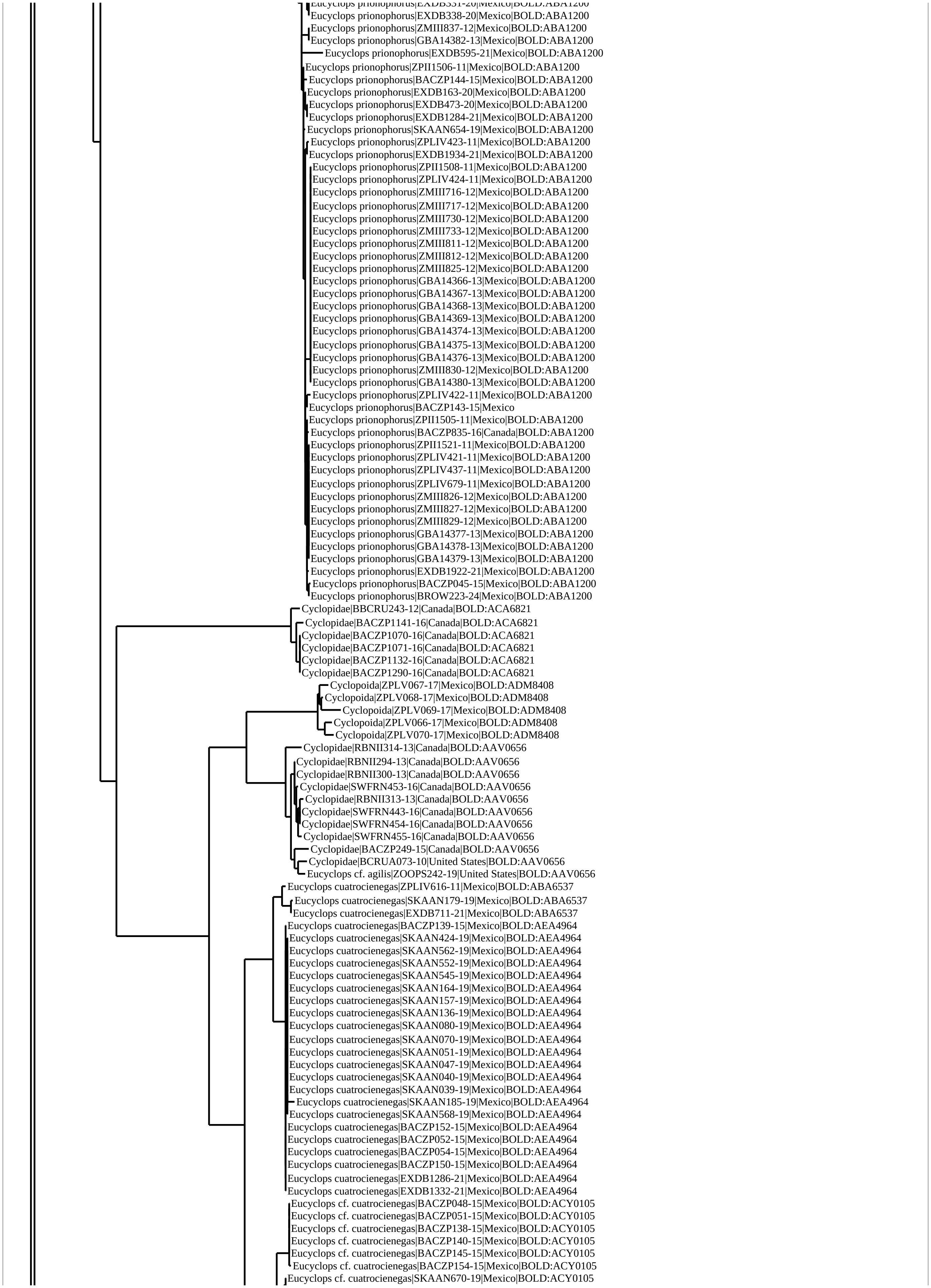
Eucyclops prionophorus|EXDB331-20|Mexico|BOLD:ABA1200
Eucyclops prionophorus|EXDB338-20|Mexico|BOLD:ABA1200
Eucyclops prionophorus|ZMIII837-12|Mexico|BOLD:ABA1200
Eucyclops prionophorus|GBA14382-13|Mexico|BOLD:ABA1200
Eucyclops prionophorus|EXDB595-21|Mexico|BOLD:ABA1200
Eucyclops prionophorus|ZPII1506-11|Mexico|BOLD:ABA1200
Eucyclops prionophorus|BACZP144-15|Mexico|BOLD:ABA1200
Eucyclops prionophorus|EXDB163-20|Mexico|BOLD:ABA1200
Eucyclops prionophorus|EXDB473-20|Mexico|BOLD:ABA1200
Eucyclops prionophorus|EXDB1284-21|Mexico|BOLD:ABA1200
Eucyclops prionophorus|SKAAN654-19|Mexico|BOLD:ABA1200
Eucyclops prionophorus|ZPLIV423-11|Mexico|BOLD:ABA1200
Eucyclops prionophorus|EXDB1934-21|Mexico|BOLD:ABA1200
Eucyclops prionophorus|ZPII1508-11|Mexico|BOLD:ABA1200
Eucyclops prionophorus|ZPLIV424-11|Mexico|BOLD:ABA1200
Eucyclops prionophorus|ZMIII716-12|Mexico|BOLD:ABA1200
Eucyclops prionophorus|ZMIII717-12|Mexico|BOLD:ABA1200
Eucyclops prionophorus|ZMIII730-12|Mexico|BOLD:ABA1200
Eucyclops prionophorus|ZMIII733-12|Mexico|BOLD:ABA1200
Eucyclops prionophorus|ZMIII811-12|Mexico|BOLD:ABA1200
Eucyclops prionophorus|ZMIII812-12|Mexico|BOLD:ABA1200
Eucyclops prionophorus|ZMIII825-12|Mexico|BOLD:ABA1200
Eucyclops prionophorus|GBA14366-13|Mexico|BOLD:ABA1200
Eucyclops prionophorus|GBA14367-13|Mexico|BOLD:ABA1200
Eucyclops prionophorus|GBA14368-13|Mexico|BOLD:ABA1200
Eucyclops prionophorus|GBA14369-13|Mexico|BOLD:ABA1200
Eucyclops prionophorus|GBA14374-13|Mexico|BOLD:ABA1200
Eucyclops prionophorus|GBA14375-13|Mexico|BOLD:ABA1200
Eucyclops prionophorus|GBA14376-13|Mexico|BOLD:ABA1200
Eucyclops prionophorus|ZMIII830-12|Mexico|BOLD:ABA1200
Eucyclops prionophorus|GBA14380-13|Mexico|BOLD:ABA1200
Eucyclops prionophorus|ZPLIV422-11|Mexico|BOLD:ABA1200
Eucyclops prionophorus|BACZP143-15|Mexico
Eucyclops prionophorus|ZPII1505-11|Mexico|BOLD:ABA1200
Eucyclops prionophorus|BACZP835-16|Canada|BOLD:ABA1200
Eucyclops prionophorus|ZPII1521-11|Mexico|BOLD:ABA1200
Eucyclops prionophorus|ZPLIV421-11|Mexico|BOLD:ABA1200
Eucyclops prionophorus|ZPLIV437-11|Mexico|BOLD:ABA1200
Eucyclops prionophorus|ZPLIV679-11|Mexico|BOLD:ABA1200
Eucyclops prionophorus|ZMIII826-12|Mexico|BOLD:ABA1200
Eucyclops prionophorus|ZMIII827-12|Mexico|BOLD:ABA1200
Eucyclops prionophorus|ZMIII829-12|Mexico|BOLD:ABA1200
Eucyclops prionophorus|GBA14377-13|Mexico|BOLD:ABA1200
Eucyclops prionophorus|GBA14378-13|Mexico|BOLD:ABA1200
Eucyclops prionophorus|GBA14379-13|Mexico|BOLD:ABA1200
Eucyclops prionophorus|EXDB1922-21|Mexico|BOLD:ABA1200
Eucyclops prionophorus|BACZP045-15|Mexico|BOLD:ABA1200
Eucyclops prionophorus|BROW223-24|Mexico|BOLD:ABA1200
Cyclopidae|BBCRU243-12|Canada|BOLD:ACA6821
Cyclopidae|BACZP1141-16|Canada|BOLD:ACA6821
Cyclopidae|BACZP1070-16|Canada|BOLD:ACA6821
Cyclopidae|BACZP1071-16|Canada|BOLD:ACA6821
Cyclopidae|BACZP1132-16|Canada|BOLD:ACA6821
Cyclopidae|BACZP1290-16|Canada|BOLD:ACA6821
Cyclopoida|ZPLV067-17|Mexico|BOLD:ADM8408
Cyclopoida|ZPLV068-17|Mexico|BOLD:ADM8408
Cyclopoida|ZPLV069-17|Mexico|BOLD:ADM8408
Cyclopoida|ZPLV066-17|Mexico|BOLD:ADM8408
Cyclopoida|ZPLV070-17|Mexico|BOLD:ADM8408
Cyclopidae|RBNII314-13|Canada|BOLD:AAV0656
Cyclopidae|RBNII294-13|Canada|BOLD:AAV0656
Cyclopidae|RBNII300-13|Canada|BOLD:AAV0656
Cyclopidae|SWFRN453-16|Canada|BOLD:AAV0656
Cyclopidae|RBNII313-13|Canada|BOLD:AAV0656
Cyclopidae|SWFRN443-16|Canada|BOLD:AAV0656
Cyclopidae|SWFRN454-16|Canada|BOLD:AAV0656
Cyclopidae|SWFRN455-16|Canada|BOLD:AAV0656
Cyclopidae|BACZP249-15|Canada|BOLD:AAV0656
Cyclopidae|BCRUA073-10|United States|BOLD:AAV0656
Eucyclops cf. agilis|ZOOPS242-19|United States|BOLD:AAV0656
Eucyclops cuatrocienegas|ZPLIV616-11|Mexico|BOLD:ABA6537
Eucyclops cuatrocienegas|SKAAN179-19|Mexico|BOLD:ABA6537
Eucyclops cuatrocienegas|EXDB711-21|Mexico|BOLD:ABA6537
Eucyclops cuatrocienegas|BACZP139-15|Mexico|BOLD:AEA4964
Eucyclops cuatrocienegas|SKAAN424-19|Mexico|BOLD:AEA4964
Eucyclops cuatrocienegas|SKAAN562-19|Mexico|BOLD:AEA4964
Eucyclops cuatrocienegas|SKAAN552-19|Mexico|BOLD:AEA4964
Eucyclops cuatrocienegas|SKAAN545-19|Mexico|BOLD:AEA4964
Eucyclops cuatrocienegas|SKAAN164-19|Mexico|BOLD:AEA4964
Eucyclops cuatrocienegas|SKAAN157-19|Mexico|BOLD:AEA4964
Eucyclops cuatrocienegas|SKAAN136-19|Mexico|BOLD:AEA4964
Eucyclops cuatrocienegas|SKAAN080-19|Mexico|BOLD:AEA4964
Eucyclops cuatrocienegas|SKAAN070-19|Mexico|BOLD:AEA4964
Eucyclops cuatrocienegas|SKAAN051-19|Mexico|BOLD:AEA4964
Eucyclops cuatrocienegas|SKAAN047-19|Mexico|BOLD:AEA4964
Eucyclops cuatrocienegas|SKAAN040-19|Mexico|BOLD:AEA4964
Eucyclops cuatrocienegas|SKAAN039-19|Mexico|BOLD:AEA4964
Eucyclops cuatrocienegas|SKAAN185-19|Mexico|BOLD:AEA4964
Eucyclops cuatrocienegas|SKAAN568-19|Mexico|BOLD:AEA4964
Eucyclops cuatrocienegas|BACZP152-15|Mexico|BOLD:AEA4964
Eucyclops cuatrocienegas|BACZP052-15|Mexico|BOLD:AEA4964
Eucyclops cuatrocienegas|BACZP054-15|Mexico|BOLD:AEA4964
Eucyclops cuatrocienegas|BACZP150-15|Mexico|BOLD:AEA4964
Eucyclops cuatrocienegas|EXDB1286-21|Mexico|BOLD:AEA4964
Eucyclops cuatrocienegas|EXDB1332-21|Mexico|BOLD:AEA4964
Eucyclops cf. cuatrocienegas|BACZP048-15|Mexico|BOLD:ACY0105
Eucyclops cf. cuatrocienegas|BACZP051-15|Mexico|BOLD:ACY0105
Eucyclops cf. cuatrocienegas|BACZP138-15|Mexico|BOLD:ACY0105
Eucyclops cf. cuatrocienegas|BACZP140-15|Mexico|BOLD:ACY0105
Eucyclops cf. cuatrocienegas|BACZP145-15|Mexico|BOLD:ACY0105
Eucyclops cf. cuatrocienegas|BACZP154-15|Mexico|BOLD:ACY0105
Eucyclops cf. cuatrocienegas|SKAAN670-19|Mexico|BOLD:ACY0105

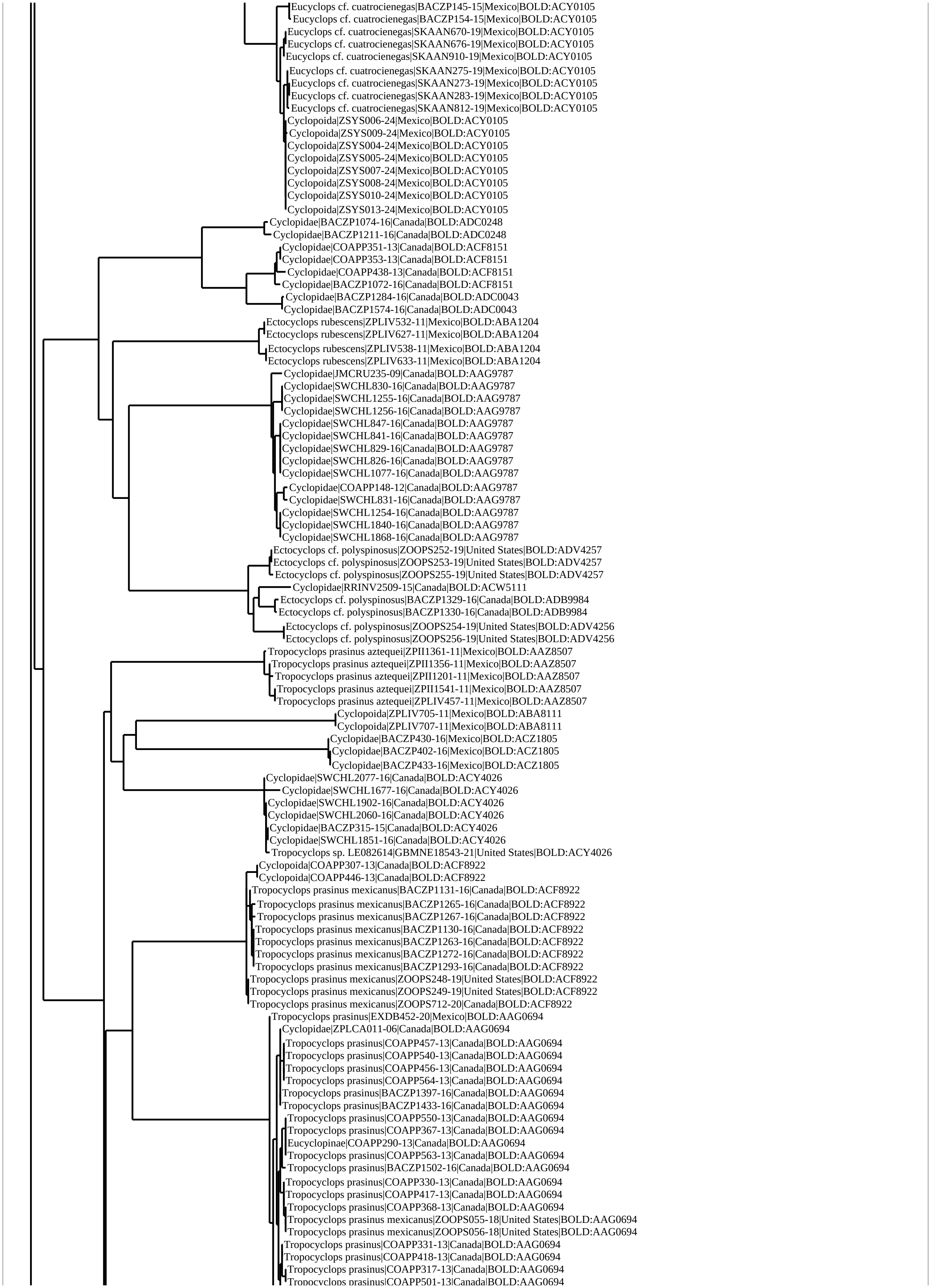
Eucyclops cf. cuatrocienegas|BACZP145-15|Mexico|BOLD:ACY0105
Eucyclops cf. cuatrocienegas|BACZP154-15|Mexico|BOLD:ACY0105
Eucyclops cf. cuatrocienegas|SKAAN670-19|Mexico|BOLD:ACY0105
Eucyclops cf. cuatrocienegas|SKAAN676-19|Mexico|BOLD:ACY0105
Eucyclops cf. cuatrocienegas|SKAAN910-19|Mexico|BOLD:ACY0105
Eucyclops cf. cuatrocienegas|SKAAN275-19|Mexico|BOLD:ACY0105
Eucyclops cf. cuatrocienegas|SKAAN273-19|Mexico|BOLD:ACY0105
Eucyclops cf. cuatrocienegas|SKAAN283-19|Mexico|BOLD:ACY0105
Eucyclops cf. cuatrocienegas|SKAAN812-19|Mexico|BOLD:ACY0105
Cyclopoida|ZSYS006-24|Mexico|BOLD:ACY0105
Cyclopoida|ZSYS009-24|Mexico|BOLD:ACY0105
Cyclopoida|ZSYS004-24|Mexico|BOLD:ACY0105
Cyclopoida|ZSYS005-24|Mexico|BOLD:ACY0105
Cyclopoida|ZSYS007-24|Mexico|BOLD:ACY0105
Cyclopoida|ZSYS008-24|Mexico|BOLD:ACY0105
Cyclopoida|ZSYS010-24|Mexico|BOLD:ACY0105
Cyclopoida|ZSYS013-24|Mexico|BOLD:ACY0105
Cyclopidae|BACZP1074-16|Canada|BOLD:ADC0248
Cyclopidae|BACZP1211-16|Canada|BOLD:ADC0248
Cyclopidae|COAPP351-13|Canada|BOLD:ACF8151
Cyclopidae|COAPP353-13|Canada|BOLD:ACF8151
Cyclopidae|COAPP438-13|Canada|BOLD:ACF8151
Cyclopidae|BACZP1072-16|Canada|BOLD:ACF8151
Cyclopidae|BACZP1284-16|Canada|BOLD:ADC0043
Cyclopidae|BACZP1574-16|Canada|BOLD:ADC0043
Ectocyclops rubescens|ZPLIV532-11|Mexico|BOLD:ABA1204
Ectocyclops rubescens|ZPLIV627-11|Mexico|BOLD:ABA1204
Ectocyclops rubescens|ZPLIV538-11|Mexico|BOLD:ABA1204
Ectocyclops rubescens|ZPLIV633-11|Mexico|BOLD:ABA1204
Cyclopidae|JMCRU235-09|Canada|BOLD:AAG9787
Cyclopidae|SWCHL830-16|Canada|BOLD:AAG9787
Cyclopidae|SWCHL1255-16|Canada|BOLD:AAG9787
Cyclopidae|SWCHL1256-16|Canada|BOLD:AAG9787
Cyclopidae|SWCHL847-16|Canada|BOLD:AAG9787
Cyclopidae|SWCHL841-16|Canada|BOLD:AAG9787
Cyclopidae|SWCHL829-16|Canada|BOLD:AAG9787
Cyclopidae|SWCHL826-16|Canada|BOLD:AAG9787
Cyclopidae|SWCHL1077-16|Canada|BOLD:AAG9787
Cyclopidae|COAPP148-12|Canada|BOLD:AAG9787
Cyclopidae|SWCHL831-16|Canada|BOLD:AAG9787
Cyclopidae|SWCHL1254-16|Canada|BOLD:AAG9787
Cyclopidae|SWCHL1840-16|Canada|BOLD:AAG9787
Cyclopidae|SWCHL1868-16|Canada|BOLD:AAG9787
Ectocyclops cf. polyspinosus|ZOOPS252-19|United States|BOLD:ADV4257
Ectocyclops cf. polyspinosus|ZOOPS253-19|United States|BOLD:ADV4257
Ectocyclops cf. polyspinosus|ZOOPS255-19|United States|BOLD:ADV4257
Cyclopidae|RRINV2509-15|Canada|BOLD:ACW5111
Ectocyclops cf. polyspinosus|BACZP1329-16|Canada|BOLD:ADB9984
Ectocyclops cf. polyspinosus|BACZP1330-16|Canada|BOLD:ADB9984
Ectocyclops cf. polyspinosus|ZOOPS254-19|United States|BOLD:ADV4256
Ectocyclops cf. polyspinosus|ZOOPS256-19|United States|BOLD:ADV4256
Tropocyclops prasinus aztequei|ZPII1361-11|Mexico|BOLD:AAZ8507
Tropocyclops prasinus aztequei|ZPII1356-11|Mexico|BOLD:AAZ8507
Tropocyclops prasinus aztequei|ZPII1201-11|Mexico|BOLD:AAZ8507
Tropocyclops prasinus aztequei|ZPII1541-11|Mexico|BOLD:AAZ8507
Tropocyclops prasinus aztequei|ZPLIV457-11|Mexico|BOLD:AAZ8507
Cyclopoida|ZPLIV705-11|Mexico|BOLD:ABA8111
Cyclopoida|ZPLIV707-11|Mexico|BOLD:ABA8111
Cyclopidae|BACZP430-16|Mexico|BOLD:ACZ1805
Cyclopidae|BACZP402-16|Mexico|BOLD:ACZ1805
Cyclopidae|BACZP433-16|Mexico|BOLD:ACZ1805
Cyclopidae|SWCHL2077-16|Canada|BOLD:ACY4026
Cyclopidae|SWCHL1677-16|Canada|BOLD:ACY4026
Cyclopidae|SWCHL1902-16|Canada|BOLD:ACY4026
Cyclopidae|SWCHL2060-16|Canada|BOLD:ACY4026
Cyclopidae|BACZP315-15|Canada|BOLD:ACY4026
Cyclopidae|SWCHL1851-16|Canada|BOLD:ACY4026
Tropocyclops sp. LE082614|GBMNE18543-21|United States|BOLD:ACY4026
Cyclopoida|COAPP307-13|Canada|BOLD:ACF8922
Cyclopoida|COAPP446-13|Canada|BOLD:ACF8922
Tropocyclops prasinus mexicanus|BACZP1131-16|Canada|BOLD:ACF8922
Tropocyclops prasinus mexicanus|BACZP1265-16|Canada|BOLD:ACF8922
Tropocyclops prasinus mexicanus|BACZP1267-16|Canada|BOLD:ACF8922
Tropocyclops prasinus mexicanus|BACZP1130-16|Canada|BOLD:ACF8922
Tropocyclops prasinus mexicanus|BACZP1263-16|Canada|BOLD:ACF8922
Tropocyclops prasinus mexicanus|BACZP1272-16|Canada|BOLD:ACF8922
Tropocyclops prasinus mexicanus|BACZP1293-16|Canada|BOLD:ACF8922
Tropocyclops prasinus mexicanus|ZOOPS248-19|United States|BOLD:ACF8922
Tropocyclops prasinus mexicanus|ZOOPS249-19|United States|BOLD:ACF8922
Tropocyclops prasinus mexicanus|ZOOPS712-20|Canada|BOLD:ACF8922
Tropocyclops prasinus|EXDB452-20|Mexico|BOLD:AAG0694
Cyclopidae|ZPLCA011-06|Canada|BOLD:AAG0694
Tropocyclops prasinus|COAPP457-13|Canada|BOLD:AAG0694
Tropocyclops prasinus|COAPP540-13|Canada|BOLD:AAG0694
Tropocyclops prasinus|COAPP456-13|Canada|BOLD:AAG0694
Tropocyclops prasinus|COAPP564-13|Canada|BOLD:AAG0694
Tropocyclops prasinus|BACZP1397-16|Canada|BOLD:AAG0694
Tropocyclops prasinus|BACZP1433-16|Canada|BOLD:AAG0694
Tropocyclops prasinus|COAPP550-13|Canada|BOLD:AAG0694
Tropocyclops prasinus|COAPP367-13|Canada|BOLD:AAG0694
Eucyclopinae|COAPP290-13|Canada|BOLD:AAG0694
Tropocyclops prasinus|COAPP563-13|Canada|BOLD:AAG0694
Tropocyclops prasinus|BACZP1502-16|Canada|BOLD:AAG0694
Tropocyclops prasinus|COAPP330-13|Canada|BOLD:AAG0694
Tropocyclops prasinus|COAPP417-13|Canada|BOLD:AAG0694
Tropocyclops prasinus|COAPP368-13|Canada|BOLD:AAG0694
Tropocyclops prasinus mexicanus|ZOOPS055-18|United States|BOLD:AAG0694
Tropocyclops prasinus mexicanus|ZOOPS056-18|United States|BOLD:AAG0694
Tropocyclops prasinus|COAPP331-13|Canada|BOLD:AAG0694
Tropocyclops prasinus|COAPP418-13|Canada|BOLD:AAG0694
Tropocyclops prasinus|COAPP317-13|Canada|BOLD:AAG0694
Tropocyclops prasinus|COAPP501-13|Canada|BOLD:AAG0694

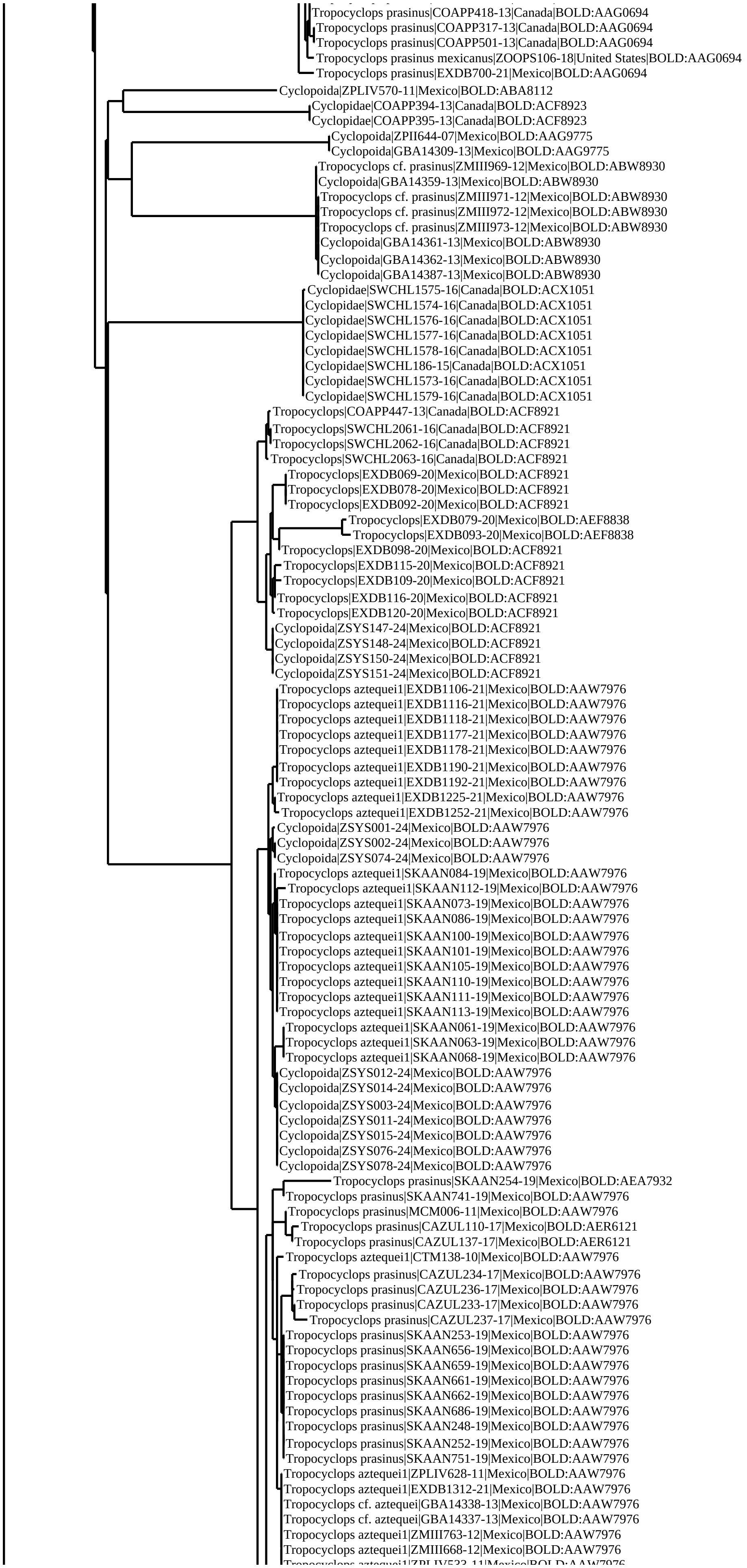
Tropocyclops prasinus|COAPP418-13|Canada|BOLD:AAG0694
Tropocyclops prasinus|COAPP317-13|Canada|BOLD:AAG0694
Tropocyclops prasinus|COAPP501-13|Canada|BOLD:AAG0694
Tropocyclops prasinus mexicanus|ZOOPS106-18|United States|BOLD:AAG0694
Tropocyclops prasinus|EXDB700-21|Mexico|BOLD:AAG0694
Cyclopoida|ZPLIV570-11|Mexico|BOLD:ABA8112
Cyclopidae|COAPP394-13|Canada|BOLD:ACF8923
Cyclopidae|COAPP395-13|Canada|BOLD:ACF8923
Cyclopoida|ZPII644-07|Mexico|BOLD:AAG9775
Cyclopoida|GBA14309-13|Mexico|BOLD:AAG9775
Tropocyclops cf. prasinus|ZMIII969-12|Mexico|BOLD:ABW8930
Cyclopoida|GBA14359-13|Mexico|BOLD:ABW8930
Tropocyclops cf. prasinus|ZMIII971-12|Mexico|BOLD:ABW8930
Tropocyclops cf. prasinus|ZMIII972-12|Mexico|BOLD:ABW8930
Tropocyclops cf. prasinus|ZMIII973-12|Mexico|BOLD:ABW8930
Cyclopoida|GBA14361-13|Mexico|BOLD:ABW8930
Cyclopoida|GBA14362-13|Mexico|BOLD:ABW8930
Cyclopoida|GBA14387-13|Mexico|BOLD:ABW8930
Cyclopidae|SWCHL1575-16|Canada|BOLD:ACX1051
Cyclopidae|SWCHL1574-16|Canada|BOLD:ACX1051
Cyclopidae|SWCHL1576-16|Canada|BOLD:ACX1051
Cyclopidae|SWCHL1577-16|Canada|BOLD:ACX1051
Cyclopidae|SWCHL1578-16|Canada|BOLD:ACX1051
Cyclopidae|SWCHL186-15|Canada|BOLD:ACX1051
Cyclopidae|SWCHL1573-16|Canada|BOLD:ACX1051
Cyclopidae|SWCHL1579-16|Canada|BOLD:ACX1051
Tropocyclops|COAPP447-13|Canada|BOLD:ACF8921
Tropocyclops|SWCHL2061-16|Canada|BOLD:ACF8921
Tropocyclops|SWCHL2062-16|Canada|BOLD:ACF8921
Tropocyclops|SWCHL2063-16|Canada|BOLD:ACF8921
Tropocyclops|EXDB069-20|Mexico|BOLD:ACF8921
Tropocyclops|EXDB078-20|Mexico|BOLD:ACF8921
Tropocyclops|EXDB092-20|Mexico|BOLD:ACF8921
Tropocyclops|EXDB079-20|Mexico|BOLD:AEF8838
Tropocyclops|EXDB093-20|Mexico|BOLD:AEF8838
Tropocyclops|EXDB098-20|Mexico|BOLD:ACF8921
Tropocyclops|EXDB115-20|Mexico|BOLD:ACF8921
Tropocyclops|EXDB109-20|Mexico|BOLD:ACF8921
Tropocyclops|EXDB116-20|Mexico|BOLD:ACF8921
Tropocyclops|EXDB120-20|Mexico|BOLD:ACF8921
Cyclopoida|ZSYS147-24|Mexico|BOLD:ACF8921
Cyclopoida|ZSYS148-24|Mexico|BOLD:ACF8921
Cyclopoida|ZSYS150-24|Mexico|BOLD:ACF8921
Cyclopoida|ZSYS151-24|Mexico|BOLD:ACF8921
Tropocyclops aztequei1|EXDB1106-21|Mexico|BOLD:AAW7976
Tropocyclops aztequei1|EXDB1116-21|Mexico|BOLD:AAW7976
Tropocyclops aztequei1|EXDB1118-21|Mexico|BOLD:AAW7976
Tropocyclops aztequei1|EXDB1177-21|Mexico|BOLD:AAW7976
Tropocyclops aztequei1|EXDB1178-21|Mexico|BOLD:AAW7976
Tropocyclops aztequei1|EXDB1190-21|Mexico|BOLD:AAW7976
Tropocyclops aztequei1|EXDB1192-21|Mexico|BOLD:AAW7976
Tropocyclops aztequei1|EXDB1225-21|Mexico|BOLD:AAW7976
Tropocyclops aztequei1|EXDB1252-21|Mexico|BOLD:AAW7976
Cyclopoida|ZSYS001-24|Mexico|BOLD:AAW7976
Cyclopoida|ZSYS002-24|Mexico|BOLD:AAW7976
Cyclopoida|ZSYS074-24|Mexico|BOLD:AAW7976
Tropocyclops aztequei1|SKAAN084-19|Mexico|BOLD:AAW7976
Tropocyclops aztequei1|SKAAN112-19|Mexico|BOLD:AAW7976
Tropocyclops aztequei1|SKAAN073-19|Mexico|BOLD:AAW7976
Tropocyclops aztequei1|SKAAN086-19|Mexico|BOLD:AAW7976
Tropocyclops aztequei1|SKAAN100-19|Mexico|BOLD:AAW7976
Tropocyclops aztequei1|SKAAN101-19|Mexico|BOLD:AAW7976
Tropocyclops aztequei1|SKAAN105-19|Mexico|BOLD:AAW7976
Tropocyclops aztequei1|SKAAN110-19|Mexico|BOLD:AAW7976
Tropocyclops aztequei1|SKAAN111-19|Mexico|BOLD:AAW7976
Tropocyclops aztequei1|SKAAN113-19|Mexico|BOLD:AAW7976
Tropocyclops aztequei1|SKAAN061-19|Mexico|BOLD:AAW7976
Tropocyclops aztequei1|SKAAN063-19|Mexico|BOLD:AAW7976
Tropocyclops aztequei1|SKAAN068-19|Mexico|BOLD:AAW7976
Cyclopoida|ZSYS012-24|Mexico|BOLD:AAW7976
Cyclopoida|ZSYS014-24|Mexico|BOLD:AAW7976
Cyclopoida|ZSYS003-24|Mexico|BOLD:AAW7976
Cyclopoida|ZSYS011-24|Mexico|BOLD:AAW7976
Cyclopoida|ZSYS015-24|Mexico|BOLD:AAW7976
Cyclopoida|ZSYS076-24|Mexico|BOLD:AAW7976
Cyclopoida|ZSYS078-24|Mexico|BOLD:AAW7976
Tropocyclops prasinus|SKAAN254-19|Mexico|BOLD:AEA7932
Tropocyclops prasinus|SKAAN741-19|Mexico|BOLD:AAW7976
Tropocyclops prasinus|MCM006-11|Mexico|BOLD:AAW7976
Tropocyclops prasinus|CAZUL110-17|Mexico|BOLD:AER6121
Tropocyclops prasinus|CAZUL137-17|Mexico|BOLD:AER6121
Tropocyclops aztequei1|CTM138-10|Mexico|BOLD:AAW7976
Tropocyclops prasinus|CAZUL234-17|Mexico|BOLD:AAW7976
Tropocyclops prasinus|CAZUL236-17|Mexico|BOLD:AAW7976
Tropocyclops prasinus|CAZUL233-17|Mexico|BOLD:AAW7976
Tropocyclops prasinus|CAZUL237-17|Mexico|BOLD:AAW7976
Tropocyclops prasinus|SKAAN253-19|Mexico|BOLD:AAW7976
Tropocyclops prasinus|SKAAN656-19|Mexico|BOLD:AAW7976
Tropocyclops prasinus|SKAAN659-19|Mexico|BOLD:AAW7976
Tropocyclops prasinus|SKAAN661-19|Mexico|BOLD:AAW7976
Tropocyclops prasinus|SKAAN662-19|Mexico|BOLD:AAW7976
Tropocyclops prasinus|SKAAN686-19|Mexico|BOLD:AAW7976
Tropocyclops prasinus|SKAAN248-19|Mexico|BOLD:AAW7976
Tropocyclops prasinus|SKAAN252-19|Mexico|BOLD:AAW7976
Tropocyclops prasinus|SKAAN751-19|Mexico|BOLD:AAW7976
Tropocyclops aztequei1|ZPLIV628-11|Mexico|BOLD:AAW7976
Tropocyclops aztequei1|EXDB1312-21|Mexico|BOLD:AAW7976
Tropocyclops cf. aztequei|GBA14338-13|Mexico|BOLD:AAW7976
Tropocyclops cf. aztequei|GBA14337-13|Mexico|BOLD:AAW7976
Tropocyclops aztequei1|ZMIII763-12|Mexico|BOLD:AAW7976
Tropocyclops aztequei1|ZMIII668-12|Mexico|BOLD:AAW7976

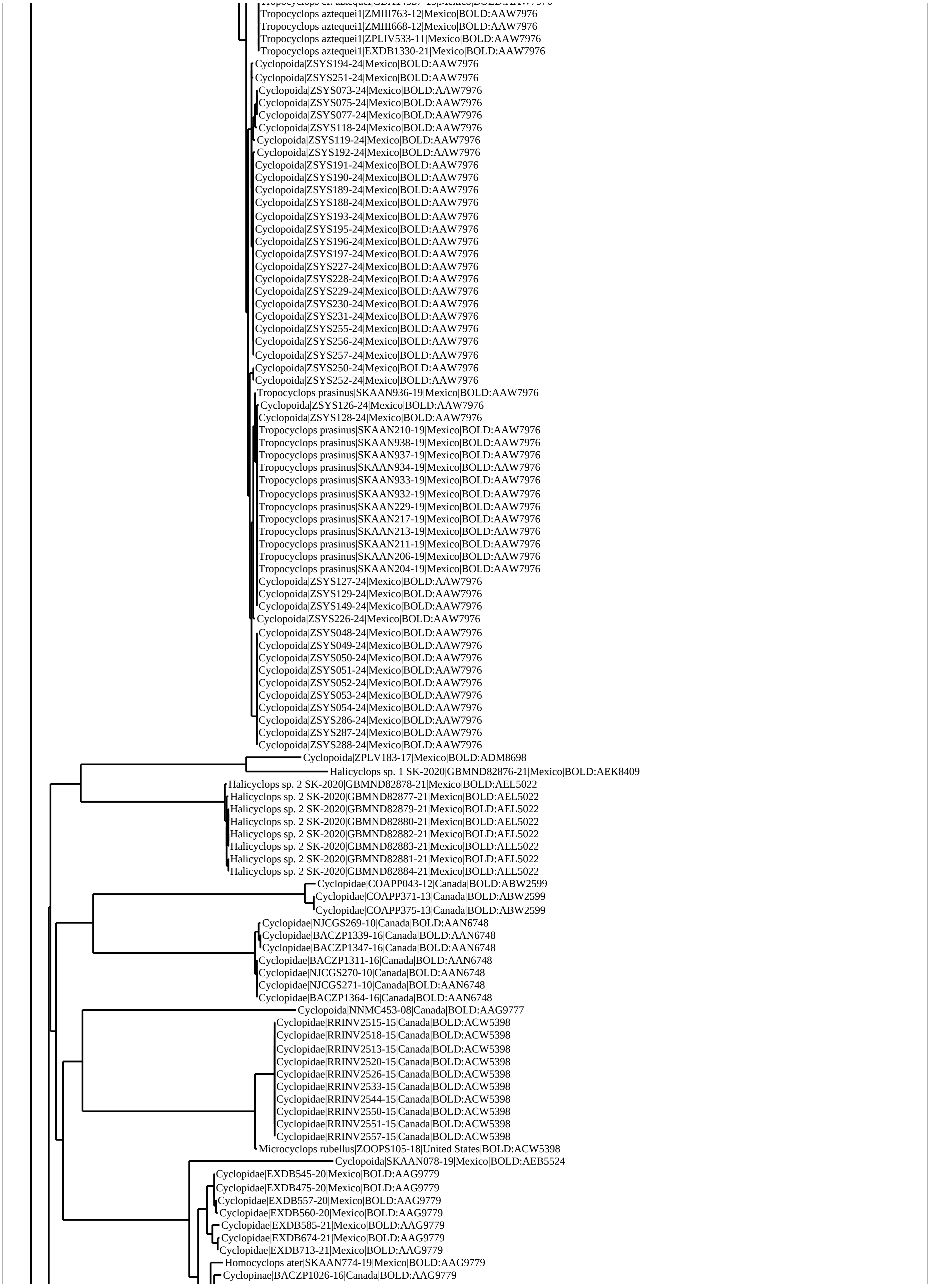
Tropocyclops aztequei1|ZMIII763-12|Mexico|BOLD:AAW7976
Tropocyclops aztequei1|ZMIII668-12|Mexico|BOLD:AAW7976
Tropocyclops aztequei1|ZPLIV533-11|Mexico|BOLD:AAW7976
Tropocyclops aztequei1|EXDB1330-21|Mexico|BOLD:AAW7976
Cyclopoida|ZSYS194-24|Mexico|BOLD:AAW7976
Cyclopoida|ZSYS251-24|Mexico|BOLD:AAW7976
Cyclopoida|ZSYS073-24|Mexico|BOLD:AAW7976
Cyclopoida|ZSYS075-24|Mexico|BOLD:AAW7976
Cyclopoida|ZSYS077-24|Mexico|BOLD:AAW7976
Cyclopoida|ZSYS118-24|Mexico|BOLD:AAW7976
Cyclopoida|ZSYS119-24|Mexico|BOLD:AAW7976
Cyclopoida|ZSYS192-24|Mexico|BOLD:AAW7976
Cyclopoida|ZSYS191-24|Mexico|BOLD:AAW7976
Cyclopoida|ZSYS190-24|Mexico|BOLD:AAW7976
Cyclopoida|ZSYS189-24|Mexico|BOLD:AAW7976
Cyclopoida|ZSYS188-24|Mexico|BOLD:AAW7976
Cyclopoida|ZSYS193-24|Mexico|BOLD:AAW7976
Cyclopoida|ZSYS195-24|Mexico|BOLD:AAW7976
Cyclopoida|ZSYS196-24|Mexico|BOLD:AAW7976
Cyclopoida|ZSYS197-24|Mexico|BOLD:AAW7976
Cyclopoida|ZSYS227-24|Mexico|BOLD:AAW7976
Cyclopoida|ZSYS228-24|Mexico|BOLD:AAW7976
Cyclopoida|ZSYS229-24|Mexico|BOLD:AAW7976
Cyclopoida|ZSYS230-24|Mexico|BOLD:AAW7976
Cyclopoida|ZSYS231-24|Mexico|BOLD:AAW7976
Cyclopoida|ZSYS255-24|Mexico|BOLD:AAW7976
Cyclopoida|ZSYS256-24|Mexico|BOLD:AAW7976
Cyclopoida|ZSYS257-24|Mexico|BOLD:AAW7976
Cyclopoida|ZSYS250-24|Mexico|BOLD:AAW7976
Cyclopoida|ZSYS252-24|Mexico|BOLD:AAW7976
Tropocyclops prasinus|SKAAN936-19|Mexico|BOLD:AAW7976
Cyclopoida|ZSYS126-24|Mexico|BOLD:AAW7976
Cyclopoida|ZSYS128-24|Mexico|BOLD:AAW7976
Tropocyclops prasinus|SKAAN210-19|Mexico|BOLD:AAW7976
Tropocyclops prasinus|SKAAN938-19|Mexico|BOLD:AAW7976
Tropocyclops prasinus|SKAAN937-19|Mexico|BOLD:AAW7976
Tropocyclops prasinus|SKAAN934-19|Mexico|BOLD:AAW7976
Tropocyclops prasinus|SKAAN933-19|Mexico|BOLD:AAW7976
Tropocyclops prasinus|SKAAN932-19|Mexico|BOLD:AAW7976
Tropocyclops prasinus|SKAAN229-19|Mexico|BOLD:AAW7976
Tropocyclops prasinus|SKAAN217-19|Mexico|BOLD:AAW7976
Tropocyclops prasinus|SKAAN213-19|Mexico|BOLD:AAW7976
Tropocyclops prasinus|SKAAN211-19|Mexico|BOLD:AAW7976
Tropocyclops prasinus|SKAAN206-19|Mexico|BOLD:AAW7976
Tropocyclops prasinus|SKAAN204-19|Mexico|BOLD:AAW7976
Cyclopoida|ZSYS127-24|Mexico|BOLD:AAW7976
Cyclopoida|ZSYS129-24|Mexico|BOLD:AAW7976
Cyclopoida|ZSYS149-24|Mexico|BOLD:AAW7976
Cyclopoida|ZSYS226-24|Mexico|BOLD:AAW7976
Cyclopoida|ZSYS048-24|Mexico|BOLD:AAW7976
Cyclopoida|ZSYS049-24|Mexico|BOLD:AAW7976
Cyclopoida|ZSYS050-24|Mexico|BOLD:AAW7976
Cyclopoida|ZSYS051-24|Mexico|BOLD:AAW7976
Cyclopoida|ZSYS052-24|Mexico|BOLD:AAW7976
Cyclopoida|ZSYS053-24|Mexico|BOLD:AAW7976
Cyclopoida|ZSYS054-24|Mexico|BOLD:AAW7976
Cyclopoida|ZSYS286-24|Mexico|BOLD:AAW7976
Cyclopoida|ZSYS287-24|Mexico|BOLD:AAW7976
Cyclopoida|ZSYS288-24|Mexico|BOLD:AAW7976
Cyclopoida|ZPLV183-17|Mexico|BOLD:ADM8698
Halicyclops sp. 1 SK-2020|GBMND82876-21|Mexico|BOLD:AEK8409
Halicyclops sp. 2 SK-2020|GBMND82878-21|Mexico|BOLD:AEL5022
Halicyclops sp. 2 SK-2020|GBMND82877-21|Mexico|BOLD:AEL5022
Halicyclops sp. 2 SK-2020|GBMND82879-21|Mexico|BOLD:AEL5022
Halicyclops sp. 2 SK-2020|GBMND82880-21|Mexico|BOLD:AEL5022
Halicyclops sp. 2 SK-2020|GBMND82882-21|Mexico|BOLD:AEL5022
Halicyclops sp. 2 SK-2020|GBMND82883-21|Mexico|BOLD:AEL5022
Halicyclops sp. 2 SK-2020|GBMND82881-21|Mexico|BOLD:AEL5022
Halicyclops sp. 2 SK-2020|GBMND82884-21|Mexico|BOLD:AEL5022
Cyclopidae|COAPP043-12|Canada|BOLD:ABW2599
Cyclopidae|COAPP371-13|Canada|BOLD:ABW2599
Cyclopidae|COAPP375-13|Canada|BOLD:ABW2599
Cyclopidae|NJCGS269-10|Canada|BOLD:AAN6748
Cyclopidae|BACZP1339-16|Canada|BOLD:AAN6748
Cyclopidae|BACZP1347-16|Canada|BOLD:AAN6748
Cyclopidae|BACZP1311-16|Canada|BOLD:AAN6748
Cyclopidae|NJCGS270-10|Canada|BOLD:AAN6748
Cyclopidae|NJCGS271-10|Canada|BOLD:AAN6748
Cyclopidae|BACZP1364-16|Canada|BOLD:AAN6748
Cyclopoida|NNMC453-08|Canada|BOLD:AAG9777
Cyclopidae|RRINV2515-15|Canada|BOLD:ACW5398
Cyclopidae|RRINV2518-15|Canada|BOLD:ACW5398
Cyclopidae|RRINV2513-15|Canada|BOLD:ACW5398
Cyclopidae|RRINV2520-15|Canada|BOLD:ACW5398
Cyclopidae|RRINV2526-15|Canada|BOLD:ACW5398
Cyclopidae|RRINV2533-15|Canada|BOLD:ACW5398
Cyclopidae|RRINV2544-15|Canada|BOLD:ACW5398
Cyclopidae|RRINV2550-15|Canada|BOLD:ACW5398
Cyclopidae|RRINV2551-15|Canada|BOLD:ACW5398
Cyclopidae|RRINV2557-15|Canada|BOLD:ACW5398
Microcyclops rubellus|ZOOPS105-18|United States|BOLD:ACW5398
Cyclopoida|SKAAN078-19|Mexico|BOLD:AEB5524
Cyclopidae|EXDB545-20|Mexico|BOLD:AAG9779
Cyclopidae|EXDB475-20|Mexico|BOLD:AAG9779
Cyclopidae|EXDB557-20|Mexico|BOLD:AAG9779
Cyclopidae|EXDB560-20|Mexico|BOLD:AAG9779
Cyclopidae|EXDB585-21|Mexico|BOLD:AAG9779
Cyclopidae|EXDB674-21|Mexico|BOLD:AAG9779
Cyclopidae|EXDB713-21|Mexico|BOLD:AAG9779
Homocyclops ater|SKAAN774-19|Mexico|BOLD:AAG9779
Cyclopinae|BACZP1026-16|Canada|BOLD:AAG9779

Cyclopidae|EXDB713-21|Mexico|BOLD:AAG9779
Homocyclops ater|SKAAN774-19|Mexico|BOLD:AAG9779
Cyclopinae|BACZP1026-16|Canada|BOLD:AAG9779
Cyclopinae|ZPLV547-18|Mexico|BOLD:AAG9779
Homocyclops ater|EXDB1000-21|Mexico|BOLD:AAG9779
Homocyclops ater|SKAAN651-19|Mexico|BOLD:AAG9779
Homocyclops ater|SKAAN796-19|Mexico|BOLD:AAG9779
Homocyclops ater|EXDB1188-21|Mexico|BOLD:AAG9779
Homocyclops ater|EXDB1337-21|Mexico|BOLD:AAG9779
Homocyclops ater|EXDB1364-21|Mexico|BOLD:AAG9779
Homocyclops ater|EXDB1391-21|Mexico|BOLD:AAG9779
Cyclopinae|SWCHL1655-16|Canada|BOLD:AAG9779
Cyclopinae|RRINV2552-15|Canada|BOLD:AAG9779
Cyclopinae|CRCN053-09|Canada|BOLD:AAG9779
Cyclopinae|SWCHL1654-16|Canada|BOLD:AAG9779
Cyclopinae|SWCHL1653-16|Canada|BOLD:AAG9779
Cyclopinae|BACZP1081-16|Canada|BOLD:AAG9779
Cyclopinae|RBNII366-13|Canada|BOLD:AAG9779
Homocyclops ater|GBMNE18537-21|United States|BOLD:AAG9779
Mesocyclops edax|EXDB799-21|Mexico|BOLD:AEI4896
Mesocyclops edax|EXDB357-20|Mexico|BOLD:AEI4896
Mesocyclops edax|EXDB346-20|Mexico|BOLD:AEI4896
Mesocyclops edax|EXDB302-20|Mexico|BOLD:AEI4896
Mesocyclops edax|EXDB247-20|Mexico|BOLD:AEI4896
Cyclopoida|EXDB230-20|Mexico|BOLD:AEI4896
Mesocyclops edax|EXDB215-20|Mexico|BOLD:AEI4896
Mesocyclops edax|EXDB183-20|Mexico|BOLD:AEI4896
Mesocyclops edax|EXDB165-20|Mexico|BOLD:AEI4896
Mesocyclops edax|EXDB159-20|Mexico|BOLD:AEI4896
Mesocyclops edax|EXDB153-20|Mexico|BOLD:AEI4896
Mesocyclops edax|EXDB144-20|Mexico|BOLD:AEI4896
Mesocyclops edax|EXDB131-20|Mexico|BOLD:AEI4896
Mesocyclops edax|EXDB125-20|Mexico|BOLD:AEI4896
Mesocyclops edax|EXDB328-20|Mexico|BOLD:AEI4896
Mesocyclops edax|EXDB148-20|Mexico|BOLD:AEI4896
Mesocyclops edax|EXDB162-20|Mexico|BOLD:AEI4896
Mesocyclops edax|EXDB243-20|Mexico|BOLD:AEI4896
Mesocyclops edax|EXDB335-20|Mexico|BOLD:AEI4896
Mesocyclops edax|EXDB363-20|Mexico|BOLD:AEI4896
Mesocyclops edax|EXDB124-20|Mexico|BOLD:AEI4896
Mesocyclops edax|EXDB155-20|Mexico|BOLD:AEI4896
Mesocyclops edax|EXDB367-20|Mexico|BOLD:AEI4896
Mesocyclops edax|EXDB173-20|Mexico|BOLD:AEI4896
Mesocyclops edax|EXDB171-20|Mexico|BOLD:AEI4896
Mesocyclops edax|EXDB137-20|Mexico|BOLD:AEI4896
Mesocyclops edax|EXDB161-20|Mexico|BOLD:AEI4896
Mesocyclops edax|EXDB294-20|Mexico|BOLD:AEI4896
Mesocyclops edax|EXDB316-20|Mexico|BOLD:AEI4896
Mesocyclops edax|EXDB334-20|Mexico|BOLD:AEI4896
Mesocyclops edax|EXDB341-20|Mexico|BOLD:AEI4896
Mesocyclops edax|EXDB364-20|Mexico|BOLD:AEI4896
Mesocyclops edax|EXDB327-20|Mexico|BOLD:AEI4896
Mesocyclops edax|EXDB368-20|Mexico|BOLD:AEI4896
Mesocyclops edax|EXDB458-20|Mexico|BOLD:AEI4896
Mesocyclops edax|EXDB587-21|Mexico|BOLD:AEI4896
Mesocyclops edax|EXDB639-21|Mexico|BOLD:AEI4896
Mesocyclops edax|SKAAN731-19|Mexico|BOLD:AEI4896
Mesocyclops edax|SKAAN744-19|Mexico|BOLD:AEI4896
Mesocyclops edax|SKAAN739-19|Mexico|BOLD:AEI4896
Mesocyclops edax|SKAAN730-19|Mexico|BOLD:AEI4896
Mesocyclops edax|SKAAN711-19|Mexico|BOLD:AEI4896
Mesocyclops edax|SKAAN757-19|Mexico|BOLD:AEI4896
Mesocyclops edax|SKAAN287-19|Mexico|BOLD:AEI4896
Mesocyclops edax|SKAAN293-19|Mexico|BOLD:AEI4896
Mesocyclops edax|SKAAN286-19|Mexico|BOLD:AEI4896
Mesocyclops edax|SKAAN284-19|Mexico|BOLD:AEI4896
Mesocyclops edax|SKAAN278-19|Mexico|BOLD:AEI4896
Mesocyclops edax|SKAAN276-19|Mexico|BOLD:AEI4896
Mesocyclops edax|SKAAN274-19|Mexico|BOLD:AEI4896
Mesocyclops edax|SKAAN268-19|Mexico|BOLD:AEI4896
Mesocyclops edax|SKAAN266-19|Mexico|BOLD:AEI4896
Mesocyclops edax|SKAAN264-19|Mexico|BOLD:AEI4896
Mesocyclops edax|SKAAN262-19|Mexico|BOLD:AEI4896
Mesocyclops|SKAAN288-19|Mexico|BOLD:AEI4897
Mesocyclops edax|SKAAN294-19|Mexico|BOLD:AEI4896
Mesocyclops edax|SKAAN709-19|Mexico|BOLD:AEI4896
Mesocyclops edax|SKAAN719-19|Mexico|BOLD:AEI4896
Mesocyclops edax|SKAAN848-19|Mexico|BOLD:AEI4896
Mesocyclops edax|SKAAN845-19|Mexico|BOLD:AEI4896
Mesocyclops edax|SKAAN844-19|Mexico|BOLD:AEI4896
Mesocyclops edax|SKAAN847-19|Mexico|BOLD:AEI4896
Mesocyclops edax|SKAAN853-19|Mexico|BOLD:AEI4896
Mesocyclops edax|SKAAN854-19|Mexico|BOLD:AEI4896
Mesocyclops edax|SKAAN860-19|Mexico|BOLD:AEI4896
Mesocyclops edax|SKAAN865-19|Mexico|BOLD:AEI4896
Mesocyclops edax|SKAAN880-19|Mexico|BOLD:AEI4896
Mesocyclops edax|SKAAN893-19|Mexico|BOLD:AEI4896
Mesocyclops edax|SKAAN901-19|Mexico|BOLD:AEI4896
Mesocyclops edax|SKAAN904-19|Mexico|BOLD:AEI4896
Mesocyclops edax|SKAAN269-19|Mexico|BOLD:AEI4896
Mesocyclops edax|SKAAN756-19|Mexico|BOLD:AEI4896
Mesocyclops edax|SKAAN736-19|Mexico|BOLD:AEI4896
Mesocyclops edax|SKAAN906-19|Mexico|BOLD:AEI4896
Mesocyclops edax|SKAAN724-19|Mexico|BOLD:AEI4896
Mesocyclops edax|SKAAN721-19|Mexico|BOLD:AEI4896
Mesocyclops edax|SKAAN868-19|Mexico|BOLD:AEI4896
Mesocyclops edax|SKAAN878-19|Mexico|BOLD:AEI4896
Mesocyclops edax|SKAAN924-19|Mexico|BOLD:AEI4896
Cyclopoida|EXDB229-20|Mexico|BOLD:AEI6940
Cyclopoida|EXDB235-20|Mexico
Mesocyclops edax|EXDB362-20|Mexico|BOLD:AEI4896
Mesocyclops edax|EXDB747-21|Mexico|BOLD:AEI4896

Cyclopoida|EXDB235-20|Mexico
Mesocyclops edax|EXDB362-20|Mexico|BOLD:AEI4896
Mesocyclops edax|EXDB747-21|Mexico|BOLD:AEI4896
Mesocyclops edax|EXDB1875-21|Mexico|BOLD:AEI4896
Cyclops|BCRUS135-10|United States|BOLD:AAG9230
Cyclops|BCRUS141-10|United States|BOLD:AAG9230
Cyclops|CAISN861-13|Canada|BOLD:AAG9230
Cyclops|BCRUA168-10|United States|BOLD:AAG9230
Cyclops|BCRUA167-10|United States|BOLD:AAG9230
Cyclops|BCRUA166-10|United States|BOLD:AAG9230
Cyclops|BCRUA165-10|United States|BOLD:AAG9230
Cyclops|BCRUA164-10|United States|BOLD:AAG9230
Cyclops|BCRUA163-10|United States|BOLD:AAG9230
Cyclops|BCRUS073-10|United States|BOLD:AAG9230
Cyclops|BBCRT172-12|United States|BOLD:AAG9230
Cyclops|ZPII930-11|Mexico|BOLD:AAG9230
Cyclops|GBA14308-13|Mexico|BOLD:AAG9230
Cyclops|JMCRU226-09|Canada|BOLD:AAG9230
Cyclops|BCRUS043-10|United States|BOLD:AAG9230
Cyclops|BCRUS045-10|United States|BOLD:AAG9230
Cyclops|BBCRT176-12|United States|BOLD:AAG9230
Cyclops|CAISN081-12|Canada|BOLD:AAG9230
Cyclops|CAISN374-12|Canada|BOLD:AAG9230
Cyclops|BCRUA149-10|United States|BOLD:AAG9230
Cyclops|BCRUA042-10|United States|BOLD:AAG9230
Cyclops|BCRUA223-10|United States|BOLD:AAG9230
Cyclops|BBCRT015-12|United States|BOLD:AAG9230
Cyclops|BBCRT016-12|United States|BOLD:AAG9230
Cyclops|BBCRT017-12|United States|BOLD:AAG9230
Cyclops|BBCRT018-12|United States|BOLD:AAG9230
Cyclops|BBCRT019-12|United States|BOLD:AAG9230
Cyclops|BBCRT021-12|United States|BOLD:AAG9230
Cyclops|BBCRT022-12|United States|BOLD:AAG9230
Cyclops|BBCRT023-12|United States|BOLD:AAG9230
Cyclops|BBCRT028-12|United States|BOLD:AAG9230
Cyclops|BBCRT029-12|United States|BOLD:AAG9230
Cyclops|BBCRT033-12|United States|BOLD:AAG9230
Cyclops|COAPP289-13|Canada|BOLD:AAG9230
Cyclops|COAPP294-13|Canada|BOLD:AAG9230
Cyclops|COAPP295-13|Canada|BOLD:AAG9230
Cyclops|COAPP298-13|Canada|BOLD:AAG9230
Cyclops|COAPP306-13|Canada|BOLD:AAG9230
Cyclops|BBCRU213-12|Canada|BOLD:AAG9230
Cyclops|BBCRU214-12|Canada|BOLD:AAG9230
Cyclops|RBNII293-13|Canada|BOLD:AAG9230
Cyclops|CAISN926-13|Canada|BOLD:AEI4894
Cyclops|BBCRU244-12|Canada|BOLD:AEI4894
Cyclops|BACZP782-16|Canada|BOLD:AEI4894
Mesocyclops edax|ZOOPS706-20|Canada|BOLD:AEI4894
Mesocyclops edax|NJNAH017-14|Canada|BOLD:AEI4895
Mesocyclops edax|NJNAH018-14|Canada|BOLD:AEI4895
Mesocyclops edax|COAPP486-13|Canada|BOLD:AEI4895
Mesocyclops edax|COAPP487-13|Canada|BOLD:AEI4895
Mesocyclops edax|NJNAH019-14|Canada|BOLD:AEI4895
Mesocyclops edax|COAPP511-13|Canada|BOLD:AEI4895
Mesocyclops edax|BACZP855-16|Canada
Mesocyclops edax|BACZP1444-16|Canada|BOLD:AEI4895
Mesocyclops edax|BACZP679-16|Canada|BOLD:AEI4895
Mesocyclops edax|BACZP760-16|Canada|BOLD:AEI4895
Mesocyclops edax|BACZP1384-16|Canada|BOLD:AEI4895
Mesocyclops edax|BACZP1391-16|Canada|BOLD:AEI4895
Mesocyclops edax|BACZP1382-16|Canada|BOLD:AEI4895
Mesocyclops edax|BACZP1383-16|Canada|BOLD:AEI4895
Mesocyclops edax|BACZP1393-16|Canada|BOLD:AEI4895
Mesocyclops edax|BACZP1394-16|Canada|BOLD:AEI4895
Mesocyclops edax|BACZP1445-16|Canada|BOLD:AEI4895
Mesocyclops edax|BACZP1446-16|Canada|BOLD:AEI4895
Mesocyclops edax|BACZP789-16|Canada|BOLD:AEI4895
Mesocyclops edax|BACZP1665-16|Canada|BOLD:AEI4895
Mesocyclops edax|BACZP1611-16|Canada|BOLD:AEI4895
Mesocyclops edax|ZOOPS709-20|Canada|BOLD:AEI4895
Mesocyclops edax|BBCRU226-12|Canada|BOLD:AEI4895
Mesocyclops edax|BACZP1214-16|Canada|BOLD:AEI4895
Mesocyclops edax|BACZP1561-16|Canada|BOLD:AEI4895
Mesocyclops edax|BACZP1269-16|Canada|BOLD:AEI4895
Mesocyclops edax|BACZP1216-16|Canada|BOLD:AEI4895
Mesocyclops edax|BACZP1213-16|Canada|BOLD:AEI4895
Mesocyclops edax|BACZP1212-16|Canada|BOLD:AEI4895
Mesocyclops edax|BACZP1210-16|Canada|BOLD:AEI4895
Mesocyclops edax|BACZP1208-16|Canada|BOLD:AEI4895
Mesocyclops edax|SWCHL182-15|Canada|BOLD:AEI4895
Mesocyclops edax|SWCHL1475-16|Canada|BOLD:AEI4895
Mesocyclops edax|BACZP1209-16|Canada|BOLD:AEI4895
Mesocyclops edax|BACZP1215-16|Canada|BOLD:AEI4895
Mesocyclops edax|BACZP1579-16|Canada|BOLD:AEI4895
Mesocyclops edax|CAISN005-12|Canada|BOLD:AEI4895
Mesocyclops edax|CAISN366-12|Canada|BOLD:AEI4895
Mesocyclops edax|ZOOPS710-20|Canada|BOLD:AEI4895
Mesocyclops edax|BACZP834-16|Canada|BOLD:AEI4895
Mesocyclops edax|BACZP790-16|Canada|BOLD:AEI4895
Mesocyclops edax|BACZP676-16|Canada|BOLD:AEI4895
Mesocyclops edax|COAPP515-13|Canada|BOLD:AEI4895
Mesocyclops edax|COAPP502-13|Canada|BOLD:AEI4895
Mesocyclops edax|COAPP500-13|Canada|BOLD:AEI4895
Mesocyclops edax|COAPP409-13|Canada|BOLD:AEI4895
Mesocyclops edax|COAPP320-13|Canada|BOLD:AEI4895
Mesocyclops edax|COAPP319-13|Canada|BOLD:AEI4895
Mesocyclops edax|COAPP318-13|Canada|BOLD:AEI4895
Mesocyclops edax|CAISN368-12|Canada|BOLD:AEI4895
Mesocyclops edax|CAISN099-12|Canada|BOLD:AEI4895
Mesocyclops edax|CAISN052-12|Canada|BOLD:AEI4895
Mesocyclops edax|CAISN025-12|Canada|BOLD:AEI4895

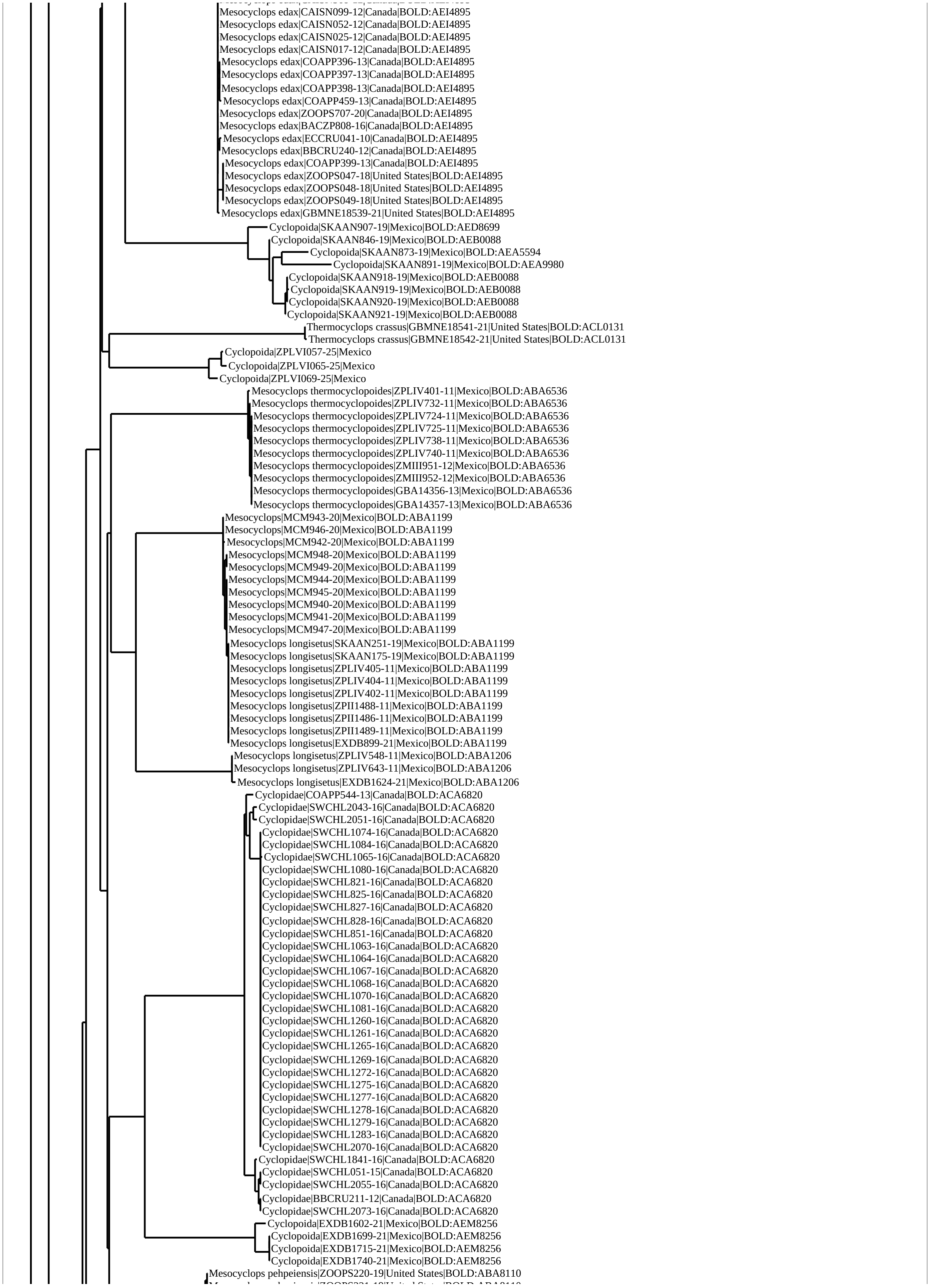
Mesocyclops edax|CAISN099-12|Canada|BOLD:AEI4895
Mesocyclops edax|CAISN052-12|Canada|BOLD:AEI4895
Mesocyclops edax|CAISN025-12|Canada|BOLD:AEI4895
Mesocyclops edax|CAISN017-12|Canada|BOLD:AEI4895
Mesocyclops edax|COAPP396-13|Canada|BOLD:AEI4895
Mesocyclops edax|COAPP397-13|Canada|BOLD:AEI4895
Mesocyclops edax|COAPP398-13|Canada|BOLD:AEI4895
Mesocyclops edax|COAPP459-13|Canada|BOLD:AEI4895
Mesocyclops edax|ZOOPS707-20|Canada|BOLD:AEI4895
Mesocyclops edax|BACZP808-16|Canada|BOLD:AEI4895
Mesocyclops edax|ECCRU041-10|Canada|BOLD:AEI4895
Mesocyclops edax|BBCRU240-12|Canada|BOLD:AEI4895
Mesocyclops edax|COAPP399-13|Canada|BOLD:AEI4895
Mesocyclops edax|ZOOPS047-18|United States|BOLD:AEI4895
Mesocyclops edax|ZOOPS048-18|United States|BOLD:AEI4895
Mesocyclops edax|ZOOPS049-18|United States|BOLD:AEI4895
Mesocyclops edax|GBMNE18539-21|United States|BOLD:AEI4895
Cyclopoida|SKAAN907-19|Mexico|BOLD:AED8699
Cyclopoida|SKAAN846-19|Mexico|BOLD:AEB0088
Cyclopoida|SKAAN873-19|Mexico|BOLD:AEA5594
Cyclopoida|SKAAN891-19|Mexico|BOLD:AEA9980
Cyclopoida|SKAAN918-19|Mexico|BOLD:AEB0088
Cyclopoida|SKAAN919-19|Mexico|BOLD:AEB0088
Cyclopoida|SKAAN920-19|Mexico|BOLD:AEB0088
Cyclopoida|SKAAN921-19|Mexico|BOLD:AEB0088
Thermocyclops crassus|GBMNE18541-21|United States|BOLD:ACL0131
Thermocyclops crassus|GBMNE18542-21|United States|BOLD:ACL0131
Cyclopoida|ZPLVI057-25|Mexico
Cyclopoida|ZPLVI065-25|Mexico
Cyclopoida|ZPLVI069-25|Mexico
Mesocyclops thermocyclopoides|ZPLIV401-11|Mexico|BOLD:ABA6536
Mesocyclops thermocyclopoides|ZPLIV732-11|Mexico|BOLD:ABA6536
Mesocyclops thermocyclopoides|ZPLIV724-11|Mexico|BOLD:ABA6536
Mesocyclops thermocyclopoides|ZPLIV725-11|Mexico|BOLD:ABA6536
Mesocyclops thermocyclopoides|ZPLIV738-11|Mexico|BOLD:ABA6536
Mesocyclops thermocyclopoides|ZPLIV740-11|Mexico|BOLD:ABA6536
Mesocyclops thermocyclopoides|ZMIII951-12|Mexico|BOLD:ABA6536
Mesocyclops thermocyclopoides|ZMIII952-12|Mexico|BOLD:ABA6536
Mesocyclops thermocyclopoides|GBA14356-13|Mexico|BOLD:ABA6536
Mesocyclops thermocyclopoides|GBA14357-13|Mexico|BOLD:ABA6536
Mesocyclops|MCM943-20|Mexico|BOLD:ABA1199
Mesocyclops|MCM946-20|Mexico|BOLD:ABA1199
Mesocyclops|MCM942-20|Mexico|BOLD:ABA1199
Mesocyclops|MCM948-20|Mexico|BOLD:ABA1199
Mesocyclops|MCM949-20|Mexico|BOLD:ABA1199
Mesocyclops|MCM944-20|Mexico|BOLD:ABA1199
Mesocyclops|MCM945-20|Mexico|BOLD:ABA1199
Mesocyclops|MCM940-20|Mexico|BOLD:ABA1199
Mesocyclops|MCM941-20|Mexico|BOLD:ABA1199
Mesocyclops|MCM947-20|Mexico|BOLD:ABA1199
Mesocyclops longisetus|SKAAN251-19|Mexico|BOLD:ABA1199
Mesocyclops longisetus|SKAAN175-19|Mexico|BOLD:ABA1199
Mesocyclops longisetus|ZPLIV405-11|Mexico|BOLD:ABA1199
Mesocyclops longisetus|ZPLIV404-11|Mexico|BOLD:ABA1199
Mesocyclops longisetus|ZPLIV402-11|Mexico|BOLD:ABA1199
Mesocyclops longisetus|ZPII1488-11|Mexico|BOLD:ABA1199
Mesocyclops longisetus|ZPII1486-11|Mexico|BOLD:ABA1199
Mesocyclops longisetus|ZPII1489-11|Mexico|BOLD:ABA1199
Mesocyclops longisetus|EXDB899-21|Mexico|BOLD:ABA1199
Mesocyclops longisetus|ZPLIV548-11|Mexico|BOLD:ABA1206
Mesocyclops longisetus|ZPLIV643-11|Mexico|BOLD:ABA1206
Mesocyclops longisetus|EXDB1624-21|Mexico|BOLD:ABA1206
Cyclopidae|COAPP544-13|Canada|BOLD:ACA6820
Cyclopidae|SWCHL2043-16|Canada|BOLD:ACA6820
Cyclopidae|SWCHL2051-16|Canada|BOLD:ACA6820
Cyclopidae|SWCHL1074-16|Canada|BOLD:ACA6820
Cyclopidae|SWCHL1084-16|Canada|BOLD:ACA6820
Cyclopidae|SWCHL1065-16|Canada|BOLD:ACA6820
Cyclopidae|SWCHL1080-16|Canada|BOLD:ACA6820
Cyclopidae|SWCHL821-16|Canada|BOLD:ACA6820
Cyclopidae|SWCHL825-16|Canada|BOLD:ACA6820
Cyclopidae|SWCHL827-16|Canada|BOLD:ACA6820
Cyclopidae|SWCHL828-16|Canada|BOLD:ACA6820
Cyclopidae|SWCHL851-16|Canada|BOLD:ACA6820
Cyclopidae|SWCHL1063-16|Canada|BOLD:ACA6820
Cyclopidae|SWCHL1064-16|Canada|BOLD:ACA6820
Cyclopidae|SWCHL1067-16|Canada|BOLD:ACA6820
Cyclopidae|SWCHL1068-16|Canada|BOLD:ACA6820
Cyclopidae|SWCHL1070-16|Canada|BOLD:ACA6820
Cyclopidae|SWCHL1081-16|Canada|BOLD:ACA6820
Cyclopidae|SWCHL1260-16|Canada|BOLD:ACA6820
Cyclopidae|SWCHL1261-16|Canada|BOLD:ACA6820
Cyclopidae|SWCHL1265-16|Canada|BOLD:ACA6820
Cyclopidae|SWCHL1269-16|Canada|BOLD:ACA6820
Cyclopidae|SWCHL1272-16|Canada|BOLD:ACA6820
Cyclopidae|SWCHL1275-16|Canada|BOLD:ACA6820
Cyclopidae|SWCHL1277-16|Canada|BOLD:ACA6820
Cyclopidae|SWCHL1278-16|Canada|BOLD:ACA6820
Cyclopidae|SWCHL1279-16|Canada|BOLD:ACA6820
Cyclopidae|SWCHL1283-16|Canada|BOLD:ACA6820
Cyclopidae|SWCHL2070-16|Canada|BOLD:ACA6820
Cyclopidae|SWCHL1841-16|Canada|BOLD:ACA6820
Cyclopidae|SWCHL051-15|Canada|BOLD:ACA6820
Cyclopidae|SWCHL2055-16|Canada|BOLD:ACA6820
Cyclopidae|BBCRU211-12|Canada|BOLD:ACA6820
Cyclopidae|SWCHL2073-16|Canada|BOLD:ACA6820
Cyclopoida|EXDB1602-21|Mexico|BOLD:AEM8256
Cyclopoida|EXDB1699-21|Mexico|BOLD:AEM8256
Cyclopoida|EXDB1715-21|Mexico|BOLD:AEM8256
Cyclopoida|EXDB1740-21|Mexico|BOLD:AEM8256
Mesocyclops pehpeiensis|ZOOPS220-19|United States|BOLD:ABA8110

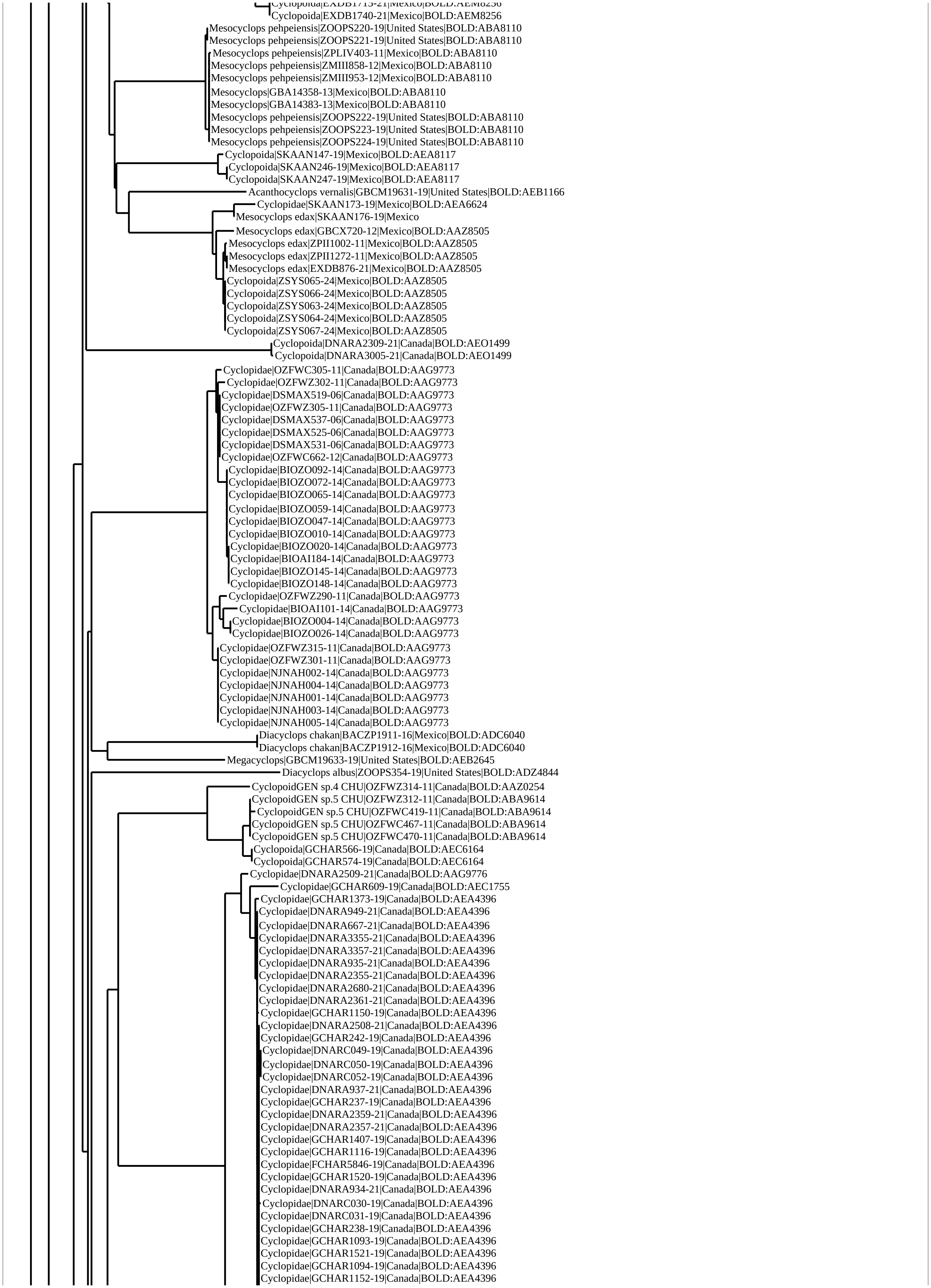

Cyclopoida|EXDB1715-21|Mexico|BOLD:AEM8256
Cyclopoida|EXDB1740-21|Mexico|BOLD:AEM8256
Mesocyclops pehpeiensis|ZOOPS220-19|United States|BOLD:ABA8110
Mesocyclops pehpeiensis|ZOOPS221-19|United States|BOLD:ABA8110
Mesocyclops pehpeiensis|ZPLIV403-11|Mexico|BOLD:ABA8110
Mesocyclops pehpeiensis|ZMIII858-12|Mexico|BOLD:ABA8110
Mesocyclops pehpeiensis|ZMIII953-12|Mexico|BOLD:ABA8110
Mesocyclops|GBA14358-13|Mexico|BOLD:ABA8110
Mesocyclops|GBA14383-13|Mexico|BOLD:ABA8110
Mesocyclops pehpeiensis|ZOOPS222-19|United States|BOLD:ABA8110
Mesocyclops pehpeiensis|ZOOPS223-19|United States|BOLD:ABA8110
Mesocyclops pehpeiensis|ZOOPS224-19|United States|BOLD:ABA8110
Cyclopoida|SKAAN147-19|Mexico|BOLD:AEA8117
Cyclopoida|SKAAN246-19|Mexico|BOLD:AEA8117
Cyclopoida|SKAAN247-19|Mexico|BOLD:AEA8117
Acanthocyclops vernalis|GBCM19631-19|United States|BOLD:AEB1166
Cyclopidae|SKAAN173-19|Mexico|BOLD:AEA6624
Mesocyclops edax|SKAAN176-19|Mexico
Mesocyclops edax|GBCX720-12|Mexico|BOLD:AAZ8505
Mesocyclops edax|ZPII1002-11|Mexico|BOLD:AAZ8505
Mesocyclops edax|ZPII1272-11|Mexico|BOLD:AAZ8505
Mesocyclops edax|EXDB876-21|Mexico|BOLD:AAZ8505
Cyclopoida|ZSYS065-24|Mexico|BOLD:AAZ8505
Cyclopoida|ZSYS066-24|Mexico|BOLD:AAZ8505
Cyclopoida|ZSYS063-24|Mexico|BOLD:AAZ8505
Cyclopoida|ZSYS064-24|Mexico|BOLD:AAZ8505
Cyclopoida|ZSYS067-24|Mexico|BOLD:AAZ8505
Cyclopoida|DNARA2309-21|Canada|BOLD:AEO1499
Cyclopoida|DNARA3005-21|Canada|BOLD:AEO1499
Cyclopidae|OZFWC305-11|Canada|BOLD:AAG9773
Cyclopidae|OZFWZ302-11|Canada|BOLD:AAG9773
Cyclopidae|DSMAX519-06|Canada|BOLD:AAG9773
Cyclopidae|OZFWZ305-11|Canada|BOLD:AAG9773
Cyclopidae|DSMAX537-06|Canada|BOLD:AAG9773
Cyclopidae|DSMAX525-06|Canada|BOLD:AAG9773
Cyclopidae|DSMAX531-06|Canada|BOLD:AAG9773
Cyclopidae|OZFWC662-12|Canada|BOLD:AAG9773
Cyclopidae|BIOZO092-14|Canada|BOLD:AAG9773
Cyclopidae|BIOZO072-14|Canada|BOLD:AAG9773
Cyclopidae|BIOZO065-14|Canada|BOLD:AAG9773
Cyclopidae|BIOZO059-14|Canada|BOLD:AAG9773
Cyclopidae|BIOZO047-14|Canada|BOLD:AAG9773
Cyclopidae|BIOZO010-14|Canada|BOLD:AAG9773
Cyclopidae|BIOZO020-14|Canada|BOLD:AAG9773
Cyclopidae|BIOAI184-14|Canada|BOLD:AAG9773
Cyclopidae|BIOZO145-14|Canada|BOLD:AAG9773
Cyclopidae|BIOZO148-14|Canada|BOLD:AAG9773
Cyclopidae|OZFWZ290-11|Canada|BOLD:AAG9773
Cyclopidae|BIOAI101-14|Canada|BOLD:AAG9773
Cyclopidae|BIOZO004-14|Canada|BOLD:AAG9773
Cyclopidae|BIOZO026-14|Canada|BOLD:AAG9773
Cyclopidae|OZFWZ315-11|Canada|BOLD:AAG9773
Cyclopidae|OZFWZ301-11|Canada|BOLD:AAG9773
Cyclopidae|NJNAH002-14|Canada|BOLD:AAG9773
Cyclopidae|NJNAH004-14|Canada|BOLD:AAG9773
Cyclopidae|NJNAH001-14|Canada|BOLD:AAG9773
Cyclopidae|NJNAH003-14|Canada|BOLD:AAG9773
Cyclopidae|NJNAH005-14|Canada|BOLD:AAG9773
Diacyclops chakan|BACZP1911-16|Mexico|BOLD:ADC6040
Diacyclops chakan|BACZP1912-16|Mexico|BOLD:ADC6040
Megacyclops|GBCM19633-19|United States|BOLD:AEB2645
Diacyclops albus|ZOOPS354-19|United States|BOLD:ADZ4844
CyclopoidGEN sp.4 CHU|OZFWZ314-11|Canada|BOLD:AAZ0254
CyclopoidGEN sp.5 CHU|OZFWZ312-11|Canada|BOLD:ABA9614
CyclopoidGEN sp.5 CHU|OZFWC419-11|Canada|BOLD:ABA9614
CyclopoidGEN sp.5 CHU|OZFWC467-11|Canada|BOLD:ABA9614
CyclopoidGEN sp.5 CHU|OZFWC470-11|Canada|BOLD:ABA9614
Cyclopoida|GCHAR566-19|Canada|BOLD:AEC6164
Cyclopoida|GCHAR574-19|Canada|BOLD:AEC6164
Cyclopidae|DNARA2509-21|Canada|BOLD:AAG9776
Cyclopidae|GCHAR609-19|Canada|BOLD:AEC1755
Cyclopidae|GCHAR1373-19|Canada|BOLD:AEA4396
Cyclopidae|DNARA949-21|Canada|BOLD:AEA4396
Cyclopidae|DNARA667-21|Canada|BOLD:AEA4396
Cyclopidae|DNARA3355-21|Canada|BOLD:AEA4396
Cyclopidae|DNARA3357-21|Canada|BOLD:AEA4396
Cyclopidae|DNARA935-21|Canada|BOLD:AEA4396
Cyclopidae|DNARA2355-21|Canada|BOLD:AEA4396
Cyclopidae|DNARA2680-21|Canada|BOLD:AEA4396
Cyclopidae|DNARA2361-21|Canada|BOLD:AEA4396
Cyclopidae|GCHAR1150-19|Canada|BOLD:AEA4396
Cyclopidae|DNARA2508-21|Canada|BOLD:AEA4396
Cyclopidae|GCHAR242-19|Canada|BOLD:AEA4396
Cyclopidae|DNARC049-19|Canada|BOLD:AEA4396
Cyclopidae|DNARC050-19|Canada|BOLD:AEA4396
Cyclopidae|DNARC052-19|Canada|BOLD:AEA4396
Cyclopidae|DNARA937-21|Canada|BOLD:AEA4396
Cyclopidae|GCHAR237-19|Canada|BOLD:AEA4396
Cyclopidae|DNARA2359-21|Canada|BOLD:AEA4396
Cyclopidae|DNARA2357-21|Canada|BOLD:AEA4396
Cyclopidae|GCHAR1407-19|Canada|BOLD:AEA4396
Cyclopidae|GCHAR1116-19|Canada|BOLD:AEA4396
Cyclopidae|FCHAR5846-19|Canada|BOLD:AEA4396
Cyclopidae|GCHAR1520-19|Canada|BOLD:AEA4396
Cyclopidae|DNARA934-21|Canada|BOLD:AEA4396
Cyclopidae|DNARC030-19|Canada|BOLD:AEA4396
Cyclopidae|DNARC031-19|Canada|BOLD:AEA4396
Cyclopidae|GCHAR238-19|Canada|BOLD:AEA4396
Cyclopidae|GCHAR1093-19|Canada|BOLD:AEA4396
Cyclopidae|GCHAR1521-19|Canada|BOLD:AEA4396
Cyclopidae|GCHAR1094-19|Canada|BOLD:AEA4396
Cyclopidae|GCHAR1152-19|Canada|BOLD:AEA4396

Cyclopidae|GCHAR1521-19|Canada|BOLD:AEA4396
Cyclopidae|GCHAR1094-19|Canada|BOLD:AEA4396
Cyclopidae|GCHAR1152-19|Canada|BOLD:AEA4396
Cyclopidae|DNARA876-21|Canada|BOLD:AEA4396
Cyclopidae|DNARA879-21|Canada|BOLD:AEA4396
Cyclopidae|DNARA936-21|Canada|BOLD:AEA4396
Cyclopidae|DNARA950-21|Canada|BOLD:AEA4396
Cyclopidae|DNARA2356-21|Canada|BOLD:AEA4396
Cyclopidae|DNARA3356-21|Canada|BOLD:AEA4396
Cyclopidae|DNARA3359-21|Canada|BOLD:AEA4396
Cyclopidae|DNARA3385-21|Canada|BOLD:AEA4396
Cyclopidae|DNARA2155-21|Canada|BOLD:AAG9776
Cyclopidae|DNARA2121-21|Canada|BOLD:AAG9776
Cyclopidae|DNARA2154-21|Canada|BOLD:AAG9776
Cyclopidae|DNARA2156-21|Canada|BOLD:AAG9776
Cyclopidae|GCHAR1173-19|Canada|BOLD:AAG9776
Cyclopidae|GCHAR1138-19|Canada|BOLD:AAG9776
Cyclopidae|DNARA2693-21|Canada|BOLD:AAG9776
Cyclopidae|GCHAR209-19|Canada|BOLD:AAG9776
Cyclopidae|GCHAR1376-19|Canada|BOLD:AAG9776
Cyclopidae|GCHAR1372-19|Canada|BOLD:AAG9776
Cyclopidae|DNARA682-21|Canada|BOLD:AAG9776
Cyclopidae|GCHAR1370-19|Canada|BOLD:AAG9776
Cyclopidae|DNARA3380-21|Canada|BOLD:AAG9776
Cyclopidae|DNARA880-21|Canada|BOLD:AAG9776
Cyclopidae|GCHAR1374-19|Canada|BOLD:AAG9776
Cyclopidae|GCHAR283-19|Canada|BOLD:AAG9776
Cyclopidae|GCHAR282-19|Canada|BOLD:AAG9776
Cyclopidae|GCHAR219-19|Canada|BOLD:AAG9776
Cyclopidae|GCHAR210-19|Canada|BOLD:AAG9776
Cyclopidae|GCHAR208-19|Canada|BOLD:AAG9776
Cyclopidae|GCHAR207-19|Canada|BOLD:AAG9776
Cyclopidae|GCHAR206-19|Canada|BOLD:AAG9776
Cyclopidae|GCHAR205-19|Canada|BOLD:AAG9776
Cyclopidae|GCHAR204-19|Canada|BOLD:AAG9776
Cyclopidae|GCHAR203-19|Canada|BOLD:AAG9776
Cyclopidae|DNARC252-19|Canada|BOLD:AAG9776
Cyclopidae|GJOA3571-21|Canada|BOLD:AAG9776
Cyclopidae|GJOA3574-21|Canada|BOLD:AAG9776
Cyclopidae|DNARA2684-21|Canada|BOLD:AAG9776
Cyclopidae|GCHAR1188-19|Canada|BOLD:AAG9776
Cyclopidae|DNARA2686-21|Canada|BOLD:AAG9776
Cyclopidae|DNARA2687-21|Canada|BOLD:AAG9776
Cyclopidae|DNARA3103-21|Canada|BOLD:AAG9776
Cyclopidae|DNARA2689-21|Canada|BOLD:AAG9776
Cyclopidae|DNARA755-21|Canada|BOLD:AAG9776
Cyclopidae|GCHAR259-19|Canada|BOLD:AAG9776
Cyclopidae|NNMC310-08|Canada|BOLD:AAG9776
Cyclopidae|NNMC363-08|Canada|BOLD:AAG9776
Cyclopidae|NNMC362-08|Canada|BOLD:AAG9776
Cyclopidae|NNMC365-08|Canada|BOLD:AAG9776
Cyclopidae|DNARA2123-21|Canada|BOLD:AAG9776
Cyclopidae|DNARA3104-21|Canada|BOLD:AAG9776
Cyclopidae|DNARA881-21|Canada|BOLD:AAG9776
Cyclopidae|DNARA2112-21|Canada|BOLD:AAG9776
Cyclopidae|DNARC234-19|Canada|BOLD:AAG9776
Cyclopidae|KUGA141-21|Canada|BOLD:AAG9776
Cyclopidae|DNARC610-19|Canada|BOLD:AAG9776
Cyclopidae|GCHAR217-19|Canada|BOLD:AAG9776
Cyclopidae|DNARA2122-21|Canada|BOLD:AAG9776
Cyclopidae|DNARA2348-21|Canada|BOLD:AAG9776
Cyclopidae|DNARA914-21|Canada|BOLD:AAG9776
Cyclopidae|GCHAR1079-19|Canada|BOLD:AAG9776
Cyclopidae|GCHAR1139-19|Canada|BOLD:AAG9776
Cyclopidae|DNARA2481-21|Canada|BOLD:AAG9776
Cyclopidae|DNARA2479-21|Canada|BOLD:AAG9776
Cyclopidae|DNARA2478-21|Canada|BOLD:AAG9776
Cyclopidae|DNARA2477-21|Canada|BOLD:AAG9776
Cyclopidae|DNARA2476-21|Canada|BOLD:AAG9776
Cyclopidae|DNARA2347-21|Canada|BOLD:AAG9776
Cyclopidae|DNARA913-21|Canada|BOLD:AAG9776
Cyclopidae|DNARA882-21|Canada|BOLD:AAG9776
Cyclopidae|DNARA875-21|Canada|BOLD:AAG9776
Cyclopidae|DNARA756-21|Canada|BOLD:AAG9776
Cyclopidae|DNARA754-21|Canada|BOLD:AAG9776
Cyclopidae|DNARA744-21|Canada|BOLD:AAG9776
Cyclopidae|DNARA743-21|Canada|BOLD:AAG9776
Cyclopidae|DNARA742-21|Canada|BOLD:AAG9776
Cyclopidae|DNARA741-21|Canada|BOLD:AAG9776
Cyclopidae|DNARA739-21|Canada|BOLD:AAG9776
Cyclopidae|GCHAR1384-19|Canada|BOLD:AAG9776
Cyclopidae|DNARC612-19|Canada|BOLD:AAG9776
Cyclopidae|DNARC227-19|Canada|BOLD:AAG9776
Cyclopidae|DNARC226-19|Canada|BOLD:AAG9776
Cyclopidae|DNARC078-19|Canada|BOLD:AAG9776
Cyclopidae|DNARC051-19|Canada|BOLD:AAG9776
Cyclopidae|DNARC037-19|Canada|BOLD:AAG9776
Cyclopidae|DNARC029-19|Canada|BOLD:AAG9776
Cyclopidae|DNARC251-19|Canada|BOLD:AAG9776
Cyclopidae|DNARC262-19|Canada|BOLD:AAG9776
Cyclopidae|DNARC264-19|Canada|BOLD:AAG9776
Cyclopidae|DNARC232-19|Canada|BOLD:AAG9776
Cyclopidae|DNARA740-21|Canada|BOLD:AAG9776
Cyclopidae|DNARA931-21|Canada|BOLD:AAG9776
Cyclopidae|DNARA2153-21|Canada|BOLD:AAG9776
Cyclopidae|DNARA2971-21|Canada|BOLD:AAG9776
Cyclopidae|DNARA2480-21|Canada|BOLD:AAG9776
Cyclopidae|DNARA2972-21|Canada|BOLD:AAG9776
Cyclopidae|DNARA3358-21|Canada|BOLD:AAG9776
Cyclopidae|DNARA3381-21|Canada|BOLD:AAG9776
Cyclopidae|DNARA3382-21|Canada|BOLD:AAG9776
Cyclopidae|DNARA3383-21|Canada|BOLD:AAG9776

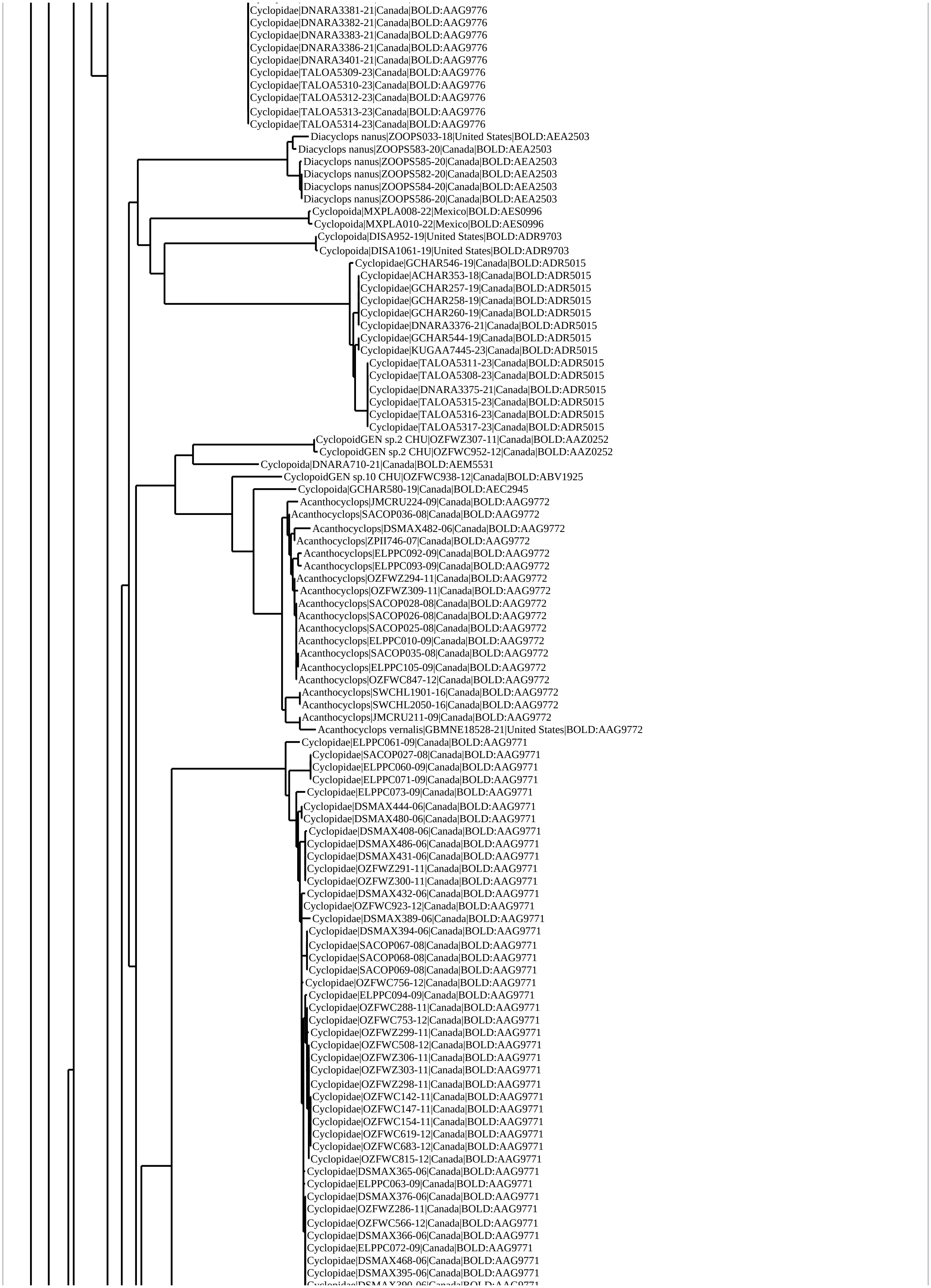
Cyclopidae|DNARA3381-21|Canada|BOLD:AAG9776
Cyclopidae|DNARA3382-21|Canada|BOLD:AAG9776
Cyclopidae|DNARA3383-21|Canada|BOLD:AAG9776
Cyclopidae|DNARA3386-21|Canada|BOLD:AAG9776
Cyclopidae|DNARA3401-21|Canada|BOLD:AAG9776
Cyclopidae|TALOA5309-23|Canada|BOLD:AAG9776
Cyclopidae|TALOA5310-23|Canada|BOLD:AAG9776
Cyclopidae|TALOA5312-23|Canada|BOLD:AAG9776
Cyclopidae|TALOA5313-23|Canada|BOLD:AAG9776
Cyclopidae|TALOA5314-23|Canada|BOLD:AAG9776
Diacyclops nanus|ZOOPS033-18|United States|BOLD:AEA2503
Diacyclops nanus|ZOOPS583-20|Canada|BOLD:AEA2503
Diacyclops nanus|ZOOPS585-20|Canada|BOLD:AEA2503
Diacyclops nanus|ZOOPS582-20|Canada|BOLD:AEA2503
Diacyclops nanus|ZOOPS584-20|Canada|BOLD:AEA2503
Diacyclops nanus|ZOOPS586-20|Canada|BOLD:AEA2503
Cyclopoida|MXPLA008-22|Mexico|BOLD:AES0996
Cyclopoida|MXPLA010-22|Mexico|BOLD:AES0996
Cyclopoida|DISA952-19|United States|BOLD:ADR9703
Cyclopoida|DISA1061-19|United States|BOLD:ADR9703
Cyclopidae|GCHAR546-19|Canada|BOLD:ADR5015
Cyclopidae|ACHAR353-18|Canada|BOLD:ADR5015
Cyclopidae|GCHAR257-19|Canada|BOLD:ADR5015
Cyclopidae|GCHAR258-19|Canada|BOLD:ADR5015
Cyclopidae|GCHAR260-19|Canada|BOLD:ADR5015
Cyclopidae|DNARA3376-21|Canada|BOLD:ADR5015
Cyclopidae|GCHAR544-19|Canada|BOLD:ADR5015
Cyclopidae|KUGAA7445-23|Canada|BOLD:ADR5015
Cyclopidae|TALOA5311-23|Canada|BOLD:ADR5015
Cyclopidae|TALOA5308-23|Canada|BOLD:ADR5015
Cyclopidae|DNARA3375-21|Canada|BOLD:ADR5015
Cyclopidae|TALOA5315-23|Canada|BOLD:ADR5015
Cyclopidae|TALOA5316-23|Canada|BOLD:ADR5015
Cyclopidae|TALOA5317-23|Canada|BOLD:ADR5015
CyclopoidGEN sp.2 CHU|OZFWZ307-11|Canada|BOLD:AAZ0252
CyclopoidGEN sp.2 CHU|OZFWC952-12|Canada|BOLD:AAZ0252
Cyclopoida|DNARA710-21|Canada|BOLD:AEM5531
CyclopoidGEN sp.10 CHU|OZFWC938-12|Canada|BOLD:ABV1925
Cyclopoida|GCHAR580-19|Canada|BOLD:AEC2945
Acanthocyclops|JMCRU224-09|Canada|BOLD:AAG9772
Acanthocyclops|SACOP036-08|Canada|BOLD:AAG9772
Acanthocyclops|DSMAX482-06|Canada|BOLD:AAG9772
Acanthocyclops|ZPII746-07|Canada|BOLD:AAG9772
Acanthocyclops|ELPPC092-09|Canada|BOLD:AAG9772
Acanthocyclops|ELPPC093-09|Canada|BOLD:AAG9772
Acanthocyclops|OZFWZ294-11|Canada|BOLD:AAG9772
Acanthocyclops|OZFWZ309-11|Canada|BOLD:AAG9772
Acanthocyclops|SACOP028-08|Canada|BOLD:AAG9772
Acanthocyclops|SACOP026-08|Canada|BOLD:AAG9772
Acanthocyclops|SACOP025-08|Canada|BOLD:AAG9772
Acanthocyclops|ELPPC010-09|Canada|BOLD:AAG9772
Acanthocyclops|SACOP035-08|Canada|BOLD:AAG9772
Acanthocyclops|ELPPC105-09|Canada|BOLD:AAG9772
Acanthocyclops|OZFWC847-12|Canada|BOLD:AAG9772
Acanthocyclops|SWCHL1901-16|Canada|BOLD:AAG9772
Acanthocyclops|SWCHL2050-16|Canada|BOLD:AAG9772
Acanthocyclops|JMCRU211-09|Canada|BOLD:AAG9772
Acanthocyclops vernalis|GBMNE18528-21|United States|BOLD:AAG9772
Cyclopidae|ELPPC061-09|Canada|BOLD:AAG9771
Cyclopidae|SACOP027-08|Canada|BOLD:AAG9771
Cyclopidae|ELPPC060-09|Canada|BOLD:AAG9771
Cyclopidae|ELPPC071-09|Canada|BOLD:AAG9771
Cyclopidae|ELPPC073-09|Canada|BOLD:AAG9771
Cyclopidae|DSMAX444-06|Canada|BOLD:AAG9771
Cyclopidae|DSMAX480-06|Canada|BOLD:AAG9771
Cyclopidae|DSMAX408-06|Canada|BOLD:AAG9771
Cyclopidae|DSMAX486-06|Canada|BOLD:AAG9771
Cyclopidae|DSMAX431-06|Canada|BOLD:AAG9771
Cyclopidae|OZFWZ291-11|Canada|BOLD:AAG9771
Cyclopidae|OZFWZ300-11|Canada|BOLD:AAG9771
Cyclopidae|DSMAX432-06|Canada|BOLD:AAG9771
Cyclopidae|OZFWC923-12|Canada|BOLD:AAG9771
Cyclopidae|DSMAX389-06|Canada|BOLD:AAG9771
Cyclopidae|DSMAX394-06|Canada|BOLD:AAG9771
Cyclopidae|SACOP067-08|Canada|BOLD:AAG9771
Cyclopidae|SACOP068-08|Canada|BOLD:AAG9771
Cyclopidae|SACOP069-08|Canada|BOLD:AAG9771
Cyclopidae|OZFWC756-12|Canada|BOLD:AAG9771
Cyclopidae|ELPPC094-09|Canada|BOLD:AAG9771
Cyclopidae|OZFWC288-11|Canada|BOLD:AAG9771
Cyclopidae|OZFWC753-12|Canada|BOLD:AAG9771
Cyclopidae|OZFWZ299-11|Canada|BOLD:AAG9771
Cyclopidae|OZFWC508-12|Canada|BOLD:AAG9771
Cyclopidae|OZFWZ306-11|Canada|BOLD:AAG9771
Cyclopidae|OZFWZ303-11|Canada|BOLD:AAG9771
Cyclopidae|OZFWZ298-11|Canada|BOLD:AAG9771
Cyclopidae|OZFWC142-11|Canada|BOLD:AAG9771
Cyclopidae|OZFWC147-11|Canada|BOLD:AAG9771
Cyclopidae|OZFWC154-11|Canada|BOLD:AAG9771
Cyclopidae|OZFWC619-12|Canada|BOLD:AAG9771
Cyclopidae|OZFWC683-12|Canada|BOLD:AAG9771
Cyclopidae|OZFWC815-12|Canada|BOLD:AAG9771
Cyclopidae|DSMAX365-06|Canada|BOLD:AAG9771
Cyclopidae|ELPPC063-09|Canada|BOLD:AAG9771
Cyclopidae|DSMAX376-06|Canada|BOLD:AAG9771
Cyclopidae|OZFWZ286-11|Canada|BOLD:AAG9771
Cyclopidae|OZFWC566-12|Canada|BOLD:AAG9771
Cyclopidae|DSMAX366-06|Canada|BOLD:AAG9771
Cyclopidae|ELPPC072-09|Canada|BOLD:AAG9771
Cyclopidae|DSMAX468-06|Canada|BOLD:AAG9771
Cyclopidae|DSMAX395-06|Canada|BOLD:AAG9771

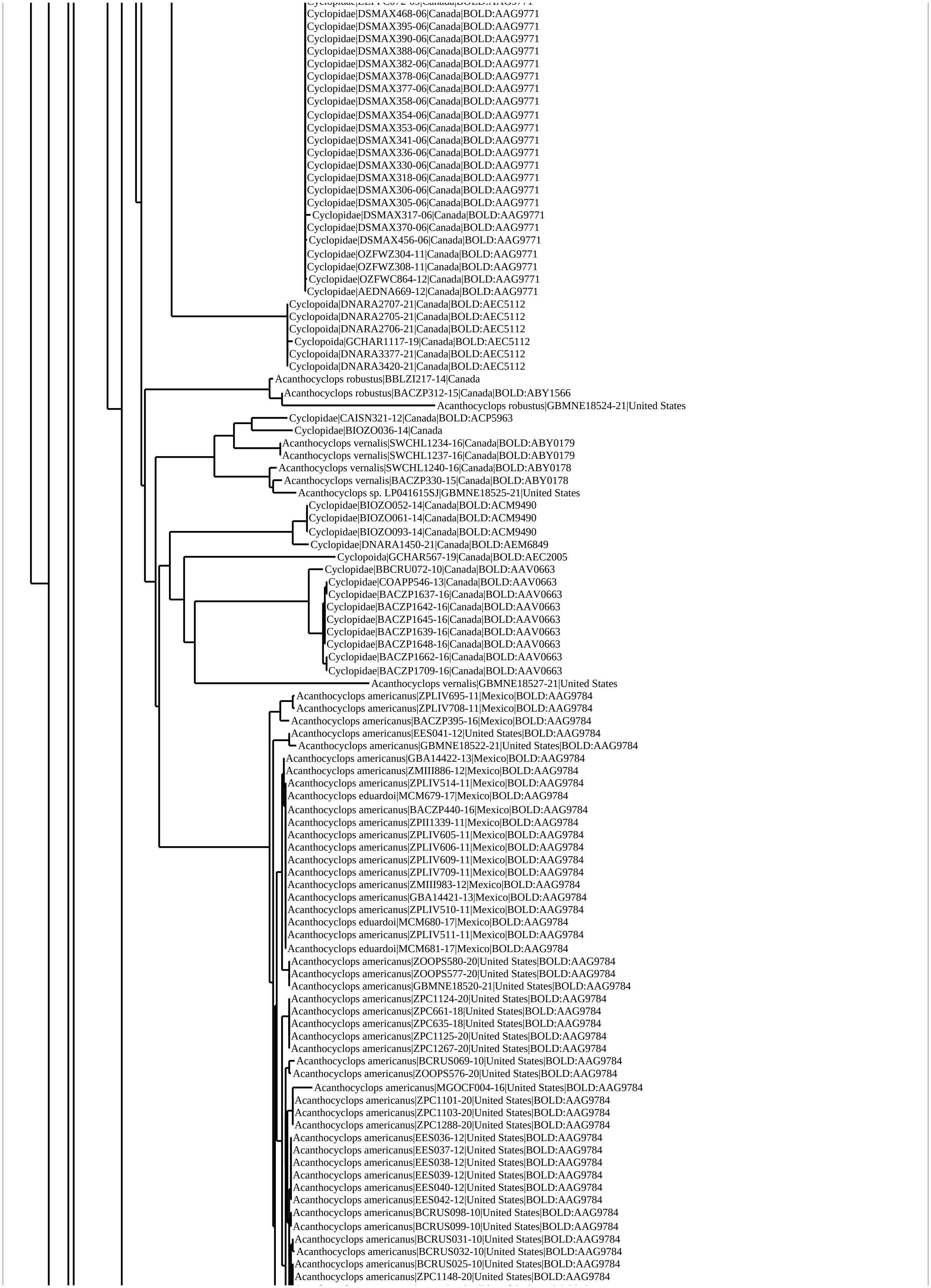
Cyclopidae|DSMAX468-06|Canada|BOLD:AAG9771
Cyclopidae|DSMAX395-06|Canada|BOLD:AAG9771
Cyclopidae|DSMAX390-06|Canada|BOLD:AAG9771
Cyclopidae|DSMAX388-06|Canada|BOLD:AAG9771
Cyclopidae|DSMAX382-06|Canada|BOLD:AAG9771
Cyclopidae|DSMAX378-06|Canada|BOLD:AAG9771
Cyclopidae|DSMAX377-06|Canada|BOLD:AAG9771
Cyclopidae|DSMAX358-06|Canada|BOLD:AAG9771
Cyclopidae|DSMAX354-06|Canada|BOLD:AAG9771
Cyclopidae|DSMAX353-06|Canada|BOLD:AAG9771
Cyclopidae|DSMAX341-06|Canada|BOLD:AAG9771
Cyclopidae|DSMAX336-06|Canada|BOLD:AAG9771
Cyclopidae|DSMAX330-06|Canada|BOLD:AAG9771
Cyclopidae|DSMAX318-06|Canada|BOLD:AAG9771
Cyclopidae|DSMAX306-06|Canada|BOLD:AAG9771
Cyclopidae|DSMAX305-06|Canada|BOLD:AAG9771
Cyclopidae|DSMAX317-06|Canada|BOLD:AAG9771
Cyclopidae|DSMAX370-06|Canada|BOLD:AAG9771
Cyclopidae|DSMAX456-06|Canada|BOLD:AAG9771
Cyclopidae|OZFWZ304-11|Canada|BOLD:AAG9771
Cyclopidae|OZFWZ308-11|Canada|BOLD:AAG9771
Cyclopidae|OZFWC864-12|Canada|BOLD:AAG9771
Cyclopidae|AEDNA669-12|Canada|BOLD:AAG9771
Cyclopoida|DNARA2707-21|Canada|BOLD:AEC5112
Cyclopoida|DNARA2705-21|Canada|BOLD:AEC5112
Cyclopoida|DNARA2706-21|Canada|BOLD:AEC5112
Cyclopoida|GCHAR1117-19|Canada|BOLD:AEC5112
Cyclopoida|DNARA3377-21|Canada|BOLD:AEC5112
Cyclopoida|DNARA3420-21|Canada|BOLD:AEC5112
Acanthocyclops robustus|BBLZI217-14|Canada
Acanthocyclops robustus|BACZP312-15|Canada|BOLD:ABY1566
Acanthocyclops robustus|GBMNE18524-21|United States
Cyclopidae|CAISN321-12|Canada|BOLD:ACP5963
Cyclopidae|BIOZO036-14|Canada
Acanthocyclops vernalis|SWCHL1234-16|Canada|BOLD:ABY0179
Acanthocyclops vernalis|SWCHL1237-16|Canada|BOLD:ABY0179
Acanthocyclops vernalis|SWCHL1240-16|Canada|BOLD:ABY0178
Acanthocyclops vernalis|BACZP330-15|Canada|BOLD:ABY0178
Acanthocyclops sp. LP041615SJ|GBMNE18525-21|United States
Cyclopidae|BIOZO052-14|Canada|BOLD:ACM9490
Cyclopidae|BIOZO061-14|Canada|BOLD:ACM9490
Cyclopidae|BIOZO093-14|Canada|BOLD:ACM9490
Cyclopidae|DNARA1450-21|Canada|BOLD:AEM6849
Cyclopoida|GCHAR567-19|Canada|BOLD:AEC2005
Cyclopidae|BBCRU072-10|Canada|BOLD:AAV0663
Cyclopidae|COAPP546-13|Canada|BOLD:AAV0663
Cyclopidae|BACZP1637-16|Canada|BOLD:AAV0663
Cyclopidae|BACZP1642-16|Canada|BOLD:AAV0663
Cyclopidae|BACZP1645-16|Canada|BOLD:AAV0663
Cyclopidae|BACZP1639-16|Canada|BOLD:AAV0663
Cyclopidae|BACZP1648-16|Canada|BOLD:AAV0663
Cyclopidae|BACZP1662-16|Canada|BOLD:AAV0663
Cyclopidae|BACZP1709-16|Canada|BOLD:AAV0663
Acanthocyclops vernalis|GBMNE18527-21|United States
Acanthocyclops americanus|ZPLIV695-11|Mexico|BOLD:AAG9784
Acanthocyclops americanus|ZPLIV708-11|Mexico|BOLD:AAG9784
Acanthocyclops americanus|BACZP395-16|Mexico|BOLD:AAG9784
Acanthocyclops americanus|EES041-12|United States|BOLD:AAG9784
Acanthocyclops americanus|GBMNE18522-21|United States|BOLD:AAG9784
Acanthocyclops americanus|GBA14422-13|Mexico|BOLD:AAG9784
Acanthocyclops americanus|ZMIII886-12|Mexico|BOLD:AAG9784
Acanthocyclops americanus|ZPLIV514-11|Mexico|BOLD:AAG9784
Acanthocyclops eduardoi|MCM679-17|Mexico|BOLD:AAG9784
Acanthocyclops americanus|BACZP440-16|Mexico|BOLD:AAG9784
Acanthocyclops americanus|ZPII1339-11|Mexico|BOLD:AAG9784
Acanthocyclops americanus|ZPLIV605-11|Mexico|BOLD:AAG9784
Acanthocyclops americanus|ZPLIV606-11|Mexico|BOLD:AAG9784
Acanthocyclops americanus|ZPLIV609-11|Mexico|BOLD:AAG9784
Acanthocyclops americanus|ZPLIV709-11|Mexico|BOLD:AAG9784
Acanthocyclops americanus|ZMIII983-12|Mexico|BOLD:AAG9784
Acanthocyclops americanus|GBA14421-13|Mexico|BOLD:AAG9784
Acanthocyclops americanus|ZPLIV510-11|Mexico|BOLD:AAG9784
Acanthocyclops eduardoi|MCM680-17|Mexico|BOLD:AAG9784
Acanthocyclops americanus|ZPLIV511-11|Mexico|BOLD:AAG9784
Acanthocyclops eduardoi|MCM681-17|Mexico|BOLD:AAG9784
Acanthocyclops americanus|ZOOPS580-20|United States|BOLD:AAG9784
Acanthocyclops americanus|ZOOPS577-20|United States|BOLD:AAG9784
Acanthocyclops americanus|GBMNE18520-21|United States|BOLD:AAG9784
Acanthocyclops americanus|ZPC1124-20|United States|BOLD:AAG9784
Acanthocyclops americanus|ZPC661-18|United States|BOLD:AAG9784
Acanthocyclops americanus|ZPC635-18|United States|BOLD:AAG9784
Acanthocyclops americanus|ZPC1125-20|United States|BOLD:AAG9784
Acanthocyclops americanus|ZPC1267-20|United States|BOLD:AAG9784
Acanthocyclops americanus|BCRUS069-10|United States|BOLD:AAG9784
Acanthocyclops americanus|ZOOPS576-20|United States|BOLD:AAG9784
Acanthocyclops americanus|MGOCF004-16|United States|BOLD:AAG9784
Acanthocyclops americanus|ZPC1101-20|United States|BOLD:AAG9784
Acanthocyclops americanus|ZPC1103-20|United States|BOLD:AAG9784
Acanthocyclops americanus|ZPC1288-20|United States|BOLD:AAG9784
Acanthocyclops americanus|EES036-12|United States|BOLD:AAG9784
Acanthocyclops americanus|EES037-12|United States|BOLD:AAG9784
Acanthocyclops americanus|EES038-12|United States|BOLD:AAG9784
Acanthocyclops americanus|EES039-12|United States|BOLD:AAG9784
Acanthocyclops americanus|EES040-12|United States|BOLD:AAG9784
Acanthocyclops americanus|EES042-12|United States|BOLD:AAG9784
Acanthocyclops americanus|BCRUS098-10|United States|BOLD:AAG9784
Acanthocyclops americanus|BCRUS099-10|United States|BOLD:AAG9784
Acanthocyclops americanus|BCRUS031-10|United States|BOLD:AAG9784
Acanthocyclops americanus|BCRUS032-10|United States|BOLD:AAG9784
Acanthocyclops americanus|BCRUS025-10|United States|BOLD:AAG9784
Acanthocyclops americanus|ZPC1148-20|United States|BOLD:AAG9784

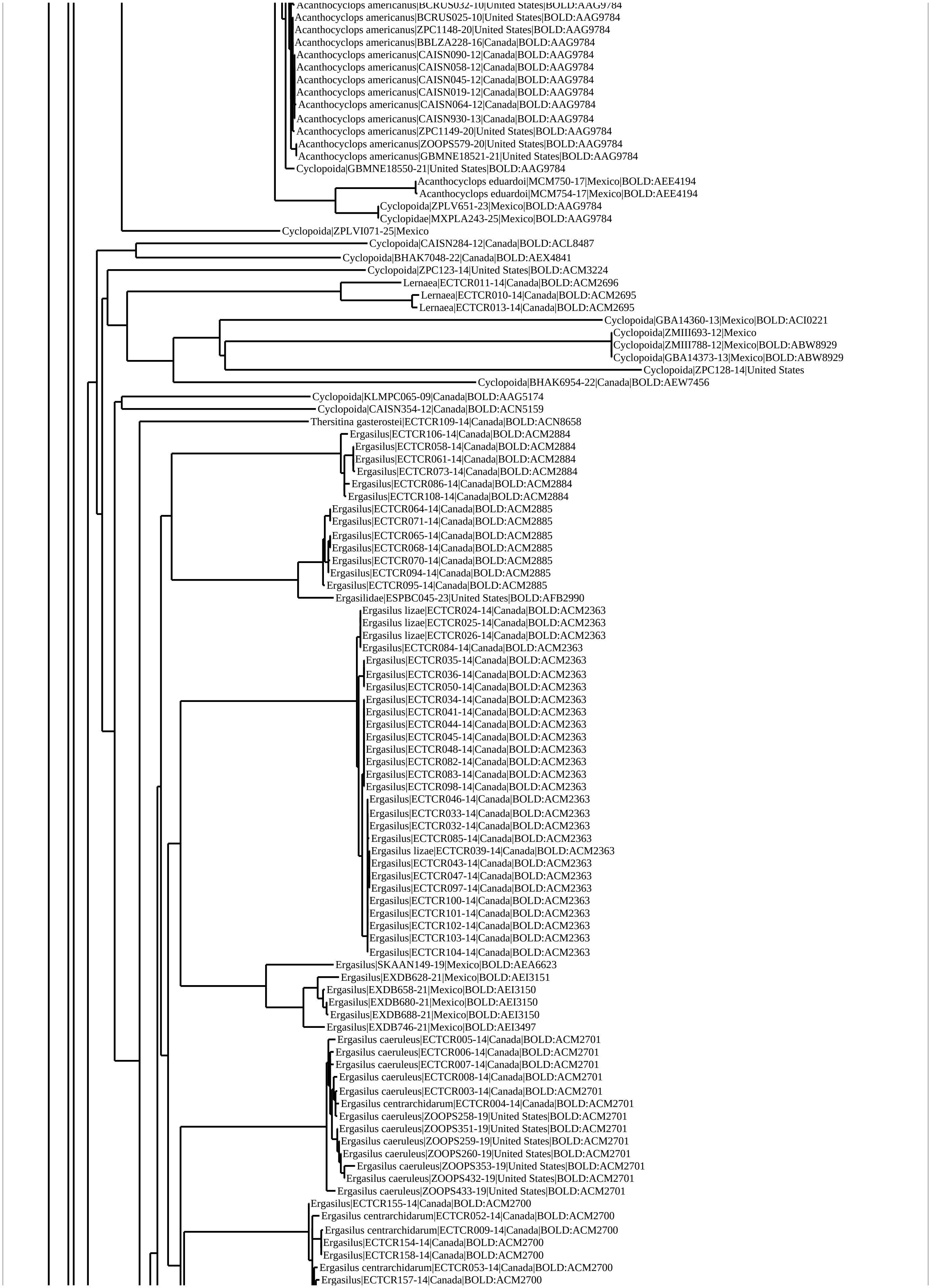
Acanthocyclops americanus|BCRUS032-10|United States|BOLD:AAG9784
Acanthocyclops americanus|BCRUS025-10|United States|BOLD:AAG9784
Acanthocyclops americanus|ZPC1148-20|United States|BOLD:AAG9784
Acanthocyclops americanus|BBLZA228-16|Canada|BOLD:AAG9784
Acanthocyclops americanus|CAISN090-12|Canada|BOLD:AAG9784
Acanthocyclops americanus|CAISN058-12|Canada|BOLD:AAG9784
Acanthocyclops americanus|CAISN045-12|Canada|BOLD:AAG9784
Acanthocyclops americanus|CAISN019-12|Canada|BOLD:AAG9784
Acanthocyclops americanus|CAISN064-12|Canada|BOLD:AAG9784
Acanthocyclops americanus|CAISN930-13|Canada|BOLD:AAG9784
Acanthocyclops americanus|ZPC1149-20|United States|BOLD:AAG9784
Acanthocyclops americanus|ZOOPS579-20|United States|BOLD:AAG9784
Acanthocyclops americanus|GBMNE18521-21|United States|BOLD:AAG9784
Cyclopoida|GBMNE18550-21|United States|BOLD:AAG9784
Acanthocyclops eduardoi|MCM750-17|Mexico|BOLD:AEE4194
Acanthocyclops eduardoi|MCM754-17|Mexico|BOLD:AEE4194
Cyclopoida|ZPLV651-23|Mexico|BOLD:AAG9784
Cyclopidae|MXPLA243-25|Mexico|BOLD:AAG9784
Cyclopoida|ZPLVI071-25|Mexico
Cyclopoida|CAISN284-12|Canada|BOLD:ACL8487
Cyclopoida|BHAK7048-22|Canada|BOLD:AEX4841
Cyclopoida|ZPC123-14|United States|BOLD:ACM3224
Lernaea|ECTCR011-14|Canada|BOLD:ACM2696
Lernaea|ECTCR010-14|Canada|BOLD:ACM2695
Lernaea|ECTCR013-14|Canada|BOLD:ACM2695
Cyclopoida|GBA14360-13|Mexico|BOLD:ACI0221
Cyclopoida|ZMIII693-12|Mexico
Cyclopoida|ZMIII788-12|Mexico|BOLD:ABW8929
Cyclopoida|GBA14373-13|Mexico|BOLD:ABW8929
Cyclopoida|ZPC128-14|United States
Cyclopoida|BHAK6954-22|Canada|BOLD:AEW7456
Cyclopoida|KLMPC065-09|Canada|BOLD:AAG5174
Cyclopoida|CAISN354-12|Canada|BOLD:ACN5159
Thersitina gasterostei|ECTCR109-14|Canada|BOLD:ACN8658
Ergasilus|ECTCR106-14|Canada|BOLD:ACM2884
Ergasilus|ECTCR058-14|Canada|BOLD:ACM2884
Ergasilus|ECTCR061-14|Canada|BOLD:ACM2884
Ergasilus|ECTCR073-14|Canada|BOLD:ACM2884
Ergasilus|ECTCR086-14|Canada|BOLD:ACM2884
Ergasilus|ECTCR108-14|Canada|BOLD:ACM2884
Ergasilus|ECTCR064-14|Canada|BOLD:ACM2885
Ergasilus|ECTCR071-14|Canada|BOLD:ACM2885
Ergasilus|ECTCR065-14|Canada|BOLD:ACM2885
Ergasilus|ECTCR068-14|Canada|BOLD:ACM2885
Ergasilus|ECTCR070-14|Canada|BOLD:ACM2885
Ergasilus|ECTCR094-14|Canada|BOLD:ACM2885
Ergasilus|ECTCR095-14|Canada|BOLD:ACM2885
Ergasilidae|ESPBC045-23|United States|BOLD:AFB2990
Ergasilus lizae|ECTCR024-14|Canada|BOLD:ACM2363
Ergasilus lizae|ECTCR025-14|Canada|BOLD:ACM2363
Ergasilus lizae|ECTCR026-14|Canada|BOLD:ACM2363
Ergasilus|ECTCR084-14|Canada|BOLD:ACM2363
Ergasilus|ECTCR035-14|Canada|BOLD:ACM2363
Ergasilus|ECTCR036-14|Canada|BOLD:ACM2363
Ergasilus|ECTCR050-14|Canada|BOLD:ACM2363
Ergasilus|ECTCR034-14|Canada|BOLD:ACM2363
Ergasilus|ECTCR041-14|Canada|BOLD:ACM2363
Ergasilus|ECTCR044-14|Canada|BOLD:ACM2363
Ergasilus|ECTCR045-14|Canada|BOLD:ACM2363
Ergasilus|ECTCR048-14|Canada|BOLD:ACM2363
Ergasilus|ECTCR082-14|Canada|BOLD:ACM2363
Ergasilus|ECTCR083-14|Canada|BOLD:ACM2363
Ergasilus|ECTCR098-14|Canada|BOLD:ACM2363
Ergasilus|ECTCR046-14|Canada|BOLD:ACM2363
Ergasilus|ECTCR033-14|Canada|BOLD:ACM2363
Ergasilus|ECTCR032-14|Canada|BOLD:ACM2363
Ergasilus|ECTCR085-14|Canada|BOLD:ACM2363
Ergasilus lizae|ECTCR039-14|Canada|BOLD:ACM2363
Ergasilus|ECTCR043-14|Canada|BOLD:ACM2363
Ergasilus|ECTCR047-14|Canada|BOLD:ACM2363
Ergasilus|ECTCR097-14|Canada|BOLD:ACM2363
Ergasilus|ECTCR100-14|Canada|BOLD:ACM2363
Ergasilus|ECTCR101-14|Canada|BOLD:ACM2363
Ergasilus|ECTCR102-14|Canada|BOLD:ACM2363
Ergasilus|ECTCR103-14|Canada|BOLD:ACM2363
Ergasilus|ECTCR104-14|Canada|BOLD:ACM2363
Ergasilus|SKAAN149-19|Mexico|BOLD:AEA6623
Ergasilus|EXDB628-21|Mexico|BOLD:AEI3151
Ergasilus|EXDB658-21|Mexico|BOLD:AEI3150
Ergasilus|EXDB680-21|Mexico|BOLD:AEI3150
Ergasilus|EXDB688-21|Mexico|BOLD:AEI3150
Ergasilus|EXDB746-21|Mexico|BOLD:AEI3497
Ergasilus caeruleus|ECTCR005-14|Canada|BOLD:ACM2701
Ergasilus caeruleus|ECTCR006-14|Canada|BOLD:ACM2701
Ergasilus caeruleus|ECTCR007-14|Canada|BOLD:ACM2701
Ergasilus caeruleus|ECTCR008-14|Canada|BOLD:ACM2701
Ergasilus caeruleus|ECTCR003-14|Canada|BOLD:ACM2701
Ergasilus centrarchidarum|ECTCR004-14|Canada|BOLD:ACM2701
Ergasilus caeruleus|ZOOPS258-19|United States|BOLD:ACM2701
Ergasilus caeruleus|ZOOPS351-19|United States|BOLD:ACM2701
Ergasilus caeruleus|ZOOPS259-19|United States|BOLD:ACM2701
Ergasilus caeruleus|ZOOPS260-19|United States|BOLD:ACM2701
Ergasilus caeruleus|ZOOPS353-19|United States|BOLD:ACM2701
Ergasilus caeruleus|ZOOPS432-19|United States|BOLD:ACM2701
Ergasilus caeruleus|ZOOPS433-19|United States|BOLD:ACM2701
Ergasilus|ECTCR155-14|Canada|BOLD:ACM2700
Ergasilus centrarchidarum|ECTCR052-14|Canada|BOLD:ACM2700
Ergasilus centrarchidarum|ECTCR009-14|Canada|BOLD:ACM2700
Ergasilus|ECTCR154-14|Canada|BOLD:ACM2700
Ergasilus|ECTCR158-14|Canada|BOLD:ACM2700
Ergasilus centrarchidarum|ECTCR053-14|Canada|BOLD:ACM2700
Ergasilus|ECTCR157-14|Canada|BOLD:ACM2700

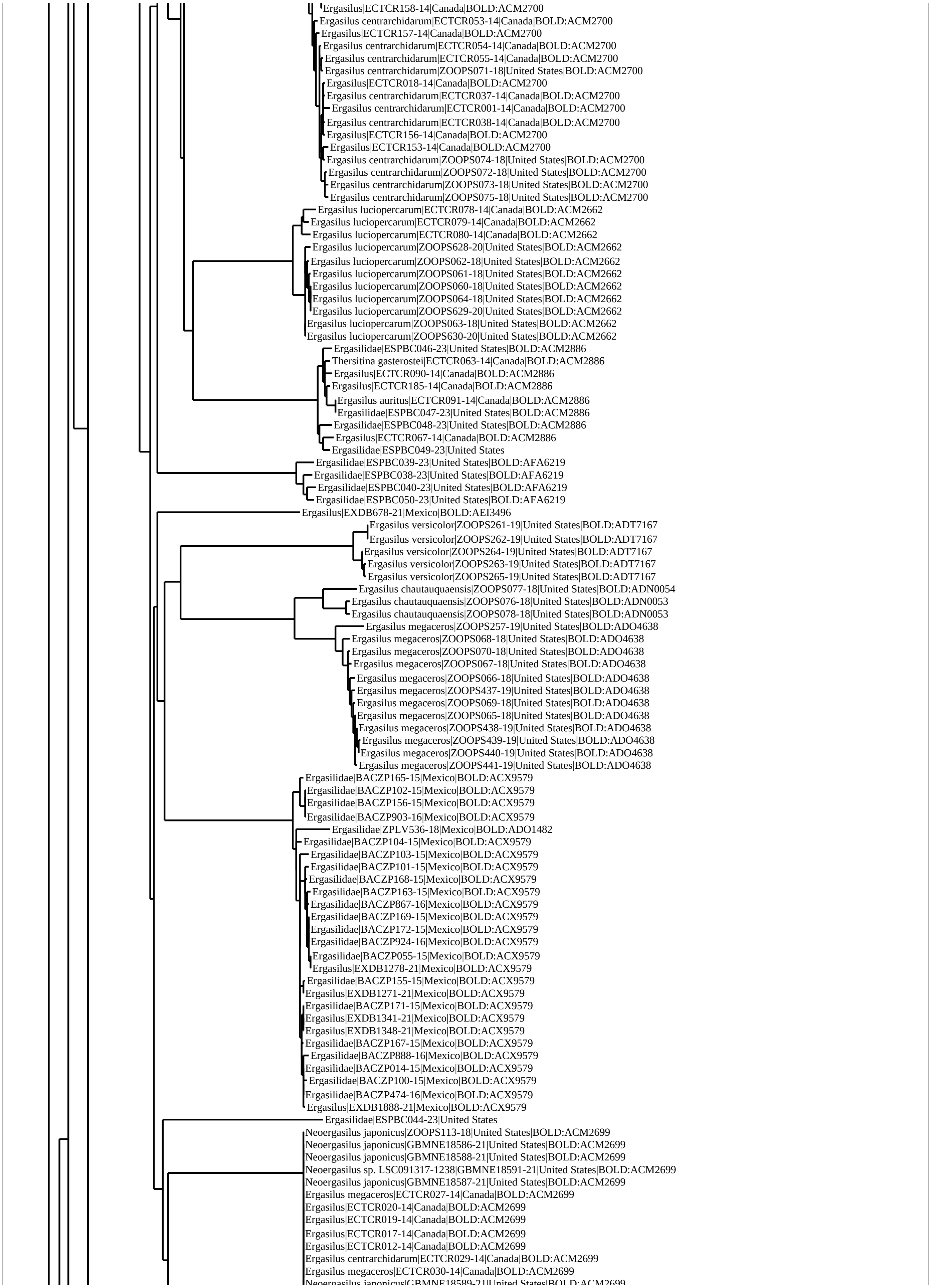
Ergasilus|ECTCR158-14|Canada|BOLD:ACM2700
Ergasilus centrarchidarum|ECTCR053-14|Canada|BOLD:ACM2700
Ergasilus|ECTCR157-14|Canada|BOLD:ACM2700
Ergasilus centrarchidarum|ECTCR054-14|Canada|BOLD:ACM2700
Ergasilus centrarchidarum|ECTCR055-14|Canada|BOLD:ACM2700
Ergasilus centrarchidarum|ZOOPS071-18|United States|BOLD:ACM2700
Ergasilus|ECTCR018-14|Canada|BOLD:ACM2700
Ergasilus centrarchidarum|ECTCR037-14|Canada|BOLD:ACM2700
Ergasilus centrarchidarum|ECTCR001-14|Canada|BOLD:ACM2700
Ergasilus centrarchidarum|ECTCR038-14|Canada|BOLD:ACM2700
Ergasilus|ECTCR156-14|Canada|BOLD:ACM2700
Ergasilus|ECTCR153-14|Canada|BOLD:ACM2700
Ergasilus centrarchidarum|ZOOPS074-18|United States|BOLD:ACM2700
Ergasilus centrarchidarum|ZOOPS072-18|United States|BOLD:ACM2700
Ergasilus centrarchidarum|ZOOPS073-18|United States|BOLD:ACM2700
Ergasilus centrarchidarum|ZOOPS075-18|United States|BOLD:ACM2700
Ergasilus luciopercarum|ECTCR078-14|Canada|BOLD:ACM2662
Ergasilus luciopercarum|ECTCR079-14|Canada|BOLD:ACM2662
Ergasilus luciopercarum|ECTCR080-14|Canada|BOLD:ACM2662
Ergasilus luciopercarum|ZOOPS628-20|United States|BOLD:ACM2662
Ergasilus luciopercarum|ZOOPS062-18|United States|BOLD:ACM2662
Ergasilus luciopercarum|ZOOPS061-18|United States|BOLD:ACM2662
Ergasilus luciopercarum|ZOOPS060-18|United States|BOLD:ACM2662
Ergasilus luciopercarum|ZOOPS064-18|United States|BOLD:ACM2662
Ergasilus luciopercarum|ZOOPS629-20|United States|BOLD:ACM2662
Ergasilus luciopercarum|ZOOPS063-18|United States|BOLD:ACM2662
Ergasilus luciopercarum|ZOOPS630-20|United States|BOLD:ACM2662
Ergasilidae|ESPBC046-23|United States|BOLD:ACM2886
Thersitina gasterostei|ECTCR063-14|Canada|BOLD:ACM2886
Ergasilus|ECTCR090-14|Canada|BOLD:ACM2886
Ergasilus|ECTCR185-14|Canada|BOLD:ACM2886
Ergasilus auritus|ECTCR091-14|Canada|BOLD:ACM2886
Ergasilidae|ESPBC047-23|United States|BOLD:ACM2886
Ergasilidae|ESPBC048-23|United States|BOLD:ACM2886
Ergasilus|ECTCR067-14|Canada|BOLD:ACM2886
Ergasilidae|ESPBC049-23|United States
Ergasilidae|ESPBC039-23|United States|BOLD:AFA6219
Ergasilidae|ESPBC038-23|United States|BOLD:AFA6219
Ergasilidae|ESPBC040-23|United States|BOLD:AFA6219
Ergasilidae|ESPBC050-23|United States|BOLD:AFA6219
Ergasilus|EXDB678-21|Mexico|BOLD:AEI3496
Ergasilus versicolor|ZOOPS261-19|United States|BOLD:ADT7167
Ergasilus versicolor|ZOOPS262-19|United States|BOLD:ADT7167
Ergasilus versicolor|ZOOPS264-19|United States|BOLD:ADT7167
Ergasilus versicolor|ZOOPS263-19|United States|BOLD:ADT7167
Ergasilus versicolor|ZOOPS265-19|United States|BOLD:ADT7167
Ergasilus chautauquaensis|ZOOPS077-18|United States|BOLD:ADN0054
Ergasilus chautauquaensis|ZOOPS076-18|United States|BOLD:ADN0053
Ergasilus chautauquaensis|ZOOPS078-18|United States|BOLD:ADN0053
Ergasilus megaceros|ZOOPS257-19|United States|BOLD:ADO4638
Ergasilus megaceros|ZOOPS068-18|United States|BOLD:ADO4638
Ergasilus megaceros|ZOOPS070-18|United States|BOLD:ADO4638
Ergasilus megaceros|ZOOPS067-18|United States|BOLD:ADO4638
Ergasilus megaceros|ZOOPS066-18|United States|BOLD:ADO4638
Ergasilus megaceros|ZOOPS437-19|United States|BOLD:ADO4638
Ergasilus megaceros|ZOOPS069-18|United States|BOLD:ADO4638
Ergasilus megaceros|ZOOPS065-18|United States|BOLD:ADO4638
Ergasilus megaceros|ZOOPS438-19|United States|BOLD:ADO4638
Ergasilus megaceros|ZOOPS439-19|United States|BOLD:ADO4638
Ergasilus megaceros|ZOOPS440-19|United States|BOLD:ADO4638
Ergasilus megaceros|ZOOPS441-19|United States|BOLD:ADO4638
Ergasilidae|BACZP165-15|Mexico|BOLD:ACX9579
Ergasilidae|BACZP102-15|Mexico|BOLD:ACX9579
Ergasilidae|BACZP156-15|Mexico|BOLD:ACX9579
Ergasilidae|BACZP903-16|Mexico|BOLD:ACX9579
Ergasilidae|ZPLV536-18|Mexico|BOLD:ADO1482
Ergasilidae|BACZP104-15|Mexico|BOLD:ACX9579
Ergasilidae|BACZP103-15|Mexico|BOLD:ACX9579
Ergasilidae|BACZP101-15|Mexico|BOLD:ACX9579
Ergasilidae|BACZP168-15|Mexico|BOLD:ACX9579
Ergasilidae|BACZP163-15|Mexico|BOLD:ACX9579
Ergasilidae|BACZP867-16|Mexico|BOLD:ACX9579
Ergasilidae|BACZP169-15|Mexico|BOLD:ACX9579
Ergasilidae|BACZP172-15|Mexico|BOLD:ACX9579
Ergasilidae|BACZP924-16|Mexico|BOLD:ACX9579
Ergasilidae|BACZP055-15|Mexico|BOLD:ACX9579
Ergasilus|EXDB1278-21|Mexico|BOLD:ACX9579
Ergasilidae|BACZP155-15|Mexico|BOLD:ACX9579
Ergasilus|EXDB1271-21|Mexico|BOLD:ACX9579
Ergasilidae|BACZP171-15|Mexico|BOLD:ACX9579
Ergasilus|EXDB1341-21|Mexico|BOLD:ACX9579
Ergasilus|EXDB1348-21|Mexico|BOLD:ACX9579
Ergasilidae|BACZP167-15|Mexico|BOLD:ACX9579
Ergasilidae|BACZP888-16|Mexico|BOLD:ACX9579
Ergasilidae|BACZP014-15|Mexico|BOLD:ACX9579
Ergasilidae|BACZP100-15|Mexico|BOLD:ACX9579
Ergasilidae|BACZP474-16|Mexico|BOLD:ACX9579
Ergasilus|EXDB1888-21|Mexico|BOLD:ACX9579
Ergasilidae|ESPBC044-23|United States
Neoergasilus japonicus|ZOOPS113-18|United States|BOLD:ACM2699
Neoergasilus japonicus|GBMNE18586-21|United States|BOLD:ACM2699
Neoergasilus japonicus|GBMNE18588-21|United States|BOLD:ACM2699
Neoergasilus sp. LSC091317-1238|GBMNE18591-21|United States|BOLD:ACM2699
Neoergasilus japonicus|GBMNE18587-21|United States|BOLD:ACM2699
Ergasilus megaceros|ECTCR027-14|Canada|BOLD:ACM2699
Ergasilus|ECTCR020-14|Canada|BOLD:ACM2699
Ergasilus|ECTCR019-14|Canada|BOLD:ACM2699
Ergasilus|ECTCR017-14|Canada|BOLD:ACM2699
Ergasilus|ECTCR012-14|Canada|BOLD:ACM2699
Ergasilus centrarchidarum|ECTCR029-14|Canada|BOLD:ACM2699
Ergasilus megaceros|ECTCR030-14|Canada|BOLD:ACM2699
Neoergasilus japonicus|GBMNE18589-21|United States|BOLD:ACM2699

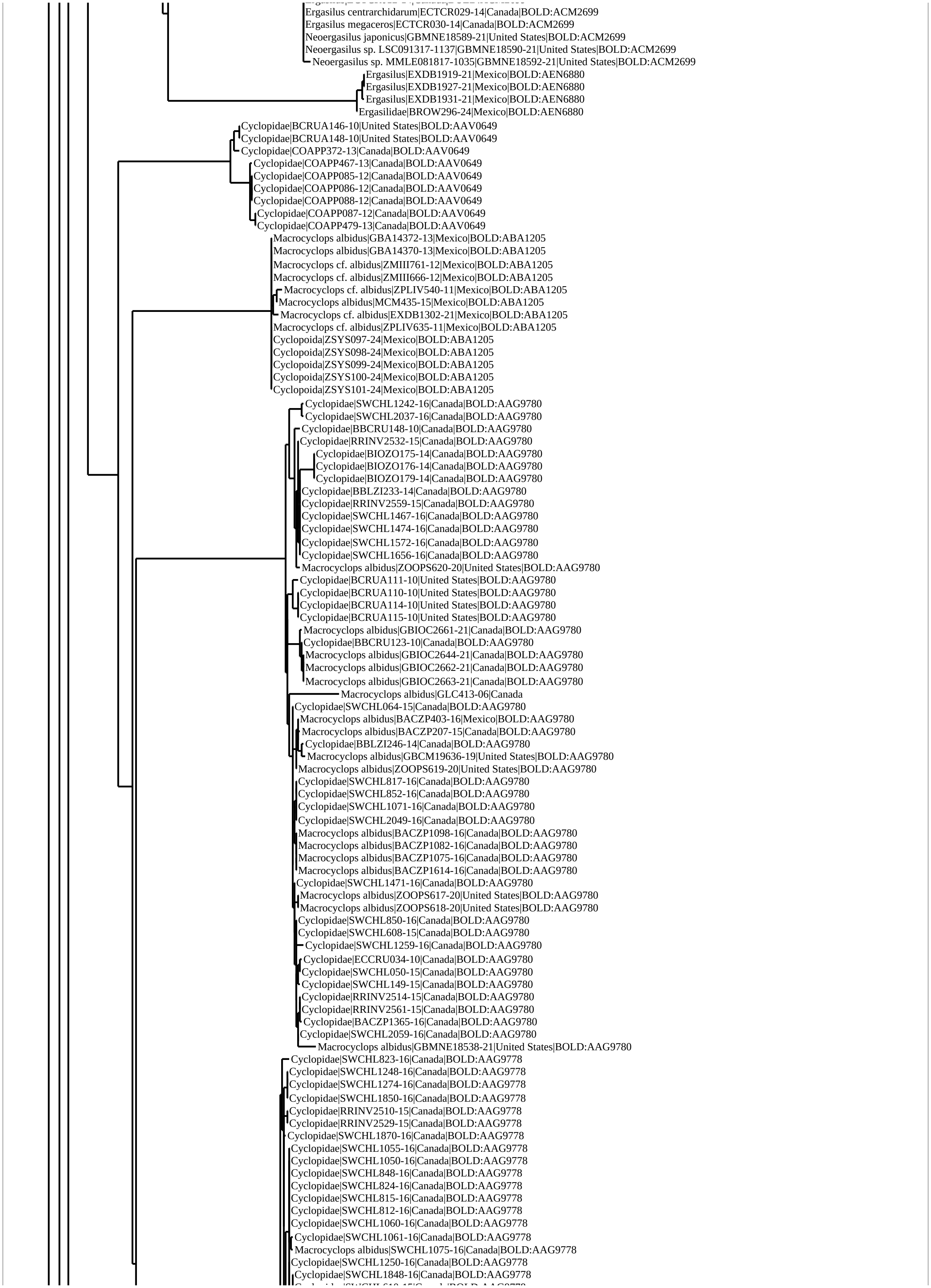
Ergasilus centrarchidarum|ECTCR029-14|Canada|BOLD:ACM2699
Ergasilus megaceros|ECTCR030-14|Canada|BOLD:ACM2699
Neoergasilus japonicus|GBMNE18589-21|United States|BOLD:ACM2699
Neoergasilus sp. LSC091317-1137|GBMNE18590-21|United States|BOLD:ACM2699
Neoergasilus sp. MMLE081817-1035|GBMNE18592-21|United States|BOLD:ACM2699
Ergasilus|EXDB1919-21|Mexico|BOLD:AEN6880
Ergasilus|EXDB1927-21|Mexico|BOLD:AEN6880
Ergasilus|EXDB1931-21|Mexico|BOLD:AEN6880
Ergasilidae|BROW296-24|Mexico|BOLD:AEN6880
Cyclopidae|BCRUA146-10|United States|BOLD:AAV0649
Cyclopidae|BCRUA148-10|United States|BOLD:AAV0649
Cyclopidae|COAPP372-13|Canada|BOLD:AAV0649
Cyclopidae|COAPP467-13|Canada|BOLD:AAV0649
Cyclopidae|COAPP085-12|Canada|BOLD:AAV0649
Cyclopidae|COAPP086-12|Canada|BOLD:AAV0649
Cyclopidae|COAPP088-12|Canada|BOLD:AAV0649
Cyclopidae|COAPP087-12|Canada|BOLD:AAV0649
Cyclopidae|COAPP479-13|Canada|BOLD:AAV0649
Macrocyclops albidus|GBA14372-13|Mexico|BOLD:ABA1205
Macrocyclops albidus|GBA14370-13|Mexico|BOLD:ABA1205
Macrocyclops cf. albidus|ZMIII761-12|Mexico|BOLD:ABA1205
Macrocyclops cf. albidus|ZMIII666-12|Mexico|BOLD:ABA1205
Macrocyclops cf. albidus|ZPLIV540-11|Mexico|BOLD:ABA1205
Macrocyclops albidus|MCM435-15|Mexico|BOLD:ABA1205
Macrocyclops cf. albidus|EXDB1302-21|Mexico|BOLD:ABA1205
Macrocyclops cf. albidus|ZPLIV635-11|Mexico|BOLD:ABA1205
Cyclopoida|ZSYS097-24|Mexico|BOLD:ABA1205
Cyclopoida|ZSYS098-24|Mexico|BOLD:ABA1205
Cyclopoida|ZSYS099-24|Mexico|BOLD:ABA1205
Cyclopoida|ZSYS100-24|Mexico|BOLD:ABA1205
Cyclopoida|ZSYS101-24|Mexico|BOLD:ABA1205
Cyclopidae|SWCHL1242-16|Canada|BOLD:AAG9780
Cyclopidae|SWCHL2037-16|Canada|BOLD:AAG9780
Cyclopidae|BBCRU148-10|Canada|BOLD:AAG9780
Cyclopidae|RRINV2532-15|Canada|BOLD:AAG9780
Cyclopidae|BIOZO175-14|Canada|BOLD:AAG9780
Cyclopidae|BIOZO176-14|Canada|BOLD:AAG9780
Cyclopidae|BIOZO179-14|Canada|BOLD:AAG9780
Cyclopidae|BBLZI233-14|Canada|BOLD:AAG9780
Cyclopidae|RRINV2559-15|Canada|BOLD:AAG9780
Cyclopidae|SWCHL1467-16|Canada|BOLD:AAG9780
Cyclopidae|SWCHL1474-16|Canada|BOLD:AAG9780
Cyclopidae|SWCHL1572-16|Canada|BOLD:AAG9780
Cyclopidae|SWCHL1656-16|Canada|BOLD:AAG9780
Macrocyclops albidus|ZOOPS620-20|United States|BOLD:AAG9780
Cyclopidae|BCRUA111-10|United States|BOLD:AAG9780
Cyclopidae|BCRUA110-10|United States|BOLD:AAG9780
Cyclopidae|BCRUA114-10|United States|BOLD:AAG9780
Cyclopidae|BCRUA115-10|United States|BOLD:AAG9780
Macrocyclops albidus|GBIOC2661-21|Canada|BOLD:AAG9780
Cyclopidae|BBCRU123-10|Canada|BOLD:AAG9780
Macrocyclops albidus|GBIOC2644-21|Canada|BOLD:AAG9780
Macrocyclops albidus|GBIOC2662-21|Canada|BOLD:AAG9780
Macrocyclops albidus|GBIOC2663-21|Canada|BOLD:AAG9780
Macrocyclops albidus|GLC413-06|Canada
Cyclopidae|SWCHL064-15|Canada|BOLD:AAG9780
Macrocyclops albidus|BACZP403-16|Mexico|BOLD:AAG9780
Macrocyclops albidus|BACZP207-15|Canada|BOLD:AAG9780
Cyclopidae|BBLZI246-14|Canada|BOLD:AAG9780
Macrocyclops albidus|GBCM19636-19|United States|BOLD:AAG9780
Macrocyclops albidus|ZOOPS619-20|United States|BOLD:AAG9780
Cyclopidae|SWCHL817-16|Canada|BOLD:AAG9780
Cyclopidae|SWCHL852-16|Canada|BOLD:AAG9780
Cyclopidae|SWCHL1071-16|Canada|BOLD:AAG9780
Cyclopidae|SWCHL2049-16|Canada|BOLD:AAG9780
Macrocyclops albidus|BACZP1098-16|Canada|BOLD:AAG9780
Macrocyclops albidus|BACZP1082-16|Canada|BOLD:AAG9780
Macrocyclops albidus|BACZP1075-16|Canada|BOLD:AAG9780
Macrocyclops albidus|BACZP1614-16|Canada|BOLD:AAG9780
Cyclopidae|SWCHL1471-16|Canada|BOLD:AAG9780
Macrocyclops albidus|ZOOPS617-20|United States|BOLD:AAG9780
Macrocyclops albidus|ZOOPS618-20|United States|BOLD:AAG9780
Cyclopidae|SWCHL850-16|Canada|BOLD:AAG9780
Cyclopidae|SWCHL608-15|Canada|BOLD:AAG9780
Cyclopidae|SWCHL1259-16|Canada|BOLD:AAG9780
Cyclopidae|ECCRU034-10|Canada|BOLD:AAG9780
Cyclopidae|SWCHL050-15|Canada|BOLD:AAG9780
Cyclopidae|SWCHL149-15|Canada|BOLD:AAG9780
Cyclopidae|RRINV2514-15|Canada|BOLD:AAG9780
Cyclopidae|RRINV2561-15|Canada|BOLD:AAG9780
Cyclopidae|BACZP1365-16|Canada|BOLD:AAG9780
Cyclopidae|SWCHL2059-16|Canada|BOLD:AAG9780
Macrocyclops albidus|GBMNE18538-21|United States|BOLD:AAG9780
Cyclopidae|SWCHL823-16|Canada|BOLD:AAG9778
Cyclopidae|SWCHL1248-16|Canada|BOLD:AAG9778
Cyclopidae|SWCHL1274-16|Canada|BOLD:AAG9778
Cyclopidae|SWCHL1850-16|Canada|BOLD:AAG9778
Cyclopidae|RRINV2510-15|Canada|BOLD:AAG9778
Cyclopidae|RRINV2529-15|Canada|BOLD:AAG9778
Cyclopidae|SWCHL1870-16|Canada|BOLD:AAG9778
Cyclopidae|SWCHL1055-16|Canada|BOLD:AAG9778
Cyclopidae|SWCHL1050-16|Canada|BOLD:AAG9778
Cyclopidae|SWCHL848-16|Canada|BOLD:AAG9778
Cyclopidae|SWCHL824-16|Canada|BOLD:AAG9778
Cyclopidae|SWCHL815-16|Canada|BOLD:AAG9778
Cyclopidae|SWCHL812-16|Canada|BOLD:AAG9778
Cyclopidae|SWCHL1060-16|Canada|BOLD:AAG9778
Cyclopidae|SWCHL1061-16|Canada|BOLD:AAG9778
Macrocyclops albidus|SWCHL1075-16|Canada|BOLD:AAG9778
Cyclopidae|SWCHL1250-16|Canada|BOLD:AAG9778
Cyclopidae|SWCHL1848-16|Canada|BOLD:AAG9778

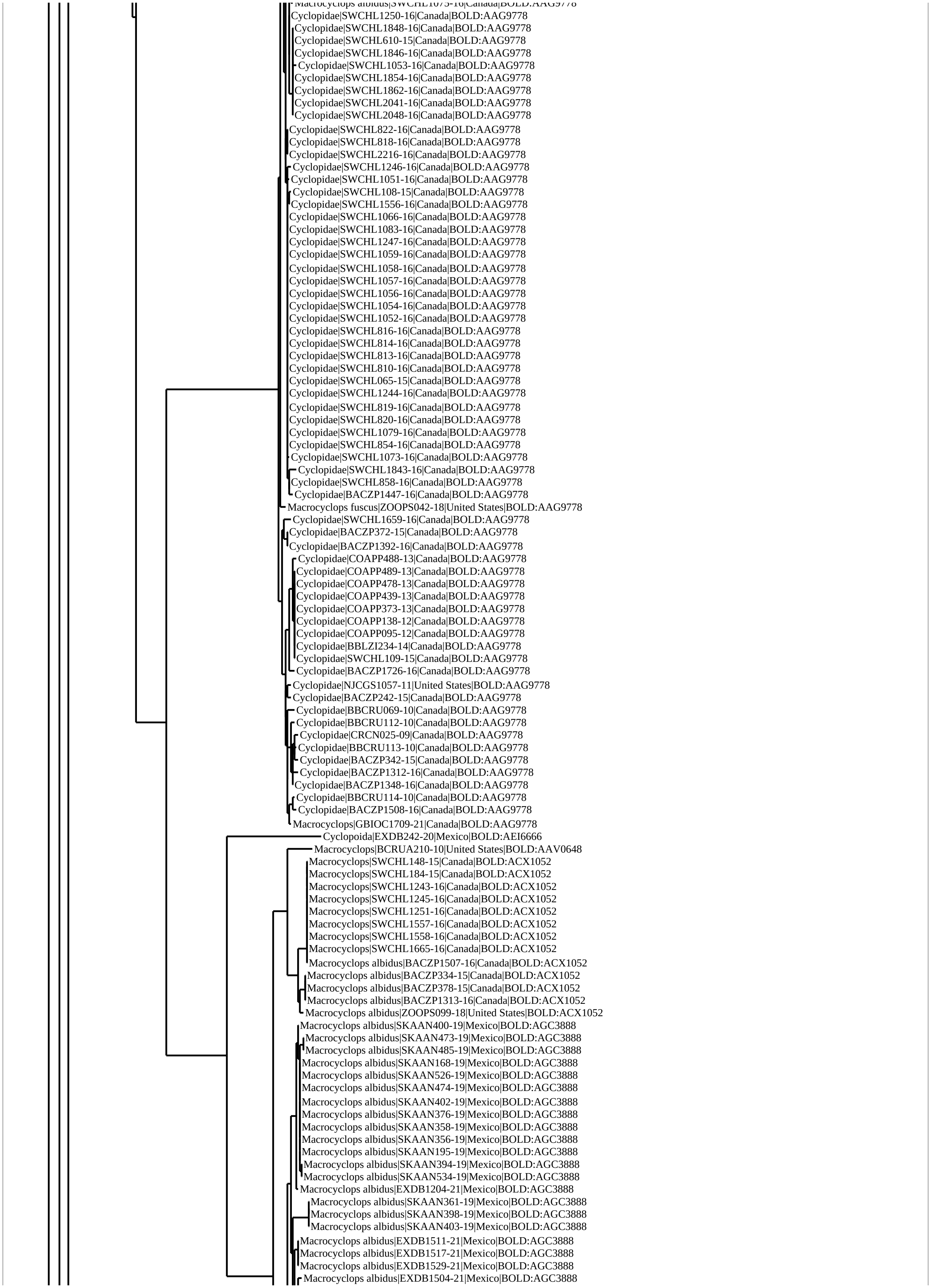
Macrocyclops albidus|SWCHL1075-16|Canada|BOLD:AAG9778
Cyclopidae|SWCHL1250-16|Canada|BOLD:AAG9778
Cyclopidae|SWCHL1848-16|Canada|BOLD:AAG9778
Cyclopidae|SWCHL610-15|Canada|BOLD:AAG9778
Cyclopidae|SWCHL1846-16|Canada|BOLD:AAG9778
Cyclopidae|SWCHL1053-16|Canada|BOLD:AAG9778
Cyclopidae|SWCHL1854-16|Canada|BOLD:AAG9778
Cyclopidae|SWCHL1862-16|Canada|BOLD:AAG9778
Cyclopidae|SWCHL2041-16|Canada|BOLD:AAG9778
Cyclopidae|SWCHL2048-16|Canada|BOLD:AAG9778
Cyclopidae|SWCHL822-16|Canada|BOLD:AAG9778
Cyclopidae|SWCHL818-16|Canada|BOLD:AAG9778
Cyclopidae|SWCHL2216-16|Canada|BOLD:AAG9778
Cyclopidae|SWCHL1246-16|Canada|BOLD:AAG9778
Cyclopidae|SWCHL1051-16|Canada|BOLD:AAG9778
Cyclopidae|SWCHL108-15|Canada|BOLD:AAG9778
Cyclopidae|SWCHL1556-16|Canada|BOLD:AAG9778
Cyclopidae|SWCHL1066-16|Canada|BOLD:AAG9778
Cyclopidae|SWCHL1083-16|Canada|BOLD:AAG9778
Cyclopidae|SWCHL1247-16|Canada|BOLD:AAG9778
Cyclopidae|SWCHL1059-16|Canada|BOLD:AAG9778
Cyclopidae|SWCHL1058-16|Canada|BOLD:AAG9778
Cyclopidae|SWCHL1057-16|Canada|BOLD:AAG9778
Cyclopidae|SWCHL1056-16|Canada|BOLD:AAG9778
Cyclopidae|SWCHL1054-16|Canada|BOLD:AAG9778
Cyclopidae|SWCHL1052-16|Canada|BOLD:AAG9778
Cyclopidae|SWCHL816-16|Canada|BOLD:AAG9778
Cyclopidae|SWCHL814-16|Canada|BOLD:AAG9778
Cyclopidae|SWCHL813-16|Canada|BOLD:AAG9778
Cyclopidae|SWCHL810-16|Canada|BOLD:AAG9778
Cyclopidae|SWCHL065-15|Canada|BOLD:AAG9778
Cyclopidae|SWCHL1244-16|Canada|BOLD:AAG9778
Cyclopidae|SWCHL819-16|Canada|BOLD:AAG9778
Cyclopidae|SWCHL820-16|Canada|BOLD:AAG9778
Cyclopidae|SWCHL1079-16|Canada|BOLD:AAG9778
Cyclopidae|SWCHL854-16|Canada|BOLD:AAG9778
Cyclopidae|SWCHL1073-16|Canada|BOLD:AAG9778
Cyclopidae|SWCHL1843-16|Canada|BOLD:AAG9778
Cyclopidae|SWCHL858-16|Canada|BOLD:AAG9778
Cyclopidae|BACZP1447-16|Canada|BOLD:AAG9778
Macrocyclops fuscus|ZOOPS042-18|United States|BOLD:AAG9778
Cyclopidae|SWCHL1659-16|Canada|BOLD:AAG9778
Cyclopidae|BACZP372-15|Canada|BOLD:AAG9778
Cyclopidae|BACZP1392-16|Canada|BOLD:AAG9778
Cyclopidae|COAPP488-13|Canada|BOLD:AAG9778
Cyclopidae|COAPP489-13|Canada|BOLD:AAG9778
Cyclopidae|COAPP478-13|Canada|BOLD:AAG9778
Cyclopidae|COAPP439-13|Canada|BOLD:AAG9778
Cyclopidae|COAPP373-13|Canada|BOLD:AAG9778
Cyclopidae|COAPP138-12|Canada|BOLD:AAG9778
Cyclopidae|COAPP095-12|Canada|BOLD:AAG9778
Cyclopidae|BBLZI234-14|Canada|BOLD:AAG9778
Cyclopidae|SWCHL109-15|Canada|BOLD:AAG9778
Cyclopidae|BACZP1726-16|Canada|BOLD:AAG9778
Cyclopidae|NJCGS1057-11|United States|BOLD:AAG9778
Cyclopidae|BACZP242-15|Canada|BOLD:AAG9778
Cyclopidae|BBCRU069-10|Canada|BOLD:AAG9778
Cyclopidae|BBCRU112-10|Canada|BOLD:AAG9778
Cyclopidae|CRCN025-09|Canada|BOLD:AAG9778
Cyclopidae|BBCRU113-10|Canada|BOLD:AAG9778
Cyclopidae|BACZP342-15|Canada|BOLD:AAG9778
Cyclopidae|BACZP1312-16|Canada|BOLD:AAG9778
Cyclopidae|BACZP1348-16|Canada|BOLD:AAG9778
Cyclopidae|BBCRU114-10|Canada|BOLD:AAG9778
Cyclopidae|BACZP1508-16|Canada|BOLD:AAG9778
Macrocyclops|GBIOC1709-21|Canada|BOLD:AAG9778
Cyclopoida|EXDB242-20|Mexico|BOLD:AEI6666
Macrocyclops|BCRUA210-10|United States|BOLD:AAV0648
Macrocyclops|SWCHL148-15|Canada|BOLD:ACX1052
Macrocyclops|SWCHL184-15|Canada|BOLD:ACX1052
Macrocyclops|SWCHL1243-16|Canada|BOLD:ACX1052
Macrocyclops|SWCHL1245-16|Canada|BOLD:ACX1052
Macrocyclops|SWCHL1251-16|Canada|BOLD:ACX1052
Macrocyclops|SWCHL1557-16|Canada|BOLD:ACX1052
Macrocyclops|SWCHL1558-16|Canada|BOLD:ACX1052
Macrocyclops|SWCHL1665-16|Canada|BOLD:ACX1052
Macrocyclops albidus|BACZP1507-16|Canada|BOLD:ACX1052
Macrocyclops albidus|BACZP334-15|Canada|BOLD:ACX1052
Macrocyclops albidus|BACZP378-15|Canada|BOLD:ACX1052
Macrocyclops albidus|BACZP1313-16|Canada|BOLD:ACX1052
Macrocyclops albidus|ZOOPS099-18|United States|BOLD:ACX1052
Macrocyclops albidus|SKAAN400-19|Mexico|BOLD:AGC3888
Macrocyclops albidus|SKAAN473-19|Mexico|BOLD:AGC3888
Macrocyclops albidus|SKAAN485-19|Mexico|BOLD:AGC3888
Macrocyclops albidus|SKAAN168-19|Mexico|BOLD:AGC3888
Macrocyclops albidus|SKAAN526-19|Mexico|BOLD:AGC3888
Macrocyclops albidus|SKAAN474-19|Mexico|BOLD:AGC3888
Macrocyclops albidus|SKAAN402-19|Mexico|BOLD:AGC3888
Macrocyclops albidus|SKAAN376-19|Mexico|BOLD:AGC3888
Macrocyclops albidus|SKAAN358-19|Mexico|BOLD:AGC3888
Macrocyclops albidus|SKAAN356-19|Mexico|BOLD:AGC3888
Macrocyclops albidus|SKAAN195-19|Mexico|BOLD:AGC3888
Macrocyclops albidus|SKAAN394-19|Mexico|BOLD:AGC3888
Macrocyclops albidus|SKAAN534-19|Mexico|BOLD:AGC3888
Macrocyclops albidus|EXDB1204-21|Mexico|BOLD:AGC3888
Macrocyclops albidus|SKAAN361-19|Mexico|BOLD:AGC3888
Macrocyclops albidus|SKAAN398-19|Mexico|BOLD:AGC3888
Macrocyclops albidus|SKAAN403-19|Mexico|BOLD:AGC3888
Macrocyclops albidus|EXDB1511-21|Mexico|BOLD:AGC3888
Macrocyclops albidus|EXDB1517-21|Mexico|BOLD:AGC3888
Macrocyclops albidus|EXDB1529-21|Mexico|BOLD:AGC3888
Macrocyclops albidus|EXDB1504-21|Mexico|BOLD:AGC3888

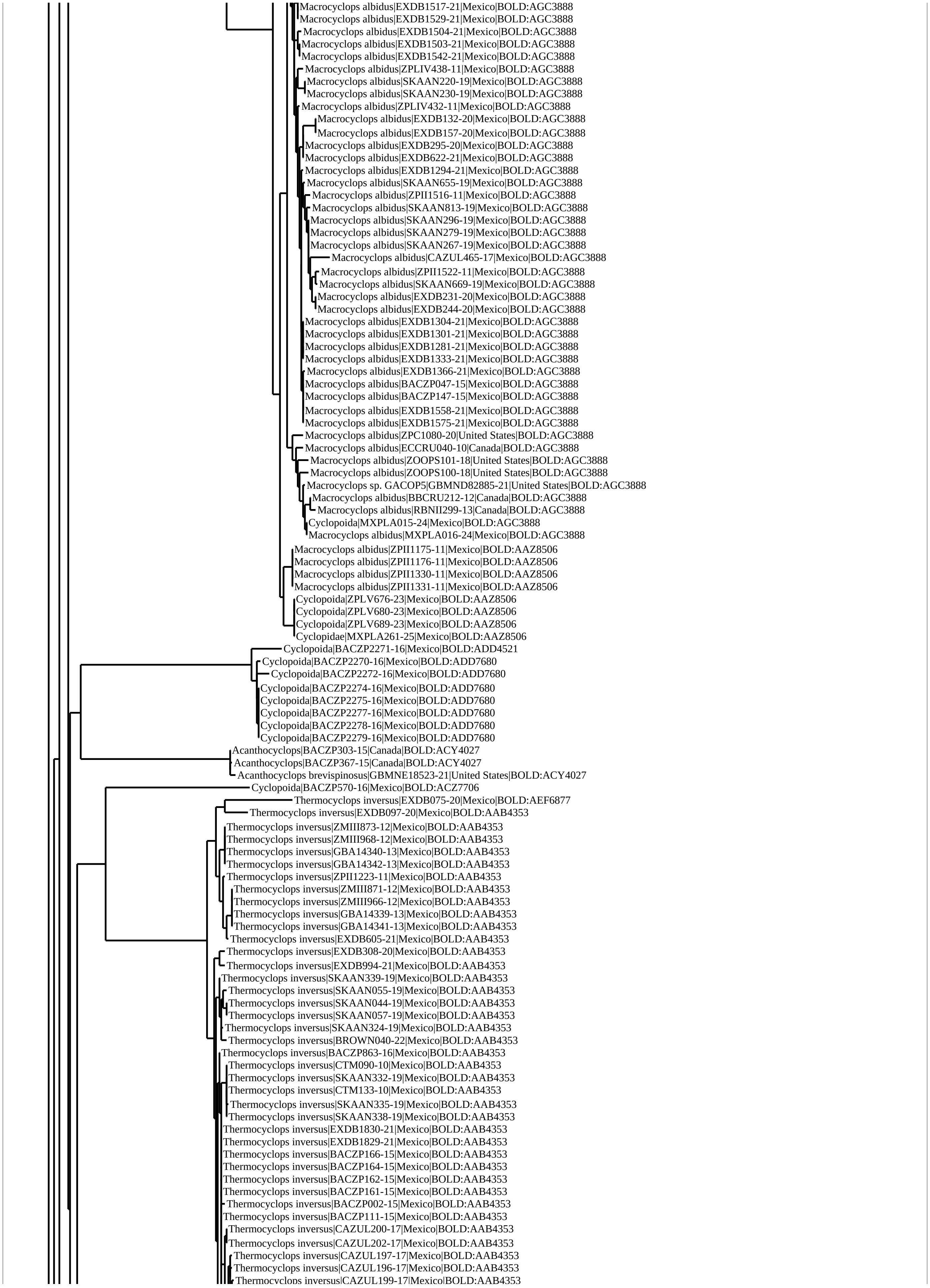

Macrocyclops albidus|EXDB1517-21|Mexico|BOLD:AGC3888
Macrocyclops albidus|EXDB1529-21|Mexico|BOLD:AGC3888
Macrocyclops albidus|EXDB1504-21|Mexico|BOLD:AGC3888
Macrocyclops albidus|EXDB1503-21|Mexico|BOLD:AGC3888
Macrocyclops albidus|EXDB1542-21|Mexico|BOLD:AGC3888
Macrocyclops albidus|ZPLIV438-11|Mexico|BOLD:AGC3888
Macrocyclops albidus|SKAAN220-19|Mexico|BOLD:AGC3888
Macrocyclops albidus|SKAAN230-19|Mexico|BOLD:AGC3888
Macrocyclops albidus|ZPLIV432-11|Mexico|BOLD:AGC3888
Macrocyclops albidus|EXDB132-20|Mexico|BOLD:AGC3888
Macrocyclops albidus|EXDB157-20|Mexico|BOLD:AGC3888
Macrocyclops albidus|EXDB295-20|Mexico|BOLD:AGC3888
Macrocyclops albidus|EXDB622-21|Mexico|BOLD:AGC3888
Macrocyclops albidus|EXDB1294-21|Mexico|BOLD:AGC3888
Macrocyclops albidus|SKAAN655-19|Mexico|BOLD:AGC3888
Macrocyclops albidus|ZPII1516-11|Mexico|BOLD:AGC3888
Macrocyclops albidus|SKAAN813-19|Mexico|BOLD:AGC3888
Macrocyclops albidus|SKAAN296-19|Mexico|BOLD:AGC3888
Macrocyclops albidus|SKAAN279-19|Mexico|BOLD:AGC3888
Macrocyclops albidus|SKAAN267-19|Mexico|BOLD:AGC3888
Macrocyclops albidus|CAZUL465-17|Mexico|BOLD:AGC3888
Macrocyclops albidus|ZPII1522-11|Mexico|BOLD:AGC3888
Macrocyclops albidus|SKAAN669-19|Mexico|BOLD:AGC3888
Macrocyclops albidus|EXDB231-20|Mexico|BOLD:AGC3888
Macrocyclops albidus|EXDB244-20|Mexico|BOLD:AGC3888
Macrocyclops albidus|EXDB1304-21|Mexico|BOLD:AGC3888
Macrocyclops albidus|EXDB1301-21|Mexico|BOLD:AGC3888
Macrocyclops albidus|EXDB1281-21|Mexico|BOLD:AGC3888
Macrocyclops albidus|EXDB1333-21|Mexico|BOLD:AGC3888
Macrocyclops albidus|EXDB1366-21|Mexico|BOLD:AGC3888
Macrocyclops albidus|BACZP047-15|Mexico|BOLD:AGC3888
Macrocyclops albidus|BACZP147-15|Mexico|BOLD:AGC3888
Macrocyclops albidus|EXDB1558-21|Mexico|BOLD:AGC3888
Macrocyclops albidus|EXDB1575-21|Mexico|BOLD:AGC3888
Macrocyclops albidus|ZPC1080-20|United States|BOLD:AGC3888
Macrocyclops albidus|ECCRU040-10|Canada|BOLD:AGC3888
Macrocyclops albidus|ZOOPS101-18|United States|BOLD:AGC3888
Macrocyclops albidus|ZOOPS100-18|United States|BOLD:AGC3888
Macrocyclops sp. GACOP5|GBMND82885-21|United States|BOLD:AGC3888
Macrocyclops albidus|BBCRU212-12|Canada|BOLD:AGC3888
Macrocyclops albidus|RBNII299-13|Canada|BOLD:AGC3888
Cyclopoida|MXPLA015-24|Mexico|BOLD:AGC3888
Macrocyclops albidus|MXPLA016-24|Mexico|BOLD:AGC3888
Macrocyclops albidus|ZPII1175-11|Mexico|BOLD:AAZ8506
Macrocyclops albidus|ZPII1176-11|Mexico|BOLD:AAZ8506
Macrocyclops albidus|ZPII1330-11|Mexico|BOLD:AAZ8506
Macrocyclops albidus|ZPII1331-11|Mexico|BOLD:AAZ8506
Cyclopoida|ZPLV676-23|Mexico|BOLD:AAZ8506
Cyclopoida|ZPLV680-23|Mexico|BOLD:AAZ8506
Cyclopoida|ZPLV689-23|Mexico|BOLD:AAZ8506
Cyclopidae|MXPLA261-25|Mexico|BOLD:AAZ8506
Cyclopoida|BACZP2271-16|Mexico|BOLD:ADD4521
Cyclopoida|BACZP2270-16|Mexico|BOLD:ADD7680
Cyclopoida|BACZP2272-16|Mexico|BOLD:ADD7680
Cyclopoida|BACZP2274-16|Mexico|BOLD:ADD7680
Cyclopoida|BACZP2275-16|Mexico|BOLD:ADD7680
Cyclopoida|BACZP2277-16|Mexico|BOLD:ADD7680
Cyclopoida|BACZP2278-16|Mexico|BOLD:ADD7680
Cyclopoida|BACZP2279-16|Mexico|BOLD:ADD7680
Acanthocyclops|BACZP303-15|Canada|BOLD:ACY4027
Acanthocyclops|BACZP367-15|Canada|BOLD:ACY4027
Acanthocyclops brevispinosus|GBMNE18523-21|United States|BOLD:ACY4027
Cyclopoida|BACZP570-16|Mexico|BOLD:ACZ7706
Thermocyclops inversus|EXDB075-20|Mexico|BOLD:AEF6877
Thermocyclops inversus|EXDB097-20|Mexico|BOLD:AAB4353
Thermocyclops inversus|ZMIII873-12|Mexico|BOLD:AAB4353
Thermocyclops inversus|ZMIII968-12|Mexico|BOLD:AAB4353
Thermocyclops inversus|GBA14340-13|Mexico|BOLD:AAB4353
Thermocyclops inversus|GBA14342-13|Mexico|BOLD:AAB4353
Thermocyclops inversus|ZPII1223-11|Mexico|BOLD:AAB4353
Thermocyclops inversus|ZMIII871-12|Mexico|BOLD:AAB4353
Thermocyclops inversus|ZMIII966-12|Mexico|BOLD:AAB4353
Thermocyclops inversus|GBA14339-13|Mexico|BOLD:AAB4353
Thermocyclops inversus|GBA14341-13|Mexico|BOLD:AAB4353
Thermocyclops inversus|EXDB605-21|Mexico|BOLD:AAB4353
Thermocyclops inversus|EXDB308-20|Mexico|BOLD:AAB4353
Thermocyclops inversus|EXDB994-21|Mexico|BOLD:AAB4353
Thermocyclops inversus|SKAAN339-19|Mexico|BOLD:AAB4353
Thermocyclops inversus|SKAAN055-19|Mexico|BOLD:AAB4353
Thermocyclops inversus|SKAAN044-19|Mexico|BOLD:AAB4353
Thermocyclops inversus|SKAAN057-19|Mexico|BOLD:AAB4353
Thermocyclops inversus|SKAAN324-19|Mexico|BOLD:AAB4353
Thermocyclops inversus|BROWN040-22|Mexico|BOLD:AAB4353
Thermocyclops inversus|BACZP863-16|Mexico|BOLD:AAB4353
Thermocyclops inversus|CTM090-10|Mexico|BOLD:AAB4353
Thermocyclops inversus|SKAAN332-19|Mexico|BOLD:AAB4353
Thermocyclops inversus|CTM133-10|Mexico|BOLD:AAB4353
Thermocyclops inversus|SKAAN335-19|Mexico|BOLD:AAB4353
Thermocyclops inversus|SKAAN338-19|Mexico|BOLD:AAB4353
Thermocyclops inversus|EXDB1830-21|Mexico|BOLD:AAB4353
Thermocyclops inversus|EXDB1829-21|Mexico|BOLD:AAB4353
Thermocyclops inversus|BACZP166-15|Mexico|BOLD:AAB4353
Thermocyclops inversus|BACZP164-15|Mexico|BOLD:AAB4353
Thermocyclops inversus|BACZP162-15|Mexico|BOLD:AAB4353
Thermocyclops inversus|BACZP161-15|Mexico|BOLD:AAB4353
Thermocyclops inversus|BACZP002-15|Mexico|BOLD:AAB4353
Thermocyclops inversus|BACZP111-15|Mexico|BOLD:AAB4353
Thermocyclops inversus|CAZUL200-17|Mexico|BOLD:AAB4353
Thermocyclops inversus|CAZUL202-17|Mexico|BOLD:AAB4353
Thermocyclops inversus|CAZUL197-17|Mexico|BOLD:AAB4353
Thermocyclops inversus|CAZUL196-17|Mexico|BOLD:AAB4353
Thermocyclops inversus|CAZUL199-17|Mexico|BOLD:AAB4353

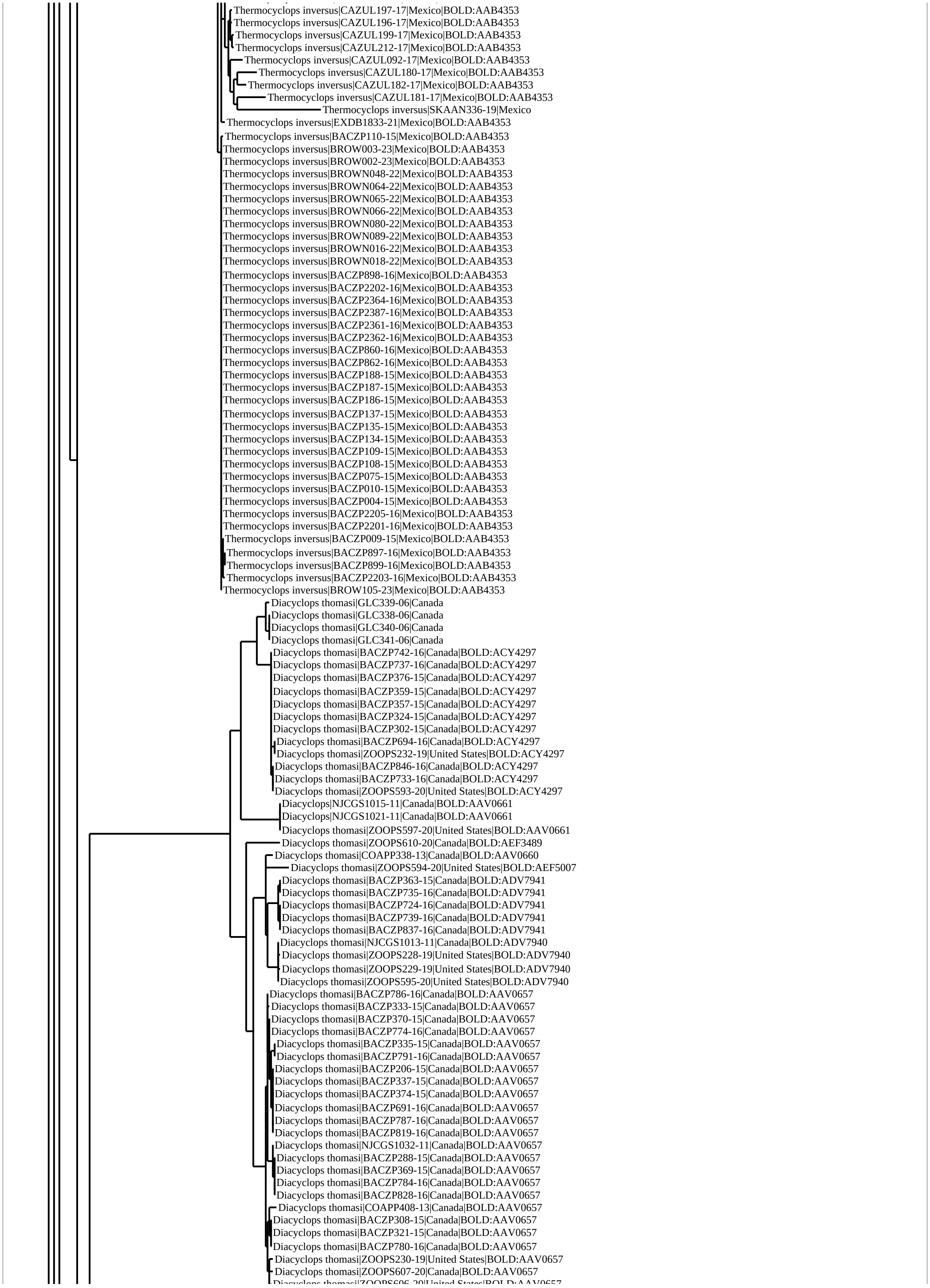
Thermocyclops inversus|CAZUL197-17|Mexico|BOLD:AAB4353
Thermocyclops inversus|CAZUL196-17|Mexico|BOLD:AAB4353
Thermocyclops inversus|CAZUL199-17|Mexico|BOLD:AAB4353
Thermocyclops inversus|CAZUL212-17|Mexico|BOLD:AAB4353
Thermocyclops inversus|CAZUL092-17|Mexico|BOLD:AAB4353
Thermocyclops inversus|CAZUL180-17|Mexico|BOLD:AAB4353
Thermocyclops inversus|CAZUL182-17|Mexico|BOLD:AAB4353
Thermocyclops inversus|CAZUL181-17|Mexico|BOLD:AAB4353
Thermocyclops inversus|SKAAN336-19|Mexico
Thermocyclops inversus|EXDB1833-21|Mexico|BOLD:AAB4353
Thermocyclops inversus|BACZP110-15|Mexico|BOLD:AAB4353
Thermocyclops inversus|BROW003-23|Mexico|BOLD:AAB4353
Thermocyclops inversus|BROW002-23|Mexico|BOLD:AAB4353
Thermocyclops inversus|BROWN048-22|Mexico|BOLD:AAB4353
Thermocyclops inversus|BROWN064-22|Mexico|BOLD:AAB4353
Thermocyclops inversus|BROWN065-22|Mexico|BOLD:AAB4353
Thermocyclops inversus|BROWN066-22|Mexico|BOLD:AAB4353
Thermocyclops inversus|BROWN080-22|Mexico|BOLD:AAB4353
Thermocyclops inversus|BROWN089-22|Mexico|BOLD:AAB4353
Thermocyclops inversus|BROWN016-22|Mexico|BOLD:AAB4353
Thermocyclops inversus|BROWN018-22|Mexico|BOLD:AAB4353
Thermocyclops inversus|BACZP898-16|Mexico|BOLD:AAB4353
Thermocyclops inversus|BACZP2202-16|Mexico|BOLD:AAB4353
Thermocyclops inversus|BACZP2364-16|Mexico|BOLD:AAB4353
Thermocyclops inversus|BACZP2387-16|Mexico|BOLD:AAB4353
Thermocyclops inversus|BACZP2361-16|Mexico|BOLD:AAB4353
Thermocyclops inversus|BACZP2362-16|Mexico|BOLD:AAB4353
Thermocyclops inversus|BACZP860-16|Mexico|BOLD:AAB4353
Thermocyclops inversus|BACZP862-16|Mexico|BOLD:AAB4353
Thermocyclops inversus|BACZP188-15|Mexico|BOLD:AAB4353
Thermocyclops inversus|BACZP187-15|Mexico|BOLD:AAB4353
Thermocyclops inversus|BACZP186-15|Mexico|BOLD:AAB4353
Thermocyclops inversus|BACZP137-15|Mexico|BOLD:AAB4353
Thermocyclops inversus|BACZP135-15|Mexico|BOLD:AAB4353
Thermocyclops inversus|BACZP134-15|Mexico|BOLD:AAB4353
Thermocyclops inversus|BACZP109-15|Mexico|BOLD:AAB4353
Thermocyclops inversus|BACZP108-15|Mexico|BOLD:AAB4353
Thermocyclops inversus|BACZP075-15|Mexico|BOLD:AAB4353
Thermocyclops inversus|BACZP010-15|Mexico|BOLD:AAB4353
Thermocyclops inversus|BACZP004-15|Mexico|BOLD:AAB4353
Thermocyclops inversus|BACZP2205-16|Mexico|BOLD:AAB4353
Thermocyclops inversus|BACZP2201-16|Mexico|BOLD:AAB4353
Thermocyclops inversus|BACZP009-15|Mexico|BOLD:AAB4353
Thermocyclops inversus|BACZP897-16|Mexico|BOLD:AAB4353
Thermocyclops inversus|BACZP899-16|Mexico|BOLD:AAB4353
Thermocyclops inversus|BACZP2203-16|Mexico|BOLD:AAB4353
Thermocyclops inversus|BROW105-23|Mexico|BOLD:AAB4353
Diacyclops thomasi|GLC339-06|Canada
Diacyclops thomasi|GLC338-06|Canada
Diacyclops thomasi|GLC340-06|Canada
Diacyclops thomasi|GLC341-06|Canada
Diacyclops thomasi|BACZP742-16|Canada|BOLD:ACY4297
Diacyclops thomasi|BACZP737-16|Canada|BOLD:ACY4297
Diacyclops thomasi|BACZP376-15|Canada|BOLD:ACY4297
Diacyclops thomasi|BACZP359-15|Canada|BOLD:ACY4297
Diacyclops thomasi|BACZP357-15|Canada|BOLD:ACY4297
Diacyclops thomasi|BACZP324-15|Canada|BOLD:ACY4297
Diacyclops thomasi|BACZP302-15|Canada|BOLD:ACY4297
Diacyclops thomasi|BACZP694-16|Canada|BOLD:ACY4297
Diacyclops thomasi|ZOOPS232-19|United States|BOLD:ACY4297
Diacyclops thomasi|BACZP846-16|Canada|BOLD:ACY4297
Diacyclops thomasi|BACZP733-16|Canada|BOLD:ACY4297
Diacyclops thomasi|ZOOPS593-20|United States|BOLD:ACY4297
Diacyclops|NJCGS1015-11|Canada|BOLD:AAV0661
Diacyclops|NJCGS1021-11|Canada|BOLD:AAV0661
Diacyclops thomasi|ZOOPS597-20|United States|BOLD:AAV0661
Diacyclops thomasi|ZOOPS610-20|Canada|BOLD:AEF3489
Diacyclops thomasi|COAPP338-13|Canada|BOLD:AAV0660
Diacyclops thomasi|ZOOPS594-20|United States|BOLD:AEF5007
Diacyclops thomasi|BACZP363-15|Canada|BOLD:ADV7941
Diacyclops thomasi|BACZP735-16|Canada|BOLD:ADV7941
Diacyclops thomasi|BACZP724-16|Canada|BOLD:ADV7941
Diacyclops thomasi|BACZP739-16|Canada|BOLD:ADV7941
Diacyclops thomasi|BACZP837-16|Canada|BOLD:ADV7941
Diacyclops thomasi|NJCGS1013-11|Canada|BOLD:ADV7940
Diacyclops thomasi|ZOOPS228-19|United States|BOLD:ADV7940
Diacyclops thomasi|ZOOPS229-19|United States|BOLD:ADV7940
Diacyclops thomasi|ZOOPS595-20|United States|BOLD:ADV7940
Diacyclops thomasi|BACZP786-16|Canada|BOLD:AAV0657
Diacyclops thomasi|BACZP333-15|Canada|BOLD:AAV0657
Diacyclops thomasi|BACZP370-15|Canada|BOLD:AAV0657
Diacyclops thomasi|BACZP774-16|Canada|BOLD:AAV0657
Diacyclops thomasi|BACZP335-15|Canada|BOLD:AAV0657
Diacyclops thomasi|BACZP791-16|Canada|BOLD:AAV0657
Diacyclops thomasi|BACZP206-15|Canada|BOLD:AAV0657
Diacyclops thomasi|BACZP337-15|Canada|BOLD:AAV0657
Diacyclops thomasi|BACZP374-15|Canada|BOLD:AAV0657
Diacyclops thomasi|BACZP691-16|Canada|BOLD:AAV0657
Diacyclops thomasi|BACZP787-16|Canada|BOLD:AAV0657
Diacyclops thomasi|BACZP819-16|Canada|BOLD:AAV0657
Diacyclops thomasi|NJCGS1032-11|Canada|BOLD:AAV0657
Diacyclops thomasi|BACZP288-15|Canada|BOLD:AAV0657
Diacyclops thomasi|BACZP369-15|Canada|BOLD:AAV0657
Diacyclops thomasi|BACZP784-16|Canada|BOLD:AAV0657
Diacyclops thomasi|BACZP828-16|Canada|BOLD:AAV0657
Diacyclops thomasi|COAPP408-13|Canada|BOLD:AAV0657
Diacyclops thomasi|BACZP308-15|Canada|BOLD:AAV0657
Diacyclops thomasi|BACZP321-15|Canada|BOLD:AAV0657
Diacyclops thomasi|BACZP780-16|Canada|BOLD:AAV0657
Diacyclops thomasi|ZOOPS230-19|United States|BOLD:AAV0657
Diacyclops thomasi|ZOOPS607-20|Canada|BOLD:AAV0657

[illegible]
Diacyclops thomasi|ZOOPS230-19|United States|BOLD:AAV0657
Diacyclops thomasi|ZOOPS607-20|Canada|BOLD:AAV0657
Diacyclops thomasi|ZOOPS606-20|United States|BOLD:AAV0657
Diacyclops thomasi|ZOOPS596-20|United States|BOLD:AAV0657
Diacyclops thomasi|ZOOPS035-18|Canada|BOLD:AAV0657
Diacyclops thomasi|ZOOPS034-18|Canada|BOLD:AAV0657
Diacyclops thomasi|ZOOPS231-19|United States|BOLD:AAV0657
Cyclopidae|SFEZ009-21|United States|BOLD:AAV0657
Cyclopidae|SFEZ011-21|United States|BOLD:AAV0657
Cyclopidae|SFEZ010-21|United States|BOLD:AAV0657
Cyclopidae|SFEZ023-21|United States|BOLD:AAV0657
CyclopoidGEN sp.6 CHU|OZFWZ292-11|Canada|BOLD:AAZ0251
CyclopoidGEN sp.6 CHU|OZFWZ295-11|Canada|BOLD:AAZ0251
CyclopoidGEN sp.6 CHU|OZFWZ296-11|Canada|BOLD:AAZ0251
Cyclops cf. divergens|ZOOPS581-20|United States|BOLD:ACL6037
Cyclops scutifer|DNARA1645-21|Canada|BOLD:AEB5314
Cyclops scutifer|DNARA127-21|Canada|BOLD:AEB5314
Cyclops scutifer|DNARA3045-21|Canada|BOLD:AEB5314
Cyclops scutifer|GBCM19635-19|Canada|BOLD:AEB5314
Cyclops scutifer|DNARA702-21|Canada|BOLD:AEB5314
Cyclops scutifer|DNARA1166-21|Canada|BOLD:AEB5314
Cyclops scutifer|DNARA498-21|Canada|BOLD:AEB5314
Cyclops scutifer|DNARA1169-21|Canada|BOLD:AEB5314
Cyclops scutifer|DNARA1168-21|Canada|BOLD:AEB5314
Cyclops scutifer|DNARA1165-21|Canada|BOLD:AEB5314
Cyclops scutifer|DNARA1164-21|Canada|BOLD:AEB5314
Cyclops scutifer|DNARA1163-21|Canada|BOLD:AEB5314
Cyclops scutifer|DNARA110-21|Canada|BOLD:AEB5314
Cyclops scutifer|DNARA1161-21|Canada|BOLD:AEB5314
Cyclops scutifer|DNARA1171-21|Canada|BOLD:AEB5314
Cyclops scutifer|DNARA1172-21|Canada|BOLD:AEB5314
Cyclops scutifer|DNARA1173-21|Canada|BOLD:AEB5314
Cyclops scutifer|DNARA1482-21|Canada|BOLD:AEB5314
Cyclops scutifer|DNARA289-21|Canada|BOLD:AEB5314
Cyclops scutifer|DNARA1687-21|Canada|BOLD:AEB5314
Cyclops scutifer|DNARA1668-21|Canada|BOLD:AEB5314
Cyclops scutifer|DNARA1692-21|Canada|BOLD:AEB5314
Cyclops scutifer|DNARA1661-21|Canada|BOLD:AEB5314
Cyclops scutifer|DNARA3360-21|Canada|BOLD:AEB5314
Cyclops scutifer|DNARA510-21|Canada|BOLD:AEB5314
Cyclops scutifer|DNARA1842-21|Canada|BOLD:AEB5314
Cyclops scutifer|DNARA1855-21|Canada|BOLD:AEB5314
Cyclops scutifer|DNARA1451-21|Canada|BOLD:AEB5314
Cyclops scutifer|DNARA1456-21|Canada|BOLD:AEB5314
Cyclops scutifer|DNARA139-21|Canada|BOLD:AEB5314
Cyclops scutifer|DNARA116-21|Canada|BOLD:AEB5314
Cyclops scutifer|DNARA115-21|Canada|BOLD:AEB5314
Cyclops scutifer|DNARA168-21|Canada|BOLD:AEB5314
Cyclops scutifer|DNARA286-21|Canada|BOLD:AEB5314
Cyclops scutifer|DNARA319-21|Canada|BOLD:AEB5314
Cyclops scutifer|DNARA329-21|Canada|BOLD:AEB5314
Cyclops scutifer|DNARA370-21|Canada|BOLD:AEB5314
Cyclops scutifer|DNARA481-21|Canada|BOLD:AEB5314
Cyclops scutifer|DNARA496-21|Canada|BOLD:AEB5314
Cyclops scutifer|DNARA497-21|Canada|BOLD:AEB5314
Cyclops scutifer|DNARA506-21|Canada|BOLD:AEB5314
Cyclops scutifer|DNARA1895-21|Canada|BOLD:AEB5314
Cyclops scutifer|DNARA1816-21|Canada|BOLD:AEB5314
Cyclops scutifer|DNARA1639-21|Canada|BOLD:AEB5314
Cyclops scutifer|DNARA2345-21|Canada|BOLD:AEB5314
Cyclops scutifer|DNARA2343-21|Canada|BOLD:AEB5314
Cyclops scutifer|DNARA1864-21|Canada|BOLD:AEB5314
Cyclops scutifer|DNARA1863-21|Canada|BOLD:AEB5314
Cyclops scutifer|DNARA1862-21|Canada|BOLD:AEB5314
Cyclops scutifer|DNARA1861-21|Canada|BOLD:AEB5314
Cyclops scutifer|DNARA1860-21|Canada|BOLD:AEB5314
Cyclops scutifer|DNARA1849-21|Canada|BOLD:AEB5314
Cyclops scutifer|DNARA1825-21|Canada|BOLD:AEB5314
Cyclops scutifer|DNARA1817-21|Canada|BOLD:AEB5314
Cyclops scutifer|DNARA1689-21|Canada|BOLD:AEB5314
Cyclops scutifer|DNARA1672-21|Canada|BOLD:AEB5314
Cyclops scutifer|DNARA1671-21|Canada|BOLD:AEB5314
Cyclops scutifer|DNARA1669-21|Canada|BOLD:AEB5314
Cyclops scutifer|DNARA1662-21|Canada|BOLD:AEB5314
Cyclops scutifer|DNARA1651-21|Canada|BOLD:AEB5314
Cyclops scutifer|DNARA1650-21|Canada|BOLD:AEB5314
Cyclops scutifer|DNARA1646-21|Canada|BOLD:AEB5314
Cyclops scutifer|DNARA1641-21|Canada|BOLD:AEB5314
Cyclops scutifer|DNARA1636-21|Canada|BOLD:AEB5314
Cyclops scutifer|DNARA1635-21|Canada|BOLD:AEB5314
Cyclops scutifer|DNARA1634-21|Canada|BOLD:AEB5314
Cyclops scutifer|DNARA1483-21|Canada|BOLD:AEB5314
Cyclops scutifer|DNARA1478-21|Canada|BOLD:AEB5314
Cyclops scutifer|DNARA1475-21|Canada|BOLD:AEB5314
Cyclops scutifer|DNARA1464-21|Canada|BOLD:AEB5314
Cyclops scutifer|DNARA1463-21|Canada|BOLD:AEB5314
Cyclops scutifer|DNARA1432-21|Canada|BOLD:AEB5314
Cyclops scutifer|DNARA1226-21|Canada|BOLD:AEB5314
Cyclops scutifer|DNARA1208-21|Canada|BOLD:AEB5314
Cyclops scutifer|DNARA1207-21|Canada|BOLD:AEB5314
Cyclops scutifer|DNARA1206-21|Canada|BOLD:AEB5314
Cyclops scutifer|DNARA1205-21|Canada|BOLD:AEB5314
Cyclops scutifer|DNARA1197-21|Canada|BOLD:AEB5314
Cyclops scutifer|DNARA1196-21|Canada|BOLD:AEB5314
Cyclops scutifer|DNARA1192-21|Canada|BOLD:AEB5314
Cyclops scutifer|DNARA701-21|Canada|BOLD:AEB5314
Cyclops scutifer|DNARA499-21|Canada|BOLD:AEB5314
Cyclops scutifer|DNARA477-21|Canada|BOLD:AEB5314
Cyclops scutifer|DNARA476-21|Canada|BOLD:AEB5314
Cyclops scutifer|DNARA371-21|Canada|BOLD:AEB5314
Cyclops scutifer|DNARA367-21|Canada|BOLD:AEB5314
[illegible]

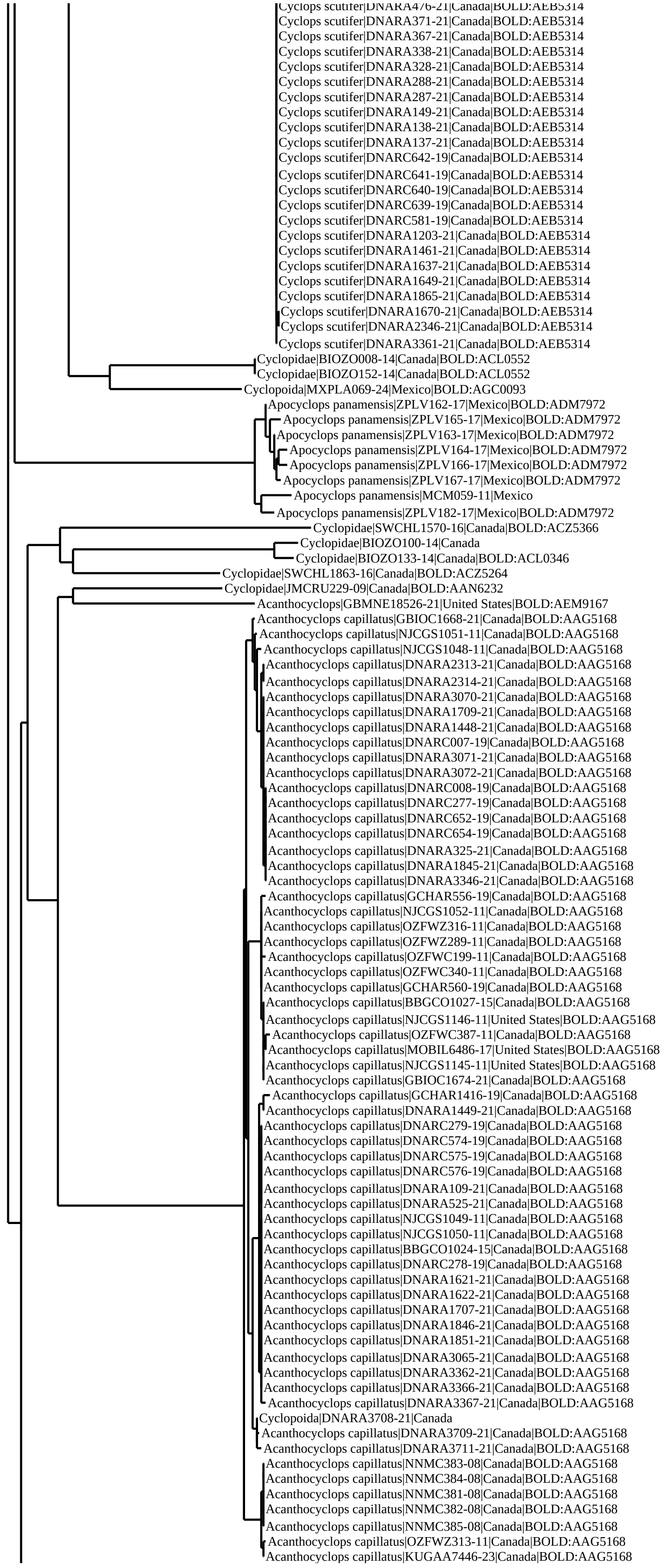
Cyclops scutifer|DNARA476-21|Canada|BOLD:AEB5314
Cyclops scutifer|DNARA371-21|Canada|BOLD:AEB5314
Cyclops scutifer|DNARA367-21|Canada|BOLD:AEB5314
Cyclops scutifer|DNARA338-21|Canada|BOLD:AEB5314
Cyclops scutifer|DNARA328-21|Canada|BOLD:AEB5314
Cyclops scutifer|DNARA288-21|Canada|BOLD:AEB5314
Cyclops scutifer|DNARA287-21|Canada|BOLD:AEB5314
Cyclops scutifer|DNARA149-21|Canada|BOLD:AEB5314
Cyclops scutifer|DNARA138-21|Canada|BOLD:AEB5314
Cyclops scutifer|DNARA137-21|Canada|BOLD:AEB5314
Cyclops scutifer|DNARC642-19|Canada|BOLD:AEB5314
Cyclops scutifer|DNARC641-19|Canada|BOLD:AEB5314
Cyclops scutifer|DNARC640-19|Canada|BOLD:AEB5314
Cyclops scutifer|DNARC639-19|Canada|BOLD:AEB5314
Cyclops scutifer|DNARC581-19|Canada|BOLD:AEB5314
Cyclops scutifer|DNARA1203-21|Canada|BOLD:AEB5314
Cyclops scutifer|DNARA1461-21|Canada|BOLD:AEB5314
Cyclops scutifer|DNARA1637-21|Canada|BOLD:AEB5314
Cyclops scutifer|DNARA1649-21|Canada|BOLD:AEB5314
Cyclops scutifer|DNARA1865-21|Canada|BOLD:AEB5314
Cyclops scutifer|DNARA1670-21|Canada|BOLD:AEB5314
Cyclops scutifer|DNARA2346-21|Canada|BOLD:AEB5314
Cyclops scutifer|DNARA3361-21|Canada|BOLD:AEB5314
Cyclopidae|BIOZO008-14|Canada|BOLD:ACL0552
Cyclopidae|BIOZO152-14|Canada|BOLD:ACL0552
Cyclopoida|MXPLA069-24|Mexico|BOLD:AGC0093
Apocyclops panamensis|ZPLV162-17|Mexico|BOLD:ADM7972
Apocyclops panamensis|ZPLV165-17|Mexico|BOLD:ADM7972
Apocyclops panamensis|ZPLV163-17|Mexico|BOLD:ADM7972
Apocyclops panamensis|ZPLV164-17|Mexico|BOLD:ADM7972
Apocyclops panamensis|ZPLV166-17|Mexico|BOLD:ADM7972
Apocyclops panamensis|ZPLV167-17|Mexico|BOLD:ADM7972
Apocyclops panamensis|MCM059-11|Mexico
Apocyclops panamensis|ZPLV182-17|Mexico|BOLD:ADM7972
Cyclopidae|SWCHL1570-16|Canada|BOLD:ACZ5366
Cyclopidae|BIOZO100-14|Canada
Cyclopidae|BIOZO133-14|Canada|BOLD:ACL0346
Cyclopidae|SWCHL1863-16|Canada|BOLD:ACZ5264
Cyclopidae|JMCRU229-09|Canada|BOLD:AAN6232
Acanthocyclops|GBMNE18526-21|United States|BOLD:AEM9167
Acanthocyclops capillatus|GBIOC1668-21|Canada|BOLD:AAG5168
Acanthocyclops capillatus|NJCGS1051-11|Canada|BOLD:AAG5168
Acanthocyclops capillatus|NJCGS1048-11|Canada|BOLD:AAG5168
Acanthocyclops capillatus|DNARA2313-21|Canada|BOLD:AAG5168
Acanthocyclops capillatus|DNARA2314-21|Canada|BOLD:AAG5168
Acanthocyclops capillatus|DNARA3070-21|Canada|BOLD:AAG5168
Acanthocyclops capillatus|DNARA1709-21|Canada|BOLD:AAG5168
Acanthocyclops capillatus|DNARA1448-21|Canada|BOLD:AAG5168
Acanthocyclops capillatus|DNARC007-19|Canada|BOLD:AAG5168
Acanthocyclops capillatus|DNARA3071-21|Canada|BOLD:AAG5168
Acanthocyclops capillatus|DNARA3072-21|Canada|BOLD:AAG5168
Acanthocyclops capillatus|DNARC008-19|Canada|BOLD:AAG5168
Acanthocyclops capillatus|DNARC277-19|Canada|BOLD:AAG5168
Acanthocyclops capillatus|DNARC652-19|Canada|BOLD:AAG5168
Acanthocyclops capillatus|DNARC654-19|Canada|BOLD:AAG5168
Acanthocyclops capillatus|DNARA325-21|Canada|BOLD:AAG5168
Acanthocyclops capillatus|DNARA1845-21|Canada|BOLD:AAG5168
Acanthocyclops capillatus|DNARA3346-21|Canada|BOLD:AAG5168
Acanthocyclops capillatus|GCHAR556-19|Canada|BOLD:AAG5168
Acanthocyclops capillatus|NJCGS1052-11|Canada|BOLD:AAG5168
Acanthocyclops capillatus|OZFWZ316-11|Canada|BOLD:AAG5168
Acanthocyclops capillatus|OZFWZ289-11|Canada|BOLD:AAG5168
Acanthocyclops capillatus|OZFWC199-11|Canada|BOLD:AAG5168
Acanthocyclops capillatus|OZFWC340-11|Canada|BOLD:AAG5168
Acanthocyclops capillatus|GCHAR560-19|Canada|BOLD:AAG5168
Acanthocyclops capillatus|BBGCO1027-15|Canada|BOLD:AAG5168
Acanthocyclops capillatus|NJCGS1146-11|United States|BOLD:AAG5168
Acanthocyclops capillatus|OZFWC387-11|Canada|BOLD:AAG5168
Acanthocyclops capillatus|MOBIL6486-17|United States|BOLD:AAG5168
Acanthocyclops capillatus|NJCGS1145-11|United States|BOLD:AAG5168
Acanthocyclops capillatus|GBIOC1674-21|Canada|BOLD:AAG5168
Acanthocyclops capillatus|GCHAR1416-19|Canada|BOLD:AAG5168
Acanthocyclops capillatus|DNARA1449-21|Canada|BOLD:AAG5168
Acanthocyclops capillatus|DNARC279-19|Canada|BOLD:AAG5168
Acanthocyclops capillatus|DNARC574-19|Canada|BOLD:AAG5168
Acanthocyclops capillatus|DNARC575-19|Canada|BOLD:AAG5168
Acanthocyclops capillatus|DNARC576-19|Canada|BOLD:AAG5168
Acanthocyclops capillatus|DNARA109-21|Canada|BOLD:AAG5168
Acanthocyclops capillatus|DNARA525-21|Canada|BOLD:AAG5168
Acanthocyclops capillatus|NJCGS1049-11|Canada|BOLD:AAG5168
Acanthocyclops capillatus|NJCGS1050-11|Canada|BOLD:AAG5168
Acanthocyclops capillatus|BBGCO1024-15|Canada|BOLD:AAG5168
Acanthocyclops capillatus|DNARC278-19|Canada|BOLD:AAG5168
Acanthocyclops capillatus|DNARA1621-21|Canada|BOLD:AAG5168
Acanthocyclops capillatus|DNARA1622-21|Canada|BOLD:AAG5168
Acanthocyclops capillatus|DNARA1707-21|Canada|BOLD:AAG5168
Acanthocyclops capillatus|DNARA1846-21|Canada|BOLD:AAG5168
Acanthocyclops capillatus|DNARA1851-21|Canada|BOLD:AAG5168
Acanthocyclops capillatus|DNARA3065-21|Canada|BOLD:AAG5168
Acanthocyclops capillatus|DNARA3362-21|Canada|BOLD:AAG5168
Acanthocyclops capillatus|DNARA3366-21|Canada|BOLD:AAG5168
Acanthocyclops capillatus|DNARA3367-21|Canada|BOLD:AAG5168
Cyclopoida|DNARA3708-21|Canada
Acanthocyclops capillatus|DNARA3709-21|Canada|BOLD:AAG5168
Acanthocyclops capillatus|DNARA3711-21|Canada|BOLD:AAG5168
Acanthocyclops capillatus|NNMC383-08|Canada|BOLD:AAG5168
Acanthocyclops capillatus|NNMC384-08|Canada|BOLD:AAG5168
Acanthocyclops capillatus|NNMC381-08|Canada|BOLD:AAG5168
Acanthocyclops capillatus|NNMC382-08|Canada|BOLD:AAG5168
Acanthocyclops capillatus|NNMC385-08|Canada|BOLD:AAG5168
Acanthocyclops capillatus|OZFWZ313-11|Canada|BOLD:AAG5168
Acanthocyclops capillatus|KUGAA7446-23|Canada|BOLD:AAG5168

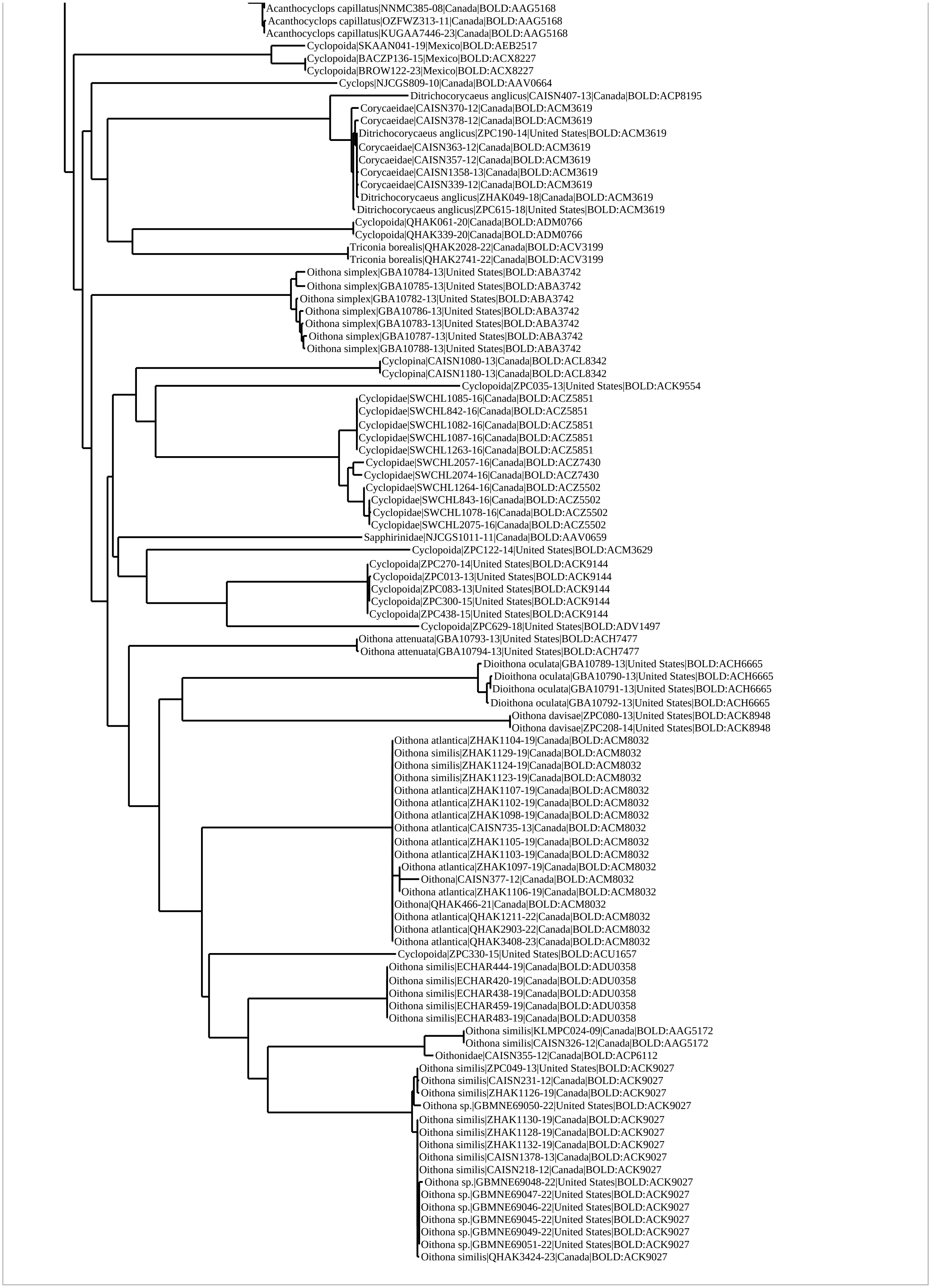
Acanthocyclops capillatus|NNMC385-08|Canada|BOLD:AAG5168
Acanthocyclops capillatus|OZFWZ313-11|Canada|BOLD:AAG5168
Acanthocyclops capillatus|KUGAA7446-23|Canada|BOLD:AAG5168
Cyclopoida|SKAAN041-19|Mexico|BOLD:AEB2517
Cyclopoida|BACZP136-15|Mexico|BOLD:ACX8227
Cyclopoida|BROW122-23|Mexico|BOLD:ACX8227
Cyclops|NJCGS809-10|Canada|BOLD:AAV0664
Ditrichocorycaeus anglicus|CAISN407-13|Canada|BOLD:ACP8195
Corycaeidae|CAISN370-12|Canada|BOLD:ACM3619
Corycaeidae|CAISN378-12|Canada|BOLD:ACM3619
Ditrichocorycaeus anglicus|ZPC190-14|United States|BOLD:ACM3619
Corycaeidae|CAISN363-12|Canada|BOLD:ACM3619
Corycaeidae|CAISN357-12|Canada|BOLD:ACM3619
Corycaeidae|CAISN1358-13|Canada|BOLD:ACM3619
Corycaeidae|CAISN339-12|Canada|BOLD:ACM3619
Ditrichocorycaeus anglicus|ZHAK049-18|Canada|BOLD:ACM3619
Ditrichocorycaeus anglicus|ZPC615-18|United States|BOLD:ACM3619
Cyclopoida|QHAK061-20|Canada|BOLD:ADM0766
Cyclopoida|QHAK339-20|Canada|BOLD:ADM0766
Triconia borealis|QHAK2028-22|Canada|BOLD:ACV3199
Triconia borealis|QHAK2741-22|Canada|BOLD:ACV3199
Oithona simplex|GBA10784-13|United States|BOLD:ABA3742
Oithona simplex|GBA10785-13|United States|BOLD:ABA3742
Oithona simplex|GBA10782-13|United States|BOLD:ABA3742
Oithona simplex|GBA10786-13|United States|BOLD:ABA3742
Oithona simplex|GBA10783-13|United States|BOLD:ABA3742
Oithona simplex|GBA10787-13|United States|BOLD:ABA3742
Oithona simplex|GBA10788-13|United States|BOLD:ABA3742
Cyclopina|CAISN1080-13|Canada|BOLD:ACL8342
Cyclopina|CAISN1180-13|Canada|BOLD:ACL8342
Cyclopoida|ZPC035-13|United States|BOLD:ACK9554
Cyclopidae|SWCHL1085-16|Canada|BOLD:ACZ5851
Cyclopidae|SWCHL842-16|Canada|BOLD:ACZ5851
Cyclopidae|SWCHL1082-16|Canada|BOLD:ACZ5851
Cyclopidae|SWCHL1087-16|Canada|BOLD:ACZ5851
Cyclopidae|SWCHL1263-16|Canada|BOLD:ACZ5851
Cyclopidae|SWCHL2057-16|Canada|BOLD:ACZ7430
Cyclopidae|SWCHL2074-16|Canada|BOLD:ACZ7430
Cyclopidae|SWCHL1264-16|Canada|BOLD:ACZ5502
Cyclopidae|SWCHL843-16|Canada|BOLD:ACZ5502
Cyclopidae|SWCHL1078-16|Canada|BOLD:ACZ5502
Cyclopidae|SWCHL2075-16|Canada|BOLD:ACZ5502
Sapphirinidae|NJCGS1011-11|Canada|BOLD:AAV0659
Cyclopoida|ZPC122-14|United States|BOLD:ACM3629
Cyclopoida|ZPC270-14|United States|BOLD:ACK9144
Cyclopoida|ZPC013-13|United States|BOLD:ACK9144
Cyclopoida|ZPC083-13|United States|BOLD:ACK9144
Cyclopoida|ZPC300-15|United States|BOLD:ACK9144
Cyclopoida|ZPC438-15|United States|BOLD:ACK9144
Cyclopoida|ZPC629-18|United States|BOLD:ADV1497
Oithona attenuata|GBA10793-13|United States|BOLD:ACH7477
Oithona attenuata|GBA10794-13|United States|BOLD:ACH7477
Dioithona oculata|GBA10789-13|United States|BOLD:ACH6665
Dioithona oculata|GBA10790-13|United States|BOLD:ACH6665
Dioithona oculata|GBA10791-13|United States|BOLD:ACH6665
Dioithona oculata|GBA10792-13|United States|BOLD:ACH6665
Oithona davisae|ZPC080-13|United States|BOLD:ACK8948
Oithona davisae|ZPC208-14|United States|BOLD:ACK8948
Oithona atlantica|ZHAK1104-19|Canada|BOLD:ACM8032
Oithona similis|ZHAK1129-19|Canada|BOLD:ACM8032
Oithona similis|ZHAK1124-19|Canada|BOLD:ACM8032
Oithona similis|ZHAK1123-19|Canada|BOLD:ACM8032
Oithona atlantica|ZHAK1107-19|Canada|BOLD:ACM8032
Oithona atlantica|ZHAK1102-19|Canada|BOLD:ACM8032
Oithona atlantica|ZHAK1098-19|Canada|BOLD:ACM8032
Oithona atlantica|CAISN735-13|Canada|BOLD:ACM8032
Oithona atlantica|ZHAK1105-19|Canada|BOLD:ACM8032
Oithona atlantica|ZHAK1103-19|Canada|BOLD:ACM8032
Oithona atlantica|ZHAK1097-19|Canada|BOLD:ACM8032
Oithona|CAISN377-12|Canada|BOLD:ACM8032
Oithona atlantica|ZHAK1106-19|Canada|BOLD:ACM8032
Oithona|QHAK466-21|Canada|BOLD:ACM8032
Oithona atlantica|QHAK1211-22|Canada|BOLD:ACM8032
Oithona atlantica|QHAK2903-22|Canada|BOLD:ACM8032
Oithona atlantica|QHAK3408-23|Canada|BOLD:ACM8032
Cyclopoida|ZPC330-15|United States|BOLD:ACU1657
Oithona similis|ECHAR444-19|Canada|BOLD:ADU0358
Oithona similis|ECHAR420-19|Canada|BOLD:ADU0358
Oithona similis|ECHAR438-19|Canada|BOLD:ADU0358
Oithona similis|ECHAR459-19|Canada|BOLD:ADU0358
Oithona similis|ECHAR483-19|Canada|BOLD:ADU0358
Oithona similis|KLMPC024-09|Canada|BOLD:AAG5172
Oithona similis|CAISN326-12|Canada|BOLD:AAG5172
Oithonidae|CAISN355-12|Canada|BOLD:ACP6112
Oithona similis|ZPC049-13|United States|BOLD:ACK9027
Oithona similis|CAISN231-12|Canada|BOLD:ACK9027
Oithona similis|ZHAK1126-19|Canada|BOLD:ACK9027
Oithona sp.|GBMNE69050-22|United States|BOLD:ACK9027
Oithona similis|ZHAK1130-19|Canada|BOLD:ACK9027
Oithona similis|ZHAK1128-19|Canada|BOLD:ACK9027
Oithona similis|ZHAK1132-19|Canada|BOLD:ACK9027
Oithona similis|CAISN1378-13|Canada|BOLD:ACK9027
Oithona similis|CAISN218-12|Canada|BOLD:ACK9027
Oithona sp.|GBMNE69048-22|United States|BOLD:ACK9027
Oithona sp.|GBMNE69047-22|United States|BOLD:ACK9027
Oithona sp.|GBMNE69046-22|United States|BOLD:ACK9027
Oithona sp.|GBMNE69045-22|United States|BOLD:ACK9027
Oithona sp.|GBMNE69049-22|United States|BOLD:ACK9027
Oithona sp.|GBMNE69051-22|United States|BOLD:ACK9027
Oithona similis|QHAK3424-23|Canada|BOLD:ACK9027
