# Supplementary material for: DNA barcoding of North American freshwater copepods (Diaptomidae and Cyclopoida): an overview after 20 years with emphasis in the Mexican fauna, the transition between the Nearctic and Neotropics
Source: PeerJ. 2026 Apr 9;14:e20989. doi: 10.7717/peerj.20989 (PMC13070316; doi:10.7717/peerj.20989)
Supplement: Supplemental Information 3 — The colored bars indicate each subset. The lowest score is represented in Rank 1 (third group from right to left). The proposed ID tree is on the right side of the bars. Each terminal branch includes same data as Supplementary Figure 1 [file peerj-14-20989-s003.pdf]

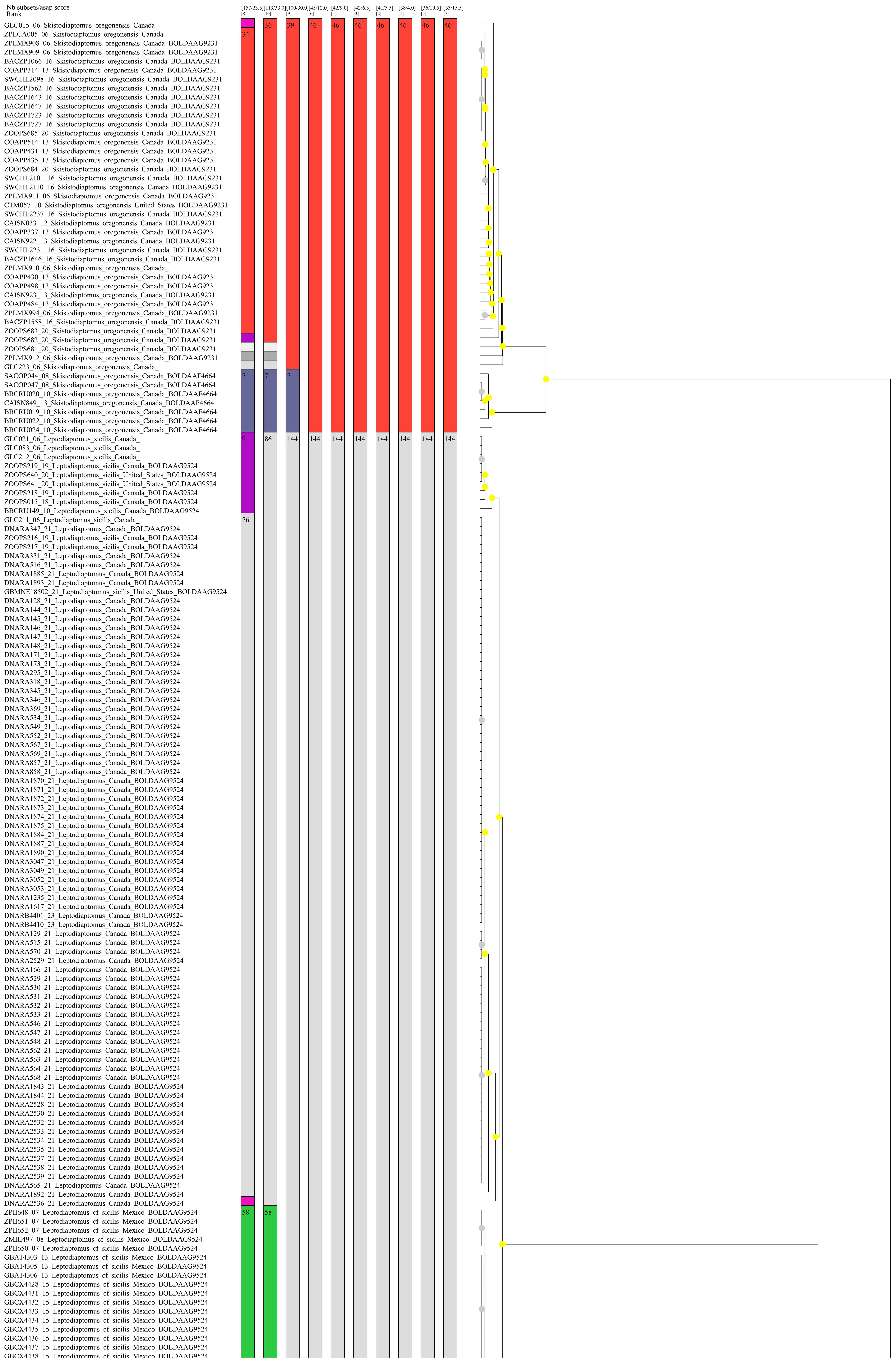
Nb subsets/asap score
Rank
[157/23.5] [119/33.0] [100/30.0] [45/12.0] [42/9.0] [42/6.5] [41/5.5] [38/4.0] [36/10.5] [33/15.5]
[8] [10] [9] [6] [4] [3] [2] [1] [5] [7]
36 39 46 46 46 46 46 46 46
34
7 7 7
9 86 144 144 144 144 144 144 144 144
76
58 58
GLC015_06_Skistodiaptomus_oregonensis_Canada_
ZPLCA005_06_Skistodiaptomus_oregonensis_Canada_
ZPLMX908_06_Skistodiaptomus_oregonensis_Canada_BOLDAAG9231
ZPLMX909_06_Skistodiaptomus_oregonensis_Canada_BOLDAAG9231
BACZP1066_16_Skistodiaptomus_oregonensis_Canada_BOLDAAG9231
COAPP314_13_Skistodiaptomus_oregonensis_Canada_BOLDAAG9231
SWCHL2098_16_Skistodiaptomus_oregonensis_Canada_BOLDAAG9231
BACZP1562_16_Skistodiaptomus_oregonensis_Canada_BOLDAAG9231
BACZP1643_16_Skistodiaptomus_oregonensis_Canada_BOLDAAG9231
BACZP1647_16_Skistodiaptomus_oregonensis_Canada_BOLDAAG9231
BACZP1723_16_Skistodiaptomus_oregonensis_Canada_BOLDAAG9231
BACZP1727_16_Skistodiaptomus_oregonensis_Canada_BOLDAAG9231
ZOOPS685_20_Skistodiaptomus_oregonensis_Canada_BOLDAAG9231
COAPP514_13_Skistodiaptomus_oregonensis_Canada_BOLDAAG9231
COAPP431_13_Skistodiaptomus_oregonensis_Canada_BOLDAAG9231
COAPP435_13_Skistodiaptomus_oregonensis_Canada_BOLDAAG9231
ZOOPS684_20_Skistodiaptomus_oregonensis_Canada_BOLDAAG9231
SWCHL2101_16_Skistodiaptomus_oregonensis_Canada_BOLDAAG9231
SWCHL2110_16_Skistodiaptomus_oregonensis_Canada_BOLDAAG9231
ZPLMX911_06_Skistodiaptomus_oregonensis_Canada_BOLDAAG9231
CTM057_10_Skistodiaptomus_oregonensis_United_States_BOLDAAG9231
SWCHL2237_16_Skistodiaptomus_oregonensis_Canada_BOLDAAG9231
CAISN033_12_Skistodiaptomus_oregonensis_Canada_BOLDAAG9231
COAPP337_13_Skistodiaptomus_oregonensis_Canada_BOLDAAG9231
CAISN922_13_Skistodiaptomus_oregonensis_Canada_BOLDAAG9231
SWCHL2231_16_Skistodiaptomus_oregonensis_Canada_BOLDAAG9231
BACZP1646_16_Skistodiaptomus_oregonensis_Canada_BOLDAAG9231
ZPLMX910_06_Skistodiaptomus_oregonensis_Canada_
COAPP430_13_Skistodiaptomus_oregonensis_Canada_BOLDAAG9231
COAPP498_13_Skistodiaptomus_oregonensis_Canada_BOLDAAG9231
CAISN923_13_Skistodiaptomus_oregonensis_Canada_BOLDAAG9231
COAPP484_13_Skistodiaptomus_oregonensis_Canada_BOLDAAG9231
ZPLMX994_06_Skistodiaptomus_oregonensis_Canada_BOLDAAG9231
BACZP1558_16_Skistodiaptomus_oregonensis_Canada_BOLDAAG9231
ZOOPS683_20_Skistodiaptomus_oregonensis_Canada_BOLDAAG9231
ZOOPS682_20_Skistodiaptomus_oregonensis_Canada_BOLDAAG9231
ZOOPS681_20_Skistodiaptomus_oregonensis_Canada_BOLDAAG9231
ZPLMX912_06_Skistodiaptomus_oregonensis_Canada_BOLDAAG9231
GLC223_06_Skistodiaptomus_oregonensis_Canada_
SACOP044_08_Skistodiaptomus_oregonensis_Canada_BOLDAAF4664
SACOP047_08_Skistodiaptomus_oregonensis_Canada_BOLDAAF4664
BBCRU020_10_Skistodiaptomus_oregonensis_Canada_BOLDAAF4664
CAISN849_13_Skistodiaptomus_oregonensis_Canada_BOLDAAF4664
BBCRU019_10_Skistodiaptomus_oregonensis_Canada_BOLDAAF4664
BBCRU022_10_Skistodiaptomus_oregonensis_Canada_BOLDAAF4664
BBCRU024_10_Skistodiaptomus_oregonensis_Canada_BOLDAAF4664
GLC021_06_Leptodiaptomus_sicilis_Canada_
GLC083_06_Leptodiaptomus_sicilis_Canada_
GLC212_06_Leptodiaptomus_sicilis_Canada_
ZOOPS219_19_Leptodiaptomus_sicilis_Canada_BOLDAAG9524
ZOOPS640_20_Leptodiaptomus_sicilis_United_States_BOLDAAG9524
ZOOPS641_20_Leptodiaptomus_sicilis_United_States_BOLDAAG9524
ZOOPS218_19_Leptodiaptomus_sicilis_Canada_BOLDAAG9524
ZOOPS015_18_Leptodiaptomus_sicilis_Canada_BOLDAAG9524
BBCRU149_10_Leptodiaptomus_sicilis_Canada_BOLDAAG9524
GLC211_06_Leptodiaptomus_sicilis_Canada_
DNARA347_21_Leptodiaptomus_Canada_BOLDAAG9524
ZOOPS216_19_Leptodiaptomus_sicilis_Canada_BOLDAAG9524
ZOOPS217_19_Leptodiaptomus_sicilis_Canada_BOLDAAG9524
DNARA331_21_Leptodiaptomus_Canada_BOLDAAG9524
DNARA516_21_Leptodiaptomus_Canada_BOLDAAG9524
DNARA1885_21_Leptodiaptomus_Canada_BOLDAAG9524
DNARA1893_21_Leptodiaptomus_Canada_BOLDAAG9524
GBMNE18502_21_Leptodiaptomus_sicilis_United_States_BOLDAAG9524
DNARA128_21_Leptodiaptomus_Canada_BOLDAAG9524
DNARA144_21_Leptodiaptomus_Canada_BOLDAAG9524
DNARA145_21_Leptodiaptomus_Canada_BOLDAAG9524
DNARA146_21_Leptodiaptomus_Canada_BOLDAAG9524
DNARA147_21_Leptodiaptomus_Canada_BOLDAAG9524
DNARA148_21_Leptodiaptomus_Canada_BOLDAAG9524
DNARA171_21_Leptodiaptomus_Canada_BOLDAAG9524
DNARA173_21_Leptodiaptomus_Canada_BOLDAAG9524
DNARA295_21_Leptodiaptomus_Canada_BOLDAAG9524
DNARA318_21_Leptodiaptomus_Canada_BOLDAAG9524
DNARA345_21_Leptodiaptomus_Canada_BOLDAAG9524
DNARA346_21_Leptodiaptomus_Canada_BOLDAAG9524
DNARA369_21_Leptodiaptomus_Canada_BOLDAAG9524
DNARA534_21_Leptodiaptomus_Canada_BOLDAAG9524
DNARA549_21_Leptodiaptomus_Canada_BOLDAAG9524
DNARA552_21_Leptodiaptomus_Canada_BOLDAAG9524
DNARA567_21_Leptodiaptomus_Canada_BOLDAAG9524
DNARA569_21_Leptodiaptomus_Canada_BOLDAAG9524
DNARA857_21_Leptodiaptomus_Canada_BOLDAAG9524
DNARA858_21_Leptodiaptomus_Canada_BOLDAAG9524
DNARA1870_21_Leptodiaptomus_Canada_BOLDAAG9524
DNARA1871_21_Leptodiaptomus_Canada_BOLDAAG9524
DNARA1872_21_Leptodiaptomus_Canada_BOLDAAG9524
DNARA1873_21_Leptodiaptomus_Canada_BOLDAAG9524
DNARA1874_21_Leptodiaptomus_Canada_BOLDAAG9524
DNARA1875_21_Leptodiaptomus_Canada_BOLDAAG9524
DNARA1884_21_Leptodiaptomus_Canada_BOLDAAG9524
DNARA1887_21_Leptodiaptomus_Canada_BOLDAAG9524
DNARA1890_21_Leptodiaptomus_Canada_BOLDAAG9524
DNARA3047_21_Leptodiaptomus_Canada_BOLDAAG9524
DNARA3049_21_Leptodiaptomus_Canada_BOLDAAG9524
DNARA3052_21_Leptodiaptomus_Canada_BOLDAAG9524
DNARA3053_21_Leptodiaptomus_Canada_BOLDAAG9524
DNARA1235_21_Leptodiaptomus_Canada_BOLDAAG9524
DNARA1617_21_Leptodiaptomus_Canada_BOLDAAG9524
DNARB4401_23_Leptodiaptomus_Canada_BOLDAAG9524
DNARB4410_23_Leptodiaptomus_Canada_BOLDAAG9524
DNARA129_21_Leptodiaptomus_Canada_BOLDAAG9524
DNARA515_21_Leptodiaptomus_Canada_BOLDAAG9524
DNARA570_21_Leptodiaptomus_Canada_BOLDAAG9524
DNARA2529_21_Leptodiaptomus_Canada_BOLDAAG9524
DNARA166_21_Leptodiaptomus_Canada_BOLDAAG9524
DNARA529_21_Leptodiaptomus_Canada_BOLDAAG9524
DNARA530_21_Leptodiaptomus_Canada_BOLDAAG9524
DNARA531_21_Leptodiaptomus_Canada_BOLDAAG9524
DNARA532_21_Leptodiaptomus_Canada_BOLDAAG9524
DNARA533_21_Leptodiaptomus_Canada_BOLDAAG9524
DNARA546_21_Leptodiaptomus_Canada_BOLDAAG9524
DNARA547_21_Leptodiaptomus_Canada_BOLDAAG9524
DNARA548_21_Leptodiaptomus_Canada_BOLDAAG9524
DNARA562_21_Leptodiaptomus_Canada_BOLDAAG9524
DNARA563_21_Leptodiaptomus_Canada_BOLDAAG9524
DNARA564_21_Leptodiaptomus_Canada_BOLDAAG9524
DNARA568_21_Leptodiaptomus_Canada_BOLDAAG9524
DNARA1843_21_Leptodiaptomus_Canada_BOLDAAG9524
DNARA1844_21_Leptodiaptomus_Canada_BOLDAAG9524
DNARA2528_21_Leptodiaptomus_Canada_BOLDAAG9524
DNARA2530_21_Leptodiaptomus_Canada_BOLDAAG9524
DNARA2532_21_Leptodiaptomus_Canada_BOLDAAG9524
DNARA2533_21_Leptodiaptomus_Canada_BOLDAAG9524
DNARA2534_21_Leptodiaptomus_Canada_BOLDAAG9524
DNARA2535_21_Leptodiaptomus_Canada_BOLDAAG9524
DNARA2537_21_Leptodiaptomus_Canada_BOLDAAG9524
DNARA2538_21_Leptodiaptomus_Canada_BOLDAAG9524
DNARA2539_21_Leptodiaptomus_Canada_BOLDAAG9524
DNARA565_21_Leptodiaptomus_Canada_BOLDAAG9524
DNARA1892_21_Leptodiaptomus_Canada_BOLDAAG9524
DNARA2536_21_Leptodiaptomus_Canada_BOLDAAG9524
ZPII648_07_Leptodiaptomus_cf_sicilis_Mexico_BOLDAAG9524
ZPII651_07_Leptodiaptomus_cf_sicilis_Mexico_BOLDAAG9524
ZPII652_07_Leptodiaptomus_cf_sicilis_Mexico_BOLDAAG9524
ZMIII497_08_Leptodiaptomus_cf_sicilis_Mexico_BOLDAAG9524
ZPII650_07_Leptodiaptomus_cf_sicilis_Mexico_BOLDAAG9524
GBA14303_13_Leptodiaptomus_cf_sicilis_Mexico_BOLDAAG9524
GBA14305_13_Leptodiaptomus_cf_sicilis_Mexico_BOLDAAG9524
GBA14306_13_Leptodiaptomus_cf_sicilis_Mexico_BOLDAAG9524
GBCX4428_15_Leptodiaptomus_cf_sicilis_Mexico_BOLDAAG9524
GBCX4431_15_Leptodiaptomus_cf_sicilis_Mexico_BOLDAAG9524
GBCX4432_15_Leptodiaptomus_cf_sicilis_Mexico_BOLDAAG9524
GBCX4433_15_Leptodiaptomus_cf_sicilis_Mexico_BOLDAAG9524
GBCX4434_15_Leptodiaptomus_cf_sicilis_Mexico_BOLDAAG9524
GBCX4435_15_Leptodiaptomus_cf_sicilis_Mexico_BOLDAAG9524
GBCX4436_15_Leptodiaptomus_cf_sicilis_Mexico_BOLDAAG9524
GBCX4437_15_Leptodiaptomus_cf_sicilis_Mexico_BOLDAAG9524

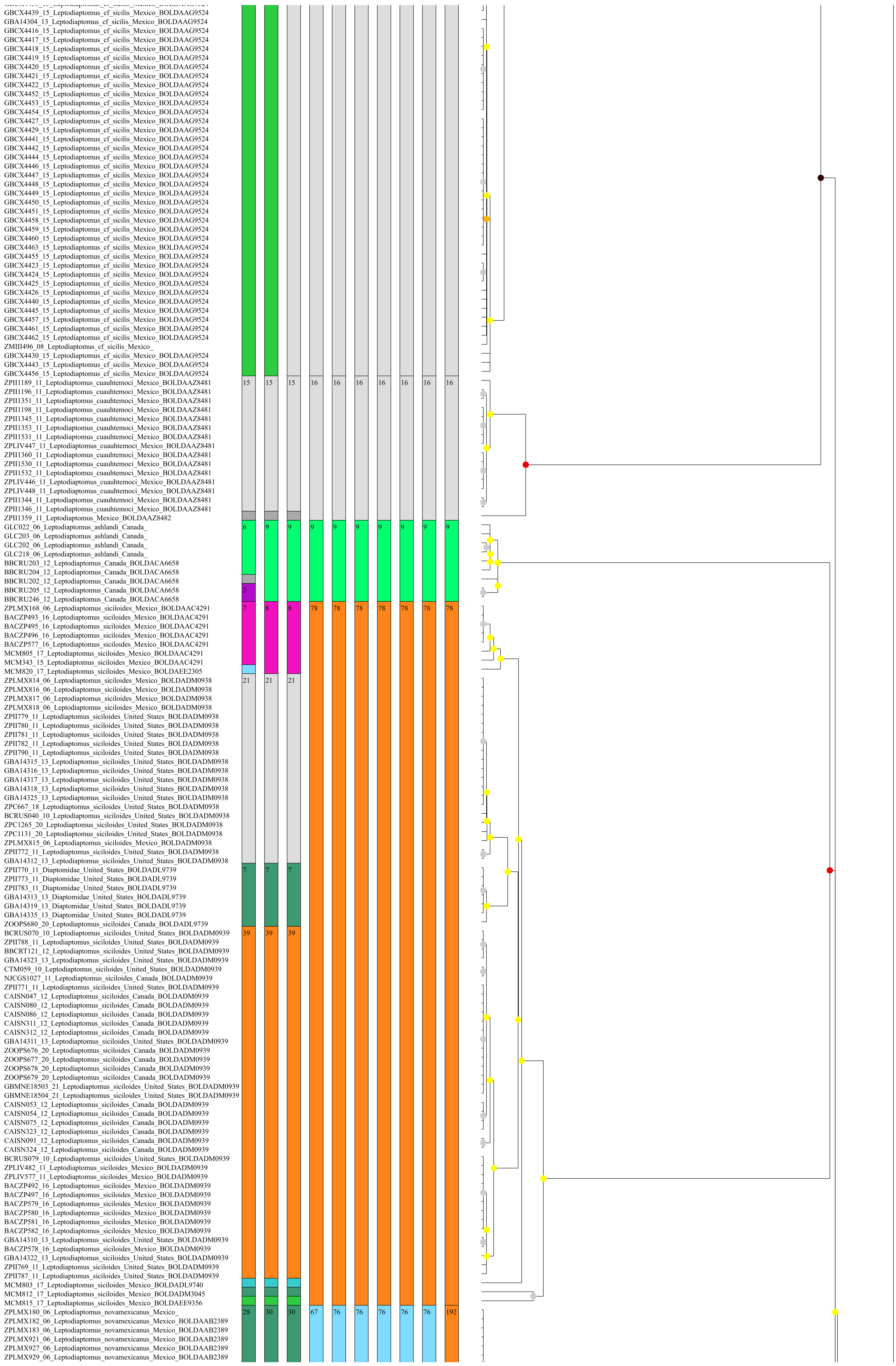

GBCX4439_15_Leptodiaptomus_cf_sicilis_Mexico_BOLDAAG9524
GBA14304_13_Leptodiaptomus_cf_sicilis_Mexico_BOLDAAG9524
GBCX4416_15_Leptodiaptomus_cf_sicilis_Mexico_BOLDAAG9524
GBCX4417_15_Leptodiaptomus_cf_sicilis_Mexico_BOLDAAG9524
GBCX4418_15_Leptodiaptomus_cf_sicilis_Mexico_BOLDAAG9524
GBCX4419_15_Leptodiaptomus_cf_sicilis_Mexico_BOLDAAG9524
GBCX4420_15_Leptodiaptomus_cf_sicilis_Mexico_BOLDAAG9524
GBCX4421_15_Leptodiaptomus_cf_sicilis_Mexico_BOLDAAG9524
GBCX4422_15_Leptodiaptomus_cf_sicilis_Mexico_BOLDAAG9524
GBCX4452_15_Leptodiaptomus_cf_sicilis_Mexico_BOLDAAG9524
GBCX4453_15_Leptodiaptomus_cf_sicilis_Mexico_BOLDAAG9524
GBCX4454_15_Leptodiaptomus_cf_sicilis_Mexico_BOLDAAG9524
GBCX4427_15_Leptodiaptomus_cf_sicilis_Mexico_BOLDAAG9524
GBCX4429_15_Leptodiaptomus_cf_sicilis_Mexico_BOLDAAG9524
GBCX4441_15_Leptodiaptomus_cf_sicilis_Mexico_BOLDAAG9524
GBCX4442_15_Leptodiaptomus_cf_sicilis_Mexico_BOLDAAG9524
GBCX4444_15_Leptodiaptomus_cf_sicilis_Mexico_BOLDAAG9524
GBCX4446_15_Leptodiaptomus_cf_sicilis_Mexico_BOLDAAG9524
GBCX4447_15_Leptodiaptomus_cf_sicilis_Mexico_BOLDAAG9524
GBCX4448_15_Leptodiaptomus_cf_sicilis_Mexico_BOLDAAG9524
GBCX4449_15_Leptodiaptomus_cf_sicilis_Mexico_BOLDAAG9524
GBCX4450_15_Leptodiaptomus_cf_sicilis_Mexico_BOLDAAG9524
GBCX4451_15_Leptodiaptomus_cf_sicilis_Mexico_BOLDAAG9524
GBCX4458_15_Leptodiaptomus_cf_sicilis_Mexico_BOLDAAG9524
GBCX4459_15_Leptodiaptomus_cf_sicilis_Mexico_BOLDAAG9524
GBCX4460_15_Leptodiaptomus_cf_sicilis_Mexico_BOLDAAG9524
GBCX4463_15_Leptodiaptomus_cf_sicilis_Mexico_BOLDAAG9524
GBCX4455_15_Leptodiaptomus_cf_sicilis_Mexico_BOLDAAG9524
GBCX4423_15_Leptodiaptomus_cf_sicilis_Mexico_BOLDAAG9524
GBCX4424_15_Leptodiaptomus_cf_sicilis_Mexico_BOLDAAG9524
GBCX4425_15_Leptodiaptomus_cf_sicilis_Mexico_BOLDAAG9524
GBCX4426_15_Leptodiaptomus_cf_sicilis_Mexico_BOLDAAG9524
GBCX4440_15_Leptodiaptomus_cf_sicilis_Mexico_BOLDAAG9524
GBCX4445_15_Leptodiaptomus_cf_sicilis_Mexico_BOLDAAG9524
GBCX4457_15_Leptodiaptomus_cf_sicilis_Mexico_BOLDAAG9524
GBCX4461_15_Leptodiaptomus_cf_sicilis_Mexico_BOLDAAG9524
GBCX4462_15_Leptodiaptomus_cf_sicilis_Mexico_BOLDAAG9524
ZMIII496_08_Leptodiaptomus_cf_sicilis_Mexico_
GBCX4430_15_Leptodiaptomus_cf_sicilis_Mexico_BOLDAAG9524
GBCX4443_15_Leptodiaptomus_cf_sicilis_Mexico_BOLDAAG9524
GBCX4456_15_Leptodiaptomus_cf_sicilis_Mexico_BOLDAAG9524
ZPII1189_11_Leptodiaptomus_cuauhtemoci_Mexico_BOLDAAZ8481
ZPII1196_11_Leptodiaptomus_cuauhtemoci_Mexico_BOLDAAZ8481
ZPII1351_11_Leptodiaptomus_cuauhtemoci_Mexico_BOLDAAZ8481
ZPII1198_11_Leptodiaptomus_cuauhtemoci_Mexico_BOLDAAZ8481
ZPII1345_11_Leptodiaptomus_cuauhtemoci_Mexico_BOLDAAZ8481
ZPII1353_11_Leptodiaptomus_cuauhtemoci_Mexico_BOLDAAZ8481
ZPII1531_11_Leptodiaptomus_cuauhtemoci_Mexico_BOLDAAZ8481
ZPLIV447_11_Leptodiaptomus_cuauhtemoci_Mexico_BOLDAAZ8481
ZPII1360_11_Leptodiaptomus_cuauhtemoci_Mexico_BOLDAAZ8481
ZPII1530_11_Leptodiaptomus_cuauhtemoci_Mexico_BOLDAAZ8481
ZPII1532_11_Leptodiaptomus_cuauhtemoci_Mexico_BOLDAAZ8481
ZPLIV446_11_Leptodiaptomus_cuauhtemoci_Mexico_BOLDAAZ8481
ZPLIV448_11_Leptodiaptomus_cuauhtemoci_Mexico_BOLDAAZ8481
ZPII1344_11_Leptodiaptomus_cuauhtemoci_Mexico_BOLDAAZ8481
ZPII1346_11_Leptodiaptomus_cuauhtemoci_Mexico_BOLDAAZ8481
ZPII1359_11_Leptodiaptomus_Mexico_BOLDAAZ8482
GLC022_06_Leptodiaptomus_ashlandi_Canada_
GLC203_06_Leptodiaptomus_ashlandi_Canada_
GLC202_06_Leptodiaptomus_ashlandi_Canada_
GLC218_06_Leptodiaptomus_ashlandi_Canada_
BBCRU203_12_Leptodiaptomus_Canada_BOLDACA6658
BBCRU204_12_Leptodiaptomus_Canada_BOLDACA6658
BBCRU202_12_Leptodiaptomus_Canada_BOLDACA6658
BBCRU205_12_Leptodiaptomus_Canada_BOLDACA6658
BBCRU246_12_Leptodiaptomus_Canada_BOLDACA6658
ZPLMX168_06_Leptodiaptomus_siciloides_Mexico_BOLDAAC4291
BACZP493_16_Leptodiaptomus_siciloides_Mexico_BOLDAAC4291
BACZP495_16_Leptodiaptomus_siciloides_Mexico_BOLDAAC4291
BACZP496_16_Leptodiaptomus_siciloides_Mexico_BOLDAAC4291
BACZP577_16_Leptodiaptomus_siciloides_Mexico_BOLDAAC4291
MCM805_17_Leptodiaptomus_siciloides_Mexico_BOLDAAC4291
MCM343_15_Leptodiaptomus_siciloides_Mexico_BOLDAAC4291
MCM820_17_Leptodiaptomus_siciloides_Mexico_BOLDAEE2305
ZPLMX814_06_Leptodiaptomus_siciloides_Mexico_BOLDADM0938
ZPLMX816_06_Leptodiaptomus_siciloides_Mexico_BOLDADM0938
ZPLMX817_06_Leptodiaptomus_siciloides_Mexico_BOLDADM0938
ZPLMX818_06_Leptodiaptomus_siciloides_Mexico_BOLDADM0938
ZPII779_11_Leptodiaptomus_siciloides_United_States_BOLDADM0938
ZPII780_11_Leptodiaptomus_siciloides_United_States_BOLDADM0938
ZPII781_11_Leptodiaptomus_siciloides_United_States_BOLDADM0938
ZPII782_11_Leptodiaptomus_siciloides_United_States_BOLDADM0938
ZPII790_11_Leptodiaptomus_siciloides_United_States_BOLDADM0938
GBA14315_13_Leptodiaptomus_siciloides_United_States_BOLDADM0938
GBA14316_13_Leptodiaptomus_siciloides_United_States_BOLDADM0938
GBA14317_13_Leptodiaptomus_siciloides_United_States_BOLDADM0938
GBA14318_13_Leptodiaptomus_siciloides_United_States_BOLDADM0938
GBA14325_13_Leptodiaptomus_siciloides_United_States_BOLDADM0938
ZPC667_18_Leptodiaptomus_siciloides_United_States_BOLDADM0938
BCRUS040_10_Leptodiaptomus_siciloides_United_States_BOLDADM0938
ZPC1265_20_Leptodiaptomus_siciloides_United_States_BOLDADM0938
ZPC1131_20_Leptodiaptomus_siciloides_United_States_BOLDADM0938
ZPLMX815_06_Leptodiaptomus_siciloides_Mexico_BOLDADM0938
ZPII772_11_Leptodiaptomus_siciloides_United_States_BOLDADM0938
GBA14312_13_Leptodiaptomus_siciloides_United_States_BOLDADM0938
ZPII770_11_Diaptomidae_United_States_BOLDADL9739
ZPII773_11_Diaptomidae_United_States_BOLDADL9739
ZPII783_11_Diaptomidae_United_States_BOLDADL9739
GBA14313_13_Diaptomidae_United_States_BOLDADL9739
GBA14319_13_Diaptomidae_United_States_BOLDADL9739
GBA14335_13_Diaptomidae_United_States_BOLDADL9739
ZOOPS680_20_Leptodiaptomus_siciloides_Canada_BOLDADL9739
BCRUS070_10_Leptodiaptomus_siciloides_United_States_BOLDADM0939
ZPII788_11_Leptodiaptomus_siciloides_United_States_BOLDADM0939
BBCRT121_12_Leptodiaptomus_siciloides_United_States_BOLDADM0939
GBA14323_13_Leptodiaptomus_siciloides_United_States_BOLDADM0939
CTM059_10_Leptodiaptomus_siciloides_United_States_BOLDADM0939
NJCGS1027_11_Leptodiaptomus_siciloides_Canada_BOLDADM0939
ZPII771_11_Leptodiaptomus_siciloides_United_States_BOLDADM0939
CAISN047_12_Leptodiaptomus_siciloides_Canada_BOLDADM0939
CAISN080_12_Leptodiaptomus_siciloides_Canada_BOLDADM0939
CAISN086_12_Leptodiaptomus_siciloides_Canada_BOLDADM0939
CAISN311_12_Leptodiaptomus_siciloides_Canada_BOLDADM0939
CAISN312_12_Leptodiaptomus_siciloides_Canada_BOLDADM0939
GBA14311_13_Leptodiaptomus_siciloides_United_States_BOLDADM0939
ZOOPS676_20_Leptodiaptomus_siciloides_Canada_BOLDADM0939
ZOOPS677_20_Leptodiaptomus_siciloides_Canada_BOLDADM0939
ZOOPS678_20_Leptodiaptomus_siciloides_Canada_BOLDADM0939
ZOOPS679_20_Leptodiaptomus_siciloides_Canada_BOLDADM0939
GBMNE18503_21_Leptodiaptomus_siciloides_United_States_BOLDADM0939
GBMNE18504_21_Leptodiaptomus_siciloides_United_States_BOLDADM0939
CAISN053_12_Leptodiaptomus_siciloides_Canada_BOLDADM0939
CAISN054_12_Leptodiaptomus_siciloides_Canada_BOLDADM0939
CAISN075_12_Leptodiaptomus_siciloides_Canada_BOLDADM0939
CAISN323_12_Leptodiaptomus_siciloides_Canada_BOLDADM0939
CAISN091_12_Leptodiaptomus_siciloides_Canada_BOLDADM0939
CAISN324_12_Leptodiaptomus_siciloides_Canada_BOLDADM0939
BCRUS079_10_Leptodiaptomus_siciloides_United_States_BOLDADM0939
ZPLIV482_11_Leptodiaptomus_siciloides_Mexico_BOLDADM0939
ZPLIV577_11_Leptodiaptomus_siciloides_Mexico_BOLDADM0939
BACZP492_16_Leptodiaptomus_siciloides_Mexico_BOLDADM0939
BACZP497_16_Leptodiaptomus_siciloides_Mexico_BOLDADM0939
BACZP579_16_Leptodiaptomus_siciloides_Mexico_BOLDADM0939
BACZP580_16_Leptodiaptomus_siciloides_Mexico_BOLDADM0939
BACZP581_16_Leptodiaptomus_siciloides_Mexico_BOLDADM0939
BACZP582_16_Leptodiaptomus_siciloides_Mexico_BOLDADM0939
GBA14310_13_Leptodiaptomus_siciloides_United_States_BOLDADM0939
BACZP578_16_Leptodiaptomus_siciloides_Mexico_BOLDADM0939
GBA14322_13_Leptodiaptomus_siciloides_United_States_BOLDADM0939
ZPII769_11_Leptodiaptomus_siciloides_United_States_BOLDADM0939
ZPII787_11_Leptodiaptomus_siciloides_United_States_BOLDADM0939
MCM803_17_Leptodiaptomus_siciloides_Mexico_BOLDADL9740
MCM812_17_Leptodiaptomus_siciloides_Mexico_BOLDADM3045
MCM815_17_Leptodiaptomus_siciloides_Mexico_BOLDAEE9356
ZPLMX180_06_Leptodiaptomus_novamexicanus_Mexico_
ZPLMX182_06_Leptodiaptomus_novamexicanus_Mexico_BOLDAAB2389
ZPLMX183_06_Leptodiaptomus_novamexicanus_Mexico_BOLDAAB2389
ZPLMX921_06_Leptodiaptomus_novamexicanus_Mexico_BOLDAAB2389
ZPLMX927_06_Leptodiaptomus_novamexicanus_Mexico_BOLDAAB2389
ZPLMX929_06_Leptodiaptomus_novamexicanus_Mexico_BOLDAAB2389

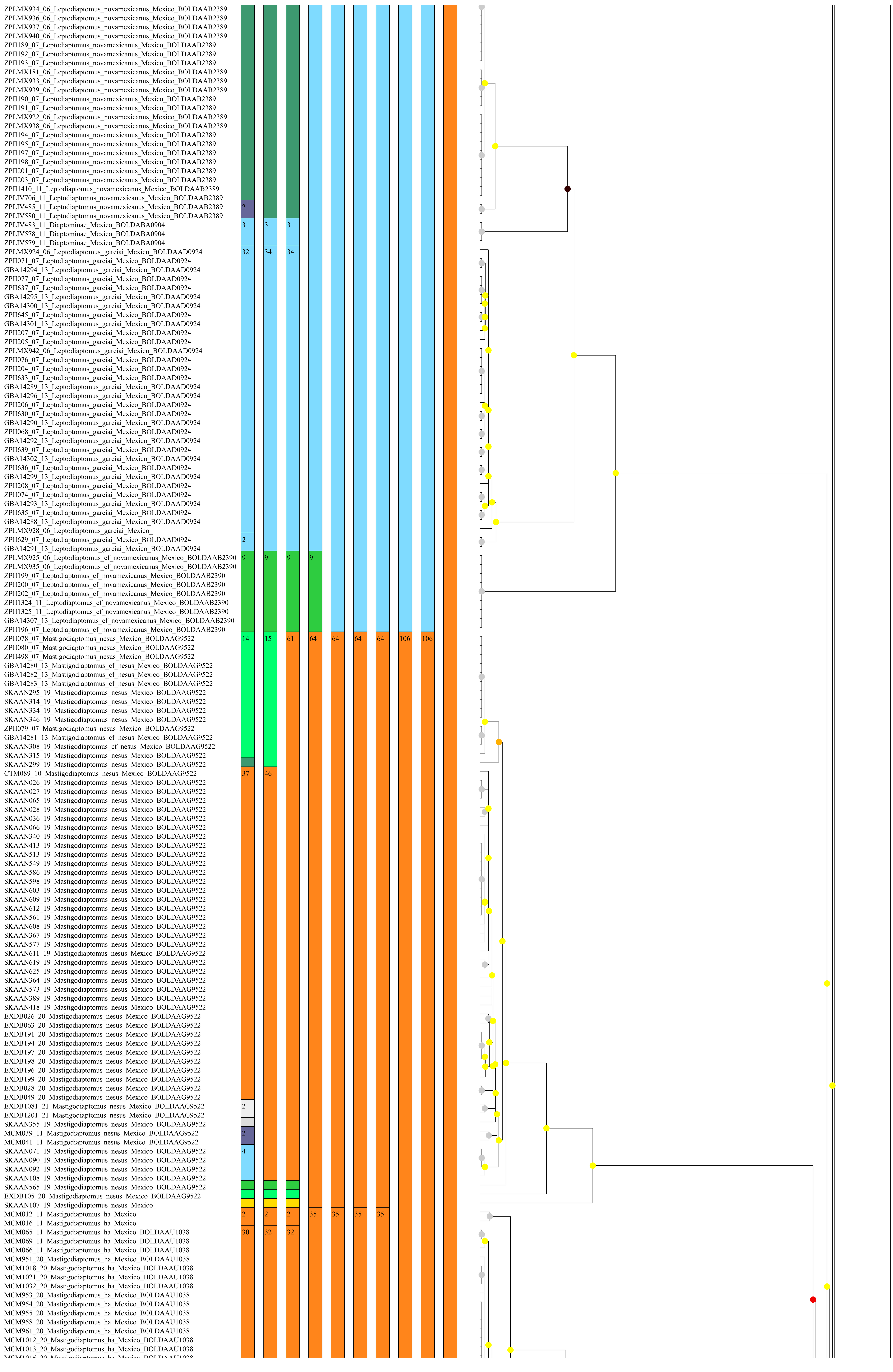
ZPLMX934_06_Leptodiaptomus_novamexicanus_Mexico_BOLDAAB2389
ZPLMX936_06_Leptodiaptomus_novamexicanus_Mexico_BOLDAAB2389
ZPLMX937_06_Leptodiaptomus_novamexicanus_Mexico_BOLDAAB2389
ZPLMX940_06_Leptodiaptomus_novamexicanus_Mexico_BOLDAAB2389
ZPII189_07_Leptodiaptomus_novamexicanus_Mexico_BOLDAAB2389
ZPII192_07_Leptodiaptomus_novamexicanus_Mexico_BOLDAAB2389
ZPII193_07_Leptodiaptomus_novamexicanus_Mexico_BOLDAAB2389
ZPLMX181_06_Leptodiaptomus_novamexicanus_Mexico_BOLDAAB2389
ZPLMX933_06_Leptodiaptomus_novamexicanus_Mexico_BOLDAAB2389
ZPLMX939_06_Leptodiaptomus_novamexicanus_Mexico_BOLDAAB2389
ZPII190_07_Leptodiaptomus_novamexicanus_Mexico_BOLDAAB2389
ZPII191_07_Leptodiaptomus_novamexicanus_Mexico_BOLDAAB2389
ZPLMX922_06_Leptodiaptomus_novamexicanus_Mexico_BOLDAAB2389
ZPLMX938_06_Leptodiaptomus_novamexicanus_Mexico_BOLDAAB2389
ZPII194_07_Leptodiaptomus_novamexicanus_Mexico_BOLDAAB2389
ZPII195_07_Leptodiaptomus_novamexicanus_Mexico_BOLDAAB2389
ZPII197_07_Leptodiaptomus_novamexicanus_Mexico_BOLDAAB2389
ZPII198_07_Leptodiaptomus_novamexicanus_Mexico_BOLDAAB2389
ZPII201_07_Leptodiaptomus_novamexicanus_Mexico_BOLDAAB2389
ZPII203_07_Leptodiaptomus_novamexicanus_Mexico_BOLDAAB2389
ZPII1410_11_Leptodiaptomus_novamexicanus_Mexico_BOLDAAB2389
ZPLIV706_11_Leptodiaptomus_novamexicanus_Mexico_BOLDAAB2389
ZPLIV485_11_Leptodiaptomus_novamexicanus_Mexico_BOLDAAB2389
ZPLIV580_11_Leptodiaptomus_novamexicanus_Mexico_BOLDAAB2389
ZPLIV483_11_Diaptominae_Mexico_BOLDABA0904
ZPLIV578_11_Diaptominae_Mexico_BOLDABA0904
ZPLIV579_11_Diaptominae_Mexico_BOLDABA0904
ZPLMX924_06_Leptodiaptomus_garciai_Mexico_BOLDAAD0924
ZPII071_07_Leptodiaptomus_garciai_Mexico_BOLDAAD0924
GBA14294_13_Leptodiaptomus_garciai_Mexico_BOLDAAD0924
ZPII077_07_Leptodiaptomus_garciai_Mexico_BOLDAAD0924
ZPII637_07_Leptodiaptomus_garciai_Mexico_BOLDAAD0924
GBA14295_13_Leptodiaptomus_garciai_Mexico_BOLDAAD0924
GBA14300_13_Leptodiaptomus_garciai_Mexico_BOLDAAD0924
ZPII645_07_Leptodiaptomus_garciai_Mexico_BOLDAAD0924
GBA14301_13_Leptodiaptomus_garciai_Mexico_BOLDAAD0924
ZPII207_07_Leptodiaptomus_garciai_Mexico_BOLDAAD0924
ZPII205_07_Leptodiaptomus_garciai_Mexico_BOLDAAD0924
ZPLMX942_06_Leptodiaptomus_garciai_Mexico_BOLDAAD0924
ZPII076_07_Leptodiaptomus_garciai_Mexico_BOLDAAD0924
ZPII204_07_Leptodiaptomus_garciai_Mexico_BOLDAAD0924
ZPII633_07_Leptodiaptomus_garciai_Mexico_BOLDAAD0924
GBA14289_13_Leptodiaptomus_garciai_Mexico_BOLDAAD0924
GBA14296_13_Leptodiaptomus_garciai_Mexico_BOLDAAD0924
ZPII206_07_Leptodiaptomus_garciai_Mexico_BOLDAAD0924
ZPII630_07_Leptodiaptomus_garciai_Mexico_BOLDAAD0924
GBA14290_13_Leptodiaptomus_garciai_Mexico_BOLDAAD0924
ZPII068_07_Leptodiaptomus_garciai_Mexico_BOLDAAD0924
GBA14292_13_Leptodiaptomus_garciai_Mexico_BOLDAAD0924
ZPII639_07_Leptodiaptomus_garciai_Mexico_BOLDAAD0924
GBA14302_13_Leptodiaptomus_garciai_Mexico_BOLDAAD0924
ZPII636_07_Leptodiaptomus_garciai_Mexico_BOLDAAD0924
GBA14299_13_Leptodiaptomus_garciai_Mexico_BOLDAAD0924
ZPII208_07_Leptodiaptomus_garciai_Mexico_BOLDAAD0924
ZPII074_07_Leptodiaptomus_garciai_Mexico_BOLDAAD0924
GBA14293_13_Leptodiaptomus_garciai_Mexico_BOLDAAD0924
ZPII635_07_Leptodiaptomus_garciai_Mexico_BOLDAAD0924
GBA14288_13_Leptodiaptomus_garciai_Mexico_BOLDAAD0924
ZPLMX928_06_Leptodiaptomus_garciai_Mexico_
ZPII629_07_Leptodiaptomus_garciai_Mexico_BOLDAAD0924
GBA14291_13_Leptodiaptomus_garciai_Mexico_BOLDAAD0924
ZPLMX925_06_Leptodiaptomus_cf_novamexicanus_Mexico_BOLDAAB2390
ZPLMX935_06_Leptodiaptomus_cf_novamexicanus_Mexico_BOLDAAB2390
ZPII199_07_Leptodiaptomus_cf_novamexicanus_Mexico_BOLDAAB2390
ZPII200_07_Leptodiaptomus_cf_novamexicanus_Mexico_BOLDAAB2390
ZPII202_07_Leptodiaptomus_cf_novamexicanus_Mexico_BOLDAAB2390
ZPII1324_11_Leptodiaptomus_cf_novamexicanus_Mexico_BOLDAAB2390
ZPII1325_11_Leptodiaptomus_cf_novamexicanus_Mexico_BOLDAAB2390
GBA14307_13_Leptodiaptomus_cf_novamexicanus_Mexico_BOLDAAB2390
ZPII196_07_Leptodiaptomus_cf_novamexicanus_Mexico_BOLDAAB2390
ZPII078_07_Mastigodiaptomus_nesus_Mexico_BOLDAAG9522
ZPII080_07_Mastigodiaptomus_nesus_Mexico_BOLDAAG9522
ZPII498_07_Mastigodiaptomus_nesus_Mexico_BOLDAAG9522
GBA14280_13_Mastigodiaptomus_cf_nesus_Mexico_BOLDAAG9522
GBA14282_13_Mastigodiaptomus_cf_nesus_Mexico_BOLDAAG9522
GBA14283_13_Mastigodiaptomus_cf_nesus_Mexico_BOLDAAG9522
SKAAN295_19_Mastigodiaptomus_nesus_Mexico_BOLDAAG9522
SKAAN314_19_Mastigodiaptomus_nesus_Mexico_BOLDAAG9522
SKAAN334_19_Mastigodiaptomus_nesus_Mexico_BOLDAAG9522
SKAAN346_19_Mastigodiaptomus_nesus_Mexico_BOLDAAG9522
ZPII079_07_Mastigodiaptomus_nesus_Mexico_BOLDAAG9522
GBA14281_13_Mastigodiaptomus_cf_nesus_Mexico_BOLDAAG9522
SKAAN308_19_Mastigodiaptomus_cf_nesus_Mexico_BOLDAAG9522
SKAAN315_19_Mastigodiaptomus_nesus_Mexico_BOLDAAG9522
SKAAN299_19_Mastigodiaptomus_nesus_Mexico_BOLDAAG9522
CTM089_10_Mastigodiaptomus_nesus_Mexico_BOLDAAG9522
SKAAN026_19_Mastigodiaptomus_nesus_Mexico_BOLDAAG9522
SKAAN027_19_Mastigodiaptomus_nesus_Mexico_BOLDAAG9522
SKAAN065_19_Mastigodiaptomus_nesus_Mexico_BOLDAAG9522
SKAAN028_19_Mastigodiaptomus_nesus_Mexico_BOLDAAG9522
SKAAN036_19_Mastigodiaptomus_nesus_Mexico_BOLDAAG9522
SKAAN066_19_Mastigodiaptomus_nesus_Mexico_BOLDAAG9522
SKAAN340_19_Mastigodiaptomus_nesus_Mexico_BOLDAAG9522
SKAAN413_19_Mastigodiaptomus_nesus_Mexico_BOLDAAG9522
SKAAN513_19_Mastigodiaptomus_nesus_Mexico_BOLDAAG9522
SKAAN549_19_Mastigodiaptomus_nesus_Mexico_BOLDAAG9522
SKAAN586_19_Mastigodiaptomus_nesus_Mexico_BOLDAAG9522
SKAAN598_19_Mastigodiaptomus_nesus_Mexico_BOLDAAG9522
SKAAN603_19_Mastigodiaptomus_nesus_Mexico_BOLDAAG9522
SKAAN609_19_Mastigodiaptomus_nesus_Mexico_BOLDAAG9522
SKAAN612_19_Mastigodiaptomus_nesus_Mexico_BOLDAAG9522
SKAAN561_19_Mastigodiaptomus_nesus_Mexico_BOLDAAG9522
SKAAN608_19_Mastigodiaptomus_nesus_Mexico_BOLDAAG9522
SKAAN367_19_Mastigodiaptomus_nesus_Mexico_BOLDAAG9522
SKAAN577_19_Mastigodiaptomus_nesus_Mexico_BOLDAAG9522
SKAAN611_19_Mastigodiaptomus_nesus_Mexico_BOLDAAG9522
SKAAN619_19_Mastigodiaptomus_nesus_Mexico_BOLDAAG9522
SKAAN625_19_Mastigodiaptomus_nesus_Mexico_BOLDAAG9522
SKAAN364_19_Mastigodiaptomus_nesus_Mexico_BOLDAAG9522
SKAAN573_19_Mastigodiaptomus_nesus_Mexico_BOLDAAG9522
SKAAN389_19_Mastigodiaptomus_nesus_Mexico_BOLDAAG9522
SKAAN418_19_Mastigodiaptomus_nesus_Mexico_BOLDAAG9522
EXDB026_20_Mastigodiaptomus_nesus_Mexico_BOLDAAG9522
EXDB063_20_Mastigodiaptomus_nesus_Mexico_BOLDAAG9522
EXDB191_20_Mastigodiaptomus_nesus_Mexico_BOLDAAG9522
EXDB194_20_Mastigodiaptomus_nesus_Mexico_BOLDAAG9522
EXDB197_20_Mastigodiaptomus_nesus_Mexico_BOLDAAG9522
EXDB198_20_Mastigodiaptomus_nesus_Mexico_BOLDAAG9522
EXDB196_20_Mastigodiaptomus_nesus_Mexico_BOLDAAG9522
EXDB199_20_Mastigodiaptomus_nesus_Mexico_BOLDAAG9522
EXDB028_20_Mastigodiaptomus_nesus_Mexico_BOLDAAG9522
EXDB049_20_Mastigodiaptomus_nesus_Mexico_BOLDAAG9522
EXDB1081_21_Mastigodiaptomus_nesus_Mexico_BOLDAAG9522
EXDB1201_21_Mastigodiaptomus_nesus_Mexico_BOLDAAG9522
SKAAN355_19_Mastigodiaptomus_nesus_Mexico_BOLDAAG9522
MCM039_11_Mastigodiaptomus_nesus_Mexico_BOLDAAG9522
MCM041_11_Mastigodiaptomus_nesus_Mexico_BOLDAAG9522
SKAAN071_19_Mastigodiaptomus_nesus_Mexico_BOLDAAG9522
SKAAN090_19_Mastigodiaptomus_nesus_Mexico_BOLDAAG9522
SKAAN092_19_Mastigodiaptomus_nesus_Mexico_BOLDAAG9522
SKAAN108_19_Mastigodiaptomus_nesus_Mexico_BOLDAAG9522
SKAAN565_19_Mastigodiaptomus_nesus_Mexico_BOLDAAG9522
EXDB105_20_Mastigodiaptomus_nesus_Mexico_BOLDAAG9522
SKAAN107_19_Mastigodiaptomus_nesus_Mexico_
MCM012_11_Mastigodiaptomus_ha_Mexico_
MCM016_11_Mastigodiaptomus_ha_Mexico_
MCM065_11_Mastigodiaptomus_ha_Mexico_BOLDAAU1038
MCM069_11_Mastigodiaptomus_ha_Mexico_BOLDAAU1038
MCM066_11_Mastigodiaptomus_ha_Mexico_BOLDAAU1038
MCM951_20_Mastigodiaptomus_ha_Mexico_BOLDAAU1038
MCM1018_20_Mastigodiaptomus_ha_Mexico_BOLDAAU1038
MCM1021_20_Mastigodiaptomus_ha_Mexico_BOLDAAU1038
MCM1032_20_Mastigodiaptomus_ha_Mexico_BOLDAAU1038
MCM953_20_Mastigodiaptomus_ha_Mexico_BOLDAAU1038
MCM954_20_Mastigodiaptomus_ha_Mexico_BOLDAAU1038
MCM955_20_Mastigodiaptomus_ha_Mexico_BOLDAAU1038
MCM958_20_Mastigodiaptomus_ha_Mexico_BOLDAAU1038
MCM961_20_Mastigodiaptomus_ha_Mexico_BOLDAAU1038
MCM1012_20_Mastigodiaptomus_ha_Mexico_BOLDAAU1038
MCM1013_20_Mastigodiaptomus_ha_Mexico_BOLDAAU1038

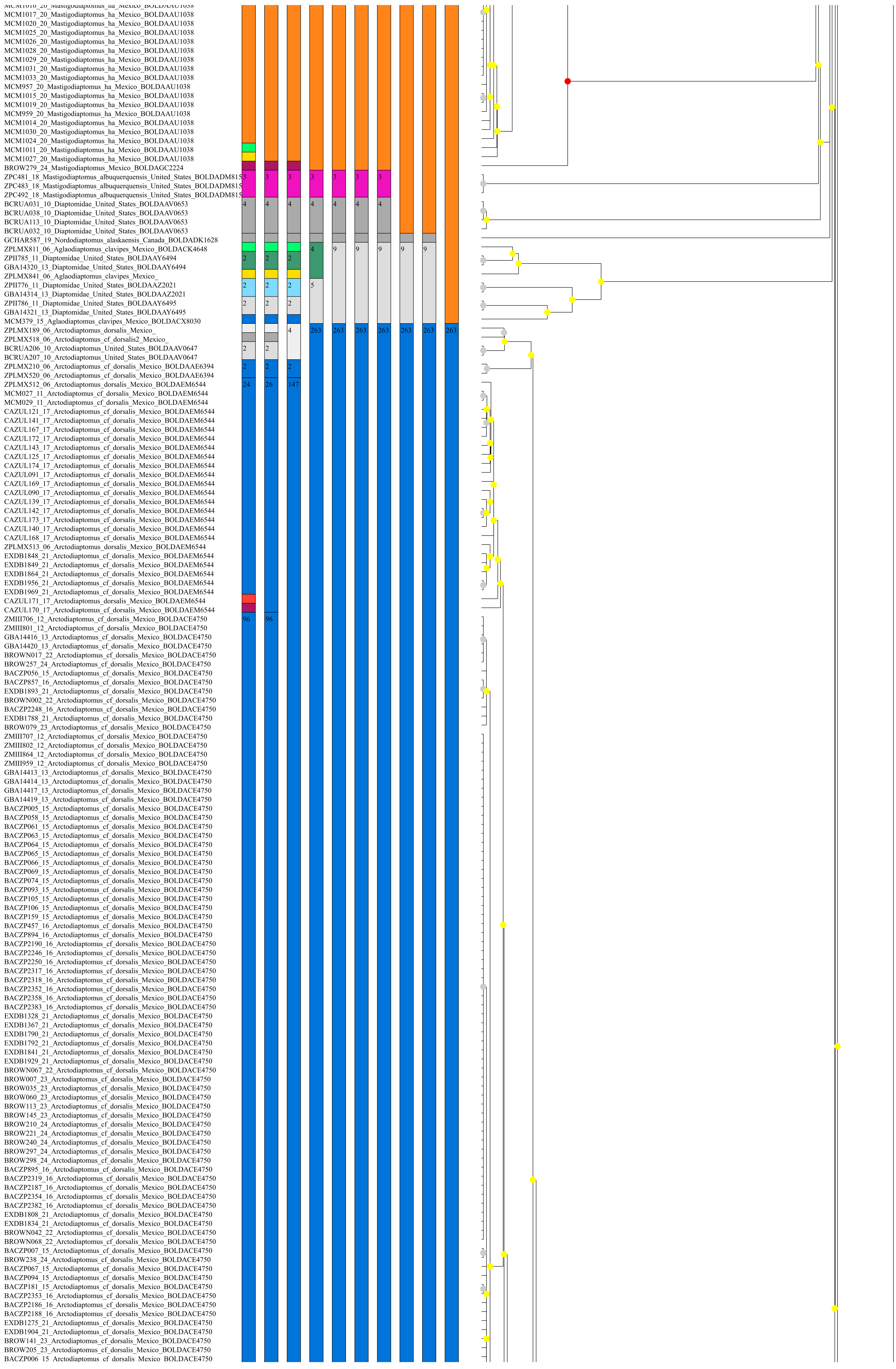

MCM1016_20_Mastigodiaptomus_ha_Mexico_BOLDAAU1038
MCM1017_20_Mastigodiaptomus_ha_Mexico_BOLDAAU1038
MCM1020_20_Mastigodiaptomus_ha_Mexico_BOLDAAU1038
MCM1025_20_Mastigodiaptomus_ha_Mexico_BOLDAAU1038
MCM1026_20_Mastigodiaptomus_ha_Mexico_BOLDAAU1038
MCM1028_20_Mastigodiaptomus_ha_Mexico_BOLDAAU1038
MCM1029_20_Mastigodiaptomus_ha_Mexico_BOLDAAU1038
MCM1031_20_Mastigodiaptomus_ha_Mexico_BOLDAAU1038
MCM1033_20_Mastigodiaptomus_ha_Mexico_BOLDAAU1038
MCM957_20_Mastigodiaptomus_ha_Mexico_BOLDAAU1038
MCM1015_20_Mastigodiaptomus_ha_Mexico_BOLDAAU1038
MCM1019_20_Mastigodiaptomus_ha_Mexico_BOLDAAU1038
MCM959_20_Mastigodiaptomus_ha_Mexico_BOLDAAU1038
MCM1014_20_Mastigodiaptomus_ha_Mexico_BOLDAAU1038
MCM1030_20_Mastigodiaptomus_ha_Mexico_BOLDAAU1038
MCM1024_20_Mastigodiaptomus_ha_Mexico_BOLDAAU1038
MCM1011_20_Mastigodiaptomus_ha_Mexico_BOLDAAU1038
MCM1027_20_Mastigodiaptomus_ha_Mexico_BOLDAAU1038
BROW279_24_Mastigodiaptomus_Mexico_BOLDAGC2224
ZPC481_18_Mastigodiaptomus_albuquerquensis_United_States_BOLDADM815
ZPC483_18_Mastigodiaptomus_albuquerquensis_United_States_BOLDADM815
ZPC492_18_Mastigodiaptomus_albuquerquensis_United_States_BOLDADM815
BCRUA031_10_Diaptomidae_United_States_BOLDAAV0653
BCRUA038_10_Diaptomidae_United_States_BOLDAAV0653
BCRUA113_10_Diaptomidae_United_States_BOLDAAV0653
BCRUA032_10_Diaptomidae_United_States_BOLDAAV0653
GCHAR587_19_Nordodiaptomus_alaskaensis_Canada_BOLDADK1628
ZPLMX811_06_Aglaodiaptomus_clavipes_Mexico_BOLDACK4648
ZPII785_11_Diaptomidae_United_States_BOLDAAY6494
GBA14320_13_Diaptomidae_United_States_BOLDAAY6494
ZPLMX841_06_Aglaodiaptomus_clavipes_Mexico_
ZPII776_11_Diaptomidae_United_States_BOLDAAZ2021
GBA14314_13_Diaptomidae_United_States_BOLDAAZ2021
ZPII786_11_Diaptomidae_United_States_BOLDAAY6495
GBA14321_13_Diaptomidae_United_States_BOLDAAY6495
MCM379_15_Aglaodiaptomus_clavipes_Mexico_BOLDACX8030
ZPLMX189_06_Arctodiaptomus_dorsalis_Mexico_
ZPLMX518_06_Arctodiaptomus_cf_dorsalis2_Mexico_
BCRUA206_10_Arctodiaptomus_United_States_BOLDAAV0647
BCRUA207_10_Arctodiaptomus_United_States_BOLDAAV0647
ZPLMX210_06_Arctodiaptomus_cf_dorsalis_Mexico_BOLDAAE6394
ZPLMX520_06_Arctodiaptomus_cf_dorsalis_Mexico_BOLDAAE6394
ZPLMX512_06_Arctodiaptomus_dorsalis_Mexico_BOLDAEM6544
MCM027_11_Arctodiaptomus_cf_dorsalis_Mexico_BOLDAEM6544
MCM029_11_Arctodiaptomus_cf_dorsalis_Mexico_BOLDAEM6544
CAZUL121_17_Arctodiaptomus_cf_dorsalis_Mexico_BOLDAEM6544
CAZUL141_17_Arctodiaptomus_cf_dorsalis_Mexico_BOLDAEM6544
CAZUL167_17_Arctodiaptomus_cf_dorsalis_Mexico_BOLDAEM6544
CAZUL172_17_Arctodiaptomus_cf_dorsalis_Mexico_BOLDAEM6544
CAZUL143_17_Arctodiaptomus_cf_dorsalis_Mexico_BOLDAEM6544
CAZUL125_17_Arctodiaptomus_cf_dorsalis_Mexico_BOLDAEM6544
CAZUL174_17_Arctodiaptomus_cf_dorsalis_Mexico_BOLDAEM6544
CAZUL091_17_Arctodiaptomus_cf_dorsalis_Mexico_BOLDAEM6544
CAZUL169_17_Arctodiaptomus_cf_dorsalis_Mexico_BOLDAEM6544
CAZUL090_17_Arctodiaptomus_cf_dorsalis_Mexico_BOLDAEM6544
CAZUL139_17_Arctodiaptomus_cf_dorsalis_Mexico_BOLDAEM6544
CAZUL142_17_Arctodiaptomus_cf_dorsalis_Mexico_BOLDAEM6544
CAZUL173_17_Arctodiaptomus_cf_dorsalis_Mexico_BOLDAEM6544
CAZUL140_17_Arctodiaptomus_cf_dorsalis_Mexico_BOLDAEM6544
CAZUL168_17_Arctodiaptomus_cf_dorsalis_Mexico_BOLDAEM6544
ZPLMX513_06_Arctodiaptomus_dorsalis_Mexico_BOLDAEM6544
EXDB1848_21_Arctodiaptomus_cf_dorsalis_Mexico_BOLDAEM6544
EXDB1849_21_Arctodiaptomus_cf_dorsalis_Mexico_BOLDAEM6544
EXDB1864_21_Arctodiaptomus_cf_dorsalis_Mexico_BOLDAEM6544
EXDB1956_21_Arctodiaptomus_cf_dorsalis_Mexico_BOLDAEM6544
EXDB1969_21_Arctodiaptomus_cf_dorsalis_Mexico_BOLDAEM6544
CAZUL171_17_Arctodiaptomus_dorsalis_Mexico_BOLDAEM6544
CAZUL170_17_Arctodiaptomus_cf_dorsalis_Mexico_BOLDAEM6544
ZMIII706_12_Arctodiaptomus_cf_dorsalis_Mexico_BOLDACE4750
ZMIII801_12_Arctodiaptomus_cf_dorsalis_Mexico_BOLDACE4750
GBA14416_13_Arctodiaptomus_cf_dorsalis_Mexico_BOLDACE4750
GBA14420_13_Arctodiaptomus_cf_dorsalis_Mexico_BOLDACE4750
BROWN017_22_Arctodiaptomus_cf_dorsalis_Mexico_BOLDACE4750
BROW257_24_Arctodiaptomus_cf_dorsalis_Mexico_BOLDACE4750
BACZP056_15_Arctodiaptomus_cf_dorsalis_Mexico_BOLDACE4750
BACZP857_16_Arctodiaptomus_cf_dorsalis_Mexico_BOLDACE4750
EXDB1893_21_Arctodiaptomus_cf_dorsalis_Mexico_BOLDACE4750
BROWN002_22_Arctodiaptomus_cf_dorsalis_Mexico_BOLDACE4750
BACZP2248_16_Arctodiaptomus_cf_dorsalis_Mexico_BOLDACE4750
EXDB1788_21_Arctodiaptomus_cf_dorsalis_Mexico_BOLDACE4750
BROW079_23_Arctodiaptomus_cf_dorsalis_Mexico_BOLDACE4750
ZMIII707_12_Arctodiaptomus_cf_dorsalis_Mexico_BOLDACE4750
ZMIII802_12_Arctodiaptomus_cf_dorsalis_Mexico_BOLDACE4750
ZMIII864_12_Arctodiaptomus_cf_dorsalis_Mexico_BOLDACE4750
ZMIII959_12_Arctodiaptomus_cf_dorsalis_Mexico_BOLDACE4750
GBA14413_13_Arctodiaptomus_cf_dorsalis_Mexico_BOLDACE4750
GBA14414_13_Arctodiaptomus_cf_dorsalis_Mexico_BOLDACE4750
GBA14417_13_Arctodiaptomus_cf_dorsalis_Mexico_BOLDACE4750
GBA14419_13_Arctodiaptomus_cf_dorsalis_Mexico_BOLDACE4750
BACZP005_15_Arctodiaptomus_cf_dorsalis_Mexico_BOLDACE4750
BACZP058_15_Arctodiaptomus_cf_dorsalis_Mexico_BOLDACE4750
BACZP061_15_Arctodiaptomus_cf_dorsalis_Mexico_BOLDACE4750
BACZP063_15_Arctodiaptomus_cf_dorsalis_Mexico_BOLDACE4750
BACZP064_15_Arctodiaptomus_cf_dorsalis_Mexico_BOLDACE4750
BACZP065_15_Arctodiaptomus_cf_dorsalis_Mexico_BOLDACE4750
BACZP066_15_Arctodiaptomus_cf_dorsalis_Mexico_BOLDACE4750
BACZP069_15_Arctodiaptomus_cf_dorsalis_Mexico_BOLDACE4750
BACZP074_15_Arctodiaptomus_cf_dorsalis_Mexico_BOLDACE4750
BACZP093_15_Arctodiaptomus_cf_dorsalis_Mexico_BOLDACE4750
BACZP105_15_Arctodiaptomus_cf_dorsalis_Mexico_BOLDACE4750
BACZP106_15_Arctodiaptomus_cf_dorsalis_Mexico_BOLDACE4750
BACZP159_15_Arctodiaptomus_cf_dorsalis_Mexico_BOLDACE4750
BACZP457_16_Arctodiaptomus_cf_dorsalis_Mexico_BOLDACE4750
BACZP894_16_Arctodiaptomus_cf_dorsalis_Mexico_BOLDACE4750
BACZP2190_16_Arctodiaptomus_cf_dorsalis_Mexico_BOLDACE4750
BACZP2246_16_Arctodiaptomus_cf_dorsalis_Mexico_BOLDACE4750
BACZP2250_16_Arctodiaptomus_cf_dorsalis_Mexico_BOLDACE4750
BACZP2317_16_Arctodiaptomus_cf_dorsalis_Mexico_BOLDACE4750
BACZP2318_16_Arctodiaptomus_cf_dorsalis_Mexico_BOLDACE4750
BACZP2352_16_Arctodiaptomus_cf_dorsalis_Mexico_BOLDACE4750
BACZP2358_16_Arctodiaptomus_cf_dorsalis_Mexico_BOLDACE4750
BACZP2383_16_Arctodiaptomus_cf_dorsalis_Mexico_BOLDACE4750
EXDB1328_21_Arctodiaptomus_cf_dorsalis_Mexico_BOLDACE4750
EXDB1367_21_Arctodiaptomus_cf_dorsalis_Mexico_BOLDACE4750
EXDB1790_21_Arctodiaptomus_cf_dorsalis_Mexico_BOLDACE4750
EXDB1792_21_Arctodiaptomus_cf_dorsalis_Mexico_BOLDACE4750
EXDB1841_21_Arctodiaptomus_cf_dorsalis_Mexico_BOLDACE4750
EXDB1929_21_Arctodiaptomus_cf_dorsalis_Mexico_BOLDACE4750
BROWN067_22_Arctodiaptomus_cf_dorsalis_Mexico_BOLDACE4750
BROW007_23_Arctodiaptomus_cf_dorsalis_Mexico_BOLDACE4750
BROW035_23_Arctodiaptomus_cf_dorsalis_Mexico_BOLDACE4750
BROW060_23_Arctodiaptomus_cf_dorsalis_Mexico_BOLDACE4750
BROW113_23_Arctodiaptomus_cf_dorsalis_Mexico_BOLDACE4750
BROW145_23_Arctodiaptomus_cf_dorsalis_Mexico_BOLDACE4750
BROW210_24_Arctodiaptomus_cf_dorsalis_Mexico_BOLDACE4750
BROW221_24_Arctodiaptomus_cf_dorsalis_Mexico_BOLDACE4750
BROW240_24_Arctodiaptomus_cf_dorsalis_Mexico_BOLDACE4750
BROW297_24_Arctodiaptomus_cf_dorsalis_Mexico_BOLDACE4750
BROW298_24_Arctodiaptomus_cf_dorsalis_Mexico_BOLDACE4750
BACZP895_16_Arctodiaptomus_cf_dorsalis_Mexico_BOLDACE4750
BACZP2319_16_Arctodiaptomus_cf_dorsalis_Mexico_BOLDACE4750
BACZP2187_16_Arctodiaptomus_cf_dorsalis_Mexico_BOLDACE4750
BACZP2354_16_Arctodiaptomus_cf_dorsalis_Mexico_BOLDACE4750
BACZP2382_16_Arctodiaptomus_cf_dorsalis_Mexico_BOLDACE4750
EXDB1808_21_Arctodiaptomus_cf_dorsalis_Mexico_BOLDACE4750
EXDB1834_21_Arctodiaptomus_cf_dorsalis_Mexico_BOLDACE4750
BROWN042_22_Arctodiaptomus_cf_dorsalis_Mexico_BOLDACE4750
BROWN068_22_Arctodiaptomus_cf_dorsalis_Mexico_BOLDACE4750
BACZP007_15_Arctodiaptomus_cf_dorsalis_Mexico_BOLDACE4750
BROW238_24_Arctodiaptomus_cf_dorsalis_Mexico_BOLDACE4750
BACZP067_15_Arctodiaptomus_cf_dorsalis_Mexico_BOLDACE4750
BACZP094_15_Arctodiaptomus_cf_dorsalis_Mexico_BOLDACE4750
BACZP181_15_Arctodiaptomus_cf_dorsalis_Mexico_BOLDACE4750
BACZP2353_16_Arctodiaptomus_cf_dorsalis_Mexico_BOLDACE4750
BACZP2186_16_Arctodiaptomus_cf_dorsalis_Mexico_BOLDACE4750
BACZP2188_16_Arctodiaptomus_cf_dorsalis_Mexico_BOLDACE4750
EXDB1275_21_Arctodiaptomus_cf_dorsalis_Mexico_BOLDACE4750
EXDB1904_21_Arctodiaptomus_cf_dorsalis_Mexico_BOLDACE4750
BROW141_23_Arctodiaptomus_cf_dorsalis_Mexico_BOLDACE4750
BROW205_23_Arctodiaptomus_cf_dorsalis_Mexico_BOLDACE4750
BACZP006_15_Arctodiaptomus_cf_dorsalis_Mexico_BOLDACE4750
3 3 3 3 3 3 3
4 4 4 4 4 4 4
4 9 9 9 9 9
2 2 2
2 2 2 5
2 2 2
263 263 263 263 263 263 263
4
2 2
2 2 2
24 26 147
96 96

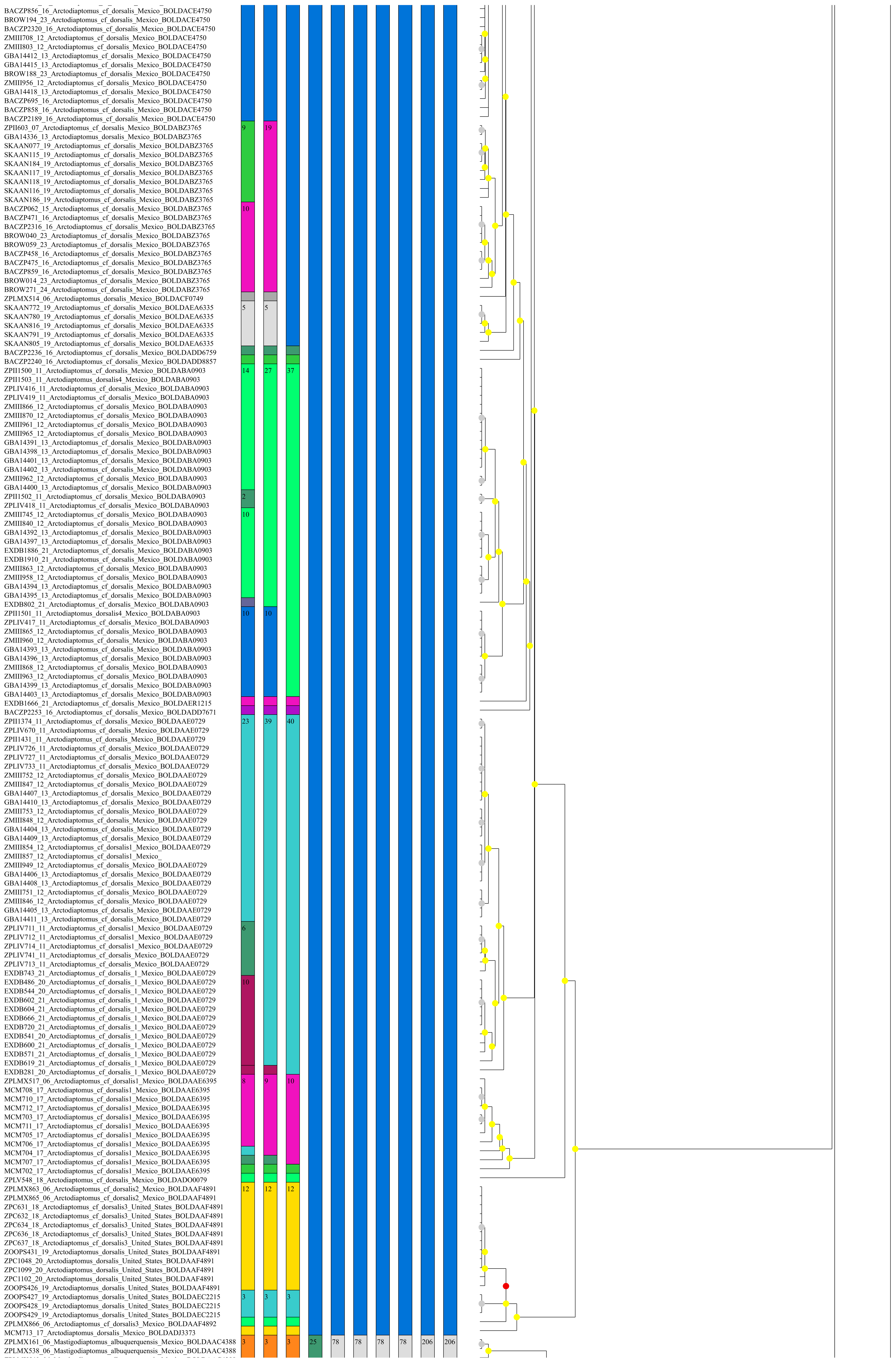
BACZP856_16_Arctodiaptomus_cf_dorsalis_Mexico_BOLDACE4750
BROW194_23_Arctodiaptomus_cf_dorsalis_Mexico_BOLDACE4750
BACZP2320_16_Arctodiaptomus_cf_dorsalis_Mexico_BOLDACE4750
ZMIII708_12_Arctodiaptomus_cf_dorsalis_Mexico_BOLDACE4750
ZMIII803_12_Arctodiaptomus_cf_dorsalis_Mexico_BOLDACE4750
GBA14412_13_Arctodiaptomus_cf_dorsalis_Mexico_BOLDACE4750
GBA14415_13_Arctodiaptomus_cf_dorsalis_Mexico_BOLDACE4750
BROW188_23_Arctodiaptomus_cf_dorsalis_Mexico_BOLDACE4750
ZMIII956_12_Arctodiaptomus_cf_dorsalis_Mexico_BOLDACE4750
GBA14418_13_Arctodiaptomus_cf_dorsalis_Mexico_BOLDACE4750
BACZP695_16_Arctodiaptomus_cf_dorsalis_Mexico_BOLDACE4750
BACZP858_16_Arctodiaptomus_cf_dorsalis_Mexico_BOLDACE4750
BACZP2189_16_Arctodiaptomus_cf_dorsalis_Mexico_BOLDACE4750
ZPII603_07_Arctodiaptomus_cf_dorsalis_Mexico_BOLDABZ3765
GBA14336_13_Arctodiaptomus_dorsalis_Mexico_BOLDABZ3765
SKAAN077_19_Arctodiaptomus_cf_dorsalis_Mexico_BOLDABZ3765
SKAAN115_19_Arctodiaptomus_cf_dorsalis_Mexico_BOLDABZ3765
SKAAN184_19_Arctodiaptomus_cf_dorsalis_Mexico_BOLDABZ3765
SKAAN117_19_Arctodiaptomus_cf_dorsalis_Mexico_BOLDABZ3765
SKAAN118_19_Arctodiaptomus_cf_dorsalis_Mexico_BOLDABZ3765
SKAAN116_19_Arctodiaptomus_cf_dorsalis_Mexico_BOLDABZ3765
SKAAN186_19_Arctodiaptomus_cf_dorsalis_Mexico_BOLDABZ3765
BACZP062_15_Arctodiaptomus_cf_dorsalis_Mexico_BOLDABZ3765
BACZP471_16_Arctodiaptomus_cf_dorsalis_Mexico_BOLDABZ3765
BACZP2316_16_Arctodiaptomus_cf_dorsalis_Mexico_BOLDABZ3765
BROW040_23_Arctodiaptomus_cf_dorsalis_Mexico_BOLDABZ3765
BROW059_23_Arctodiaptomus_cf_dorsalis_Mexico_BOLDABZ3765
BACZP458_16_Arctodiaptomus_cf_dorsalis_Mexico_BOLDABZ3765
BACZP475_16_Arctodiaptomus_cf_dorsalis_Mexico_BOLDABZ3765
BACZP859_16_Arctodiaptomus_cf_dorsalis_Mexico_BOLDABZ3765
BROW014_23_Arctodiaptomus_cf_dorsalis_Mexico_BOLDABZ3765
BROW271_24_Arctodiaptomus_cf_dorsalis_Mexico_BOLDABZ3765
ZPLMX514_06_Arctodiaptomus_dorsalis_Mexico_BOLDACF0749
SKAAN772_19_Arctodiaptomus_cf_dorsalis_Mexico_BOLDAEA6335
SKAAN780_19_Arctodiaptomus_cf_dorsalis_Mexico_BOLDAEA6335
SKAAN816_19_Arctodiaptomus_cf_dorsalis_Mexico_BOLDAEA6335
SKAAN791_19_Arctodiaptomus_cf_dorsalis_Mexico_BOLDAEA6335
SKAAN805_19_Arctodiaptomus_cf_dorsalis_Mexico_BOLDAEA6335
BACZP2236_16_Arctodiaptomus_cf_dorsalis_Mexico_BOLDADD6759
BACZP2240_16_Arctodiaptomus_cf_dorsalis_Mexico_BOLDADD8857
ZPII1500_11_Arctodiaptomus_cf_dorsalis_Mexico_BOLDABA0903
ZPII1503_11_Arctodiaptomus_dorsalis4_Mexico_BOLDABA0903
ZPLIV416_11_Arctodiaptomus_cf_dorsalis_Mexico_BOLDABA0903
ZPLIV419_11_Arctodiaptomus_cf_dorsalis_Mexico_BOLDABA0903
ZMIII866_12_Arctodiaptomus_cf_dorsalis_Mexico_BOLDABA0903
ZMIII870_12_Arctodiaptomus_cf_dorsalis_Mexico_BOLDABA0903
ZMIII961_12_Arctodiaptomus_cf_dorsalis_Mexico_BOLDABA0903
ZMIII965_12_Arctodiaptomus_cf_dorsalis_Mexico_BOLDABA0903
GBA14391_13_Arctodiaptomus_cf_dorsalis_Mexico_BOLDABA0903
GBA14398_13_Arctodiaptomus_cf_dorsalis_Mexico_BOLDABA0903
GBA14401_13_Arctodiaptomus_cf_dorsalis_Mexico_BOLDABA0903
GBA14402_13_Arctodiaptomus_cf_dorsalis_Mexico_BOLDABA0903
ZMIII962_12_Arctodiaptomus_cf_dorsalis_Mexico_BOLDABA0903
GBA14400_13_Arctodiaptomus_cf_dorsalis_Mexico_BOLDABA0903
ZPII1502_11_Arctodiaptomus_cf_dorsalis_Mexico_BOLDABA0903
ZPLIV418_11_Arctodiaptomus_cf_dorsalis_Mexico_BOLDABA0903
ZMIII745_12_Arctodiaptomus_cf_dorsalis_Mexico_BOLDABA0903
ZMIII840_12_Arctodiaptomus_cf_dorsalis_Mexico_BOLDABA0903
GBA14392_13_Arctodiaptomus_cf_dorsalis_Mexico_BOLDABA0903
GBA14397_13_Arctodiaptomus_cf_dorsalis_Mexico_BOLDABA0903
EXDB1886_21_Arctodiaptomus_cf_dorsalis_Mexico_BOLDABA0903
EXDB1910_21_Arctodiaptomus_cf_dorsalis_Mexico_BOLDABA0903
ZMIII863_12_Arctodiaptomus_cf_dorsalis_Mexico_BOLDABA0903
ZMIII958_12_Arctodiaptomus_cf_dorsalis_Mexico_BOLDABA0903
GBA14394_13_Arctodiaptomus_cf_dorsalis_Mexico_BOLDABA0903
GBA14395_13_Arctodiaptomus_cf_dorsalis_Mexico_BOLDABA0903
EXDB802_21_Arctodiaptomus_cf_dorsalis_Mexico_BOLDABA0903
ZPII1501_11_Arctodiaptomus_dorsalis4_Mexico_BOLDABA0903
ZPLIV417_11_Arctodiaptomus_cf_dorsalis_Mexico_BOLDABA0903
ZMIII865_12_Arctodiaptomus_cf_dorsalis_Mexico_BOLDABA0903
ZMIII960_12_Arctodiaptomus_cf_dorsalis_Mexico_BOLDABA0903
GBA14393_13_Arctodiaptomus_cf_dorsalis_Mexico_BOLDABA0903
GBA14396_13_Arctodiaptomus_cf_dorsalis_Mexico_BOLDABA0903
ZMIII868_12_Arctodiaptomus_cf_dorsalis_Mexico_BOLDABA0903
ZMIII963_12_Arctodiaptomus_cf_dorsalis_Mexico_BOLDABA0903
GBA14399_13_Arctodiaptomus_cf_dorsalis_Mexico_BOLDABA0903
GBA14403_13_Arctodiaptomus_cf_dorsalis_Mexico_BOLDABA0903
EXDB1666_21_Arctodiaptomus_cf_dorsalis_Mexico_BOLDAER1215
BACZP2253_16_Arctodiaptomus_cf_dorsalis_Mexico_BOLDADD7671
ZPII1374_11_Arctodiaptomus_cf_dorsalis_Mexico_BOLDAAE0729
ZPLIV670_11_Arctodiaptomus_cf_dorsalis_Mexico_BOLDAAE0729
ZPII1431_11_Arctodiaptomus_cf_dorsalis_Mexico_BOLDAAE0729
ZPLIV726_11_Arctodiaptomus_cf_dorsalis_Mexico_BOLDAAE0729
ZPLIV727_11_Arctodiaptomus_cf_dorsalis_Mexico_BOLDAAE0729
ZPLIV733_11_Arctodiaptomus_cf_dorsalis_Mexico_BOLDAAE0729
ZMIII752_12_Arctodiaptomus_cf_dorsalis_Mexico_BOLDAAE0729
ZMIII847_12_Arctodiaptomus_cf_dorsalis_Mexico_BOLDAAE0729
GBA14407_13_Arctodiaptomus_cf_dorsalis_Mexico_BOLDAAE0729
GBA14410_13_Arctodiaptomus_cf_dorsalis_Mexico_BOLDAAE0729
ZMIII753_12_Arctodiaptomus_cf_dorsalis_Mexico_BOLDAAE0729
ZMIII848_12_Arctodiaptomus_cf_dorsalis_Mexico_BOLDAAE0729
GBA14404_13_Arctodiaptomus_cf_dorsalis_Mexico_BOLDAAE0729
GBA14409_13_Arctodiaptomus_cf_dorsalis_Mexico_BOLDAAE0729
ZMIII854_12_Arctodiaptomus_cf_dorsalis1_Mexico_BOLDAAE0729
ZMIII857_12_Arctodiaptomus_cf_dorsalis1_Mexico_
ZMIII949_12_Arctodiaptomus_cf_dorsalis_Mexico_BOLDAAE0729
GBA14406_13_Arctodiaptomus_cf_dorsalis_Mexico_BOLDAAE0729
GBA14408_13_Arctodiaptomus_cf_dorsalis_Mexico_BOLDAAE0729
ZMIII751_12_Arctodiaptomus_cf_dorsalis_Mexico_BOLDAAE0729
ZMIII846_12_Arctodiaptomus_cf_dorsalis_Mexico_BOLDAAE0729
GBA14405_13_Arctodiaptomus_cf_dorsalis_Mexico_BOLDAAE0729
GBA14411_13_Arctodiaptomus_cf_dorsalis_Mexico_BOLDAAE0729
ZPLIV711_11_Arctodiaptomus_cf_dorsalis1_Mexico_BOLDAAE0729
ZPLIV712_11_Arctodiaptomus_cf_dorsalis1_Mexico_BOLDAAE0729
ZPLIV714_11_Arctodiaptomus_cf_dorsalis1_Mexico_BOLDAAE0729
ZPLIV741_11_Arctodiaptomus_cf_dorsalis_Mexico_BOLDAAE0729
ZPLIV713_11_Arctodiaptomus_cf_dorsalis_Mexico_BOLDAAE0729
EXDB743_21_Arctodiaptomus_cf_dorsalis_1_Mexico_BOLDAAE0729
EXDB486_20_Arctodiaptomus_cf_dorsalis_1_Mexico_BOLDAAE0729
EXDB544_20_Arctodiaptomus_cf_dorsalis_1_Mexico_BOLDAAE0729
EXDB602_21_Arctodiaptomus_cf_dorsalis_1_Mexico_BOLDAAE0729
EXDB604_21_Arctodiaptomus_cf_dorsalis_1_Mexico_BOLDAAE0729
EXDB666_21_Arctodiaptomus_cf_dorsalis_1_Mexico_BOLDAAE0729
EXDB720_21_Arctodiaptomus_cf_dorsalis_1_Mexico_BOLDAAE0729
EXDB541_20_Arctodiaptomus_cf_dorsalis_1_Mexico_BOLDAAE0729
EXDB600_21_Arctodiaptomus_cf_dorsalis_1_Mexico_BOLDAAE0729
EXDB571_21_Arctodiaptomus_cf_dorsalis_1_Mexico_BOLDAAE0729
EXDB619_21_Arctodiaptomus_cf_dorsalis_1_Mexico_BOLDAAE0729
EXDB281_20_Arctodiaptomus_cf_dorsalis_1_Mexico_BOLDAAE0729
ZPLMX517_06_Arctodiaptomus_cf_dorsalis1_Mexico_BOLDAAE6395
MCM708_17_Arctodiaptomus_cf_dorsalis1_Mexico_BOLDAAE6395
MCM710_17_Arctodiaptomus_cf_dorsalis1_Mexico_BOLDAAE6395
MCM712_17_Arctodiaptomus_cf_dorsalis1_Mexico_BOLDAAE6395
MCM703_17_Arctodiaptomus_cf_dorsalis1_Mexico_BOLDAAE6395
MCM711_17_Arctodiaptomus_cf_dorsalis1_Mexico_BOLDAAE6395
MCM705_17_Arctodiaptomus_cf_dorsalis1_Mexico_BOLDAAE6395
MCM706_17_Arctodiaptomus_cf_dorsalis1_Mexico_BOLDAAE6395
MCM704_17_Arctodiaptomus_cf_dorsalis1_Mexico_BOLDAAE6395
MCM707_17_Arctodiaptomus_cf_dorsalis1_Mexico_BOLDAAE6395
MCM702_17_Arctodiaptomus_cf_dorsalis1_Mexico_BOLDAAE6395
ZPLV548_18_Arctodiaptomus_cf_dorsalis_Mexico_BOLDADO0079
ZPLMX863_06_Arctodiaptomus_cf_dorsalis2_Mexico_BOLDAAF4891
ZPLMX865_06_Arctodiaptomus_cf_dorsalis2_Mexico_BOLDAAF4891
ZPC631_18_Arctodiaptomus_cf_dorsalis3_United_States_BOLDAAF4891
ZPC632_18_Arctodiaptomus_cf_dorsalis3_United_States_BOLDAAF4891
ZPC634_18_Arctodiaptomus_cf_dorsalis3_United_States_BOLDAAF4891
ZPC636_18_Arctodiaptomus_cf_dorsalis3_United_States_BOLDAAF4891
ZPC637_18_Arctodiaptomus_cf_dorsalis3_United_States_BOLDAAF4891
ZOOPS431_19_Arctodiaptomus_dorsalis_United_States_BOLDAAF4891
ZPC1048_20_Arctodiaptomus_dorsalis_United_States_BOLDAAF4891
ZPC1099_20_Arctodiaptomus_dorsalis_United_States_BOLDAAF4891
ZPC1102_20_Arctodiaptomus_dorsalis_United_States_BOLDAAF4891
ZOOPS426_19_Arctodiaptomus_dorsalis_United_States_BOLDAAF4891
ZOOPS427_19_Arctodiaptomus_dorsalis_United_States_BOLDAEC2215
ZOOPS428_19_Arctodiaptomus_dorsalis_United_States_BOLDAEC2215
ZOOPS429_19_Arctodiaptomus_dorsalis_United_States_BOLDAEC2215
ZPLMX866_06_Arctodiaptomus_cf_dorsalis3_Mexico_BOLDAAF4892
MCM713_17_Arctodiaptomus_dorsalis_Mexico_BOLDADJ3373
ZPLMX161_06_Mastigodiaptomus_albuquerquensis_Mexico_BOLDAAC4388
ZPLMX538_06_Mastigodiaptomus_albuquerquensis_Mexico_BOLDAAC4388
9
19
10
5
5
14
27
37
2
10
10
10
23
39
40
6
10
8
9
10
12
12
12
3
3
3
3
3
3
25
78
78
78
78
206
206

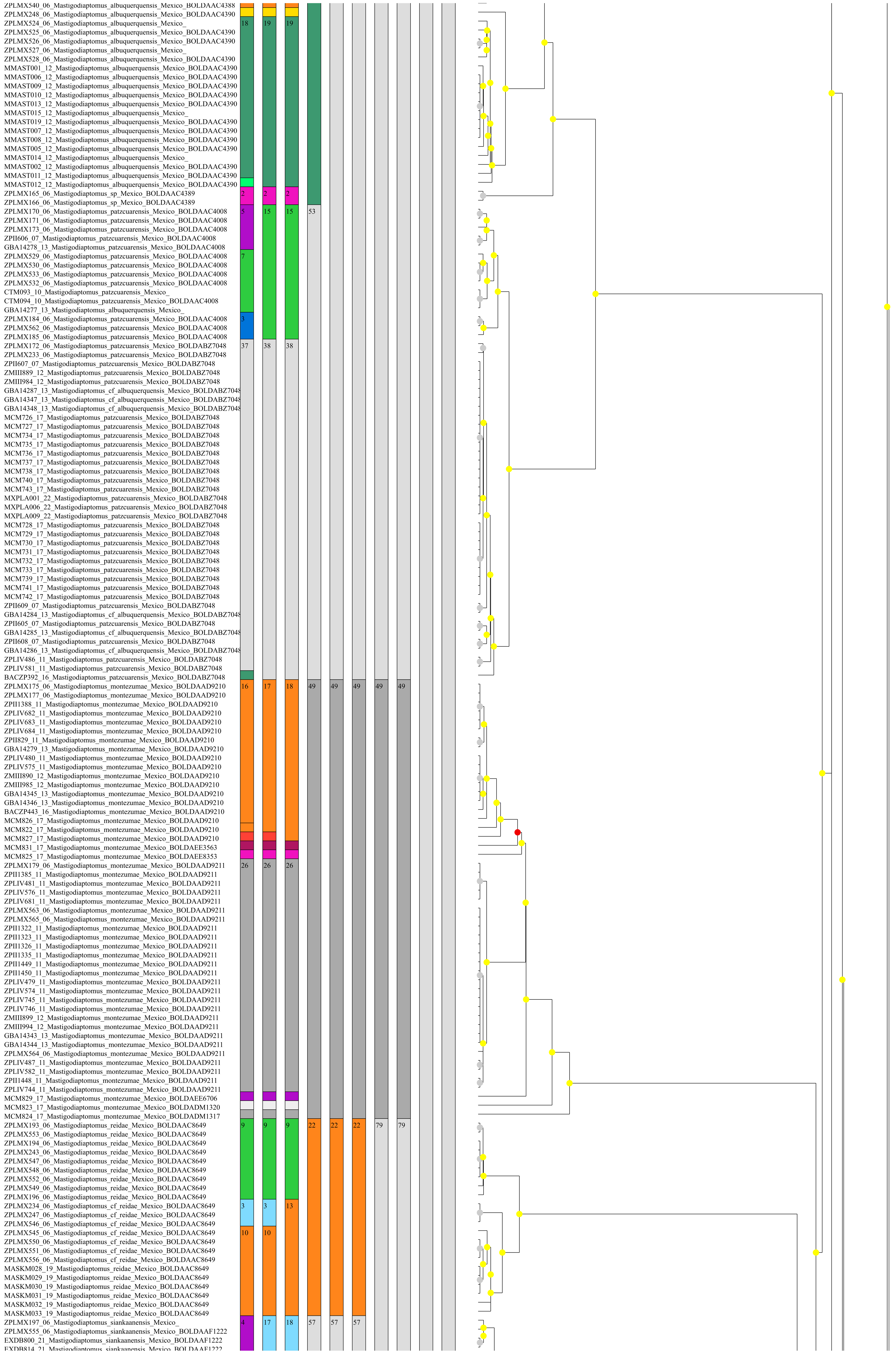
ZPLMX540_06_Mastigodiaptomus_albuquerquensis_Mexico_BOLDAAC4388
ZPLMX248_06_Mastigodiaptomus_albuquerquensis_Mexico_BOLDAAC4390
ZPLMX524_06_Mastigodiaptomus_albuquerquensis_Mexico_
ZPLMX525_06_Mastigodiaptomus_albuquerquensis_Mexico_BOLDAAC4390
ZPLMX526_06_Mastigodiaptomus_albuquerquensis_Mexico_BOLDAAC4390
ZPLMX527_06_Mastigodiaptomus_albuquerquensis_Mexico_
ZPLMX528_06_Mastigodiaptomus_albuquerquensis_Mexico_BOLDAAC4390
MMAST001_12_Mastigodiaptomus_albuquerquensis_Mexico_BOLDAAC4390
MMAST006_12_Mastigodiaptomus_albuquerquensis_Mexico_BOLDAAC4390
MMAST009_12_Mastigodiaptomus_albuquerquensis_Mexico_BOLDAAC4390
MMAST010_12_Mastigodiaptomus_albuquerquensis_Mexico_BOLDAAC4390
MMAST013_12_Mastigodiaptomus_albuquerquensis_Mexico_BOLDAAC4390
MMAST015_12_Mastigodiaptomus_albuquerquensis_Mexico_
MMAST019_12_Mastigodiaptomus_albuquerquensis_Mexico_BOLDAAC4390
MMAST007_12_Mastigodiaptomus_albuquerquensis_Mexico_BOLDAAC4390
MMAST008_12_Mastigodiaptomus_albuquerquensis_Mexico_BOLDAAC4390
MMAST005_12_Mastigodiaptomus_albuquerquensis_Mexico_BOLDAAC4390
MMAST014_12_Mastigodiaptomus_albuquerquensis_Mexico_
MMAST002_12_Mastigodiaptomus_albuquerquensis_Mexico_BOLDAAC4390
MMAST011_12_Mastigodiaptomus_albuquerquensis_Mexico_BOLDAAC4390
MMAST012_12_Mastigodiaptomus_albuquerquensis_Mexico_BOLDAAC4390
ZPLMX165_06_Mastigodiaptomus_sp_Mexico_BOLDAAC4389
ZPLMX166_06_Mastigodiaptomus_sp_Mexico_BOLDAAC4389
ZPLMX170_06_Mastigodiaptomus_patzcuarensis_Mexico_BOLDAAC4008
ZPLMX171_06_Mastigodiaptomus_patzcuarensis_Mexico_BOLDAAC4008
ZPLMX173_06_Mastigodiaptomus_patzcuarensis_Mexico_BOLDAAC4008
ZPII606_07_Mastigodiaptomus_patzcuarensis_Mexico_BOLDAAC4008
GBA14278_13_Mastigodiaptomus_patzcuarensis_Mexico_BOLDAAC4008
ZPLMX529_06_Mastigodiaptomus_patzcuarensis_Mexico_BOLDAAC4008
ZPLMX530_06_Mastigodiaptomus_patzcuarensis_Mexico_BOLDAAC4008
ZPLMX533_06_Mastigodiaptomus_patzcuarensis_Mexico_BOLDAAC4008
ZPLMX532_06_Mastigodiaptomus_patzcuarensis_Mexico_BOLDAAC4008
CTM093_10_Mastigodiaptomus_patzcuarensis_Mexico_
CTM094_10_Mastigodiaptomus_patzcuarensis_Mexico_BOLDAAC4008
GBA14277_13_Mastigodiaptomus_albuquerquensis_Mexico_
ZPLMX184_06_Mastigodiaptomus_patzcuarensis_Mexico_BOLDAAC4008
ZPLMX562_06_Mastigodiaptomus_patzcuarensis_Mexico_BOLDAAC4008
ZPLMX185_06_Mastigodiaptomus_patzcuarensis_Mexico_BOLDAAC4008
ZPLMX172_06_Mastigodiaptomus_patzcuarensis_Mexico_BOLDABZ7048
ZPLMX233_06_Mastigodiaptomus_patzcuarensis_Mexico_BOLDABZ7048
ZPII607_07_Mastigodiaptomus_patzcuarensis_Mexico_BOLDABZ7048
ZMIII889_12_Mastigodiaptomus_patzcuarensis_Mexico_BOLDABZ7048
ZMIII984_12_Mastigodiaptomus_patzcuarensis_Mexico_BOLDABZ7048
GBA14287_13_Mastigodiaptomus_cf_albuquerquensis_Mexico_BOLDABZ7048
GBA14347_13_Mastigodiaptomus_cf_albuquerquensis_Mexico_BOLDABZ7048
GBA14348_13_Mastigodiaptomus_cf_albuquerquensis_Mexico_BOLDABZ7048
MCM726_17_Mastigodiaptomus_patzcuarensis_Mexico_BOLDABZ7048
MCM727_17_Mastigodiaptomus_patzcuarensis_Mexico_BOLDABZ7048
MCM734_17_Mastigodiaptomus_patzcuarensis_Mexico_BOLDABZ7048
MCM735_17_Mastigodiaptomus_patzcuarensis_Mexico_BOLDABZ7048
MCM736_17_Mastigodiaptomus_patzcuarensis_Mexico_BOLDABZ7048
MCM737_17_Mastigodiaptomus_patzcuarensis_Mexico_BOLDABZ7048
MCM738_17_Mastigodiaptomus_patzcuarensis_Mexico_BOLDABZ7048
MCM740_17_Mastigodiaptomus_patzcuarensis_Mexico_BOLDABZ7048
MCM743_17_Mastigodiaptomus_patzcuarensis_Mexico_BOLDABZ7048
MXPLA001_22_Mastigodiaptomus_patzcuarensis_Mexico_BOLDABZ7048
MXPLA006_22_Mastigodiaptomus_patzcuarensis_Mexico_BOLDABZ7048
MXPLA009_22_Mastigodiaptomus_patzcuarensis_Mexico_BOLDABZ7048
MCM728_17_Mastigodiaptomus_patzcuarensis_Mexico_BOLDABZ7048
MCM729_17_Mastigodiaptomus_patzcuarensis_Mexico_BOLDABZ7048
MCM730_17_Mastigodiaptomus_patzcuarensis_Mexico_BOLDABZ7048
MCM731_17_Mastigodiaptomus_patzcuarensis_Mexico_BOLDABZ7048
MCM732_17_Mastigodiaptomus_patzcuarensis_Mexico_BOLDABZ7048
MCM733_17_Mastigodiaptomus_patzcuarensis_Mexico_BOLDABZ7048
MCM739_17_Mastigodiaptomus_patzcuarensis_Mexico_BOLDABZ7048
MCM741_17_Mastigodiaptomus_patzcuarensis_Mexico_BOLDABZ7048
MCM742_17_Mastigodiaptomus_patzcuarensis_Mexico_BOLDABZ7048
ZPII609_07_Mastigodiaptomus_patzcuarensis_Mexico_BOLDABZ7048
GBA14284_13_Mastigodiaptomus_cf_albuquerquensis_Mexico_BOLDABZ7048
ZPII605_07_Mastigodiaptomus_patzcuarensis_Mexico_BOLDABZ7048
GBA14285_13_Mastigodiaptomus_cf_albuquerquensis_Mexico_BOLDABZ7048
ZPII608_07_Mastigodiaptomus_patzcuarensis_Mexico_BOLDABZ7048
GBA14286_13_Mastigodiaptomus_cf_albuquerquensis_Mexico_BOLDABZ7048
ZPLIV486_11_Mastigodiaptomus_patzcuarensis_Mexico_BOLDABZ7048
ZPLIV581_11_Mastigodiaptomus_patzcuarensis_Mexico_BOLDABZ7048
BACZP392_16_Mastigodiaptomus_patzcuarensis_Mexico_BOLDABZ7048
ZPLMX175_06_Mastigodiaptomus_montezumae_Mexico_BOLDAAD9210
ZPLMX177_06_Mastigodiaptomus_montezumae_Mexico_BOLDAAD9210
ZPII1388_11_Mastigodiaptomus_montezumae_Mexico_BOLDAAD9210
ZPLIV682_11_Mastigodiaptomus_montezumae_Mexico_BOLDAAD9210
ZPLIV683_11_Mastigodiaptomus_montezumae_Mexico_BOLDAAD9210
ZPLIV684_11_Mastigodiaptomus_montezumae_Mexico_BOLDAAD9210
ZPII829_11_Mastigodiaptomus_montezumae_Mexico_BOLDAAD9210
GBA14279_13_Mastigodiaptomus_montezumae_Mexico_BOLDAAD9210
ZPLIV480_11_Mastigodiaptomus_montezumae_Mexico_BOLDAAD9210
ZPLIV575_11_Mastigodiaptomus_montezumae_Mexico_BOLDAAD9210
ZMIII890_12_Mastigodiaptomus_montezumae_Mexico_BOLDAAD9210
ZMIII985_12_Mastigodiaptomus_montezumae_Mexico_BOLDAAD9210
GBA14345_13_Mastigodiaptomus_montezumae_Mexico_BOLDAAD9210
GBA14346_13_Mastigodiaptomus_montezumae_Mexico_BOLDAAD9210
BACZP443_16_Mastigodiaptomus_montezumae_Mexico_BOLDAAD9210
MCM826_17_Mastigodiaptomus_montezumae_Mexico_BOLDAAD9210
MCM822_17_Mastigodiaptomus_montezumae_Mexico_BOLDAAD9210
MCM827_17_Mastigodiaptomus_montezumae_Mexico_BOLDAAD9210
MCM831_17_Mastigodiaptomus_montezumae_Mexico_BOLDAEE3563
MCM825_17_Mastigodiaptomus_montezumae_Mexico_BOLDAEE8353
ZPLMX179_06_Mastigodiaptomus_montezumae_Mexico_BOLDAAD9211
ZPII1385_11_Mastigodiaptomus_montezumae_Mexico_BOLDAAD9211
ZPLIV481_11_Mastigodiaptomus_montezumae_Mexico_BOLDAAD9211
ZPLIV576_11_Mastigodiaptomus_montezumae_Mexico_BOLDAAD9211
ZPLIV681_11_Mastigodiaptomus_montezumae_Mexico_BOLDAAD9211
ZPLMX563_06_Mastigodiaptomus_montezumae_Mexico_BOLDAAD9211
ZPLMX565_06_Mastigodiaptomus_montezumae_Mexico_BOLDAAD9211
ZPII1322_11_Mastigodiaptomus_montezumae_Mexico_BOLDAAD9211
ZPII1323_11_Mastigodiaptomus_montezumae_Mexico_BOLDAAD9211
ZPII1326_11_Mastigodiaptomus_montezumae_Mexico_BOLDAAD9211
ZPII1335_11_Mastigodiaptomus_montezumae_Mexico_BOLDAAD9211
ZPII1449_11_Mastigodiaptomus_montezumae_Mexico_BOLDAAD9211
ZPII1450_11_Mastigodiaptomus_montezumae_Mexico_BOLDAAD9211
ZPLIV479_11_Mastigodiaptomus_montezumae_Mexico_BOLDAAD9211
ZPLIV574_11_Mastigodiaptomus_montezumae_Mexico_BOLDAAD9211
ZPLIV745_11_Mastigodiaptomus_montezumae_Mexico_BOLDAAD9211
ZPLIV746_11_Mastigodiaptomus_montezumae_Mexico_BOLDAAD9211
ZMIII899_12_Mastigodiaptomus_montezumae_Mexico_BOLDAAD9211
ZMIII994_12_Mastigodiaptomus_montezumae_Mexico_BOLDAAD9211
GBA14343_13_Mastigodiaptomus_montezumae_Mexico_BOLDAAD9211
GBA14344_13_Mastigodiaptomus_montezumae_Mexico_BOLDAAD9211
ZPLMX564_06_Mastigodiaptomus_montezumae_Mexico_BOLDAAD9211
ZPLIV487_11_Mastigodiaptomus_montezumae_Mexico_BOLDAAD9211
ZPLIV582_11_Mastigodiaptomus_montezumae_Mexico_BOLDAAD9211
ZPII1448_11_Mastigodiaptomus_montezumae_Mexico_BOLDAAD9211
ZPLIV744_11_Mastigodiaptomus_montezumae_Mexico_BOLDAAD9211
MCM829_17_Mastigodiaptomus_montezumae_Mexico_BOLDAEE6706
MCM823_17_Mastigodiaptomus_montezumae_Mexico_BOLDADM1320
MCM824_17_Mastigodiaptomus_montezumae_Mexico_BOLDADM1317
ZPLMX193_06_Mastigodiaptomus_reidae_Mexico_BOLDAAC8649
ZPLMX553_06_Mastigodiaptomus_reidae_Mexico_BOLDAAC8649
ZPLMX194_06_Mastigodiaptomus_reidae_Mexico_BOLDAAC8649
ZPLMX243_06_Mastigodiaptomus_reidae_Mexico_BOLDAAC8649
ZPLMX547_06_Mastigodiaptomus_reidae_Mexico_BOLDAAC8649
ZPLMX548_06_Mastigodiaptomus_reidae_Mexico_BOLDAAC8649
ZPLMX552_06_Mastigodiaptomus_reidae_Mexico_BOLDAAC8649
ZPLMX549_06_Mastigodiaptomus_reidae_Mexico_BOLDAAC8649
ZPLMX196_06_Mastigodiaptomus_reidae_Mexico_BOLDAAC8649
ZPLMX234_06_Mastigodiaptomus_cf_reidae_Mexico_BOLDAAC8649
ZPLMX247_06_Mastigodiaptomus_cf_reidae_Mexico_BOLDAAC8649
ZPLMX546_06_Mastigodiaptomus_cf_reidae_Mexico_BOLDAAC8649
ZPLMX545_06_Mastigodiaptomus_cf_reidae_Mexico_BOLDAAC8649
ZPLMX550_06_Mastigodiaptomus_cf_reidae_Mexico_BOLDAAC8649
ZPLMX551_06_Mastigodiaptomus_cf_reidae_Mexico_BOLDAAC8649
ZPLMX556_06_Mastigodiaptomus_cf_reidae_Mexico_BOLDAAC8649
MASKM028_19_Mastigodiaptomus_reidae_Mexico_BOLDAAC8649
MASKM029_19_Mastigodiaptomus_reidae_Mexico_BOLDAAC8649
MASKM030_19_Mastigodiaptomus_reidae_Mexico_BOLDAAC8649
MASKM031_19_Mastigodiaptomus_reidae_Mexico_BOLDAAC8649
MASKM032_19_Mastigodiaptomus_reidae_Mexico_BOLDAAC8649
MASKM033_19_Mastigodiaptomus_reidae_Mexico_BOLDAAC8649
ZPLMX197_06_Mastigodiaptomus_siankaanensis_Mexico_
ZPLMX555_06_Mastigodiaptomus_siankaanensis_Mexico_BOLDAAF1222
EXDB800_21_Mastigodiaptomus_siankaanensis_Mexico_BOLDAAF1222
EXDB814_21_Mastigodiaptomus_siankaanensis_Mexico_BOLDAAF1222

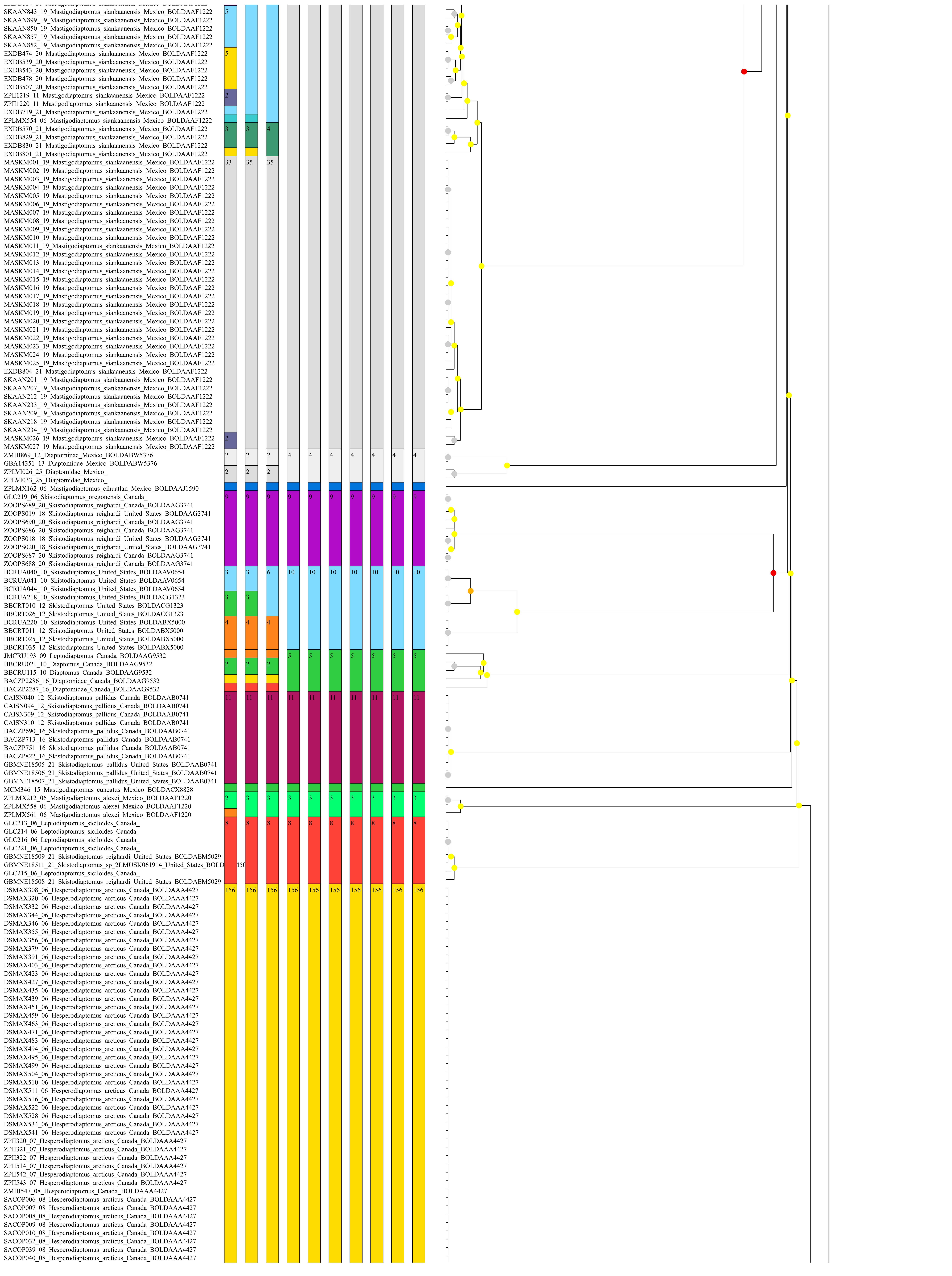
SKAAN843_19_Mastigodiaptomus_siankaanensis_Mexico_BOLDAAF1222
SKAAN899_19_Mastigodiaptomus_siankaanensis_Mexico_BOLDAAF1222
SKAAN850_19_Mastigodiaptomus_siankaanensis_Mexico_BOLDAAF1222
SKAAN857_19_Mastigodiaptomus_siankaanensis_Mexico_BOLDAAF1222
SKAAN852_19_Mastigodiaptomus_siankaanensis_Mexico_BOLDAAF1222
EXDB474_20_Mastigodiaptomus_siankaanensis_Mexico_BOLDAAF1222
EXDB539_20_Mastigodiaptomus_siankaanensis_Mexico_BOLDAAF1222
EXDB543_20_Mastigodiaptomus_siankaanensis_Mexico_BOLDAAF1222
EXDB478_20_Mastigodiaptomus_siankaanensis_Mexico_BOLDAAF1222
EXDB507_20_Mastigodiaptomus_siankaanensis_Mexico_BOLDAAF1222
ZPII1219_11_Mastigodiaptomus_siankaanensis_Mexico_BOLDAAF1222
ZPII1220_11_Mastigodiaptomus_siankaanensis_Mexico_BOLDAAF1222
EXDB719_21_Mastigodiaptomus_siankaanensis_Mexico_BOLDAAF1222
ZPLMX554_06_Mastigodiaptomus_siankaanensis_Mexico_BOLDAAF1222
EXDB570_21_Mastigodiaptomus_siankaanensis_Mexico_BOLDAAF1222
EXDB829_21_Mastigodiaptomus_siankaanensis_Mexico_BOLDAAF1222
EXDB830_21_Mastigodiaptomus_siankaanensis_Mexico_BOLDAAF1222
EXDB801_21_Mastigodiaptomus_siankaanensis_Mexico_BOLDAAF1222
MASKM001_19_Mastigodiaptomus_siankaanensis_Mexico_BOLDAAF1222
MASKM002_19_Mastigodiaptomus_siankaanensis_Mexico_BOLDAAF1222
MASKM003_19_Mastigodiaptomus_siankaanensis_Mexico_BOLDAAF1222
MASKM004_19_Mastigodiaptomus_siankaanensis_Mexico_BOLDAAF1222
MASKM005_19_Mastigodiaptomus_siankaanensis_Mexico_BOLDAAF1222
MASKM006_19_Mastigodiaptomus_siankaanensis_Mexico_BOLDAAF1222
MASKM007_19_Mastigodiaptomus_siankaanensis_Mexico_BOLDAAF1222
MASKM008_19_Mastigodiaptomus_siankaanensis_Mexico_BOLDAAF1222
MASKM009_19_Mastigodiaptomus_siankaanensis_Mexico_BOLDAAF1222
MASKM010_19_Mastigodiaptomus_siankaanensis_Mexico_BOLDAAF1222
MASKM011_19_Mastigodiaptomus_siankaanensis_Mexico_BOLDAAF1222
MASKM012_19_Mastigodiaptomus_siankaanensis_Mexico_BOLDAAF1222
MASKM013_19_Mastigodiaptomus_siankaanensis_Mexico_BOLDAAF1222
MASKM014_19_Mastigodiaptomus_siankaanensis_Mexico_BOLDAAF1222
MASKM015_19_Mastigodiaptomus_siankaanensis_Mexico_BOLDAAF1222
MASKM016_19_Mastigodiaptomus_siankaanensis_Mexico_BOLDAAF1222
MASKM017_19_Mastigodiaptomus_siankaanensis_Mexico_BOLDAAF1222
MASKM018_19_Mastigodiaptomus_siankaanensis_Mexico_BOLDAAF1222
MASKM019_19_Mastigodiaptomus_siankaanensis_Mexico_BOLDAAF1222
MASKM020_19_Mastigodiaptomus_siankaanensis_Mexico_BOLDAAF1222
MASKM021_19_Mastigodiaptomus_siankaanensis_Mexico_BOLDAAF1222
MASKM022_19_Mastigodiaptomus_siankaanensis_Mexico_BOLDAAF1222
MASKM023_19_Mastigodiaptomus_siankaanensis_Mexico_BOLDAAF1222
MASKM024_19_Mastigodiaptomus_siankaanensis_Mexico_BOLDAAF1222
MASKM025_19_Mastigodiaptomus_siankaanensis_Mexico_BOLDAAF1222
EXDB804_21_Mastigodiaptomus_siankaanensis_Mexico_BOLDAAF1222
SKAAN201_19_Mastigodiaptomus_siankaanensis_Mexico_BOLDAAF1222
SKAAN207_19_Mastigodiaptomus_siankaanensis_Mexico_BOLDAAF1222
SKAAN212_19_Mastigodiaptomus_siankaanensis_Mexico_BOLDAAF1222
SKAAN233_19_Mastigodiaptomus_siankaanensis_Mexico_BOLDAAF1222
SKAAN209_19_Mastigodiaptomus_siankaanensis_Mexico_BOLDAAF1222
SKAAN218_19_Mastigodiaptomus_siankaanensis_Mexico_BOLDAAF1222
SKAAN234_19_Mastigodiaptomus_siankaanensis_Mexico_BOLDAAF1222
MASKM026_19_Mastigodiaptomus_siankaanensis_Mexico_BOLDAAF1222
MASKM027_19_Mastigodiaptomus_siankaanensis_Mexico_BOLDAAF1222
ZMIII869_12_Diaptominae_Mexico_BOLDABW5376
GBA14351_13_Diaptomidae_Mexico_BOLDABW5376
ZPLVI026_25_Diaptomidae_Mexico_
ZPLVI033_25_Diaptomidae_Mexico_
ZPLMX162_06_Mastigodiaptomus_cihuatlan_Mexico_BOLDAAJ1590
GLC219_06_Skistodiaptomus_oregonensis_Canada_
ZOOPS689_20_Skistodiaptomus_reighardi_Canada_BOLDAAG3741
ZOOPS019_18_Skistodiaptomus_reighardi_United_States_BOLDAAG3741
ZOOPS690_20_Skistodiaptomus_reighardi_Canada_BOLDAAG3741
ZOOPS686_20_Skistodiaptomus_reighardi_Canada_BOLDAAG3741
ZOOPS018_18_Skistodiaptomus_reighardi_United_States_BOLDAAG3741
ZOOPS020_18_Skistodiaptomus_reighardi_United_States_BOLDAAG3741
ZOOPS687_20_Skistodiaptomus_reighardi_Canada_BOLDAAG3741
ZOOPS688_20_Skistodiaptomus_reighardi_Canada_BOLDAAG3741
BCRUA040_10_Skistodiaptomus_United_States_BOLDAAV0654
BCRUA041_10_Skistodiaptomus_United_States_BOLDAAV0654
BCRUA044_10_Skistodiaptomus_United_States_BOLDAAV0654
BCRUA218_10_Skistodiaptomus_United_States_BOLDACG1323
BBCRT010_12_Skistodiaptomus_United_States_BOLDACG1323
BBCRT026_12_Skistodiaptomus_United_States_BOLDACG1323
BCRUA220_10_Skistodiaptomus_United_States_BOLDABX5000
BBCRT011_12_Skistodiaptomus_United_States_BOLDABX5000
BBCRT025_12_Skistodiaptomus_United_States_BOLDABX5000
BBCRT035_12_Skistodiaptomus_United_States_BOLDABX5000
JMCRU193_09_Leptodiaptomus_Canada_BOLDAAG9532
BBCRU021_10_Diaptomus_Canada_BOLDAAG9532
BBCRU115_10_Diaptomus_Canada_BOLDAAG9532
BACZP2286_16_Diaptomidae_Canada_BOLDAAG9532
BACZP2287_16_Diaptomidae_Canada_BOLDAAG9532
CAISN040_12_Skistodiaptomus_pallidus_Canada_BOLDAAB0741
CAISN094_12_Skistodiaptomus_pallidus_Canada_BOLDAAB0741
CAISN309_12_Skistodiaptomus_pallidus_Canada_BOLDAAB0741
CAISN310_12_Skistodiaptomus_pallidus_Canada_BOLDAAB0741
BACZP690_16_Skistodiaptomus_pallidus_Canada_BOLDAAB0741
BACZP713_16_Skistodiaptomus_pallidus_Canada_BOLDAAB0741
BACZP751_16_Skistodiaptomus_pallidus_Canada_BOLDAAB0741
BACZP822_16_Skistodiaptomus_pallidus_Canada_BOLDAAB0741
GBMNE18505_21_Skistodiaptomus_pallidus_United_States_BOLDAAB0741
GBMNE18506_21_Skistodiaptomus_pallidus_United_States_BOLDAAB0741
GBMNE18507_21_Skistodiaptomus_pallidus_United_States_BOLDAAB0741
MCM346_15_Mastigodiaptomus_cuneatus_Mexico_BOLDACX8828
ZPLMX212_06_Mastigodiaptomus_alexei_Mexico_BOLDAAF1220
ZPLMX558_06_Mastigodiaptomus_alexei_Mexico_BOLDAAF1220
ZPLMX561_06_Mastigodiaptomus_alexei_Mexico_BOLDAAF1220
GLC213_06_Leptodiaptomus_siciloides_Canada_
GLC214_06_Leptodiaptomus_siciloides_Canada_
GLC216_06_Leptodiaptomus_siciloides_Canada_
GLC221_06_Leptodiaptomus_siciloides_Canada_
GBMNE18509_21_Skistodiaptomus_reighardi_United_States_BOLDAEM5029
GBMNE18511_21_Skistodiaptomus_sp_2LMUSK061914_United_States_BOLD
GLC215_06_Leptodiaptomus_siciloides_Canada_
GBMNE18508_21_Skistodiaptomus_reighardi_United_States_BOLDAEM5029
DSMAX308_06_Hesperodiaptomus_arcticus_Canada_BOLDAAA4427
DSMAX320_06_Hesperodiaptomus_arcticus_Canada_BOLDAAA4427
DSMAX332_06_Hesperodiaptomus_arcticus_Canada_BOLDAAA4427
DSMAX344_06_Hesperodiaptomus_arcticus_Canada_BOLDAAA4427
DSMAX355_06_Hesperodiaptomus_arcticus_Canada_BOLDAAA4427
DSMAX346_06_Hesperodiaptomus_arcticus_Canada_BOLDAAA4427
DSMAX356_06_Hesperodiaptomus_arcticus_Canada_BOLDAAA4427
DSMAX379_06_Hesperodiaptomus_arcticus_Canada_BOLDAAA4427
DSMAX391_06_Hesperodiaptomus_arcticus_Canada_BOLDAAA4427
DSMAX403_06_Hesperodiaptomus_arcticus_Canada_BOLDAAA4427
DSMAX423_06_Hesperodiaptomus_arcticus_Canada_BOLDAAA4427
DSMAX427_06_Hesperodiaptomus_arcticus_Canada_BOLDAAA4427
DSMAX439_06_Hesperodiaptomus_arcticus_Canada_BOLDAAA4427
DSMAX435_06_Hesperodiaptomus_arcticus_Canada_BOLDAAA4427
DSMAX451_06_Hesperodiaptomus_arcticus_Canada_BOLDAAA4427
DSMAX459_06_Hesperodiaptomus_arcticus_Canada_BOLDAAA4427
DSMAX463_06_Hesperodiaptomus_arcticus_Canada_BOLDAAA4427
DSMAX471_06_Hesperodiaptomus_arcticus_Canada_BOLDAAA4427
DSMAX483_06_Hesperodiaptomus_arcticus_Canada_BOLDAAA4427
DSMAX494_06_Hesperodiaptomus_arcticus_Canada_BOLDAAA4427
DSMAX495_06_Hesperodiaptomus_arcticus_Canada_BOLDAAA4427
DSMAX499_06_Hesperodiaptomus_arcticus_Canada_BOLDAAA4427
DSMAX504_06_Hesperodiaptomus_arcticus_Canada_BOLDAAA4427
DSMAX510_06_Hesperodiaptomus_arcticus_Canada_BOLDAAA4427
DSMAX511_06_Hesperodiaptomus_arcticus_Canada_BOLDAAA4427
DSMAX516_06_Hesperodiaptomus_arcticus_Canada_BOLDAAA4427
DSMAX522_06_Hesperodiaptomus_arcticus_Canada_BOLDAAA4427
DSMAX528_06_Hesperodiaptomus_arcticus_Canada_BOLDAAA4427
DSMAX534_06_Hesperodiaptomus_arcticus_Canada_BOLDAAA4427
DSMAX541_06_Hesperodiaptomus_arcticus_Canada_BOLDAAA4427
ZPII320_07_Hesperodiaptomus_arcticus_Canada_BOLDAAA4427
ZPII321_07_Hesperodiaptomus_arcticus_Canada_BOLDAAA4427
ZPII322_07_Hesperodiaptomus_arcticus_Canada_BOLDAAA4427
ZPII514_07_Hesperodiaptomus_arcticus_Canada_BOLDAAA4427
ZPII542_07_Hesperodiaptomus_arcticus_Canada_BOLDAAA4427
ZPII543_07_Hesperodiaptomus_arcticus_Canada_BOLDAAA4427
ZMIII547_08_Hesperodiaptomus_Canada_BOLDAAA4427
SACOP006_08_Hesperodiaptomus_arcticus_Canada_BOLDAAA4427
SACOP007_08_Hesperodiaptomus_arcticus_Canada_BOLDAAA4427
SACOP008_08_Hesperodiaptomus_arcticus_Canada_BOLDAAA4427
SACOP009_08_Hesperodiaptomus_arcticus_Canada_BOLDAAA4427
SACOP010_08_Hesperodiaptomus_arcticus_Canada_BOLDAAA4427
SACOP032_08_Hesperodiaptomus_arcticus_Canada_BOLDAAA4427
SACOP039_08_Hesperodiaptomus_arcticus_Canada_BOLDAAA4427
SACOP040_08_Hesperodiaptomus_arcticus_Canada_BOLDAAA4427

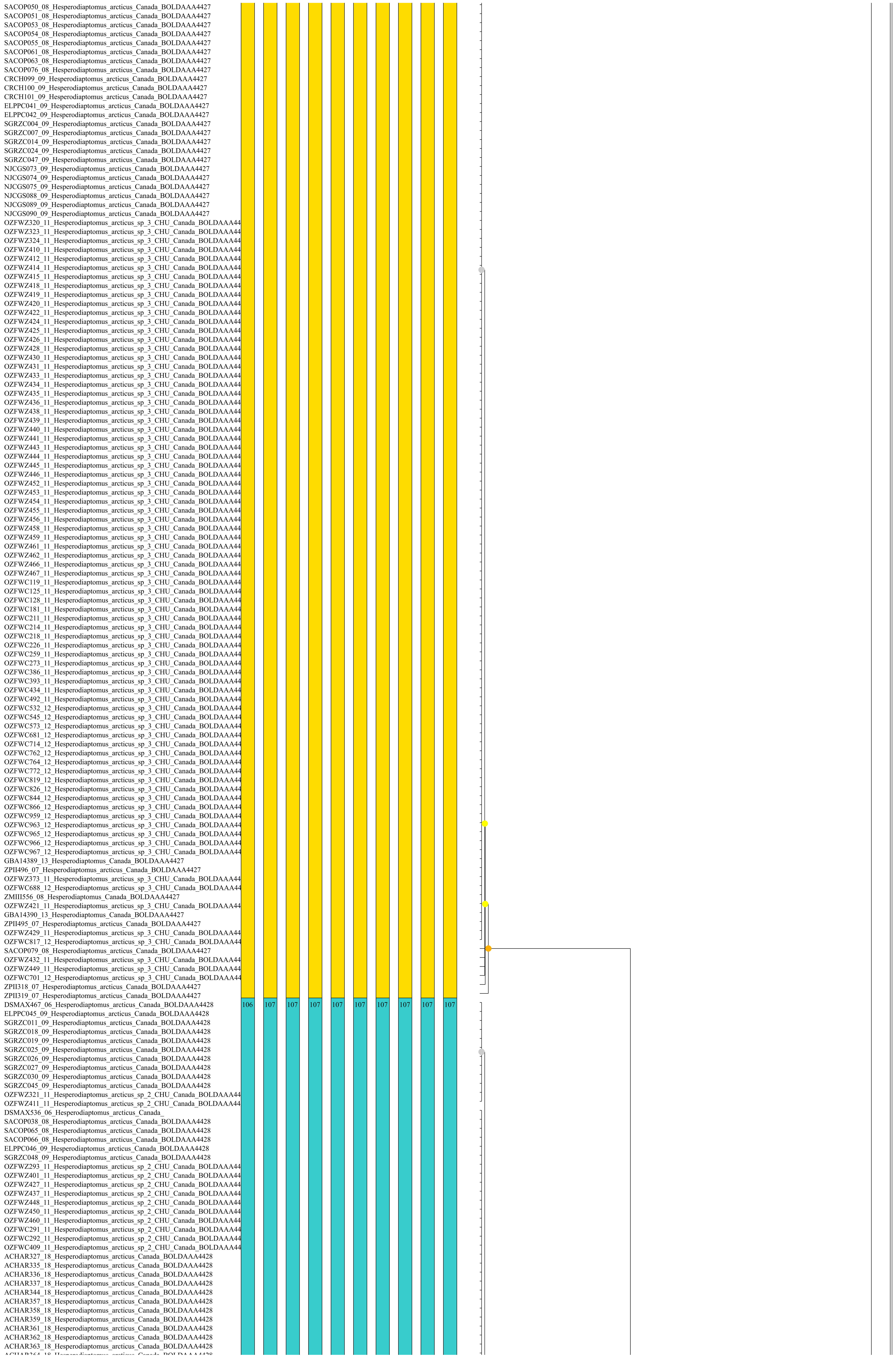
SACOP050_08_Hesperodiaptomus_arcticus_Canada_BOLDAAA4427
SACOP051_08_Hesperodiaptomus_arcticus_Canada_BOLDAAA4427
SACOP053_08_Hesperodiaptomus_arcticus_Canada_BOLDAAA4427
SACOP054_08_Hesperodiaptomus_arcticus_Canada_BOLDAAA4427
SACOP055_08_Hesperodiaptomus_arcticus_Canada_BOLDAAA4427
SACOP061_08_Hesperodiaptomus_arcticus_Canada_BOLDAAA4427
SACOP063_08_Hesperodiaptomus_arcticus_Canada_BOLDAAA4427
SACOP076_08_Hesperodiaptomus_arcticus_Canada_BOLDAAA4427
CRCH099_09_Hesperodiaptomus_arcticus_Canada_BOLDAAA4427
CRCH100_09_Hesperodiaptomus_arcticus_Canada_BOLDAAA4427
CRCH101_09_Hesperodiaptomus_arcticus_Canada_BOLDAAA4427
ELPPC041_09_Hesperodiaptomus_arcticus_Canada_BOLDAAA4427
ELPPC042_09_Hesperodiaptomus_arcticus_Canada_BOLDAAA4427
SGRZC004_09_Hesperodiaptomus_arcticus_Canada_BOLDAAA4427
SGRZC007_09_Hesperodiaptomus_arcticus_Canada_BOLDAAA4427
SGRZC014_09_Hesperodiaptomus_arcticus_Canada_BOLDAAA4427
SGRZC024_09_Hesperodiaptomus_arcticus_Canada_BOLDAAA4427
SGRZC047_09_Hesperodiaptomus_arcticus_Canada_BOLDAAA4427
NJCGS073_09_Hesperodiaptomus_arcticus_Canada_BOLDAAA4427
NJCGS074_09_Hesperodiaptomus_arcticus_Canada_BOLDAAA4427
NJCGS075_09_Hesperodiaptomus_arcticus_Canada_BOLDAAA4427
NJCGS088_09_Hesperodiaptomus_arcticus_Canada_BOLDAAA4427
NJCGS089_09_Hesperodiaptomus_arcticus_Canada_BOLDAAA4427
NJCGS090_09_Hesperodiaptomus_arcticus_Canada_BOLDAAA4427
OZFWZ320_11_Hesperodiaptomus_arcticus_sp_3_CHU_Canada_BOLDAAA44
OZFWZ323_11_Hesperodiaptomus_arcticus_sp_3_CHU_Canada_BOLDAAA44
OZFWZ324_11_Hesperodiaptomus_arcticus_sp_3_CHU_Canada_BOLDAAA44
OZFWZ410_11_Hesperodiaptomus_arcticus_sp_3_CHU_Canada_BOLDAAA44
OZFWZ412_11_Hesperodiaptomus_arcticus_sp_3_CHU_Canada_BOLDAAA44
OZFWZ414_11_Hesperodiaptomus_arcticus_sp_3_CHU_Canada_BOLDAAA44
OZFWZ415_11_Hesperodiaptomus_arcticus_sp_3_CHU_Canada_BOLDAAA44
OZFWZ418_11_Hesperodiaptomus_arcticus_sp_3_CHU_Canada_BOLDAAA44
OZFWZ419_11_Hesperodiaptomus_arcticus_sp_3_CHU_Canada_BOLDAAA44
OZFWZ420_11_Hesperodiaptomus_arcticus_sp_3_CHU_Canada_BOLDAAA44
OZFWZ422_11_Hesperodiaptomus_arcticus_sp_3_CHU_Canada_BOLDAAA44
OZFWZ424_11_Hesperodiaptomus_arcticus_sp_3_CHU_Canada_BOLDAAA44
OZFWZ425_11_Hesperodiaptomus_arcticus_sp_3_CHU_Canada_BOLDAAA44
OZFWZ426_11_Hesperodiaptomus_arcticus_sp_3_CHU_Canada_BOLDAAA44
OZFWZ428_11_Hesperodiaptomus_arcticus_sp_3_CHU_Canada_BOLDAAA44
OZFWZ430_11_Hesperodiaptomus_arcticus_sp_3_CHU_Canada_BOLDAAA44
OZFWZ431_11_Hesperodiaptomus_arcticus_sp_3_CHU_Canada_BOLDAAA44
OZFWZ433_11_Hesperodiaptomus_arcticus_sp_3_CHU_Canada_BOLDAAA44
OZFWZ434_11_Hesperodiaptomus_arcticus_sp_3_CHU_Canada_BOLDAAA44
OZFWZ435_11_Hesperodiaptomus_arcticus_sp_3_CHU_Canada_BOLDAAA44
OZFWZ436_11_Hesperodiaptomus_arcticus_sp_3_CHU_Canada_BOLDAAA44
OZFWZ438_11_Hesperodiaptomus_arcticus_sp_3_CHU_Canada_BOLDAAA44
OZFWZ439_11_Hesperodiaptomus_arcticus_sp_3_CHU_Canada_BOLDAAA44
OZFWZ440_11_Hesperodiaptomus_arcticus_sp_3_CHU_Canada_BOLDAAA44
OZFWZ441_11_Hesperodiaptomus_arcticus_sp_3_CHU_Canada_BOLDAAA44
OZFWZ443_11_Hesperodiaptomus_arcticus_sp_3_CHU_Canada_BOLDAAA44
OZFWZ444_11_Hesperodiaptomus_arcticus_sp_3_CHU_Canada_BOLDAAA44
OZFWZ445_11_Hesperodiaptomus_arcticus_sp_3_CHU_Canada_BOLDAAA44
OZFWZ446_11_Hesperodiaptomus_arcticus_sp_3_CHU_Canada_BOLDAAA44
OZFWZ452_11_Hesperodiaptomus_arcticus_sp_3_CHU_Canada_BOLDAAA44
OZFWZ453_11_Hesperodiaptomus_arcticus_sp_3_CHU_Canada_BOLDAAA44
OZFWZ454_11_Hesperodiaptomus_arcticus_sp_3_CHU_Canada_BOLDAAA44
OZFWZ455_11_Hesperodiaptomus_arcticus_sp_3_CHU_Canada_BOLDAAA44
OZFWZ456_11_Hesperodiaptomus_arcticus_sp_3_CHU_Canada_BOLDAAA44
OZFWZ458_11_Hesperodiaptomus_arcticus_sp_3_CHU_Canada_BOLDAAA44
OZFWZ459_11_Hesperodiaptomus_arcticus_sp_3_CHU_Canada_BOLDAAA44
OZFWZ461_11_Hesperodiaptomus_arcticus_sp_3_CHU_Canada_BOLDAAA44
OZFWZ462_11_Hesperodiaptomus_arcticus_sp_3_CHU_Canada_BOLDAAA44
OZFWZ466_11_Hesperodiaptomus_arcticus_sp_3_CHU_Canada_BOLDAAA44
OZFWZ467_11_Hesperodiaptomus_arcticus_sp_3_CHU_Canada_BOLDAAA44
OZFWC119_11_Hesperodiaptomus_arcticus_sp_3_CHU_Canada_BOLDAAA44
OZFWC125_11_Hesperodiaptomus_arcticus_sp_3_CHU_Canada_BOLDAAA44
OZFWC128_11_Hesperodiaptomus_arcticus_sp_3_CHU_Canada_BOLDAAA44
OZFWC181_11_Hesperodiaptomus_arcticus_sp_3_CHU_Canada_BOLDAAA44
OZFWC211_11_Hesperodiaptomus_arcticus_sp_3_CHU_Canada_BOLDAAA44
OZFWC214_11_Hesperodiaptomus_arcticus_sp_3_CHU_Canada_BOLDAAA44
OZFWC218_11_Hesperodiaptomus_arcticus_sp_3_CHU_Canada_BOLDAAA44
OZFWC226_11_Hesperodiaptomus_arcticus_sp_3_CHU_Canada_BOLDAAA44
OZFWC259_11_Hesperodiaptomus_arcticus_sp_3_CHU_Canada_BOLDAAA44
OZFWC273_11_Hesperodiaptomus_arcticus_sp_3_CHU_Canada_BOLDAAA44
OZFWC386_11_Hesperodiaptomus_arcticus_sp_3_CHU_Canada_BOLDAAA44
OZFWC393_11_Hesperodiaptomus_arcticus_sp_3_CHU_Canada_BOLDAAA44
OZFWC434_11_Hesperodiaptomus_arcticus_sp_3_CHU_Canada_BOLDAAA44
OZFWC492_11_Hesperodiaptomus_arcticus_sp_3_CHU_Canada_BOLDAAA44
OZFWC532_12_Hesperodiaptomus_arcticus_sp_3_CHU_Canada_BOLDAAA44
OZFWC545_12_Hesperodiaptomus_arcticus_sp_3_CHU_Canada_BOLDAAA44
OZFWC573_12_Hesperodiaptomus_arcticus_sp_3_CHU_Canada_BOLDAAA44
OZFWC681_12_Hesperodiaptomus_arcticus_sp_3_CHU_Canada_BOLDAAA44
OZFWC714_12_Hesperodiaptomus_arcticus_sp_3_CHU_Canada_BOLDAAA44
OZFWC762_12_Hesperodiaptomus_arcticus_sp_3_CHU_Canada_BOLDAAA44
OZFWC764_12_Hesperodiaptomus_arcticus_sp_3_CHU_Canada_BOLDAAA44
OZFWC772_12_Hesperodiaptomus_arcticus_sp_3_CHU_Canada_BOLDAAA44
OZFWC819_12_Hesperodiaptomus_arcticus_sp_3_CHU_Canada_BOLDAAA44
OZFWC826_12_Hesperodiaptomus_arcticus_sp_3_CHU_Canada_BOLDAAA44
OZFWC844_12_Hesperodiaptomus_arcticus_sp_3_CHU_Canada_BOLDAAA44
OZFWC866_12_Hesperodiaptomus_arcticus_sp_3_CHU_Canada_BOLDAAA44
OZFWC959_12_Hesperodiaptomus_arcticus_sp_3_CHU_Canada_BOLDAAA44
OZFWC963_12_Hesperodiaptomus_arcticus_sp_3_CHU_Canada_BOLDAAA44
OZFWC965_12_Hesperodiaptomus_arcticus_sp_3_CHU_Canada_BOLDAAA44
OZFWC966_12_Hesperodiaptomus_arcticus_sp_3_CHU_Canada_BOLDAAA44
OZFWC967_12_Hesperodiaptomus_arcticus_sp_3_CHU_Canada_BOLDAAA44
GBA14389_13_Hesperodiaptomus_Canada_BOLDAAA4427
ZPII496_07_Hesperodiaptomus_arcticus_Canada_BOLDAAA4427
OZFWZ373_11_Hesperodiaptomus_arcticus_sp_3_CHU_Canada_BOLDAAA44
OZFWC688_12_Hesperodiaptomus_arcticus_sp_3_CHU_Canada_BOLDAAA44
ZMIII556_08_Hesperodiaptomus_Canada_BOLDAAA4427
OZFWZ421_11_Hesperodiaptomus_arcticus_sp_3_CHU_Canada_BOLDAAA44
GBA14390_13_Hesperodiaptomus_Canada_BOLDAAA4427
ZPII495_07_Hesperodiaptomus_arcticus_Canada_BOLDAAA4427
OZFWZ429_11_Hesperodiaptomus_arcticus_sp_3_CHU_Canada_BOLDAAA44
OZFWC817_12_Hesperodiaptomus_arcticus_sp_3_CHU_Canada_BOLDAAA44
SACOP079_08_Hesperodiaptomus_arcticus_Canada_BOLDAAA4427
OZFWZ432_11_Hesperodiaptomus_arcticus_sp_3_CHU_Canada_BOLDAAA44
OZFWZ449_11_Hesperodiaptomus_arcticus_sp_3_CHU_Canada_BOLDAAA44
OZFWC701_12_Hesperodiaptomus_arcticus_sp_3_CHU_Canada_BOLDAAA44
ZPII318_07_Hesperodiaptomus_arcticus_Canada_BOLDAAA4427
ZPII319_07_Hesperodiaptomus_arcticus_Canada_BOLDAAA4427
DSMAX467_06_Hesperodiaptomus_arcticus_Canada_BOLDAAA4428
ELPPC045_09_Hesperodiaptomus_arcticus_Canada_BOLDAAA4428
SGRZC011_09_Hesperodiaptomus_arcticus_Canada_BOLDAAA4428
SGRZC018_09_Hesperodiaptomus_arcticus_Canada_BOLDAAA4428
SGRZC019_09_Hesperodiaptomus_arcticus_Canada_BOLDAAA4428
SGRZC025_09_Hesperodiaptomus_arcticus_Canada_BOLDAAA4428
SGRZC026_09_Hesperodiaptomus_arcticus_Canada_BOLDAAA4428
SGRZC027_09_Hesperodiaptomus_arcticus_Canada_BOLDAAA4428
SGRZC030_09_Hesperodiaptomus_arcticus_Canada_BOLDAAA4428
SGRZC045_09_Hesperodiaptomus_arcticus_Canada_BOLDAAA4428
OZFWZ321_11_Hesperodiaptomus_arcticus_sp_2_CHU_Canada_BOLDAAA44
OZFWZ411_11_Hesperodiaptomus_arcticus_sp_2_CHU_Canada_BOLDAAA44
DSMAX536_06_Hesperodiaptomus_arcticus_Canada_
SACOP038_08_Hesperodiaptomus_arcticus_Canada_BOLDAAA4428
SACOP065_08_Hesperodiaptomus_arcticus_Canada_BOLDAAA4428
SACOP066_08_Hesperodiaptomus_arcticus_Canada_BOLDAAA4428
ELPPC046_09_Hesperodiaptomus_arcticus_Canada_BOLDAAA4428
SGRZC048_09_Hesperodiaptomus_arcticus_Canada_BOLDAAA4428
OZFWZ293_11_Hesperodiaptomus_arcticus_sp_2_CHU_Canada_BOLDAAA44
OZFWZ401_11_Hesperodiaptomus_arcticus_sp_2_CHU_Canada_BOLDAAA44
OZFWZ427_11_Hesperodiaptomus_arcticus_sp_2_CHU_Canada_BOLDAAA44
OZFWZ437_11_Hesperodiaptomus_arcticus_sp_2_CHU_Canada_BOLDAAA44
OZFWZ448_11_Hesperodiaptomus_arcticus_sp_2_CHU_Canada_BOLDAAA44
OZFWZ450_11_Hesperodiaptomus_arcticus_sp_2_CHU_Canada_BOLDAAA44
OZFWZ460_11_Hesperodiaptomus_arcticus_sp_2_CHU_Canada_BOLDAAA44
OZFWC291_11_Hesperodiaptomus_arcticus_sp_2_CHU_Canada_BOLDAAA44
OZFWC292_11_Hesperodiaptomus_arcticus_sp_2_CHU_Canada_BOLDAAA44
OZFWC409_11_Hesperodiaptomus_arcticus_sp_2_CHU_Canada_BOLDAAA44
ACHAR327_18_Hesperodiaptomus_arcticus_Canada_BOLDAAA4428
ACHAR335_18_Hesperodiaptomus_arcticus_Canada_BOLDAAA4428
ACHAR336_18_Hesperodiaptomus_arcticus_Canada_BOLDAAA4428
ACHAR337_18_Hesperodiaptomus_arcticus_Canada_BOLDAAA4428
ACHAR344_18_Hesperodiaptomus_arcticus_Canada_BOLDAAA4428
ACHAR357_18_Hesperodiaptomus_arcticus_Canada_BOLDAAA4428
ACHAR358_18_Hesperodiaptomus_arcticus_Canada_BOLDAAA4428
ACHAR359_18_Hesperodiaptomus_arcticus_Canada_BOLDAAA4428
ACHAR361_18_Hesperodiaptomus_arcticus_Canada_BOLDAAA4428
ACHAR362_18_Hesperodiaptomus_arcticus_Canada_BOLDAAA4428
ACHAR363_18_Hesperodiaptomus_arcticus_Canada_BOLDAAA4428
106
107
107
107
107
107
107
107
107
107

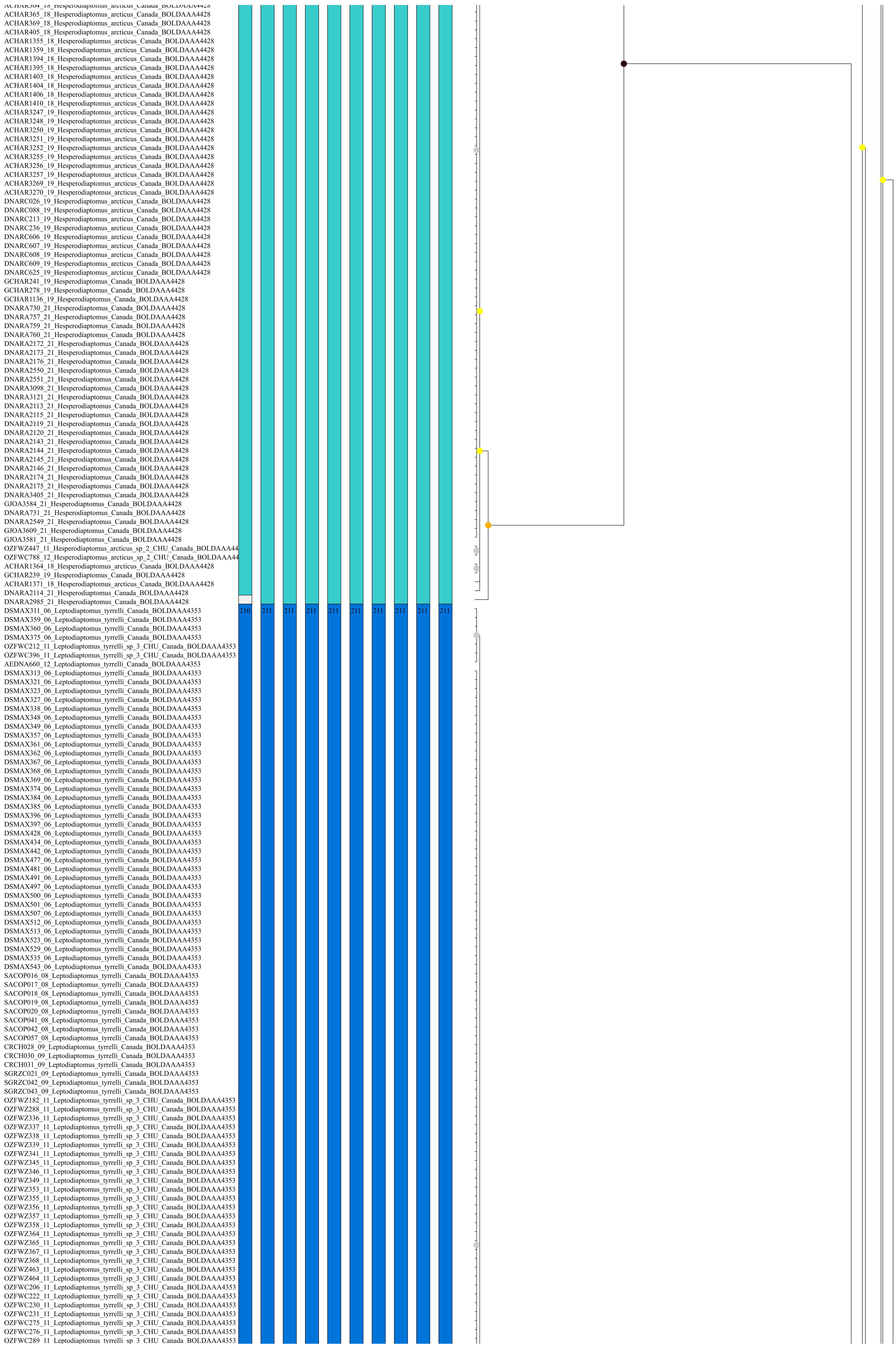

ACHAR364_18_Hesperodiaptomus_arcticus_Canada_BOLDAAA4428
ACHAR365_18_Hesperodiaptomus_arcticus_Canada_BOLDAAA4428
ACHAR369_18_Hesperodiaptomus_arcticus_Canada_BOLDAAA4428
ACHAR405_18_Hesperodiaptomus_arcticus_Canada_BOLDAAA4428
ACHAR1355_18_Hesperodiaptomus_arcticus_Canada_BOLDAAA4428
ACHAR1359_18_Hesperodiaptomus_arcticus_Canada_BOLDAAA4428
ACHAR1394_18_Hesperodiaptomus_arcticus_Canada_BOLDAAA4428
ACHAR1395_18_Hesperodiaptomus_arcticus_Canada_BOLDAAA4428
ACHAR1403_18_Hesperodiaptomus_arcticus_Canada_BOLDAAA4428
ACHAR1404_18_Hesperodiaptomus_arcticus_Canada_BOLDAAA4428
ACHAR1406_18_Hesperodiaptomus_arcticus_Canada_BOLDAAA4428
ACHAR1410_18_Hesperodiaptomus_arcticus_Canada_BOLDAAA4428
ACHAR3247_19_Hesperodiaptomus_arcticus_Canada_BOLDAAA4428
ACHAR3248_19_Hesperodiaptomus_arcticus_Canada_BOLDAAA4428
ACHAR3250_19_Hesperodiaptomus_arcticus_Canada_BOLDAAA4428
ACHAR3251_19_Hesperodiaptomus_arcticus_Canada_BOLDAAA4428
ACHAR3252_19_Hesperodiaptomus_arcticus_Canada_BOLDAAA4428
ACHAR3255_19_Hesperodiaptomus_arcticus_Canada_BOLDAAA4428
ACHAR3256_19_Hesperodiaptomus_arcticus_Canada_BOLDAAA4428
ACHAR3257_19_Hesperodiaptomus_arcticus_Canada_BOLDAAA4428
ACHAR3269_19_Hesperodiaptomus_arcticus_Canada_BOLDAAA4428
ACHAR3270_19_Hesperodiaptomus_arcticus_Canada_BOLDAAA4428
DNARC026_19_Hesperodiaptomus_arcticus_Canada_BOLDAAA4428
DNARC088_19_Hesperodiaptomus_arcticus_Canada_BOLDAAA4428
DNARC213_19_Hesperodiaptomus_arcticus_Canada_BOLDAAA4428
DNARC236_19_Hesperodiaptomus_arcticus_Canada_BOLDAAA4428
DNARC606_19_Hesperodiaptomus_arcticus_Canada_BOLDAAA4428
DNARC607_19_Hesperodiaptomus_arcticus_Canada_BOLDAAA4428
DNARC608_19_Hesperodiaptomus_arcticus_Canada_BOLDAAA4428
DNARC609_19_Hesperodiaptomus_arcticus_Canada_BOLDAAA4428
DNARC625_19_Hesperodiaptomus_arcticus_Canada_BOLDAAA4428
GCHAR241_19_Hesperodiaptomus_Canada_BOLDAAA4428
GCHAR278_19_Hesperodiaptomus_Canada_BOLDAAA4428
GCHAR1136_19_Hesperodiaptomus_Canada_BOLDAAA4428
DNARA730_21_Hesperodiaptomus_Canada_BOLDAAA4428
DNARA757_21_Hesperodiaptomus_Canada_BOLDAAA4428
DNARA759_21_Hesperodiaptomus_Canada_BOLDAAA4428
DNARA760_21_Hesperodiaptomus_Canada_BOLDAAA4428
DNARA2172_21_Hesperodiaptomus_Canada_BOLDAAA4428
DNARA2173_21_Hesperodiaptomus_Canada_BOLDAAA4428
DNARA2176_21_Hesperodiaptomus_Canada_BOLDAAA4428
DNARA2550_21_Hesperodiaptomus_Canada_BOLDAAA4428
DNARA2551_21_Hesperodiaptomus_Canada_BOLDAAA4428
DNARA3098_21_Hesperodiaptomus_Canada_BOLDAAA4428
DNARA3121_21_Hesperodiaptomus_Canada_BOLDAAA4428
DNARA2113_21_Hesperodiaptomus_Canada_BOLDAAA4428
DNARA2115_21_Hesperodiaptomus_Canada_BOLDAAA4428
DNARA2119_21_Hesperodiaptomus_Canada_BOLDAAA4428
DNARA2120_21_Hesperodiaptomus_Canada_BOLDAAA4428
DNARA2143_21_Hesperodiaptomus_Canada_BOLDAAA4428
DNARA2144_21_Hesperodiaptomus_Canada_BOLDAAA4428
DNARA2145_21_Hesperodiaptomus_Canada_BOLDAAA4428
DNARA2146_21_Hesperodiaptomus_Canada_BOLDAAA4428
DNARA2174_21_Hesperodiaptomus_Canada_BOLDAAA4428
DNARA2175_21_Hesperodiaptomus_Canada_BOLDAAA4428
DNARA3405_21_Hesperodiaptomus_Canada_BOLDAAA4428
GJOA3584_21_Hesperodiaptomus_Canada_BOLDAAA4428
DNARA731_21_Hesperodiaptomus_Canada_BOLDAAA4428
DNARA2549_21_Hesperodiaptomus_Canada_BOLDAAA4428
GJOA3609_21_Hesperodiaptomus_Canada_BOLDAAA4428
GJOA3581_21_Hesperodiaptomus_Canada_BOLDAAA4428
OZFWZ447_11_Hesperodiaptomus_arcticus_sp_2_CHU_Canada_BOLDAAA44
OZFWC788_12_Hesperodiaptomus_arcticus_sp_2_CHU_Canada_BOLDAAA44
ACHAR1364_18_Hesperodiaptomus_arcticus_Canada_BOLDAAA4428
GCHAR239_19_Hesperodiaptomus_Canada_BOLDAAA4428
ACHAR1371_18_Hesperodiaptomus_arcticus_Canada_BOLDAAA4428
DNARA2114_21_Hesperodiaptomus_Canada_BOLDAAA4428
DNARA2985_21_Hesperodiaptomus_Canada_BOLDAAA4428
DSMAX311_06_Leptodiaptomus_tyrrelli_Canada_BOLDAAA4353
210
211
211
211
211
211
211
211
211
211
DSMAX359_06_Leptodiaptomus_tyrrelli_Canada_BOLDAAA4353
DSMAX360_06_Leptodiaptomus_tyrrelli_Canada_BOLDAAA4353
DSMAX375_06_Leptodiaptomus_tyrrelli_Canada_BOLDAAA4353
OZFWC212_11_Leptodiaptomus_tyrrelli_sp_3_CHU_Canada_BOLDAAA4353
OZFWC396_11_Leptodiaptomus_tyrrelli_sp_3_CHU_Canada_BOLDAAA4353
AEDNA660_12_Leptodiaptomus_tyrrelli_Canada_BOLDAAA4353
DSMAX313_06_Leptodiaptomus_tyrrelli_Canada_BOLDAAA4353
DSMAX321_06_Leptodiaptomus_tyrrelli_Canada_BOLDAAA4353
DSMAX323_06_Leptodiaptomus_tyrrelli_Canada_BOLDAAA4353
DSMAX327_06_Leptodiaptomus_tyrrelli_Canada_BOLDAAA4353
DSMAX338_06_Leptodiaptomus_tyrrelli_Canada_BOLDAAA4353
DSMAX348_06_Leptodiaptomus_tyrrelli_Canada_BOLDAAA4353
DSMAX349_06_Leptodiaptomus_tyrrelli_Canada_BOLDAAA4353
DSMAX357_06_Leptodiaptomus_tyrrelli_Canada_BOLDAAA4353
DSMAX361_06_Leptodiaptomus_tyrrelli_Canada_BOLDAAA4353
DSMAX362_06_Leptodiaptomus_tyrrelli_Canada_BOLDAAA4353
DSMAX367_06_Leptodiaptomus_tyrrelli_Canada_BOLDAAA4353
DSMAX368_06_Leptodiaptomus_tyrrelli_Canada_BOLDAAA4353
DSMAX369_06_Leptodiaptomus_tyrrelli_Canada_BOLDAAA4353
DSMAX374_06_Leptodiaptomus_tyrrelli_Canada_BOLDAAA4353
DSMAX384_06_Leptodiaptomus_tyrrelli_Canada_BOLDAAA4353
DSMAX385_06_Leptodiaptomus_tyrrelli_Canada_BOLDAAA4353
DSMAX396_06_Leptodiaptomus_tyrrelli_Canada_BOLDAAA4353
DSMAX397_06_Leptodiaptomus_tyrrelli_Canada_BOLDAAA4353
DSMAX428_06_Leptodiaptomus_tyrrelli_Canada_BOLDAAA4353
DSMAX434_06_Leptodiaptomus_tyrrelli_Canada_BOLDAAA4353
DSMAX442_06_Leptodiaptomus_tyrrelli_Canada_BOLDAAA4353
DSMAX477_06_Leptodiaptomus_tyrrelli_Canada_BOLDAAA4353
DSMAX481_06_Leptodiaptomus_tyrrelli_Canada_BOLDAAA4353
DSMAX491_06_Leptodiaptomus_tyrrelli_Canada_BOLDAAA4353
DSMAX497_06_Leptodiaptomus_tyrrelli_Canada_BOLDAAA4353
DSMAX500_06_Leptodiaptomus_tyrrelli_Canada_BOLDAAA4353
DSMAX501_06_Leptodiaptomus_tyrrelli_Canada_BOLDAAA4353
DSMAX507_06_Leptodiaptomus_tyrrelli_Canada_BOLDAAA4353
DSMAX512_06_Leptodiaptomus_tyrrelli_Canada_BOLDAAA4353
DSMAX513_06_Leptodiaptomus_tyrrelli_Canada_BOLDAAA4353
DSMAX523_06_Leptodiaptomus_tyrrelli_Canada_BOLDAAA4353
DSMAX529_06_Leptodiaptomus_tyrrelli_Canada_BOLDAAA4353
DSMAX535_06_Leptodiaptomus_tyrrelli_Canada_BOLDAAA4353
DSMAX543_06_Leptodiaptomus_tyrrelli_Canada_BOLDAAA4353
SACOP016_08_Leptodiaptomus_tyrrelli_Canada_BOLDAAA4353
SACOP017_08_Leptodiaptomus_tyrrelli_Canada_BOLDAAA4353
SACOP018_08_Leptodiaptomus_tyrrelli_Canada_BOLDAAA4353
SACOP019_08_Leptodiaptomus_tyrrelli_Canada_BOLDAAA4353
SACOP020_08_Leptodiaptomus_tyrrelli_Canada_BOLDAAA4353
SACOP041_08_Leptodiaptomus_tyrrelli_Canada_BOLDAAA4353
SACOP042_08_Leptodiaptomus_tyrrelli_Canada_BOLDAAA4353
SACOP057_08_Leptodiaptomus_tyrrelli_Canada_BOLDAAA4353
CRCH028_09_Leptodiaptomus_tyrrelli_Canada_BOLDAAA4353
CRCH030_09_Leptodiaptomus_tyrrelli_Canada_BOLDAAA4353
CRCH031_09_Leptodiaptomus_tyrrelli_Canada_BOLDAAA4353
SGRZC021_09_Leptodiaptomus_tyrrelli_Canada_BOLDAAA4353
SGRZC042_09_Leptodiaptomus_tyrrelli_Canada_BOLDAAA4353
SGRZC043_09_Leptodiaptomus_tyrrelli_Canada_BOLDAAA4353
OZFWZ182_11_Leptodiaptomus_tyrrelli_sp_3_CHU_Canada_BOLDAAA4353
OZFWZ288_11_Leptodiaptomus_tyrrelli_sp_3_CHU_Canada_BOLDAAA4353
OZFWZ336_11_Leptodiaptomus_tyrrelli_sp_3_CHU_Canada_BOLDAAA4353
OZFWZ337_11_Leptodiaptomus_tyrrelli_sp_3_CHU_Canada_BOLDAAA4353
OZFWZ338_11_Leptodiaptomus_tyrrelli_sp_3_CHU_Canada_BOLDAAA4353
OZFWZ339_11_Leptodiaptomus_tyrrelli_sp_3_CHU_Canada_BOLDAAA4353
OZFWZ341_11_Leptodiaptomus_tyrrelli_sp_3_CHU_Canada_BOLDAAA4353
OZFWZ345_11_Leptodiaptomus_tyrrelli_sp_3_CHU_Canada_BOLDAAA4353
OZFWZ346_11_Leptodiaptomus_tyrrelli_sp_3_CHU_Canada_BOLDAAA4353
OZFWZ349_11_Leptodiaptomus_tyrrelli_sp_3_CHU_Canada_BOLDAAA4353
OZFWZ353_11_Leptodiaptomus_tyrrelli_sp_3_CHU_Canada_BOLDAAA4353
OZFWZ355_11_Leptodiaptomus_tyrrelli_sp_3_CHU_Canada_BOLDAAA4353
OZFWZ356_11_Leptodiaptomus_tyrrelli_sp_3_CHU_Canada_BOLDAAA4353
OZFWZ357_11_Leptodiaptomus_tyrrelli_sp_3_CHU_Canada_BOLDAAA4353
OZFWZ358_11_Leptodiaptomus_tyrrelli_sp_3_CHU_Canada_BOLDAAA4353
OZFWZ364_11_Leptodiaptomus_tyrrelli_sp_3_CHU_Canada_BOLDAAA4353
OZFWZ365_11_Leptodiaptomus_tyrrelli_sp_3_CHU_Canada_BOLDAAA4353
OZFWZ367_11_Leptodiaptomus_tyrrelli_sp_3_CHU_Canada_BOLDAAA4353
OZFWZ368_11_Leptodiaptomus_tyrrelli_sp_3_CHU_Canada_BOLDAAA4353
OZFWZ463_11_Leptodiaptomus_tyrrelli_sp_3_CHU_Canada_BOLDAAA4353
OZFWZ464_11_Leptodiaptomus_tyrrelli_sp_3_CHU_Canada_BOLDAAA4353
OZFWC206_11_Leptodiaptomus_tyrrelli_sp_3_CHU_Canada_BOLDAAA4353
OZFWC222_11_Leptodiaptomus_tyrrelli_sp_3_CHU_Canada_BOLDAAA4353
OZFWC230_11_Leptodiaptomus_tyrrelli_sp_3_CHU_Canada_BOLDAAA4353
OZFWC231_11_Leptodiaptomus_tyrrelli_sp_3_CHU_Canada_BOLDAAA4353
OZFWC275_11_Leptodiaptomus_tyrrelli_sp_3_CHU_Canada_BOLDAAA4353
OZFWC276_11_Leptodiaptomus_tyrrelli_sp_3_CHU_Canada_BOLDAAA4353
OZFWC289_11_Leptodiaptomus_tyrrelli_sp_3_CHU_Canada_BOLDAAA4353

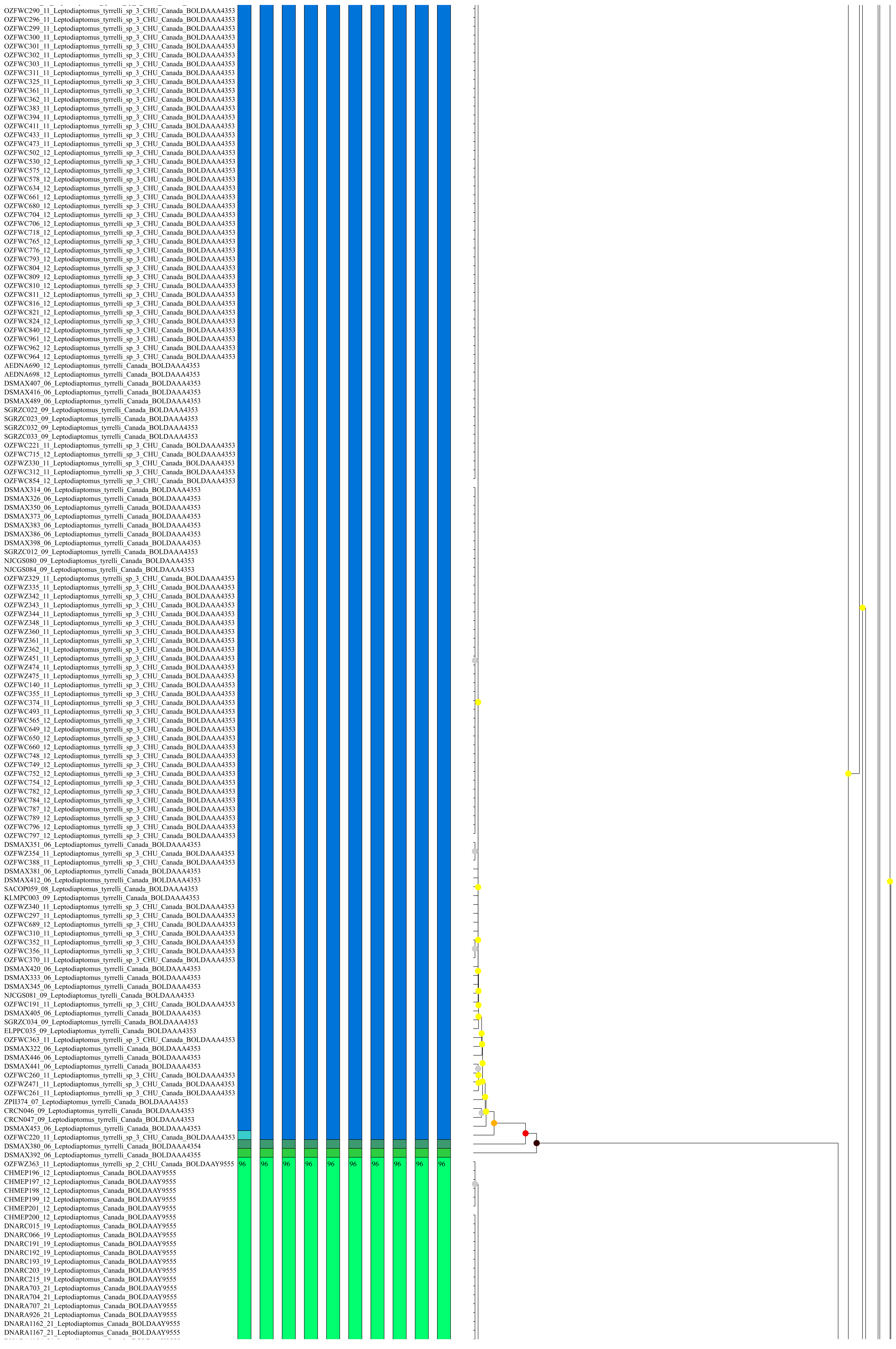

OZFWC290_11_Leptodiaptomus_tyrrelli_sp_3_CHU_Canada_BOLDAAA4353
OZFWC296_11_Leptodiaptomus_tyrrelli_sp_3_CHU_Canada_BOLDAAA4353
OZFWC299_11_Leptodiaptomus_tyrrelli_sp_3_CHU_Canada_BOLDAAA4353
OZFWC300_11_Leptodiaptomus_tyrrelli_sp_3_CHU_Canada_BOLDAAA4353
OZFWC301_11_Leptodiaptomus_tyrrelli_sp_3_CHU_Canada_BOLDAAA4353
OZFWC302_11_Leptodiaptomus_tyrrelli_sp_3_CHU_Canada_BOLDAAA4353
OZFWC303_11_Leptodiaptomus_tyrrelli_sp_3_CHU_Canada_BOLDAAA4353
OZFWC311_11_Leptodiaptomus_tyrrelli_sp_3_CHU_Canada_BOLDAAA4353
OZFWC325_11_Leptodiaptomus_tyrrelli_sp_3_CHU_Canada_BOLDAAA4353
OZFWC361_11_Leptodiaptomus_tyrrelli_sp_3_CHU_Canada_BOLDAAA4353
OZFWC362_11_Leptodiaptomus_tyrrelli_sp_3_CHU_Canada_BOLDAAA4353
OZFWC383_11_Leptodiaptomus_tyrrelli_sp_3_CHU_Canada_BOLDAAA4353
OZFWC394_11_Leptodiaptomus_tyrrelli_sp_3_CHU_Canada_BOLDAAA4353
OZFWC411_11_Leptodiaptomus_tyrrelli_sp_3_CHU_Canada_BOLDAAA4353
OZFWC433_11_Leptodiaptomus_tyrrelli_sp_3_CHU_Canada_BOLDAAA4353
OZFWC473_11_Leptodiaptomus_tyrrelli_sp_3_CHU_Canada_BOLDAAA4353
OZFWC502_12_Leptodiaptomus_tyrrelli_sp_3_CHU_Canada_BOLDAAA4353
OZFWC530_12_Leptodiaptomus_tyrrelli_sp_3_CHU_Canada_BOLDAAA4353
OZFWC575_12_Leptodiaptomus_tyrrelli_sp_3_CHU_Canada_BOLDAAA4353
OZFWC578_12_Leptodiaptomus_tyrrelli_sp_3_CHU_Canada_BOLDAAA4353
OZFWC634_12_Leptodiaptomus_tyrrelli_sp_3_CHU_Canada_BOLDAAA4353
OZFWC661_12_Leptodiaptomus_tyrrelli_sp_3_CHU_Canada_BOLDAAA4353
OZFWC680_12_Leptodiaptomus_tyrrelli_sp_3_CHU_Canada_BOLDAAA4353
OZFWC704_12_Leptodiaptomus_tyrrelli_sp_3_CHU_Canada_BOLDAAA4353
OZFWC706_12_Leptodiaptomus_tyrrelli_sp_3_CHU_Canada_BOLDAAA4353
OZFWC718_12_Leptodiaptomus_tyrrelli_sp_3_CHU_Canada_BOLDAAA4353
OZFWC765_12_Leptodiaptomus_tyrrelli_sp_3_CHU_Canada_BOLDAAA4353
OZFWC776_12_Leptodiaptomus_tyrrelli_sp_3_CHU_Canada_BOLDAAA4353
OZFWC793_12_Leptodiaptomus_tyrrelli_sp_3_CHU_Canada_BOLDAAA4353
OZFWC804_12_Leptodiaptomus_tyrrelli_sp_3_CHU_Canada_BOLDAAA4353
OZFWC809_12_Leptodiaptomus_tyrrelli_sp_3_CHU_Canada_BOLDAAA4353
OZFWC810_12_Leptodiaptomus_tyrrelli_sp_3_CHU_Canada_BOLDAAA4353
OZFWC811_12_Leptodiaptomus_tyrrelli_sp_3_CHU_Canada_BOLDAAA4353
OZFWC816_12_Leptodiaptomus_tyrrelli_sp_3_CHU_Canada_BOLDAAA4353
OZFWC821_12_Leptodiaptomus_tyrrelli_sp_3_CHU_Canada_BOLDAAA4353
OZFWC824_12_Leptodiaptomus_tyrrelli_sp_3_CHU_Canada_BOLDAAA4353
OZFWC840_12_Leptodiaptomus_tyrrelli_sp_3_CHU_Canada_BOLDAAA4353
OZFWC961_12_Leptodiaptomus_tyrrelli_sp_3_CHU_Canada_BOLDAAA4353
OZFWC962_12_Leptodiaptomus_tyrrelli_sp_3_CHU_Canada_BOLDAAA4353
OZFWC964_12_Leptodiaptomus_tyrrelli_sp_3_CHU_Canada_BOLDAAA4353
AEDNA690_12_Leptodiaptomus_tyrrelli_Canada_BOLDAAA4353
AEDNA698_12_Leptodiaptomus_tyrrelli_Canada_BOLDAAA4353
DSMAX407_06_Leptodiaptomus_tyrrelli_Canada_BOLDAAA4353
DSMAX416_06_Leptodiaptomus_tyrrelli_Canada_BOLDAAA4353
DSMAX489_06_Leptodiaptomus_tyrrelli_Canada_BOLDAAA4353
SGRZC022_09_Leptodiaptomus_tyrrelli_Canada_BOLDAAA4353
SGRZC023_09_Leptodiaptomus_tyrrelli_Canada_BOLDAAA4353
SGRZC032_09_Leptodiaptomus_tyrrelli_Canada_BOLDAAA4353
SGRZC033_09_Leptodiaptomus_tyrrelli_Canada_BOLDAAA4353
OZFWC221_11_Leptodiaptomus_tyrrelli_sp_3_CHU_Canada_BOLDAAA4353
OZFWC715_12_Leptodiaptomus_tyrrelli_sp_3_CHU_Canada_BOLDAAA4353
OZFWZ330_11_Leptodiaptomus_tyrrelli_sp_3_CHU_Canada_BOLDAAA4353
OZFWC312_11_Leptodiaptomus_tyrrelli_sp_3_CHU_Canada_BOLDAAA4353
OZFWC854_12_Leptodiaptomus_tyrrelli_sp_3_CHU_Canada_BOLDAAA4353
DSMAX314_06_Leptodiaptomus_tyrrelli_Canada_BOLDAAA4353
DSMAX326_06_Leptodiaptomus_tyrrelli_Canada_BOLDAAA4353
DSMAX350_06_Leptodiaptomus_tyrrelli_Canada_BOLDAAA4353
DSMAX373_06_Leptodiaptomus_tyrrelli_Canada_BOLDAAA4353
DSMAX383_06_Leptodiaptomus_tyrrelli_Canada_BOLDAAA4353
DSMAX386_06_Leptodiaptomus_tyrrelli_Canada_BOLDAAA4353
DSMAX398_06_Leptodiaptomus_tyrrelli_Canada_BOLDAAA4353
SGRZC012_09_Leptodiaptomus_tyrrelli_Canada_BOLDAAA4353
NJCGS080_09_Leptodiaptomus_tyrelli_Canada_BOLDAAA4353
NJCGS084_09_Leptodiaptomus_tyrelli_Canada_BOLDAAA4353
OZFWZ329_11_Leptodiaptomus_tyrrelli_sp_3_CHU_Canada_BOLDAAA4353
OZFWZ335_11_Leptodiaptomus_tyrrelli_sp_3_CHU_Canada_BOLDAAA4353
OZFWZ342_11_Leptodiaptomus_tyrrelli_sp_3_CHU_Canada_BOLDAAA4353
OZFWZ343_11_Leptodiaptomus_tyrrelli_sp_3_CHU_Canada_BOLDAAA4353
OZFWZ344_11_Leptodiaptomus_tyrrelli_sp_3_CHU_Canada_BOLDAAA4353
OZFWZ348_11_Leptodiaptomus_tyrrelli_sp_3_CHU_Canada_BOLDAAA4353
OZFWZ360_11_Leptodiaptomus_tyrrelli_sp_3_CHU_Canada_BOLDAAA4353
OZFWZ361_11_Leptodiaptomus_tyrrelli_sp_3_CHU_Canada_BOLDAAA4353
OZFWZ362_11_Leptodiaptomus_tyrrelli_sp_3_CHU_Canada_BOLDAAA4353
OZFWZ451_11_Leptodiaptomus_tyrrelli_sp_3_CHU_Canada_BOLDAAA4353
OZFWZ474_11_Leptodiaptomus_tyrrelli_sp_3_CHU_Canada_BOLDAAA4353
OZFWZ475_11_Leptodiaptomus_tyrrelli_sp_3_CHU_Canada_BOLDAAA4353
OZFWC140_11_Leptodiaptomus_tyrrelli_sp_3_CHU_Canada_BOLDAAA4353
OZFWC355_11_Leptodiaptomus_tyrrelli_sp_3_CHU_Canada_BOLDAAA4353
OZFWC374_11_Leptodiaptomus_tyrrelli_sp_3_CHU_Canada_BOLDAAA4353
OZFWC493_11_Leptodiaptomus_tyrrelli_sp_3_CHU_Canada_BOLDAAA4353
OZFWC565_12_Leptodiaptomus_tyrrelli_sp_3_CHU_Canada_BOLDAAA4353
OZFWC649_12_Leptodiaptomus_tyrrelli_sp_3_CHU_Canada_BOLDAAA4353
OZFWC650_12_Leptodiaptomus_tyrrelli_sp_3_CHU_Canada_BOLDAAA4353
OZFWC660_12_Leptodiaptomus_tyrrelli_sp_3_CHU_Canada_BOLDAAA4353
OZFWC748_12_Leptodiaptomus_tyrrelli_sp_3_CHU_Canada_BOLDAAA4353
OZFWC749_12_Leptodiaptomus_tyrrelli_sp_3_CHU_Canada_BOLDAAA4353
OZFWC752_12_Leptodiaptomus_tyrrelli_sp_3_CHU_Canada_BOLDAAA4353
OZFWC754_12_Leptodiaptomus_tyrrelli_sp_3_CHU_Canada_BOLDAAA4353
OZFWC782_12_Leptodiaptomus_tyrrelli_sp_3_CHU_Canada_BOLDAAA4353
OZFWC784_12_Leptodiaptomus_tyrrelli_sp_3_CHU_Canada_BOLDAAA4353
OZFWC787_12_Leptodiaptomus_tyrrelli_sp_3_CHU_Canada_BOLDAAA4353
OZFWC789_12_Leptodiaptomus_tyrrelli_sp_3_CHU_Canada_BOLDAAA4353
OZFWC796_12_Leptodiaptomus_tyrrelli_sp_3_CHU_Canada_BOLDAAA4353
OZFWC797_12_Leptodiaptomus_tyrrelli_sp_3_CHU_Canada_BOLDAAA4353
DSMAX351_06_Leptodiaptomus_tyrrelli_Canada_BOLDAAA4353
OZFWZ354_11_Leptodiaptomus_tyrrelli_sp_3_CHU_Canada_BOLDAAA4353
OZFWC388_11_Leptodiaptomus_tyrrelli_sp_3_CHU_Canada_BOLDAAA4353
DSMAX381_06_Leptodiaptomus_tyrrelli_Canada_BOLDAAA4353
DSMAX412_06_Leptodiaptomus_tyrrelli_Canada_BOLDAAA4353
SACOP059_08_Leptodiaptomus_tyrrelli_Canada_BOLDAAA4353
KLMPC003_09_Leptodiaptomus_tyrrelli_Canada_BOLDAAA4353
OZFWZ340_11_Leptodiaptomus_tyrrelli_sp_3_CHU_Canada_BOLDAAA4353
OZFWC297_11_Leptodiaptomus_tyrrelli_sp_3_CHU_Canada_BOLDAAA4353
OZFWC689_12_Leptodiaptomus_tyrrelli_sp_3_CHU_Canada_BOLDAAA4353
OZFWC310_11_Leptodiaptomus_tyrrelli_sp_3_CHU_Canada_BOLDAAA4353
OZFWC352_11_Leptodiaptomus_tyrrelli_sp_3_CHU_Canada_BOLDAAA4353
OZFWC356_11_Leptodiaptomus_tyrrelli_sp_3_CHU_Canada_BOLDAAA4353
OZFWC370_11_Leptodiaptomus_tyrrelli_sp_3_CHU_Canada_BOLDAAA4353
DSMAX420_06_Leptodiaptomus_tyrrelli_Canada_BOLDAAA4353
DSMAX333_06_Leptodiaptomus_tyrrelli_Canada_BOLDAAA4353
DSMAX345_06_Leptodiaptomus_tyrrelli_Canada_BOLDAAA4353
NJCGS081_09_Leptodiaptomus_tyrelli_Canada_BOLDAAA4353
OZFWC191_11_Leptodiaptomus_tyrrelli_sp_3_CHU_Canada_BOLDAAA4353
DSMAX405_06_Leptodiaptomus_tyrrelli_Canada_BOLDAAA4353
SGRZC034_09_Leptodiaptomus_tyrrelli_Canada_BOLDAAA4353
ELPPC035_09_Leptodiaptomus_tyrrelli_Canada_BOLDAAA4353
OZFWC363_11_Leptodiaptomus_tyrrelli_sp_3_CHU_Canada_BOLDAAA4353
DSMAX322_06_Leptodiaptomus_tyrrelli_Canada_BOLDAAA4353
DSMAX446_06_Leptodiaptomus_tyrrelli_Canada_BOLDAAA4353
DSMAX441_06_Leptodiaptomus_tyrrelli_Canada_BOLDAAA4353
OZFWC260_11_Leptodiaptomus_tyrrelli_sp_3_CHU_Canada_BOLDAAA4353
OZFWZ471_11_Leptodiaptomus_tyrrelli_sp_3_CHU_Canada_BOLDAAA4353
OZFWC261_11_Leptodiaptomus_tyrrelli_sp_3_CHU_Canada_BOLDAAA4353
ZPII374_07_Leptodiaptomus_tyrrelli_Canada_BOLDAAA4353
CRCN046_09_Leptodiaptomus_tyrrelli_Canada_BOLDAAA4353
CRCN047_09_Leptodiaptomus_tyrrelli_Canada_BOLDAAA4353
DSMAX453_06_Leptodiaptomus_tyrrelli_Canada_BOLDAAA4353
OZFWC220_11_Leptodiaptomus_tyrrelli_sp_3_CHU_Canada_BOLDAAA4353
DSMAX380_06_Leptodiaptomus_tyrrelli_Canada_BOLDAAA4354
DSMAX392_06_Leptodiaptomus_tyrrelli_Canada_BOLDAAA4355
OZFWZ363_11_Leptodiaptomus_tyrrelli_sp_2_CHU_Canada_BOLDAAY9555
96
96
96
96
96
96
96
96
96
96
CHMEP196_12_Leptodiaptomus_Canada_BOLDAAY9555
CHMEP197_12_Leptodiaptomus_Canada_BOLDAAY9555
CHMEP198_12_Leptodiaptomus_Canada_BOLDAAY9555
CHMEP199_12_Leptodiaptomus_Canada_BOLDAAY9555
CHMEP201_12_Leptodiaptomus_Canada_BOLDAAY9555
CHMEP200_12_Leptodiaptomus_Canada_BOLDAAY9555
DNARC015_19_Leptodiaptomus_Canada_BOLDAAY9555
DNARC066_19_Leptodiaptomus_Canada_BOLDAAY9555
DNARC191_19_Leptodiaptomus_Canada_BOLDAAY9555
DNARC192_19_Leptodiaptomus_Canada_BOLDAAY9555
DNARC193_19_Leptodiaptomus_Canada_BOLDAAY9555
DNARC203_19_Leptodiaptomus_Canada_BOLDAAY9555
DNARC215_19_Leptodiaptomus_Canada_BOLDAAY9555
DNARA703_21_Leptodiaptomus_Canada_BOLDAAY9555
DNARA704_21_Leptodiaptomus_Canada_BOLDAAY9555
DNARA707_21_Leptodiaptomus_Canada_BOLDAAY9555
DNARA926_21_Leptodiaptomus_Canada_BOLDAAY9555
DNARA1162_21_Leptodiaptomus_Canada_BOLDAAY9555
DNARA1167_21_Leptodiaptomus_Canada_BOLDAAY9555

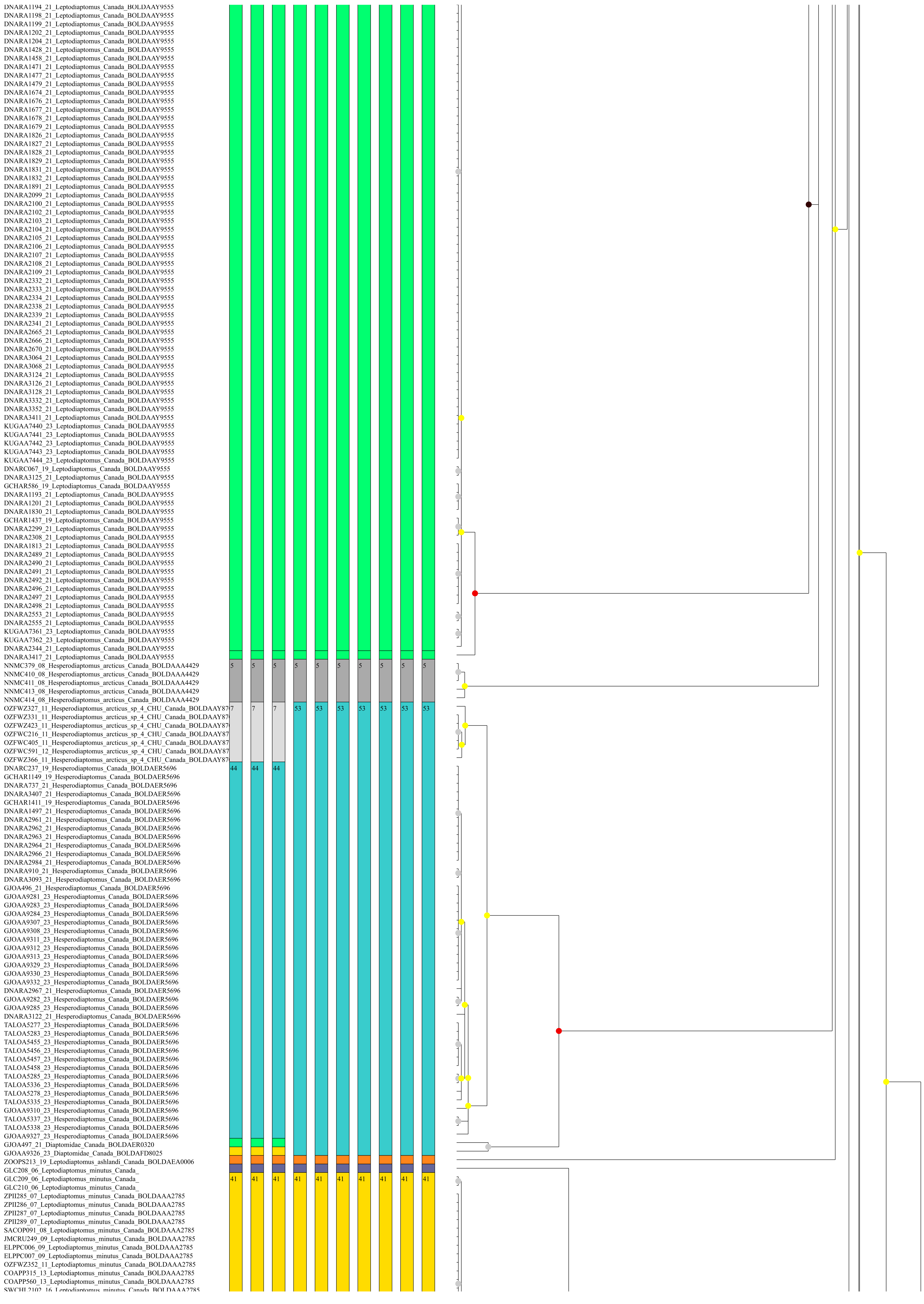
DNARA1194_21_Leptodiaptomus_Canada_BOLDAAY9555
DNARA1198_21_Leptodiaptomus_Canada_BOLDAAY9555
DNARA1199_21_Leptodiaptomus_Canada_BOLDAAY9555
DNARA1202_21_Leptodiaptomus_Canada_BOLDAAY9555
DNARA1204_21_Leptodiaptomus_Canada_BOLDAAY9555
DNARA1428_21_Leptodiaptomus_Canada_BOLDAAY9555
DNARA1458_21_Leptodiaptomus_Canada_BOLDAAY9555
DNARA1471_21_Leptodiaptomus_Canada_BOLDAAY9555
DNARA1477_21_Leptodiaptomus_Canada_BOLDAAY9555
DNARA1479_21_Leptodiaptomus_Canada_BOLDAAY9555
DNARA1674_21_Leptodiaptomus_Canada_BOLDAAY9555
DNARA1676_21_Leptodiaptomus_Canada_BOLDAAY9555
DNARA1677_21_Leptodiaptomus_Canada_BOLDAAY9555
DNARA1678_21_Leptodiaptomus_Canada_BOLDAAY9555
DNARA1679_21_Leptodiaptomus_Canada_BOLDAAY9555
DNARA1826_21_Leptodiaptomus_Canada_BOLDAAY9555
DNARA1827_21_Leptodiaptomus_Canada_BOLDAAY9555
DNARA1828_21_Leptodiaptomus_Canada_BOLDAAY9555
DNARA1829_21_Leptodiaptomus_Canada_BOLDAAY9555
DNARA1831_21_Leptodiaptomus_Canada_BOLDAAY9555
DNARA1832_21_Leptodiaptomus_Canada_BOLDAAY9555
DNARA1891_21_Leptodiaptomus_Canada_BOLDAAY9555
DNARA2099_21_Leptodiaptomus_Canada_BOLDAAY9555
DNARA2100_21_Leptodiaptomus_Canada_BOLDAAY9555
DNARA2102_21_Leptodiaptomus_Canada_BOLDAAY9555
DNARA2103_21_Leptodiaptomus_Canada_BOLDAAY9555
DNARA2104_21_Leptodiaptomus_Canada_BOLDAAY9555
DNARA2105_21_Leptodiaptomus_Canada_BOLDAAY9555
DNARA2106_21_Leptodiaptomus_Canada_BOLDAAY9555
DNARA2107_21_Leptodiaptomus_Canada_BOLDAAY9555
DNARA2108_21_Leptodiaptomus_Canada_BOLDAAY9555
DNARA2109_21_Leptodiaptomus_Canada_BOLDAAY9555
DNARA2332_21_Leptodiaptomus_Canada_BOLDAAY9555
DNARA2333_21_Leptodiaptomus_Canada_BOLDAAY9555
DNARA2334_21_Leptodiaptomus_Canada_BOLDAAY9555
DNARA2338_21_Leptodiaptomus_Canada_BOLDAAY9555
DNARA2339_21_Leptodiaptomus_Canada_BOLDAAY9555
DNARA2341_21_Leptodiaptomus_Canada_BOLDAAY9555
DNARA2665_21_Leptodiaptomus_Canada_BOLDAAY9555
DNARA2666_21_Leptodiaptomus_Canada_BOLDAAY9555
DNARA2670_21_Leptodiaptomus_Canada_BOLDAAY9555
DNARA3064_21_Leptodiaptomus_Canada_BOLDAAY9555
DNARA3068_21_Leptodiaptomus_Canada_BOLDAAY9555
DNARA3124_21_Leptodiaptomus_Canada_BOLDAAY9555
DNARA3126_21_Leptodiaptomus_Canada_BOLDAAY9555
DNARA3128_21_Leptodiaptomus_Canada_BOLDAAY9555
DNARA3332_21_Leptodiaptomus_Canada_BOLDAAY9555
DNARA3352_21_Leptodiaptomus_Canada_BOLDAAY9555
DNARA3411_21_Leptodiaptomus_Canada_BOLDAAY9555
KUGAA7440_23_Leptodiaptomus_Canada_BOLDAAY9555
KUGAA7441_23_Leptodiaptomus_Canada_BOLDAAY9555
KUGAA7442_23_Leptodiaptomus_Canada_BOLDAAY9555
KUGAA7443_23_Leptodiaptomus_Canada_BOLDAAY9555
KUGAA7444_23_Leptodiaptomus_Canada_BOLDAAY9555
DNARC067_19_Leptodiaptomus_Canada_BOLDAAY9555
DNARA3125_21_Leptodiaptomus_Canada_BOLDAAY9555
GCHAR586_19_Leptodiaptomus_Canada_BOLDAAY9555
DNARA1193_21_Leptodiaptomus_Canada_BOLDAAY9555
DNARA1201_21_Leptodiaptomus_Canada_BOLDAAY9555
DNARA1830_21_Leptodiaptomus_Canada_BOLDAAY9555
GCHAR1437_19_Leptodiaptomus_Canada_BOLDAAY9555
DNARA2299_21_Leptodiaptomus_Canada_BOLDAAY9555
DNARA2308_21_Leptodiaptomus_Canada_BOLDAAY9555
DNARA1813_21_Leptodiaptomus_Canada_BOLDAAY9555
DNARA2489_21_Leptodiaptomus_Canada_BOLDAAY9555
DNARA2490_21_Leptodiaptomus_Canada_BOLDAAY9555
DNARA2491_21_Leptodiaptomus_Canada_BOLDAAY9555
DNARA2492_21_Leptodiaptomus_Canada_BOLDAAY9555
DNARA2496_21_Leptodiaptomus_Canada_BOLDAAY9555
DNARA2497_21_Leptodiaptomus_Canada_BOLDAAY9555
DNARA2498_21_Leptodiaptomus_Canada_BOLDAAY9555
DNARA2553_21_Leptodiaptomus_Canada_BOLDAAY9555
DNARA2555_21_Leptodiaptomus_Canada_BOLDAAY9555
KUGAA7361_23_Leptodiaptomus_Canada_BOLDAAY9555
KUGAA7362_23_Leptodiaptomus_Canada_BOLDAAY9555
DNARA2344_21_Leptodiaptomus_Canada_BOLDAAY9555
DNARA3417_21_Leptodiaptomus_Canada_BOLDAAY9555
NNMC379_08_Hesperodiaptomus_arcticus_Canada_BOLDAAA4429
NNMC410_08_Hesperodiaptomus_arcticus_Canada_BOLDAAA4429
NNMC411_08_Hesperodiaptomus_arcticus_Canada_BOLDAAA4429
NNMC413_08_Hesperodiaptomus_arcticus_Canada_BOLDAAA4429
NNMC414_08_Hesperodiaptomus_arcticus_Canada_BOLDAAA4429
OZFWZ327_11_Hesperodiaptomus_arcticus_sp_4_CHU_Canada_BOLDAAY87
OZFWZ331_11_Hesperodiaptomus_arcticus_sp_4_CHU_Canada_BOLDAAY87
OZFWZ423_11_Hesperodiaptomus_arcticus_sp_4_CHU_Canada_BOLDAAY87
OZFWC216_11_Hesperodiaptomus_arcticus_sp_4_CHU_Canada_BOLDAAY87
OZFWC405_11_Hesperodiaptomus_arcticus_sp_4_CHU_Canada_BOLDAAY87
OZFWC591_12_Hesperodiaptomus_arcticus_sp_4_CHU_Canada_BOLDAAY87
OZFWZ366_11_Hesperodiaptomus_arcticus_sp_4_CHU_Canada_BOLDAAY87
DNARC237_19_Hesperodiaptomus_Canada_BOLDAER5696
GCHAR1149_19_Hesperodiaptomus_Canada_BOLDAER5696
DNARA737_21_Hesperodiaptomus_Canada_BOLDAER5696
DNARA3407_21_Hesperodiaptomus_Canada_BOLDAER5696
GCHAR1411_19_Hesperodiaptomus_Canada_BOLDAER5696
DNARA1497_21_Hesperodiaptomus_Canada_BOLDAER5696
DNARA2961_21_Hesperodiaptomus_Canada_BOLDAER5696
DNARA2962_21_Hesperodiaptomus_Canada_BOLDAER5696
DNARA2963_21_Hesperodiaptomus_Canada_BOLDAER5696
DNARA2964_21_Hesperodiaptomus_Canada_BOLDAER5696
DNARA2966_21_Hesperodiaptomus_Canada_BOLDAER5696
DNARA2984_21_Hesperodiaptomus_Canada_BOLDAER5696
DNARA910_21_Hesperodiaptomus_Canada_BOLDAER5696
DNARA3093_21_Hesperodiaptomus_Canada_BOLDAER5696
GJOA496_21_Hesperodiaptomus_Canada_BOLDAER5696
GJOAA9281_23_Hesperodiaptomus_Canada_BOLDAER5696
GJOAA9283_23_Hesperodiaptomus_Canada_BOLDAER5696
GJOAA9284_23_Hesperodiaptomus_Canada_BOLDAER5696
GJOAA9307_23_Hesperodiaptomus_Canada_BOLDAER5696
GJOAA9308_23_Hesperodiaptomus_Canada_BOLDAER5696
GJOAA9311_23_Hesperodiaptomus_Canada_BOLDAER5696
GJOAA9312_23_Hesperodiaptomus_Canada_BOLDAER5696
GJOAA9313_23_Hesperodiaptomus_Canada_BOLDAER5696
GJOAA9329_23_Hesperodiaptomus_Canada_BOLDAER5696
GJOAA9330_23_Hesperodiaptomus_Canada_BOLDAER5696
GJOAA9332_23_Hesperodiaptomus_Canada_BOLDAER5696
DNARA2967_21_Hesperodiaptomus_Canada_BOLDAER5696
GJOAA9282_23_Hesperodiaptomus_Canada_BOLDAER5696
GJOAA9285_23_Hesperodiaptomus_Canada_BOLDAER5696
DNARA3122_21_Hesperodiaptomus_Canada_BOLDAER5696
TALOA5277_23_Hesperodiaptomus_Canada_BOLDAER5696
TALOA5283_23_Hesperodiaptomus_Canada_BOLDAER5696
TALOA5455_23_Hesperodiaptomus_Canada_BOLDAER5696
TALOA5456_23_Hesperodiaptomus_Canada_BOLDAER5696
TALOA5457_23_Hesperodiaptomus_Canada_BOLDAER5696
TALOA5458_23_Hesperodiaptomus_Canada_BOLDAER5696
TALOA5285_23_Hesperodiaptomus_Canada_BOLDAER5696
TALOA5336_23_Hesperodiaptomus_Canada_BOLDAER5696
TALOA5278_23_Hesperodiaptomus_Canada_BOLDAER5696
TALOA5335_23_Hesperodiaptomus_Canada_BOLDAER5696
GJOAA9310_23_Hesperodiaptomus_Canada_BOLDAER5696
TALOA5337_23_Hesperodiaptomus_Canada_BOLDAER5696
TALOA5338_23_Hesperodiaptomus_Canada_BOLDAER5696
GJOAA9327_23_Hesperodiaptomus_Canada_BOLDAER5696
GJOA497_21_Diaptomidae_Canada_BOLDAER0320
GJOAA9326_23_Diaptomidae_Canada_BOLDAFD8025
ZOOPS213_19_Leptodiaptomus_ashlandi_Canada_BOLDAEA0006
GLC208_06_Leptodiaptomus_minutus_Canada_
GLC209_06_Leptodiaptomus_minutus_Canada_
GLC210_06_Leptodiaptomus_minutus_Canada_
ZPII285_07_Leptodiaptomus_minutus_Canada_BOLDAAA2785
ZPII286_07_Leptodiaptomus_minutus_Canada_BOLDAAA2785
ZPII287_07_Leptodiaptomus_minutus_Canada_BOLDAAA2785
ZPII289_07_Leptodiaptomus_minutus_Canada_BOLDAAA2785
SACOP091_08_Leptodiaptomus_minutus_Canada_BOLDAAA2785
JMCRU249_09_Leptodiaptomus_minutus_Canada_BOLDAAA2785
ELPPC006_09_Leptodiaptomus_minutus_Canada_BOLDAAA2785
ELPPC007_09_Leptodiaptomus_minutus_Canada_BOLDAAA2785
OZFWZ352_11_Leptodiaptomus_minutus_Canada_BOLDAAA2785
COAPP315_13_Leptodiaptomus_minutus_Canada_BOLDAAA2785
COAPP560_13_Leptodiaptomus_minutus_Canada_BOLDAAA2785
SWCHL2102_16_Leptodiaptomus_minutus_Canada_BOLDAAA2785
5
7
53
44
41

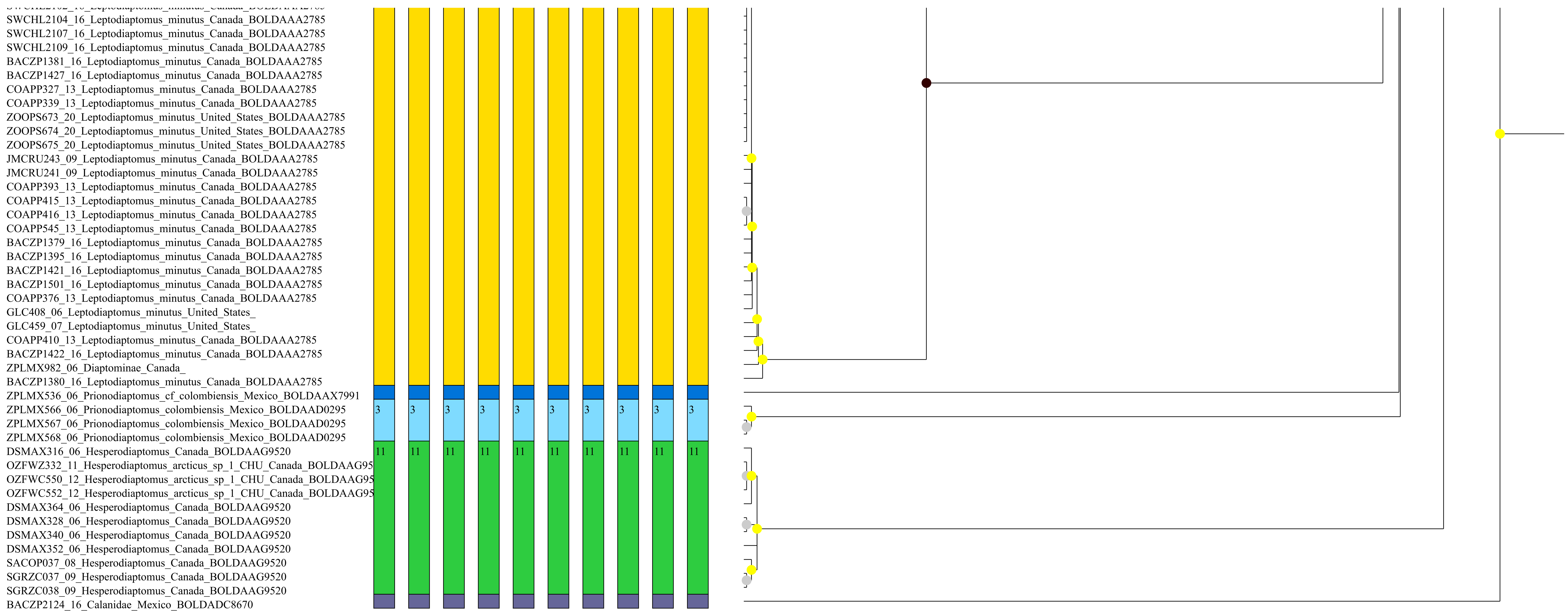
SWCHL2104_16_Leptodiaptomus_minutus_Canada_BOLDAAA2785
SWCHL2107_16_Leptodiaptomus_minutus_Canada_BOLDAAA2785
SWCHL2109_16_Leptodiaptomus_minutus_Canada_BOLDAAA2785
BACZP1381_16_Leptodiaptomus_minutus_Canada_BOLDAAA2785
BACZP1427_16_Leptodiaptomus_minutus_Canada_BOLDAAA2785
COAPP327_13_Leptodiaptomus_minutus_Canada_BOLDAAA2785
COAPP339_13_Leptodiaptomus_minutus_Canada_BOLDAAA2785
ZOOPS673_20_Leptodiaptomus_minutus_United_States_BOLDAAA2785
ZOOPS674_20_Leptodiaptomus_minutus_United_States_BOLDAAA2785
ZOOPS675_20_Leptodiaptomus_minutus_United_States_BOLDAAA2785
JMCRU243_09_Leptodiaptomus_minutus_Canada_BOLDAAA2785
JMCRU241_09_Leptodiaptomus_minutus_Canada_BOLDAAA2785
COAPP393_13_Leptodiaptomus_minutus_Canada_BOLDAAA2785
COAPP415_13_Leptodiaptomus_minutus_Canada_BOLDAAA2785
COAPP416_13_Leptodiaptomus_minutus_Canada_BOLDAAA2785
COAPP545_13_Leptodiaptomus_minutus_Canada_BOLDAAA2785
BACZP1379_16_Leptodiaptomus_minutus_Canada_BOLDAAA2785
BACZP1395_16_Leptodiaptomus_minutus_Canada_BOLDAAA2785
BACZP1421_16_Leptodiaptomus_minutus_Canada_BOLDAAA2785
BACZP1501_16_Leptodiaptomus_minutus_Canada_BOLDAAA2785
COAPP376_13_Leptodiaptomus_minutus_Canada_BOLDAAA2785
GLC408_06_Leptodiaptomus_minutus_United_States_
GLC459_07_Leptodiaptomus_minutus_United_States_
COAPP410_13_Leptodiaptomus_minutus_Canada_BOLDAAA2785
BACZP1422_16_Leptodiaptomus_minutus_Canada_BOLDAAA2785
ZPLMX982_06_Diaptominae_Canada_
BACZP1380_16_Leptodiaptomus_minutus_Canada_BOLDAAA2785
ZPLMX536_06_Prionodiaptomus_cf_colombiensis_Mexico_BOLDAAX7991
ZPLMX566_06_Prionodiaptomus_colombiensis_Mexico_BOLDAAD0295
ZPLMX567_06_Prionodiaptomus_colombiensis_Mexico_BOLDAAD0295
ZPLMX568_06_Prionodiaptomus_colombiensis_Mexico_BOLDAAD0295
DSMAX316_06_Hesperodiaptomus_Canada_BOLDAAG9520
OZFWZ332_11_Hesperodiaptomus_arcticus_sp_1_CHU_Canada_BOLDAAG95
OZFWC550_12_Hesperodiaptomus_arcticus_sp_1_CHU_Canada_BOLDAAG95
OZFWC552_12_Hesperodiaptomus_arcticus_sp_1_CHU_Canada_BOLDAAG95
DSMAX364_06_Hesperodiaptomus_Canada_BOLDAAG9520
DSMAX328_06_Hesperodiaptomus_Canada_BOLDAAG9520
DSMAX340_06_Hesperodiaptomus_Canada_BOLDAAG9520
DSMAX352_06_Hesperodiaptomus_Canada_BOLDAAG9520
SACOP037_08_Hesperodiaptomus_Canada_BOLDAAG9520
SGRZC037_09_Hesperodiaptomus_Canada_BOLDAAG9520
SGRZC038_09_Hesperodiaptomus_Canada_BOLDAAG9520
BACZP2124_16_Calanidae_Mexico_BOLDADC8670
3
11
