# Supplementary material for: DNA barcoding of North American freshwater copepods (Diaptomidae and Cyclopoida): an overview after 20 years with emphasis in the Mexican fauna, the transition between the Nearctic and Neotropics
Source: PeerJ. 2026 Apr 9;14:e20989. doi: 10.7717/peerj.20989 (PMC13070316; doi:10.7717/peerj.20989)
Supplement: Supplemental Information 4 — The colored bars indicate each subset. The lowest score is represented in the Rank 1 at the top of the bars, being the sixth from right to left. The proposed ID tree is on the right side of the bars. Each terminal branch includes the same data as Supplementary Figure 2 [file peerj-14-20989-s004.pdf]

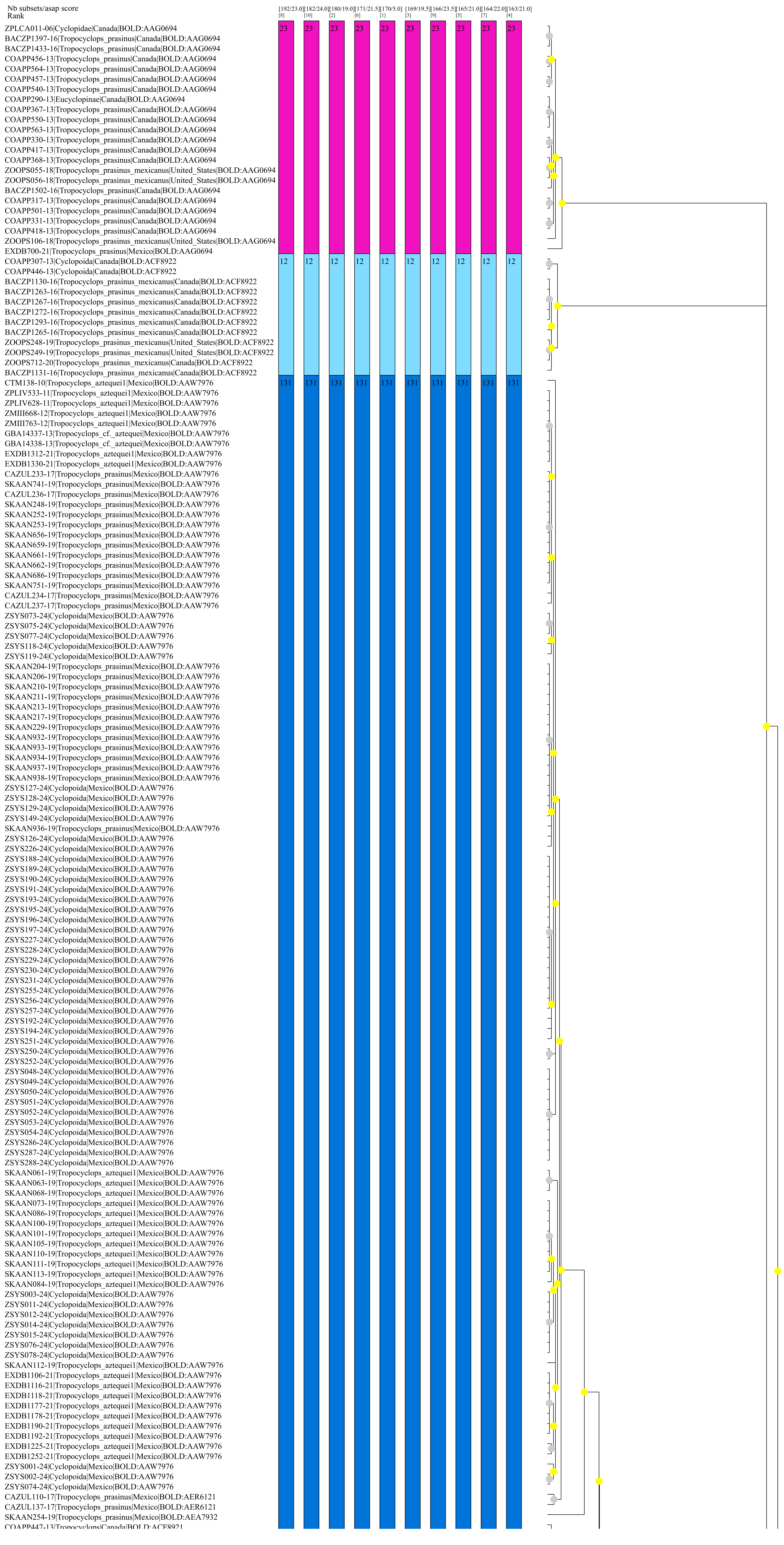

Nb subsets/asap score
Rank
[192/23.0][182/24.0][180/19.0][171/21.5][170/5.0] [169/19.5][166/23.5][165/21.0][164/22.0][163/21.0]
[8] [10] [2] [6] [1] [3] [9] [5] [7] [4]
23 23 23 23 23 23 23 23 23 23
12 12 12 12 12 12 12 12 12 12
131 131 131 131 131 131 131 131 131 131
ZPLCA011-06|Cyclopidae|Canada|BOLD:AAG0694
BACZP1397-16|Tropocyclops_prasinus|Canada|BOLD:AAG0694
BACZP1433-16|Tropocyclops_prasinus|Canada|BOLD:AAG0694
COAPP456-13|Tropocyclops_prasinus|Canada|BOLD:AAG0694
COAPP564-13|Tropocyclops_prasinus|Canada|BOLD:AAG0694
COAPP457-13|Tropocyclops_prasinus|Canada|BOLD:AAG0694
COAPP540-13|Tropocyclops_prasinus|Canada|BOLD:AAG0694
COAPP290-13|Eucyclopinae|Canada|BOLD:AAG0694
COAPP367-13|Tropocyclops_prasinus|Canada|BOLD:AAG0694
COAPP550-13|Tropocyclops_prasinus|Canada|BOLD:AAG0694
COAPP563-13|Tropocyclops_prasinus|Canada|BOLD:AAG0694
COAPP330-13|Tropocyclops_prasinus|Canada|BOLD:AAG0694
COAPP417-13|Tropocyclops_prasinus|Canada|BOLD:AAG0694
COAPP368-13|Tropocyclops_prasinus|Canada|BOLD:AAG0694
ZOOPS055-18|Tropocyclops_prasinus_mexicanus|United_States|BOLD:AAG0694
ZOOPS056-18|Tropocyclops_prasinus_mexicanus|United_States|BOLD:AAG0694
BACZP1502-16|Tropocyclops_prasinus|Canada|BOLD:AAG0694
COAPP317-13|Tropocyclops_prasinus|Canada|BOLD:AAG0694
COAPP501-13|Tropocyclops_prasinus|Canada|BOLD:AAG0694
COAPP331-13|Tropocyclops_prasinus|Canada|BOLD:AAG0694
COAPP418-13|Tropocyclops_prasinus|Canada|BOLD:AAG0694
ZOOPS106-18|Tropocyclops_prasinus_mexicanus|United_States|BOLD:AAG0694
EXDB700-21|Tropocyclops_prasinus|Mexico|BOLD:AAG0694
COAPP307-13|Cyclopoida|Canada|BOLD:ACF8922
COAPP446-13|Cyclopoida|Canada|BOLD:ACF8922
BACZP1130-16|Tropocyclops_prasinus_mexicanus|Canada|BOLD:ACF8922
BACZP1263-16|Tropocyclops_prasinus_mexicanus|Canada|BOLD:ACF8922
BACZP1267-16|Tropocyclops_prasinus_mexicanus|Canada|BOLD:ACF8922
BACZP1272-16|Tropocyclops_prasinus_mexicanus|Canada|BOLD:ACF8922
BACZP1293-16|Tropocyclops_prasinus_mexicanus|Canada|BOLD:ACF8922
BACZP1265-16|Tropocyclops_prasinus_mexicanus|Canada|BOLD:ACF8922
ZOOPS248-19|Tropocyclops_prasinus_mexicanus|United_States|BOLD:ACF8922
ZOOPS249-19|Tropocyclops_prasinus_mexicanus|United_States|BOLD:ACF8922
ZOOPS712-20|Tropocyclops_prasinus_mexicanus|Canada|BOLD:ACF8922
BACZP1131-16|Tropocyclops_prasinus_mexicanus|Canada|BOLD:ACF8922
CTM138-10|Tropocyclops_aztequei1|Mexico|BOLD:AAW7976
ZPLIV533-11|Tropocyclops_aztequei1|Mexico|BOLD:AAW7976
ZPLIV628-11|Tropocyclops_aztequei1|Mexico|BOLD:AAW7976
ZMIII668-12|Tropocyclops_aztequei1|Mexico|BOLD:AAW7976
ZMIII763-12|Tropocyclops_aztequei1|Mexico|BOLD:AAW7976
GBA14337-13|Tropocyclops_cf._aztequei|Mexico|BOLD:AAW7976
GBA14338-13|Tropocyclops_cf._aztequei|Mexico|BOLD:AAW7976
EXDB1312-21|Tropocyclops_aztequei1|Mexico|BOLD:AAW7976
EXDB1330-21|Tropocyclops_aztequei1|Mexico|BOLD:AAW7976
CAZUL233-17|Tropocyclops_prasinus|Mexico|BOLD:AAW7976
SKAAN741-19|Tropocyclops_prasinus|Mexico|BOLD:AAW7976
CAZUL236-17|Tropocyclops_prasinus|Mexico|BOLD:AAW7976
SKAAN248-19|Tropocyclops_prasinus|Mexico|BOLD:AAW7976
SKAAN252-19|Tropocyclops_prasinus|Mexico|BOLD:AAW7976
SKAAN253-19|Tropocyclops_prasinus|Mexico|BOLD:AAW7976
SKAAN656-19|Tropocyclops_prasinus|Mexico|BOLD:AAW7976
SKAAN659-19|Tropocyclops_prasinus|Mexico|BOLD:AAW7976
SKAAN661-19|Tropocyclops_prasinus|Mexico|BOLD:AAW7976
SKAAN662-19|Tropocyclops_prasinus|Mexico|BOLD:AAW7976
SKAAN686-19|Tropocyclops_prasinus|Mexico|BOLD:AAW7976
SKAAN751-19|Tropocyclops_prasinus|Mexico|BOLD:AAW7976
CAZUL234-17|Tropocyclops_prasinus|Mexico|BOLD:AAW7976
CAZUL237-17|Tropocyclops_prasinus|Mexico|BOLD:AAW7976
ZSYS073-24|Cyclopoida|Mexico|BOLD:AAW7976
ZSYS075-24|Cyclopoida|Mexico|BOLD:AAW7976
ZSYS077-24|Cyclopoida|Mexico|BOLD:AAW7976
ZSYS118-24|Cyclopoida|Mexico|BOLD:AAW7976
ZSYS119-24|Cyclopoida|Mexico|BOLD:AAW7976
SKAAN204-19|Tropocyclops_prasinus|Mexico|BOLD:AAW7976
SKAAN206-19|Tropocyclops_prasinus|Mexico|BOLD:AAW7976
SKAAN210-19|Tropocyclops_prasinus|Mexico|BOLD:AAW7976
SKAAN211-19|Tropocyclops_prasinus|Mexico|BOLD:AAW7976
SKAAN213-19|Tropocyclops_prasinus|Mexico|BOLD:AAW7976
SKAAN217-19|Tropocyclops_prasinus|Mexico|BOLD:AAW7976
SKAAN229-19|Tropocyclops_prasinus|Mexico|BOLD:AAW7976
SKAAN932-19|Tropocyclops_prasinus|Mexico|BOLD:AAW7976
SKAAN933-19|Tropocyclops_prasinus|Mexico|BOLD:AAW7976
SKAAN934-19|Tropocyclops_prasinus|Mexico|BOLD:AAW7976
SKAAN937-19|Tropocyclops_prasinus|Mexico|BOLD:AAW7976
SKAAN938-19|Tropocyclops_prasinus|Mexico|BOLD:AAW7976
ZSYS127-24|Cyclopoida|Mexico|BOLD:AAW7976
ZSYS128-24|Cyclopoida|Mexico|BOLD:AAW7976
ZSYS129-24|Cyclopoida|Mexico|BOLD:AAW7976
ZSYS149-24|Cyclopoida|Mexico|BOLD:AAW7976
SKAAN936-19|Tropocyclops_prasinus|Mexico|BOLD:AAW7976
ZSYS126-24|Cyclopoida|Mexico|BOLD:AAW7976
ZSYS226-24|Cyclopoida|Mexico|BOLD:AAW7976
ZSYS188-24|Cyclopoida|Mexico|BOLD:AAW7976
ZSYS189-24|Cyclopoida|Mexico|BOLD:AAW7976
ZSYS190-24|Cyclopoida|Mexico|BOLD:AAW7976
ZSYS191-24|Cyclopoida|Mexico|BOLD:AAW7976
ZSYS193-24|Cyclopoida|Mexico|BOLD:AAW7976
ZSYS195-24|Cyclopoida|Mexico|BOLD:AAW7976
ZSYS196-24|Cyclopoida|Mexico|BOLD:AAW7976
ZSYS197-24|Cyclopoida|Mexico|BOLD:AAW7976
ZSYS227-24|Cyclopoida|Mexico|BOLD:AAW7976
ZSYS228-24|Cyclopoida|Mexico|BOLD:AAW7976
ZSYS229-24|Cyclopoida|Mexico|BOLD:AAW7976
ZSYS230-24|Cyclopoida|Mexico|BOLD:AAW7976
ZSYS231-24|Cyclopoida|Mexico|BOLD:AAW7976
ZSYS255-24|Cyclopoida|Mexico|BOLD:AAW7976
ZSYS256-24|Cyclopoida|Mexico|BOLD:AAW7976
ZSYS257-24|Cyclopoida|Mexico|BOLD:AAW7976
ZSYS192-24|Cyclopoida|Mexico|BOLD:AAW7976
ZSYS194-24|Cyclopoida|Mexico|BOLD:AAW7976
ZSYS251-24|Cyclopoida|Mexico|BOLD:AAW7976
ZSYS250-24|Cyclopoida|Mexico|BOLD:AAW7976
ZSYS252-24|Cyclopoida|Mexico|BOLD:AAW7976
ZSYS048-24|Cyclopoida|Mexico|BOLD:AAW7976
ZSYS049-24|Cyclopoida|Mexico|BOLD:AAW7976
ZSYS050-24|Cyclopoida|Mexico|BOLD:AAW7976
ZSYS051-24|Cyclopoida|Mexico|BOLD:AAW7976
ZSYS052-24|Cyclopoida|Mexico|BOLD:AAW7976
ZSYS053-24|Cyclopoida|Mexico|BOLD:AAW7976
ZSYS054-24|Cyclopoida|Mexico|BOLD:AAW7976
ZSYS286-24|Cyclopoida|Mexico|BOLD:AAW7976
ZSYS287-24|Cyclopoida|Mexico|BOLD:AAW7976
ZSYS288-24|Cyclopoida|Mexico|BOLD:AAW7976
SKAAN061-19|Tropocyclops_aztequei1|Mexico|BOLD:AAW7976
SKAAN063-19|Tropocyclops_aztequei1|Mexico|BOLD:AAW7976
SKAAN068-19|Tropocyclops_aztequei1|Mexico|BOLD:AAW7976
SKAAN073-19|Tropocyclops_aztequei1|Mexico|BOLD:AAW7976
SKAAN086-19|Tropocyclops_aztequei1|Mexico|BOLD:AAW7976
SKAAN100-19|Tropocyclops_aztequei1|Mexico|BOLD:AAW7976
SKAAN101-19|Tropocyclops_aztequei1|Mexico|BOLD:AAW7976
SKAAN105-19|Tropocyclops_aztequei1|Mexico|BOLD:AAW7976
SKAAN110-19|Tropocyclops_aztequei1|Mexico|BOLD:AAW7976
SKAAN111-19|Tropocyclops_aztequei1|Mexico|BOLD:AAW7976
SKAAN113-19|Tropocyclops_aztequei1|Mexico|BOLD:AAW7976
SKAAN084-19|Tropocyclops_aztequei1|Mexico|BOLD:AAW7976
ZSYS003-24|Cyclopoida|Mexico|BOLD:AAW7976
ZSYS011-24|Cyclopoida|Mexico|BOLD:AAW7976
ZSYS012-24|Cyclopoida|Mexico|BOLD:AAW7976
ZSYS014-24|Cyclopoida|Mexico|BOLD:AAW7976
ZSYS015-24|Cyclopoida|Mexico|BOLD:AAW7976
ZSYS076-24|Cyclopoida|Mexico|BOLD:AAW7976
ZSYS078-24|Cyclopoida|Mexico|BOLD:AAW7976
SKAAN112-19|Tropocyclops_aztequei1|Mexico|BOLD:AAW7976
EXDB1106-21|Tropocyclops_aztequei1|Mexico|BOLD:AAW7976
EXDB1116-21|Tropocyclops_aztequei1|Mexico|BOLD:AAW7976
EXDB1118-21|Tropocyclops_aztequei1|Mexico|BOLD:AAW7976
EXDB1177-21|Tropocyclops_aztequei1|Mexico|BOLD:AAW7976
EXDB1178-21|Tropocyclops_aztequei1|Mexico|BOLD:AAW7976
EXDB1190-21|Tropocyclops_aztequei1|Mexico|BOLD:AAW7976
EXDB1192-21|Tropocyclops_aztequei1|Mexico|BOLD:AAW7976
EXDB1225-21|Tropocyclops_aztequei1|Mexico|BOLD:AAW7976
EXDB1252-21|Tropocyclops_aztequei1|Mexico|BOLD:AAW7976
ZSYS001-24|Cyclopoida|Mexico|BOLD:AAW7976
ZSYS002-24|Cyclopoida|Mexico|BOLD:AAW7976
ZSYS074-24|Cyclopoida|Mexico|BOLD:AAW7976
CAZUL110-17|Tropocyclops_prasinus|Mexico|BOLD:AER6121
CAZUL137-17|Tropocyclops_prasinus|Mexico|BOLD:AER6121
SKAAN254-19|Tropocyclops_prasinus|Mexico|BOLD:AEA7932

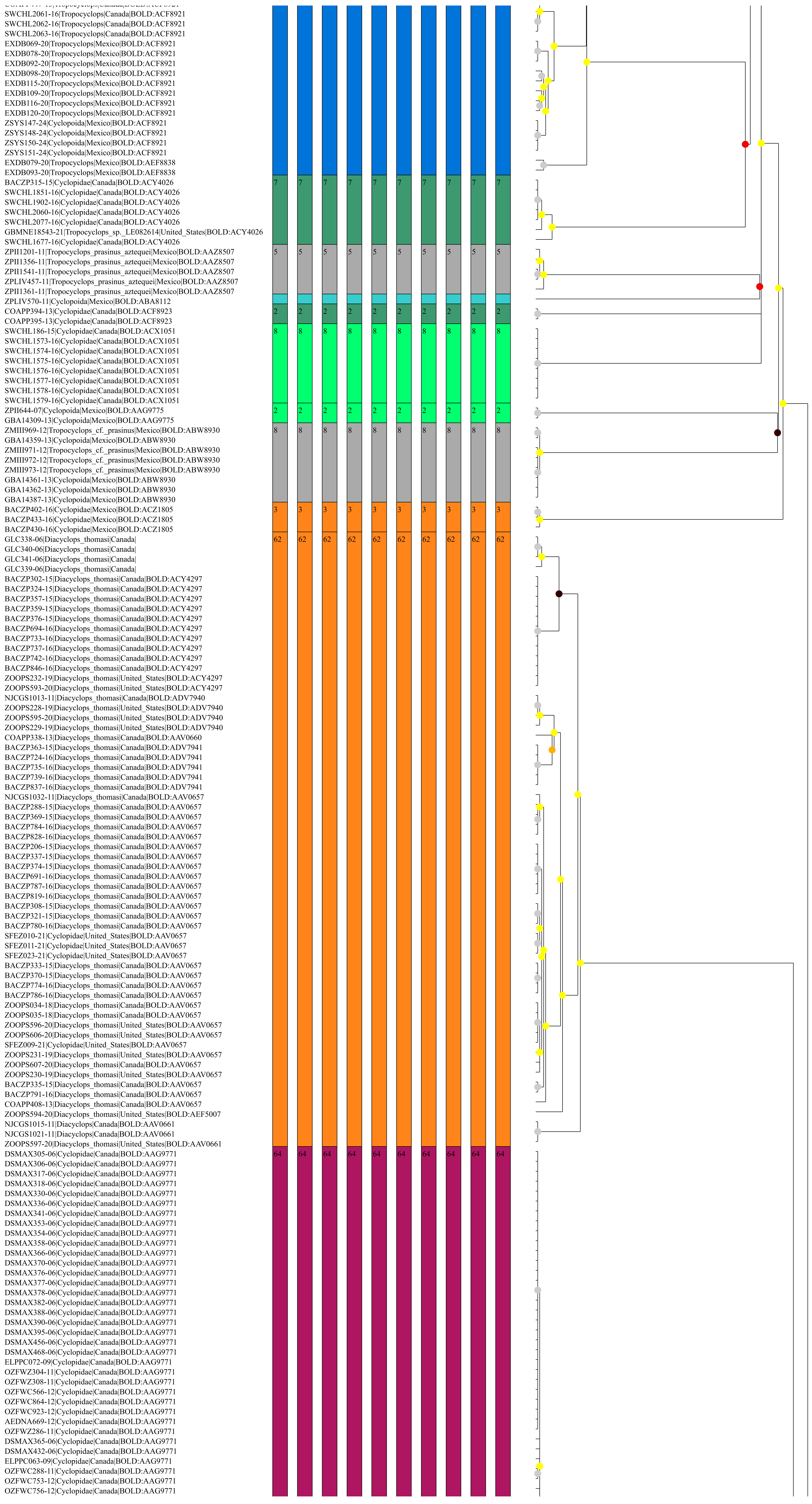

SWCHL2061-16|Tropocyclops|Canada|BOLD:ACF8921
SWCHL2062-16|Tropocyclops|Canada|BOLD:ACF8921
SWCHL2063-16|Tropocyclops|Canada|BOLD:ACF8921
EXDB069-20|Tropocyclops|Mexico|BOLD:ACF8921
EXDB078-20|Tropocyclops|Mexico|BOLD:ACF8921
EXDB092-20|Tropocyclops|Mexico|BOLD:ACF8921
EXDB098-20|Tropocyclops|Mexico|BOLD:ACF8921
EXDB115-20|Tropocyclops|Mexico|BOLD:ACF8921
EXDB109-20|Tropocyclops|Mexico|BOLD:ACF8921
EXDB116-20|Tropocyclops|Mexico|BOLD:ACF8921
EXDB120-20|Tropocyclops|Mexico|BOLD:ACF8921
ZSYS147-24|Cyclopoida|Mexico|BOLD:ACF8921
ZSYS148-24|Cyclopoida|Mexico|BOLD:ACF8921
ZSYS150-24|Cyclopoida|Mexico|BOLD:ACF8921
ZSYS151-24|Cyclopoida|Mexico|BOLD:ACF8921
EXDB079-20|Tropocyclops|Mexico|BOLD:AEF8838
EXDB093-20|Tropocyclops|Mexico|BOLD:AEF8838
BACZP315-15|Cyclopidae|Canada|BOLD:ACY4026
SWCHL1851-16|Cyclopidae|Canada|BOLD:ACY4026
SWCHL1902-16|Cyclopidae|Canada|BOLD:ACY4026
SWCHL2060-16|Cyclopidae|Canada|BOLD:ACY4026
SWCHL2077-16|Cyclopidae|Canada|BOLD:ACY4026
GBMNE18543-21|Tropocyclops_sp._LE082614|United_States|BOLD:ACY4026
SWCHL1677-16|Cyclopidae|Canada|BOLD:ACY4026
ZPII1201-11|Tropocyclops_prasinus_aztequei|Mexico|BOLD:AAZ8507
ZPII1356-11|Tropocyclops_prasinus_aztequei|Mexico|BOLD:AAZ8507
ZPII1541-11|Tropocyclops_prasinus_aztequei|Mexico|BOLD:AAZ8507
ZPLIV457-11|Tropocyclops_prasinus_aztequei|Mexico|BOLD:AAZ8507
ZPII1361-11|Tropocyclops_prasinus_aztequei|Mexico|BOLD:AAZ8507
ZPLIV570-11|Cyclopoida|Mexico|BOLD:ABA8112
COAPP394-13|Cyclopidae|Canada|BOLD:ACF8923
COAPP395-13|Cyclopidae|Canada|BOLD:ACF8923
SWCHL186-15|Cyclopidae|Canada|BOLD:ACX1051
SWCHL1573-16|Cyclopidae|Canada|BOLD:ACX1051
SWCHL1574-16|Cyclopidae|Canada|BOLD:ACX1051
SWCHL1575-16|Cyclopidae|Canada|BOLD:ACX1051
SWCHL1576-16|Cyclopidae|Canada|BOLD:ACX1051
SWCHL1577-16|Cyclopidae|Canada|BOLD:ACX1051
SWCHL1578-16|Cyclopidae|Canada|BOLD:ACX1051
SWCHL1579-16|Cyclopidae|Canada|BOLD:ACX1051
ZPII644-07|Cyclopoida|Mexico|BOLD:AAG9775
GBA14309-13|Cyclopoida|Mexico|BOLD:AAG9775
ZMIII969-12|Tropocyclops_cf._prasinus|Mexico|BOLD:ABW8930
GBA14359-13|Cyclopoida|Mexico|BOLD:ABW8930
ZMIII971-12|Tropocyclops_cf._prasinus|Mexico|BOLD:ABW8930
ZMIII972-12|Tropocyclops_cf._prasinus|Mexico|BOLD:ABW8930
ZMIII973-12|Tropocyclops_cf._prasinus|Mexico|BOLD:ABW8930
GBA14361-13|Cyclopoida|Mexico|BOLD:ABW8930
GBA14362-13|Cyclopoida|Mexico|BOLD:ABW8930
GBA14387-13|Cyclopoida|Mexico|BOLD:ABW8930
BACZP402-16|Cyclopidae|Mexico|BOLD:ACZ1805
BACZP433-16|Cyclopidae|Mexico|BOLD:ACZ1805
BACZP430-16|Cyclopidae|Mexico|BOLD:ACZ1805
GLC338-06|Diacyclops_thomasi|Canada|
GLC340-06|Diacyclops_thomasi|Canada|
GLC341-06|Diacyclops_thomasi|Canada|
GLC339-06|Diacyclops_thomasi|Canada|
BACZP302-15|Diacyclops_thomasi|Canada|BOLD:ACY4297
BACZP324-15|Diacyclops_thomasi|Canada|BOLD:ACY4297
BACZP357-15|Diacyclops_thomasi|Canada|BOLD:ACY4297
BACZP359-15|Diacyclops_thomasi|Canada|BOLD:ACY4297
BACZP376-15|Diacyclops_thomasi|Canada|BOLD:ACY4297
BACZP694-16|Diacyclops_thomasi|Canada|BOLD:ACY4297
BACZP733-16|Diacyclops_thomasi|Canada|BOLD:ACY4297
BACZP737-16|Diacyclops_thomasi|Canada|BOLD:ACY4297
BACZP742-16|Diacyclops_thomasi|Canada|BOLD:ACY4297
BACZP846-16|Diacyclops_thomasi|Canada|BOLD:ACY4297
ZOOPS232-19|Diacyclops_thomasi|United_States|BOLD:ACY4297
ZOOPS593-20|Diacyclops_thomasi|United_States|BOLD:ACY4297
NJCGS1013-11|Diacyclops_thomasi|Canada|BOLD:ADV7940
ZOOPS228-19|Diacyclops_thomasi|United_States|BOLD:ADV7940
ZOOPS595-20|Diacyclops_thomasi|United_States|BOLD:ADV7940
ZOOPS229-19|Diacyclops_thomasi|United_States|BOLD:ADV7940
COAPP338-13|Diacyclops_thomasi|Canada|BOLD:AAV0660
BACZP363-15|Diacyclops_thomasi|Canada|BOLD:ADV7941
BACZP724-16|Diacyclops_thomasi|Canada|BOLD:ADV7941
BACZP735-16|Diacyclops_thomasi|Canada|BOLD:ADV7941
BACZP739-16|Diacyclops_thomasi|Canada|BOLD:ADV7941
BACZP837-16|Diacyclops_thomasi|Canada|BOLD:ADV7941
NJCGS1032-11|Diacyclops_thomasi|Canada|BOLD:AAV0657
BACZP288-15|Diacyclops_thomasi|Canada|BOLD:AAV0657
BACZP369-15|Diacyclops_thomasi|Canada|BOLD:AAV0657
BACZP784-16|Diacyclops_thomasi|Canada|BOLD:AAV0657
BACZP828-16|Diacyclops_thomasi|Canada|BOLD:AAV0657
BACZP206-15|Diacyclops_thomasi|Canada|BOLD:AAV0657
BACZP337-15|Diacyclops_thomasi|Canada|BOLD:AAV0657
BACZP374-15|Diacyclops_thomasi|Canada|BOLD:AAV0657
BACZP691-16|Diacyclops_thomasi|Canada|BOLD:AAV0657
BACZP787-16|Diacyclops_thomasi|Canada|BOLD:AAV0657
BACZP819-16|Diacyclops_thomasi|Canada|BOLD:AAV0657
BACZP308-15|Diacyclops_thomasi|Canada|BOLD:AAV0657
BACZP321-15|Diacyclops_thomasi|Canada|BOLD:AAV0657
BACZP780-16|Diacyclops_thomasi|Canada|BOLD:AAV0657
SFEZ010-21|Cyclopidae|United_States|BOLD:AAV0657
SFEZ011-21|Cyclopidae|United_States|BOLD:AAV0657
SFEZ023-21|Cyclopidae|United_States|BOLD:AAV0657
BACZP333-15|Diacyclops_thomasi|Canada|BOLD:AAV0657
BACZP370-15|Diacyclops_thomasi|Canada|BOLD:AAV0657
BACZP774-16|Diacyclops_thomasi|Canada|BOLD:AAV0657
BACZP786-16|Diacyclops_thomasi|Canada|BOLD:AAV0657
ZOOPS034-18|Diacyclops_thomasi|Canada|BOLD:AAV0657
ZOOPS035-18|Diacyclops_thomasi|Canada|BOLD:AAV0657
ZOOPS596-20|Diacyclops_thomasi|United_States|BOLD:AAV0657
ZOOPS606-20|Diacyclops_thomasi|United_States|BOLD:AAV0657
SFEZ009-21|Cyclopidae|United_States|BOLD:AAV0657
ZOOPS231-19|Diacyclops_thomasi|United_States|BOLD:AAV0657
ZOOPS607-20|Diacyclops_thomasi|Canada|BOLD:AAV0657
ZOOPS230-19|Diacyclops_thomasi|United_States|BOLD:AAV0657
BACZP335-15|Diacyclops_thomasi|Canada|BOLD:AAV0657
BACZP791-16|Diacyclops_thomasi|Canada|BOLD:AAV0657
COAPP408-13|Diacyclops_thomasi|Canada|BOLD:AAV0657
ZOOPS594-20|Diacyclops_thomasi|United_States|BOLD:AEF5007
NJCGS1015-11|Diacyclops|Canada|BOLD:AAV0661
NJCGS1021-11|Diacyclops|Canada|BOLD:AAV0661
ZOOPS597-20|Diacyclops_thomasi|United_States|BOLD:AAV0661
DSMAX305-06|Cyclopidae|Canada|BOLD:AAG9771
DSMAX306-06|Cyclopidae|Canada|BOLD:AAG9771
DSMAX317-06|Cyclopidae|Canada|BOLD:AAG9771
DSMAX318-06|Cyclopidae|Canada|BOLD:AAG9771
DSMAX330-06|Cyclopidae|Canada|BOLD:AAG9771
DSMAX336-06|Cyclopidae|Canada|BOLD:AAG9771
DSMAX341-06|Cyclopidae|Canada|BOLD:AAG9771
DSMAX353-06|Cyclopidae|Canada|BOLD:AAG9771
DSMAX354-06|Cyclopidae|Canada|BOLD:AAG9771
DSMAX358-06|Cyclopidae|Canada|BOLD:AAG9771
DSMAX366-06|Cyclopidae|Canada|BOLD:AAG9771
DSMAX370-06|Cyclopidae|Canada|BOLD:AAG9771
DSMAX376-06|Cyclopidae|Canada|BOLD:AAG9771
DSMAX377-06|Cyclopidae|Canada|BOLD:AAG9771
DSMAX378-06|Cyclopidae|Canada|BOLD:AAG9771
DSMAX382-06|Cyclopidae|Canada|BOLD:AAG9771
DSMAX388-06|Cyclopidae|Canada|BOLD:AAG9771
DSMAX390-06|Cyclopidae|Canada|BOLD:AAG9771
DSMAX395-06|Cyclopidae|Canada|BOLD:AAG9771
DSMAX456-06|Cyclopidae|Canada|BOLD:AAG9771
DSMAX468-06|Cyclopidae|Canada|BOLD:AAG9771
ELPPC072-09|Cyclopidae|Canada|BOLD:AAG9771
OZFWZ304-11|Cyclopidae|Canada|BOLD:AAG9771
OZFWZ308-11|Cyclopidae|Canada|BOLD:AAG9771
OZFWC566-12|Cyclopidae|Canada|BOLD:AAG9771
OZFWC864-12|Cyclopidae|Canada|BOLD:AAG9771
OZFWC923-12|Cyclopidae|Canada|BOLD:AAG9771
AEDNA669-12|Cyclopidae|Canada|BOLD:AAG9771
OZFWZ286-11|Cyclopidae|Canada|BOLD:AAG9771
DSMAX365-06|Cyclopidae|Canada|BOLD:AAG9771
DSMAX432-06|Cyclopidae|Canada|BOLD:AAG9771
ELPPC063-09|Cyclopidae|Canada|BOLD:AAG9771
OZFWC288-11|Cyclopidae|Canada|BOLD:AAG9771
OZFWC753-12|Cyclopidae|Canada|BOLD:AAG9771
OZFWC756-12|Cyclopidae|Canada|BOLD:AAG9771
7
5
2
8
2
8
3
62
64

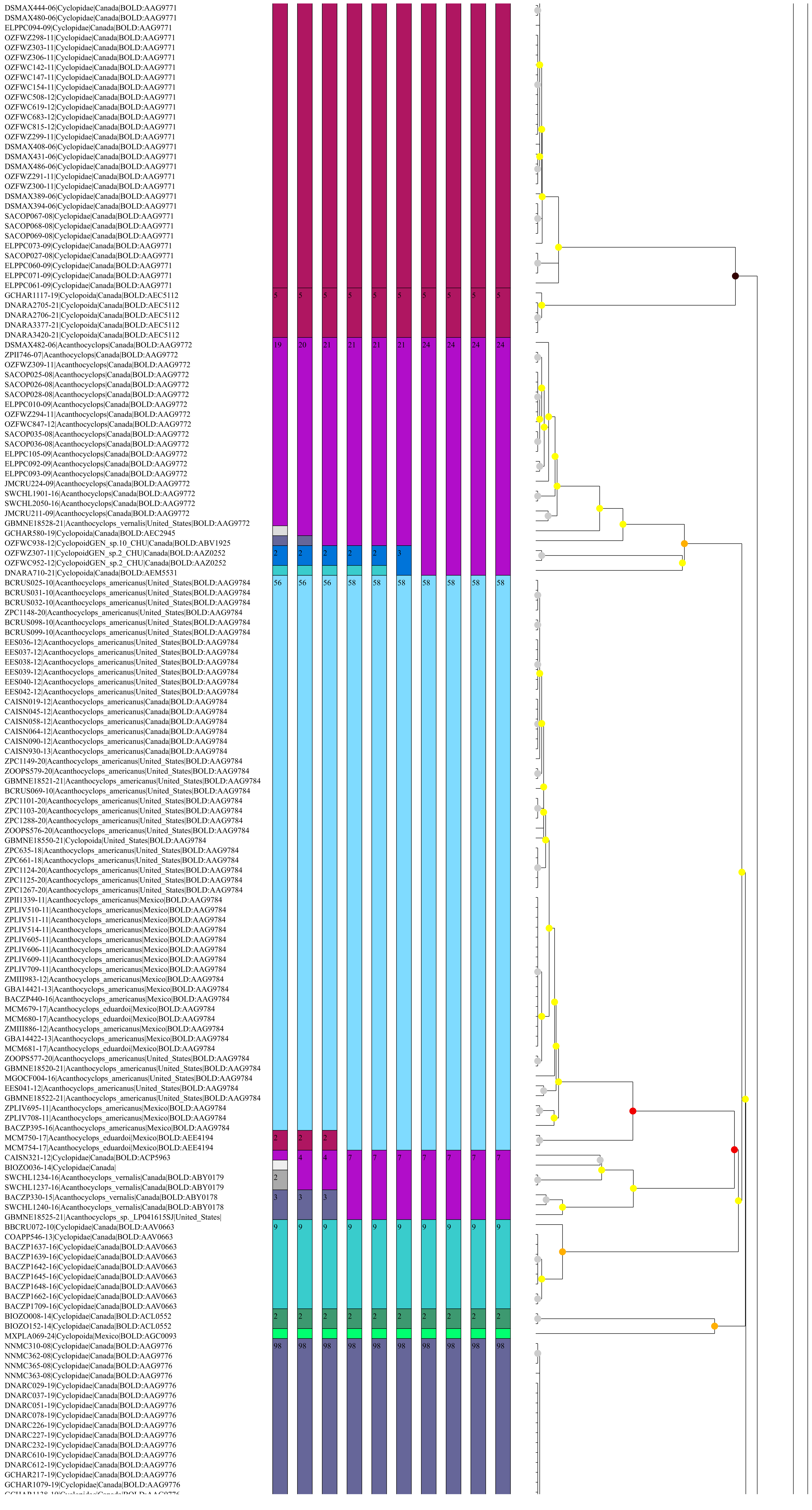
DSMAX444-06|Cyclopidae|Canada|BOLD:AAG9771
DSMAX480-06|Cyclopidae|Canada|BOLD:AAG9771
ELPPC094-09|Cyclopidae|Canada|BOLD:AAG9771
OZFWZ298-11|Cyclopidae|Canada|BOLD:AAG9771
OZFWZ303-11|Cyclopidae|Canada|BOLD:AAG9771
OZFWZ306-11|Cyclopidae|Canada|BOLD:AAG9771
OZFWC142-11|Cyclopidae|Canada|BOLD:AAG9771
OZFWC147-11|Cyclopidae|Canada|BOLD:AAG9771
OZFWC154-11|Cyclopidae|Canada|BOLD:AAG9771
OZFWC508-12|Cyclopidae|Canada|BOLD:AAG9771
OZFWC619-12|Cyclopidae|Canada|BOLD:AAG9771
OZFWC683-12|Cyclopidae|Canada|BOLD:AAG9771
OZFWC815-12|Cyclopidae|Canada|BOLD:AAG9771
OZFWZ299-11|Cyclopidae|Canada|BOLD:AAG9771
DSMAX408-06|Cyclopidae|Canada|BOLD:AAG9771
DSMAX431-06|Cyclopidae|Canada|BOLD:AAG9771
DSMAX486-06|Cyclopidae|Canada|BOLD:AAG9771
OZFWZ291-11|Cyclopidae|Canada|BOLD:AAG9771
OZFWZ300-11|Cyclopidae|Canada|BOLD:AAG9771
DSMAX389-06|Cyclopidae|Canada|BOLD:AAG9771
DSMAX394-06|Cyclopidae|Canada|BOLD:AAG9771
SACOP067-08|Cyclopidae|Canada|BOLD:AAG9771
SACOP068-08|Cyclopidae|Canada|BOLD:AAG9771
SACOP069-08|Cyclopidae|Canada|BOLD:AAG9771
ELPPC073-09|Cyclopidae|Canada|BOLD:AAG9771
SACOP027-08|Cyclopidae|Canada|BOLD:AAG9771
ELPPC060-09|Cyclopidae|Canada|BOLD:AAG9771
ELPPC071-09|Cyclopidae|Canada|BOLD:AAG9771
ELPPC061-09|Cyclopidae|Canada|BOLD:AAG9771
GCHAR1117-19|Cyclopoida|Canada|BOLD:AEC5112
DNARA2705-21|Cyclopoida|Canada|BOLD:AEC5112
DNARA2706-21|Cyclopoida|Canada|BOLD:AEC5112
DNARA3377-21|Cyclopoida|Canada|BOLD:AEC5112
DNARA3420-21|Cyclopoida|Canada|BOLD:AEC5112
DSMAX482-06|Acanthocyclops|Canada|BOLD:AAG9772
ZPII746-07|Acanthocyclops|Canada|BOLD:AAG9772
OZFWZ309-11|Acanthocyclops|Canada|BOLD:AAG9772
SACOP025-08|Acanthocyclops|Canada|BOLD:AAG9772
SACOP026-08|Acanthocyclops|Canada|BOLD:AAG9772
SACOP028-08|Acanthocyclops|Canada|BOLD:AAG9772
ELPPC010-09|Acanthocyclops|Canada|BOLD:AAG9772
OZFWZ294-11|Acanthocyclops|Canada|BOLD:AAG9772
OZFWC847-12|Acanthocyclops|Canada|BOLD:AAG9772
SACOP035-08|Acanthocyclops|Canada|BOLD:AAG9772
SACOP036-08|Acanthocyclops|Canada|BOLD:AAG9772
ELPPC105-09|Acanthocyclops|Canada|BOLD:AAG9772
ELPPC092-09|Acanthocyclops|Canada|BOLD:AAG9772
ELPPC093-09|Acanthocyclops|Canada|BOLD:AAG9772
JMCRU224-09|Acanthocyclops|Canada|BOLD:AAG9772
SWCHL1901-16|Acanthocyclops|Canada|BOLD:AAG9772
SWCHL2050-16|Acanthocyclops|Canada|BOLD:AAG9772
JMCRU211-09|Acanthocyclops|Canada|BOLD:AAG9772
GBMNE18528-21|Acanthocyclops_vernalis|United_States|BOLD:AAG9772
GCHAR580-19|Cyclopoida|Canada|BOLD:AEC2945
OZFWC938-12|CyclopoidGEN_sp.10_CHU|Canada|BOLD:ABV1925
OZFWZ307-11|CyclopoidGEN_sp.2_CHU|Canada|BOLD:AAZ0252
OZFWC952-12|CyclopoidGEN_sp.2_CHU|Canada|BOLD:AAZ0252
DNARA710-21|Cyclopoida|Canada|BOLD:AEM5531
BCRUS025-10|Acanthocyclops_americanus|United_States|BOLD:AAG9784
BCRUS031-10|Acanthocyclops_americanus|United_States|BOLD:AAG9784
BCRUS032-10|Acanthocyclops_americanus|United_States|BOLD:AAG9784
ZPC1148-20|Acanthocyclops_americanus|United_States|BOLD:AAG9784
BCRUS098-10|Acanthocyclops_americanus|United_States|BOLD:AAG9784
BCRUS099-10|Acanthocyclops_americanus|United_States|BOLD:AAG9784
EES036-12|Acanthocyclops_americanus|United_States|BOLD:AAG9784
EES037-12|Acanthocyclops_americanus|United_States|BOLD:AAG9784
EES038-12|Acanthocyclops_americanus|United_States|BOLD:AAG9784
EES039-12|Acanthocyclops_americanus|United_States|BOLD:AAG9784
EES040-12|Acanthocyclops_americanus|United_States|BOLD:AAG9784
EES042-12|Acanthocyclops_americanus|United_States|BOLD:AAG9784
CAISN019-12|Acanthocyclops_americanus|Canada|BOLD:AAG9784
CAISN045-12|Acanthocyclops_americanus|Canada|BOLD:AAG9784
CAISN058-12|Acanthocyclops_americanus|Canada|BOLD:AAG9784
CAISN064-12|Acanthocyclops_americanus|Canada|BOLD:AAG9784
CAISN090-12|Acanthocyclops_americanus|Canada|BOLD:AAG9784
CAISN930-13|Acanthocyclops_americanus|Canada|BOLD:AAG9784
ZPC1149-20|Acanthocyclops_americanus|United_States|BOLD:AAG9784
ZOOPS579-20|Acanthocyclops_americanus|United_States|BOLD:AAG9784
GBMNE18521-21|Acanthocyclops_americanus|United_States|BOLD:AAG9784
BCRUS069-10|Acanthocyclops_americanus|United_States|BOLD:AAG9784
ZPC1101-20|Acanthocyclops_americanus|United_States|BOLD:AAG9784
ZPC1103-20|Acanthocyclops_americanus|United_States|BOLD:AAG9784
ZPC1288-20|Acanthocyclops_americanus|United_States|BOLD:AAG9784
ZOOPS576-20|Acanthocyclops_americanus|United_States|BOLD:AAG9784
GBMNE18550-21|Cyclopoida|United_States|BOLD:AAG9784
ZPC635-18|Acanthocyclops_americanus|United_States|BOLD:AAG9784
ZPC661-18|Acanthocyclops_americanus|United_States|BOLD:AAG9784
ZPC1124-20|Acanthocyclops_americanus|United_States|BOLD:AAG9784
ZPC1125-20|Acanthocyclops_americanus|United_States|BOLD:AAG9784
ZPC1267-20|Acanthocyclops_americanus|United_States|BOLD:AAG9784
ZPII1339-11|Acanthocyclops_americanus|Mexico|BOLD:AAG9784
ZPLIV510-11|Acanthocyclops_americanus|Mexico|BOLD:AAG9784
ZPLIV511-11|Acanthocyclops_americanus|Mexico|BOLD:AAG9784
ZPLIV514-11|Acanthocyclops_americanus|Mexico|BOLD:AAG9784
ZPLIV605-11|Acanthocyclops_americanus|Mexico|BOLD:AAG9784
ZPLIV606-11|Acanthocyclops_americanus|Mexico|BOLD:AAG9784
ZPLIV609-11|Acanthocyclops_americanus|Mexico|BOLD:AAG9784
ZPLIV709-11|Acanthocyclops_americanus|Mexico|BOLD:AAG9784
ZMIII983-12|Acanthocyclops_americanus|Mexico|BOLD:AAG9784
GBA14421-13|Acanthocyclops_americanus|Mexico|BOLD:AAG9784
BACZP440-16|Acanthocyclops_americanus|Mexico|BOLD:AAG9784
MCM679-17|Acanthocyclops_eduardoi|Mexico|BOLD:AAG9784
MCM680-17|Acanthocyclops_eduardoi|Mexico|BOLD:AAG9784
ZMIII886-12|Acanthocyclops_americanus|Mexico|BOLD:AAG9784
GBA14422-13|Acanthocyclops_americanus|Mexico|BOLD:AAG9784
MCM681-17|Acanthocyclops_eduardoi|Mexico|BOLD:AAG9784
ZOOPS577-20|Acanthocyclops_americanus|United_States|BOLD:AAG9784
GBMNE18520-21|Acanthocyclops_americanus|United_States|BOLD:AAG9784
MGOCF004-16|Acanthocyclops_americanus|United_States|BOLD:AAG9784
EES041-12|Acanthocyclops_americanus|United_States|BOLD:AAG9784
GBMNE18522-21|Acanthocyclops_americanus|United_States|BOLD:AAG9784
ZPLIV695-11|Acanthocyclops_americanus|Mexico|BOLD:AAG9784
ZPLIV708-11|Acanthocyclops_americanus|Mexico|BOLD:AAG9784
BACZP395-16|Acanthocyclops_americanus|Mexico|BOLD:AAG9784
MCM750-17|Acanthocyclops_eduardoi|Mexico|BOLD:AEE4194
MCM754-17|Acanthocyclops_eduardoi|Mexico|BOLD:AEE4194
CAISN321-12|Cyclopidae|Canada|BOLD:ACP5963
BIOZO036-14|Cyclopidae|Canada|
SWCHL1234-16|Acanthocyclops_vernalis|Canada|BOLD:ABY0179
SWCHL1237-16|Acanthocyclops_vernalis|Canada|BOLD:ABY0179
BACZP330-15|Acanthocyclops_vernalis|Canada|BOLD:ABY0178
SWCHL1240-16|Acanthocyclops_vernalis|Canada|BOLD:ABY0178
GBMNE18525-21|Acanthocyclops_sp._LP041615SJ|United_States|
BBCRU072-10|Cyclopidae|Canada|BOLD:AAV0663
COAPP546-13|Cyclopidae|Canada|BOLD:AAV0663
BACZP1637-16|Cyclopidae|Canada|BOLD:AAV0663
BACZP1639-16|Cyclopidae|Canada|BOLD:AAV0663
BACZP1642-16|Cyclopidae|Canada|BOLD:AAV0663
BACZP1645-16|Cyclopidae|Canada|BOLD:AAV0663
BACZP1648-16|Cyclopidae|Canada|BOLD:AAV0663
BACZP1662-16|Cyclopidae|Canada|BOLD:AAV0663
BACZP1709-16|Cyclopidae|Canada|BOLD:AAV0663
BIOZO008-14|Cyclopidae|Canada|BOLD:ACL0552
BIOZO152-14|Cyclopidae|Canada|BOLD:ACL0552
MXPLA069-24|Cyclopoida|Mexico|BOLD:AGC0093
NNMC310-08|Cyclopidae|Canada|BOLD:AAG9776
NNMC362-08|Cyclopidae|Canada|BOLD:AAG9776
NNMC365-08|Cyclopidae|Canada|BOLD:AAG9776
NNMC363-08|Cyclopidae|Canada|BOLD:AAG9776
DNARC029-19|Cyclopidae|Canada|BOLD:AAG9776
DNARC037-19|Cyclopidae|Canada|BOLD:AAG9776
DNARC051-19|Cyclopidae|Canada|BOLD:AAG9776
DNARC078-19|Cyclopidae|Canada|BOLD:AAG9776
DNARC226-19|Cyclopidae|Canada|BOLD:AAG9776
DNARC227-19|Cyclopidae|Canada|BOLD:AAG9776
DNARC232-19|Cyclopidae|Canada|BOLD:AAG9776
DNARC610-19|Cyclopidae|Canada|BOLD:AAG9776
DNARC612-19|Cyclopidae|Canada|BOLD:AAG9776
GCHAR217-19|Cyclopidae|Canada|BOLD:AAG9776
GCHAR1079-19|Cyclopidae|Canada|BOLD:AAG9776

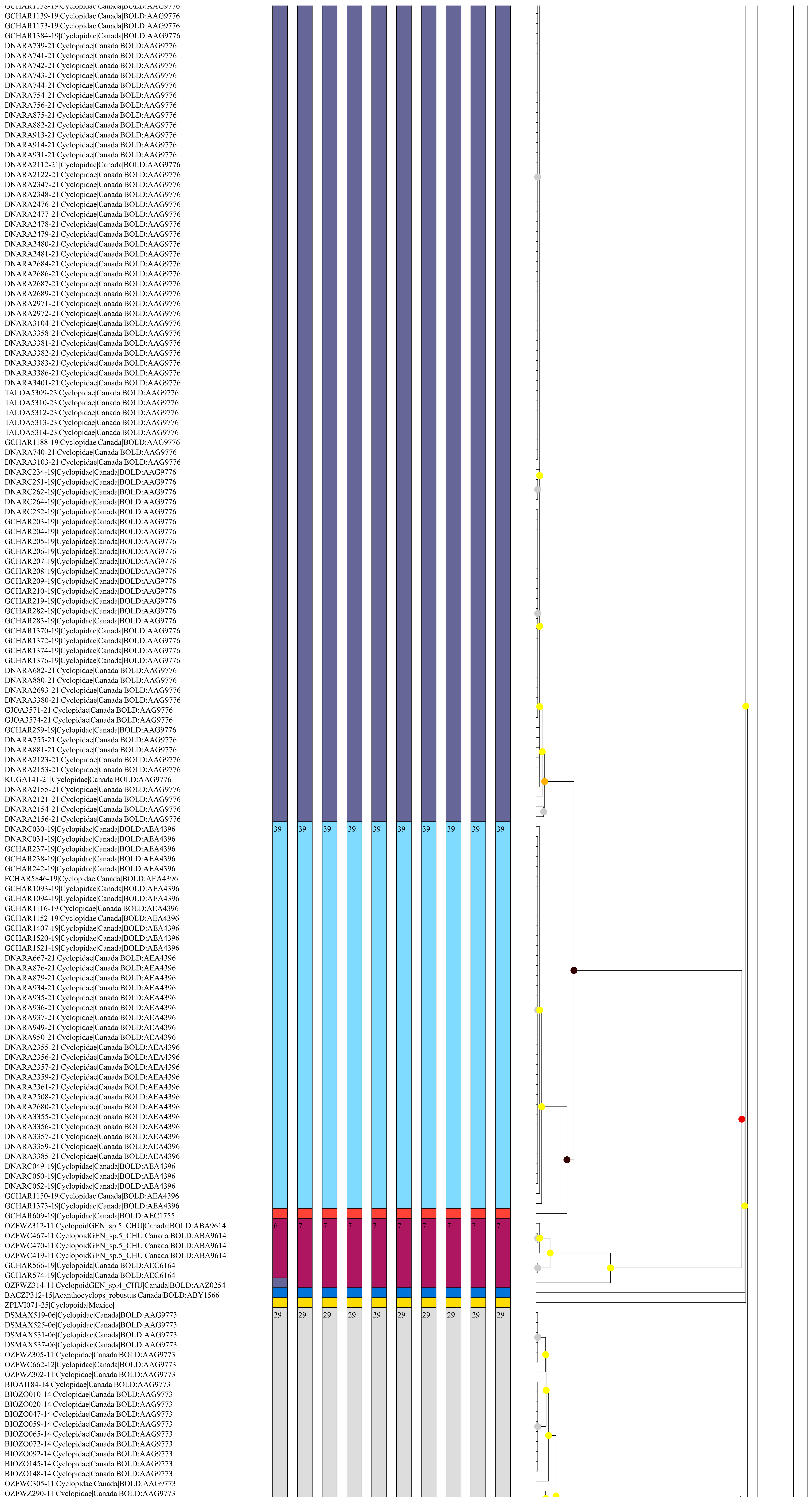
GCHAR1138-19|Cyclopidae|Canada|BOLD:AAG9776
GCHAR1139-19|Cyclopidae|Canada|BOLD:AAG9776
GCHAR1173-19|Cyclopidae|Canada|BOLD:AAG9776
GCHAR1384-19|Cyclopidae|Canada|BOLD:AAG9776
DNARA739-21|Cyclopidae|Canada|BOLD:AAG9776
DNARA741-21|Cyclopidae|Canada|BOLD:AAG9776
DNARA742-21|Cyclopidae|Canada|BOLD:AAG9776
DNARA743-21|Cyclopidae|Canada|BOLD:AAG9776
DNARA744-21|Cyclopidae|Canada|BOLD:AAG9776
DNARA754-21|Cyclopidae|Canada|BOLD:AAG9776
DNARA756-21|Cyclopidae|Canada|BOLD:AAG9776
DNARA875-21|Cyclopidae|Canada|BOLD:AAG9776
DNARA882-21|Cyclopidae|Canada|BOLD:AAG9776
DNARA913-21|Cyclopidae|Canada|BOLD:AAG9776
DNARA914-21|Cyclopidae|Canada|BOLD:AAG9776
DNARA931-21|Cyclopidae|Canada|BOLD:AAG9776
DNARA2112-21|Cyclopidae|Canada|BOLD:AAG9776
DNARA2122-21|Cyclopidae|Canada|BOLD:AAG9776
DNARA2347-21|Cyclopidae|Canada|BOLD:AAG9776
DNARA2348-21|Cyclopidae|Canada|BOLD:AAG9776
DNARA2476-21|Cyclopidae|Canada|BOLD:AAG9776
DNARA2477-21|Cyclopidae|Canada|BOLD:AAG9776
DNARA2478-21|Cyclopidae|Canada|BOLD:AAG9776
DNARA2479-21|Cyclopidae|Canada|BOLD:AAG9776
DNARA2480-21|Cyclopidae|Canada|BOLD:AAG9776
DNARA2481-21|Cyclopidae|Canada|BOLD:AAG9776
DNARA2684-21|Cyclopidae|Canada|BOLD:AAG9776
DNARA2686-21|Cyclopidae|Canada|BOLD:AAG9776
DNARA2687-21|Cyclopidae|Canada|BOLD:AAG9776
DNARA2689-21|Cyclopidae|Canada|BOLD:AAG9776
DNARA2971-21|Cyclopidae|Canada|BOLD:AAG9776
DNARA2972-21|Cyclopidae|Canada|BOLD:AAG9776
DNARA3104-21|Cyclopidae|Canada|BOLD:AAG9776
DNARA3358-21|Cyclopidae|Canada|BOLD:AAG9776
DNARA3381-21|Cyclopidae|Canada|BOLD:AAG9776
DNARA3382-21|Cyclopidae|Canada|BOLD:AAG9776
DNARA3383-21|Cyclopidae|Canada|BOLD:AAG9776
DNARA3386-21|Cyclopidae|Canada|BOLD:AAG9776
DNARA3401-21|Cyclopidae|Canada|BOLD:AAG9776
TALOA5309-23|Cyclopidae|Canada|BOLD:AAG9776
TALOA5310-23|Cyclopidae|Canada|BOLD:AAG9776
TALOA5312-23|Cyclopidae|Canada|BOLD:AAG9776
TALOA5313-23|Cyclopidae|Canada|BOLD:AAG9776
TALOA5314-23|Cyclopidae|Canada|BOLD:AAG9776
GCHAR1188-19|Cyclopidae|Canada|BOLD:AAG9776
DNARA740-21|Cyclopidae|Canada|BOLD:AAG9776
DNARA3103-21|Cyclopidae|Canada|BOLD:AAG9776
DNARC234-19|Cyclopidae|Canada|BOLD:AAG9776
DNARC251-19|Cyclopidae|Canada|BOLD:AAG9776
DNARC262-19|Cyclopidae|Canada|BOLD:AAG9776
DNARC264-19|Cyclopidae|Canada|BOLD:AAG9776
DNARC252-19|Cyclopidae|Canada|BOLD:AAG9776
GCHAR203-19|Cyclopidae|Canada|BOLD:AAG9776
GCHAR204-19|Cyclopidae|Canada|BOLD:AAG9776
GCHAR205-19|Cyclopidae|Canada|BOLD:AAG9776
GCHAR206-19|Cyclopidae|Canada|BOLD:AAG9776
GCHAR207-19|Cyclopidae|Canada|BOLD:AAG9776
GCHAR208-19|Cyclopidae|Canada|BOLD:AAG9776
GCHAR209-19|Cyclopidae|Canada|BOLD:AAG9776
GCHAR210-19|Cyclopidae|Canada|BOLD:AAG9776
GCHAR219-19|Cyclopidae|Canada|BOLD:AAG9776
GCHAR282-19|Cyclopidae|Canada|BOLD:AAG9776
GCHAR283-19|Cyclopidae|Canada|BOLD:AAG9776
GCHAR1370-19|Cyclopidae|Canada|BOLD:AAG9776
GCHAR1372-19|Cyclopidae|Canada|BOLD:AAG9776
GCHAR1374-19|Cyclopidae|Canada|BOLD:AAG9776
GCHAR1376-19|Cyclopidae|Canada|BOLD:AAG9776
DNARA682-21|Cyclopidae|Canada|BOLD:AAG9776
DNARA880-21|Cyclopidae|Canada|BOLD:AAG9776
DNARA2693-21|Cyclopidae|Canada|BOLD:AAG9776
DNARA3380-21|Cyclopidae|Canada|BOLD:AAG9776
GJOA3571-21|Cyclopidae|Canada|BOLD:AAG9776
GJOA3574-21|Cyclopidae|Canada|BOLD:AAG9776
GCHAR259-19|Cyclopidae|Canada|BOLD:AAG9776
DNARA755-21|Cyclopidae|Canada|BOLD:AAG9776
DNARA881-21|Cyclopidae|Canada|BOLD:AAG9776
DNARA2123-21|Cyclopidae|Canada|BOLD:AAG9776
DNARA2153-21|Cyclopidae|Canada|BOLD:AAG9776
KUGA141-21|Cyclopidae|Canada|BOLD:AAG9776
DNARA2155-21|Cyclopidae|Canada|BOLD:AAG9776
DNARA2121-21|Cyclopidae|Canada|BOLD:AAG9776
DNARA2154-21|Cyclopidae|Canada|BOLD:AAG9776
DNARA2156-21|Cyclopidae|Canada|BOLD:AAG9776
DNARC030-19|Cyclopidae|Canada|BOLD:AEA4396
DNARC031-19|Cyclopidae|Canada|BOLD:AEA4396
GCHAR237-19|Cyclopidae|Canada|BOLD:AEA4396
GCHAR238-19|Cyclopidae|Canada|BOLD:AEA4396
GCHAR242-19|Cyclopidae|Canada|BOLD:AEA4396
FCHAR5846-19|Cyclopidae|Canada|BOLD:AEA4396
GCHAR1093-19|Cyclopidae|Canada|BOLD:AEA4396
GCHAR1094-19|Cyclopidae|Canada|BOLD:AEA4396
GCHAR1116-19|Cyclopidae|Canada|BOLD:AEA4396
GCHAR1152-19|Cyclopidae|Canada|BOLD:AEA4396
GCHAR1407-19|Cyclopidae|Canada|BOLD:AEA4396
GCHAR1520-19|Cyclopidae|Canada|BOLD:AEA4396
GCHAR1521-19|Cyclopidae|Canada|BOLD:AEA4396
DNARA667-21|Cyclopidae|Canada|BOLD:AEA4396
DNARA876-21|Cyclopidae|Canada|BOLD:AEA4396
DNARA879-21|Cyclopidae|Canada|BOLD:AEA4396
DNARA934-21|Cyclopidae|Canada|BOLD:AEA4396
DNARA935-21|Cyclopidae|Canada|BOLD:AEA4396
DNARA936-21|Cyclopidae|Canada|BOLD:AEA4396
DNARA937-21|Cyclopidae|Canada|BOLD:AEA4396
DNARA949-21|Cyclopidae|Canada|BOLD:AEA4396
DNARA950-21|Cyclopidae|Canada|BOLD:AEA4396
DNARA2355-21|Cyclopidae|Canada|BOLD:AEA4396
DNARA2356-21|Cyclopidae|Canada|BOLD:AEA4396
DNARA2357-21|Cyclopidae|Canada|BOLD:AEA4396
DNARA2359-21|Cyclopidae|Canada|BOLD:AEA4396
DNARA2361-21|Cyclopidae|Canada|BOLD:AEA4396
DNARA2508-21|Cyclopidae|Canada|BOLD:AEA4396
DNARA2680-21|Cyclopidae|Canada|BOLD:AEA4396
DNARA3355-21|Cyclopidae|Canada|BOLD:AEA4396
DNARA3356-21|Cyclopidae|Canada|BOLD:AEA4396
DNARA3357-21|Cyclopidae|Canada|BOLD:AEA4396
DNARA3359-21|Cyclopidae|Canada|BOLD:AEA4396
DNARA3385-21|Cyclopidae|Canada|BOLD:AEA4396
DNARC049-19|Cyclopidae|Canada|BOLD:AEA4396
DNARC050-19|Cyclopidae|Canada|BOLD:AEA4396
DNARC052-19|Cyclopidae|Canada|BOLD:AEA4396
GCHAR1150-19|Cyclopidae|Canada|BOLD:AEA4396
GCHAR1373-19|Cyclopidae|Canada|BOLD:AEA4396
GCHAR609-19|Cyclopidae|Canada|BOLD:AEC1755
OZFWZ312-11|CyclopoidGEN_sp.5_CHU|Canada|BOLD:ABA9614
OZFWC467-11|CyclopoidGEN_sp.5_CHU|Canada|BOLD:ABA9614
OZFWC470-11|CyclopoidGEN_sp.5_CHU|Canada|BOLD:ABA9614
OZFWC419-11|CyclopoidGEN_sp.5_CHU|Canada|BOLD:ABA9614
GCHAR566-19|Cyclopoida|Canada|BOLD:AEC6164
GCHAR574-19|Cyclopoida|Canada|BOLD:AEC6164
OZFWZ314-11|CyclopoidGEN_sp.4_CHU|Canada|BOLD:AAZ0254
BACZP312-15|Acanthocyclops_robustus|Canada|BOLD:ABY1566
ZPLVI071-25|Cyclopoida|Mexico|
DSMAX519-06|Cyclopidae|Canada|BOLD:AAG9773
DSMAX525-06|Cyclopidae|Canada|BOLD:AAG9773
DSMAX531-06|Cyclopidae|Canada|BOLD:AAG9773
DSMAX537-06|Cyclopidae|Canada|BOLD:AAG9773
OZFWZ305-11|Cyclopidae|Canada|BOLD:AAG9773
OZFWC662-12|Cyclopidae|Canada|BOLD:AAG9773
OZFWZ302-11|Cyclopidae|Canada|BOLD:AAG9773
BIOAI184-14|Cyclopidae|Canada|BOLD:AAG9773
BIOZO010-14|Cyclopidae|Canada|BOLD:AAG9773
BIOZO020-14|Cyclopidae|Canada|BOLD:AAG9773
BIOZO047-14|Cyclopidae|Canada|BOLD:AAG9773
BIOZO059-14|Cyclopidae|Canada|BOLD:AAG9773
BIOZO065-14|Cyclopidae|Canada|BOLD:AAG9773
BIOZO072-14|Cyclopidae|Canada|BOLD:AAG9773
BIOZO092-14|Cyclopidae|Canada|BOLD:AAG9773
BIOZO145-14|Cyclopidae|Canada|BOLD:AAG9773
BIOZO148-14|Cyclopidae|Canada|BOLD:AAG9773
OZFWC305-11|Cyclopidae|Canada|BOLD:AAG9773
OZFWZ290-11|Cyclopidae|Canada|BOLD:AAG9773
39 39 39 39 39 39 39 39 39 39
6 7 7 7 7 7 7 7 7 7
29 29 29 29 29 29 29 29 29 29

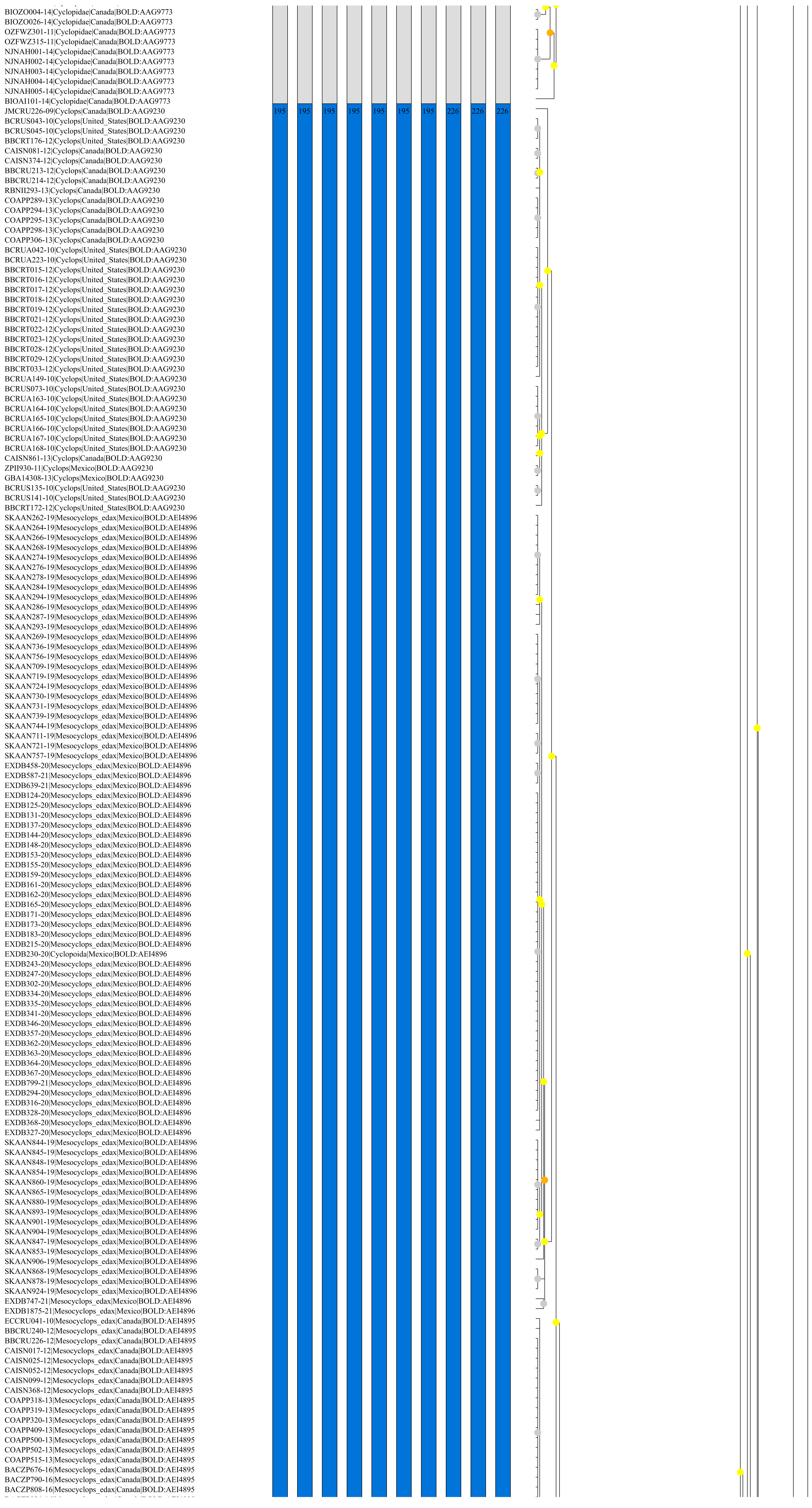

BIOZO004-14|Cyclopidae|Canada|BOLD:AAG9773
BIOZO026-14|Cyclopidae|Canada|BOLD:AAG9773
OZFWZ301-11|Cyclopidae|Canada|BOLD:AAG9773
OZFWZ315-11|Cyclopidae|Canada|BOLD:AAG9773
NJNAH001-14|Cyclopidae|Canada|BOLD:AAG9773
NJNAH002-14|Cyclopidae|Canada|BOLD:AAG9773
NJNAH003-14|Cyclopidae|Canada|BOLD:AAG9773
NJNAH004-14|Cyclopidae|Canada|BOLD:AAG9773
NJNAH005-14|Cyclopidae|Canada|BOLD:AAG9773
BIOAI101-14|Cyclopidae|Canada|BOLD:AAG9773
JMCRU226-09|Cyclops|Canada|BOLD:AAG9230
BCRUS043-10|Cyclops|United_States|BOLD:AAG9230
BCRUS045-10|Cyclops|United_States|BOLD:AAG9230
BBCRT176-12|Cyclops|United_States|BOLD:AAG9230
CAISN081-12|Cyclops|Canada|BOLD:AAG9230
CAISN374-12|Cyclops|Canada|BOLD:AAG9230
BBCRU213-12|Cyclops|Canada|BOLD:AAG9230
BBCRU214-12|Cyclops|Canada|BOLD:AAG9230
RBNII293-13|Cyclops|Canada|BOLD:AAG9230
COAPP289-13|Cyclops|Canada|BOLD:AAG9230
COAPP294-13|Cyclops|Canada|BOLD:AAG9230
COAPP295-13|Cyclops|Canada|BOLD:AAG9230
COAPP298-13|Cyclops|Canada|BOLD:AAG9230
COAPP306-13|Cyclops|Canada|BOLD:AAG9230
BCRUA042-10|Cyclops|United_States|BOLD:AAG9230
BCRUA223-10|Cyclops|United_States|BOLD:AAG9230
BBCRT015-12|Cyclops|United_States|BOLD:AAG9230
BBCRT016-12|Cyclops|United_States|BOLD:AAG9230
BBCRT017-12|Cyclops|United_States|BOLD:AAG9230
BBCRT018-12|Cyclops|United_States|BOLD:AAG9230
BBCRT019-12|Cyclops|United_States|BOLD:AAG9230
BBCRT021-12|Cyclops|United_States|BOLD:AAG9230
BBCRT022-12|Cyclops|United_States|BOLD:AAG9230
BBCRT023-12|Cyclops|United_States|BOLD:AAG9230
BBCRT028-12|Cyclops|United_States|BOLD:AAG9230
BBCRT029-12|Cyclops|United_States|BOLD:AAG9230
BBCRT033-12|Cyclops|United_States|BOLD:AAG9230
BCRUA149-10|Cyclops|United_States|BOLD:AAG9230
BCRUS073-10|Cyclops|United_States|BOLD:AAG9230
BCRUA163-10|Cyclops|United_States|BOLD:AAG9230
BCRUA164-10|Cyclops|United_States|BOLD:AAG9230
BCRUA165-10|Cyclops|United_States|BOLD:AAG9230
BCRUA166-10|Cyclops|United_States|BOLD:AAG9230
BCRUA167-10|Cyclops|United_States|BOLD:AAG9230
BCRUA168-10|Cyclops|United_States|BOLD:AAG9230
CAISN861-13|Cyclops|Canada|BOLD:AAG9230
ZPII930-11|Cyclops|Mexico|BOLD:AAG9230
GBA14308-13|Cyclops|Mexico|BOLD:AAG9230
BCRUS135-10|Cyclops|United_States|BOLD:AAG9230
BCRUS141-10|Cyclops|United_States|BOLD:AAG9230
BBCRT172-12|Cyclops|United_States|BOLD:AAG9230
SKAAN262-19|Mesocyclops_edax|Mexico|BOLD:AEI4896
SKAAN264-19|Mesocyclops_edax|Mexico|BOLD:AEI4896
SKAAN266-19|Mesocyclops_edax|Mexico|BOLD:AEI4896
SKAAN268-19|Mesocyclops_edax|Mexico|BOLD:AEI4896
SKAAN274-19|Mesocyclops_edax|Mexico|BOLD:AEI4896
SKAAN276-19|Mesocyclops_edax|Mexico|BOLD:AEI4896
SKAAN278-19|Mesocyclops_edax|Mexico|BOLD:AEI4896
SKAAN284-19|Mesocyclops_edax|Mexico|BOLD:AEI4896
SKAAN294-19|Mesocyclops_edax|Mexico|BOLD:AEI4896
SKAAN286-19|Mesocyclops_edax|Mexico|BOLD:AEI4896
SKAAN287-19|Mesocyclops_edax|Mexico|BOLD:AEI4896
SKAAN293-19|Mesocyclops_edax|Mexico|BOLD:AEI4896
SKAAN269-19|Mesocyclops_edax|Mexico|BOLD:AEI4896
SKAAN736-19|Mesocyclops_edax|Mexico|BOLD:AEI4896
SKAAN756-19|Mesocyclops_edax|Mexico|BOLD:AEI4896
SKAAN709-19|Mesocyclops_edax|Mexico|BOLD:AEI4896
SKAAN719-19|Mesocyclops_edax|Mexico|BOLD:AEI4896
SKAAN724-19|Mesocyclops_edax|Mexico|BOLD:AEI4896
SKAAN730-19|Mesocyclops_edax|Mexico|BOLD:AEI4896
SKAAN731-19|Mesocyclops_edax|Mexico|BOLD:AEI4896
SKAAN739-19|Mesocyclops_edax|Mexico|BOLD:AEI4896
SKAAN744-19|Mesocyclops_edax|Mexico|BOLD:AEI4896
SKAAN711-19|Mesocyclops_edax|Mexico|BOLD:AEI4896
SKAAN721-19|Mesocyclops_edax|Mexico|BOLD:AEI4896
SKAAN757-19|Mesocyclops_edax|Mexico|BOLD:AEI4896
EXDB458-20|Mesocyclops_edax|Mexico|BOLD:AEI4896
EXDB587-21|Mesocyclops_edax|Mexico|BOLD:AEI4896
EXDB639-21|Mesocyclops_edax|Mexico|BOLD:AEI4896
EXDB124-20|Mesocyclops_edax|Mexico|BOLD:AEI4896
EXDB125-20|Mesocyclops_edax|Mexico|BOLD:AEI4896
EXDB131-20|Mesocyclops_edax|Mexico|BOLD:AEI4896
EXDB137-20|Mesocyclops_edax|Mexico|BOLD:AEI4896
EXDB144-20|Mesocyclops_edax|Mexico|BOLD:AEI4896
EXDB148-20|Mesocyclops_edax|Mexico|BOLD:AEI4896
EXDB153-20|Mesocyclops_edax|Mexico|BOLD:AEI4896
EXDB155-20|Mesocyclops_edax|Mexico|BOLD:AEI4896
EXDB159-20|Mesocyclops_edax|Mexico|BOLD:AEI4896
EXDB161-20|Mesocyclops_edax|Mexico|BOLD:AEI4896
EXDB162-20|Mesocyclops_edax|Mexico|BOLD:AEI4896
EXDB165-20|Mesocyclops_edax|Mexico|BOLD:AEI4896
EXDB171-20|Mesocyclops_edax|Mexico|BOLD:AEI4896
EXDB173-20|Mesocyclops_edax|Mexico|BOLD:AEI4896
EXDB183-20|Mesocyclops_edax|Mexico|BOLD:AEI4896
EXDB215-20|Mesocyclops_edax|Mexico|BOLD:AEI4896
EXDB230-20|Cyclopoida|Mexico|BOLD:AEI4896
EXDB243-20|Mesocyclops_edax|Mexico|BOLD:AEI4896
EXDB247-20|Mesocyclops_edax|Mexico|BOLD:AEI4896
EXDB302-20|Mesocyclops_edax|Mexico|BOLD:AEI4896
EXDB334-20|Mesocyclops_edax|Mexico|BOLD:AEI4896
EXDB335-20|Mesocyclops_edax|Mexico|BOLD:AEI4896
EXDB341-20|Mesocyclops_edax|Mexico|BOLD:AEI4896
EXDB346-20|Mesocyclops_edax|Mexico|BOLD:AEI4896
EXDB357-20|Mesocyclops_edax|Mexico|BOLD:AEI4896
EXDB362-20|Mesocyclops_edax|Mexico|BOLD:AEI4896
EXDB363-20|Mesocyclops_edax|Mexico|BOLD:AEI4896
EXDB364-20|Mesocyclops_edax|Mexico|BOLD:AEI4896
EXDB367-20|Mesocyclops_edax|Mexico|BOLD:AEI4896
EXDB799-21|Mesocyclops_edax|Mexico|BOLD:AEI4896
EXDB294-20|Mesocyclops_edax|Mexico|BOLD:AEI4896
EXDB316-20|Mesocyclops_edax|Mexico|BOLD:AEI4896
EXDB328-20|Mesocyclops_edax|Mexico|BOLD:AEI4896
EXDB368-20|Mesocyclops_edax|Mexico|BOLD:AEI4896
EXDB327-20|Mesocyclops_edax|Mexico|BOLD:AEI4896
SKAAN844-19|Mesocyclops_edax|Mexico|BOLD:AEI4896
SKAAN845-19|Mesocyclops_edax|Mexico|BOLD:AEI4896
SKAAN848-19|Mesocyclops_edax|Mexico|BOLD:AEI4896
SKAAN854-19|Mesocyclops_edax|Mexico|BOLD:AEI4896
SKAAN860-19|Mesocyclops_edax|Mexico|BOLD:AEI4896
SKAAN865-19|Mesocyclops_edax|Mexico|BOLD:AEI4896
SKAAN880-19|Mesocyclops_edax|Mexico|BOLD:AEI4896
SKAAN893-19|Mesocyclops_edax|Mexico|BOLD:AEI4896
SKAAN901-19|Mesocyclops_edax|Mexico|BOLD:AEI4896
SKAAN904-19|Mesocyclops_edax|Mexico|BOLD:AEI4896
SKAAN847-19|Mesocyclops_edax|Mexico|BOLD:AEI4896
SKAAN853-19|Mesocyclops_edax|Mexico|BOLD:AEI4896
SKAAN906-19|Mesocyclops_edax|Mexico|BOLD:AEI4896
SKAAN868-19|Mesocyclops_edax|Mexico|BOLD:AEI4896
SKAAN878-19|Mesocyclops_edax|Mexico|BOLD:AEI4896
SKAAN924-19|Mesocyclops_edax|Mexico|BOLD:AEI4896
EXDB747-21|Mesocyclops_edax|Mexico|BOLD:AEI4896
EXDB1875-21|Mesocyclops_edax|Mexico|BOLD:AEI4896
ECCRU041-10|Mesocyclops_edax|Canada|BOLD:AEI4895
BBCRU240-12|Mesocyclops_edax|Canada|BOLD:AEI4895
BBCRU226-12|Mesocyclops_edax|Canada|BOLD:AEI4895
CAISN017-12|Mesocyclops_edax|Canada|BOLD:AEI4895
CAISN025-12|Mesocyclops_edax|Canada|BOLD:AEI4895
CAISN052-12|Mesocyclops_edax|Canada|BOLD:AEI4895
CAISN099-12|Mesocyclops_edax|Canada|BOLD:AEI4895
CAISN368-12|Mesocyclops_edax|Canada|BOLD:AEI4895
COAPP318-13|Mesocyclops_edax|Canada|BOLD:AEI4895
COAPP319-13|Mesocyclops_edax|Canada|BOLD:AEI4895
COAPP320-13|Mesocyclops_edax|Canada|BOLD:AEI4895
COAPP409-13|Mesocyclops_edax|Canada|BOLD:AEI4895
COAPP500-13|Mesocyclops_edax|Canada|BOLD:AEI4895
COAPP502-13|Mesocyclops_edax|Canada|BOLD:AEI4895
COAPP515-13|Mesocyclops_edax|Canada|BOLD:AEI4895
BACZP676-16|Mesocyclops_edax|Canada|BOLD:AEI4895
BACZP790-16|Mesocyclops_edax|Canada|BOLD:AEI4895
BACZP808-16|Mesocyclops_edax|Canada|BOLD:AEI4895
195
195
195
195
195
195
195
226
226
226

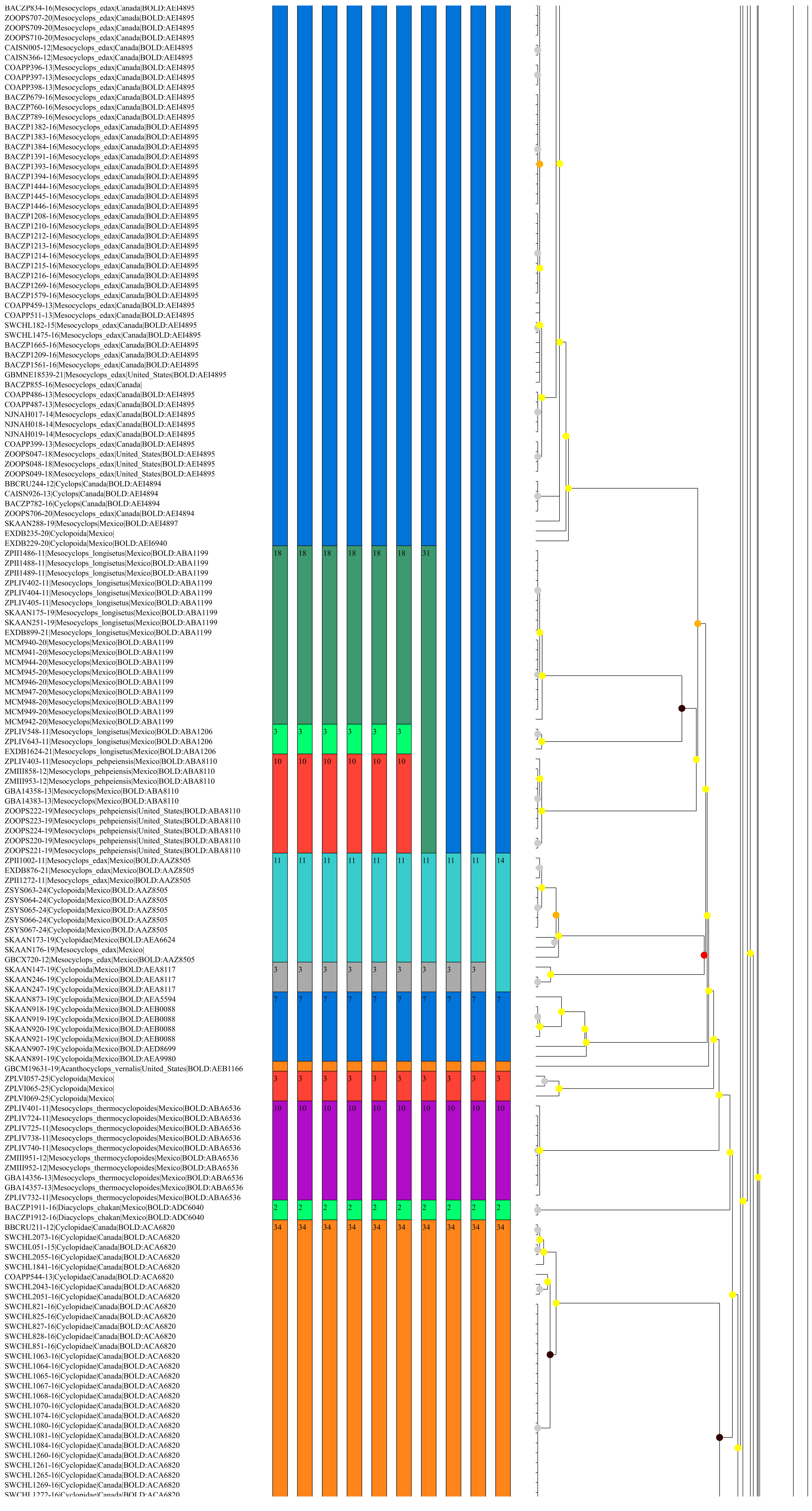
BACZP834-16|Mesocyclops_edax|Canada|BOLD:AEI4895
ZOOPS707-20|Mesocyclops_edax|Canada|BOLD:AEI4895
ZOOPS709-20|Mesocyclops_edax|Canada|BOLD:AEI4895
ZOOPS710-20|Mesocyclops_edax|Canada|BOLD:AEI4895
CAISN005-12|Mesocyclops_edax|Canada|BOLD:AEI4895
CAISN366-12|Mesocyclops_edax|Canada|BOLD:AEI4895
COAPP396-13|Mesocyclops_edax|Canada|BOLD:AEI4895
COAPP397-13|Mesocyclops_edax|Canada|BOLD:AEI4895
COAPP398-13|Mesocyclops_edax|Canada|BOLD:AEI4895
BACZP679-16|Mesocyclops_edax|Canada|BOLD:AEI4895
BACZP760-16|Mesocyclops_edax|Canada|BOLD:AEI4895
BACZP789-16|Mesocyclops_edax|Canada|BOLD:AEI4895
BACZP1382-16|Mesocyclops_edax|Canada|BOLD:AEI4895
BACZP1383-16|Mesocyclops_edax|Canada|BOLD:AEI4895
BACZP1384-16|Mesocyclops_edax|Canada|BOLD:AEI4895
BACZP1391-16|Mesocyclops_edax|Canada|BOLD:AEI4895
BACZP1393-16|Mesocyclops_edax|Canada|BOLD:AEI4895
BACZP1394-16|Mesocyclops_edax|Canada|BOLD:AEI4895
BACZP1444-16|Mesocyclops_edax|Canada|BOLD:AEI4895
BACZP1445-16|Mesocyclops_edax|Canada|BOLD:AEI4895
BACZP1446-16|Mesocyclops_edax|Canada|BOLD:AEI4895
BACZP1208-16|Mesocyclops_edax|Canada|BOLD:AEI4895
BACZP1210-16|Mesocyclops_edax|Canada|BOLD:AEI4895
BACZP1212-16|Mesocyclops_edax|Canada|BOLD:AEI4895
BACZP1213-16|Mesocyclops_edax|Canada|BOLD:AEI4895
BACZP1214-16|Mesocyclops_edax|Canada|BOLD:AEI4895
BACZP1215-16|Mesocyclops_edax|Canada|BOLD:AEI4895
BACZP1216-16|Mesocyclops_edax|Canada|BOLD:AEI4895
BACZP1269-16|Mesocyclops_edax|Canada|BOLD:AEI4895
BACZP1579-16|Mesocyclops_edax|Canada|BOLD:AEI4895
COAPP459-13|Mesocyclops_edax|Canada|BOLD:AEI4895
COAPP511-13|Mesocyclops_edax|Canada|BOLD:AEI4895
SWCHL182-15|Mesocyclops_edax|Canada|BOLD:AEI4895
SWCHL1475-16|Mesocyclops_edax|Canada|BOLD:AEI4895
BACZP1665-16|Mesocyclops_edax|Canada|BOLD:AEI4895
BACZP1209-16|Mesocyclops_edax|Canada|BOLD:AEI4895
BACZP1561-16|Mesocyclops_edax|Canada|BOLD:AEI4895
GBMNE18539-21|Mesocyclops_edax|United_States|BOLD:AEI4895
BACZP855-16|Mesocyclops_edax|Canada|
COAPP486-13|Mesocyclops_edax|Canada|BOLD:AEI4895
COAPP487-13|Mesocyclops_edax|Canada|BOLD:AEI4895
NJNAH017-14|Mesocyclops_edax|Canada|BOLD:AEI4895
NJNAH018-14|Mesocyclops_edax|Canada|BOLD:AEI4895
NJNAH019-14|Mesocyclops_edax|Canada|BOLD:AEI4895
COAPP399-13|Mesocyclops_edax|Canada|BOLD:AEI4895
ZOOPS047-18|Mesocyclops_edax|United_States|BOLD:AEI4895
ZOOPS048-18|Mesocyclops_edax|United_States|BOLD:AEI4895
ZOOPS049-18|Mesocyclops_edax|United_States|BOLD:AEI4895
BBCRU244-12|Cyclops|Canada|BOLD:AEI4894
CAISN926-13|Cyclops|Canada|BOLD:AEI4894
BACZP782-16|Cyclops|Canada|BOLD:AEI4894
ZOOPS706-20|Mesocyclops_edax|Canada|BOLD:AEI4894
SKAAN288-19|Mesocyclops|Mexico|BOLD:AEI4897
EXDB235-20|Cyclopoida|Mexico|
EXDB229-20|Cyclopoida|Mexico|BOLD:AEI6940
ZPII1486-11|Mesocyclops_longisetus|Mexico|BOLD:ABA1199
ZPII1488-11|Mesocyclops_longisetus|Mexico|BOLD:ABA1199
ZPII1489-11|Mesocyclops_longisetus|Mexico|BOLD:ABA1199
ZPLIV402-11|Mesocyclops_longisetus|Mexico|BOLD:ABA1199
ZPLIV404-11|Mesocyclops_longisetus|Mexico|BOLD:ABA1199
ZPLIV405-11|Mesocyclops_longisetus|Mexico|BOLD:ABA1199
SKAAN175-19|Mesocyclops_longisetus|Mexico|BOLD:ABA1199
SKAAN251-19|Mesocyclops_longisetus|Mexico|BOLD:ABA1199
EXDB899-21|Mesocyclops_longisetus|Mexico|BOLD:ABA1199
MCM940-20|Mesocyclops|Mexico|BOLD:ABA1199
MCM941-20|Mesocyclops|Mexico|BOLD:ABA1199
MCM944-20|Mesocyclops|Mexico|BOLD:ABA1199
MCM945-20|Mesocyclops|Mexico|BOLD:ABA1199
MCM946-20|Mesocyclops|Mexico|BOLD:ABA1199
MCM947-20|Mesocyclops|Mexico|BOLD:ABA1199
MCM948-20|Mesocyclops|Mexico|BOLD:ABA1199
MCM949-20|Mesocyclops|Mexico|BOLD:ABA1199
MCM942-20|Mesocyclops|Mexico|BOLD:ABA1199
ZPLIV548-11|Mesocyclops_longisetus|Mexico|BOLD:ABA1206
ZPLIV643-11|Mesocyclops_longisetus|Mexico|BOLD:ABA1206
EXDB1624-21|Mesocyclops_longisetus|Mexico|BOLD:ABA1206
ZPLIV403-11|Mesocyclops_pehpeiensis|Mexico|BOLD:ABA8110
ZMIII858-12|Mesocyclops_pehpeiensis|Mexico|BOLD:ABA8110
ZMIII953-12|Mesocyclops_pehpeiensis|Mexico|BOLD:ABA8110
GBA14358-13|Mesocyclops|Mexico|BOLD:ABA8110
GBA14383-13|Mesocyclops|Mexico|BOLD:ABA8110
ZOOPS222-19|Mesocyclops_pehpeiensis|United_States|BOLD:ABA8110
ZOOPS223-19|Mesocyclops_pehpeiensis|United_States|BOLD:ABA8110
ZOOPS224-19|Mesocyclops_pehpeiensis|United_States|BOLD:ABA8110
ZOOPS220-19|Mesocyclops_pehpeiensis|United_States|BOLD:ABA8110
ZOOPS221-19|Mesocyclops_pehpeiensis|United_States|BOLD:ABA8110
ZPII1002-11|Mesocyclops_edax|Mexico|BOLD:AAZ8505
EXDB876-21|Mesocyclops_edax|Mexico|BOLD:AAZ8505
ZPII1272-11|Mesocyclops_edax|Mexico|BOLD:AAZ8505
ZSYS063-24|Cyclopoida|Mexico|BOLD:AAZ8505
ZSYS064-24|Cyclopoida|Mexico|BOLD:AAZ8505
ZSYS065-24|Cyclopoida|Mexico|BOLD:AAZ8505
ZSYS066-24|Cyclopoida|Mexico|BOLD:AAZ8505
ZSYS067-24|Cyclopoida|Mexico|BOLD:AAZ8505
SKAAN173-19|Cyclopidae|Mexico|BOLD:AEA6624
SKAAN176-19|Mesocyclops_edax|Mexico|
GBCX720-12|Mesocyclops_edax|Mexico|BOLD:AAZ8505
SKAAN147-19|Cyclopoida|Mexico|BOLD:AEA8117
SKAAN246-19|Cyclopoida|Mexico|BOLD:AEA8117
SKAAN247-19|Cyclopoida|Mexico|BOLD:AEA8117
SKAAN873-19|Cyclopoida|Mexico|BOLD:AEA5594
SKAAN918-19|Cyclopoida|Mexico|BOLD:AEB0088
SKAAN919-19|Cyclopoida|Mexico|BOLD:AEB0088
SKAAN920-19|Cyclopoida|Mexico|BOLD:AEB0088
SKAAN921-19|Cyclopoida|Mexico|BOLD:AEB0088
SKAAN907-19|Cyclopoida|Mexico|BOLD:AED8699
SKAAN891-19|Cyclopoida|Mexico|BOLD:AEA9980
GBCM19631-19|Acanthocyclops_vernalis|United_States|BOLD:AEB1166
ZPLVI057-25|Cyclopoida|Mexico|
ZPLVI065-25|Cyclopoida|Mexico|
ZPLVI069-25|Cyclopoida|Mexico|
ZPLIV401-11|Mesocyclops_thermocyclopoides|Mexico|BOLD:ABA6536
ZPLIV724-11|Mesocyclops_thermocyclopoides|Mexico|BOLD:ABA6536
ZPLIV725-11|Mesocyclops_thermocyclopoides|Mexico|BOLD:ABA6536
ZPLIV738-11|Mesocyclops_thermocyclopoides|Mexico|BOLD:ABA6536
ZPLIV740-11|Mesocyclops_thermocyclopoides|Mexico|BOLD:ABA6536
ZMIII951-12|Mesocyclops_thermocyclopoides|Mexico|BOLD:ABA6536
ZMIII952-12|Mesocyclops_thermocyclopoides|Mexico|BOLD:ABA6536
GBA14356-13|Mesocyclops_thermocyclopoides|Mexico|BOLD:ABA6536
GBA14357-13|Mesocyclops_thermocyclopoides|Mexico|BOLD:ABA6536
ZPLIV732-11|Mesocyclops_thermocyclopoides|Mexico|BOLD:ABA6536
BACZP1911-16|Diacyclops_chakan|Mexico|BOLD:ADC6040
BACZP1912-16|Diacyclops_chakan|Mexico|BOLD:ADC6040
BBCRU211-12|Cyclopidae|Canada|BOLD:ACA6820
SWCHL2073-16|Cyclopidae|Canada|BOLD:ACA6820
SWCHL051-15|Cyclopidae|Canada|BOLD:ACA6820
SWCHL2055-16|Cyclopidae|Canada|BOLD:ACA6820
SWCHL1841-16|Cyclopidae|Canada|BOLD:ACA6820
COAPP544-13|Cyclopidae|Canada|BOLD:ACA6820
SWCHL2043-16|Cyclopidae|Canada|BOLD:ACA6820
SWCHL2051-16|Cyclopidae|Canada|BOLD:ACA6820
SWCHL821-16|Cyclopidae|Canada|BOLD:ACA6820
SWCHL825-16|Cyclopidae|Canada|BOLD:ACA6820
SWCHL827-16|Cyclopidae|Canada|BOLD:ACA6820
SWCHL828-16|Cyclopidae|Canada|BOLD:ACA6820
SWCHL851-16|Cyclopidae|Canada|BOLD:ACA6820
SWCHL1063-16|Cyclopidae|Canada|BOLD:ACA6820
SWCHL1064-16|Cyclopidae|Canada|BOLD:ACA6820
SWCHL1065-16|Cyclopidae|Canada|BOLD:ACA6820
SWCHL1067-16|Cyclopidae|Canada|BOLD:ACA6820
SWCHL1068-16|Cyclopidae|Canada|BOLD:ACA6820
SWCHL1070-16|Cyclopidae|Canada|BOLD:ACA6820
SWCHL1074-16|Cyclopidae|Canada|BOLD:ACA6820
SWCHL1080-16|Cyclopidae|Canada|BOLD:ACA6820
SWCHL1081-16|Cyclopidae|Canada|BOLD:ACA6820
SWCHL1084-16|Cyclopidae|Canada|BOLD:ACA6820
SWCHL1260-16|Cyclopidae|Canada|BOLD:ACA6820
SWCHL1261-16|Cyclopidae|Canada|BOLD:ACA6820
SWCHL1265-16|Cyclopidae|Canada|BOLD:ACA6820
SWCHL1269-16|Cyclopidae|Canada|BOLD:ACA6820
SWCHL1272-16|Cyclopidae|Canada|BOLD:ACA6820

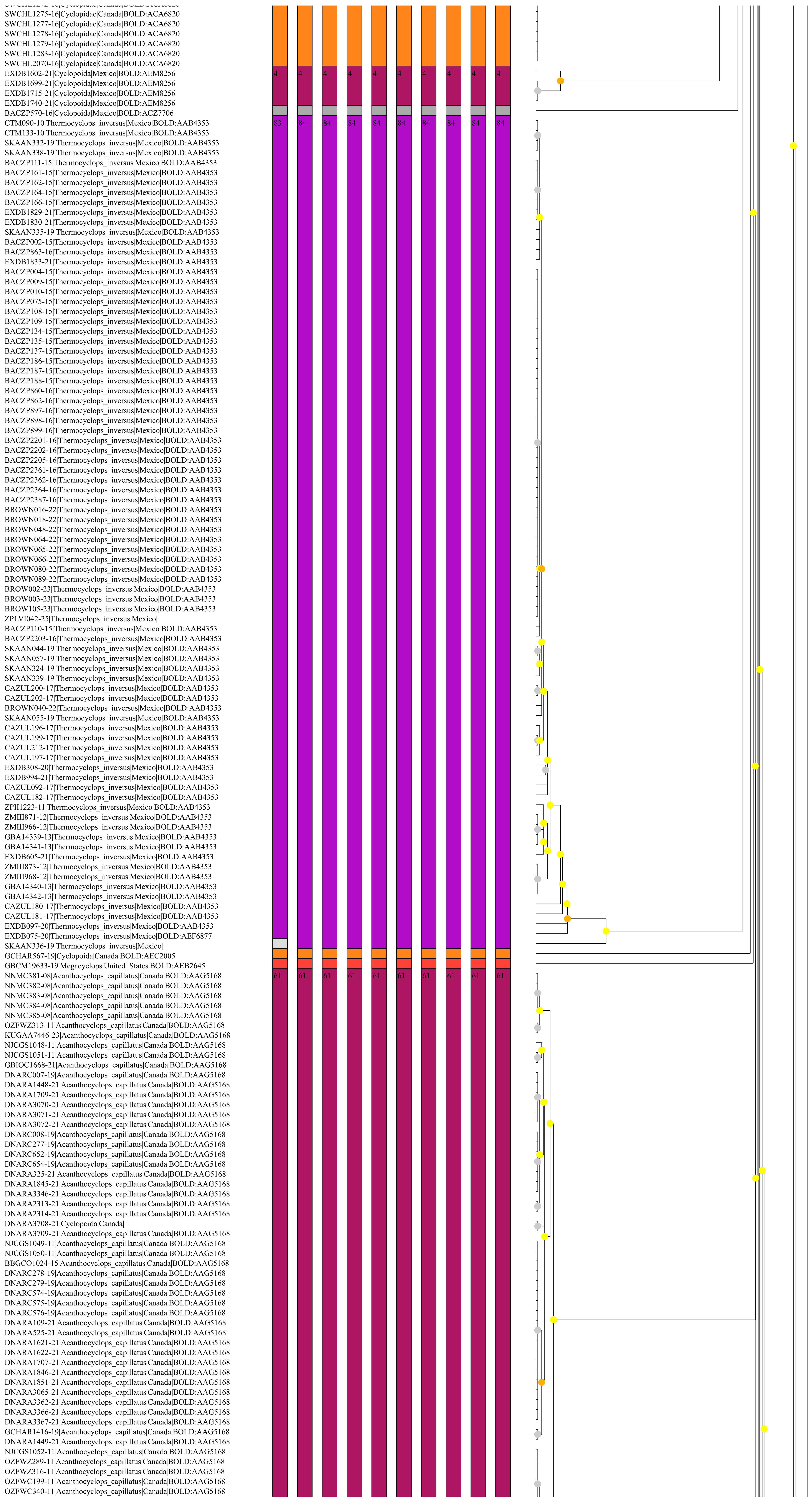
SWCHL1275-16|Cyclopidae|Canada|BOLD:ACA6820
SWCHL1277-16|Cyclopidae|Canada|BOLD:ACA6820
SWCHL1278-16|Cyclopidae|Canada|BOLD:ACA6820
SWCHL1279-16|Cyclopidae|Canada|BOLD:ACA6820
SWCHL1283-16|Cyclopidae|Canada|BOLD:ACA6820
SWCHL2070-16|Cyclopidae|Canada|BOLD:ACA6820
EXDB1602-21|Cyclopoida|Mexico|BOLD:AEM8256
EXDB1699-21|Cyclopoida|Mexico|BOLD:AEM8256
EXDB1715-21|Cyclopoida|Mexico|BOLD:AEM8256
EXDB1740-21|Cyclopoida|Mexico|BOLD:AEM8256
BACZP570-16|Cyclopoida|Mexico|BOLD:ACZ7706
CTM090-10|Thermocyclops_inversus|Mexico|BOLD:AAB4353
CTM133-10|Thermocyclops_inversus|Mexico|BOLD:AAB4353
SKAAN332-19|Thermocyclops_inversus|Mexico|BOLD:AAB4353
SKAAN338-19|Thermocyclops_inversus|Mexico|BOLD:AAB4353
BACZP111-15|Thermocyclops_inversus|Mexico|BOLD:AAB4353
BACZP161-15|Thermocyclops_inversus|Mexico|BOLD:AAB4353
BACZP162-15|Thermocyclops_inversus|Mexico|BOLD:AAB4353
BACZP164-15|Thermocyclops_inversus|Mexico|BOLD:AAB4353
BACZP166-15|Thermocyclops_inversus|Mexico|BOLD:AAB4353
EXDB1829-21|Thermocyclops_inversus|Mexico|BOLD:AAB4353
EXDB1830-21|Thermocyclops_inversus|Mexico|BOLD:AAB4353
SKAAN335-19|Thermocyclops_inversus|Mexico|BOLD:AAB4353
BACZP002-15|Thermocyclops_inversus|Mexico|BOLD:AAB4353
BACZP863-16|Thermocyclops_inversus|Mexico|BOLD:AAB4353
EXDB1833-21|Thermocyclops_inversus|Mexico|BOLD:AAB4353
BACZP004-15|Thermocyclops_inversus|Mexico|BOLD:AAB4353
BACZP009-15|Thermocyclops_inversus|Mexico|BOLD:AAB4353
BACZP010-15|Thermocyclops_inversus|Mexico|BOLD:AAB4353
BACZP075-15|Thermocyclops_inversus|Mexico|BOLD:AAB4353
BACZP108-15|Thermocyclops_inversus|Mexico|BOLD:AAB4353
BACZP109-15|Thermocyclops_inversus|Mexico|BOLD:AAB4353
BACZP134-15|Thermocyclops_inversus|Mexico|BOLD:AAB4353
BACZP135-15|Thermocyclops_inversus|Mexico|BOLD:AAB4353
BACZP137-15|Thermocyclops_inversus|Mexico|BOLD:AAB4353
BACZP186-15|Thermocyclops_inversus|Mexico|BOLD:AAB4353
BACZP187-15|Thermocyclops_inversus|Mexico|BOLD:AAB4353
BACZP188-15|Thermocyclops_inversus|Mexico|BOLD:AAB4353
BACZP860-16|Thermocyclops_inversus|Mexico|BOLD:AAB4353
BACZP862-16|Thermocyclops_inversus|Mexico|BOLD:AAB4353
BACZP897-16|Thermocyclops_inversus|Mexico|BOLD:AAB4353
BACZP898-16|Thermocyclops_inversus|Mexico|BOLD:AAB4353
BACZP899-16|Thermocyclops_inversus|Mexico|BOLD:AAB4353
BACZP2201-16|Thermocyclops_inversus|Mexico|BOLD:AAB4353
BACZP2202-16|Thermocyclops_inversus|Mexico|BOLD:AAB4353
BACZP2205-16|Thermocyclops_inversus|Mexico|BOLD:AAB4353
BACZP2361-16|Thermocyclops_inversus|Mexico|BOLD:AAB4353
BACZP2362-16|Thermocyclops_inversus|Mexico|BOLD:AAB4353
BACZP2364-16|Thermocyclops_inversus|Mexico|BOLD:AAB4353
BACZP2387-16|Thermocyclops_inversus|Mexico|BOLD:AAB4353
BROWN016-22|Thermocyclops_inversus|Mexico|BOLD:AAB4353
BROWN018-22|Thermocyclops_inversus|Mexico|BOLD:AAB4353
BROWN048-22|Thermocyclops_inversus|Mexico|BOLD:AAB4353
BROWN064-22|Thermocyclops_inversus|Mexico|BOLD:AAB4353
BROWN065-22|Thermocyclops_inversus|Mexico|BOLD:AAB4353
BROWN066-22|Thermocyclops_inversus|Mexico|BOLD:AAB4353
BROWN080-22|Thermocyclops_inversus|Mexico|BOLD:AAB4353
BROWN089-22|Thermocyclops_inversus|Mexico|BOLD:AAB4353
BROW002-23|Thermocyclops_inversus|Mexico|BOLD:AAB4353
BROW003-23|Thermocyclops_inversus|Mexico|BOLD:AAB4353
BROW105-23|Thermocyclops_inversus|Mexico|BOLD:AAB4353
ZPLVI042-25|Thermocyclops_inversus|Mexico|
BACZP110-15|Thermocyclops_inversus|Mexico|BOLD:AAB4353
BACZP2203-16|Thermocyclops_inversus|Mexico|BOLD:AAB4353
SKAAN044-19|Thermocyclops_inversus|Mexico|BOLD:AAB4353
SKAAN057-19|Thermocyclops_inversus|Mexico|BOLD:AAB4353
SKAAN324-19|Thermocyclops_inversus|Mexico|BOLD:AAB4353
SKAAN339-19|Thermocyclops_inversus|Mexico|BOLD:AAB4353
CAZUL200-17|Thermocyclops_inversus|Mexico|BOLD:AAB4353
CAZUL202-17|Thermocyclops_inversus|Mexico|BOLD:AAB4353
BROWN040-22|Thermocyclops_inversus|Mexico|BOLD:AAB4353
SKAAN055-19|Thermocyclops_inversus|Mexico|BOLD:AAB4353
CAZUL196-17|Thermocyclops_inversus|Mexico|BOLD:AAB4353
CAZUL199-17|Thermocyclops_inversus|Mexico|BOLD:AAB4353
CAZUL212-17|Thermocyclops_inversus|Mexico|BOLD:AAB4353
CAZUL197-17|Thermocyclops_inversus|Mexico|BOLD:AAB4353
EXDB308-20|Thermocyclops_inversus|Mexico|BOLD:AAB4353
EXDB994-21|Thermocyclops_inversus|Mexico|BOLD:AAB4353
CAZUL092-17|Thermocyclops_inversus|Mexico|BOLD:AAB4353
CAZUL182-17|Thermocyclops_inversus|Mexico|BOLD:AAB4353
ZPII1223-11|Thermocyclops_inversus|Mexico|BOLD:AAB4353
ZMIII871-12|Thermocyclops_inversus|Mexico|BOLD:AAB4353
ZMIII966-12|Thermocyclops_inversus|Mexico|BOLD:AAB4353
GBA14339-13|Thermocyclops_inversus|Mexico|BOLD:AAB4353
GBA14341-13|Thermocyclops_inversus|Mexico|BOLD:AAB4353
EXDB605-21|Thermocyclops_inversus|Mexico|BOLD:AAB4353
ZMIII873-12|Thermocyclops_inversus|Mexico|BOLD:AAB4353
ZMIII968-12|Thermocyclops_inversus|Mexico|BOLD:AAB4353
GBA14340-13|Thermocyclops_inversus|Mexico|BOLD:AAB4353
GBA14342-13|Thermocyclops_inversus|Mexico|BOLD:AAB4353
CAZUL180-17|Thermocyclops_inversus|Mexico|BOLD:AAB4353
CAZUL181-17|Thermocyclops_inversus|Mexico|BOLD:AAB4353
EXDB097-20|Thermocyclops_inversus|Mexico|BOLD:AAB4353
EXDB075-20|Thermocyclops_inversus|Mexico|BOLD:AEF6877
SKAAN336-19|Thermocyclops_inversus|Mexico|
GCHAR567-19|Cyclopoida|Canada|BOLD:AEC2005
GBCM19633-19|Megacyclops|United_States|BOLD:AEB2645
NNMC381-08|Acanthocyclops_capillatus|Canada|BOLD:AAG5168
NNMC382-08|Acanthocyclops_capillatus|Canada|BOLD:AAG5168
NNMC383-08|Acanthocyclops_capillatus|Canada|BOLD:AAG5168
NNMC384-08|Acanthocyclops_capillatus|Canada|BOLD:AAG5168
NNMC385-08|Acanthocyclops_capillatus|Canada|BOLD:AAG5168
OZFWZ313-11|Acanthocyclops_capillatus|Canada|BOLD:AAG5168
KUGAA7446-23|Acanthocyclops_capillatus|Canada|BOLD:AAG5168
NJCGS1048-11|Acanthocyclops_capillatus|Canada|BOLD:AAG5168
NJCGS1051-11|Acanthocyclops_capillatus|Canada|BOLD:AAG5168
GBIOC1668-21|Acanthocyclops_capillatus|Canada|BOLD:AAG5168
DNARC007-19|Acanthocyclops_capillatus|Canada|BOLD:AAG5168
DNARA1448-21|Acanthocyclops_capillatus|Canada|BOLD:AAG5168
DNARA1709-21|Acanthocyclops_capillatus|Canada|BOLD:AAG5168
DNARA3070-21|Acanthocyclops_capillatus|Canada|BOLD:AAG5168
DNARA3071-21|Acanthocyclops_capillatus|Canada|BOLD:AAG5168
DNARA3072-21|Acanthocyclops_capillatus|Canada|BOLD:AAG5168
DNARC008-19|Acanthocyclops_capillatus|Canada|BOLD:AAG5168
DNARC277-19|Acanthocyclops_capillatus|Canada|BOLD:AAG5168
DNARC652-19|Acanthocyclops_capillatus|Canada|BOLD:AAG5168
DNARC654-19|Acanthocyclops_capillatus|Canada|BOLD:AAG5168
DNARA325-21|Acanthocyclops_capillatus|Canada|BOLD:AAG5168
DNARA1845-21|Acanthocyclops_capillatus|Canada|BOLD:AAG5168
DNARA3346-21|Acanthocyclops_capillatus|Canada|BOLD:AAG5168
DNARA2313-21|Acanthocyclops_capillatus|Canada|BOLD:AAG5168
DNARA2314-21|Acanthocyclops_capillatus|Canada|BOLD:AAG5168
DNARA3708-21|Cyclopoida|Canada|
DNARA3709-21|Acanthocyclops_capillatus|Canada|BOLD:AAG5168
NJCGS1049-11|Acanthocyclops_capillatus|Canada|BOLD:AAG5168
NJCGS1050-11|Acanthocyclops_capillatus|Canada|BOLD:AAG5168
BBGCO1024-15|Acanthocyclops_capillatus|Canada|BOLD:AAG5168
DNARC278-19|Acanthocyclops_capillatus|Canada|BOLD:AAG5168
DNARC279-19|Acanthocyclops_capillatus|Canada|BOLD:AAG5168
DNARC574-19|Acanthocyclops_capillatus|Canada|BOLD:AAG5168
DNARC575-19|Acanthocyclops_capillatus|Canada|BOLD:AAG5168
DNARC576-19|Acanthocyclops_capillatus|Canada|BOLD:AAG5168
DNARA109-21|Acanthocyclops_capillatus|Canada|BOLD:AAG5168
DNARA525-21|Acanthocyclops_capillatus|Canada|BOLD:AAG5168
DNARA1621-21|Acanthocyclops_capillatus|Canada|BOLD:AAG5168
DNARA1622-21|Acanthocyclops_capillatus|Canada|BOLD:AAG5168
DNARA1707-21|Acanthocyclops_capillatus|Canada|BOLD:AAG5168
DNARA1846-21|Acanthocyclops_capillatus|Canada|BOLD:AAG5168
DNARA1851-21|Acanthocyclops_capillatus|Canada|BOLD:AAG5168
DNARA3065-21|Acanthocyclops_capillatus|Canada|BOLD:AAG5168
DNARA3362-21|Acanthocyclops_capillatus|Canada|BOLD:AAG5168
DNARA3366-21|Acanthocyclops_capillatus|Canada|BOLD:AAG5168
DNARA3367-21|Acanthocyclops_capillatus|Canada|BOLD:AAG5168
GCHAR1416-19|Acanthocyclops_capillatus|Canada|BOLD:AAG5168
DNARA1449-21|Acanthocyclops_capillatus|Canada|BOLD:AAG5168
NJCGS1052-11|Acanthocyclops_capillatus|Canada|BOLD:AAG5168
OZFWZ289-11|Acanthocyclops_capillatus|Canada|BOLD:AAG5168
OZFWZ316-11|Acanthocyclops_capillatus|Canada|BOLD:AAG5168
OZFWC199-11|Acanthocyclops_capillatus|Canada|BOLD:AAG5168
OZFWC340-11|Acanthocyclops_capillatus|Canada|BOLD:AAG5168
4 4 4 4 4 4 4 4 4 4
83 84 84 84 84 84 84 84 84 84
61 61 61 61 61 61 61 61 61 61

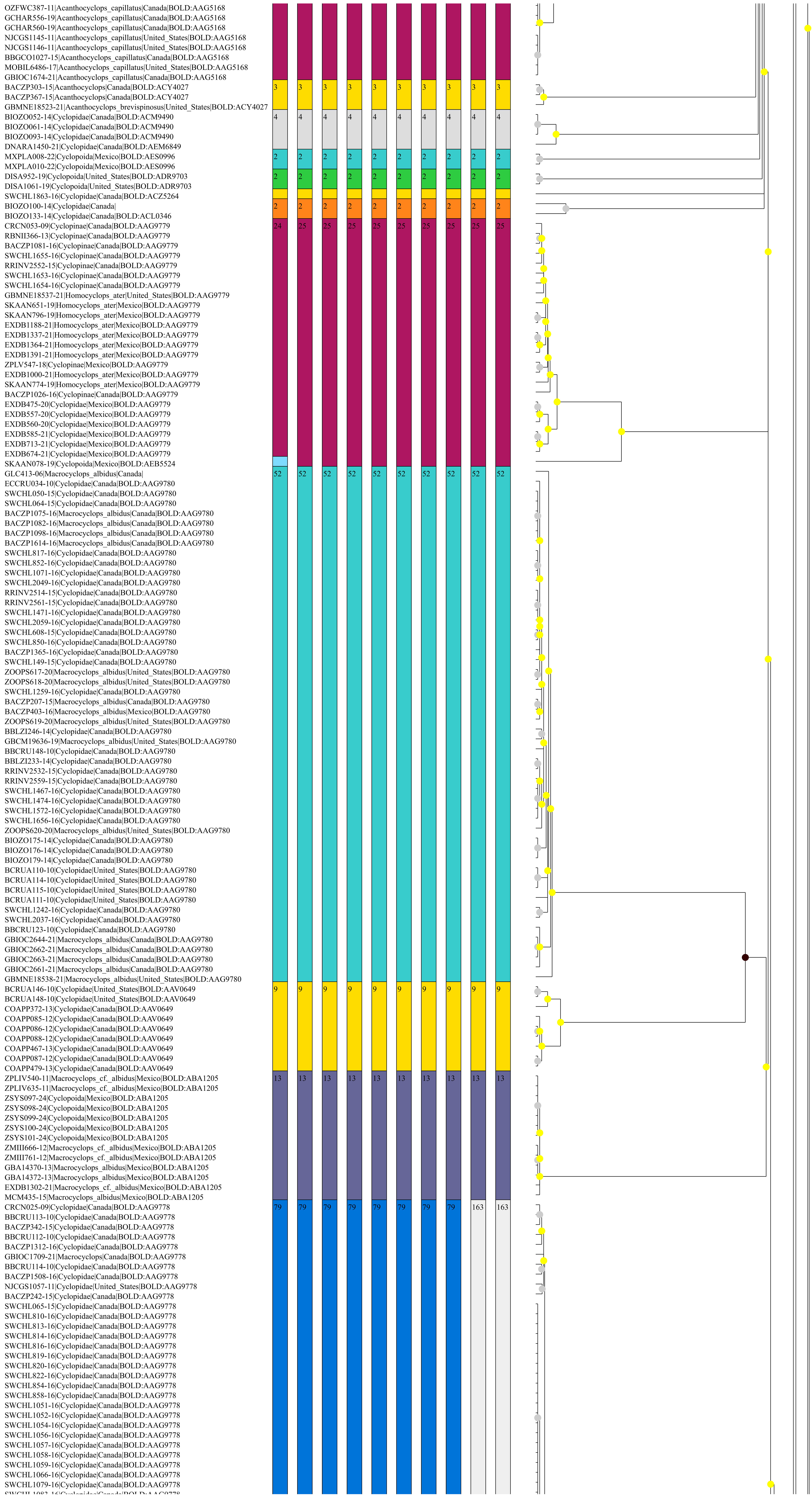
OZFWC387-11|Acanthocyclops_capillatus|Canada|BOLD:AAG5168
GCHAR556-19|Acanthocyclops_capillatus|Canada|BOLD:AAG5168
GCHAR560-19|Acanthocyclops_capillatus|Canada|BOLD:AAG5168
NJCGS1145-11|Acanthocyclops_capillatus|United_States|BOLD:AAG5168
NJCGS1146-11|Acanthocyclops_capillatus|United_States|BOLD:AAG5168
BBGCO1027-15|Acanthocyclops_capillatus|Canada|BOLD:AAG5168
MOBIL6486-17|Acanthocyclops_capillatus|United_States|BOLD:AAG5168
GBIOC1674-21|Acanthocyclops_capillatus|Canada|BOLD:AAG5168
BACZP303-15|Acanthocyclops|Canada|BOLD:ACY4027
BACZP367-15|Acanthocyclops|Canada|BOLD:ACY4027
GBMNE18523-21|Acanthocyclops_brevispinosus|United_States|BOLD:ACY4027
BIOZO052-14|Cyclopidae|Canada|BOLD:ACM9490
BIOZO061-14|Cyclopidae|Canada|BOLD:ACM9490
BIOZO093-14|Cyclopidae|Canada|BOLD:ACM9490
DNARA1450-21|Cyclopidae|Canada|BOLD:AEM6849
MXPLA008-22|Cyclopoida|Mexico|BOLD:AES0996
MXPLA010-22|Cyclopoida|Mexico|BOLD:AES0996
DISA952-19|Cyclopoida|United_States|BOLD:ADR9703
DISA1061-19|Cyclopoida|United_States|BOLD:ADR9703
SWCHL1863-16|Cyclopidae|Canada|BOLD:ACZ5264
BIOZO100-14|Cyclopidae|Canada|
BIOZO133-14|Cyclopidae|Canada|BOLD:ACL0346
CRCN053-09|Cyclopinae|Canada|BOLD:AAG9779
RBNII366-13|Cyclopinae|Canada|BOLD:AAG9779
BACZP1081-16|Cyclopinae|Canada|BOLD:AAG9779
SWCHL1655-16|Cyclopinae|Canada|BOLD:AAG9779
RRINV2552-15|Cyclopinae|Canada|BOLD:AAG9779
SWCHL1653-16|Cyclopinae|Canada|BOLD:AAG9779
SWCHL1654-16|Cyclopinae|Canada|BOLD:AAG9779
GBMNE18537-21|Homocyclops_ater|United_States|BOLD:AAG9779
SKAAN651-19|Homocyclops_ater|Mexico|BOLD:AAG9779
SKAAN796-19|Homocyclops_ater|Mexico|BOLD:AAG9779
EXDB1188-21|Homocyclops_ater|Mexico|BOLD:AAG9779
EXDB1337-21|Homocyclops_ater|Mexico|BOLD:AAG9779
EXDB1364-21|Homocyclops_ater|Mexico|BOLD:AAG9779
EXDB1391-21|Homocyclops_ater|Mexico|BOLD:AAG9779
ZPLV547-18|Cyclopinae|Mexico|BOLD:AAG9779
EXDB1000-21|Homocyclops_ater|Mexico|BOLD:AAG9779
SKAAN774-19|Homocyclops_ater|Mexico|BOLD:AAG9779
BACZP1026-16|Cyclopinae|Canada|BOLD:AAG9779
EXDB475-20|Cyclopidae|Mexico|BOLD:AAG9779
EXDB557-20|Cyclopidae|Mexico|BOLD:AAG9779
EXDB560-20|Cyclopidae|Mexico|BOLD:AAG9779
EXDB585-21|Cyclopidae|Mexico|BOLD:AAG9779
EXDB713-21|Cyclopidae|Mexico|BOLD:AAG9779
EXDB674-21|Cyclopidae|Mexico|BOLD:AAG9779
SKAAN078-19|Cyclopoida|Mexico|BOLD:AEB5524
GLC413-06|Macrocyclops_albidus|Canada|
ECCRU034-10|Cyclopidae|Canada|BOLD:AAG9780
SWCHL050-15|Cyclopidae|Canada|BOLD:AAG9780
SWCHL064-15|Cyclopidae|Canada|BOLD:AAG9780
BACZP1075-16|Macrocyclops_albidus|Canada|BOLD:AAG9780
BACZP1082-16|Macrocyclops_albidus|Canada|BOLD:AAG9780
BACZP1098-16|Macrocyclops_albidus|Canada|BOLD:AAG9780
BACZP1614-16|Macrocyclops_albidus|Canada|BOLD:AAG9780
SWCHL817-16|Cyclopidae|Canada|BOLD:AAG9780
SWCHL852-16|Cyclopidae|Canada|BOLD:AAG9780
SWCHL1071-16|Cyclopidae|Canada|BOLD:AAG9780
SWCHL2049-16|Cyclopidae|Canada|BOLD:AAG9780
RRINV2514-15|Cyclopidae|Canada|BOLD:AAG9780
RRINV2561-15|Cyclopidae|Canada|BOLD:AAG9780
SWCHL1471-16|Cyclopidae|Canada|BOLD:AAG9780
SWCHL2059-16|Cyclopidae|Canada|BOLD:AAG9780
SWCHL608-15|Cyclopidae|Canada|BOLD:AAG9780
SWCHL850-16|Cyclopidae|Canada|BOLD:AAG9780
BACZP1365-16|Cyclopidae|Canada|BOLD:AAG9780
SWCHL149-15|Cyclopidae|Canada|BOLD:AAG9780
ZOOPS617-20|Macrocyclops_albidus|United_States|BOLD:AAG9780
ZOOPS618-20|Macrocyclops_albidus|United_States|BOLD:AAG9780
SWCHL1259-16|Cyclopidae|Canada|BOLD:AAG9780
BACZP207-15|Macrocyclops_albidus|Canada|BOLD:AAG9780
BACZP403-16|Macrocyclops_albidus|Mexico|BOLD:AAG9780
ZOOPS619-20|Macrocyclops_albidus|United_States|BOLD:AAG9780
BBLZI246-14|Cyclopidae|Canada|BOLD:AAG9780
GBCM19636-19|Macrocyclops_albidus|United_States|BOLD:AAG9780
BBCRU148-10|Cyclopidae|Canada|BOLD:AAG9780
BBLZI233-14|Cyclopidae|Canada|BOLD:AAG9780
RRINV2532-15|Cyclopidae|Canada|BOLD:AAG9780
RRINV2559-15|Cyclopidae|Canada|BOLD:AAG9780
SWCHL1467-16|Cyclopidae|Canada|BOLD:AAG9780
SWCHL1474-16|Cyclopidae|Canada|BOLD:AAG9780
SWCHL1572-16|Cyclopidae|Canada|BOLD:AAG9780
SWCHL1656-16|Cyclopidae|Canada|BOLD:AAG9780
ZOOPS620-20|Macrocyclops_albidus|United_States|BOLD:AAG9780
BIOZO175-14|Cyclopidae|Canada|BOLD:AAG9780
BIOZO176-14|Cyclopidae|Canada|BOLD:AAG9780
BIOZO179-14|Cyclopidae|Canada|BOLD:AAG9780
BCRUA110-10|Cyclopidae|United_States|BOLD:AAG9780
BCRUA114-10|Cyclopidae|United_States|BOLD:AAG9780
BCRUA115-10|Cyclopidae|United_States|BOLD:AAG9780
BCRUA111-10|Cyclopidae|United_States|BOLD:AAG9780
SWCHL1242-16|Cyclopidae|Canada|BOLD:AAG9780
SWCHL2037-16|Cyclopidae|Canada|BOLD:AAG9780
BBCRU123-10|Cyclopidae|Canada|BOLD:AAG9780
GBIOC2644-21|Macrocyclops_albidus|Canada|BOLD:AAG9780
GBIOC2662-21|Macrocyclops_albidus|Canada|BOLD:AAG9780
GBIOC2663-21|Macrocyclops_albidus|Canada|BOLD:AAG9780
GBIOC2661-21|Macrocyclops_albidus|Canada|BOLD:AAG9780
GBMNE18538-21|Macrocyclops_albidus|United_States|BOLD:AAG9780
BCRUA146-10|Cyclopidae|United_States|BOLD:AAV0649
BCRUA148-10|Cyclopidae|United_States|BOLD:AAV0649
COAPP372-13|Cyclopidae|Canada|BOLD:AAV0649
COAPP085-12|Cyclopidae|Canada|BOLD:AAV0649
COAPP086-12|Cyclopidae|Canada|BOLD:AAV0649
COAPP088-12|Cyclopidae|Canada|BOLD:AAV0649
COAPP467-13|Cyclopidae|Canada|BOLD:AAV0649
COAPP087-12|Cyclopidae|Canada|BOLD:AAV0649
COAPP479-13|Cyclopidae|Canada|BOLD:AAV0649
ZPLIV540-11|Macrocyclops_cf._albidus|Mexico|BOLD:ABA1205
ZPLIV635-11|Macrocyclops_cf._albidus|Mexico|BOLD:ABA1205
ZSYS097-24|Cyclopoida|Mexico|BOLD:ABA1205
ZSYS098-24|Cyclopoida|Mexico|BOLD:ABA1205
ZSYS099-24|Cyclopoida|Mexico|BOLD:ABA1205
ZSYS100-24|Cyclopoida|Mexico|BOLD:ABA1205
ZSYS101-24|Cyclopoida|Mexico|BOLD:ABA1205
ZMIII666-12|Macrocyclops_cf._albidus|Mexico|BOLD:ABA1205
ZMIII761-12|Macrocyclops_cf._albidus|Mexico|BOLD:ABA1205
GBA14370-13|Macrocyclops_albidus|Mexico|BOLD:ABA1205
GBA14372-13|Macrocyclops_albidus|Mexico|BOLD:ABA1205
EXDB1302-21|Macrocyclops_cf._albidus|Mexico|BOLD:ABA1205
MCM435-15|Macrocyclops_albidus|Mexico|BOLD:ABA1205
CRCN025-09|Cyclopidae|Canada|BOLD:AAG9778
BBCRU113-10|Cyclopidae|Canada|BOLD:AAG9778
BACZP342-15|Cyclopidae|Canada|BOLD:AAG9778
BBCRU112-10|Cyclopidae|Canada|BOLD:AAG9778
BACZP1312-16|Cyclopidae|Canada|BOLD:AAG9778
GBIOC1709-21|Macrocyclops|Canada|BOLD:AAG9778
BBCRU114-10|Cyclopidae|Canada|BOLD:AAG9778
BACZP1508-16|Cyclopidae|Canada|BOLD:AAG9778
NJCGS1057-11|Cyclopidae|United_States|BOLD:AAG9778
BACZP242-15|Cyclopidae|Canada|BOLD:AAG9778
SWCHL065-15|Cyclopidae|Canada|BOLD:AAG9778
SWCHL810-16|Cyclopidae|Canada|BOLD:AAG9778
SWCHL813-16|Cyclopidae|Canada|BOLD:AAG9778
SWCHL814-16|Cyclopidae|Canada|BOLD:AAG9778
SWCHL816-16|Cyclopidae|Canada|BOLD:AAG9778
SWCHL819-16|Cyclopidae|Canada|BOLD:AAG9778
SWCHL820-16|Cyclopidae|Canada|BOLD:AAG9778
SWCHL822-16|Cyclopidae|Canada|BOLD:AAG9778
SWCHL854-16|Cyclopidae|Canada|BOLD:AAG9778
SWCHL858-16|Cyclopidae|Canada|BOLD:AAG9778
SWCHL1051-16|Cyclopidae|Canada|BOLD:AAG9778
SWCHL1052-16|Cyclopidae|Canada|BOLD:AAG9778
SWCHL1054-16|Cyclopidae|Canada|BOLD:AAG9778
SWCHL1056-16|Cyclopidae|Canada|BOLD:AAG9778
SWCHL1057-16|Cyclopidae|Canada|BOLD:AAG9778
SWCHL1058-16|Cyclopidae|Canada|BOLD:AAG9778
SWCHL1059-16|Cyclopidae|Canada|BOLD:AAG9778
SWCHL1066-16|Cyclopidae|Canada|BOLD:AAG9778
SWCHL1079-16|Cyclopidae|Canada|BOLD:AAG9778
3
4
2
2
2
24
25
52
9
13
79
163

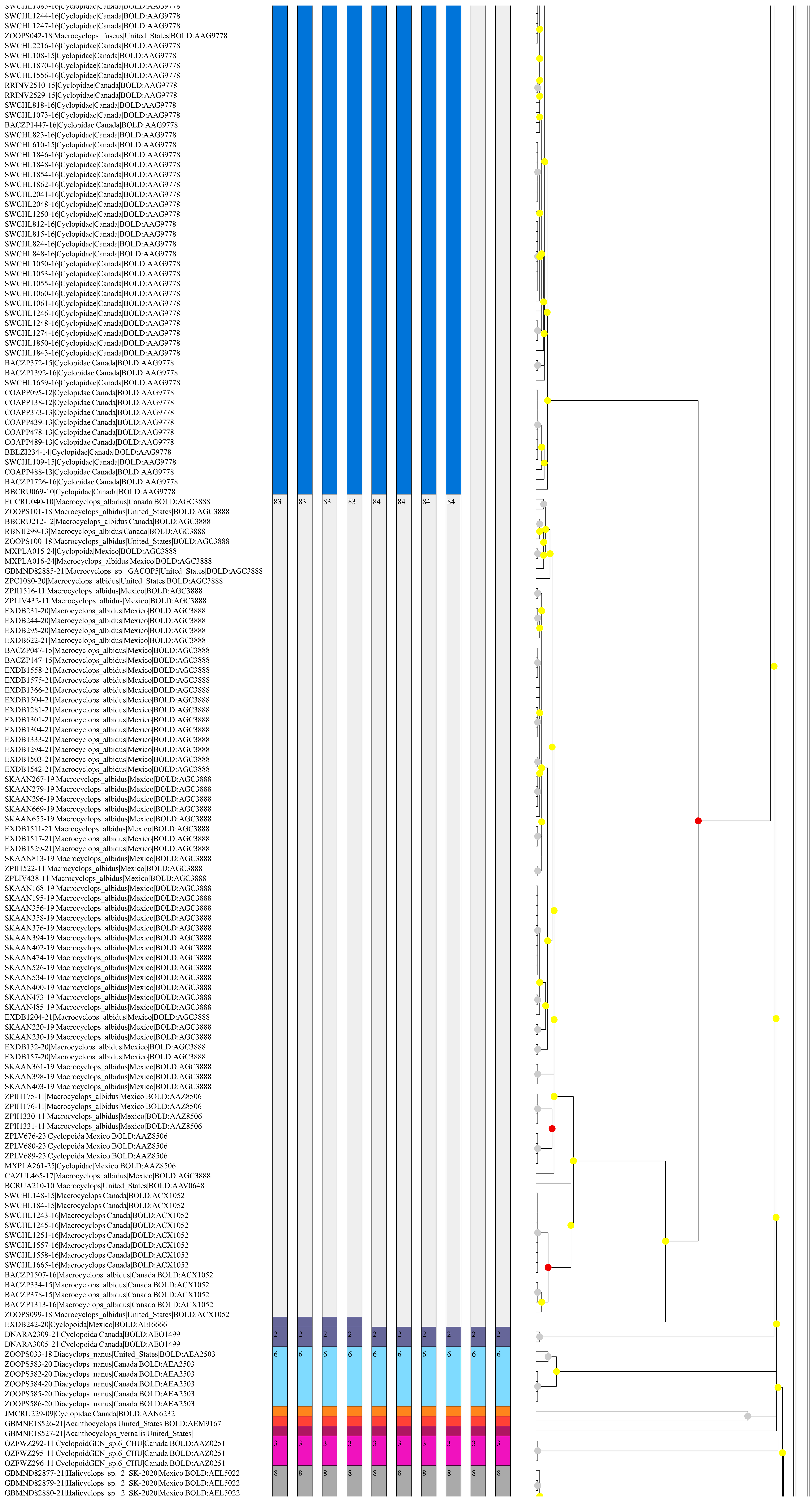
SWCHL1063-16|Cyclopidae|Canada|BOLD:AAG9778
SWCHL1244-16|Cyclopidae|Canada|BOLD:AAG9778
SWCHL1247-16|Cyclopidae|Canada|BOLD:AAG9778
ZOOPS042-18|Macrocyclops_fuscus|United_States|BOLD:AAG9778
SWCHL2216-16|Cyclopidae|Canada|BOLD:AAG9778
SWCHL108-15|Cyclopidae|Canada|BOLD:AAG9778
SWCHL1870-16|Cyclopidae|Canada|BOLD:AAG9778
SWCHL1556-16|Cyclopidae|Canada|BOLD:AAG9778
RRINV2510-15|Cyclopidae|Canada|BOLD:AAG9778
RRINV2529-15|Cyclopidae|Canada|BOLD:AAG9778
SWCHL818-16|Cyclopidae|Canada|BOLD:AAG9778
SWCHL1073-16|Cyclopidae|Canada|BOLD:AAG9778
BACZP1447-16|Cyclopidae|Canada|BOLD:AAG9778
SWCHL823-16|Cyclopidae|Canada|BOLD:AAG9778
SWCHL610-15|Cyclopidae|Canada|BOLD:AAG9778
SWCHL1846-16|Cyclopidae|Canada|BOLD:AAG9778
SWCHL1848-16|Cyclopidae|Canada|BOLD:AAG9778
SWCHL1854-16|Cyclopidae|Canada|BOLD:AAG9778
SWCHL1862-16|Cyclopidae|Canada|BOLD:AAG9778
SWCHL2041-16|Cyclopidae|Canada|BOLD:AAG9778
SWCHL2048-16|Cyclopidae|Canada|BOLD:AAG9778
SWCHL1250-16|Cyclopidae|Canada|BOLD:AAG9778
SWCHL812-16|Cyclopidae|Canada|BOLD:AAG9778
SWCHL815-16|Cyclopidae|Canada|BOLD:AAG9778
SWCHL824-16|Cyclopidae|Canada|BOLD:AAG9778
SWCHL848-16|Cyclopidae|Canada|BOLD:AAG9778
SWCHL1050-16|Cyclopidae|Canada|BOLD:AAG9778
SWCHL1053-16|Cyclopidae|Canada|BOLD:AAG9778
SWCHL1055-16|Cyclopidae|Canada|BOLD:AAG9778
SWCHL1060-16|Cyclopidae|Canada|BOLD:AAG9778
SWCHL1061-16|Cyclopidae|Canada|BOLD:AAG9778
SWCHL1246-16|Cyclopidae|Canada|BOLD:AAG9778
SWCHL1248-16|Cyclopidae|Canada|BOLD:AAG9778
SWCHL1274-16|Cyclopidae|Canada|BOLD:AAG9778
SWCHL1850-16|Cyclopidae|Canada|BOLD:AAG9778
SWCHL1843-16|Cyclopidae|Canada|BOLD:AAG9778
BACZP372-15|Cyclopidae|Canada|BOLD:AAG9778
BACZP1392-16|Cyclopidae|Canada|BOLD:AAG9778
SWCHL1659-16|Cyclopidae|Canada|BOLD:AAG9778
COAPP095-12|Cyclopidae|Canada|BOLD:AAG9778
COAPP138-12|Cyclopidae|Canada|BOLD:AAG9778
COAPP373-13|Cyclopidae|Canada|BOLD:AAG9778
COAPP439-13|Cyclopidae|Canada|BOLD:AAG9778
COAPP478-13|Cyclopidae|Canada|BOLD:AAG9778
COAPP489-13|Cyclopidae|Canada|BOLD:AAG9778
BBLZI234-14|Cyclopidae|Canada|BOLD:AAG9778
SWCHL109-15|Cyclopidae|Canada|BOLD:AAG9778
COAPP488-13|Cyclopidae|Canada|BOLD:AAG9778
BACZP1726-16|Cyclopidae|Canada|BOLD:AAG9778
BBCRU069-10|Cyclopidae|Canada|BOLD:AAG9778
ECCRU040-10|Macrocyclops_albidus|Canada|BOLD:AGC3888
ZOOPS101-18|Macrocyclops_albidus|United_States|BOLD:AGC3888
BBCRU212-12|Macrocyclops_albidus|Canada|BOLD:AGC3888
RBNII299-13|Macrocyclops_albidus|Canada|BOLD:AGC3888
ZOOPS100-18|Macrocyclops_albidus|United_States|BOLD:AGC3888
MXPLA015-24|Cyclopoida|Mexico|BOLD:AGC3888
MXPLA016-24|Macrocyclops_albidus|Mexico|BOLD:AGC3888
GBMND82885-21|Macrocyclops_sp._GACOP5|United_States|BOLD:AGC3888
ZPC1080-20|Macrocyclops_albidus|United_States|BOLD:AGC3888
ZPII1516-11|Macrocyclops_albidus|Mexico|BOLD:AGC3888
ZPLIV432-11|Macrocyclops_albidus|Mexico|BOLD:AGC3888
EXDB231-20|Macrocyclops_albidus|Mexico|BOLD:AGC3888
EXDB244-20|Macrocyclops_albidus|Mexico|BOLD:AGC3888
EXDB295-20|Macrocyclops_albidus|Mexico|BOLD:AGC3888
EXDB622-21|Macrocyclops_albidus|Mexico|BOLD:AGC3888
BACZP047-15|Macrocyclops_albidus|Mexico|BOLD:AGC3888
BACZP147-15|Macrocyclops_albidus|Mexico|BOLD:AGC3888
EXDB1558-21|Macrocyclops_albidus|Mexico|BOLD:AGC3888
EXDB1575-21|Macrocyclops_albidus|Mexico|BOLD:AGC3888
EXDB1366-21|Macrocyclops_albidus|Mexico|BOLD:AGC3888
EXDB1504-21|Macrocyclops_albidus|Mexico|BOLD:AGC3888
EXDB1281-21|Macrocyclops_albidus|Mexico|BOLD:AGC3888
EXDB1301-21|Macrocyclops_albidus|Mexico|BOLD:AGC3888
EXDB1304-21|Macrocyclops_albidus|Mexico|BOLD:AGC3888
EXDB1333-21|Macrocyclops_albidus|Mexico|BOLD:AGC3888
EXDB1294-21|Macrocyclops_albidus|Mexico|BOLD:AGC3888
EXDB1503-21|Macrocyclops_albidus|Mexico|BOLD:AGC3888
EXDB1542-21|Macrocyclops_albidus|Mexico|BOLD:AGC3888
SKAAN267-19|Macrocyclops_albidus|Mexico|BOLD:AGC3888
SKAAN279-19|Macrocyclops_albidus|Mexico|BOLD:AGC3888
SKAAN296-19|Macrocyclops_albidus|Mexico|BOLD:AGC3888
SKAAN669-19|Macrocyclops_albidus|Mexico|BOLD:AGC3888
SKAAN655-19|Macrocyclops_albidus|Mexico|BOLD:AGC3888
EXDB1511-21|Macrocyclops_albidus|Mexico|BOLD:AGC3888
EXDB1517-21|Macrocyclops_albidus|Mexico|BOLD:AGC3888
EXDB1529-21|Macrocyclops_albidus|Mexico|BOLD:AGC3888
SKAAN813-19|Macrocyclops_albidus|Mexico|BOLD:AGC3888
ZPII1522-11|Macrocyclops_albidus|Mexico|BOLD:AGC3888
ZPLIV438-11|Macrocyclops_albidus|Mexico|BOLD:AGC3888
SKAAN168-19|Macrocyclops_albidus|Mexico|BOLD:AGC3888
SKAAN195-19|Macrocyclops_albidus|Mexico|BOLD:AGC3888
SKAAN356-19|Macrocyclops_albidus|Mexico|BOLD:AGC3888
SKAAN358-19|Macrocyclops_albidus|Mexico|BOLD:AGC3888
SKAAN376-19|Macrocyclops_albidus|Mexico|BOLD:AGC3888
SKAAN394-19|Macrocyclops_albidus|Mexico|BOLD:AGC3888
SKAAN402-19|Macrocyclops_albidus|Mexico|BOLD:AGC3888
SKAAN474-19|Macrocyclops_albidus|Mexico|BOLD:AGC3888
SKAAN526-19|Macrocyclops_albidus|Mexico|BOLD:AGC3888
SKAAN534-19|Macrocyclops_albidus|Mexico|BOLD:AGC3888
SKAAN400-19|Macrocyclops_albidus|Mexico|BOLD:AGC3888
SKAAN473-19|Macrocyclops_albidus|Mexico|BOLD:AGC3888
SKAAN485-19|Macrocyclops_albidus|Mexico|BOLD:AGC3888
EXDB1204-21|Macrocyclops_albidus|Mexico|BOLD:AGC3888
SKAAN220-19|Macrocyclops_albidus|Mexico|BOLD:AGC3888
SKAAN230-19|Macrocyclops_albidus|Mexico|BOLD:AGC3888
EXDB132-20|Macrocyclops_albidus|Mexico|BOLD:AGC3888
EXDB157-20|Macrocyclops_albidus|Mexico|BOLD:AGC3888
SKAAN361-19|Macrocyclops_albidus|Mexico|BOLD:AGC3888
SKAAN398-19|Macrocyclops_albidus|Mexico|BOLD:AGC3888
SKAAN403-19|Macrocyclops_albidus|Mexico|BOLD:AGC3888
ZPII1175-11|Macrocyclops_albidus|Mexico|BOLD:AAZ8506
ZPII1176-11|Macrocyclops_albidus|Mexico|BOLD:AAZ8506
ZPII1330-11|Macrocyclops_albidus|Mexico|BOLD:AAZ8506
ZPII1331-11|Macrocyclops_albidus|Mexico|BOLD:AAZ8506
ZPLV676-23|Cyclopoida|Mexico|BOLD:AAZ8506
ZPLV680-23|Cyclopoida|Mexico|BOLD:AAZ8506
ZPLV689-23|Cyclopoida|Mexico|BOLD:AAZ8506
MXPLA261-25|Cyclopidae|Mexico|BOLD:AAZ8506
CAZUL465-17|Macrocyclops_albidus|Mexico|BOLD:AGC3888
BCRUA210-10|Macrocyclops|United_States|BOLD:AAV0648
SWCHL148-15|Macrocyclops|Canada|BOLD:ACX1052
SWCHL184-15|Macrocyclops|Canada|BOLD:ACX1052
SWCHL1243-16|Macrocyclops|Canada|BOLD:ACX1052
SWCHL1245-16|Macrocyclops|Canada|BOLD:ACX1052
SWCHL1251-16|Macrocyclops|Canada|BOLD:ACX1052
SWCHL1557-16|Macrocyclops|Canada|BOLD:ACX1052
SWCHL1558-16|Macrocyclops|Canada|BOLD:ACX1052
SWCHL1665-16|Macrocyclops|Canada|BOLD:ACX1052
BACZP1507-16|Macrocyclops_albidus|Canada|BOLD:ACX1052
BACZP334-15|Macrocyclops_albidus|Canada|BOLD:ACX1052
BACZP378-15|Macrocyclops_albidus|Canada|BOLD:ACX1052
BACZP1313-16|Macrocyclops_albidus|Canada|BOLD:ACX1052
ZOOPS099-18|Macrocyclops_albidus|United_States|BOLD:ACX1052
EXDB242-20|Cyclopoida|Mexico|BOLD:AEI6666
DNARA2309-21|Cyclopoida|Canada|BOLD:AEO1499
DNARA3005-21|Cyclopoida|Canada|BOLD:AEO1499
ZOOPS033-18|Diacyclops_nanus|United_States|BOLD:AEA2503
ZOOPS583-20|Diacyclops_nanus|Canada|BOLD:AEA2503
ZOOPS582-20|Diacyclops_nanus|Canada|BOLD:AEA2503
ZOOPS584-20|Diacyclops_nanus|Canada|BOLD:AEA2503
ZOOPS585-20|Diacyclops_nanus|Canada|BOLD:AEA2503
ZOOPS586-20|Diacyclops_nanus|Canada|BOLD:AEA2503
JMCRU229-09|Cyclopidae|Canada|BOLD:AAN6232
GBMNE18526-21|Acanthocyclops|United_States|BOLD:AEM9167
GBMNE18527-21|Acanthocyclops_vernalis|United_States|
OZFWZ292-11|CyclopoidGEN_sp.6_CHU|Canada|BOLD:AAZ0251
OZFWZ295-11|CyclopoidGEN_sp.6_CHU|Canada|BOLD:AAZ0251
OZFWZ296-11|CyclopoidGEN_sp.6_CHU|Canada|BOLD:AAZ0251
GBMND82877-21|Halicyclops_sp._2_SK-2020|Mexico|BOLD:AEL5022
GBMND82879-21|Halicyclops_sp._2_SK-2020|Mexico|BOLD:AEL5022
GBMND82880-21|Halicyclops_sp._2_SK-2020|Mexico|BOLD:AEL5022
83 83 83 83 84 84 84 84
2 2 2 2 2 2 2 2 2 2
6 6 6 6 6 6 6 6 6 6
3 3 3 3 3 3 3 3 3 3
8 8 8 8 8 8 8 8 8 8

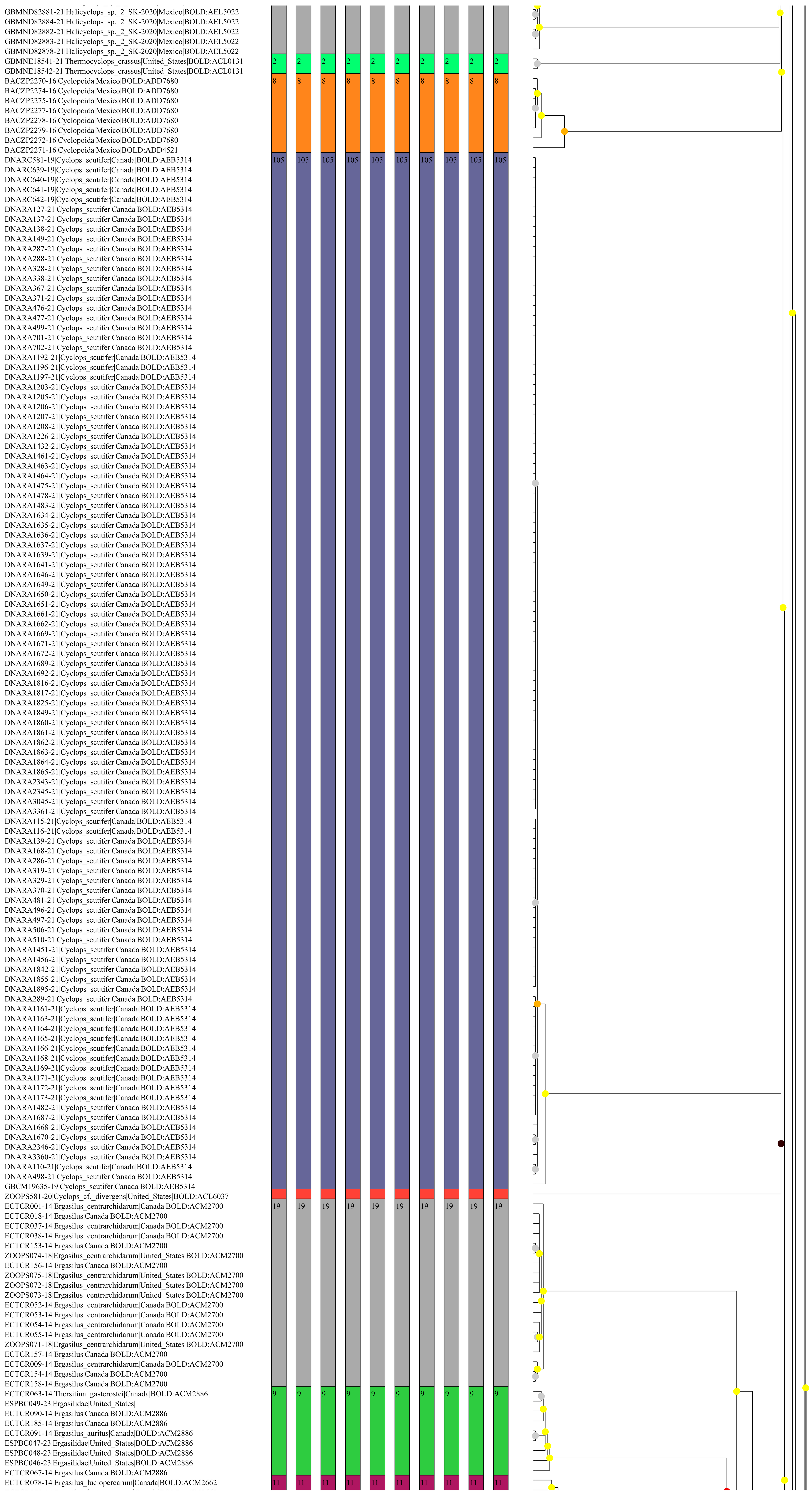

GBMND82881-21|Halicyclops_sp._2_SK-2020|Mexico|BOLD:AEL5022
GBMND82884-21|Halicyclops_sp._2_SK-2020|Mexico|BOLD:AEL5022
GBMND82882-21|Halicyclops_sp._2_SK-2020|Mexico|BOLD:AEL5022
GBMND82883-21|Halicyclops_sp._2_SK-2020|Mexico|BOLD:AEL5022
GBMND82878-21|Halicyclops_sp._2_SK-2020|Mexico|BOLD:AEL5022
GBMNE18541-21|Thermocyclops_crassus|United_States|BOLD:ACL0131
GBMNE18542-21|Thermocyclops_crassus|United_States|BOLD:ACL0131
BACZP2270-16|Cyclopoida|Mexico|BOLD:ADD7680
BACZP2274-16|Cyclopoida|Mexico|BOLD:ADD7680
BACZP2275-16|Cyclopoida|Mexico|BOLD:ADD7680
BACZP2277-16|Cyclopoida|Mexico|BOLD:ADD7680
BACZP2278-16|Cyclopoida|Mexico|BOLD:ADD7680
BACZP2279-16|Cyclopoida|Mexico|BOLD:ADD7680
BACZP2272-16|Cyclopoida|Mexico|BOLD:ADD7680
BACZP2271-16|Cyclopoida|Mexico|BOLD:ADD4521
DNARC581-19|Cyclops_scutifer|Canada|BOLD:AEB5314
DNARC639-19|Cyclops_scutifer|Canada|BOLD:AEB5314
DNARC640-19|Cyclops_scutifer|Canada|BOLD:AEB5314
DNARC641-19|Cyclops_scutifer|Canada|BOLD:AEB5314
DNARC642-19|Cyclops_scutifer|Canada|BOLD:AEB5314
DNARA127-21|Cyclops_scutifer|Canada|BOLD:AEB5314
DNARA137-21|Cyclops_scutifer|Canada|BOLD:AEB5314
DNARA138-21|Cyclops_scutifer|Canada|BOLD:AEB5314
DNARA149-21|Cyclops_scutifer|Canada|BOLD:AEB5314
DNARA287-21|Cyclops_scutifer|Canada|BOLD:AEB5314
DNARA288-21|Cyclops_scutifer|Canada|BOLD:AEB5314
DNARA328-21|Cyclops_scutifer|Canada|BOLD:AEB5314
DNARA338-21|Cyclops_scutifer|Canada|BOLD:AEB5314
DNARA367-21|Cyclops_scutifer|Canada|BOLD:AEB5314
DNARA371-21|Cyclops_scutifer|Canada|BOLD:AEB5314
DNARA476-21|Cyclops_scutifer|Canada|BOLD:AEB5314
DNARA477-21|Cyclops_scutifer|Canada|BOLD:AEB5314
DNARA499-21|Cyclops_scutifer|Canada|BOLD:AEB5314
DNARA701-21|Cyclops_scutifer|Canada|BOLD:AEB5314
DNARA702-21|Cyclops_scutifer|Canada|BOLD:AEB5314
DNARA1192-21|Cyclops_scutifer|Canada|BOLD:AEB5314
DNARA1196-21|Cyclops_scutifer|Canada|BOLD:AEB5314
DNARA1197-21|Cyclops_scutifer|Canada|BOLD:AEB5314
DNARA1203-21|Cyclops_scutifer|Canada|BOLD:AEB5314
DNARA1205-21|Cyclops_scutifer|Canada|BOLD:AEB5314
DNARA1206-21|Cyclops_scutifer|Canada|BOLD:AEB5314
DNARA1207-21|Cyclops_scutifer|Canada|BOLD:AEB5314
DNARA1208-21|Cyclops_scutifer|Canada|BOLD:AEB5314
DNARA1226-21|Cyclops_scutifer|Canada|BOLD:AEB5314
DNARA1432-21|Cyclops_scutifer|Canada|BOLD:AEB5314
DNARA1461-21|Cyclops_scutifer|Canada|BOLD:AEB5314
DNARA1463-21|Cyclops_scutifer|Canada|BOLD:AEB5314
DNARA1464-21|Cyclops_scutifer|Canada|BOLD:AEB5314
DNARA1475-21|Cyclops_scutifer|Canada|BOLD:AEB5314
DNARA1478-21|Cyclops_scutifer|Canada|BOLD:AEB5314
DNARA1483-21|Cyclops_scutifer|Canada|BOLD:AEB5314
DNARA1634-21|Cyclops_scutifer|Canada|BOLD:AEB5314
DNARA1635-21|Cyclops_scutifer|Canada|BOLD:AEB5314
DNARA1636-21|Cyclops_scutifer|Canada|BOLD:AEB5314
DNARA1637-21|Cyclops_scutifer|Canada|BOLD:AEB5314
DNARA1639-21|Cyclops_scutifer|Canada|BOLD:AEB5314
DNARA1641-21|Cyclops_scutifer|Canada|BOLD:AEB5314
DNARA1646-21|Cyclops_scutifer|Canada|BOLD:AEB5314
DNARA1649-21|Cyclops_scutifer|Canada|BOLD:AEB5314
DNARA1650-21|Cyclops_scutifer|Canada|BOLD:AEB5314
DNARA1651-21|Cyclops_scutifer|Canada|BOLD:AEB5314
DNARA1661-21|Cyclops_scutifer|Canada|BOLD:AEB5314
DNARA1662-21|Cyclops_scutifer|Canada|BOLD:AEB5314
DNARA1669-21|Cyclops_scutifer|Canada|BOLD:AEB5314
DNARA1671-21|Cyclops_scutifer|Canada|BOLD:AEB5314
DNARA1672-21|Cyclops_scutifer|Canada|BOLD:AEB5314
DNARA1689-21|Cyclops_scutifer|Canada|BOLD:AEB5314
DNARA1692-21|Cyclops_scutifer|Canada|BOLD:AEB5314
DNARA1816-21|Cyclops_scutifer|Canada|BOLD:AEB5314
DNARA1817-21|Cyclops_scutifer|Canada|BOLD:AEB5314
DNARA1825-21|Cyclops_scutifer|Canada|BOLD:AEB5314
DNARA1849-21|Cyclops_scutifer|Canada|BOLD:AEB5314
DNARA1860-21|Cyclops_scutifer|Canada|BOLD:AEB5314
DNARA1861-21|Cyclops_scutifer|Canada|BOLD:AEB5314
DNARA1862-21|Cyclops_scutifer|Canada|BOLD:AEB5314
DNARA1863-21|Cyclops_scutifer|Canada|BOLD:AEB5314
DNARA1864-21|Cyclops_scutifer|Canada|BOLD:AEB5314
DNARA1865-21|Cyclops_scutifer|Canada|BOLD:AEB5314
DNARA2343-21|Cyclops_scutifer|Canada|BOLD:AEB5314
DNARA2345-21|Cyclops_scutifer|Canada|BOLD:AEB5314
DNARA3045-21|Cyclops_scutifer|Canada|BOLD:AEB5314
DNARA3361-21|Cyclops_scutifer|Canada|BOLD:AEB5314
DNARA115-21|Cyclops_scutifer|Canada|BOLD:AEB5314
DNARA116-21|Cyclops_scutifer|Canada|BOLD:AEB5314
DNARA139-21|Cyclops_scutifer|Canada|BOLD:AEB5314
DNARA168-21|Cyclops_scutifer|Canada|BOLD:AEB5314
DNARA286-21|Cyclops_scutifer|Canada|BOLD:AEB5314
DNARA319-21|Cyclops_scutifer|Canada|BOLD:AEB5314
DNARA329-21|Cyclops_scutifer|Canada|BOLD:AEB5314
DNARA370-21|Cyclops_scutifer|Canada|BOLD:AEB5314
DNARA481-21|Cyclops_scutifer|Canada|BOLD:AEB5314
DNARA496-21|Cyclops_scutifer|Canada|BOLD:AEB5314
DNARA497-21|Cyclops_scutifer|Canada|BOLD:AEB5314
DNARA506-21|Cyclops_scutifer|Canada|BOLD:AEB5314
DNARA510-21|Cyclops_scutifer|Canada|BOLD:AEB5314
DNARA1451-21|Cyclops_scutifer|Canada|BOLD:AEB5314
DNARA1456-21|Cyclops_scutifer|Canada|BOLD:AEB5314
DNARA1842-21|Cyclops_scutifer|Canada|BOLD:AEB5314
DNARA1855-21|Cyclops_scutifer|Canada|BOLD:AEB5314
DNARA1895-21|Cyclops_scutifer|Canada|BOLD:AEB5314
DNARA289-21|Cyclops_scutifer|Canada|BOLD:AEB5314
DNARA1161-21|Cyclops_scutifer|Canada|BOLD:AEB5314
DNARA1163-21|Cyclops_scutifer|Canada|BOLD:AEB5314
DNARA1164-21|Cyclops_scutifer|Canada|BOLD:AEB5314
DNARA1165-21|Cyclops_scutifer|Canada|BOLD:AEB5314
DNARA1166-21|Cyclops_scutifer|Canada|BOLD:AEB5314
DNARA1168-21|Cyclops_scutifer|Canada|BOLD:AEB5314
DNARA1169-21|Cyclops_scutifer|Canada|BOLD:AEB5314
DNARA1171-21|Cyclops_scutifer|Canada|BOLD:AEB5314
DNARA1172-21|Cyclops_scutifer|Canada|BOLD:AEB5314
DNARA1173-21|Cyclops_scutifer|Canada|BOLD:AEB5314
DNARA1482-21|Cyclops_scutifer|Canada|BOLD:AEB5314
DNARA1687-21|Cyclops_scutifer|Canada|BOLD:AEB5314
DNARA1668-21|Cyclops_scutifer|Canada|BOLD:AEB5314
DNARA1670-21|Cyclops_scutifer|Canada|BOLD:AEB5314
DNARA2346-21|Cyclops_scutifer|Canada|BOLD:AEB5314
DNARA3360-21|Cyclops_scutifer|Canada|BOLD:AEB5314
DNARA110-21|Cyclops_scutifer|Canada|BOLD:AEB5314
DNARA498-21|Cyclops_scutifer|Canada|BOLD:AEB5314
GBCM19635-19|Cyclops_scutifer|Canada|BOLD:AEB5314
ZOOPS581-20|Cyclops_cf._divergens|United_States|BOLD:ACL6037
ECTCR001-14|Ergasilus_centrarchidarum|Canada|BOLD:ACM2700
ECTCR018-14|Ergasilus|Canada|BOLD:ACM2700
ECTCR037-14|Ergasilus_centrarchidarum|Canada|BOLD:ACM2700
ECTCR038-14|Ergasilus_centrarchidarum|Canada|BOLD:ACM2700
ECTCR153-14|Ergasilus|Canada|BOLD:ACM2700
ZOOPS074-18|Ergasilus_centrarchidarum|United_States|BOLD:ACM2700
ECTCR156-14|Ergasilus|Canada|BOLD:ACM2700
ZOOPS075-18|Ergasilus_centrarchidarum|United_States|BOLD:ACM2700
ZOOPS072-18|Ergasilus_centrarchidarum|United_States|BOLD:ACM2700
ZOOPS073-18|Ergasilus_centrarchidarum|United_States|BOLD:ACM2700
ECTCR052-14|Ergasilus_centrarchidarum|Canada|BOLD:ACM2700
ECTCR053-14|Ergasilus_centrarchidarum|Canada|BOLD:ACM2700
ECTCR054-14|Ergasilus_centrarchidarum|Canada|BOLD:ACM2700
ECTCR055-14|Ergasilus_centrarchidarum|Canada|BOLD:ACM2700
ZOOPS071-18|Ergasilus_centrarchidarum|United_States|BOLD:ACM2700
ECTCR157-14|Ergasilus|Canada|BOLD:ACM2700
ECTCR009-14|Ergasilus_centrarchidarum|Canada|BOLD:ACM2700
ECTCR154-14|Ergasilus|Canada|BOLD:ACM2700
ECTCR158-14|Ergasilus|Canada|BOLD:ACM2700
ECTCR063-14|Thersitina_gasterostei|Canada|BOLD:ACM2886
ESPBC049-23|Ergasilidae|United_States|
ECTCR090-14|Ergasilus|Canada|BOLD:ACM2886
ECTCR185-14|Ergasilus|Canada|BOLD:ACM2886
ECTCR091-14|Ergasilus_auritus|Canada|BOLD:ACM2886
ESPBC047-23|Ergasilidae|United_States|BOLD:ACM2886
ESPBC048-23|Ergasilidae|United_States|BOLD:ACM2886
ESPBC046-23|Ergasilidae|United_States|BOLD:ACM2886
ECTCR067-14|Ergasilus|Canada|BOLD:ACM2886
ECTCR078-14|Ergasilus_luciopercarum|Canada|BOLD:ACM2662
2 2 2 2 2 2 2 2 2 2
8 8 8 8 8 8 8 8 8 8
105 105 105 105 105 105 105 105 105 105
19 19 19 19 19 19 19 19 19 19
9 9 9 9 9 9 9 9 9 9
11 11 11 11 11 11 11 11 11 11

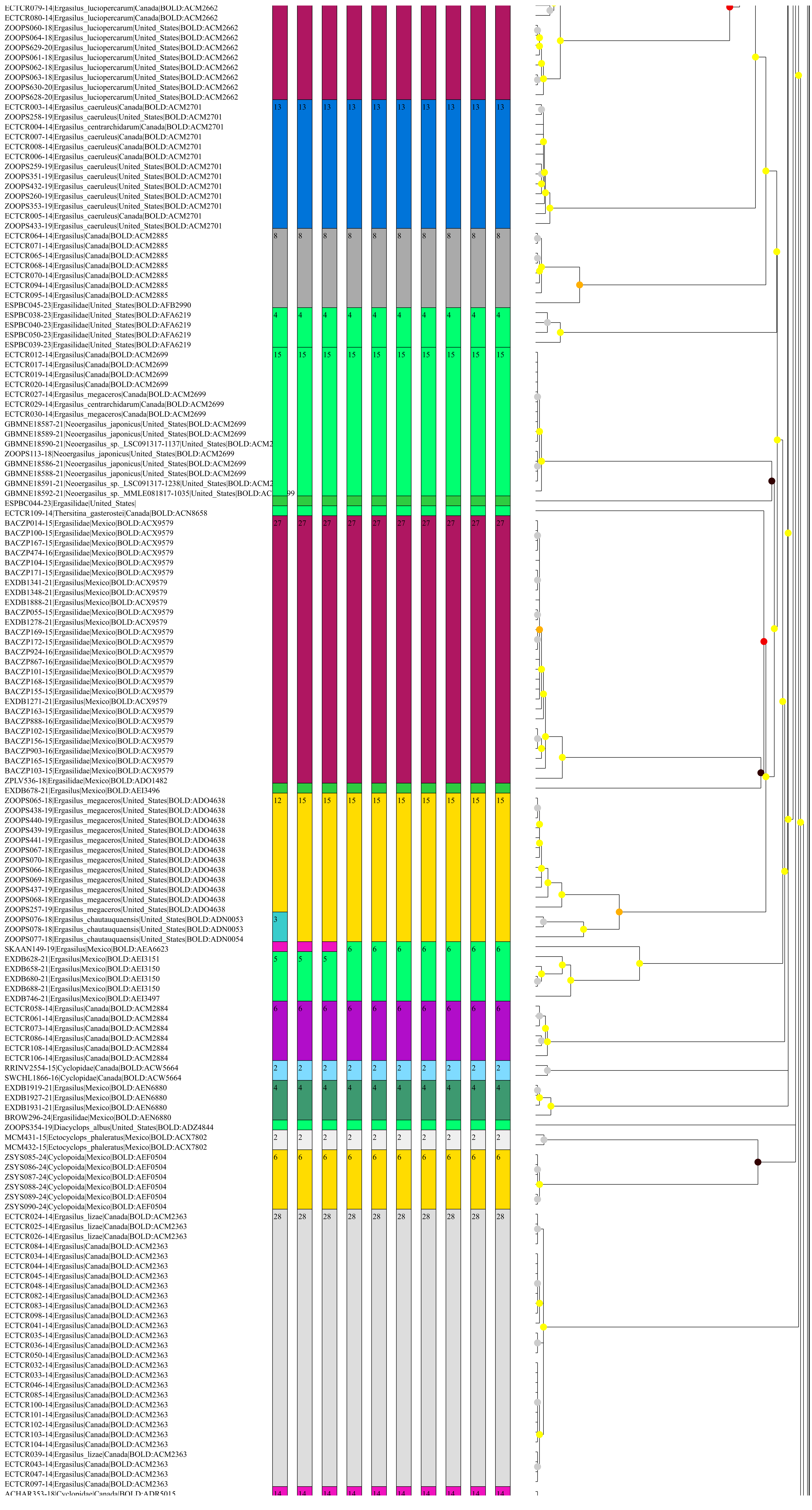
ECTCR079-14|Ergasilus_luciopercarum|Canada|BOLD:ACM2662
ECTCR080-14|Ergasilus_luciopercarum|Canada|BOLD:ACM2662
ZOOPS060-18|Ergasilus_luciopercarum|United_States|BOLD:ACM2662
ZOOPS064-18|Ergasilus_luciopercarum|United_States|BOLD:ACM2662
ZOOPS629-20|Ergasilus_luciopercarum|United_States|BOLD:ACM2662
ZOOPS061-18|Ergasilus_luciopercarum|United_States|BOLD:ACM2662
ZOOPS062-18|Ergasilus_luciopercarum|United_States|BOLD:ACM2662
ZOOPS063-18|Ergasilus_luciopercarum|United_States|BOLD:ACM2662
ZOOPS630-20|Ergasilus_luciopercarum|United_States|BOLD:ACM2662
ZOOPS628-20|Ergasilus_luciopercarum|United_States|BOLD:ACM2662
ECTCR003-14|Ergasilus_caeruleus|Canada|BOLD:ACM2701
ZOOPS258-19|Ergasilus_caeruleus|United_States|BOLD:ACM2701
ECTCR004-14|Ergasilus_centrarchidarum|Canada|BOLD:ACM2701
ECTCR007-14|Ergasilus_caeruleus|Canada|BOLD:ACM2701
ECTCR008-14|Ergasilus_caeruleus|Canada|BOLD:ACM2701
ECTCR006-14|Ergasilus_caeruleus|Canada|BOLD:ACM2701
ZOOPS259-19|Ergasilus_caeruleus|United_States|BOLD:ACM2701
ZOOPS351-19|Ergasilus_caeruleus|United_States|BOLD:ACM2701
ZOOPS432-19|Ergasilus_caeruleus|United_States|BOLD:ACM2701
ZOOPS260-19|Ergasilus_caeruleus|United_States|BOLD:ACM2701
ZOOPS353-19|Ergasilus_caeruleus|United_States|BOLD:ACM2701
ECTCR005-14|Ergasilus_caeruleus|Canada|BOLD:ACM2701
ZOOPS433-19|Ergasilus_caeruleus|United_States|BOLD:ACM2701
ECTCR064-14|Ergasilus|Canada|BOLD:ACM2885
ECTCR071-14|Ergasilus|Canada|BOLD:ACM2885
ECTCR065-14|Ergasilus|Canada|BOLD:ACM2885
ECTCR068-14|Ergasilus|Canada|BOLD:ACM2885
ECTCR070-14|Ergasilus|Canada|BOLD:ACM2885
ECTCR094-14|Ergasilus|Canada|BOLD:ACM2885
ECTCR095-14|Ergasilus|Canada|BOLD:ACM2885
ESPBC045-23|Ergasilidae|United_States|BOLD:AFB2990
ESPBC038-23|Ergasilidae|United_States|BOLD:AFA6219
ESPBC040-23|Ergasilidae|United_States|BOLD:AFA6219
ESPBC050-23|Ergasilidae|United_States|BOLD:AFA6219
ESPBC039-23|Ergasilidae|United_States|BOLD:AFA6219
ECTCR012-14|Ergasilus|Canada|BOLD:ACM2699
ECTCR017-14|Ergasilus|Canada|BOLD:ACM2699
ECTCR019-14|Ergasilus|Canada|BOLD:ACM2699
ECTCR020-14|Ergasilus|Canada|BOLD:ACM2699
ECTCR027-14|Ergasilus_megaceros|Canada|BOLD:ACM2699
ECTCR029-14|Ergasilus_centrarchidarum|Canada|BOLD:ACM2699
ECTCR030-14|Ergasilus_megaceros|Canada|BOLD:ACM2699
GBMNE18587-21|Neoergasilus_japonicus|United_States|BOLD:ACM2699
GBMNE18589-21|Neoergasilus_japonicus|United_States|BOLD:ACM2699
GBMNE18590-21|Neoergasilus_sp._LSC091317-1137|United_States|BOLD:ACM2
ZOOPS113-18|Neoergasilus_japonicus|United_States|BOLD:ACM2699
GBMNE18586-21|Neoergasilus_japonicus|United_States|BOLD:ACM2699
GBMNE18588-21|Neoergasilus_japonicus|United_States|BOLD:ACM2699
GBMNE18591-21|Neoergasilus_sp._LSC091317-1238|United_States|BOLD:ACM2
GBMNE18592-21|Neoergasilus_sp._MMLE081817-1035|United_States|BOLD:AC 99
ESPBC044-23|Ergasilidae|United_States|
ECTCR109-14|Thersitina_gasterostei|Canada|BOLD:ACN8658
BACZP014-15|Ergasilidae|Mexico|BOLD:ACX9579
BACZP100-15|Ergasilidae|Mexico|BOLD:ACX9579
BACZP167-15|Ergasilidae|Mexico|BOLD:ACX9579
BACZP474-16|Ergasilidae|Mexico|BOLD:ACX9579
BACZP104-15|Ergasilidae|Mexico|BOLD:ACX9579
BACZP171-15|Ergasilidae|Mexico|BOLD:ACX9579
EXDB1341-21|Ergasilus|Mexico|BOLD:ACX9579
EXDB1348-21|Ergasilus|Mexico|BOLD:ACX9579
EXDB1888-21|Ergasilus|Mexico|BOLD:ACX9579
BACZP055-15|Ergasilidae|Mexico|BOLD:ACX9579
EXDB1278-21|Ergasilus|Mexico|BOLD:ACX9579
BACZP169-15|Ergasilidae|Mexico|BOLD:ACX9579
BACZP172-15|Ergasilidae|Mexico|BOLD:ACX9579
BACZP924-16|Ergasilidae|Mexico|BOLD:ACX9579
BACZP867-16|Ergasilidae|Mexico|BOLD:ACX9579
BACZP101-15|Ergasilidae|Mexico|BOLD:ACX9579
BACZP168-15|Ergasilidae|Mexico|BOLD:ACX9579
BACZP155-15|Ergasilidae|Mexico|BOLD:ACX9579
EXDB1271-21|Ergasilus|Mexico|BOLD:ACX9579
BACZP163-15|Ergasilidae|Mexico|BOLD:ACX9579
BACZP888-16|Ergasilidae|Mexico|BOLD:ACX9579
BACZP102-15|Ergasilidae|Mexico|BOLD:ACX9579
BACZP156-15|Ergasilidae|Mexico|BOLD:ACX9579
BACZP903-16|Ergasilidae|Mexico|BOLD:ACX9579
BACZP165-15|Ergasilidae|Mexico|BOLD:ACX9579
BACZP103-15|Ergasilidae|Mexico|BOLD:ACX9579
ZPLV536-18|Ergasilidae|Mexico|BOLD:ADO1482
EXDB678-21|Ergasilus|Mexico|BOLD:AEI3496
ZOOPS065-18|Ergasilus_megaceros|United_States|BOLD:ADO4638
ZOOPS438-19|Ergasilus_megaceros|United_States|BOLD:ADO4638
ZOOPS440-19|Ergasilus_megaceros|United_States|BOLD:ADO4638
ZOOPS439-19|Ergasilus_megaceros|United_States|BOLD:ADO4638
ZOOPS441-19|Ergasilus_megaceros|United_States|BOLD:ADO4638
ZOOPS067-18|Ergasilus_megaceros|United_States|BOLD:ADO4638
ZOOPS070-18|Ergasilus_megaceros|United_States|BOLD:ADO4638
ZOOPS066-18|Ergasilus_megaceros|United_States|BOLD:ADO4638
ZOOPS069-18|Ergasilus_megaceros|United_States|BOLD:ADO4638
ZOOPS437-19|Ergasilus_megaceros|United_States|BOLD:ADO4638
ZOOPS068-18|Ergasilus_megaceros|United_States|BOLD:ADO4638
ZOOPS257-19|Ergasilus_megaceros|United_States|BOLD:ADO4638
ZOOPS076-18|Ergasilus_chautauquaensis|United_States|BOLD:ADN0053
ZOOPS078-18|Ergasilus_chautauquaensis|United_States|BOLD:ADN0053
ZOOPS077-18|Ergasilus_chautauquaensis|United_States|BOLD:ADN0054
SKAAN149-19|Ergasilus|Mexico|BOLD:AEA6623
EXDB628-21|Ergasilus|Mexico|BOLD:AEI3151
EXDB658-21|Ergasilus|Mexico|BOLD:AEI3150
EXDB680-21|Ergasilus|Mexico|BOLD:AEI3150
EXDB688-21|Ergasilus|Mexico|BOLD:AEI3150
EXDB746-21|Ergasilus|Mexico|BOLD:AEI3497
ECTCR058-14|Ergasilus|Canada|BOLD:ACM2884
ECTCR061-14|Ergasilus|Canada|BOLD:ACM2884
ECTCR073-14|Ergasilus|Canada|BOLD:ACM2884
ECTCR086-14|Ergasilus|Canada|BOLD:ACM2884
ECTCR108-14|Ergasilus|Canada|BOLD:ACM2884
ECTCR106-14|Ergasilus|Canada|BOLD:ACM2884
RRINV2554-15|Cyclopidae|Canada|BOLD:ACW5664
SWCHL1866-16|Cyclopidae|Canada|BOLD:ACW5664
EXDB1919-21|Ergasilus|Mexico|BOLD:AEN6880
EXDB1927-21|Ergasilus|Mexico|BOLD:AEN6880
EXDB1931-21|Ergasilus|Mexico|BOLD:AEN6880
BROW296-24|Ergasilidae|Mexico|BOLD:AEN6880
ZOOPS354-19|Diacyclops_albus|United_States|BOLD:ADZ4844
MCM431-15|Ectocyclops_phaleratus|Mexico|BOLD:ACX7802
MCM432-15|Ectocyclops_phaleratus|Mexico|BOLD:ACX7802
ZSYS085-24|Cyclopoida|Mexico|BOLD:AEF0504
ZSYS086-24|Cyclopoida|Mexico|BOLD:AEF0504
ZSYS087-24|Cyclopoida|Mexico|BOLD:AEF0504
ZSYS088-24|Cyclopoida|Mexico|BOLD:AEF0504
ZSYS089-24|Cyclopoida|Mexico|BOLD:AEF0504
ZSYS090-24|Cyclopoida|Mexico|BOLD:AEF0504
ECTCR024-14|Ergasilus_lizae|Canada|BOLD:ACM2363
ECTCR025-14|Ergasilus_lizae|Canada|BOLD:ACM2363
ECTCR026-14|Ergasilus_lizae|Canada|BOLD:ACM2363
ECTCR084-14|Ergasilus|Canada|BOLD:ACM2363
ECTCR034-14|Ergasilus|Canada|BOLD:ACM2363
ECTCR045-14|Ergasilus|Canada|BOLD:ACM2363
ECTCR044-14|Ergasilus|Canada|BOLD:ACM2363
ECTCR048-14|Ergasilus|Canada|BOLD:ACM2363
ECTCR082-14|Ergasilus|Canada|BOLD:ACM2363
ECTCR083-14|Ergasilus|Canada|BOLD:ACM2363
ECTCR098-14|Ergasilus|Canada|BOLD:ACM2363
ECTCR041-14|Ergasilus|Canada|BOLD:ACM2363
ECTCR035-14|Ergasilus|Canada|BOLD:ACM2363
ECTCR036-14|Ergasilus|Canada|BOLD:ACM2363
ECTCR050-14|Ergasilus|Canada|BOLD:ACM2363
ECTCR032-14|Ergasilus|Canada|BOLD:ACM2363
ECTCR033-14|Ergasilus|Canada|BOLD:ACM2363
ECTCR046-14|Ergasilus|Canada|BOLD:ACM2363
ECTCR085-14|Ergasilus|Canada|BOLD:ACM2363
ECTCR100-14|Ergasilus|Canada|BOLD:ACM2363
ECTCR101-14|Ergasilus|Canada|BOLD:ACM2363
ECTCR102-14|Ergasilus|Canada|BOLD:ACM2363
ECTCR103-14|Ergasilus|Canada|BOLD:ACM2363
ECTCR104-14|Ergasilus|Canada|BOLD:ACM2363
ECTCR039-14|Ergasilus_lizae|Canada|BOLD:ACM2363
ECTCR043-14|Ergasilus|Canada|BOLD:ACM2363
ECTCR047-14|Ergasilus|Canada|BOLD:ACM2363
ECTCR097-14|Ergasilus|Canada|BOLD:ACM2363
ACHAR353-18|Cyclopidae|Canada|BOLD:ADR5015

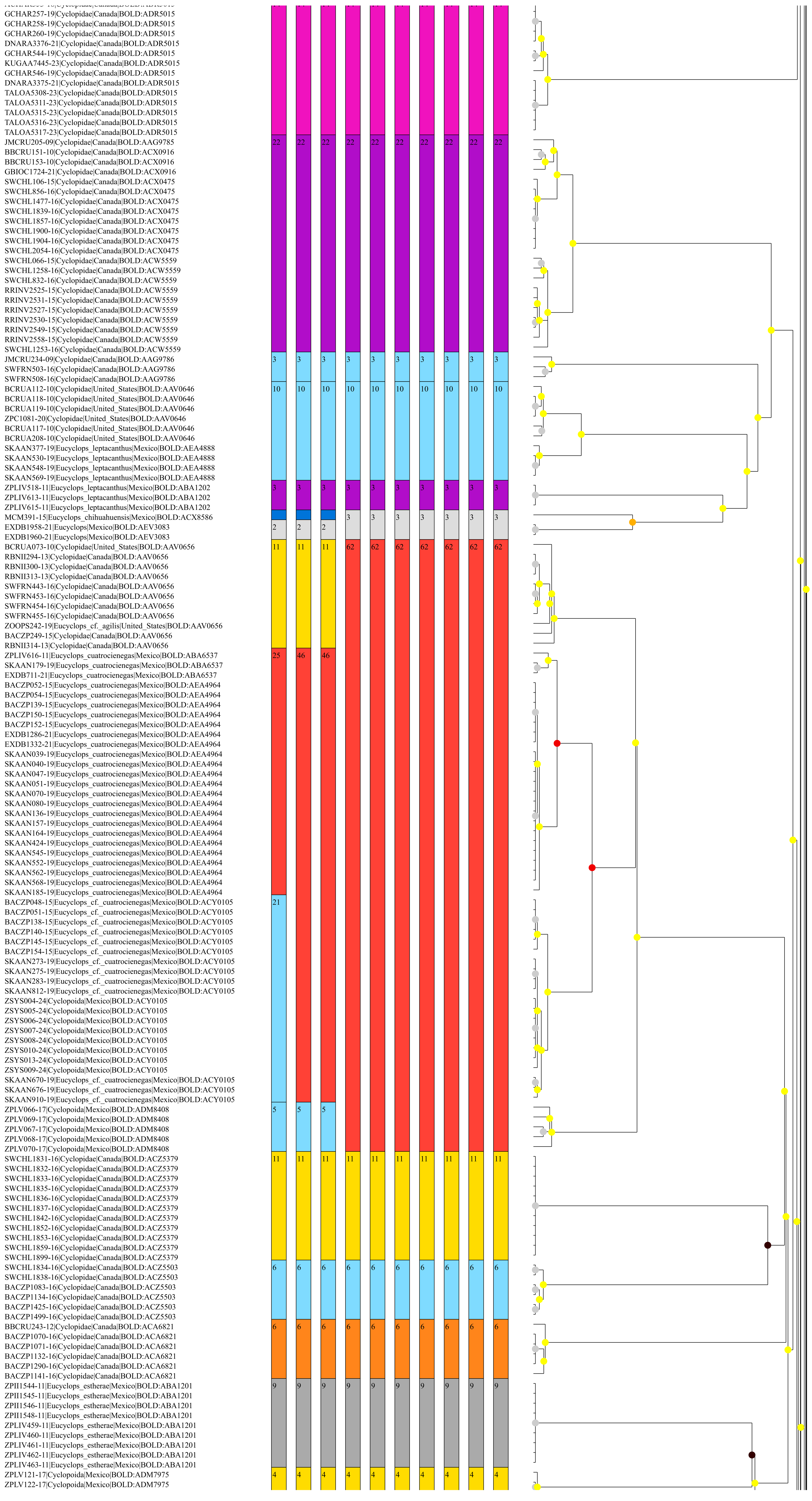
GCHAR257-19|Cyclopidae|Canada|BOLD:ADR5015
GCHAR258-19|Cyclopidae|Canada|BOLD:ADR5015
GCHAR260-19|Cyclopidae|Canada|BOLD:ADR5015
DNARA3376-21|Cyclopidae|Canada|BOLD:ADR5015
GCHAR544-19|Cyclopidae|Canada|BOLD:ADR5015
KUGAA7445-23|Cyclopidae|Canada|BOLD:ADR5015
GCHAR546-19|Cyclopidae|Canada|BOLD:ADR5015
DNARA3375-21|Cyclopidae|Canada|BOLD:ADR5015
TALOA5308-23|Cyclopidae|Canada|BOLD:ADR5015
TALOA5311-23|Cyclopidae|Canada|BOLD:ADR5015
TALOA5315-23|Cyclopidae|Canada|BOLD:ADR5015
TALOA5316-23|Cyclopidae|Canada|BOLD:ADR5015
TALOA5317-23|Cyclopidae|Canada|BOLD:ADR5015
JMCRU205-09|Cyclopidae|Canada|BOLD:AAG9785
BBCRU151-10|Cyclopidae|Canada|BOLD:ACX0916
BBCRU153-10|Cyclopidae|Canada|BOLD:ACX0916
GBIOC1724-21|Cyclopidae|Canada|BOLD:ACX0916
SWCHL106-15|Cyclopidae|Canada|BOLD:ACX0475
SWCHL856-16|Cyclopidae|Canada|BOLD:ACX0475
SWCHL1477-16|Cyclopidae|Canada|BOLD:ACX0475
SWCHL1839-16|Cyclopidae|Canada|BOLD:ACX0475
SWCHL1857-16|Cyclopidae|Canada|BOLD:ACX0475
SWCHL1900-16|Cyclopidae|Canada|BOLD:ACX0475
SWCHL1904-16|Cyclopidae|Canada|BOLD:ACX0475
SWCHL2054-16|Cyclopidae|Canada|BOLD:ACX0475
SWCHL066-15|Cyclopidae|Canada|BOLD:ACW5559
SWCHL1258-16|Cyclopidae|Canada|BOLD:ACW5559
SWCHL832-16|Cyclopidae|Canada|BOLD:ACW5559
RRINV2525-15|Cyclopidae|Canada|BOLD:ACW5559
RRINV2531-15|Cyclopidae|Canada|BOLD:ACW5559
RRINV2527-15|Cyclopidae|Canada|BOLD:ACW5559
RRINV2530-15|Cyclopidae|Canada|BOLD:ACW5559
RRINV2549-15|Cyclopidae|Canada|BOLD:ACW5559
RRINV2558-15|Cyclopidae|Canada|BOLD:ACW5559
SWCHL1253-16|Cyclopidae|Canada|BOLD:ACW5559
JMCRU234-09|Cyclopidae|Canada|BOLD:AAG9786
SWFRN503-16|Cyclopidae|Canada|BOLD:AAG9786
SWFRN508-16|Cyclopidae|Canada|BOLD:AAG9786
BCRUA112-10|Cyclopidae|United_States|BOLD:AAV0646
BCRUA118-10|Cyclopidae|United_States|BOLD:AAV0646
BCRUA119-10|Cyclopidae|United_States|BOLD:AAV0646
ZPC1081-20|Cyclopidae|United_States|BOLD:AAV0646
BCRUA117-10|Cyclopidae|United_States|BOLD:AAV0646
BCRUA208-10|Cyclopidae|United_States|BOLD:AAV0646
SKAAN377-19|Eucyclops_leptacanthus|Mexico|BOLD:AEA4888
SKAAN530-19|Eucyclops_leptacanthus|Mexico|BOLD:AEA4888
SKAAN548-19|Eucyclops_leptacanthus|Mexico|BOLD:AEA4888
SKAAN569-19|Eucyclops_leptacanthus|Mexico|BOLD:AEA4888
ZPLIV518-11|Eucyclops_leptacanthus|Mexico|BOLD:ABA1202
ZPLIV613-11|Eucyclops_leptacanthus|Mexico|BOLD:ABA1202
ZPLIV615-11|Eucyclops_leptacanthus|Mexico|BOLD:ABA1202
MCM391-15|Eucyclops_chihuahuensis|Mexico|BOLD:ACX8586
EXDB1958-21|Eucyclops|Mexico|BOLD:AEV3083
EXDB1960-21|Eucyclops|Mexico|BOLD:AEV3083
BCRUA073-10|Cyclopidae|United_States|BOLD:AAV0656
RBNII294-13|Cyclopidae|Canada|BOLD:AAV0656
RBNII300-13|Cyclopidae|Canada|BOLD:AAV0656
RBNII313-13|Cyclopidae|Canada|BOLD:AAV0656
SWFRN443-16|Cyclopidae|Canada|BOLD:AAV0656
SWFRN453-16|Cyclopidae|Canada|BOLD:AAV0656
SWFRN454-16|Cyclopidae|Canada|BOLD:AAV0656
SWFRN455-16|Cyclopidae|Canada|BOLD:AAV0656
ZOOPS242-19|Eucyclops_cf._agilis|United_States|BOLD:AAV0656
BACZP249-15|Cyclopidae|Canada|BOLD:AAV0656
RBNII314-13|Cyclopidae|Canada|BOLD:AAV0656
ZPLIV616-11|Eucyclops_cuatrocienegas|Mexico|BOLD:ABA6537
SKAAN179-19|Eucyclops_cuatrocienegas|Mexico|BOLD:ABA6537
EXDB711-21|Eucyclops_cuatrocienegas|Mexico|BOLD:ABA6537
BACZP052-15|Eucyclops_cuatrocienegas|Mexico|BOLD:AEA4964
BACZP054-15|Eucyclops_cuatrocienegas|Mexico|BOLD:AEA4964
BACZP139-15|Eucyclops_cuatrocienegas|Mexico|BOLD:AEA4964
BACZP150-15|Eucyclops_cuatrocienegas|Mexico|BOLD:AEA4964
BACZP152-15|Eucyclops_cuatrocienegas|Mexico|BOLD:AEA4964
EXDB1286-21|Eucyclops_cuatrocienegas|Mexico|BOLD:AEA4964
EXDB1332-21|Eucyclops_cuatrocienegas|Mexico|BOLD:AEA4964
SKAAN039-19|Eucyclops_cuatrocienegas|Mexico|BOLD:AEA4964
SKAAN040-19|Eucyclops_cuatrocienegas|Mexico|BOLD:AEA4964
SKAAN047-19|Eucyclops_cuatrocienegas|Mexico|BOLD:AEA4964
SKAAN051-19|Eucyclops_cuatrocienegas|Mexico|BOLD:AEA4964
SKAAN070-19|Eucyclops_cuatrocienegas|Mexico|BOLD:AEA4964
SKAAN080-19|Eucyclops_cuatrocienegas|Mexico|BOLD:AEA4964
SKAAN136-19|Eucyclops_cuatrocienegas|Mexico|BOLD:AEA4964
SKAAN157-19|Eucyclops_cuatrocienegas|Mexico|BOLD:AEA4964
SKAAN164-19|Eucyclops_cuatrocienegas|Mexico|BOLD:AEA4964
SKAAN424-19|Eucyclops_cuatrocienegas|Mexico|BOLD:AEA4964
SKAAN545-19|Eucyclops_cuatrocienegas|Mexico|BOLD:AEA4964
SKAAN552-19|Eucyclops_cuatrocienegas|Mexico|BOLD:AEA4964
SKAAN562-19|Eucyclops_cuatrocienegas|Mexico|BOLD:AEA4964
SKAAN568-19|Eucyclops_cuatrocienegas|Mexico|BOLD:AEA4964
SKAAN185-19|Eucyclops_cuatrocienegas|Mexico|BOLD:AEA4964
BACZP048-15|Eucyclops_cf._cuatrocienegas|Mexico|BOLD:ACY0105
BACZP051-15|Eucyclops_cf._cuatrocienegas|Mexico|BOLD:ACY0105
BACZP138-15|Eucyclops_cf._cuatrocienegas|Mexico|BOLD:ACY0105
BACZP140-15|Eucyclops_cf._cuatrocienegas|Mexico|BOLD:ACY0105
BACZP145-15|Eucyclops_cf._cuatrocienegas|Mexico|BOLD:ACY0105
BACZP154-15|Eucyclops_cf._cuatrocienegas|Mexico|BOLD:ACY0105
SKAAN273-19|Eucyclops_cf._cuatrocienegas|Mexico|BOLD:ACY0105
SKAAN275-19|Eucyclops_cf._cuatrocienegas|Mexico|BOLD:ACY0105
SKAAN283-19|Eucyclops_cf._cuatrocienegas|Mexico|BOLD:ACY0105
SKAAN812-19|Eucyclops_cf._cuatrocienegas|Mexico|BOLD:ACY0105
ZSYS004-24|Cyclopoida|Mexico|BOLD:ACY0105
ZSYS005-24|Cyclopoida|Mexico|BOLD:ACY0105
ZSYS006-24|Cyclopoida|Mexico|BOLD:ACY0105
ZSYS007-24|Cyclopoida|Mexico|BOLD:ACY0105
ZSYS008-24|Cyclopoida|Mexico|BOLD:ACY0105
ZSYS010-24|Cyclopoida|Mexico|BOLD:ACY0105
ZSYS013-24|Cyclopoida|Mexico|BOLD:ACY0105
ZSYS009-24|Cyclopoida|Mexico|BOLD:ACY0105
SKAAN670-19|Eucyclops_cf._cuatrocienegas|Mexico|BOLD:ACY0105
SKAAN676-19|Eucyclops_cf._cuatrocienegas|Mexico|BOLD:ACY0105
SKAAN910-19|Eucyclops_cf._cuatrocienegas|Mexico|BOLD:ACY0105
ZPLV066-17|Cyclopoida|Mexico|BOLD:ADM8408
ZPLV069-17|Cyclopoida|Mexico|BOLD:ADM8408
ZPLV067-17|Cyclopoida|Mexico|BOLD:ADM8408
ZPLV068-17|Cyclopoida|Mexico|BOLD:ADM8408
ZPLV070-17|Cyclopoida|Mexico|BOLD:ADM8408
SWCHL1831-16|Cyclopidae|Canada|BOLD:ACZ5379
SWCHL1832-16|Cyclopidae|Canada|BOLD:ACZ5379
SWCHL1833-16|Cyclopidae|Canada|BOLD:ACZ5379
SWCHL1835-16|Cyclopidae|Canada|BOLD:ACZ5379
SWCHL1836-16|Cyclopidae|Canada|BOLD:ACZ5379
SWCHL1837-16|Cyclopidae|Canada|BOLD:ACZ5379
SWCHL1842-16|Cyclopidae|Canada|BOLD:ACZ5379
SWCHL1852-16|Cyclopidae|Canada|BOLD:ACZ5379
SWCHL1853-16|Cyclopidae|Canada|BOLD:ACZ5379
SWCHL1859-16|Cyclopidae|Canada|BOLD:ACZ5379
SWCHL1899-16|Cyclopidae|Canada|BOLD:ACZ5379
SWCHL1834-16|Cyclopidae|Canada|BOLD:ACZ5503
SWCHL1838-16|Cyclopidae|Canada|BOLD:ACZ5503
BACZP1083-16|Cyclopidae|Canada|BOLD:ACZ5503
BACZP1134-16|Cyclopidae|Canada|BOLD:ACZ5503
BACZP1425-16|Cyclopidae|Canada|BOLD:ACZ5503
BACZP1499-16|Cyclopidae|Canada|BOLD:ACZ5503
BBCRU243-12|Cyclopidae|Canada|BOLD:ACA6821
BACZP1070-16|Cyclopidae|Canada|BOLD:ACA6821
BACZP1071-16|Cyclopidae|Canada|BOLD:ACA6821
BACZP1132-16|Cyclopidae|Canada|BOLD:ACA6821
BACZP1290-16|Cyclopidae|Canada|BOLD:ACA6821
BACZP1141-16|Cyclopidae|Canada|BOLD:ACA6821
ZPII1544-11|Eucyclops_estherae|Mexico|BOLD:ABA1201
ZPII1545-11|Eucyclops_estherae|Mexico|BOLD:ABA1201
ZPII1546-11|Eucyclops_estherae|Mexico|BOLD:ABA1201
ZPII1548-11|Eucyclops_estherae|Mexico|BOLD:ABA1201
ZPLIV459-11|Eucyclops_estherae|Mexico|BOLD:ABA1201
ZPLIV460-11|Eucyclops_estherae|Mexico|BOLD:ABA1201
ZPLIV461-11|Eucyclops_estherae|Mexico|BOLD:ABA1201
ZPLIV462-11|Eucyclops_estherae|Mexico|BOLD:ABA1201
ZPLIV463-11|Eucyclops_estherae|Mexico|BOLD:ABA1201
ZPLV121-17|Cyclopoida|Mexico|BOLD:ADM7975
ZPLV122-17|Cyclopoida|Mexico|BOLD:ADM7975

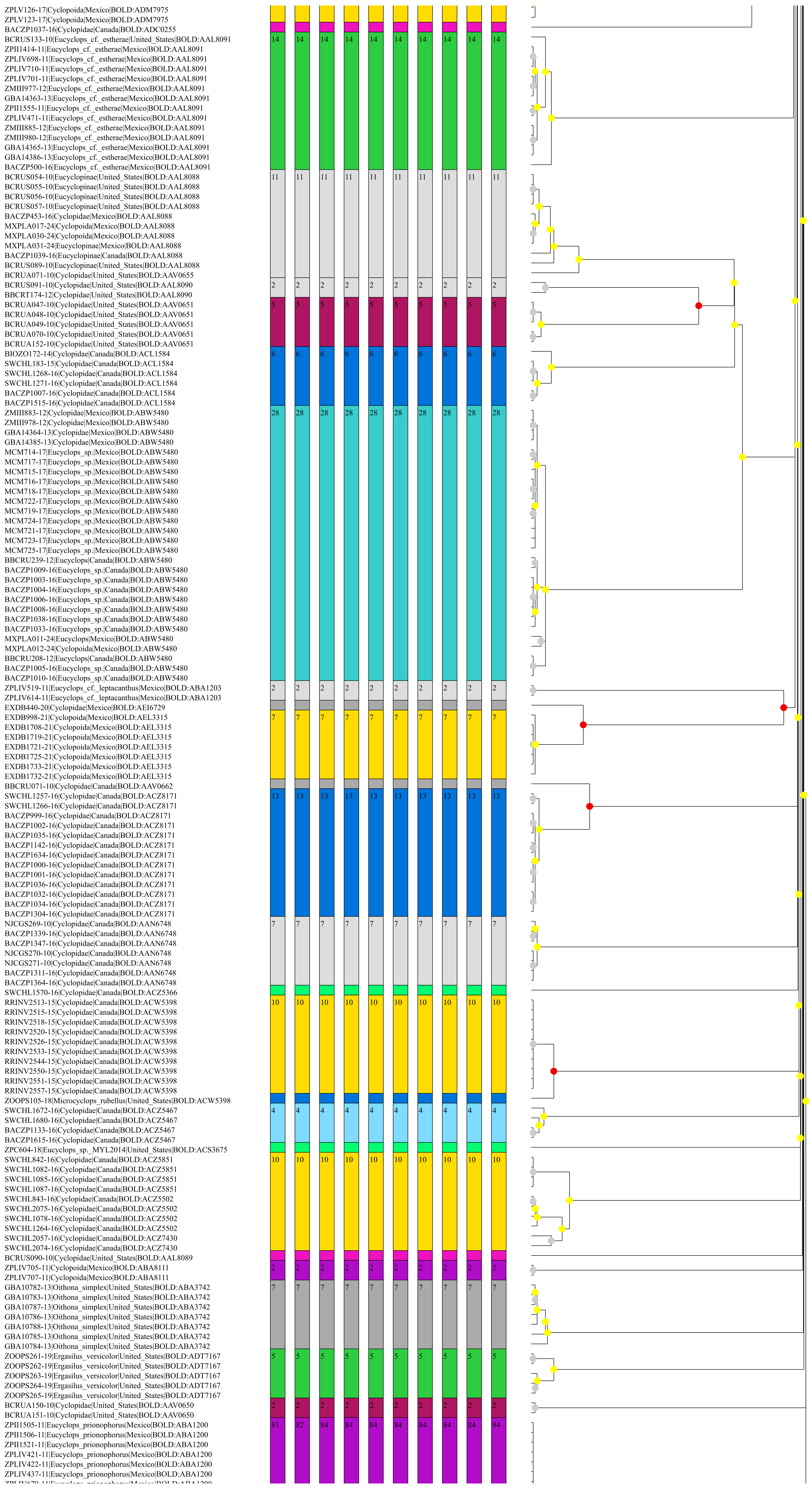
ZPLV126-17|Cyclopoida|Mexico|BOLD:ADM7975
ZPLV123-17|Cyclopoida|Mexico|BOLD:ADM7975
BACZP1037-16|Cyclopidae|Canada|BOLD:ADC0255
BCRUS133-10|Eucyclops_cf._estherae|United_States|BOLD:AAL8091
ZPII1414-11|Eucyclops_cf._estherae|Mexico|BOLD:AAL8091
ZPLIV698-11|Eucyclops_cf._estherae|Mexico|BOLD:AAL8091
ZPLIV710-11|Eucyclops_cf._estherae|Mexico|BOLD:AAL8091
ZPLIV701-11|Eucyclops_cf._estherae|Mexico|BOLD:AAL8091
ZMIII977-12|Eucyclops_cf._estherae|Mexico|BOLD:AAL8091
GBA14363-13|Eucyclops_cf._estherae|Mexico|BOLD:AAL8091
ZPII1555-11|Eucyclops_cf._estherae|Mexico|BOLD:AAL8091
ZPLIV471-11|Eucyclops_cf._estherae|Mexico|BOLD:AAL8091
ZMIII885-12|Eucyclops_cf._estherae|Mexico|BOLD:AAL8091
ZMIII980-12|Eucyclops_cf._estherae|Mexico|BOLD:AAL8091
GBA14365-13|Eucyclops_cf._estherae|Mexico|BOLD:AAL8091
GBA14386-13|Eucyclops_cf._estherae|Mexico|BOLD:AAL8091
BACZP500-16|Eucyclops_cf._estherae|Mexico|BOLD:AAL8091
BCRUS054-10|Eucyclopinae|United_States|BOLD:AAL8088
BCRUS055-10|Eucyclopinae|United_States|BOLD:AAL8088
BCRUS056-10|Eucyclopinae|United_States|BOLD:AAL8088
BCRUS057-10|Eucyclopinae|United_States|BOLD:AAL8088
BACZP453-16|Cyclopidae|Mexico|BOLD:AAL8088
MXPLA017-24|Cyclopoida|Mexico|BOLD:AAL8088
MXPLA030-24|Cyclopoida|Mexico|BOLD:AAL8088
MXPLA031-24|Eucyclopinae|Mexico|BOLD:AAL8088
BACZP1039-16|Eucyclopinae|Canada|BOLD:AAL8088
BCRUS089-10|Eucyclopinae|United_States|BOLD:AAL8088
BCRUA071-10|Cyclopidae|United_States|BOLD:AAV0655
BCRUS091-10|Cyclopidae|United_States|BOLD:AAL8090
BBCRT174-12|Cyclopidae|United_States|BOLD:AAL8090
BCRUA047-10|Cyclopidae|United_States|BOLD:AAV0651
BCRUA048-10|Cyclopidae|United_States|BOLD:AAV0651
BCRUA049-10|Cyclopidae|United_States|BOLD:AAV0651
BCRUA070-10|Cyclopidae|United_States|BOLD:AAV0651
BCRUA152-10|Cyclopidae|United_States|BOLD:AAV0651
BIOZO172-14|Cyclopidae|Canada|BOLD:ACL1584
SWCHL183-15|Cyclopidae|Canada|BOLD:ACL1584
SWCHL1268-16|Cyclopidae|Canada|BOLD:ACL1584
SWCHL1271-16|Cyclopidae|Canada|BOLD:ACL1584
BACZP1007-16|Cyclopidae|Canada|BOLD:ACL1584
BACZP1515-16|Cyclopidae|Canada|BOLD:ACL1584
ZMIII883-12|Cyclopidae|Mexico|BOLD:ABW5480
ZMIII978-12|Cyclopidae|Mexico|BOLD:ABW5480
GBA14364-13|Cyclopidae|Mexico|BOLD:ABW5480
GBA14385-13|Cyclopidae|Mexico|BOLD:ABW5480
MCM714-17|Eucyclops_sp.|Mexico|BOLD:ABW5480
MCM717-17|Eucyclops_sp.|Mexico|BOLD:ABW5480
MCM715-17|Eucyclops_sp.|Mexico|BOLD:ABW5480
MCM716-17|Eucyclops_sp.|Mexico|BOLD:ABW5480
MCM718-17|Eucyclops_sp.|Mexico|BOLD:ABW5480
MCM722-17|Eucyclops_sp.|Mexico|BOLD:ABW5480
MCM719-17|Eucyclops_sp.|Mexico|BOLD:ABW5480
MCM724-17|Eucyclops_sp.|Mexico|BOLD:ABW5480
MCM721-17|Eucyclops_sp.|Mexico|BOLD:ABW5480
MCM723-17|Eucyclops_sp.|Mexico|BOLD:ABW5480
MCM725-17|Eucyclops_sp.|Mexico|BOLD:ABW5480
BBCRU239-12|Eucyclops|Canada|BOLD:ABW5480
BACZP1009-16|Eucyclops_sp.|Canada|BOLD:ABW5480
BACZP1003-16|Eucyclops_sp.|Canada|BOLD:ABW5480
BACZP1004-16|Eucyclops_sp.|Canada|BOLD:ABW5480
BACZP1006-16|Eucyclops_sp.|Canada|BOLD:ABW5480
BACZP1008-16|Eucyclops_sp.|Canada|BOLD:ABW5480
BACZP1038-16|Eucyclops_sp.|Canada|BOLD:ABW5480
BACZP1033-16|Eucyclops_sp.|Canada|BOLD:ABW5480
MXPLA011-24|Eucyclops|Mexico|BOLD:ABW5480
MXPLA012-24|Cyclopoida|Mexico|BOLD:ABW5480
BBCRU208-12|Eucyclops|Canada|BOLD:ABW5480
BACZP1005-16|Eucyclops_sp.|Canada|BOLD:ABW5480
BACZP1010-16|Eucyclops_sp.|Canada|BOLD:ABW5480
ZPLIV519-11|Eucyclops_cf._leptacanthus|Mexico|BOLD:ABA1203
ZPLIV614-11|Eucyclops_cf._leptacanthus|Mexico|BOLD:ABA1203
EXDB440-20|Cyclopidae|Mexico|BOLD:AEI6729
EXDB998-21|Cyclopoida|Mexico|BOLD:AEL3315
EXDB1708-21|Cyclopoida|Mexico|BOLD:AEL3315
EXDB1719-21|Cyclopoida|Mexico|BOLD:AEL3315
EXDB1721-21|Cyclopoida|Mexico|BOLD:AEL3315
EXDB1725-21|Cyclopoida|Mexico|BOLD:AEL3315
EXDB1733-21|Cyclopoida|Mexico|BOLD:AEL3315
EXDB1732-21|Cyclopoida|Mexico|BOLD:AEL3315
BBCRU071-10|Cyclopidae|Canada|BOLD:AAV0662
SWCHL1257-16|Cyclopidae|Canada|BOLD:ACZ8171
SWCHL1266-16|Cyclopidae|Canada|BOLD:ACZ8171
BACZP999-16|Cyclopidae|Canada|BOLD:ACZ8171
BACZP1002-16|Cyclopidae|Canada|BOLD:ACZ8171
BACZP1035-16|Cyclopidae|Canada|BOLD:ACZ8171
BACZP1142-16|Cyclopidae|Canada|BOLD:ACZ8171
BACZP1634-16|Cyclopidae|Canada|BOLD:ACZ8171
BACZP1000-16|Cyclopidae|Canada|BOLD:ACZ8171
BACZP1001-16|Cyclopidae|Canada|BOLD:ACZ8171
BACZP1036-16|Cyclopidae|Canada|BOLD:ACZ8171
BACZP1032-16|Cyclopidae|Canada|BOLD:ACZ8171
BACZP1034-16|Cyclopidae|Canada|BOLD:ACZ8171
BACZP1304-16|Cyclopidae|Canada|BOLD:ACZ8171
NJCGS269-10|Cyclopidae|Canada|BOLD:AAN6748
BACZP1339-16|Cyclopidae|Canada|BOLD:AAN6748
BACZP1347-16|Cyclopidae|Canada|BOLD:AAN6748
NJCGS270-10|Cyclopidae|Canada|BOLD:AAN6748
NJCGS271-10|Cyclopidae|Canada|BOLD:AAN6748
BACZP1311-16|Cyclopidae|Canada|BOLD:AAN6748
BACZP1364-16|Cyclopidae|Canada|BOLD:AAN6748
SWCHL1570-16|Cyclopidae|Canada|BOLD:ACZ5366
RRINV2513-15|Cyclopidae|Canada|BOLD:ACW5398
RRINV2515-15|Cyclopidae|Canada|BOLD:ACW5398
RRINV2518-15|Cyclopidae|Canada|BOLD:ACW5398
RRINV2520-15|Cyclopidae|Canada|BOLD:ACW5398
RRINV2526-15|Cyclopidae|Canada|BOLD:ACW5398
RRINV2533-15|Cyclopidae|Canada|BOLD:ACW5398
RRINV2544-15|Cyclopidae|Canada|BOLD:ACW5398
RRINV2550-15|Cyclopidae|Canada|BOLD:ACW5398
RRINV2551-15|Cyclopidae|Canada|BOLD:ACW5398
RRINV2557-15|Cyclopidae|Canada|BOLD:ACW5398
ZOOPS105-18|Microcyclops_rubellus|United_States|BOLD:ACW5398
SWCHL1672-16|Cyclopidae|Canada|BOLD:ACZ5467
SWCHL1680-16|Cyclopidae|Canada|BOLD:ACZ5467
BACZP1133-16|Cyclopidae|Canada|BOLD:ACZ5467
BACZP1615-16|Cyclopidae|Canada|BOLD:ACZ5467
ZPC604-18|Eucyclops_sp._MYL2014|United_States|BOLD:ACS3675
SWCHL842-16|Cyclopidae|Canada|BOLD:ACZ5851
SWCHL1082-16|Cyclopidae|Canada|BOLD:ACZ5851
SWCHL1085-16|Cyclopidae|Canada|BOLD:ACZ5851
SWCHL1087-16|Cyclopidae|Canada|BOLD:ACZ5851
SWCHL843-16|Cyclopidae|Canada|BOLD:ACZ5502
SWCHL2075-16|Cyclopidae|Canada|BOLD:ACZ5502
SWCHL1078-16|Cyclopidae|Canada|BOLD:ACZ5502
SWCHL1264-16|Cyclopidae|Canada|BOLD:ACZ5502
SWCHL2057-16|Cyclopidae|Canada|BOLD:ACZ7430
SWCHL2074-16|Cyclopidae|Canada|BOLD:ACZ7430
BCRUS090-10|Cyclopidae|United_States|BOLD:AAL8089
ZPLIV705-11|Cyclopoida|Mexico|BOLD:ABA8111
ZPLIV707-11|Cyclopoida|Mexico|BOLD:ABA8111
GBA10782-13|Oithona_simplex|United_States|BOLD:ABA3742
GBA10783-13|Oithona_simplex|United_States|BOLD:ABA3742
GBA10787-13|Oithona_simplex|United_States|BOLD:ABA3742
GBA10786-13|Oithona_simplex|United_States|BOLD:ABA3742
GBA10788-13|Oithona_simplex|United_States|BOLD:ABA3742
GBA10785-13|Oithona_simplex|United_States|BOLD:ABA3742
GBA10784-13|Oithona_simplex|United_States|BOLD:ABA3742
ZOOPS261-19|Ergasilus_versicolor|United_States|BOLD:ADT7167
ZOOPS262-19|Ergasilus_versicolor|United_States|BOLD:ADT7167
ZOOPS263-19|Ergasilus_versicolor|United_States|BOLD:ADT7167
ZOOPS264-19|Ergasilus_versicolor|United_States|BOLD:ADT7167
ZOOPS265-19|Ergasilus_versicolor|United_States|BOLD:ADT7167
BCRUA150-10|Cyclopidae|United_States|BOLD:AAV0650
BCRUA151-10|Cyclopidae|United_States|BOLD:AAV0650
ZPII1505-11|Eucyclops_prionophorus|Mexico|BOLD:ABA1200
ZPII1506-11|Eucyclops_prionophorus|Mexico|BOLD:ABA1200
ZPII1521-11|Eucyclops_prionophorus|Mexico|BOLD:ABA1200
ZPLIV421-11|Eucyclops_prionophorus|Mexico|BOLD:ABA1200
ZPLIV422-11|Eucyclops_prionophorus|Mexico|BOLD:ABA1200
ZPLIV437-11|Eucyclops_prionophorus|Mexico|BOLD:ABA1200

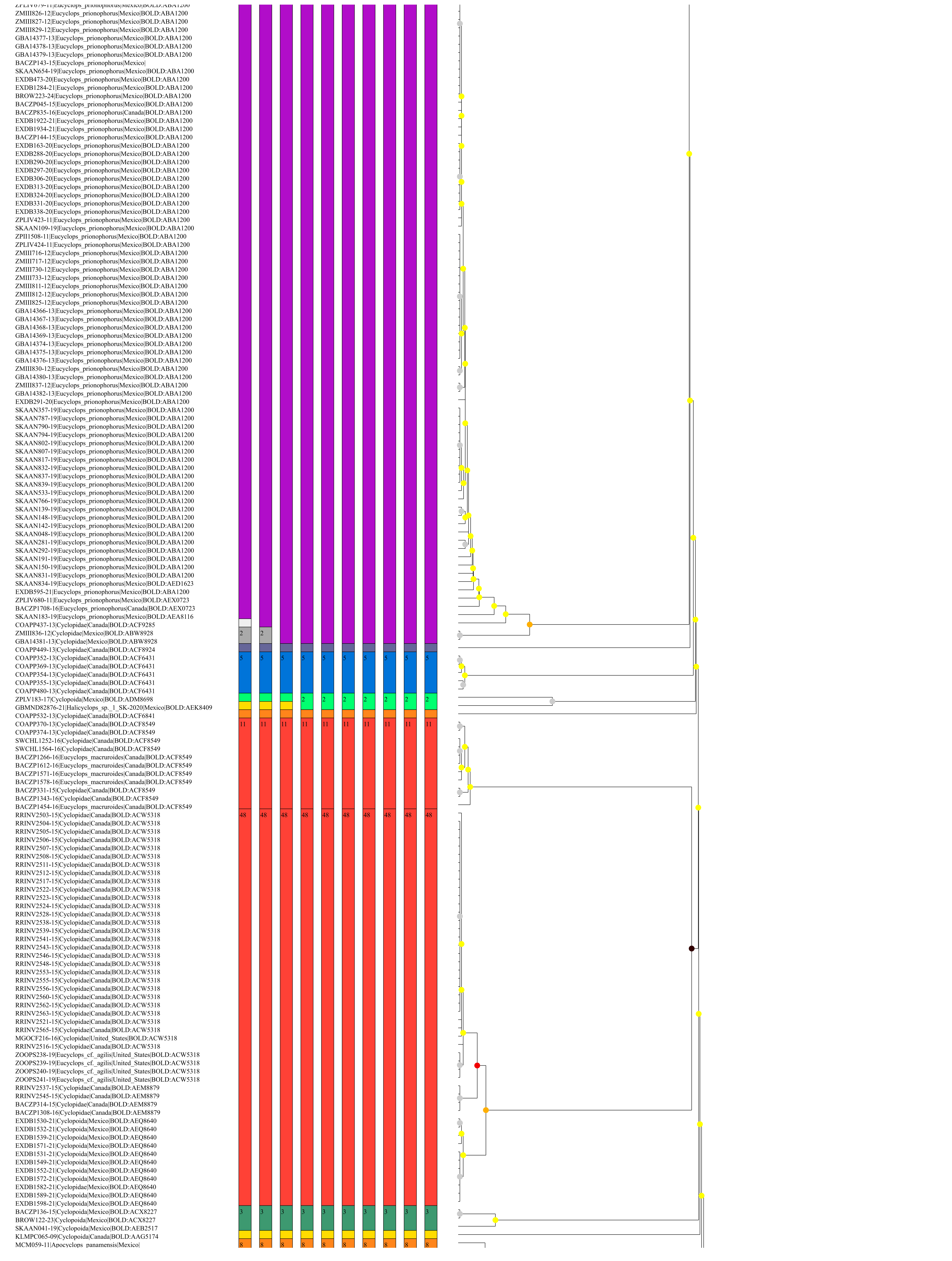
ZMIII826-12|Eucyclops_prionophorus|Mexico|BOLD:ABA1200
ZMIII827-12|Eucyclops_prionophorus|Mexico|BOLD:ABA1200
ZMIII829-12|Eucyclops_prionophorus|Mexico|BOLD:ABA1200
GBA14377-13|Eucyclops_prionophorus|Mexico|BOLD:ABA1200
GBA14378-13|Eucyclops_prionophorus|Mexico|BOLD:ABA1200
GBA14379-13|Eucyclops_prionophorus|Mexico|BOLD:ABA1200
BACZP143-15|Eucyclops_prionophorus|Mexico|
SKAAN654-19|Eucyclops_prionophorus|Mexico|BOLD:ABA1200
EXDB473-20|Eucyclops_prionophorus|Mexico|BOLD:ABA1200
EXDB1284-21|Eucyclops_prionophorus|Mexico|BOLD:ABA1200
BROW223-24|Eucyclops_prionophorus|Mexico|BOLD:ABA1200
BACZP045-15|Eucyclops_prionophorus|Mexico|BOLD:ABA1200
BACZP835-16|Eucyclops_prionophorus|Canada|BOLD:ABA1200
EXDB1922-21|Eucyclops_prionophorus|Mexico|BOLD:ABA1200
EXDB1934-21|Eucyclops_prionophorus|Mexico|BOLD:ABA1200
BACZP144-15|Eucyclops_prionophorus|Mexico|BOLD:ABA1200
EXDB163-20|Eucyclops_prionophorus|Mexico|BOLD:ABA1200
EXDB288-20|Eucyclops_prionophorus|Mexico|BOLD:ABA1200
EXDB290-20|Eucyclops_prionophorus|Mexico|BOLD:ABA1200
EXDB297-20|Eucyclops_prionophorus|Mexico|BOLD:ABA1200
EXDB306-20|Eucyclops_prionophorus|Mexico|BOLD:ABA1200
EXDB313-20|Eucyclops_prionophorus|Mexico|BOLD:ABA1200
EXDB324-20|Eucyclops_prionophorus|Mexico|BOLD:ABA1200
EXDB331-20|Eucyclops_prionophorus|Mexico|BOLD:ABA1200
EXDB338-20|Eucyclops_prionophorus|Mexico|BOLD:ABA1200
ZPLIV423-11|Eucyclops_prionophorus|Mexico|BOLD:ABA1200
SKAAN109-19|Eucyclops_prionophorus|Mexico|BOLD:ABA1200
ZPII1508-11|Eucyclops_prionophorus|Mexico|BOLD:ABA1200
ZPLIV424-11|Eucyclops_prionophorus|Mexico|BOLD:ABA1200
ZMIII716-12|Eucyclops_prionophorus|Mexico|BOLD:ABA1200
ZMIII717-12|Eucyclops_prionophorus|Mexico|BOLD:ABA1200
ZMIII730-12|Eucyclops_prionophorus|Mexico|BOLD:ABA1200
ZMIII733-12|Eucyclops_prionophorus|Mexico|BOLD:ABA1200
ZMIII811-12|Eucyclops_prionophorus|Mexico|BOLD:ABA1200
ZMIII812-12|Eucyclops_prionophorus|Mexico|BOLD:ABA1200
ZMIII825-12|Eucyclops_prionophorus|Mexico|BOLD:ABA1200
GBA14366-13|Eucyclops_prionophorus|Mexico|BOLD:ABA1200
GBA14367-13|Eucyclops_prionophorus|Mexico|BOLD:ABA1200
GBA14368-13|Eucyclops_prionophorus|Mexico|BOLD:ABA1200
GBA14369-13|Eucyclops_prionophorus|Mexico|BOLD:ABA1200
GBA14374-13|Eucyclops_prionophorus|Mexico|BOLD:ABA1200
GBA14375-13|Eucyclops_prionophorus|Mexico|BOLD:ABA1200
GBA14376-13|Eucyclops_prionophorus|Mexico|BOLD:ABA1200
ZMIII830-12|Eucyclops_prionophorus|Mexico|BOLD:ABA1200
GBA14380-13|Eucyclops_prionophorus|Mexico|BOLD:ABA1200
ZMIII837-12|Eucyclops_prionophorus|Mexico|BOLD:ABA1200
GBA14382-13|Eucyclops_prionophorus|Mexico|BOLD:ABA1200
EXDB291-20|Eucyclops_prionophorus|Mexico|BOLD:ABA1200
SKAAN357-19|Eucyclops_prionophorus|Mexico|BOLD:ABA1200
SKAAN787-19|Eucyclops_prionophorus|Mexico|BOLD:ABA1200
SKAAN790-19|Eucyclops_prionophorus|Mexico|BOLD:ABA1200
SKAAN794-19|Eucyclops_prionophorus|Mexico|BOLD:ABA1200
SKAAN802-19|Eucyclops_prionophorus|Mexico|BOLD:ABA1200
SKAAN807-19|Eucyclops_prionophorus|Mexico|BOLD:ABA1200
SKAAN817-19|Eucyclops_prionophorus|Mexico|BOLD:ABA1200
SKAAN832-19|Eucyclops_prionophorus|Mexico|BOLD:ABA1200
SKAAN837-19|Eucyclops_prionophorus|Mexico|BOLD:ABA1200
SKAAN839-19|Eucyclops_prionophorus|Mexico|BOLD:ABA1200
SKAAN533-19|Eucyclops_prionophorus|Mexico|BOLD:ABA1200
SKAAN766-19|Eucyclops_prionophorus|Mexico|BOLD:ABA1200
SKAAN139-19|Eucyclops_prionophorus|Mexico|BOLD:ABA1200
SKAAN148-19|Eucyclops_prionophorus|Mexico|BOLD:ABA1200
SKAAN142-19|Eucyclops_prionophorus|Mexico|BOLD:ABA1200
SKAAN048-19|Eucyclops_prionophorus|Mexico|BOLD:ABA1200
SKAAN281-19|Eucyclops_prionophorus|Mexico|BOLD:ABA1200
SKAAN292-19|Eucyclops_prionophorus|Mexico|BOLD:ABA1200
SKAAN191-19|Eucyclops_prionophorus|Mexico|BOLD:ABA1200
SKAAN150-19|Eucyclops_prionophorus|Mexico|BOLD:ABA1200
SKAAN831-19|Eucyclops_prionophorus|Mexico|BOLD:ABA1200
SKAAN834-19|Eucyclops_prionophorus|Mexico|BOLD:AED1623
EXDB595-21|Eucyclops_prionophorus|Mexico|BOLD:ABA1200
ZPLIV680-11|Eucyclops_prionophorus|Mexico|BOLD:AEX0723
BACZP1708-16|Eucyclops_prionophorus|Canada|BOLD:AEX0723
SKAAN183-19|Eucyclops_prionophorus|Mexico|BOLD:AEA8116
COAPP437-13|Cyclopidae|Canada|BOLD:ACF9285
ZMIII836-12|Cyclopidae|Mexico|BOLD:ABW8928
GBA14381-13|Cyclopidae|Mexico|BOLD:ABW8928
COAPP449-13|Cyclopidae|Canada|BOLD:ACF8924
COAPP352-13|Cyclopidae|Canada|BOLD:ACF6431
COAPP369-13|Cyclopidae|Canada|BOLD:ACF6431
COAPP354-13|Cyclopidae|Canada|BOLD:ACF6431
COAPP355-13|Cyclopidae|Canada|BOLD:ACF6431
COAPP480-13|Cyclopidae|Canada|BOLD:ACF6431
ZPLV183-17|Cyclopoida|Mexico|BOLD:ADM8698
GBMND82876-21|Halicyclops_sp._1_SK-2020|Mexico|BOLD:AEK8409
COAPP532-13|Cyclopidae|Canada|BOLD:ACF6841
COAPP370-13|Cyclopidae|Canada|BOLD:ACF8549
COAPP374-13|Cyclopidae|Canada|BOLD:ACF8549
SWCHL1252-16|Cyclopidae|Canada|BOLD:ACF8549
SWCHL1564-16|Cyclopidae|Canada|BOLD:ACF8549
BACZP1266-16|Eucyclops_macruroides|Canada|BOLD:ACF8549
BACZP1612-16|Eucyclops_macruroides|Canada|BOLD:ACF8549
BACZP1571-16|Eucyclops_macruroides|Canada|BOLD:ACF8549
BACZP1578-16|Eucyclops_macruroides|Canada|BOLD:ACF8549
BACZP331-15|Cyclopidae|Canada|BOLD:ACF8549
BACZP1343-16|Cyclopidae|Canada|BOLD:ACF8549
BACZP1454-16|Eucyclops_macruroides|Canada|BOLD:ACF8549
RRINV2503-15|Cyclopidae|Canada|BOLD:ACW5318
RRINV2504-15|Cyclopidae|Canada|BOLD:ACW5318
RRINV2505-15|Cyclopidae|Canada|BOLD:ACW5318
RRINV2506-15|Cyclopidae|Canada|BOLD:ACW5318
RRINV2507-15|Cyclopidae|Canada|BOLD:ACW5318
RRINV2508-15|Cyclopidae|Canada|BOLD:ACW5318
RRINV2511-15|Cyclopidae|Canada|BOLD:ACW5318
RRINV2512-15|Cyclopidae|Canada|BOLD:ACW5318
RRINV2517-15|Cyclopidae|Canada|BOLD:ACW5318
RRINV2522-15|Cyclopidae|Canada|BOLD:ACW5318
RRINV2523-15|Cyclopidae|Canada|BOLD:ACW5318
RRINV2524-15|Cyclopidae|Canada|BOLD:ACW5318
RRINV2528-15|Cyclopidae|Canada|BOLD:ACW5318
RRINV2538-15|Cyclopidae|Canada|BOLD:ACW5318
RRINV2539-15|Cyclopidae|Canada|BOLD:ACW5318
RRINV2541-15|Cyclopidae|Canada|BOLD:ACW5318
RRINV2543-15|Cyclopidae|Canada|BOLD:ACW5318
RRINV2546-15|Cyclopidae|Canada|BOLD:ACW5318
RRINV2548-15|Cyclopidae|Canada|BOLD:ACW5318
RRINV2553-15|Cyclopidae|Canada|BOLD:ACW5318
RRINV2555-15|Cyclopidae|Canada|BOLD:ACW5318
RRINV2556-15|Cyclopidae|Canada|BOLD:ACW5318
RRINV2560-15|Cyclopidae|Canada|BOLD:ACW5318
RRINV2562-15|Cyclopidae|Canada|BOLD:ACW5318
RRINV2563-15|Cyclopidae|Canada|BOLD:ACW5318
RRINV2565-15|Cyclopidae|Canada|BOLD:ACW5318
RRINV2521-15|Cyclopidae|Canada|BOLD:ACW5318
RRINV2565-15|Cyclopidae|Canada|BOLD:ACW5318
MGOCF216-16|Cyclopidae|United_States|BOLD:ACW5318
RRINV2516-15|Cyclopidae|Canada|BOLD:ACW5318
ZOOPS238-19|Eucyclops_cf._agilis|United_States|BOLD:ACW5318
ZOOPS239-19|Eucyclops_cf._agilis|United_States|BOLD:ACW5318
ZOOPS240-19|Eucyclops_cf._agilis|United_States|BOLD:ACW5318
ZOOPS241-19|Eucyclops_cf._agilis|United_States|BOLD:ACW5318
RRINV2537-15|Cyclopidae|Canada|BOLD:AEM8879
RRINV2545-15|Cyclopidae|Canada|BOLD:AEM8879
BACZP314-15|Cyclopidae|Canada|BOLD:AEM8879
BACZP1308-16|Cyclopidae|Canada|BOLD:AEM8879
EXDB1530-21|Cyclopoida|Mexico|BOLD:AEQ8640
EXDB1532-21|Cyclopoida|Mexico|BOLD:AEQ8640
EXDB1539-21|Cyclopoida|Mexico|BOLD:AEQ8640
EXDB1571-21|Cyclopoida|Mexico|BOLD:AEQ8640
EXDB1531-21|Cyclopoida|Mexico|BOLD:AEQ8640
EXDB1549-21|Cyclopoida|Mexico|BOLD:AEQ8640
EXDB1552-21|Cyclopoida|Mexico|BOLD:AEQ8640
EXDB1572-21|Cyclopoida|Mexico|BOLD:AEQ8640
EXDB1582-21|Cyclopidae|Mexico|BOLD:AEQ8640
EXDB1589-21|Cyclopoida|Mexico|BOLD:AEQ8640
EXDB1598-21|Cyclopoida|Mexico|BOLD:AEQ8640
BACZP136-15|Cyclopoida|Mexico|BOLD:ACX8227
BROW122-23|Cyclopoida|Mexico|BOLD:ACX8227
SKAAN041-19|Cyclopoida|Mexico|BOLD:AEB2517
KLMPC065-09|Cyclopoida|Canada|BOLD:AAG5174
MCM059-11|Apocyclops_panamensis|Mexico|

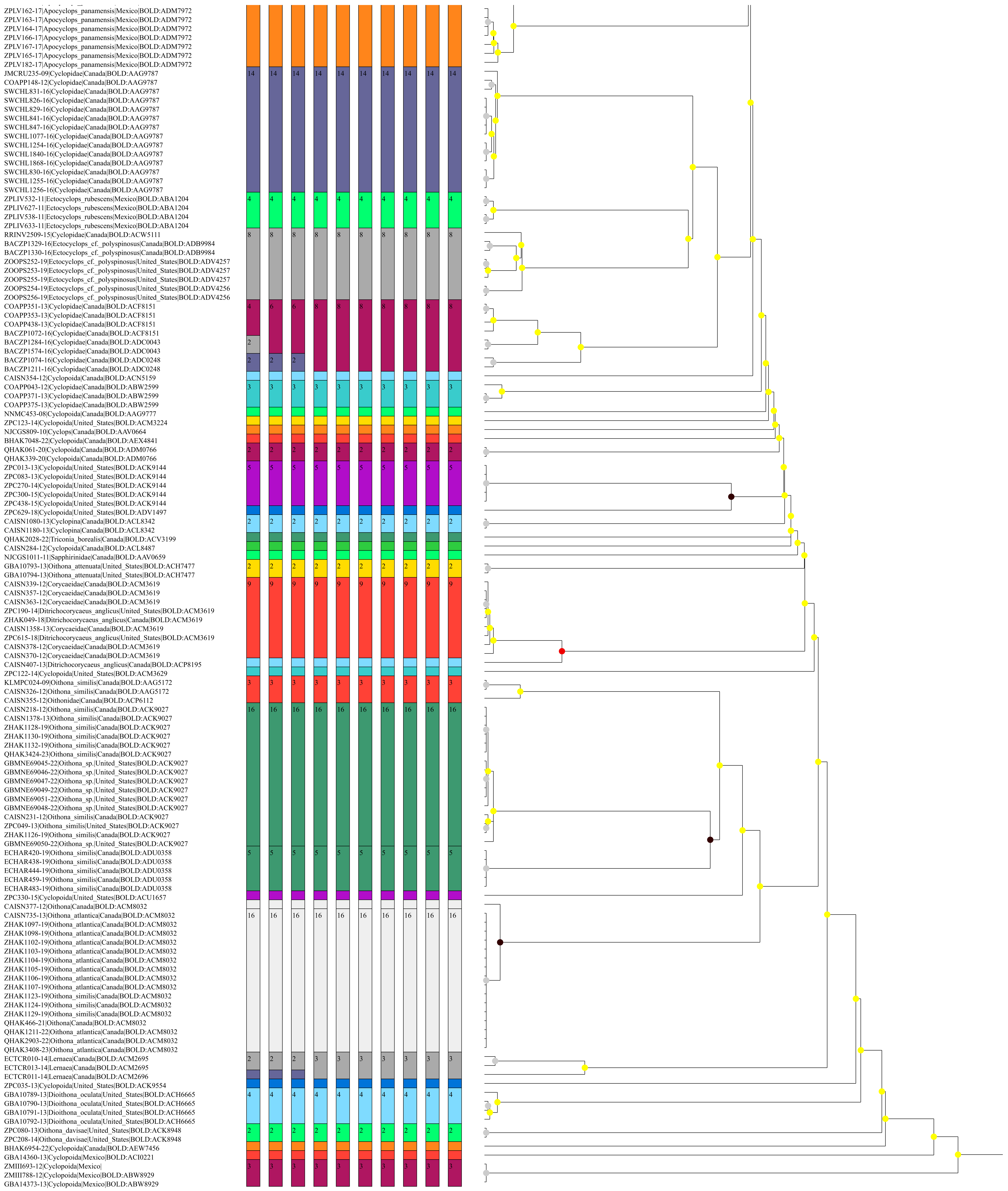
ZPLV162-17|Apocyclops_panamensis|Mexico|BOLD:ADM7972
ZPLV163-17|Apocyclops_panamensis|Mexico|BOLD:ADM7972
ZPLV164-17|Apocyclops_panamensis|Mexico|BOLD:ADM7972
ZPLV166-17|Apocyclops_panamensis|Mexico|BOLD:ADM7972
ZPLV167-17|Apocyclops_panamensis|Mexico|BOLD:ADM7972
ZPLV165-17|Apocyclops_panamensis|Mexico|BOLD:ADM7972
ZPLV182-17|Apocyclops_panamensis|Mexico|BOLD:ADM7972
JMCRU235-09|Cyclopidae|Canada|BOLD:AAG9787
COAPP148-12|Cyclopidae|Canada|BOLD:AAG9787
SWCHL831-16|Cyclopidae|Canada|BOLD:AAG9787
SWCHL826-16|Cyclopidae|Canada|BOLD:AAG9787
SWCHL829-16|Cyclopidae|Canada|BOLD:AAG9787
SWCHL841-16|Cyclopidae|Canada|BOLD:AAG9787
SWCHL847-16|Cyclopidae|Canada|BOLD:AAG9787
SWCHL1077-16|Cyclopidae|Canada|BOLD:AAG9787
SWCHL1254-16|Cyclopidae|Canada|BOLD:AAG9787
SWCHL1840-16|Cyclopidae|Canada|BOLD:AAG9787
SWCHL1868-16|Cyclopidae|Canada|BOLD:AAG9787
SWCHL830-16|Cyclopidae|Canada|BOLD:AAG9787
SWCHL1255-16|Cyclopidae|Canada|BOLD:AAG9787
SWCHL1256-16|Cyclopidae|Canada|BOLD:AAG9787
ZPLIV532-11|Ectocyclops_rubescens|Mexico|BOLD:ABA1204
ZPLIV627-11|Ectocyclops_rubescens|Mexico|BOLD:ABA1204
ZPLIV538-11|Ectocyclops_rubescens|Mexico|BOLD:ABA1204
ZPLIV633-11|Ectocyclops_rubescens|Mexico|BOLD:ABA1204
RRINV2509-15|Cyclopidae|Canada|BOLD:ACW5111
BACZP1329-16|Ectocyclops_cf._polyspinosus|Canada|BOLD:ADB9984
BACZP1330-16|Ectocyclops_cf._polyspinosus|Canada|BOLD:ADB9984
ZOOPS252-19|Ectocyclops_cf._polyspinosus|United_States|BOLD:ADV4257
ZOOPS253-19|Ectocyclops_cf._polyspinosus|United_States|BOLD:ADV4257
ZOOPS255-19|Ectocyclops_cf._polyspinosus|United_States|BOLD:ADV4257
ZOOPS254-19|Ectocyclops_cf._polyspinosus|United_States|BOLD:ADV4256
ZOOPS256-19|Ectocyclops_cf._polyspinosus|United_States|BOLD:ADV4256
COAPP351-13|Cyclopidae|Canada|BOLD:ACF8151
COAPP353-13|Cyclopidae|Canada|BOLD:ACF8151
COAPP438-13|Cyclopidae|Canada|BOLD:ACF8151
BACZP1072-16|Cyclopidae|Canada|BOLD:ACF8151
BACZP1284-16|Cyclopidae|Canada|BOLD:ADC0043
BACZP1574-16|Cyclopidae|Canada|BOLD:ADC0043
BACZP1074-16|Cyclopidae|Canada|BOLD:ADC0248
BACZP1211-16|Cyclopidae|Canada|BOLD:ADC0248
CAISN354-12|Cyclopoida|Canada|BOLD:ACN5159
COAPP043-12|Cyclopidae|Canada|BOLD:ABW2599
COAPP371-13|Cyclopidae|Canada|BOLD:ABW2599
COAPP375-13|Cyclopidae|Canada|BOLD:ABW2599
NNMC453-08|Cyclopoida|Canada|BOLD:AAG9777
ZPC123-14|Cyclopoida|United_States|BOLD:ACM3224
NJCGS809-10|Cyclops|Canada|BOLD:AAV0664
BHAK7048-22|Cyclopoida|Canada|BOLD:AEX4841
QHAK061-20|Cyclopoida|Canada|BOLD:ADM0766
QHAK339-20|Cyclopoida|Canada|BOLD:ADM0766
ZPC013-13|Cyclopoida|United_States|BOLD:ACK9144
ZPC083-13|Cyclopoida|United_States|BOLD:ACK9144
ZPC270-14|Cyclopoida|United_States|BOLD:ACK9144
ZPC300-15|Cyclopoida|United_States|BOLD:ACK9144
ZPC438-15|Cyclopoida|United_States|BOLD:ACK9144
ZPC629-18|Cyclopoida|United_States|BOLD:ADV1497
CAISN1080-13|Cyclopina|Canada|BOLD:ACL8342
CAISN1180-13|Cyclopina|Canada|BOLD:ACL8342
QHAK2028-22|Triconia_borealis|Canada|BOLD:ACV3199
CAISN284-12|Cyclopoida|Canada|BOLD:ACL8487
NJCGS1011-11|Sapphirinidae|Canada|BOLD:AAV0659
GBA10793-13|Oithona_attenuata|United_States|BOLD:ACH7477
GBA10794-13|Oithona_attenuata|United_States|BOLD:ACH7477
CAISN339-12|Corycaeidae|Canada|BOLD:ACM3619
CAISN357-12|Corycaeidae|Canada|BOLD:ACM3619
CAISN363-12|Corycaeidae|Canada|BOLD:ACM3619
ZPC190-14|Ditrichocorycaeus_anglicus|United_States|BOLD:ACM3619
ZHAK049-18|Ditrichocorycaeus_anglicus|Canada|BOLD:ACM3619
CAISN1358-13|Corycaeidae|Canada|BOLD:ACM3619
ZPC615-18|Ditrichocorycaeus_anglicus|United_States|BOLD:ACM3619
CAISN378-12|Corycaeidae|Canada|BOLD:ACM3619
CAISN370-12|Corycaeidae|Canada|BOLD:ACM3619
CAISN407-13|Ditrichocorycaeus_anglicus|Canada|BOLD:ACP8195
ZPC122-14|Cyclopoida|United_States|BOLD:ACM3629
KLMPC024-09|Oithona_similis|Canada|BOLD:AAG5172
CAISN326-12|Oithona_similis|Canada|BOLD:AAG5172
CAISN355-12|Oithonidae|Canada|BOLD:ACP6112
CAISN218-12|Oithona_similis|Canada|BOLD:ACK9027
CAISN1378-13|Oithona_similis|Canada|BOLD:ACK9027
ZHAK1128-19|Oithona_similis|Canada|BOLD:ACK9027
ZHAK1130-19|Oithona_similis|Canada|BOLD:ACK9027
ZHAK1132-19|Oithona_similis|Canada|BOLD:ACK9027
QHAK3424-23|Oithona_similis|Canada|BOLD:ACK9027
GBMNE69045-22|Oithona_sp.|United_States|BOLD:ACK9027
GBMNE69046-22|Oithona_sp.|United_States|BOLD:ACK9027
GBMNE69047-22|Oithona_sp.|United_States|BOLD:ACK9027
GBMNE69049-22|Oithona_sp.|United_States|BOLD:ACK9027
GBMNE69048-22|Oithona_sp.|United_States|BOLD:ACK9027
GBMNE69051-22|Oithona_sp.|United_States|BOLD:ACK9027
CAISN231-12|Oithona_similis|Canada|BOLD:ACK9027
ZPC049-13|Oithona_similis|United_States|BOLD:ACK9027
ZHAK1126-19|Oithona_similis|Canada|BOLD:ACK9027
GBMNE69050-22|Oithona_sp.|United_States|BOLD:ACK9027
ECHAR420-19|Oithona_similis|Canada|BOLD:ADU0358
ECHAR438-19|Oithona_similis|Canada|BOLD:ADU0358
ECHAR444-19|Oithona_similis|Canada|BOLD:ADU0358
ECHAR459-19|Oithona_similis|Canada|BOLD:ADU0358
ECHAR483-19|Oithona_similis|Canada|BOLD:ADU0358
ZPC330-15|Cyclopoida|United_States|BOLD:ACU1657
CAISN377-12|Oithona|Canada|BOLD:ACM8032
CAISN735-13|Oithona_atlantica|Canada|BOLD:ACM8032
ZHAK1097-19|Oithona_atlantica|Canada|BOLD:ACM8032
ZHAK1098-19|Oithona_atlantica|Canada|BOLD:ACM8032
ZHAK1102-19|Oithona_atlantica|Canada|BOLD:ACM8032
ZHAK1103-19|Oithona_atlantica|Canada|BOLD:ACM8032
ZHAK1104-19|Oithona_atlantica|Canada|BOLD:ACM8032
ZHAK1105-19|Oithona_atlantica|Canada|BOLD:ACM8032
ZHAK1106-19|Oithona_atlantica|Canada|BOLD:ACM8032
ZHAK1107-19|Oithona_atlantica|Canada|BOLD:ACM8032
ZHAK1123-19|Oithona_similis|Canada|BOLD:ACM8032
ZHAK1124-19|Oithona_similis|Canada|BOLD:ACM8032
ZHAK1129-19|Oithona_similis|Canada|BOLD:ACM8032
QHAK466-21|Oithona|Canada|BOLD:ACM8032
QHAK1211-22|Oithona_atlantica|Canada|BOLD:ACM8032
QHAK2903-22|Oithona_atlantica|Canada|BOLD:ACM8032
QHAK3408-23|Oithona_atlantica|Canada|BOLD:ACM8032
ECTCR010-14|Lernaea|Canada|BOLD:ACM2695
ECTCR013-14|Lernaea|Canada|BOLD:ACM2695
ECTCR011-14|Lernaea|Canada|BOLD:ACM2696
ZPC035-13|Cyclopoida|United_States|BOLD:ACK9554
GBA10789-13|Dioithona_oculata|United_States|BOLD:ACH6665
GBA10790-13|Dioithona_oculata|United_States|BOLD:ACH6665
GBA10791-13|Dioithona_oculata|United_States|BOLD:ACH6665
GBA10792-13|Dioithona_oculata|United_States|BOLD:ACH6665
ZPC080-13|Oithona_davisae|United_States|BOLD:ACK8948
ZPC208-14|Oithona_davisae|United_States|BOLD:ACK8948
BHAK6954-22|Cyclopoida|Canada|BOLD:AEW7456
GBA14360-13|Cyclopoida|Mexico|BOLD:ACI0221
ZMIII693-12|Cyclopoida|Mexico|
ZMIII788-12|Cyclopoida|Mexico|BOLD:ABW8929
GBA14373-13|Cyclopoida|Mexico|BOLD:ABW8929
