# Supplementary material for: DNA barcoding of North American freshwater copepods (Diaptomidae and Cyclopoida): an overview after 20 years with emphasis in the Mexican fauna, the transition between the Nearctic and Neotropics
Source: PeerJ. 2026 Apr 9;14:e20989. doi: 10.7717/peerj.20989 (PMC13070316; doi:10.7717/peerj.20989)
Supplement: Supplemental Information 9 [file peerj-14-20989-s009.docx]

| Dataset | Raw data in Dataset (specimens) | Filters used for NJ-tree in BOLD | Number of sequences included in the NJ-tree | Number of sequences after alignment in MEGA, included in ASAP (574 bp) |
| --- | --- | --- | --- | --- |
| DS-DIAPTOMID | 1717 | Lenght>500 bp only  Exclude misidentifications  Exclude stop codons  Exclude contaminants | 1701 | 1696 |
| DS-CYCLOPNA | 2291 | Lenght>500 bp only  Exclude misidentifications  Exclude stop codons  Exclude contaminants | 2269 | 2265 |
